# Trueness and Precision of Four Intraoral Scanners in Oral Implantology: A Comparative *in Vitro* Study

**DOI:** 10.1371/journal.pone.0163107

**Published:** 2016-09-29

**Authors:** Francesco G. Mangano, Giovanni Veronesi, Uli Hauschild, Eitan Mijiritsky, Carlo Mangano

**Affiliations:** 1 Department of Surgical and Morphological Science, Dental School, Insubria University, Varese, Italy; 2 Academic Unit of Digital Dentistry, IRCCS S. Raffaele Hospital, Milan, Italy; 3 Department of Clinical and Experimental Medicine, Medical School, Insubria University, Varese, Italy; 4 Freelance Researcher, Sanremo, Italy; 5 Department of Oral Rehabilitation, The Maurice and Gabriela Goldschleger School of Dental Medicine, University of Tel Aviv, Ramat Tel Aviv, Israel; Second University of Naples, ITALY

## Abstract

**Purpose:**

The aim of this study was to compare the trueness and precision of four intraoral scanners used in oral implantology.

**Methods:**

Two stone models were prepared, representing a partially and a totally edentulous maxilla, with three and six implant analogues, respectively, and polyether-ether-ketone (PEEK) cylinders screwed on. The models were digitized with an industrial scanner (IScan D104I®) used as a reference, and with four intraoral scanners (Trios®; CS 3500®; Zfx Intrascan®; Planscan®). Five scans were taken for each model, using each different intraoral scanner. All datasets were loaded into reverse-engineering software (Geomagics 2012®), where intraoral scans were superimposed on the reference model, to evaluate general trueness, and superimposed on each other within groups, to evaluate general precision. General trueness and precision of any scanner were compared by model type, through an ANOVA model including scanner, model and their interaction. Finally, the distance and angles between simulated implants were measured in each group, and compared to those of the reference model, to evaluate local trueness.

**Results:**

In the partially edentulous maxilla, CS 3500® had the best general trueness (47.8 μm) and precision (40.8 μm), followed by Trios® (trueness 71.2 μm, precision 51.0 μm), Zfx Intrascan® (trueness 117.0 μm, precision 126.2 μm), and Planscan® (trueness 233.4 μm, precision 219.8 μm). With regard to general trueness, Trios® was significantly better than Planscan®, CS 3500® was significantly better than Zfx Intrascan® and Planscan®, and Zfx Intrascan® was significantly better than Planscan®; with regard to general precision, Trios® was significantly better than Zfx Intrascan® and Planscan®, CS 3500® was significantly better than Zfx Intrascan® and Planscan®, and Zfx Intrascan® was significantly better than Planscan®. In the totally edentulous maxilla, CS 3500® had the best performance in terms of general trueness (63.2 μm) and precision (55.2 μm), followed by Trios® (trueness 71.6 μm, precision 67.0 μm), Zfx Intrascan® (trueness 103.0 μm, precision 112.4 μm), and Planscan® (trueness 253.4 μm, precision 204.2 μm). With regard to general trueness, Trios® was significantly better than Planscan®, CS 3500® was significantly better than Zfx Intrascan® and Planscan®, and Zfx Intrascan® was significantly better than Planscan®; with regard to general precision, Trios® was significantly better than Zfx Intrascan® and Planscan®, CS 3500® was significantly better than Zfx Intrascan® and Planscan®, and Zfx Intrascan® was significantly better than Planscan®. Local trueness values confirmed these results.

**Conclusions:**

Although no differences in trueness and precision were found between partially and totally edentulous models, statistically significant differences were found between the different scanners. Further studies are required to confirm these results.

## Introduction

In recent years, several intraoral scanners have been introduced into the market, and an increasing number of dental clinics have decided to adopt these powerful devices for capturing digital impressions [1–3]. Intraoral scanners allow the capturing of digital impressions of the dental arches using only a light beam, without the need of individual trays and materials (alginate, silicone, polyether) that are traditionally used to take impressions [2–4]. Conventional impressions are generally not appreciated by patients: they represent an unpleasant procedure, especially for those with a pronounced gag reflex [4,5]. The possibility to effectively replace conventional impressions is the main advantage of intraoral digital impressions, which results in a reduction of the costs for materials [6–8]. Other advantages are the immediate control of the quality of the impression, and the possibility of obtaining three-dimensional models (3D) which can be electronically sent to the laboratory, saving time and money; finally, digital impressions can act as powerful marketing tools for patients [6–10].

Although capturing a digital impression is rather simple for the clinician, the working mechanism of intraoral scanners is rather complex [2,3]. The scanner projects a light beam (laser or structured light) onto the surfaces to be analyzed; the deformation that the light undergoes on such surfaces is captured by two or more cameras, and exploited for the calculation of 3D coordinates, with the aid of powerful processing software [2,3,11]. This software generates point clouds and meshes, and is therefore responsible for 3D reconstruction of the scanned surfaces. Since the scanner software collects and processes thousands of frames per second, it is of fundamental importance that the stored images are assembled correctly, in order to obtain a reliable model [2,3,11,12].

Beyond the purely clinical and operational aspects (ease of use, speed, size of the tip, etc.), the main features that an intraoral scanner should possess in order to capture high-quality impressions are trueness and precision [1–3,11,12]. These terms have a well-established mathematical meaning, and cannot be used as synonyms [12,13]. Trueness is defined as the ability of a measurement to match the actual value of the quantity being measured, while precision is defined as the ability of a measurement to be consistently repeated [12,13]. Ideally, an intraoral scanner should possess high trueness (it should be capable of matching reality as closely as possible), but also high precision (it should be able to consistently replicate results, obtaining the same measurement each time) [12,13]. In order to evaluate the trueness of an intraoral scanner, a reference measurement/ model of the same scanned object, obtained with powerful industrial equipment (coordinate measuring machines/ articulated arms or industrial optical scanners with accuracy < 5 μm), is required; with reverse-engineering software, the intraoral scan will be superimposed on the reference model, in order to mathematically evaluate deviations between measurements [13,14]. On the other hand, to evaluate precision, the simple superposition of different scans obtained with the same intraoral scanner is performed [13,14].

Several *in vitro* studies have shown that intraoral scanners can capture impressions of sufficient quality, compatible with the fabrication of simple (inlays, onlays, single crowns) to complex (fixed partial prostheses) restorations, in dentate patients [15–17]. These findings have been confirmed by a series of clinical studies [18–22].

However, few studies have compared the trueness and precision of different intraoral scanners [23–27]. These studies mostly report on first-generation scanners, and do not deal with the most powerful and recent devices: the scientific literature struggles to keep up with the industry [23–27]. In addition, to date, only a few studies have investigated the ability of intraoral scanners to capture high-quality impressions in patients with dental implants [27,28]. Taking such impressions is a complex procedure [27,28]. Despite the introduction of specific non-reflective polyether-ether-ketone (PEEK) devices (scanbodies) for transferring the correct implant position, in fact, edentulous areas can be difficult to read and mathematically interpret for intraoral scanners [27–30].

Hence, the aim of this study was to compare the trueness and precision of four modern intraoral scanners, in two different situations: in a partially edentulous maxilla with three implants, and in a totally edentulous maxilla with six implants.

## Materials and Methods

### The models

Nine high-precision PEEK scanbodies were prepared: this material was chosen for its optical properties since, unlike titanium cylinders, PEEK does not reflect light [30]. It is well known that intraoral scanners may have difficulties scanning reflective, shiny surfaces [30]. Then, two different stone models were prepared, representing different clinical situations. The first model represented a partially edentulous maxilla, with implant analogues (BTK implants®, Dueville, Vicenza, Italy) in positions 21, 24 and 26, with three high-precision PEEK cylinders screwed on. The second model represented a totally edentulous maxilla, with the same implant analogues in positions 16, 14, 11, 21, 24 and 26, and six high-precision PEEK cylinders screwed on.

### Study design

Four intraoral scanner systems (Trios® 2, 3-Shape, Copenhagen, Denmark; CS 3500®, Carestream Health, Rochester, NY, US; Zfx Intrascan®, MHT S.p.A., Verona, Italy; and Planmeca Planscan®, E4D Technologies, LLC, Richardson, TX, USA), as well as a powerful reference scanner (IScan D104I®, Imetric3D GmbH, Courgenay, Switzerland) were used in the present study. The IScan D104I is a 3D-structured light scanner, which provides the option of scanning an entire arch in less than 3 minutes. The manufacturer reports for this scanner a trueness of < 5 μm and a precision of <10 μm, at temperatures between 15–30°C. In the present study, all scans were made under the same conditions (in the same room with a temperature of 20°, humidity of 45%, and air pressure of 760 ± 5 mmHg); the same dentist with long experience in using intraoral scanners performed all scans. The scans proceeded in the following order. First, the two stone models (partially and a totally edentulous maxilla, respectively) were scanned with the reference scanner; three scans were taken for each model. For each model, all generated datasets were imported into powerful reverse-engineering software (Studio 2012®, Geomagic, Morrisville, NC, USA) and superimposed on each other, in order to validate the manufacturer’s data. One dataset for each model was then selected as the reference dataset (R1) for the trueness measurements of all intraoral scanners. Second, the two stone models were scanned with the four intraoral scanners. After calibration, scans (n = 5) were taken for each model, using each different device. The sequence of scans was the result of randomization, in order to reduce the potential effects of operator fatigue; the scans were taken sequentially, with an interval of 10 minutes, in order to allow the operator to rest and the device to cool down. A specific scanning technique was followed for all intraoral devices; In brief, starting from the first quadrant (superior right), the tip of the scanner draw an arc movement, from vestibular to palatal and back, slowly moving forward so that teeth, scanbodies and gingiva were scanned from vestibular to palatal (and back), passing over the occlusal plane.

#### Trios

Trios® 2 (3-Shape, Copenhagen, Denmark) is a powerful and fast structured light scanner, working under the principle of confocal microscopy and ultrafast optical scanning. It does not require opacization of the model (it is powder-free), and it produces in-colour images. The latter is an interesting aspect, since colour scanning can help to differentiate the natural tooth structure and the gingival tissues, and therefore it may help dentists to identify the margin lines. The software version 2014–1 (release 1.3.3.1) was used here. Trios® has a big wand, and this helps to avoid scanning of unwanted tissues, such as tongue, cheeks or lips; obviously, these tissues can be digitally removed during the impression, in real time, using proprietary software. Trios® is available in cart and pod solution: the latter allows the clinician to use a laptop, into which the scanner is plugged via a USB port, even if the connection is not direct (many connecting cables are required). Trios® produces proprietary (.DCM) files, which can be opened only by the 3-Shape computer-assisted-design (CAD) system. Since the scanner does not automatically allow conversion or export of these proprietary files into common solid-to-layer (.STL) files, readable from all CAD systems, Trios® is defined as a closed system. Finally, the 3-Shape CAD software can be used to design several kinds of restorations and frameworks (crowns, bridges, inlays, onlays, veneers, bars), although the company does not have a dedicated milling machine for in-office restoration.

#### CS 3500

CS 3500® (Carestream Health, Rochester, NY, US) is a powder-free intraoral scanner that allows prosthodontists to scan patients’ teeth and obtain in-colour 3D images. CS 3500® works under the principle of single image acquisition, therefore the acquisition can be slower than with other scanners; the wand is not big, therefore sufficient overlapping of the single images (≥ 50% of the previous image) is important. Software version 2016–4 (release 2.1.4.10) was used here. CS 3500® produces proprietary files (.CSZ) that can be immediately converted into (.STL) files, therefore it works as an open system: as files can be opened by different CAD software available on the market, the data can be virtually sent to any laboratory in the world. Finally, Carestream has a proprietary software and milling unit for designing and fabrication of inlays, onlays, crowns, short-span bridges and veneers: a complete in-office digital workflow is possible. CS 3500® can be easily plugged into a laptop via a USB port.

#### ZFX Intrascan

ZFX Intrascan® (powered by MHT S.p.A., Verona, Italy) is an intraoral scanner working under the principle of confocal microscopy and the Moireè effect. It uses a red laser and although it can be considered a powder-free scanner, it does not produce in-colour images. Like the Trios® scanner, ZFX Intrascan® has a big wand; however, it is an open system since it allows the export of.STL files without any limitation. Like the CS 3500®, ZFX Intrascan® scans can be opened by all CAD software available on the market, and therefore used to design crowns, bridges, inlays, onlays, and veneers. Finally, ZFX Intrascan® can be easily plugged into a laptop via a USB port. The version of the software used here was the 0.9 RC33 2.8.

#### Planmeca Planscan

The Planmeca PlanScan® (powered by E4D Technologies, LLC, Richardson, TX, USA) works under the principle of optical coherence tomography and confocal microscopy. This powder-free scanner employs a blue light with real-time laser video-streaming technology to produce in-colour images. It has tips of various dimensions with built-in heated mirrors. Planmeca Planscan® is an open system, since it allows conversion of the acquired proprietary files into.STL files, readable by all CAD systems. Like CS 3500® and ZFX Intrascan®, the PlanScan® can be easily connected to a laptop via a USB port. The Planmeca PlanCAD software includes the scanning software (release 5–2015) and the CAD software, together with a laptop PC; finally, like Carestream, Planmeca has a proprietary milling machine available for the fabrication of full in-office digital restorations such as inlays, onlays, crowns, bridges, veneers.

### General trueness and precision

All 3D surface models (the reference R1 models acquired with the powerful optical scanner, as well as all.STL files obtained with the 4 different intraoral scanners) were imported into the Studio 2012® reverse-engineering software (Geomagic, Morrisville, NC, USA). Here, small artefacts identified as independent polygons were automatically removed using the “mesh doctor” function, and models were cut/trimmed to remove all unnecessary information, using the “cut with planes” function. A preformed template was adopted to cut all models in the most uniform manner: with this, uniform models were obtained and saved in specific folders. Before commencing the superimposition of 3D models, the validity of the method was tested, and the following operations were made for both the partially and totally edentulous models. In brief, the reference R1 model was imported into the software, duplicated and moved to another location; these two identical models were then superimposed and registered, and the software calculated the difference between the two surfaces. This test was repeated five times, in order to certify the reliability of the procedure. After these validation tests, it was possible to proceed with the superimposition for the evaluation of the general trueness, which was performed as previously reported [31]. In brief, all 3D surface models obtained from each intraoral scanners were superimposed to the corresponding R1 reference model, using the “three-point registration” function. The three points were easily identified on the surface of the implant scanbodies. After this first rough alignment, the “best fit” alignment function was used for the final registration. After defining the reference dataset (R1), as well as the parameters for the registration, the corresponding polygons of the selected models were automatically superimposed. An “iterative-closest-point” algorithm, also defined as robust-iterative-closest-point (RICP), was used for superimposition. The distances between the R1 and the superimposed models were minimized using a point-to-plane method; congruence between specific corresponding structures was calculated. Therefore, the distances between corresponding areas of R1 and all superimposed models were colour-coded on the superimposed models for visualization of the result, using the “3D deviation” function. A colour map was generated, where the distances between specific points of interest were quantified, overall, and in all three planes of space. All deviations were therefore visualized and calculated. The colour maps indicated inward (blue) or outward (red) displacement between overlaid structures. An absence of change was indicated by a green colour. For each superimposition, mean and standard deviations (SD) were obtained.

### Local trueness

The local trueness of the four scanners evaluated in this study was calculated by measuring the distances and angles present between the different scanbodies; the centers of the scanbodies were taken as reference points for calculating, as previously reported by van der Meer and colleagues [27]. In brief, each scan was imported in the aforementioned software (Studio 2012®, Geomagic, Morrisville, NC, USA). The scanned cylinders were isolated, registered with the original computer-assisted-design (CAD) models of the scanbodies, and imported into the reverse-engineering software: this allowed the identification of the exact centre of each cylinder. The validity and reliability of the superposition method was confirmed, as previously reported. In brief, a CAD cylinder was imported into the reverse-engineering software, duplicated, and moved to another location: these two cylinders were then registered, and the software calculated the difference between the two identical surfaces. This test was repeated ten times, in order to certify the validity of the procedure. After that, linear and angular measurement tools were used to calculate the distances and angles between the centres of the scanbodies. All these data were inserted into a table for comparison with the corresponding measures taken on the reference (R1) model.

### Statistical analysis

We performed a statistical analysis for mean absolute deviations. Trueness was defined from the comparison between each scan (1 to 5 for every scanner) and the reference model (R1). The analysis was first stratified by the model (partially and totally edentulous maxilla). For each scanner, we estimated the mean trueness and its standard deviation from analysis of variance, and tested all possible pairwise comparisons between scanners, using the Tukey method for multiple comparisons. We report in tables’ footnotes the minimum significant mean differences after the Tukey’s correction, as a guidance for data interpretation. Bartlett’s test was used for the assumption of homoscedasticity of variances across groups. These analyses were replicated for precision, defined as the comparison between scans made with the same instrument. Then, we compared trueness and precision of any given scanner by model type using an analysis of variance model including scanner, model and their interaction. For each scanner, we also had 5 measures of local trueness parameters (partially edentulous model: 3 distances and 1 angle; totally edentulous model: 7 distances and 4 angles). For each parameter, we plotted the minimum and maximum measurements, as well as the mean value, which constitutes the trueness. All statistical analyses were conducted using SAS software release 9.4 (SAS Institute, Cary, NC), while plots were drawn using R (3.2.3 release).

## Results

The manufacturer’s data of the reference scanner were essentially validated, since the mean difference between our three reference scans was 6.3 ± 6.6 μm in the partially edentulous model, and 14.4 ± 7.9 μm in the fully edentulous model.

The registration/superimposition method for the evaluation of general trueness and precision was found reliable, as the final result of the validation tests was a negligible mean registration error in both 3D models (2.8 ± 3.0 nm in the partially edentulous maxilla; 3.2 ± 1.7 nm in the fully edentulous maxilla): this certified the validity of the procedure. Similar results were obtained in the validation of the registration method for the evaluation of local trueness: a negligible error of 2.8 ± 2.5 nm certified the reliability of the overlapping procedure.

The general trueness and precision of the four intraoral scanners for the partially and totally edentulous models are summarized in Tables 1 and 2, respectively.

**Table 1:** Mean general trueness and standard deviation (SD), in μm, for partially and totally edentulous maxilla, and p values testing the scanner by model interaction. N = 5 scans for each scanner and model type.

Scanner	Partially edentulous maxilla	Totally edentulous maxilla	p-value^1^
	Mean Trueness ± SD (μm)	Mean Trueness ± SD (μm)	
Trios®	71.2 ± 19.5 †	71.6 ± 26.7 †	0.9
CS 3500®	47.8 ± 7.3 ‡ §	63.2 ± 7.5 ‡ §	0.4
Zfx Intrascan®	117.0 ± 28.6 ‡ ¶	103.0 ± 26.9 ‡ ¶	0.5
Planscan®	233.4 ± 62.6 † § ¶	253.4 ± 13.6 † § ¶	0.3

The same symbol after SD indicates differences in trueness between scanner pairs (Tukey-adjustment for multiple comparison). Minimum significant difference across scanners: 65.1 μm and 37.1 μm for partially and totally edentulous maxilla models, respectively.

1 p-value testing of the interaction between scanner and model type (partially vs totally edentulous maxilla): a p-value > 0.05 indicates no difference in scanner trueness according to model type.

**Table 2:** Mean general precision and standard deviation (SD), in μm, for partially and totally edentulous maxilla, and p values testing the scanner by model interaction. N = 5 scans for each scanner and model type.

Scanner	Partially edentulous maxilla	Totally edentulous maxilla	p-value^1^
	Mean Precision ± SD (μm)	Mean Precision ± SD (μm)	
Trios®	51.0 ± 18.5 † ‡	67.0 ± 32.2 † ‡	0.4
CS 3500®	40.8 ± 6.4 § ¶	55.2 ± 10.4 § ¶	0.4
Zfx Intrascan®	126.2 ± 21.2 † § °	112.4 ± 22.6 † § °	0.5
Planscan®	219.8 ± 59.1 ‡ ¶ °	204.2 ± 22.7 ‡ ¶ °	0.4

The same symbol after SD indicates differences in trueness between scanner pairs (Tukey-adjustment for multiple comparison). Minimum significant difference across scanners: 59.5 μm and 42.2 μm for partially and totally edentulous maxilla models, respectively.

1 p-value testing of the interaction between scanner and model type (partially vs totally edentulous maxilla): a p-value > 0.05 indicates no difference in scanner precision according to model type.

**Fig 1:**
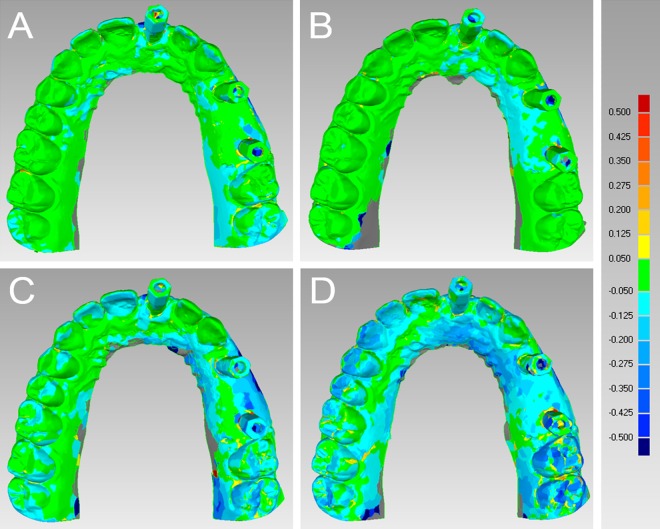
General trueness in the partially edentulous maxilla. The best single result obtained with each device were: (A) Trios® 48 ± 81 μm; (B) CS 3500® 37 ± 81 μm; (C) Zfx Intrascan® 76 ± 97 μm; (D) Planscan® 124 ± 106 μm.

**Fig 2:**
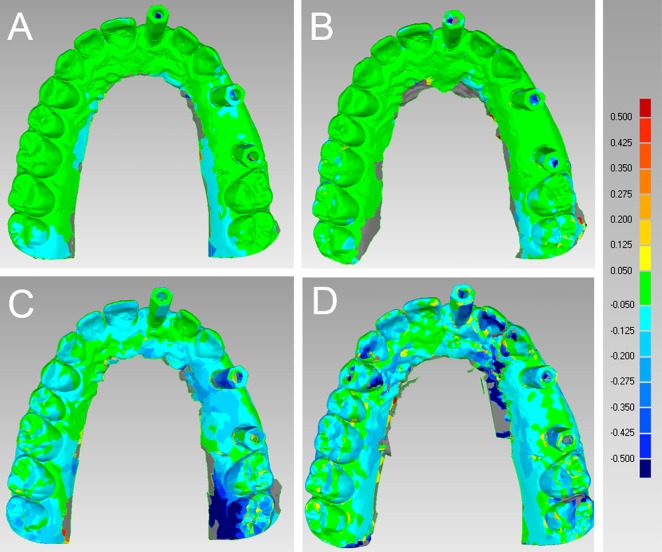
General precision in the partially edentulous maxilla. The best single result obtained with each device were: (A) Trios® 28 ± 44 μm; (B) CS 3500® 34 ± 66 μm; (C) Zfx Intrascan® 94 ± 106 μm; (D) Planscan® 115 ± 123 μm.

In the partially edentulous model, CS 3500® had the best performance in terms of general trueness (47.8 μm) and precision (40.8 μm), followed by Trios® (general trueness and precision of 71.2 μm and 51.0 μm, respectively), Zfx Intrascan® (general trueness and precision of 117.0 μm and 126.2 μm, respectively) and Planscan® (general trueness and precision of 233.4 μm and 219.8 μm, respectively). With regard to general trueness, Trios® was significantly better than Planscan®, CS 3500® was significantly better than Zfx Intrascan® and Planscan®, and Zfx Intrascan® was significantly better than Planscan®. With regard to general precision, Trios® was significantly better than Zfx Intrascan® and Planscan®, CS 3500® was significantly better than Zfx Intrascan® and Planscan®, and Zfx Intrascan® was significantly better than Planscan®. The analysis of deviations (colour maps) for trueness and precision in the partially edentulous patient is reported in Figs 1 and 2, S1 File.

**Fig 3:**
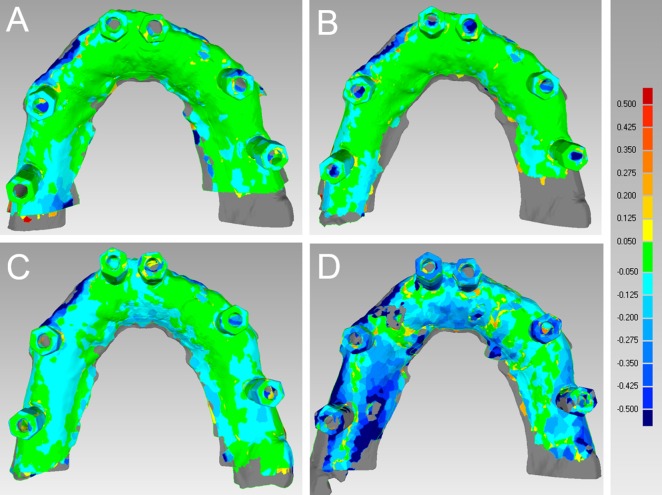
General trueness in the totally edentulous maxilla. The best single result obtained with each device were: (A) Trios® 57 ± 95 μm; (B) CS 3500® 51 ± 88 μm; (C) Zfx Intrascan® 83 ± 105 μm; (D) Planscan® 234 ± 188 μm.

**Fig 4:**
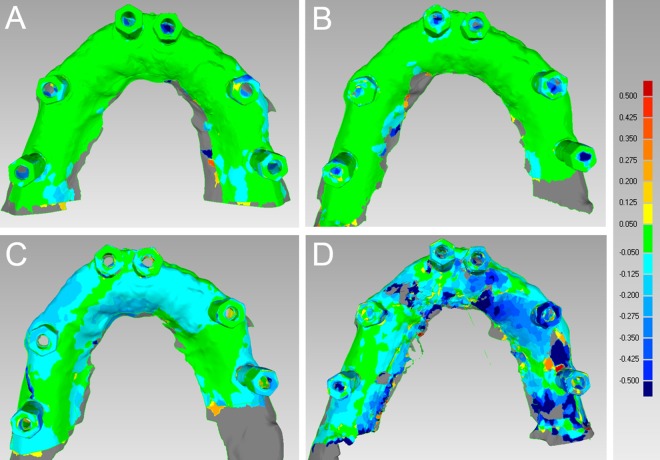
General precision in the totally edentulous maxilla. The best single result obtained with each device were: (A) Trios® 35 ± 76 μm; (B) CS 3500® 38 ± 67 μm; (C) Zfx Intrascan® 87 ± 101 μm; (D) Planscan® 179 ± 172 μm.

In the totally edentulous model, CS 3500® had the best performance in terms of general trueness (63.2 μm) and precision (55.2 μm), followed by Trios® (general trueness and precision of 71.6 μm and 67.0 μm, respectively), Zfx Intrascan® (general trueness and precision of 103.0 μm and 112.4 μm, respectively) and Planscan® (general trueness and precision of 253.4 μm and 204.2 μm, respectively). With regard to general trueness, Trios® was significantly better than Planscan®, CS 3500® was significantly better than Zfx Intrascan® and Planscan®, and Zfx Intrascan® was significantly better than Planscan®. With regard to general precision, Trios® was significantly better than Zfx Intrascan® and Planscan®, CS 3500® was significantly better than Zfx Intrascan® and Planscan®, and Zfx Intrascan® was significantly better than Planscan®. The analysis of deviations (colour maps) for trueness and precision in the totally edentulous patient is summarized in Figs 3 and 4, S1 File.

**Fig 5:**
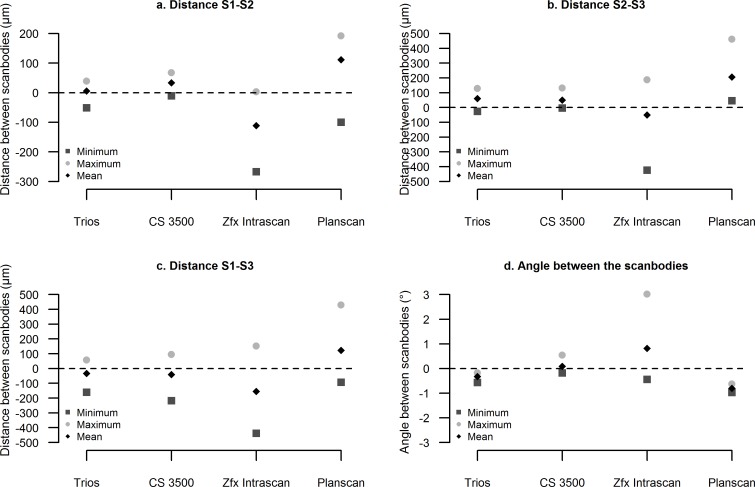
Local trueness in the partially edentulous maxilla. Distances and angle between the scanbodies.

**Fig 6:**
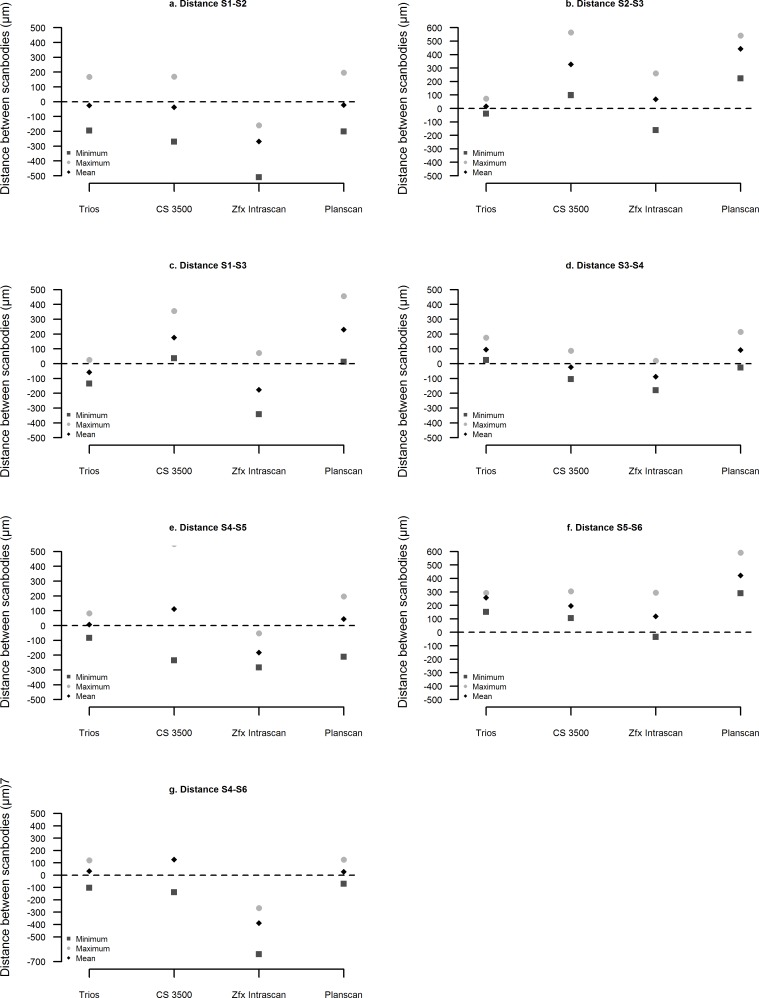
Local trueness in the totally edentulous maxilla. Distances between the scanbodies.

**Fig 7:**
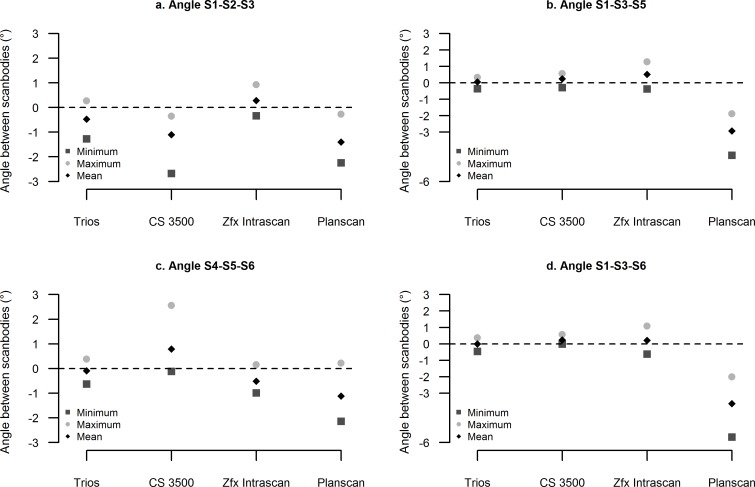
Local trueness in the totally edentulous maxilla. Angles between the scanbodies.

Finally, the local trueness of the four intraoral scanners for the partially and totally edentulous models are illustrated in Figs 5, 6 and 7, respectively.

## Discussion

The digital revolution is changing the world, and dentistry is no exception. The introduction of a whole range of digital devices (intraoral, extraoral and face scanners, cone beam computed tomography with low dose radiation—CBCT) and processing software (computer-assisted-design/computer-assisted-manufacturing—CAD/CAM prosthetic software, software for planning implant surgery, etc.), together with new aesthetic materials and powerful manufacturing and prototyping tools (milling machines and 3D printers), is radically transforming the dental profession [1,3,5,32]. Intraoral scanners have been introduced to allow dentists to take optical impressions of the dental arches, using only a beam of light [2,3,10]. Optical impressions are supplanting conventional impressions, which involve tray and impression materials: this last procedure, unwelcome to patients, is likely to disappear over the next few years [5–9, 32–35].

Although several *in vitro* and *in vivo* studies have demonstrated that intraoral scanners can be a valuable tool for taking impressions of single and multiple abutments in fully dentate patients [15–22], it is still not clear whether these devices can be successfully used in implantology, particularly in the case of long-span prosthetic restorations [21,22,27–29]. Moreover, little is known about the trueness and precision of the different devices available on the market [11,13,23–28].

Until now, in fact, only a few studies have compared the trueness and precision of different intraoral scanners [23–27], and most of these works are on fully dentate models [23–26]. In an *in vitro* study by Schaefer and colleagues [23] on the impact of digital impressions on the adaptation of ceramic partial crowns, an acrylic model of a mandibular first molar was prepared to receive a partial-coverage lithium disilicate crown. The preparation was scanned with an optical industrial reference scanner, then using 4 different intraoral scanners (CEREC AC Bluecam®, iTero®, Lava COS® and cara TRIOS®) [23]. Before restorations were designed and machined from lithium disilicate blanks, data from intraoral scanners were loaded into reverse-engineering software, and superimposed on the reference model, in order to evaluate the trueness of the different impression systems. Mean marginal internal discrepancies were found to be 90 ± 92 μm (iTero®), 109 ± 93 μm (Lava COS®), 128 ± 106 μm (Cara TRIOS®), and 146 ± 84 μm (CEREC AC Bluecam®), respectively [23]. Differences among impression systems were statistically significant at p < 0.001. The authors concluded that although all fabricated restorations showed acceptable marginal gap sizes, the investigated digital impression systems demonstrated significant differences and fit discrepancies [23]. Similar results were found by Nedelcu and colleagues [24], who evaluated the trueness and precision of 4 intraoral scanners, assessing the influence of different test materials and coating thicknesses. The authors concluded that intraoral scanners should be used with caution, in selected clinical contexts such as shorter-spanned prosthetic solutions, until the accuracy and precision of these devices improves, and therefore the validation of the complete digital workflow will be extended to more challenging situations (long-span prosthesis, full-arch restorations) [24]. The conclusions of this work appear shareable: to date, in fact, only a few studies have compared the trueness and precision of different scanners in difficult contexts [25–27], such as the scan of multiple implant abutments in partially and totally edentulous patients [27,28]. In a recent *in vitro* study, Patzelt and colleagues compared the trueness and precision of 4 intraoral scanners in full-arch scans of fully dentate patients, with 14 prepared abutments [25]. A representative model was digitized with a reference scanner (IScan D101®, Imetric 3D GmbH, Courgenay, Switzerland), and then with four different intraoral scanners (CEREC AC Bluecam®, iTero®, Lava COS®, and Zfx Intrascan®). Datasets obtained from different intraoral scanners were loaded into 3D-analysis software, then superimposed on the reference scan for the evaluation of trueness, and superimposed on each other within groups for the evaluation of repeatability (precision). At the end of the study, mean trueness values were between 38 and 332.9 μm [25]. With regard to trueness, CEREC AC Bluecam® was the worst scanner (332.9 ± 64.8 μm), while Lava COS® was the best (38.0 ± 14.3 μm). The other scanners showed similar results in terms of trueness, with iTero® (49.0 ± 13.6 μm) followed by Zfx Intrascan® (73.7 ± 26.6 μm). A statistically significant difference (p<0.05) was found between the trueness of CEREC AC Bluecam® and other scanners, as well as between Zfx Intrascan® and Lava COS® [25]. Mean repeatability (precision) values ranged from 37.9 ± 99.1 μm. Lava COS® was the most precise (37.9 ± 19.1 μm), followed by iTero® (40.4 ± 11.3 μm); CEREC AC Bluecam® (99.1 ± 37.4 μm) and Zfx Intrascan® (90.2 ± 26.7 μm) were the least precise. With regard to precision, statistically significant differences were found between CEREC AC Bluecam® and Lava COS®, CEREC AC Bluecam® and iTero®, Zfx Intrascan® and Lava COS®, and Zfx Intrascan® and iTero® (p <0.05) [25].

All these comparative studies on dentate models support the concept that by using different intraoral scanners, significantly different results can be achieved [23–25]. Generally it is believed that the dentate model is the easiest to deal with for intraoral scanners: in fact, the presence of occlusal surfaces with their peculiar geometry may help these devices to achieve a better result [2,3,26]. However, this has not been proven yet: it is therefore important to investigate the feasibility and accuracy in digitizing partially and fully edentulous jaws, particularly in the patient with dental implants.

In a recent study by Patzelt and colleagues [26], two representative edentulous jaws models (maxilla and mandible) were digitized using an industrial reference scanner (laser scanner), and four different intraoral scanners (CEREC AC Bluecam®, iTero®, Lava COS®, and Zfx Intrascan®). Again, all datasets were loaded into 3D-evaluation software, where intraoral scans were superimposed on the reference model to evaluate trueness, and superimposed on each other within groups to evaluate precision [26]. At the end of the study, mean trueness values ranged from 44.1 to 591.8 μm. With regard to trueness, the Lava COS® was the best scanner (52.9 ± 23.8 maxilla, 44.1 ± 5.0 mandible), followed by iTero® (139.5 ± 72.4 maxilla, 154.7 ± 67.9 mandible) and Zfx Intrascan® (283.8 ± 187.3 maxilla, 253.8 ± 127.1 mandible); CEREC AC Bluecam® was the worst (591.8 ± 377.9 maxilla, 558.4 ± 616.2 mandible) [26]. With regard to repeatability, mean precision values ranged from 21.6 to 698.0 μm. The Lava COS® was the most precise scanner (30.8 ± 17.0 maxilla, 21.6 ± 10.1 mandible) followed by iTero® (166.8 ± 89.0 maxilla, 217.3 ± 109.2 mandible) and Zfx Intrascan® (425.3 ± 278.6 maxilla, 319.4 ± 127.5 mandible); CEREC AC Bluecam® was the least precise (332.4 ± 183.3 maxilla, 698.0 ± 585.5 mandible) [26]. With regard to overall 3D deviations, although no differences were found between maxillary and mandibular jaw scans, the accuracy of the intraoral scanner differed significantly (p<0.05), and only one scanner was sufficiently accurate for this application: therefore, the authors concluded that at present, direct digitization of edentulous jaws should not be recommended *in vivo* [26].

In an interesting *in vitro* study, van der Meer and colleagues [27] compared the trueness of three different intraoral scanners (CEREC AC Bluecam®, iTero®, and Lava COS®) using a model of a partially edentulous patient with 3 implants. The implants were connected with 3 PEEK cylinders, and 10 different scans of the model were taken for each intraoral scanner; all data were then imported into reverse-engineering software, where the distance between the centres of the cylinders and the angulation between the cylinders were assessed [27]. These values were then compared to the measurements obtained with an industrial 3D scan of the master model. With regard to distance errors, when considering mean distance errors in full arch impressions (in absolute values and in consistency for measured distances) Lava COS® showed the smallest and most consistent, while CEREC AC Bluecam® showed the largest and least consistent [27]. All angulation errors were small [27]. The authors concluded that an increase in distance and angular errors should be expected with intraoral scanners over the length of the arch, due to an accumulation of registration errors of the patched 3D surfaces [27]: this in accordance with other more recent studies [12,14]. Papaspyridakos and colleagues [28] compared the trueness of digital and conventional impression techniques in implant patients. In brief, a stone cast of a fully edentulous mandible with five implants was fabricated to serve as master cast [28]. Scanbodies were screwed on, then the cast was digitized with a powerful, modern intraoral scanner (Trios®, 3-Shape, Copenhagen, Denmark). After this, conventional impressions of the master cast were taken using polyether, with a splinted and a non-splinted technique, respectively [28]. A powerful extraoral scanner was then used to scan the master cast (in order to obtain a reference STL dataset) and the splinted and non-splinted conventional impressions. Solid-to-layer (STL) datasets from digital and conventional impressions were then superimposed on the STL dataset from the master cast to calculate errors and deviations [28]. At the end of the study, the quality of digital implant impressions was similar to that of conventional polyether impressions [28]; among conventional impressions, the splinted technique was preferable to the non-splinted, in terms of accuracy [28]. These results have been confirmed by a recent clinical study [29], in which the same intraoral scanner has been used.

Although all these comparative studies were very well designed and provided important information, they were mostly based on first-generation scanners [26,27]; moreover, only a few of them focused on dental implants [27,28].

Hence, in our present study, we have evaluated the general trueness and precision of four modern intraoral scanners, in two different settings: a partially edentulous model with three implants, and a fully edentulous model with six implants. In addition, since this general mathematical analysis of the quality of the obtained models may not indicate specific error fluctuations over longer spans (such as the distances or angles between different implant scanbodies), we also measured the distances and angles over a longer span between simulated implants, in order to evaluate the local trueness of the investigated devices.

At the end of the study, although no differences in trueness and precision were found between partially and totally edentulous models, statistically significant differences were found between the different scanners. In fact, with regard to general trueness, Trios® was statistically superior than Planscan®, CS 3500® was statistically superior than Zfx Intrascan® and Planscan®, and Zfx Intrascan® was statistically superior than Planscan®. Similar results were found for general precision, where Trios® was statistically superior than Zfx Intrascan® and Planscan®, CS 3500® was statistically superior than Zfx Intrascan® and Planscan®, and Zfx Intrascan® was statistically superior than Planscan®. Local trueness values confirmed these results. Our present work seems to support the concept that, using intraoral scanners of the latest generation, it is possible to take sufficiently accurate impressions, even in challenging situations, to theoretically allow the fabrication of long-span implant-supported restorations. In fact, with at least two of the intraoral scanners used in this study (CS 3500®, Carestream Health, Rochester, NY, US and Trios®, 3-Shape, Copenhagen, Denmark) the general and local trueness values were theoretically compatible with the fabrication of complex restorations, such as long-span implant-supported fixed partial prostheses or full-arches. The other two scanners (Zfx Intrascan®, MHT S.p.A., Verona, Italy, and Planmeca Planscan®, E4D Technologies, LLC, Richardson, TX, USA) however, were found not suitable for taking implant impressions in the long-span partially or totally edentulous patient. As a consequence, in accordance with previous studies [26,27], the results of our study suggest that care should be taken before using intraoral scanners for capturing digital impressions in implant patients with long-span prosthesis (and particularly in the case of fixed full arch implant-supported restorations). Trueness and precision of intraoral scanners need therefore to be improved, before direct digitization of edentulous jaws can be recommended *in vivo*.

Our present study has limits. First, it is an *in vitro* study, therefore the present results should be validated *in vivo*. In fact, *in vivo* there are many more difficulties and/or variables (presence of saliva, blood, limited mouth opening typical in some patients) which can affect the final outcome and quality of digital impressions [29]. Second, although very powerful, the scanner used as a reference (IScan D101®, Imetric 3D GmbH) was a desktop scanner: this may represent another limitation of the present study. The use of a more powerful optical industrial scanner, or even better a contact scanner, that can physically probe the surface of the scanned models (such as articulated arm or coordinate measuring machine, CMM) could be preferable. In fact, contact scanners are still the best references in terms of trueness and precision, although they are slow and expensive. Third, with regard to local trueness evaluation, since the measurements were not broken down into xyz components, it is not clear in what direction the deviations point to. Fourth, some limitations are related to the sample size. The number of scans for each instrument (n = 5) is a convenient sample size taking into account similar studies [25,26] and the available resources. The standard deviation for trueness and precision for the Planscan instrument was larger than for the remaining scanners, leading to a rejection of the homogeneity of variances in the ANOVA models. However, this was due to one scan with increased trueness, and one with increased precision, with respect to the Planscan average parameters. Therefore, by deleting these observations we would have respected the homogeneity assumption, but the mean trueness and precision for the last scanner would have worsened, thus confirming the statistical significance of the differences reported in Tables 1 and 2. Finally, it is important to remember that companies are continuously investing, in order to improve the trueness and precision of their intraoral scanners, particularly with regard to the acquisition/reconstruction software. Consequently, it is possible that the present study’s results could be challenged by the latest release of acquisition/reconstruction software. This is a positive element, and there is no doubt that in a short time, it will be possible to take sufficiently accurate intraoral digital impressions *in vivo*, using different scanners, and even in difficult contexts [31,32].

## Conclusions

In the present *in vitro* study we have compared the trueness and precision of four intraoral scanners in oral implantology, in two challenging models (a partially edentulous patient with three implants, and a totally edentulous patient with 6 implants). Although no differences in trueness and precision were found between the partially and totally edentulous models, the investigated digital impression systems differed significantly. Further *in vivo* studies are required to validate these results. At present, care should be taken before using intraoral scanners for capturing digital impressions in implant patients with long-span prosthesis.

## Supporting Information

S1 File (PDF)
