# Supplementary material for: Trueness and Precision of Four Intraoral Scanners in Oral Implantology: A Comparative in Vitro Study
Source: PLoS One. 2016 Sep 29;11(9):e0163107. doi: 10.1371/journal.pone.0163107 (PMC5042463; doi:10.1371/journal.pone.0163107)
Supplement: S1 File — (PDF) [file pone.0163107.s001.pdf]

Trueness and Precision of Four Intraoral Scanners in Oral Implantology: a Comparative in Vitro Study

Francesco G. Mangano [1, 2, ¶, *], Giovanni Veronesi [3, &], Uli Hauschild [4, &], Eitan Mijiritsky [5], and Carlo Mangano [2, ¶]

SUPPORTING INFORMATIONS

VALIDATION OF THE MANUFACTURER'S DATA FOR THE REFERENCE SCANNER (IMETRIC) IN BOTH THE PARTIALLY AND TOTALLY EDENTULOUS MODELS.

Methods. The two stone models (partially and a totally edentulos maxilla, respectively) were scanned with the reference scanner; three scans were taken for each model. For each model, all generated datasets were imported into a powerful reverse-engineering software (Studio 2012®, Geomagic, Morrisville, NC, USA) and superimposed each other, in order to validate the manufacturer's data. One dataset for each model was then selected as the reference dataset (R1) for the trueness measurements of all intraoral scanners.

Results. The manufacturer's data of the reference scanner were validated, since a minimal difference was found between the difference scans (6.3 ± 6.6 micrometers in the partially edentulous model; 14.4 ± 7.9 micrometers in the fully edentulous model).

VALIDATION OF THE MANUFACTURER DATA

PARTIALLY EDENTULOUS MODEL

Values setting: 0.25, 0.02, -0.02, -0.25

3 different scans with Imetric are taken and superimposed each other,

IN ORDER TO VALIDATE THE MANUFACTURER’S DATA.

The final result was a precision of 6.3 ± 6.6 micrometers.

Validation of manufacturer's data the partially edentulous model — Imetric 1 vs Imetric 2

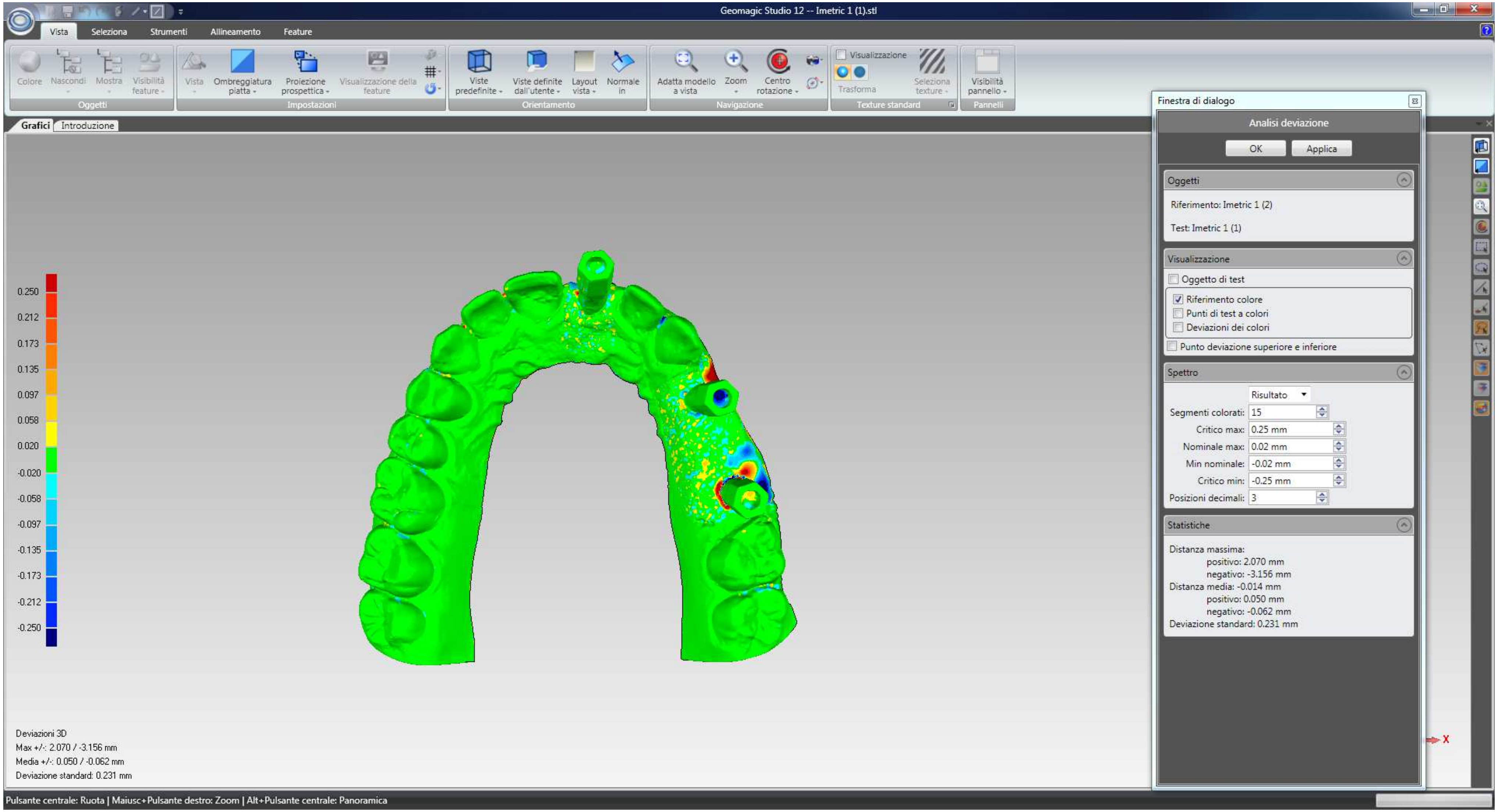

Imetric 1 vs Imetric 2

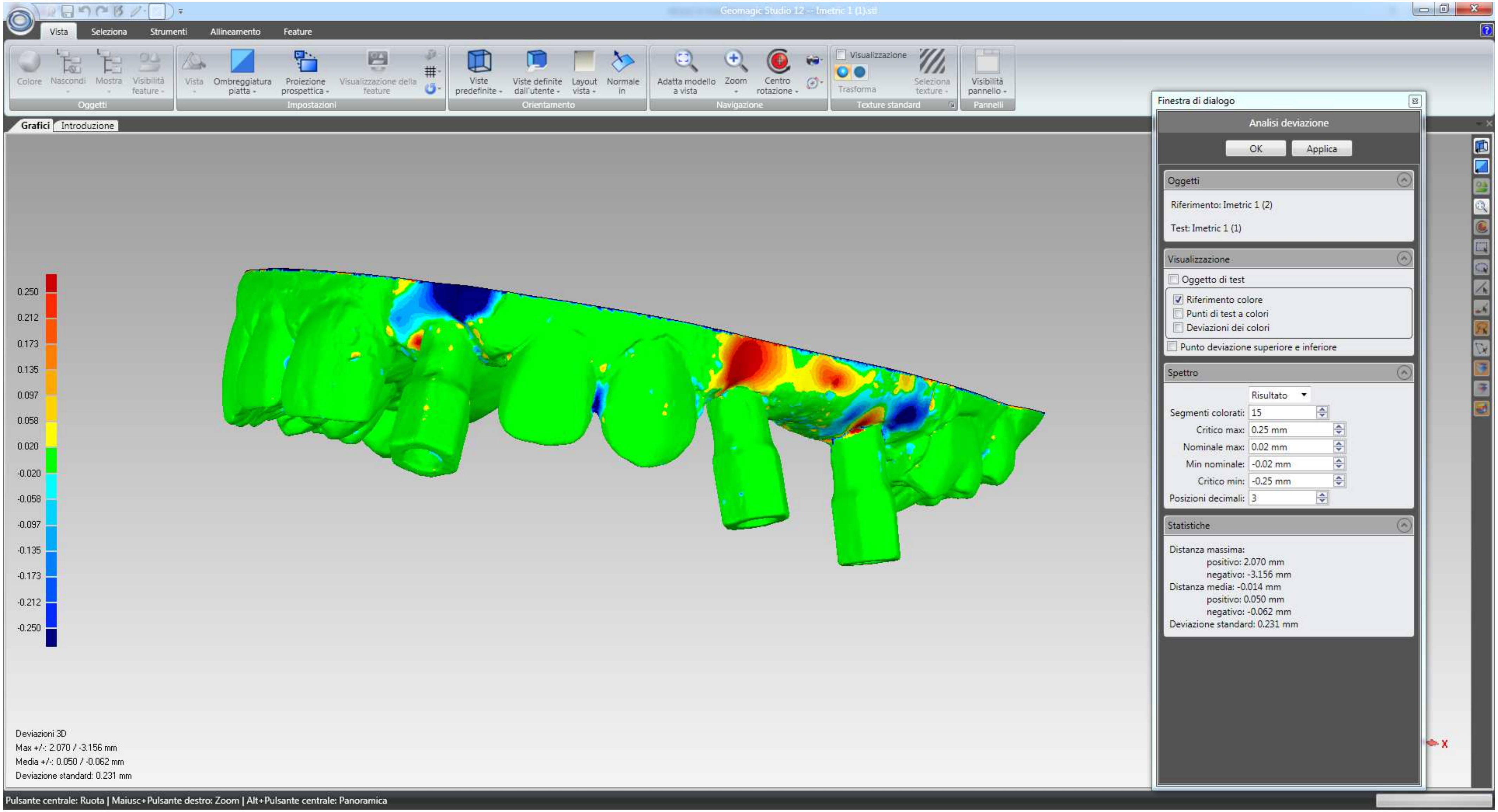

Imetric 1 vs Imetric 2

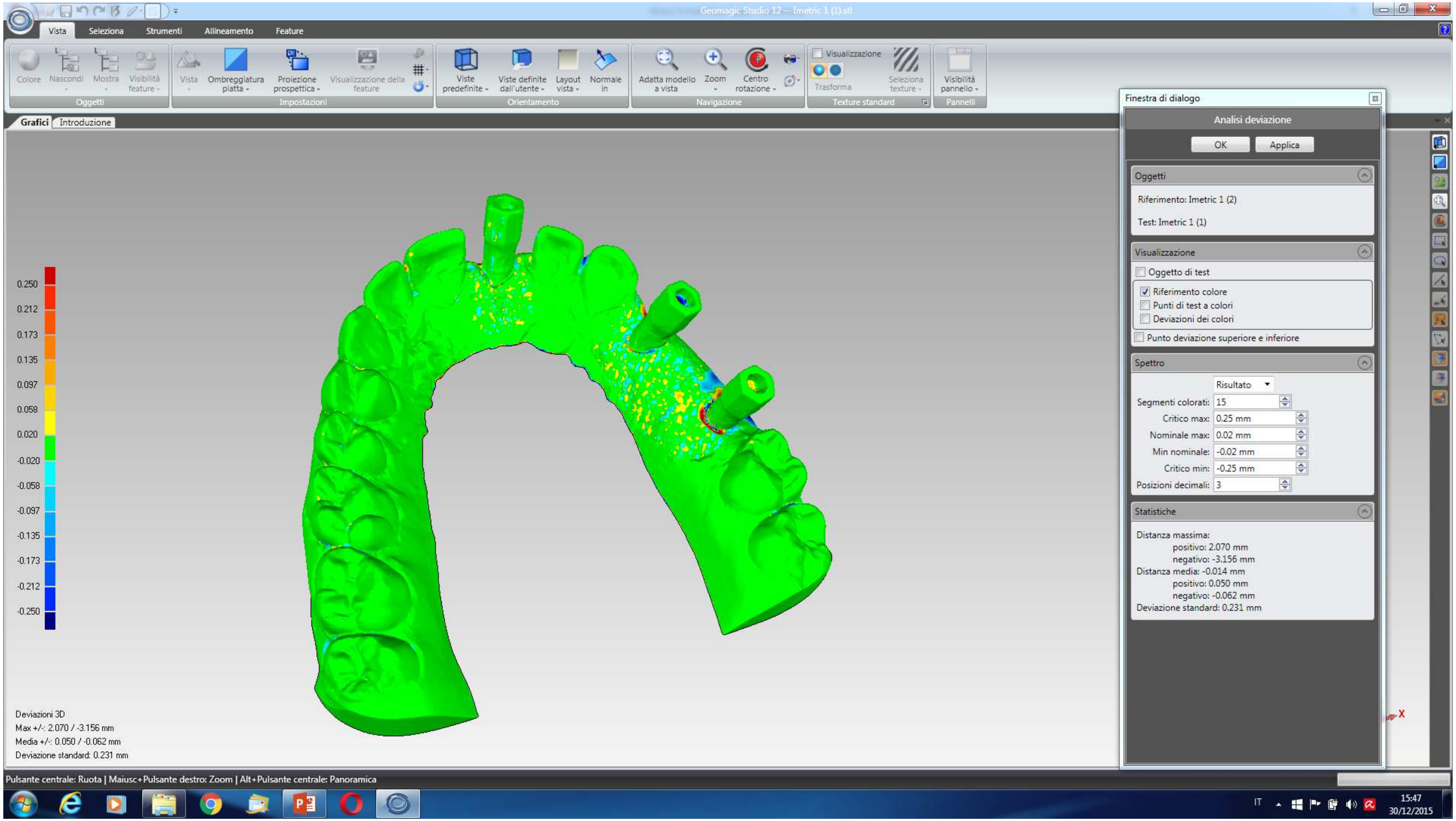

Imetric 2 vs Imetric 3

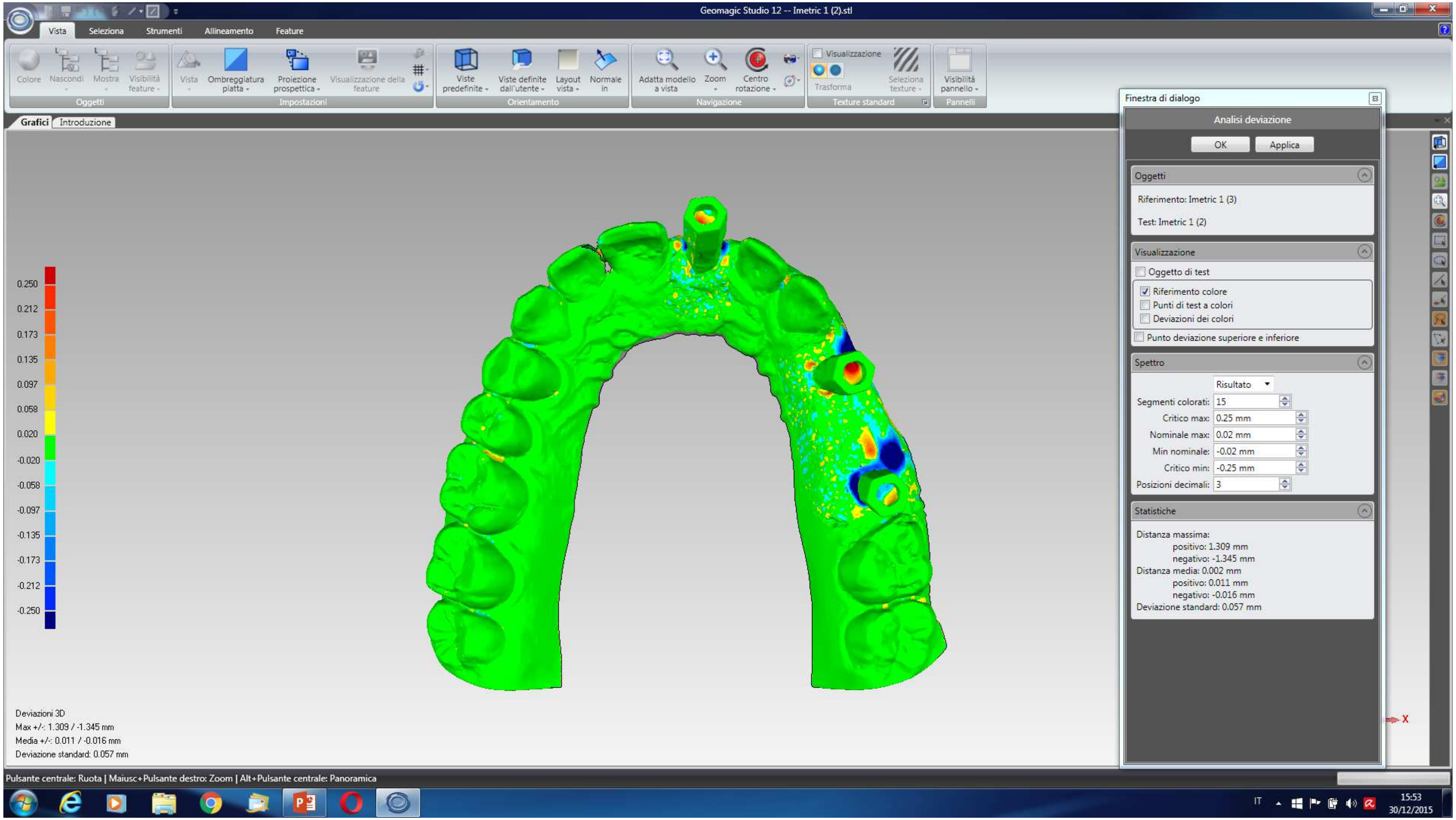

Imetric 2 vs Imetric 3

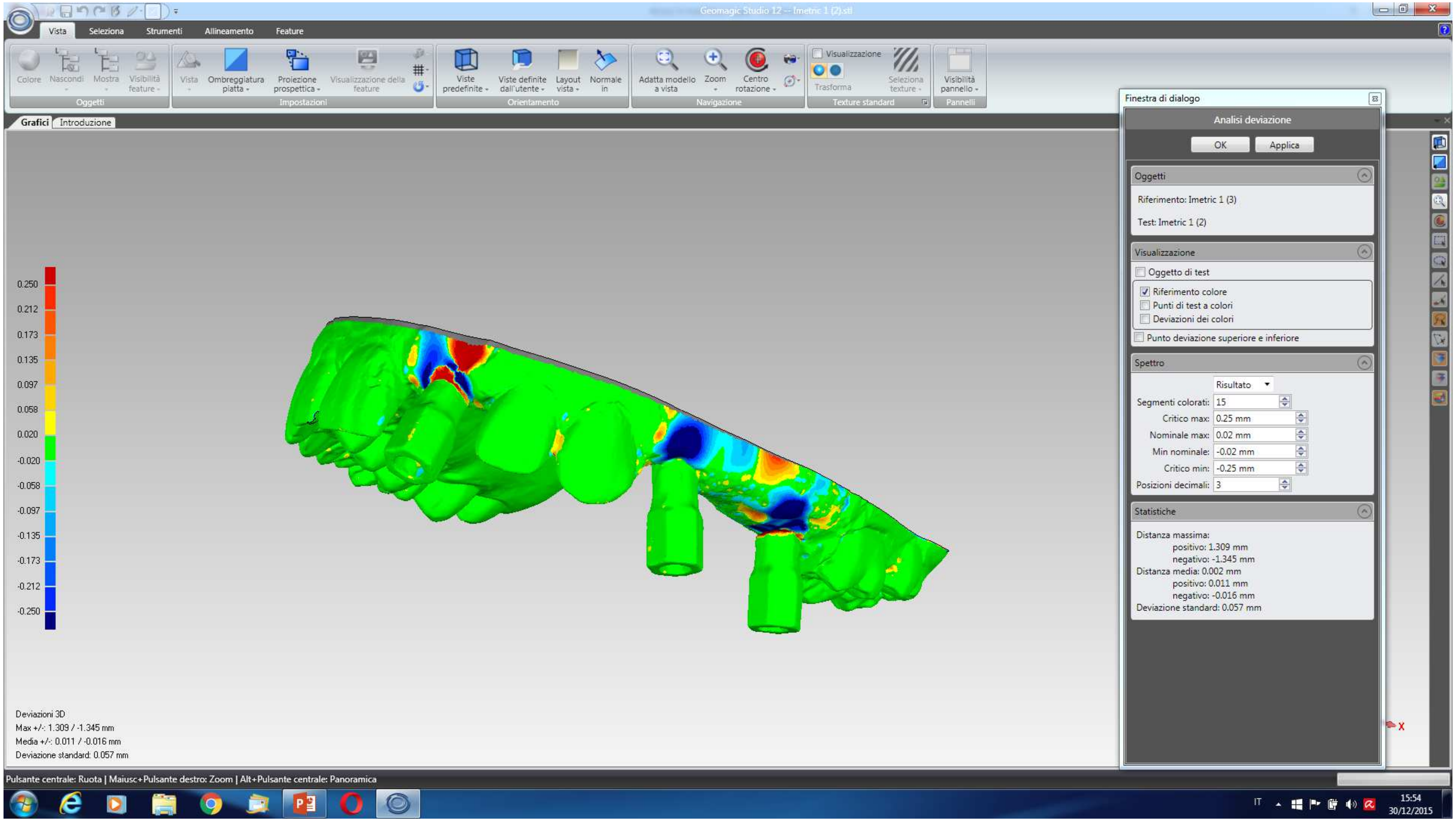

Imetric 2 vs Imetric 3

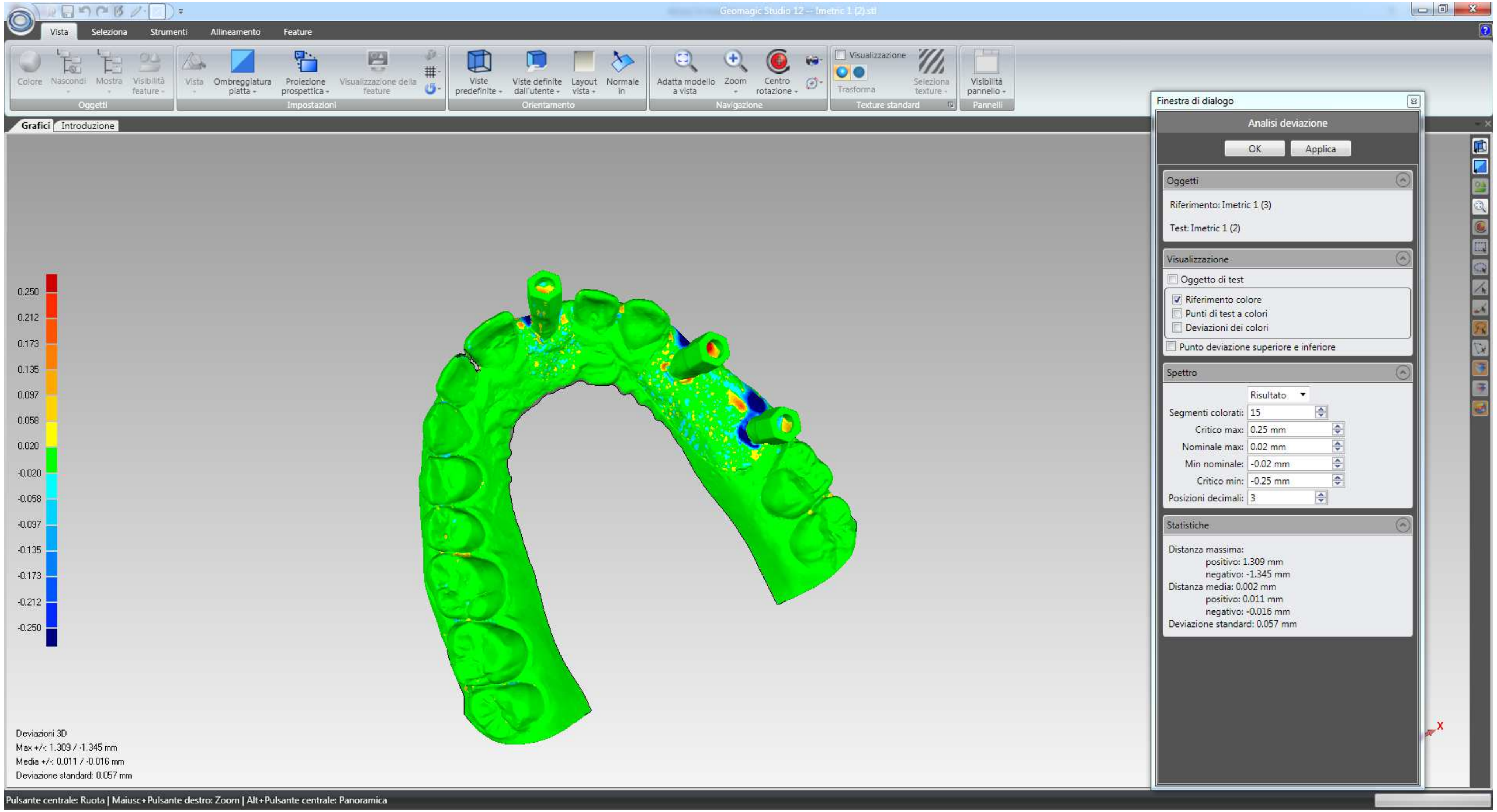

Imetric 1 vs Imetric 3

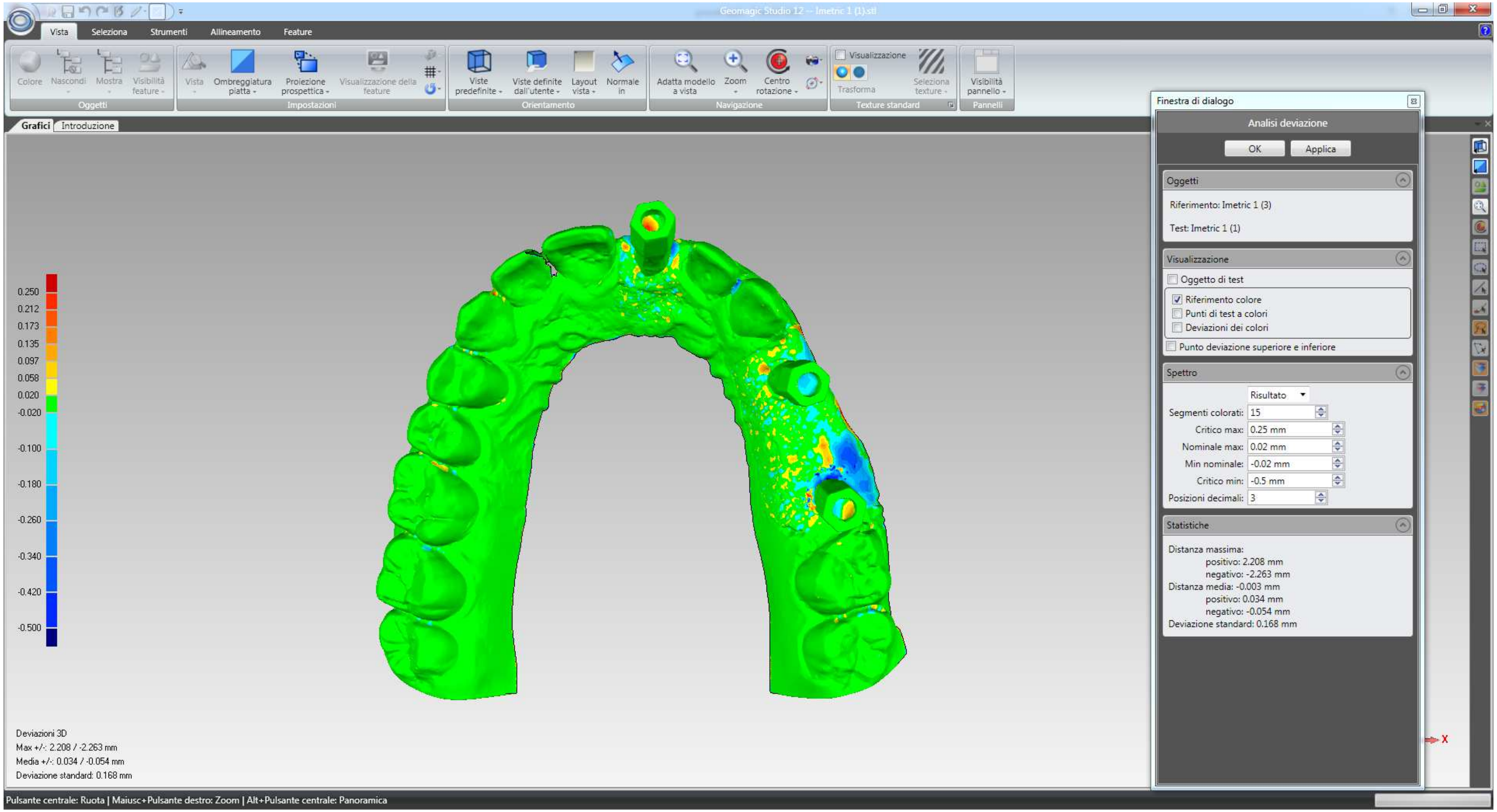

Imetric 1 vs Imetric 3

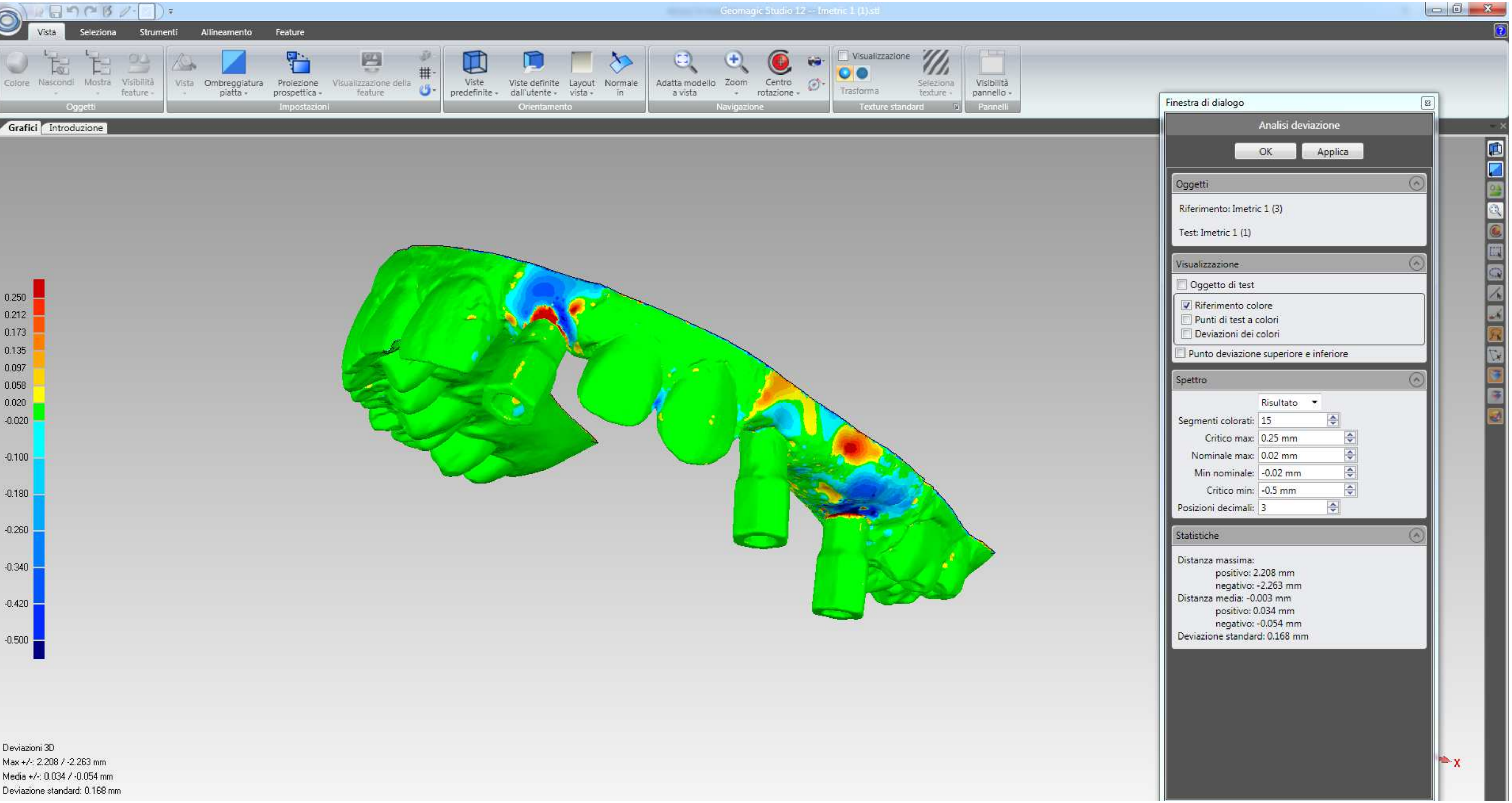

Imetric 1 vs Imetric 3

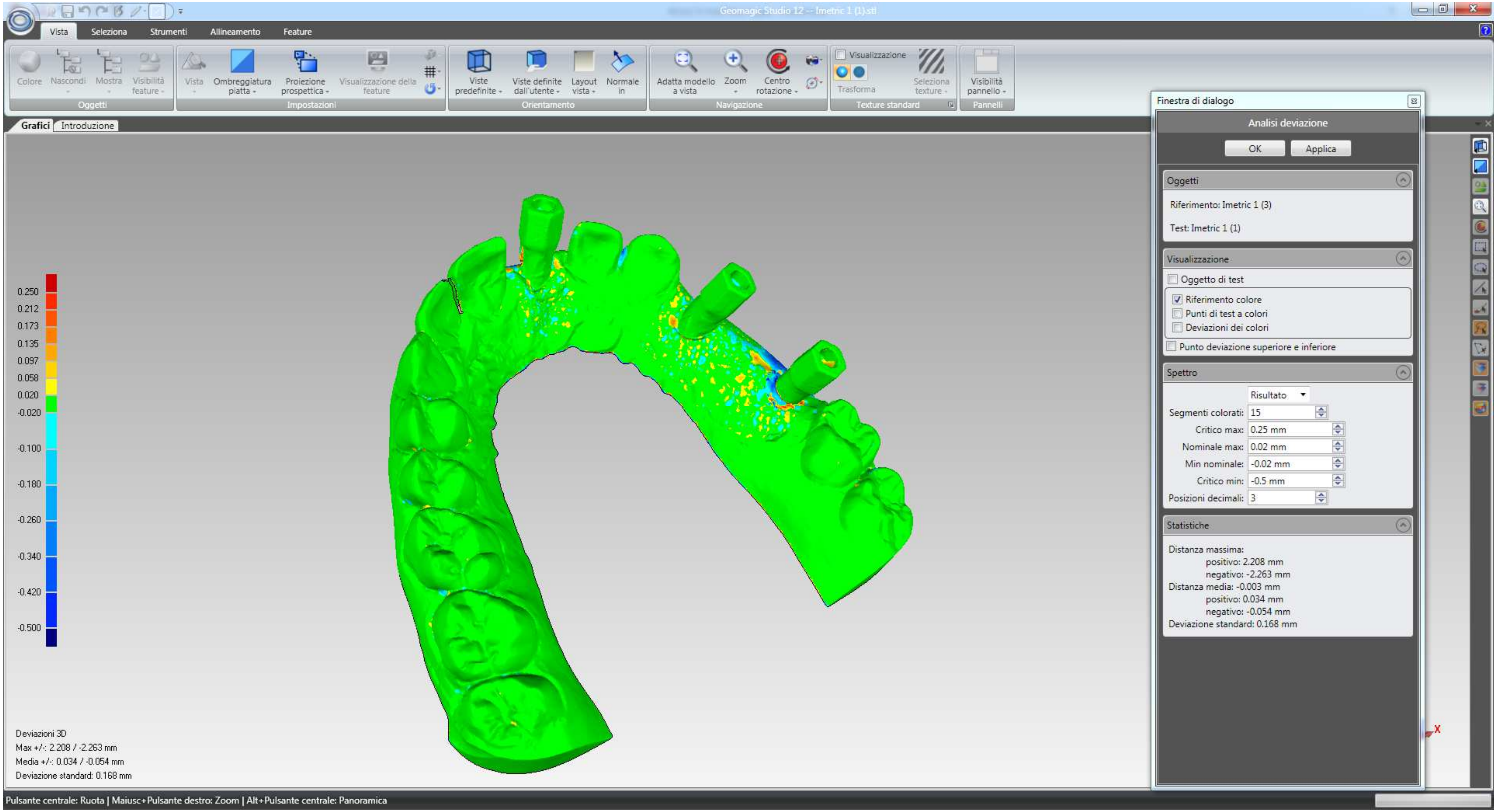

VALIDATION OF THE MANUFACTURER DATA

TOTALLY EDENTULOUS MODEL

Values setting: 0.25, 0.02, -0.02, -0.25

3 different scans with Imetric are taken and superimposed each other,

IN ORDER TO VALIDATE THE MANUFACTURER'S DATA.

The final result was a precision of 14.4 ± 7.9 micrometers.

GENERAL TRUENESS AND PRECISION. VALIDITY/RELIABILITY OF THE REGISTRATION METHODS. CALIBRATION

Before to start with the superimposition of 3D models, for the evaluation of general trueness and precision, the validity of the method was tested, and the following operations were made for both the partially and totally edentulous models. In brief, the reference R1 model was imported in the reverse engineering software, duplicated and moved to another position in the space; these two identical models were then superimposed and registered, and the software calculated the difference between the two surfaces. This test was repeated 5 times per each model (overall, 10 tests were made) and the final result was a negligible mean registration error (2.8 ± 3.0 nanometers in the partially edentulous maxilla; 3.2 ± 1.7 nanometers in the fully edentulous maxilla): this certified the reliability of the procedure.

In the following slides you can find the validation process. We started with the 5 tests for the partially edentulous model, and we complete the calibration with the completely edentulous model.

Step 1: the reference R1 model was imported in the reverse engineering software, duplicated and moved to another location

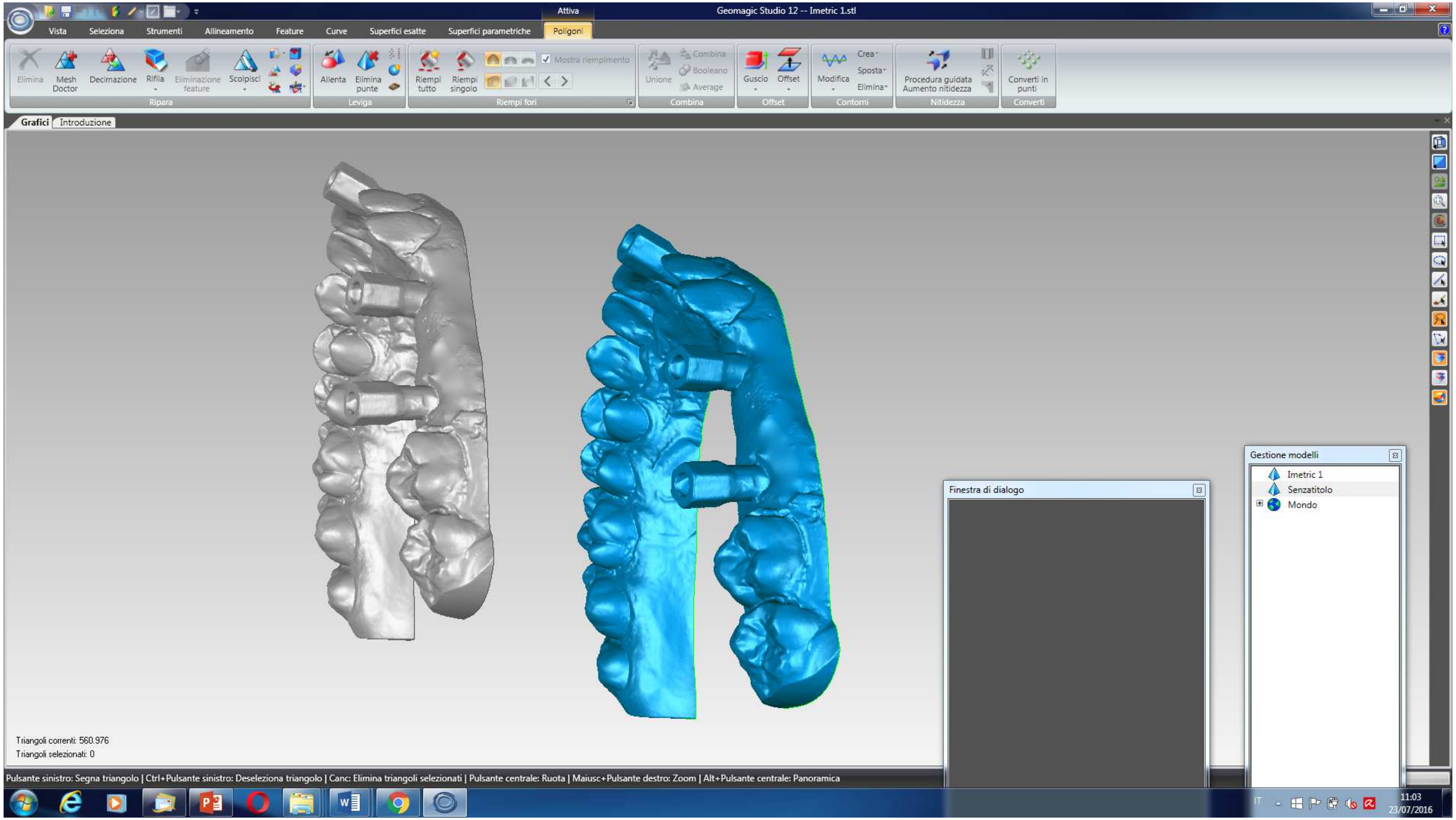

These two identical models were then superimposed and registered, first with the “three point registration..”

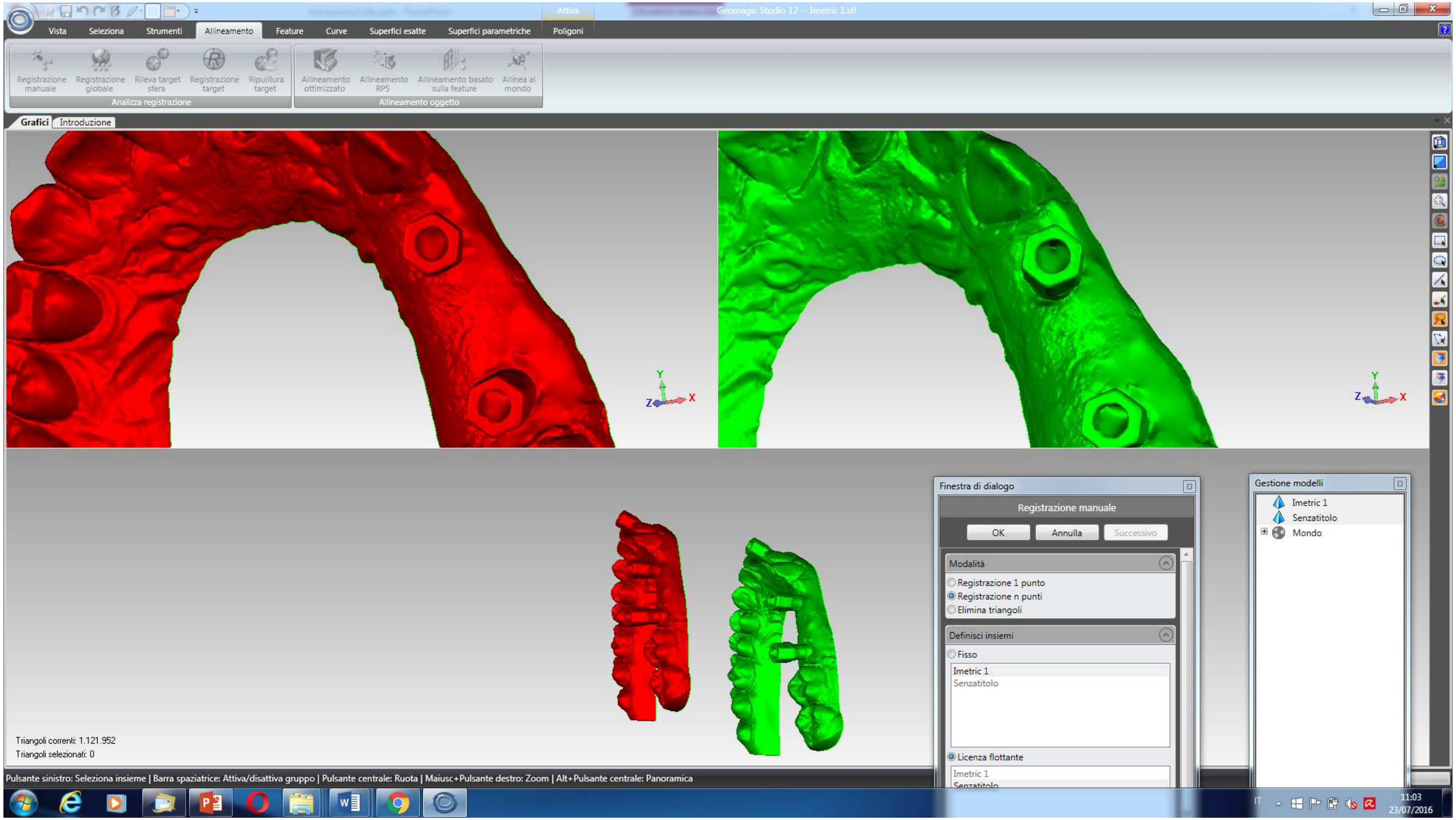

..then with the robust-iterative-closest-point (RICP), which was used for the final superimposition..

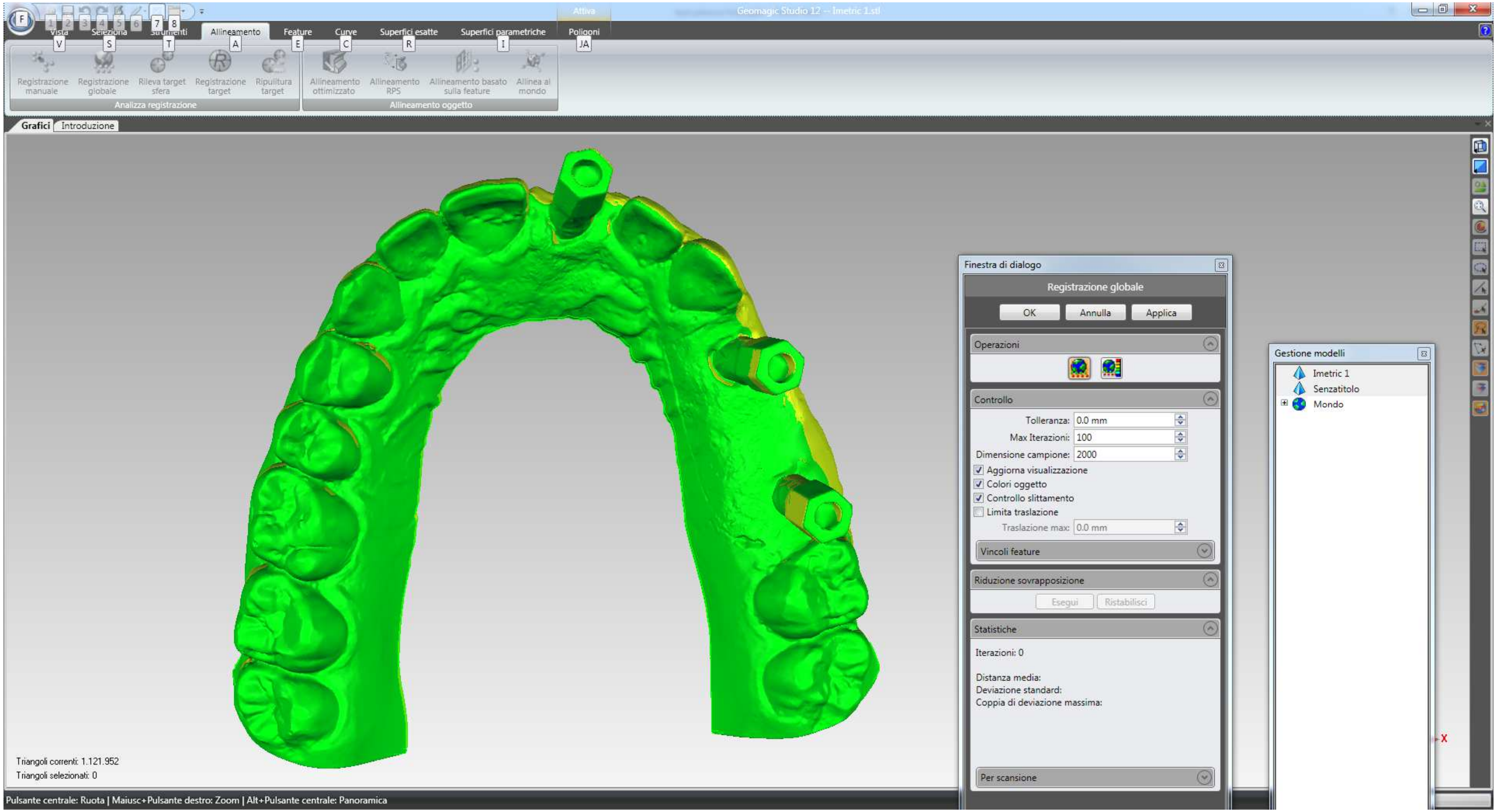

Then with the robust-iterative-closest-point (RICP), which was used for the final superimposition..

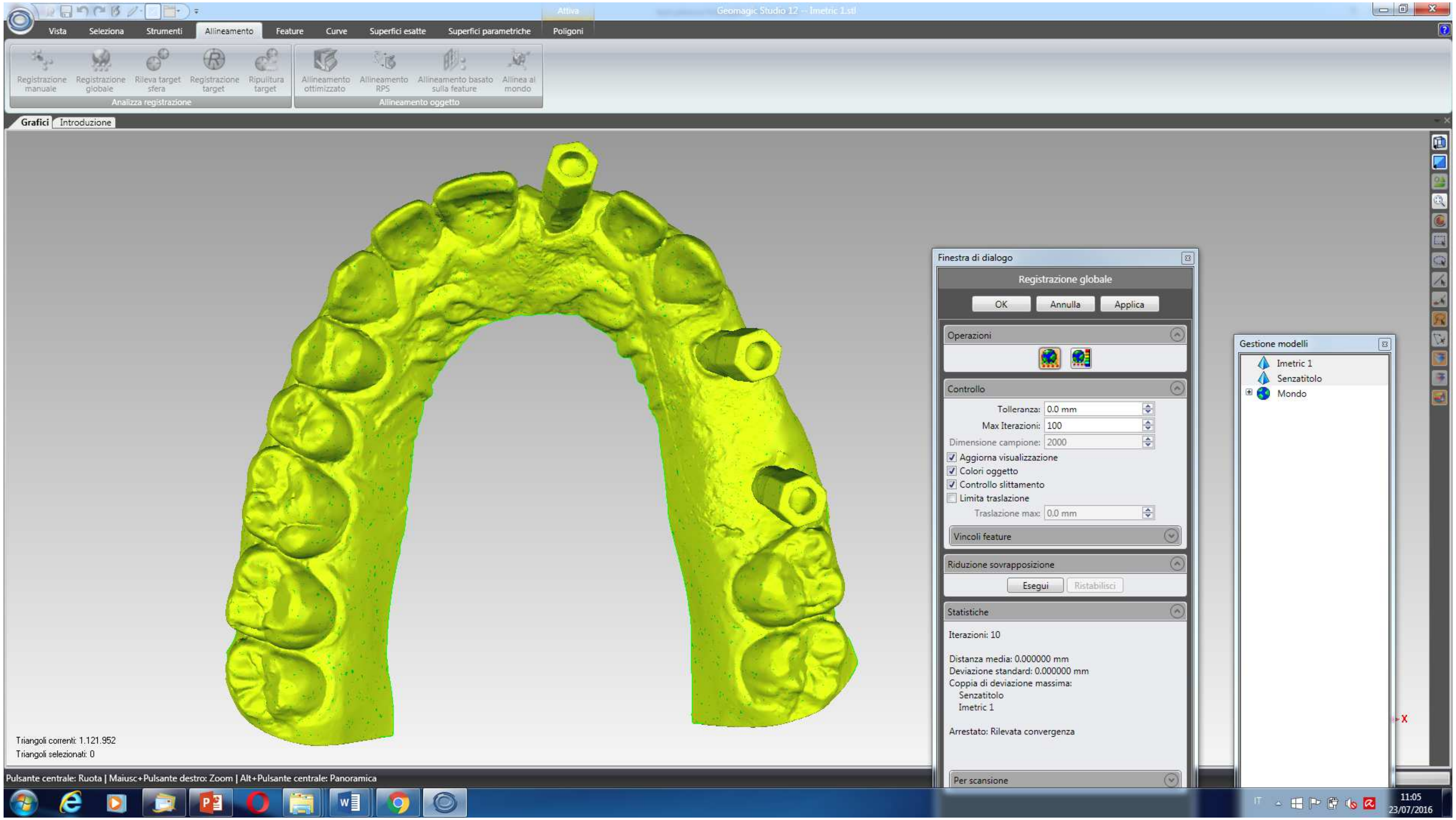

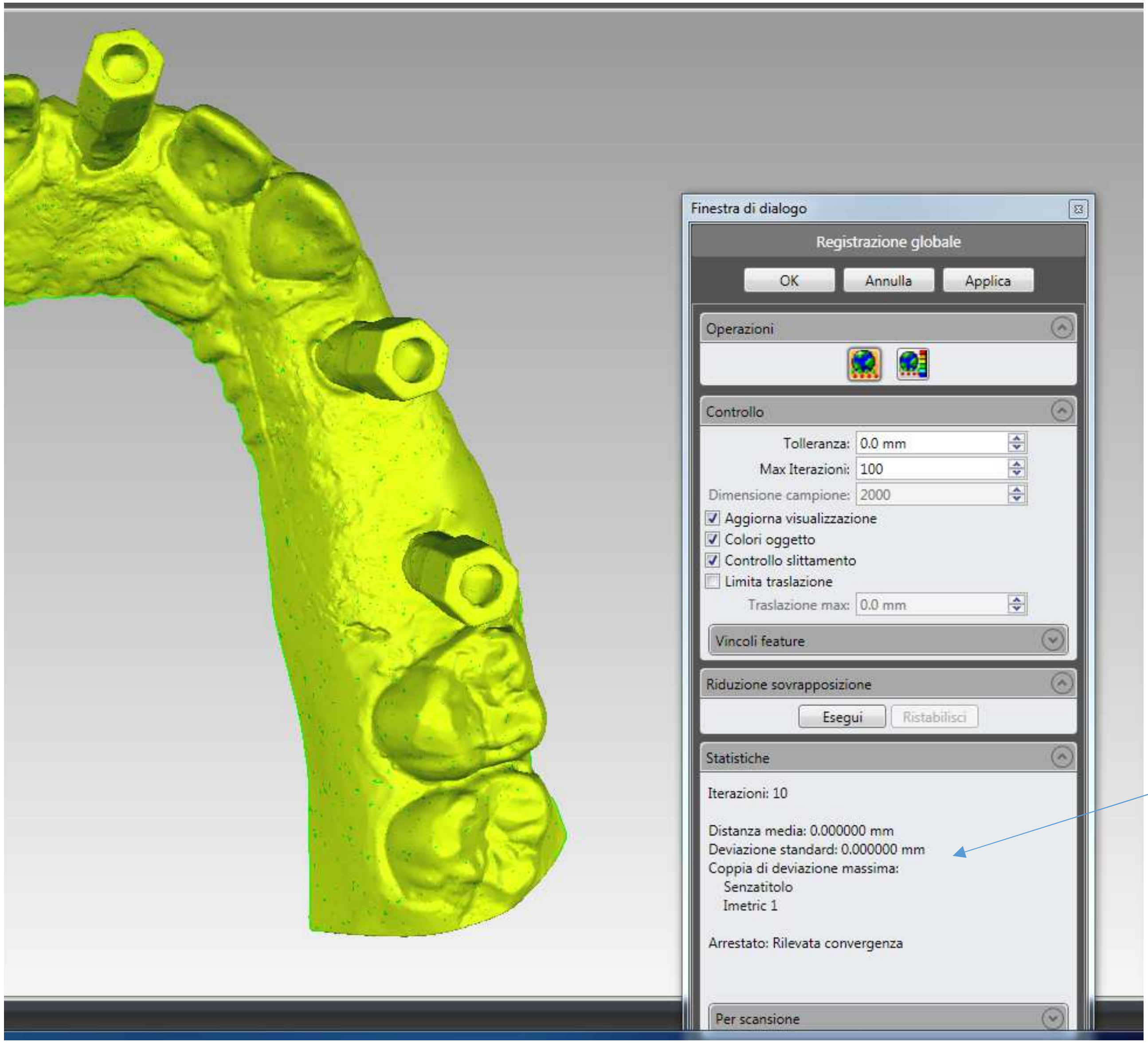

In this first test, the error was 0 nm

The overlapping was perfect

Then the same test was repeated 4 times in order to verify the validity of the registration/ overlapping method, for the same model (the partially edentulous model)

Test n° 2

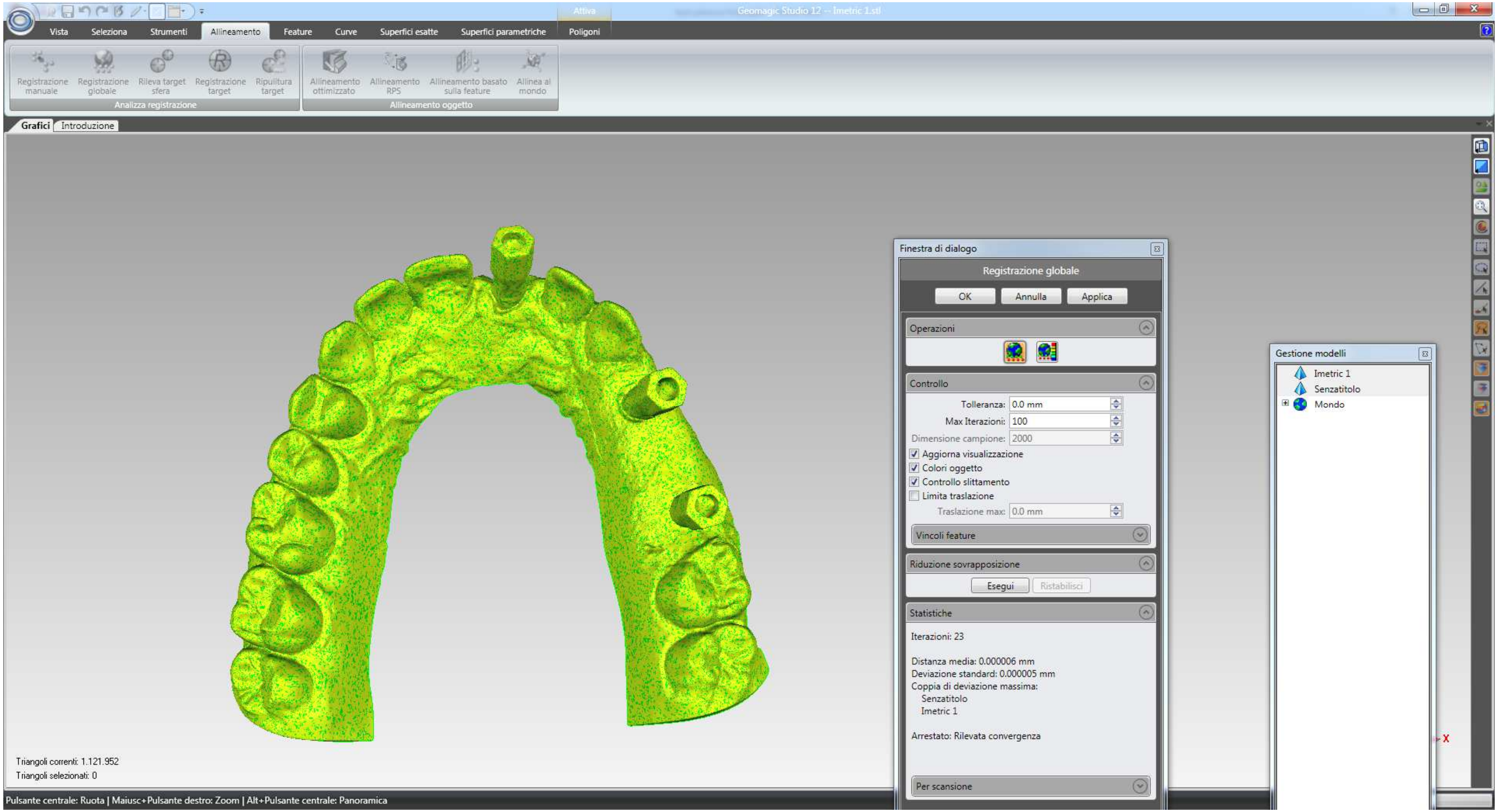
Geomagic Studio 12 -- Imetric 1.stl
Vista
Seleziona
Strumenti
Allineamento
Feature
Curve
Superfici esatte
Superfici parametriche
Poligoni
Registrazione manuale
Registrazione globale
Rileva target sfera
Registrazione target
Ripulitura target
Analizza registrazione
Allineamento ottimizzato
Allineamento RPS
Allineamento basato sulla feature
Allinea al mondo
Allineamento oggetto
Grafici
Introduzione
Finestra di dialogo
Registrazione globale
OK
Annulla
Applica
Operazioni
Controllo
Tolleranza: 0.0 mm
Max Iterazioni: 100
Dimensione campione: 2000
Aggiorna visualizzazione
Colori oggetto
Controllo slittamento
Limita traslazione
Traslazione max: 0.0 mm
Vincoli feature
Riduzione sovrapposizione
Esegui
Ristabilisci
Statistiche
Iterazioni: 23
Distanza media: 0.000006 mm
Deviazione standard: 0.000005 mm
Coppia di deviazione massima:
Senzatitolo
Imetric 1
Arrestato: Rilevata convergenza
Per scansione
Gestione modelli
Imetric 1
Senzatitolo
Mondo
Triangoli correnti: 1.121.952
Triangoli selezionati: 0
Pulsante centrale: Ruota | Maiusc+Pulsante destro: Zoom | Alt+Pulsante centrale: Panoramica

Test n° 3

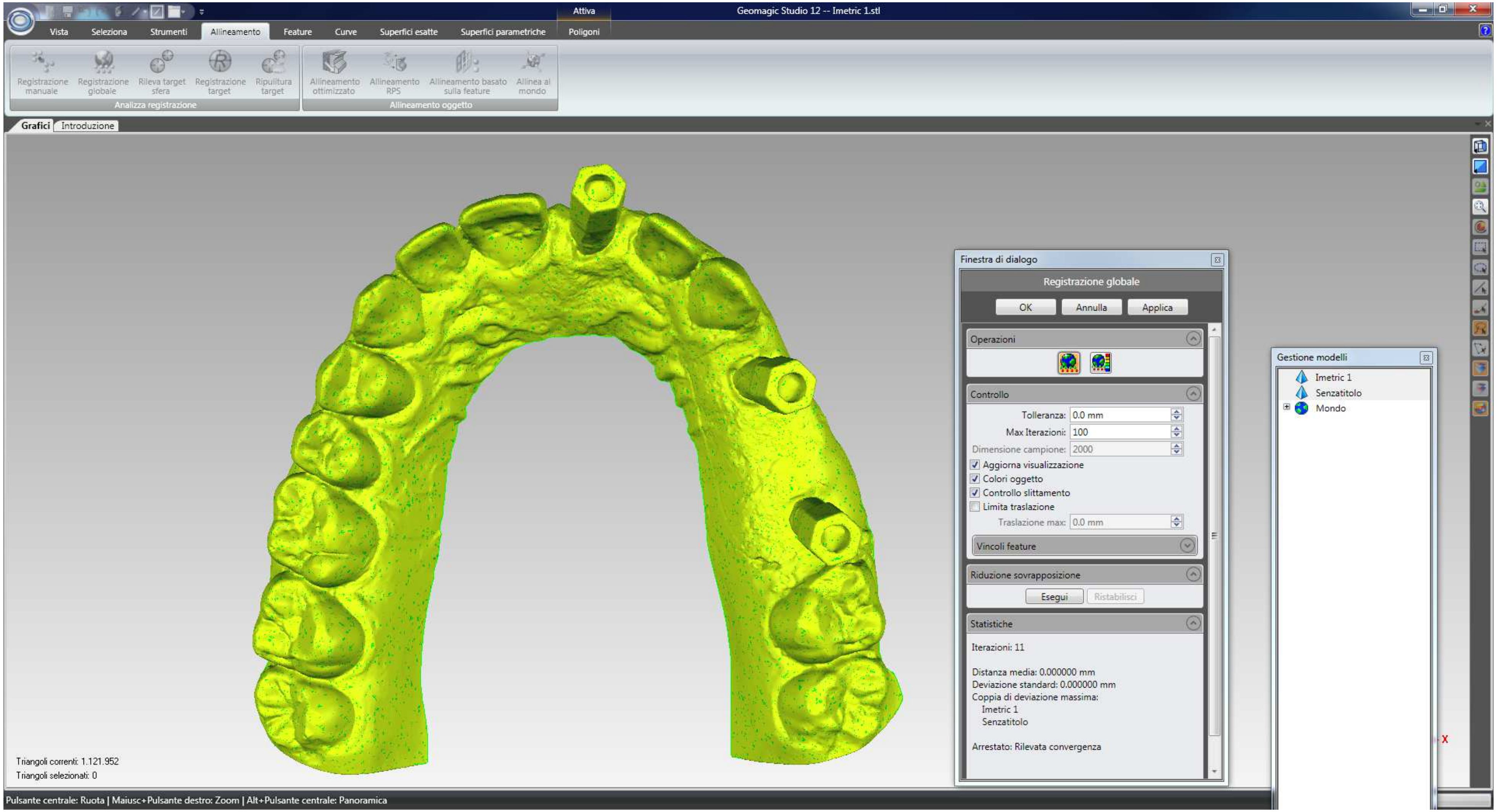
Geomagic Studio 12 -- Imetric 1.stl
Attiva
Vista
Seleziona
Strumenti
Allineamento
Feature
Curve
Superfici esatte
Superfici parametriche
Poligoni
Registrazione manuale
Registrazione globale
Rileva target sfera
Registrazione target
Ripulitura target
Analizza registrazione
Allineamento ottimizzato
Allineamento RPS
Allineamento basato sulla feature
Allinea al mondo
Allineamento oggetto
Grafici
Introduzione
Finestra di dialogo
Registrazione globale
OK
Annulla
Applica
Operazioni
Controllo
Tolleranza: 0.0 mm
Max Iterazioni: 100
Dimensione campione: 2000
Aggiorna visualizzazione
Colori oggetto
Controllo slittamento
Limita traslazione
Traslazione max: 0.0 mm
Vincoli feature
Riduzione sovrapposizione
Esegui
Ristabilisci
Statistiche
Iterazioni: 11
Distanza media: 0.000000 mm
Deviazione standard: 0.000000 mm
Coppia di deviazione massima:
Imetric 1
Senzatitolo
Arrestato: Rilevata convergenza
Gestione modelli
Imetric 1
Senzatitolo
Mondo
Triangoli correnti: 1.121.952
Triangoli selezionati: 0
Pulsante centrale: Ruota | Maiusc+Pulsante destro: Zoom | Alt+Pulsante centrale: Panoramica

Test n° 4

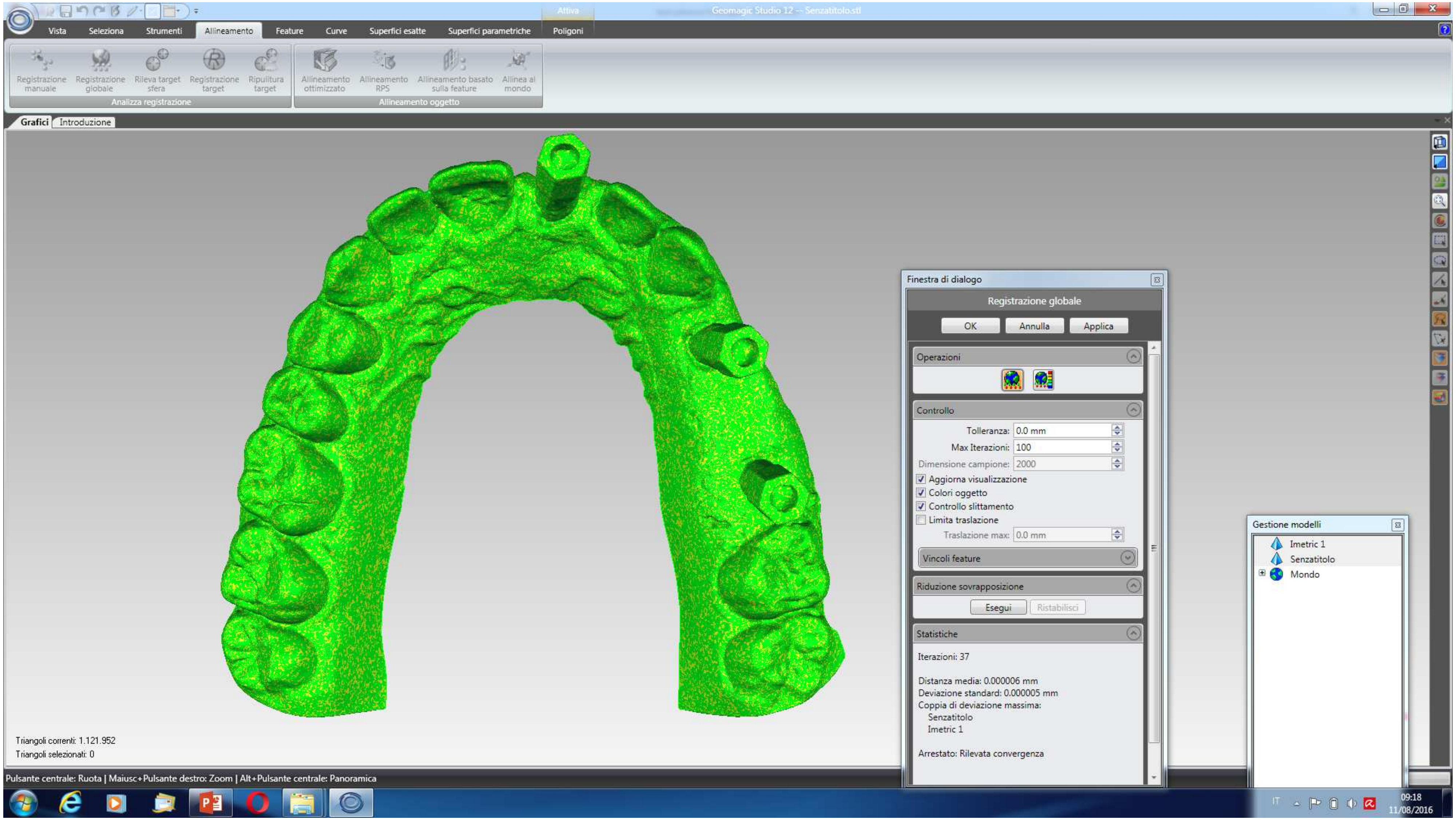
Geomagic Studio 12 -- Senzatitolo.stl
Vista
Seleziona
Strumenti
Allineamento
Feature
Curve
Superfici esatte
Superfici parametriche
Poligoni
Registrazione manuale
Registrazione globale
Rileva target sfera
Registrazione target
Ripulitura target
Analizza registrazione
Allineamento ottimizzato
Allineamento RPS
Allineamento basato sulla feature
Allinea al mondo
Allineamento oggetto
Grafici
Introduzione
Finestra di dialogo
Registrazione globale
OK
Annulla
Applica
Operazioni
Controllo
Tolleranza: 0.0 mm
Max Iterazioni: 100
Dimensione campione: 2000
Aggiorna visualizzazione
Colori oggetto
Controllo slittamento
Limita traslazione
Traslazione max: 0.0 mm
Vincoli feature
Riduzione sovrapposizione
Esegui
Ristabilisci
Statistiche
Iterazioni: 37
Distanza media: 0.000006 mm
Deviazione standard: 0.000005 mm
Coppia di deviazione massima:
Senzatitolo
Imetric 1
Arrestato: Rilevata convergenza
Gestione modelli
Imetric 1
Senzatitolo
Mondo
Triangoli correnti: 1.121.952
Triangoli selezionati: 0
Pulsante centrale: Ruota | Maiusc+Pulsante destro: Zoom | Alt+Pulsante centrale: Panoramica
09:18
11/08/2016

Test n° 5

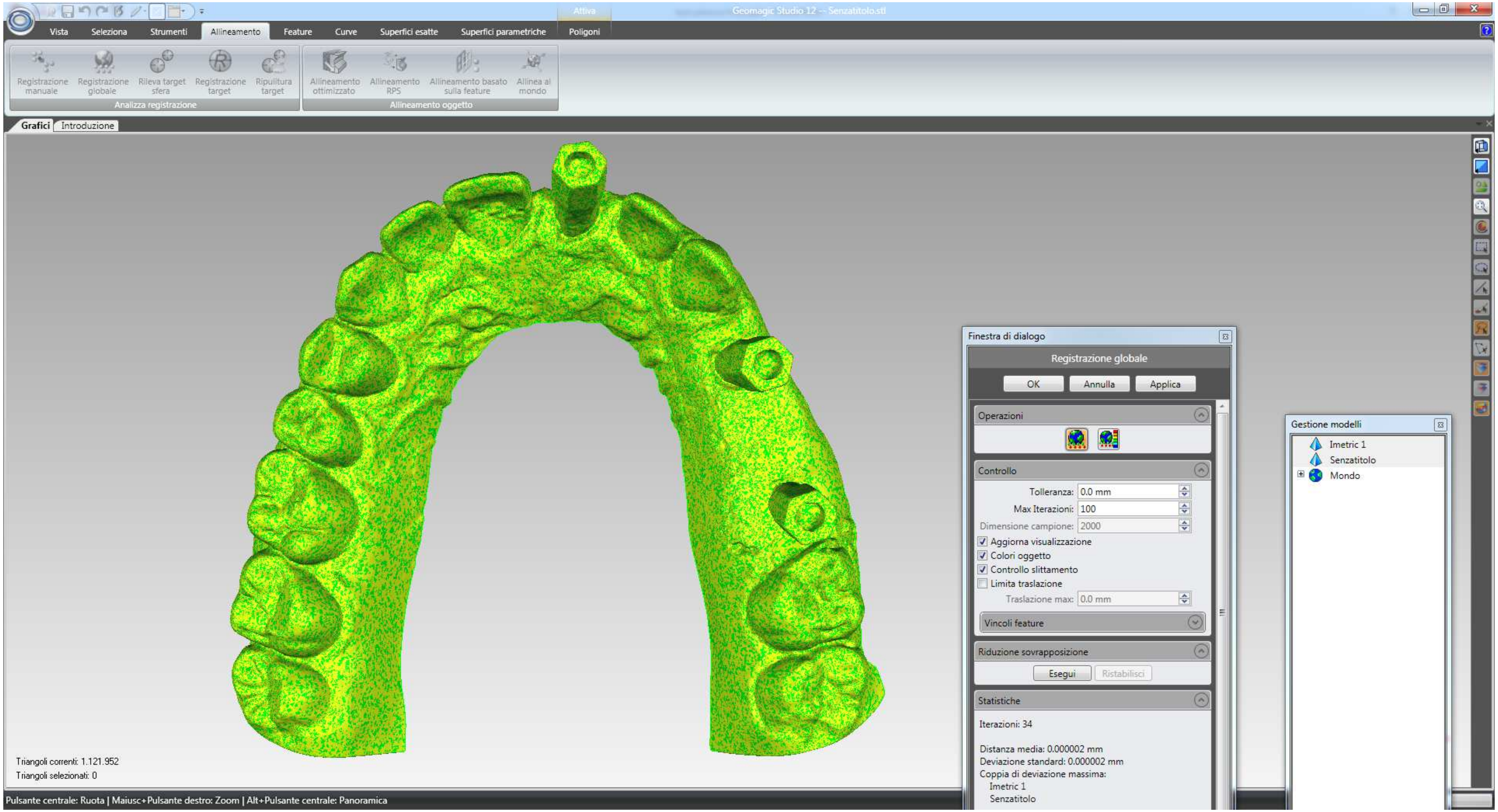

Geomagic Studio 12 -- Senzatitolo.stl
Vista
Seleziona
Strumenti
Allineamento
Feature
Curve
Superfici esatte
Superfici parametriche
Poligoni
Registrazione manuale
Registrazione globale
Rileva target sfera
Registrazione target
Ripulitura target
Analizza registrazione
Allineamento ottimizzato
Allineamento RPS
Allineamento basato sulla feature
Allinea al mondo
Allineamento oggetto
Grafici
Introduzione
Finestra di dialogo
Registrazione globale
OK
Annulla
Applica
Operazioni
Controllo
Tolleranza: 0.0 mm
Max Iterazioni: 100
Dimensione campione: 2000
Aggiorna visualizzazione
Colori oggetto
Controllo slittamento
Limita traslazione
Traslazione max: 0.0 mm
Vincoli feature
Riduzione sovrapposizione
Esegui
Ristabilisci
Statistiche
Iterazioni: 34
Distanza media: 0.000002 mm
Deviazione standard: 0.000002 mm
Coppia di deviazione massima:
Imetric 1
Senzatitolo
Gestione modelli
Imetric 1
Senzatitolo
Mondo
Triangoli correnti: 1.121.952
Triangoli selezionati: 0
Pulsante centrale: Ruota | Maiusc+Pulsante destro: Zoom | Alt+Pulsante centrale: Panoramica

VALIDATION ERRORS IN THE PARTIALLY EDENTULOUS MODEL: RESULTS FROM THE 5 AFOREMENTIONED TESTS AND THE OVERALL MEAN AND SD

	Mean	SD
Test 1	0 nm	0 nm
Test 2	6 nm	5 nm
Test 3	0 nm	0 nm
Test 4	6 nm	5 nm
Test 5	2 nm	2 nm
Overall	2.8 (3.0) nm	

The same procedure was performed with 5 validation test for the fully edentulous model.

Test n° 1 TOTALLY EDENTULOUS MODEL

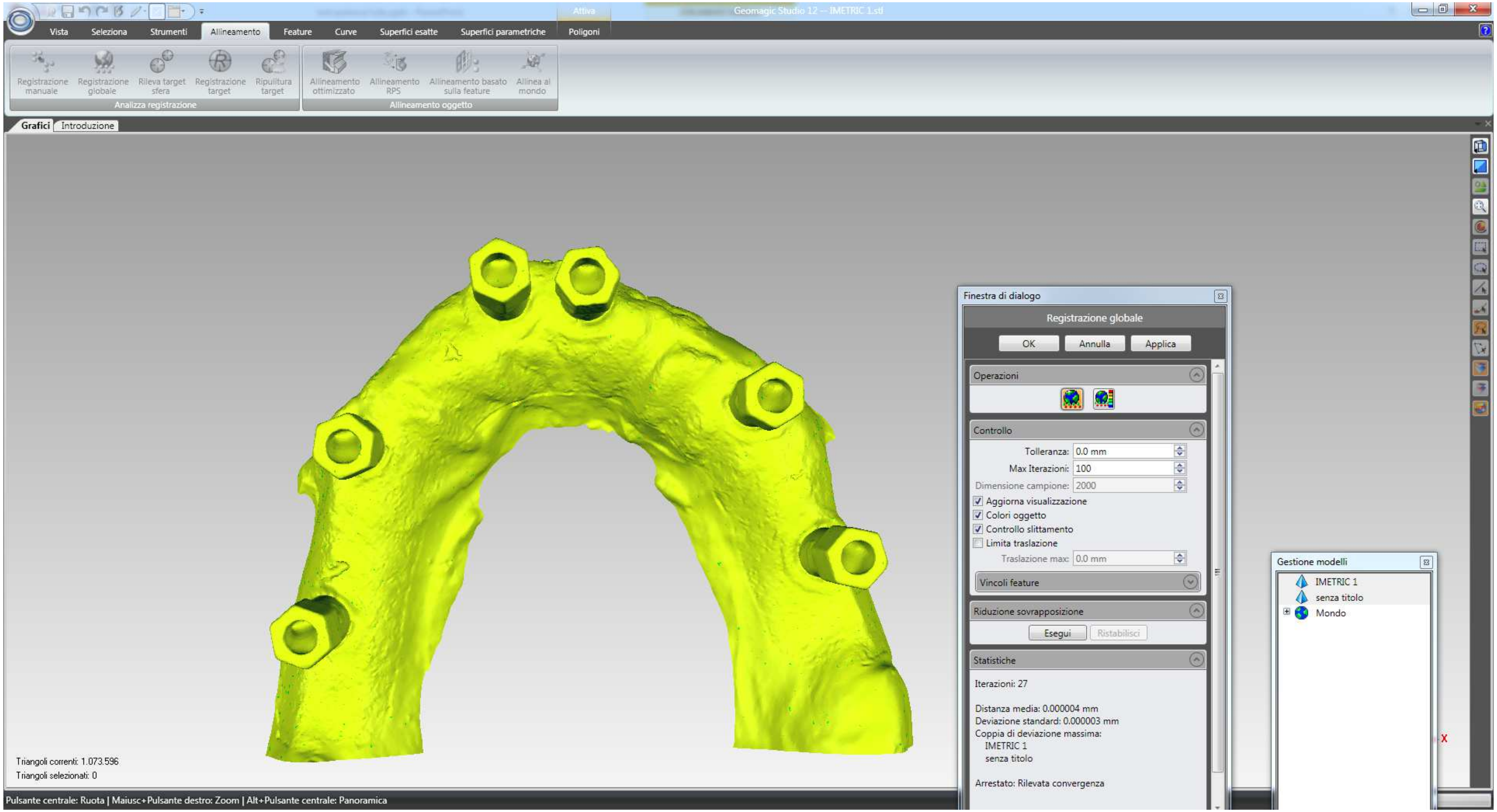

Test n° 2

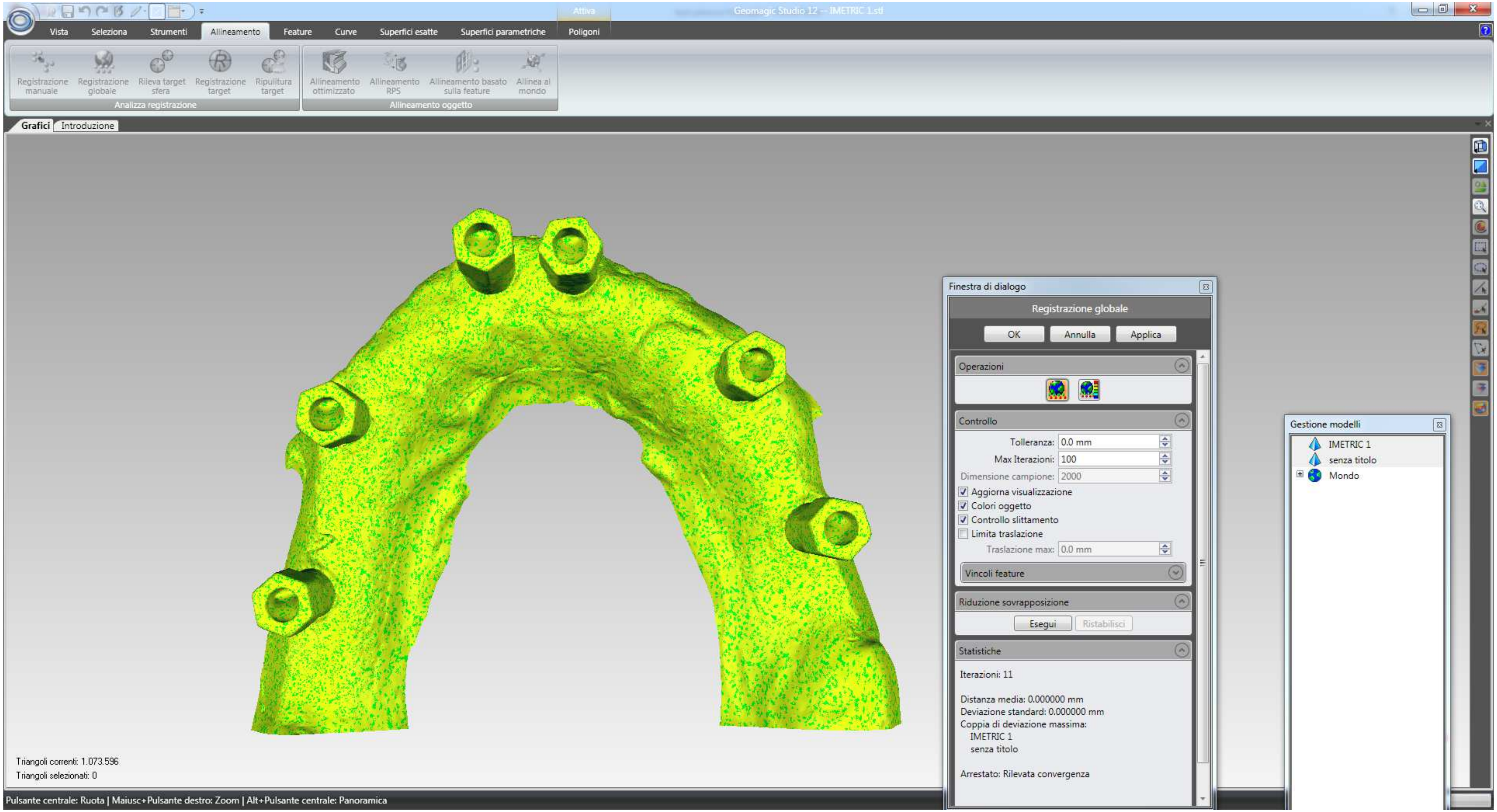
Geomagic Studio 12 -- IMETRIC 1.stl
Vista
Seleziona
Strumenti
Allineamento
Feature
Curve
Superfici esatte
Superfici parametriche
Poligoni
Registrazione manuale
Registrazione globale
Rileva target sfera
Registrazione target
Ripulitura target
Allineamento ottimizzato
Allineamento RPS
Allineamento basato sulla feature
Allinea al mondo
Analizza registrazione
Allineamento oggetto
Grafici
Introduzione
Finestra di dialogo
Registrazione globale
OK
Annulla
Applica
Operazioni
Controllo
Tolleranza: 0.0 mm
Max Iterazioni: 100
Dimensione campione: 2000
Aggiorna visualizzazione
Colori oggetto
Controllo slittamento
Limita traslazione
Traslazione max: 0.0 mm
Vincoli feature
Riduzione sovrapposizione
Esegui
Ristabilisci
Statistiche
Iterazioni: 11
Distanza media: 0.000000 mm
Deviazione standard: 0.000000 mm
Coppia di deviazione massima:
IMETRIC 1
senza titolo
Arrestato: Rilevata convergenza
Gestione modelli
IMETRIC 1
senza titolo
Mondo
Triangoli correnti: 1.073.596
Triangoli selezionati: 0
Pulsante centrale: Ruota | Maiusc+Pulsante destro: Zoom | Alt+Pulsante centrale: Panoramica

Test n° 3

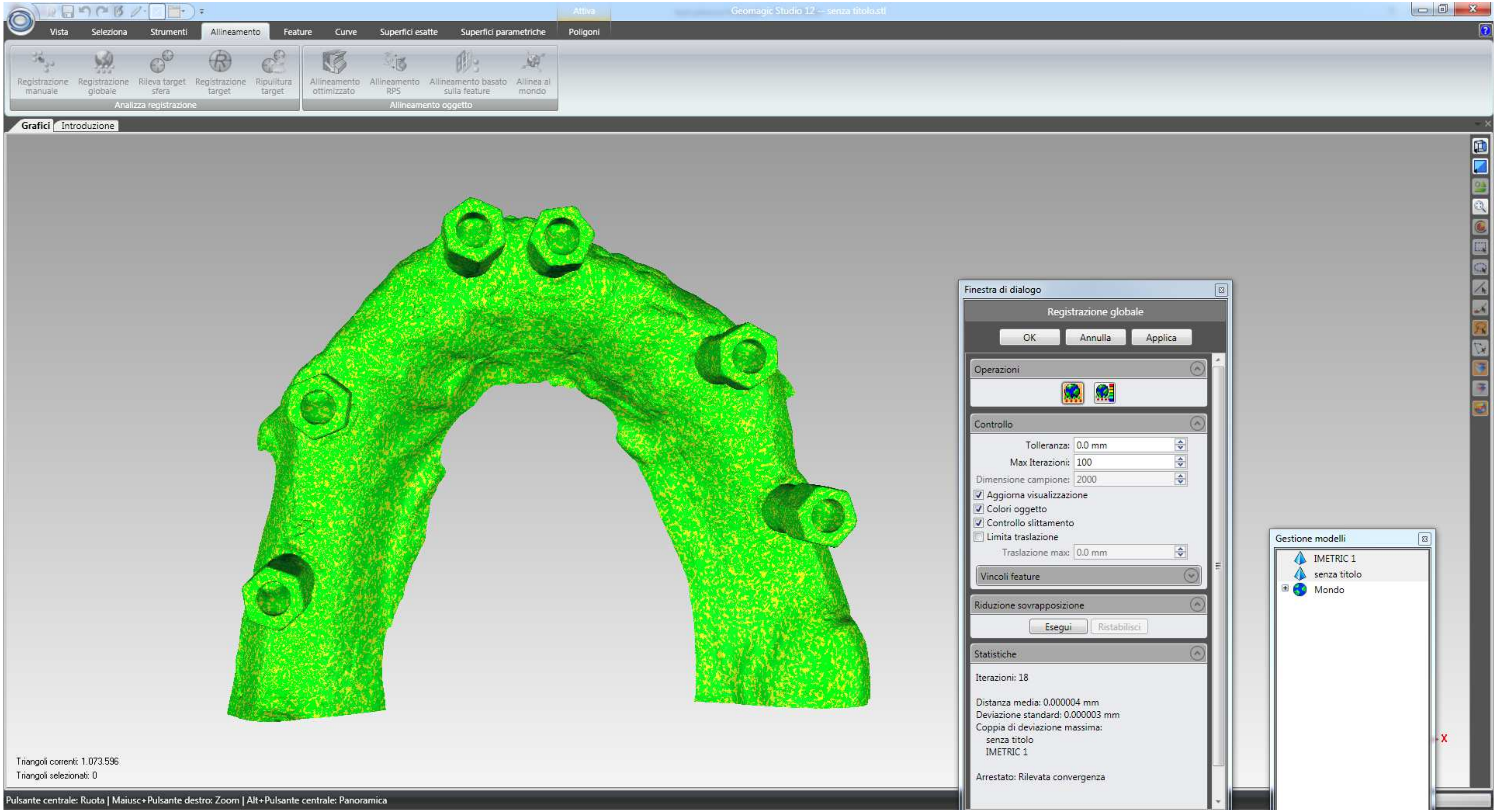

Geomagic Studio 12 -- senza titolo.stl
Attiva
Vista
Seleziona
Strumenti
Allineamento
Feature
Curve
Superfici esatte
Superfici parametriche
Poligoni
Registrazione manuale
Registrazione globale
Rileva target sfera
Registrazione target
Ripulitura target
Analizza registrazione
Allineamento ottimizzato
Allineamento RPS
Allineamento basato sulla feature
Allinea al mondo
Allineamento oggetto
Grafici
Introduzione
Finestra di dialogo
Registrazione globale
OK
Annulla
Applica
Operazioni
Controllo
Tolleranza: 0.0 mm
Max Iterazioni: 100
Dimensione campione: 2000
Aggiorna visualizzazione
Colori oggetto
Controllo slittamento
Limita traslazione
Traslazione max: 0.0 mm
Vincoli feature
Riduzione sovrapposizione
Esegui
Ristabilisci
Statistiche
Iterazioni: 18
Distanza media: 0.000004 mm
Deviazione standard: 0.000003 mm
Coppia di deviazione massima:
senza titolo
IMETRIC 1
Arrestato: Rilevata convergenza
Gestione modelli
IMETRIC 1
senza titolo
Mondo
Triangoli correnti: 1.073.596
Triangoli selezionati: 0
Pulsante centrale: Ruota | Maiusc+Pulsante destro: Zoom | Alt+Pulsante centrale: Panoramica

Test n° 4

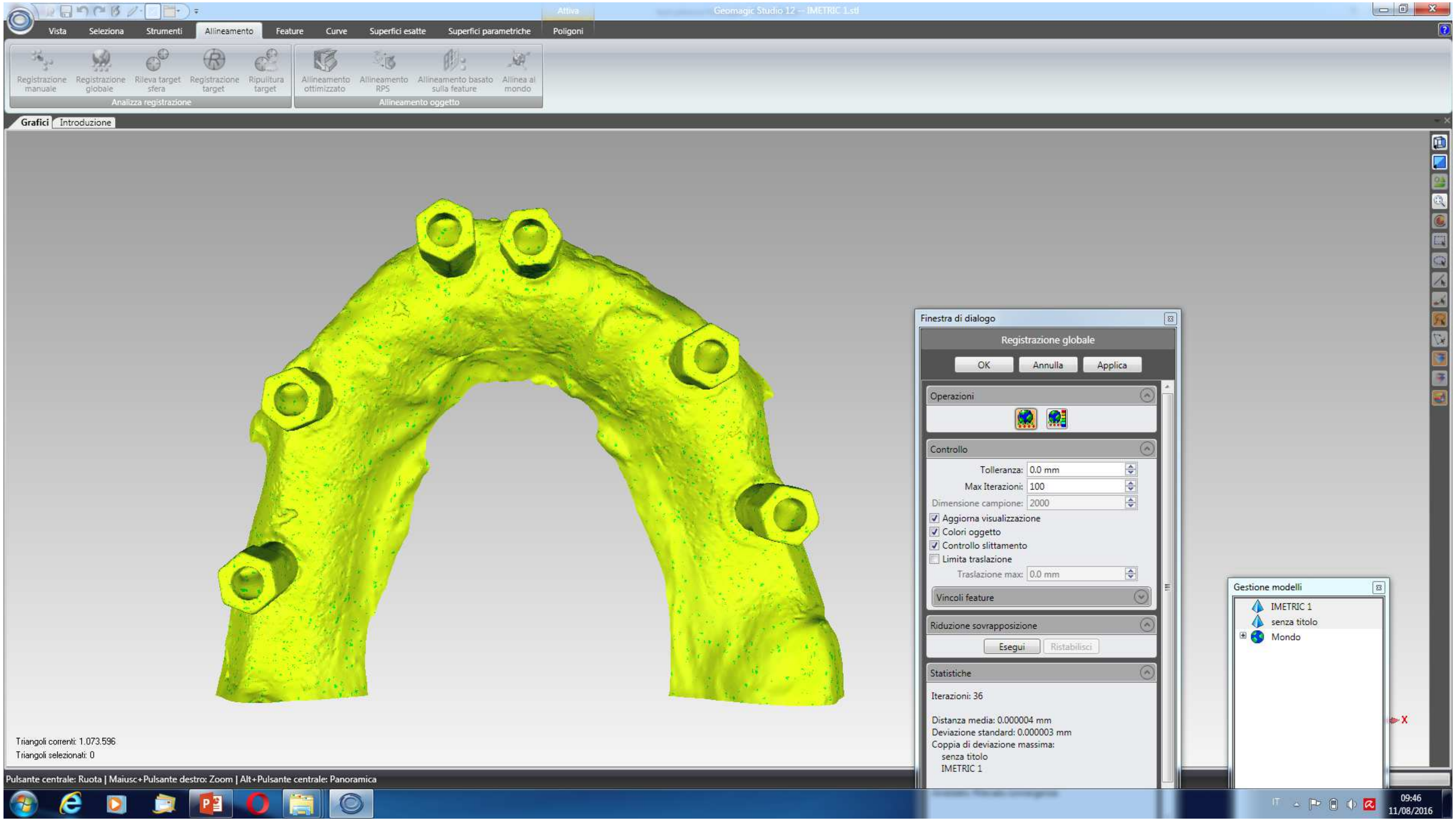
Geomagic Studio 12 -- IMETRIC 1.stl
Vista
Seleziona
Strumenti
Allineamento
Feature
Curve
Superfici esatte
Superfici parametriche
Poligoni
Registrazione manuale
Registrazione globale
Rileva target sfera
Registrazione target
Ripulitura target
Analizza registrazione
Allineamento ottimizzato
Allineamento RPS
Allineamento basato sulla feature
Allinea al mondo
Allineamento oggetto
Grafici
Introduzione
Finestra di dialogo
Registrazione globale
OK
Annulla
Applica
Operazioni
Controllo
Tolleranza: 0.0 mm
Max Iterazioni: 100
Dimensione campione: 2000
Aggiorna visualizzazione
Colori oggetto
Controllo slittamento
Limita traslazione
Traslazione max: 0.0 mm
Vincoli feature
Riduzione sovrapposizione
Esegui
Ristabilisci
Statistiche
Iterazioni: 36
Distanza media: 0.000004 mm
Deviazione standard: 0.000003 mm
Coppia di deviazione massima:
senza titolo
IMETRIC 1
Gestione modelli
IMETRIC 1
senza titolo
Mondo
Triangoli correnti: 1.073.596
Triangoli selezionati: 0
Pulsante centrale: Ruota | Maiusc+Pulsante destro: Zoom | Alt+Pulsante centrale: Panoramica
09:46
11/08/2016

Test n° 5

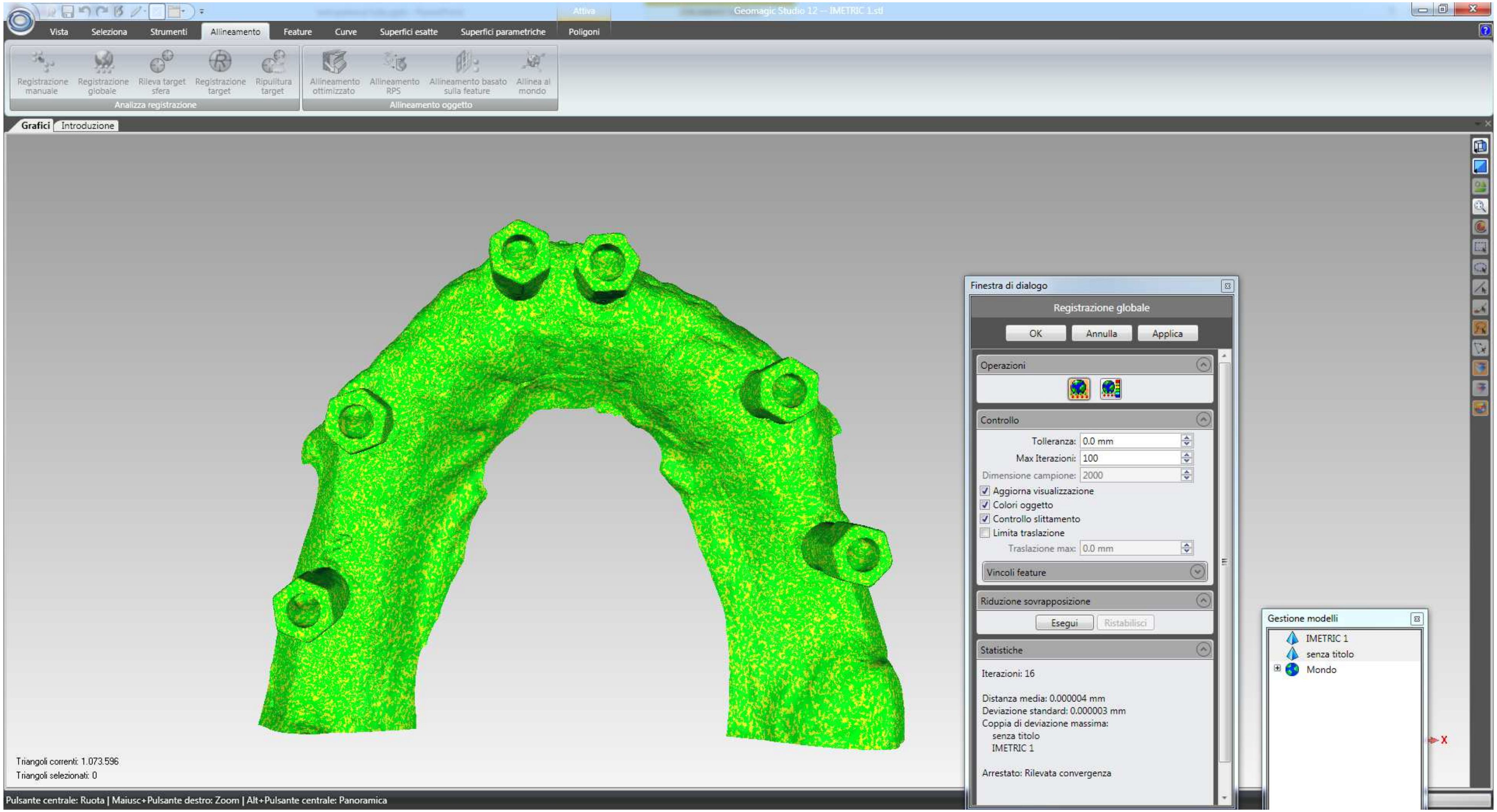
Geomagic Studio 12 -- IMETRIC 1.stl
Vista
Seleziona
Strumenti
Allineamento
Feature
Curve
Superfici esatte
Superfici parametriche
Poligoni
Registrazione manuale
Registrazione globale
Rileva target sfera
Registrazione target
Ripulitura target
Analizza registrazione
Allineamento ottimizzato
Allineamento RPS
Allineamento basato sulla feature
Allinea al mondo
Allineamento oggetto
Grafici
Introduzione
Finestra di dialogo
Registrazione globale
OK
Annulla
Applica
Operazioni
Controllo
Tolleranza: 0.0 mm
Max Iterazioni: 100
Dimensione campione: 2000
Aggiorna visualizzazione
Colori oggetto
Controllo slittamento
Limita traslazione
Traslazione max: 0.0 mm
Vincoli feature
Riduzione sovrapposizione
Esegui
Ristabilisci
Statistiche
Iterazioni: 16
Distanza media: 0.000004 mm
Deviazione standard: 0.000003 mm
Coppia di deviazione massima:
senza titolo
IMETRIC 1
Arrestato: Rilevata convergenza
Gestione modelli
IMETRIC 1
senza titolo
Mondo
Triangoli correnti: 1.073.596
Triangoli selezionati: 0
Pulsante centrale: Ruota | Maiusc+Pulsante destro: Zoom | Alt+Pulsante centrale: Panoramica

VALIDATION ERRORS IN THE TOTALLY EDENTULOUS MODEL: RESULTS FROM THE 5 AFOREMENTIONED TESTS AND THE OVERALL MEAN AND SD

	Mean	Ds
Test 1	4 nm	3 nm
Test 2	0 nm	0 nm
Test 3	4 nm	3 nm
Test 4	4 nm	3 nm
Test 5	4 nm	3 nm
Overall	3.2 (1.7) nm	

GENERAL TRUENESS EVALUATIONS FOR TRIOS, ZFX INTRASCAN, CARESTREAM, PLANSCAN (SCANS WERE RANDOM BUT HERE WE ORDERED THEM) IN THE PARTIALLY EDENTULOUS MODEL

GENERAL SETTINGS FOR FINAL REGISTRATION (TRUENESS):

0.5 mm, 0.05 mm, -0.05 mm, -0.5 mm (see scale)

TRIOS 1 vs REF IMETRIC

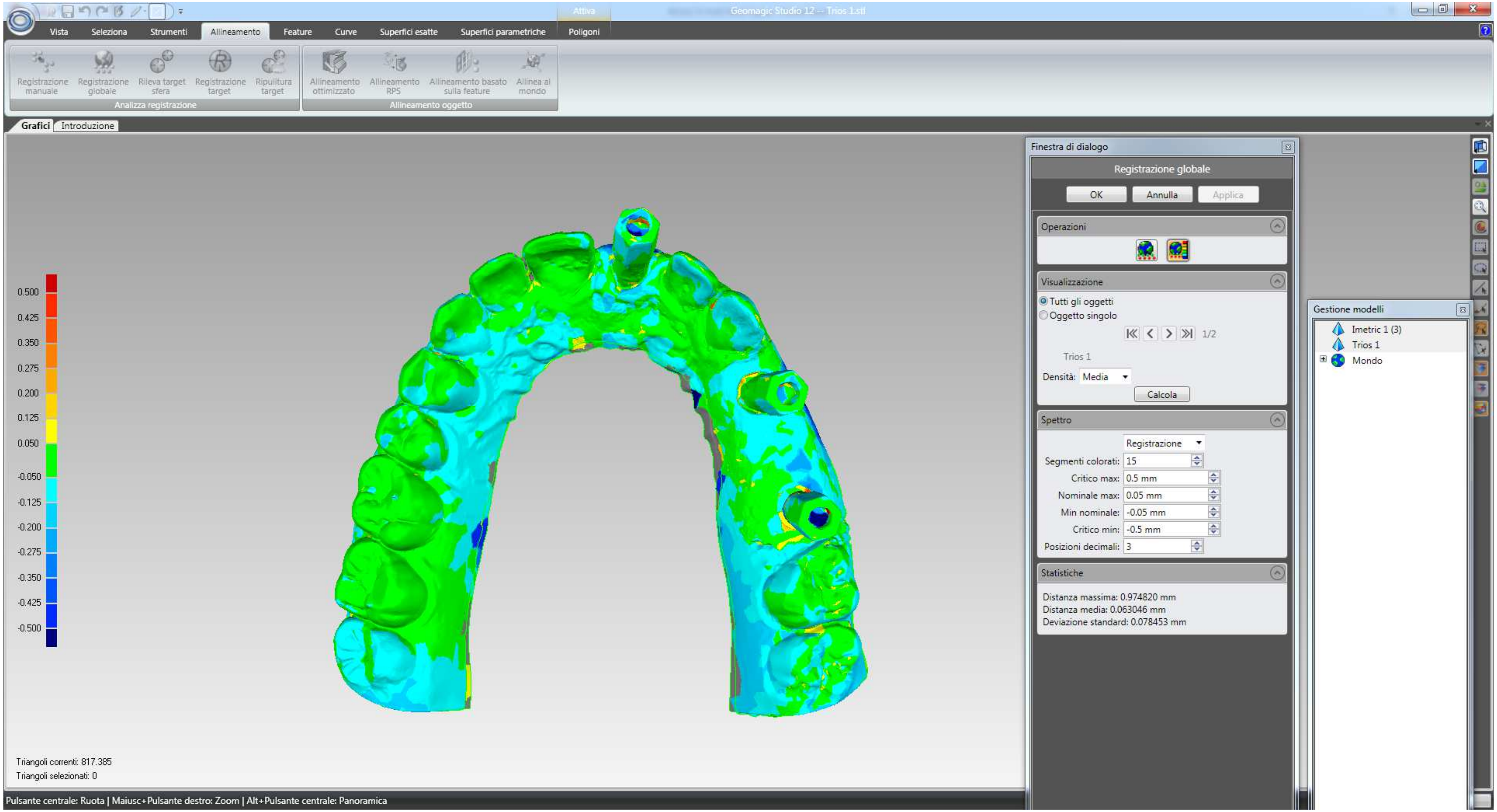

TRIOS 1 vs REF (IMETRIC)

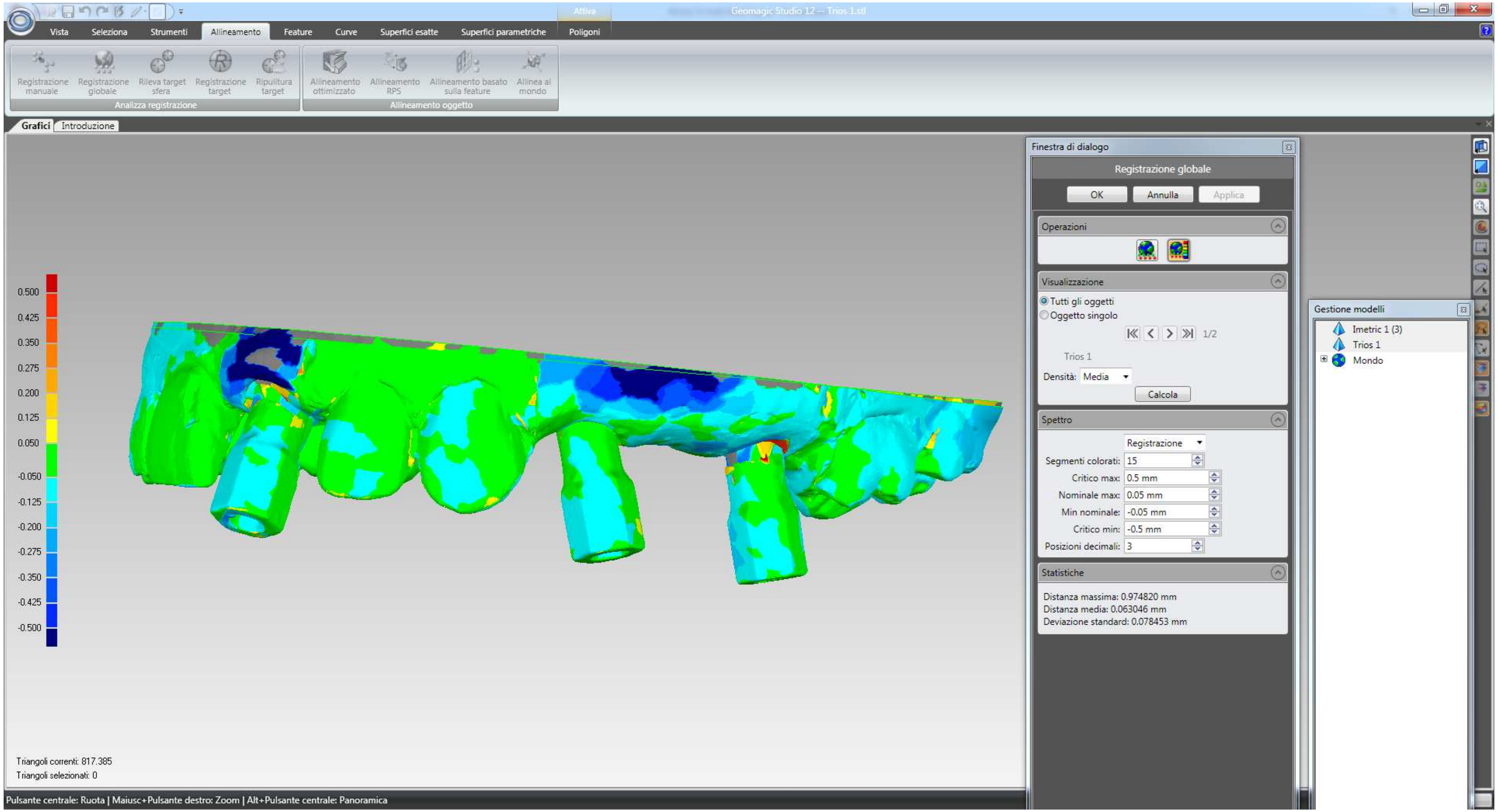

TRIOS 2 vs REF (IMETRIC)

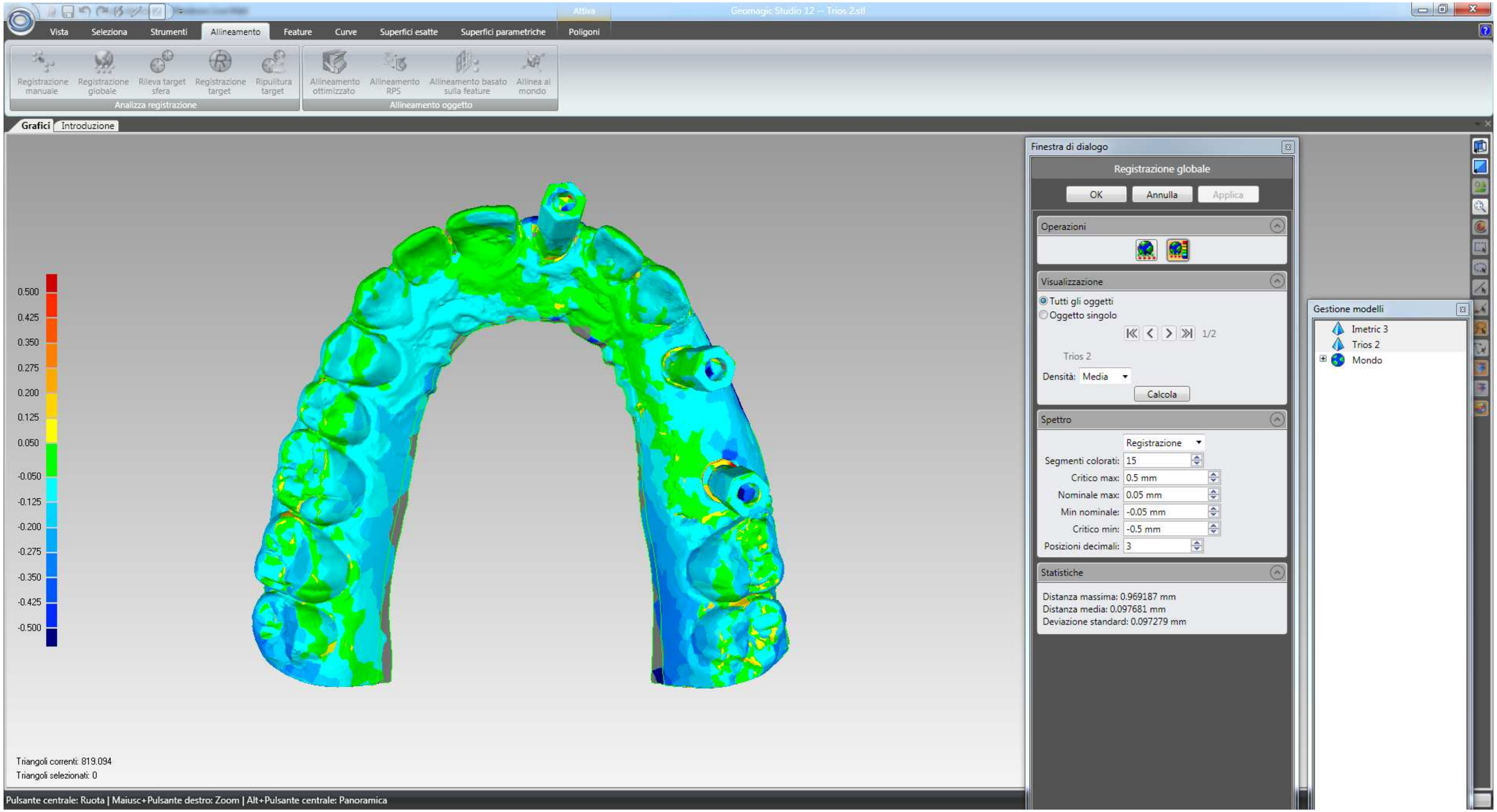

TRIOS 2 vs REF (IMETRIC)

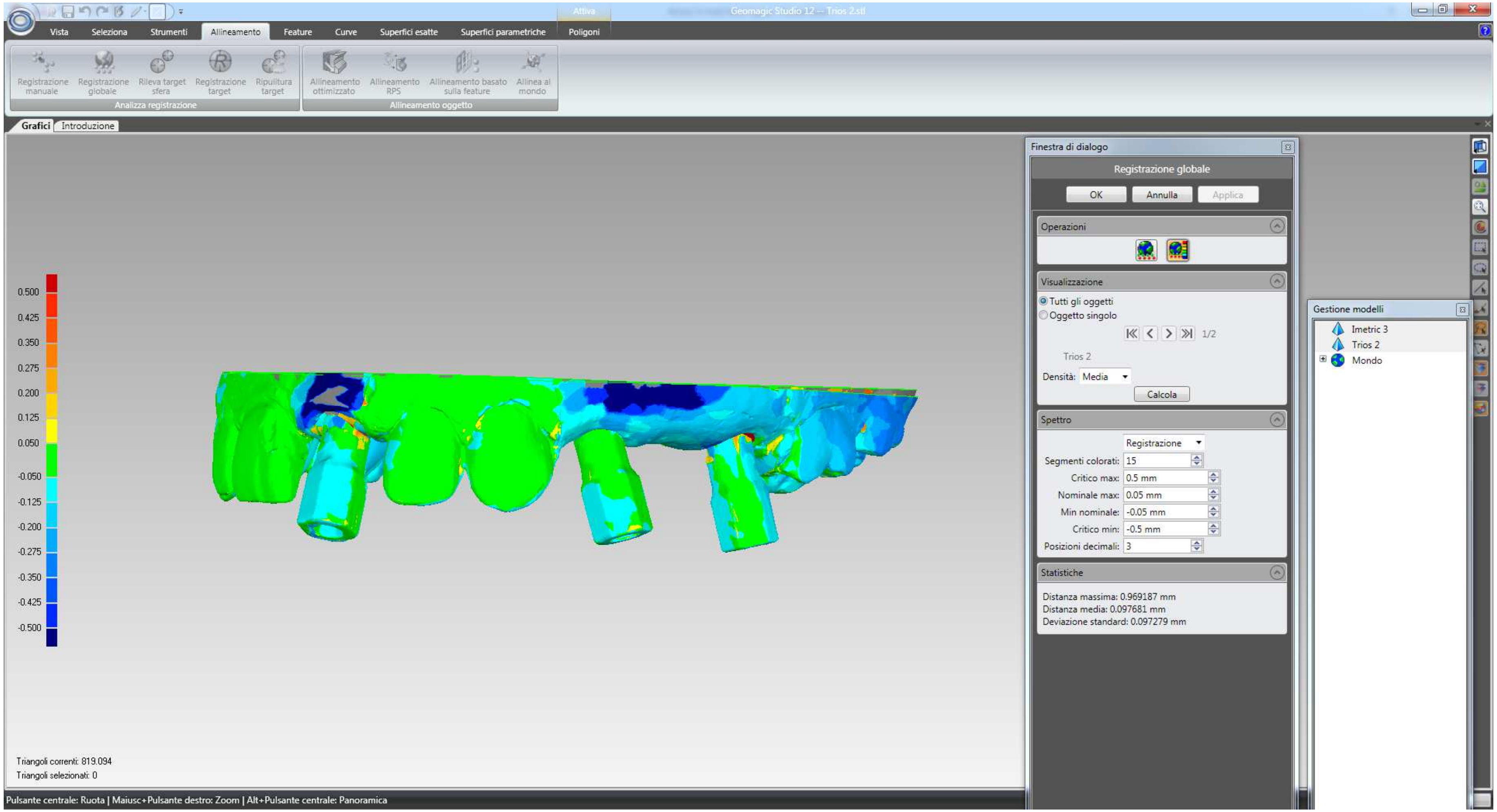

TRIOS 3 vs REF (IMETRIC)

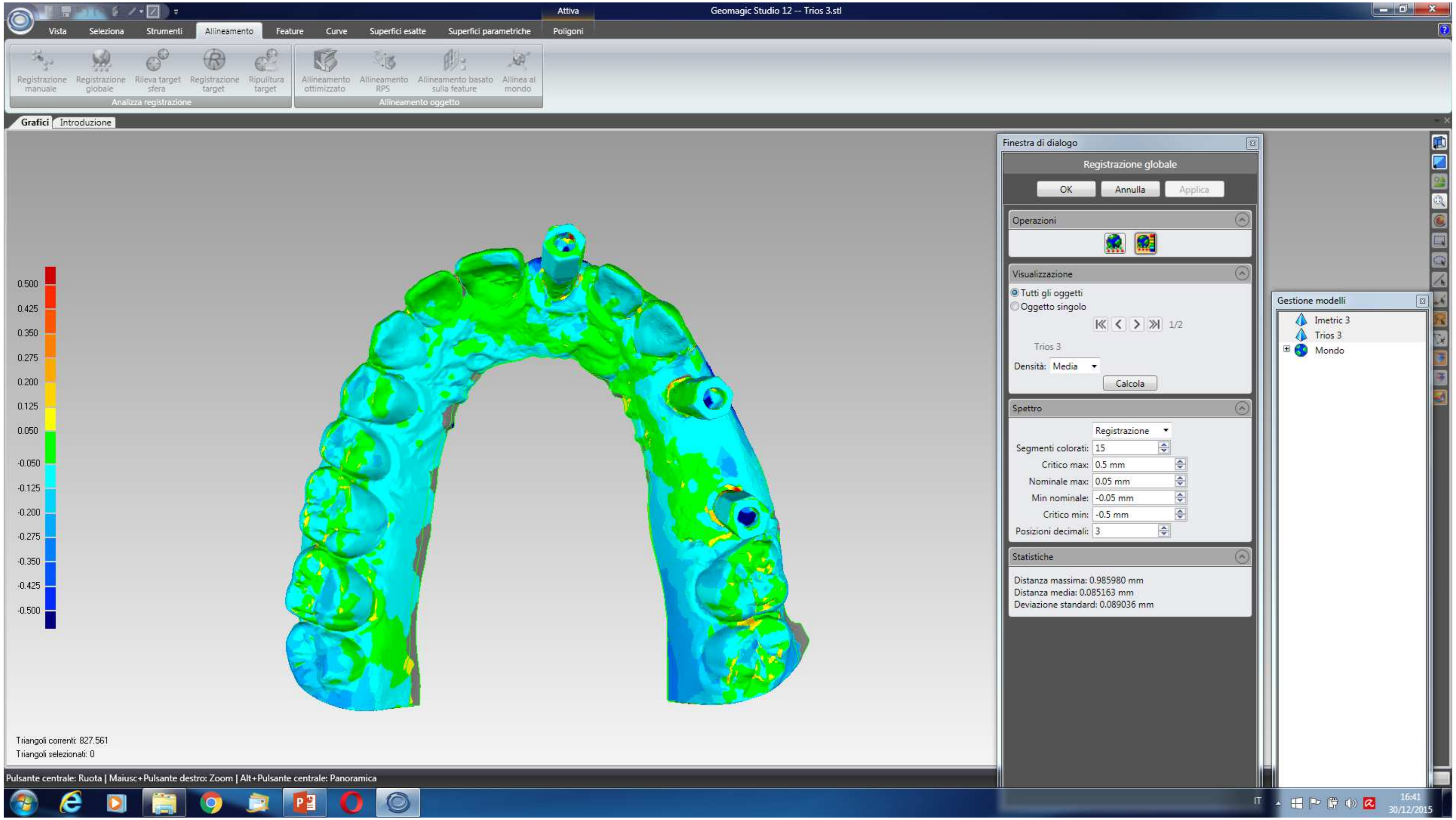

TRIOS 3 vs REF (IMETRIC)

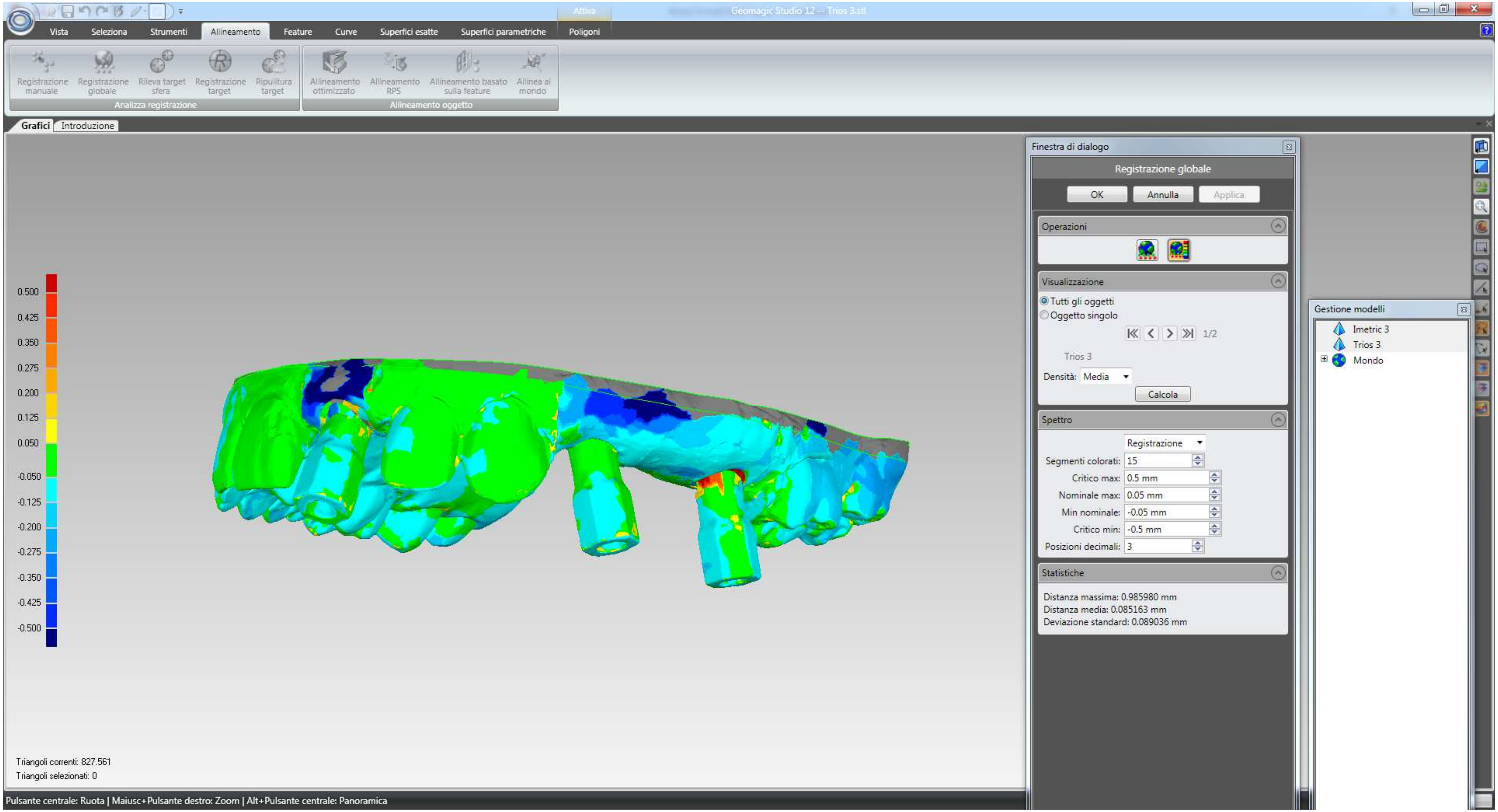

TRIOS 4 vs REF (IMETRIC)

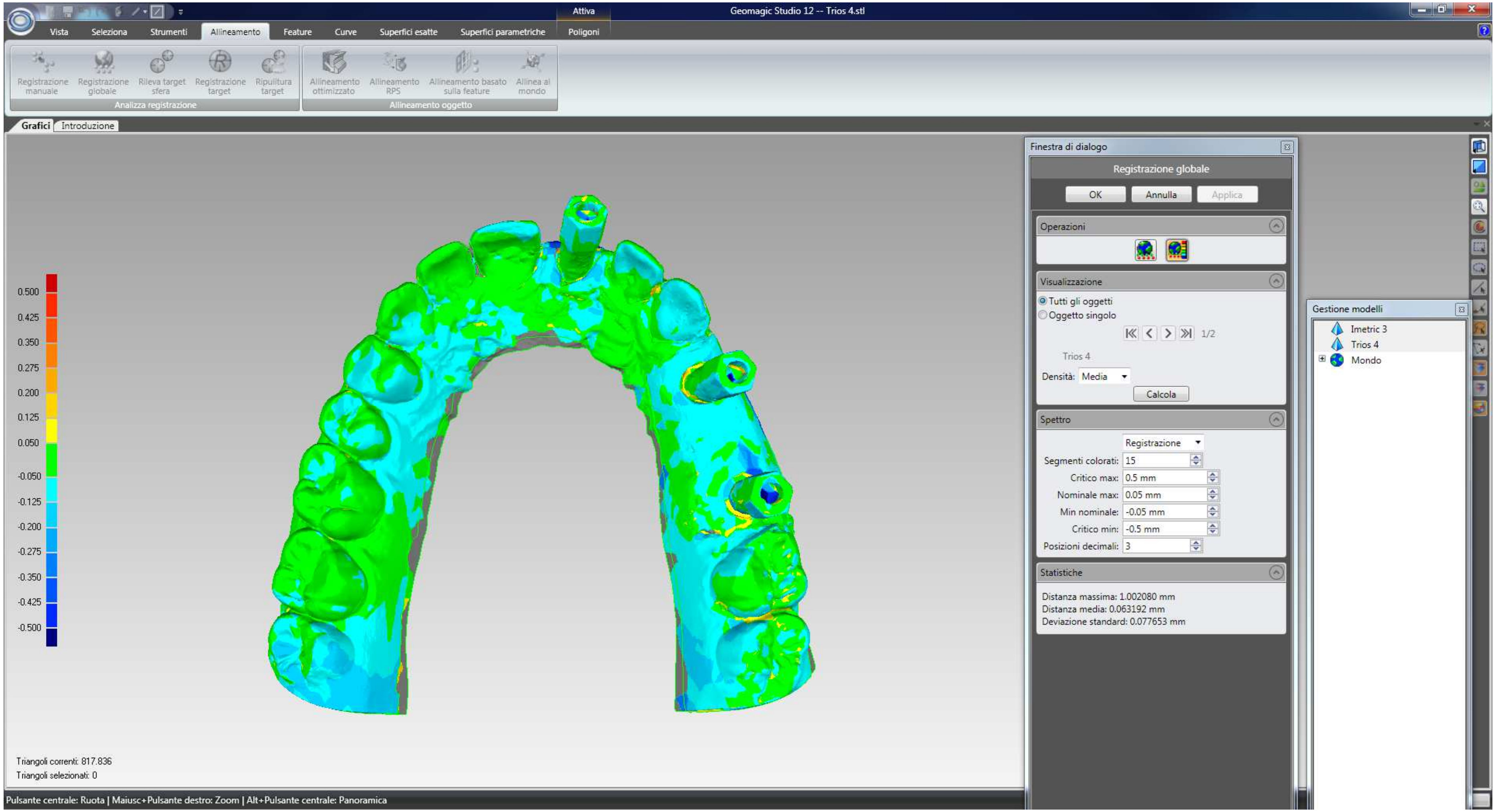

TRIOS 4 vs REF (IMETRIC)

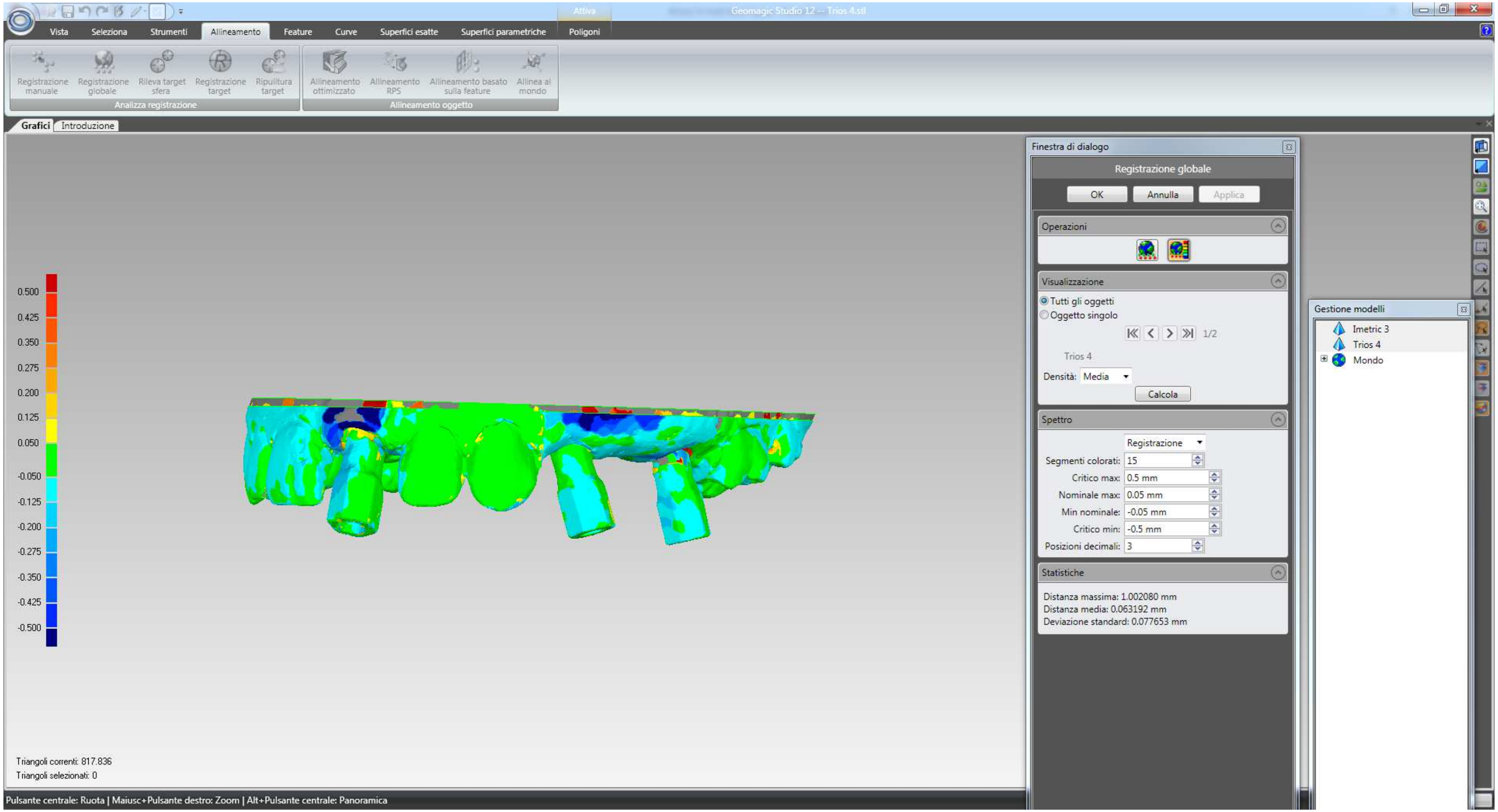

TRIOS 5 vs REF (IMETRIC)

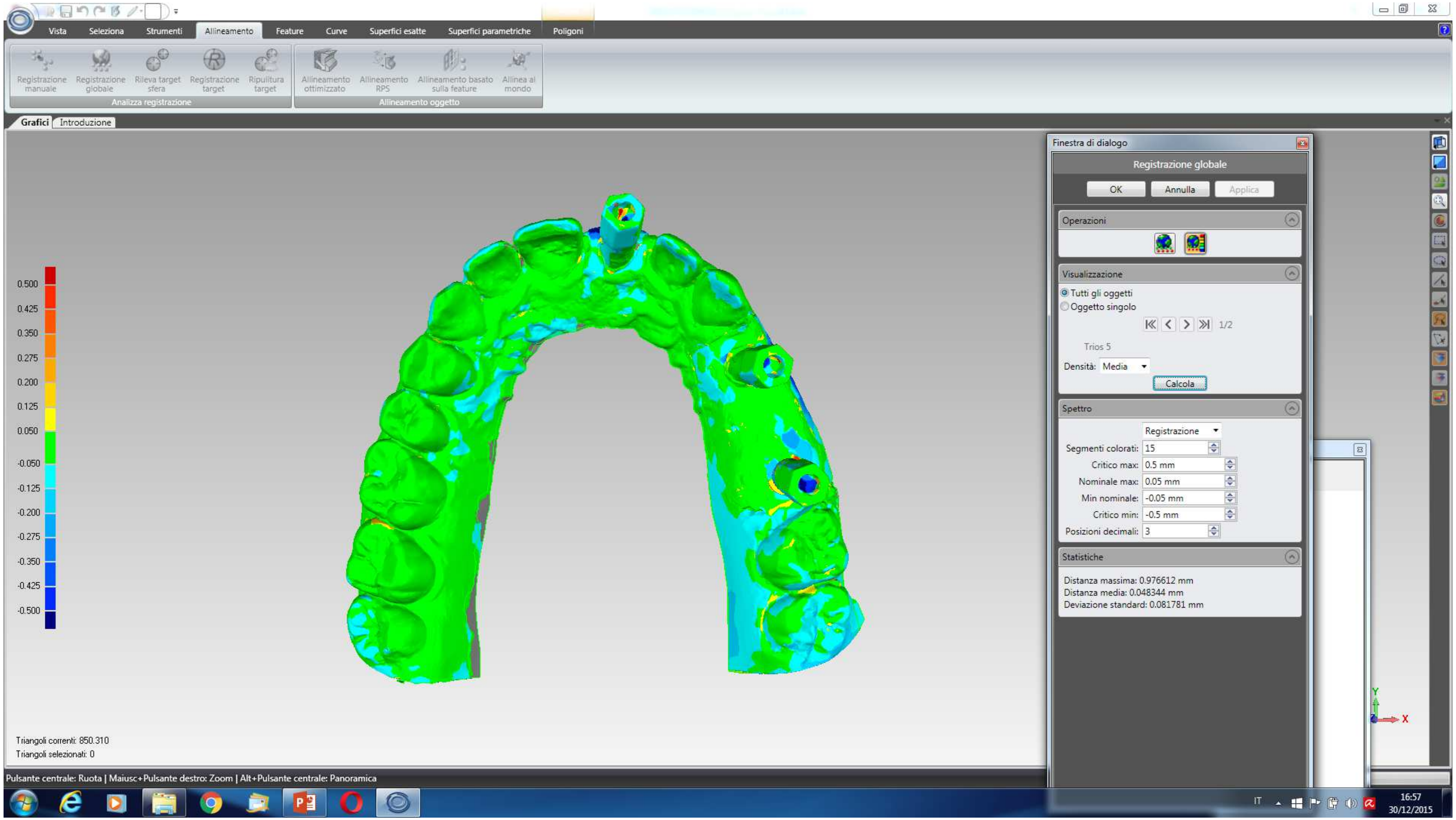

TRIOS 5 vs REF (IMETRIC)

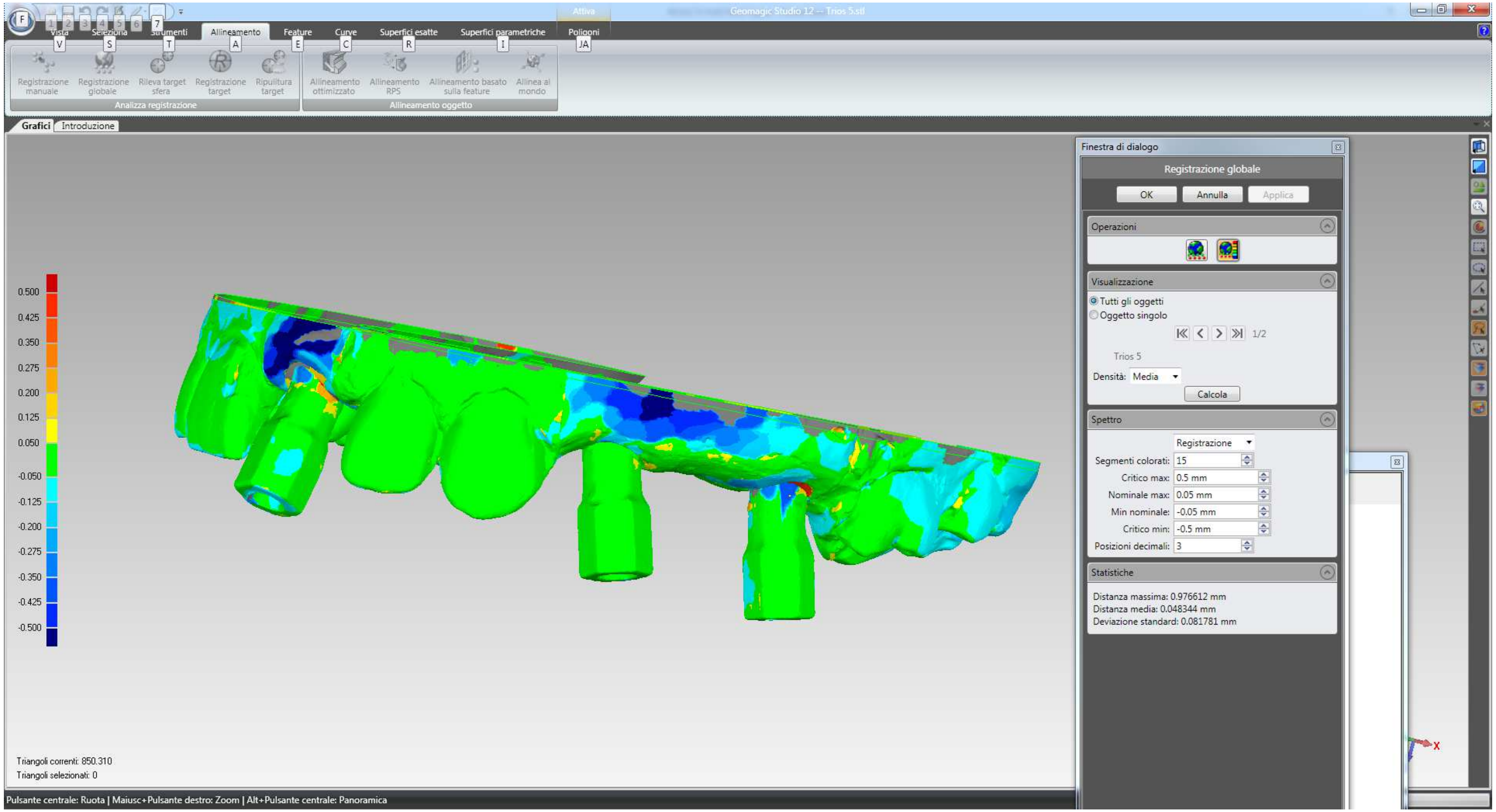

General trueness values, TRIOS in the partially edentulous model

	Mean distance	SD	Maximum distance
Trios 1	0.063	0.078	0.974
Trios 2	0.097	0.097	0.969
Trios 3	0.085	0.089	0.985
Trios 4	0.063	0.077	1.004
Trios 5	0.048	0.081	0.976

Overall Trios general TRUENESS: 0.071 (0.019)

ZFX 1 VS REF (IMETRIC)

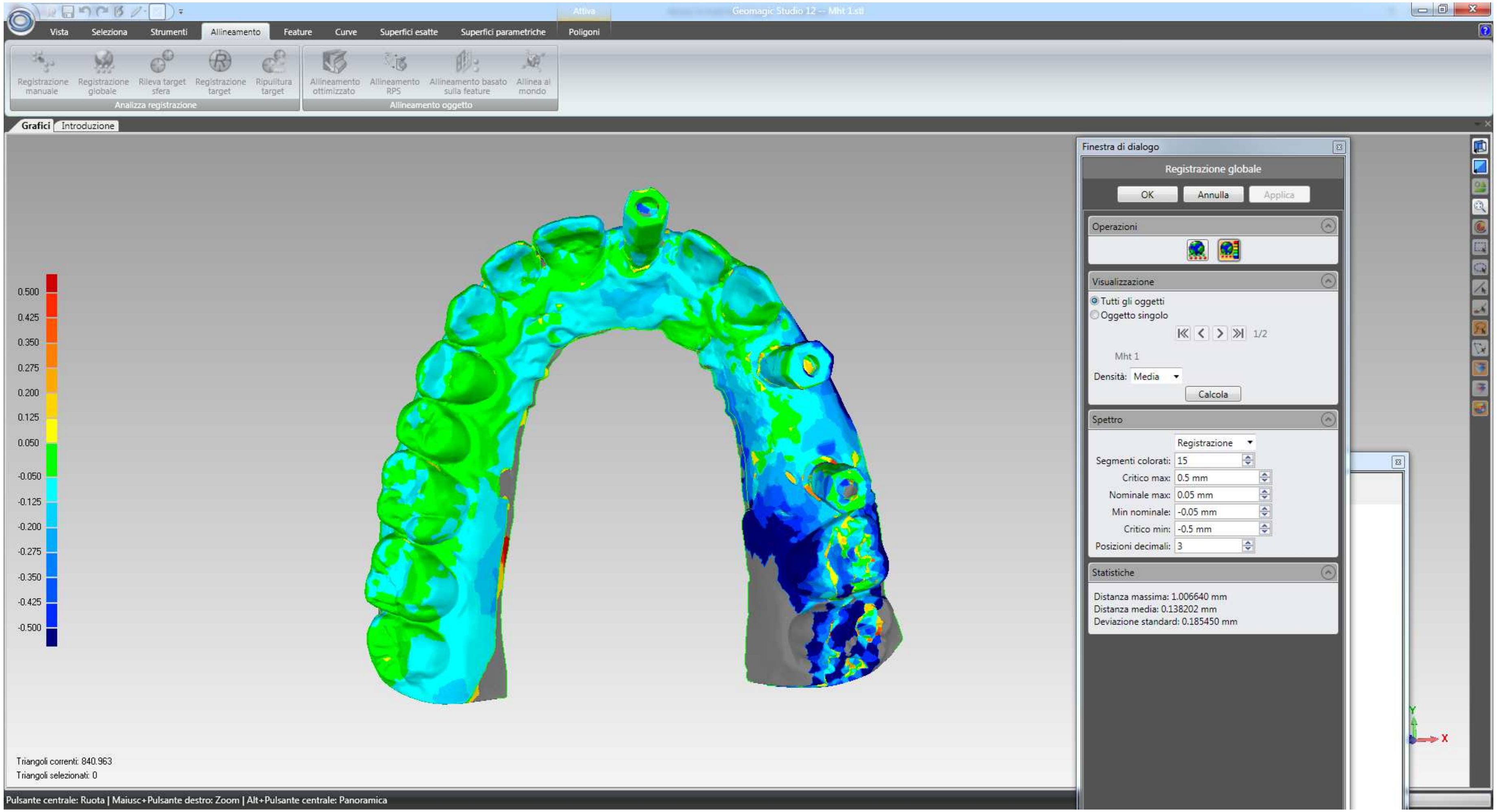

ZFX 1 VS REF (IMETRIC)

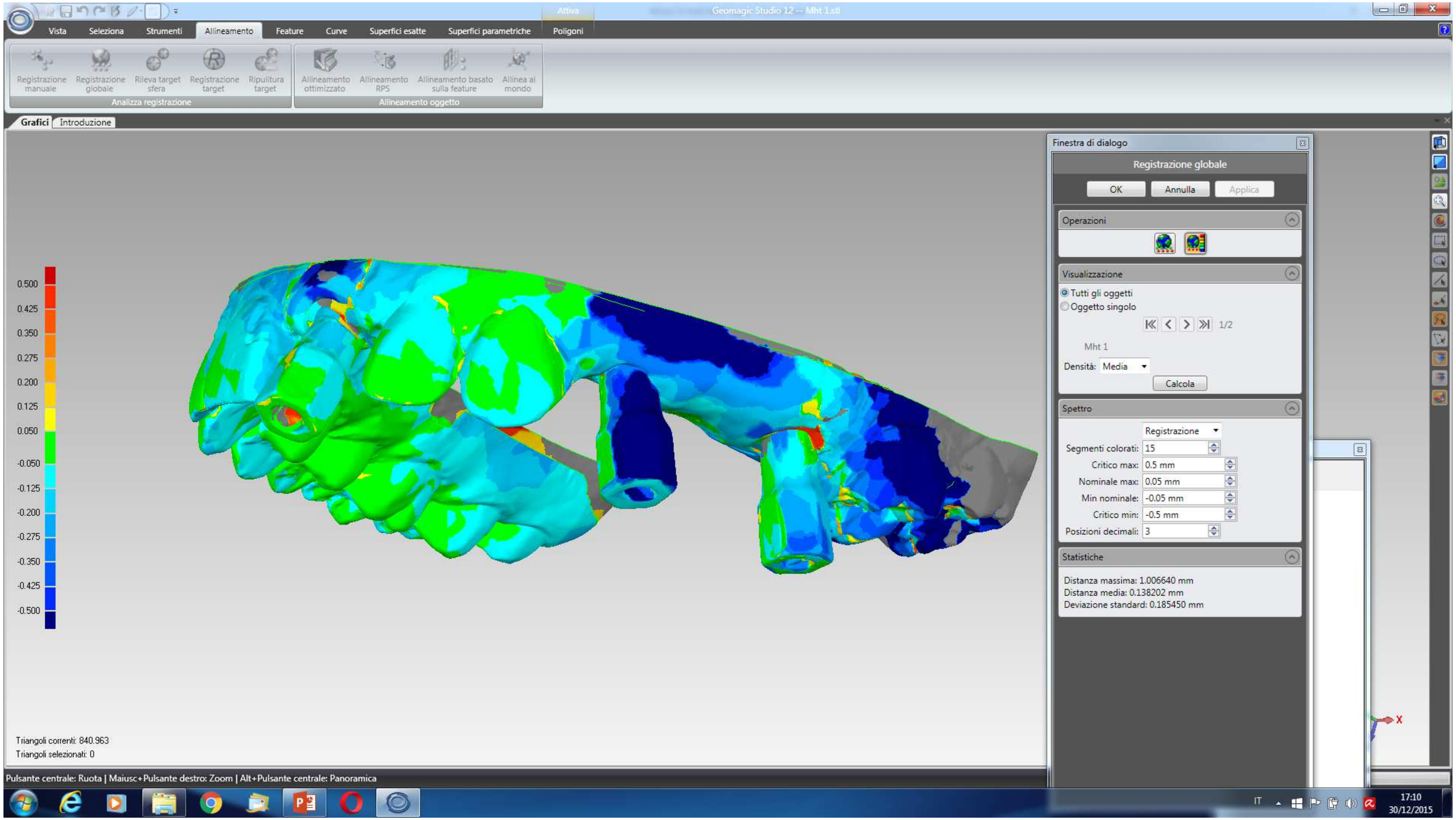

ZFX 2 VS REF (IMETRIC)

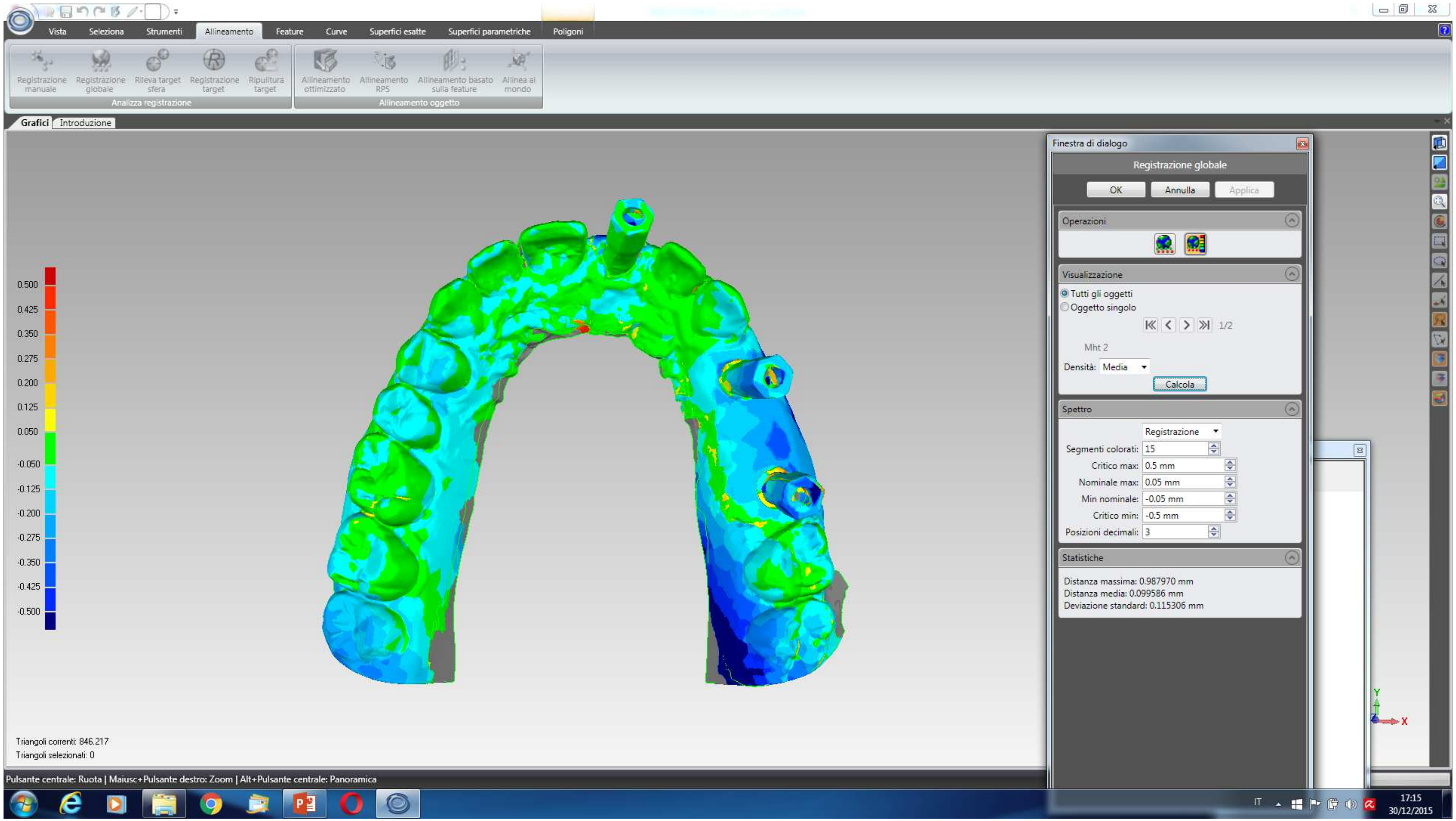

ZFX 2 VS REF (IMETRIC)

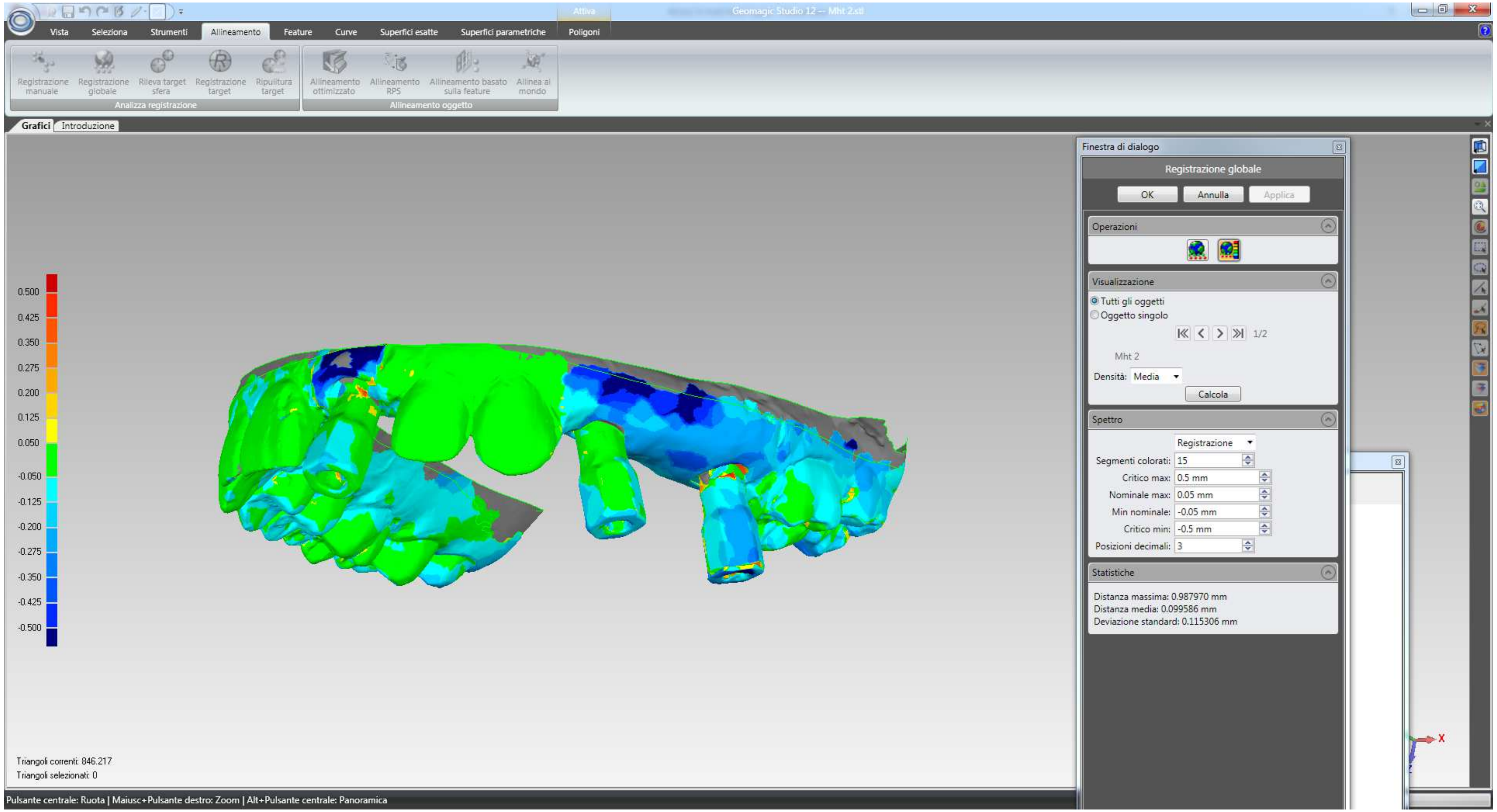

ZFX 3 VS REF (IMETRIC)

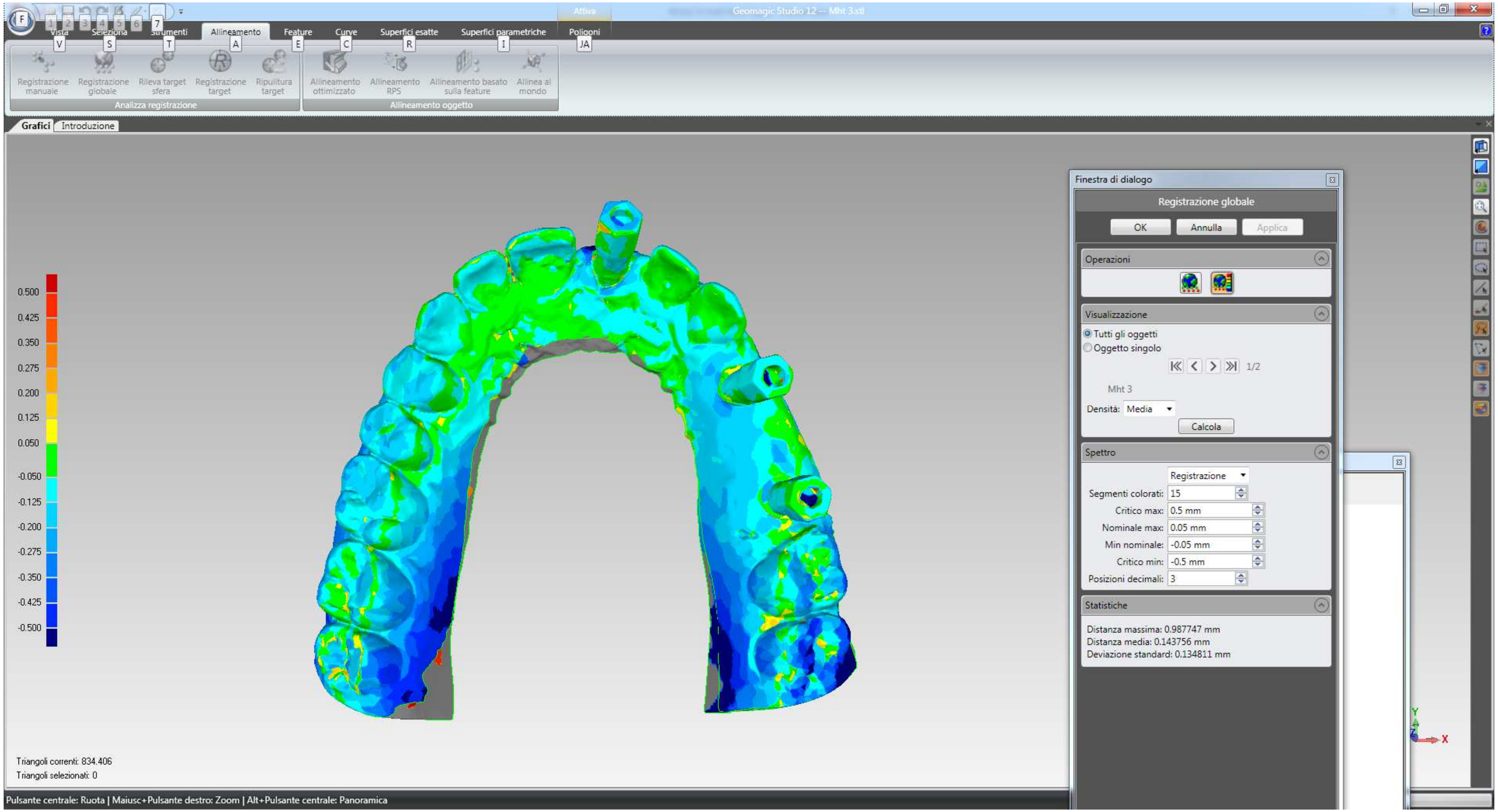

ZFX 3 VS REF (IMETRIC)

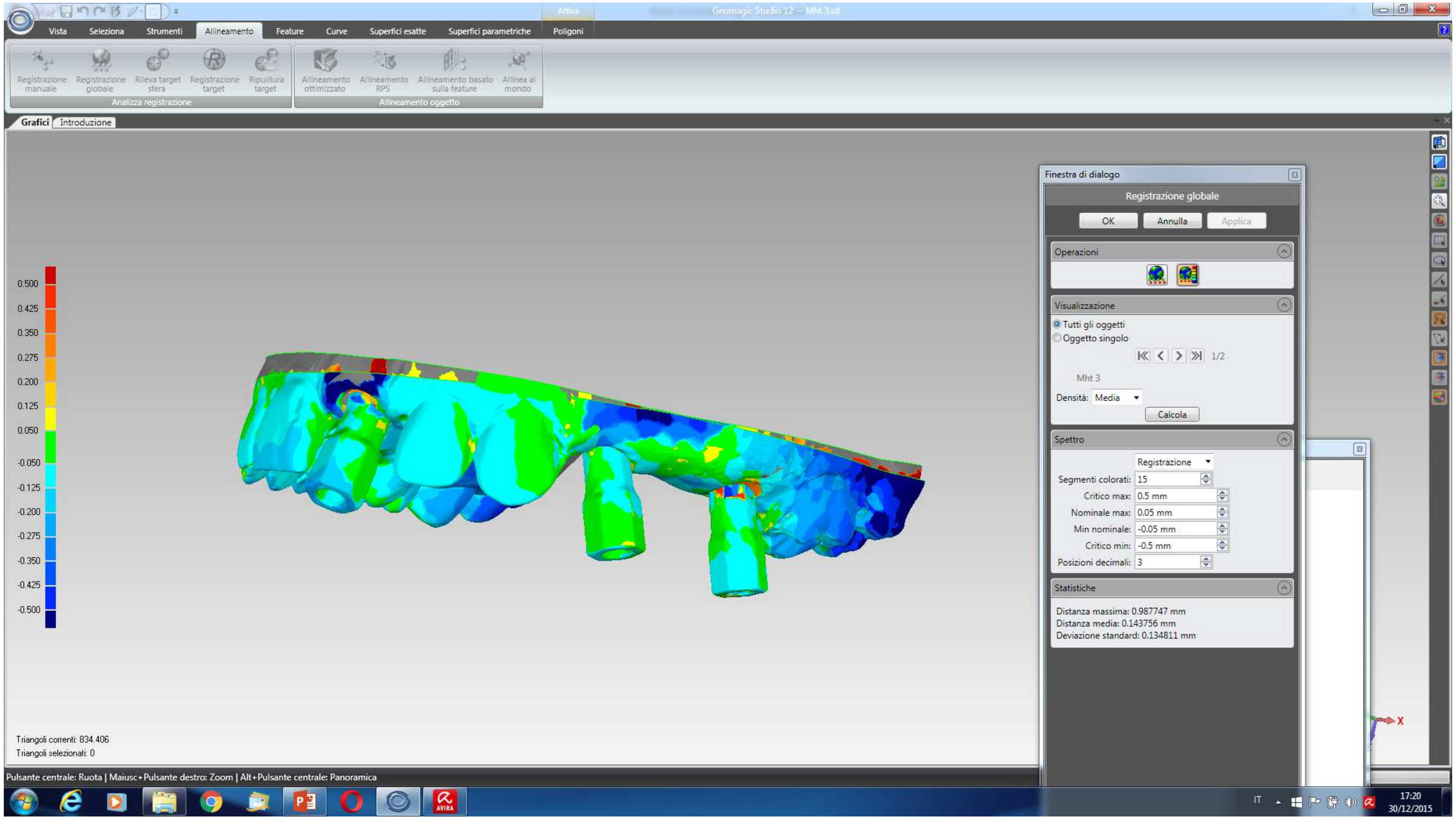

ZFX 4 VS REF (IMETRIC)

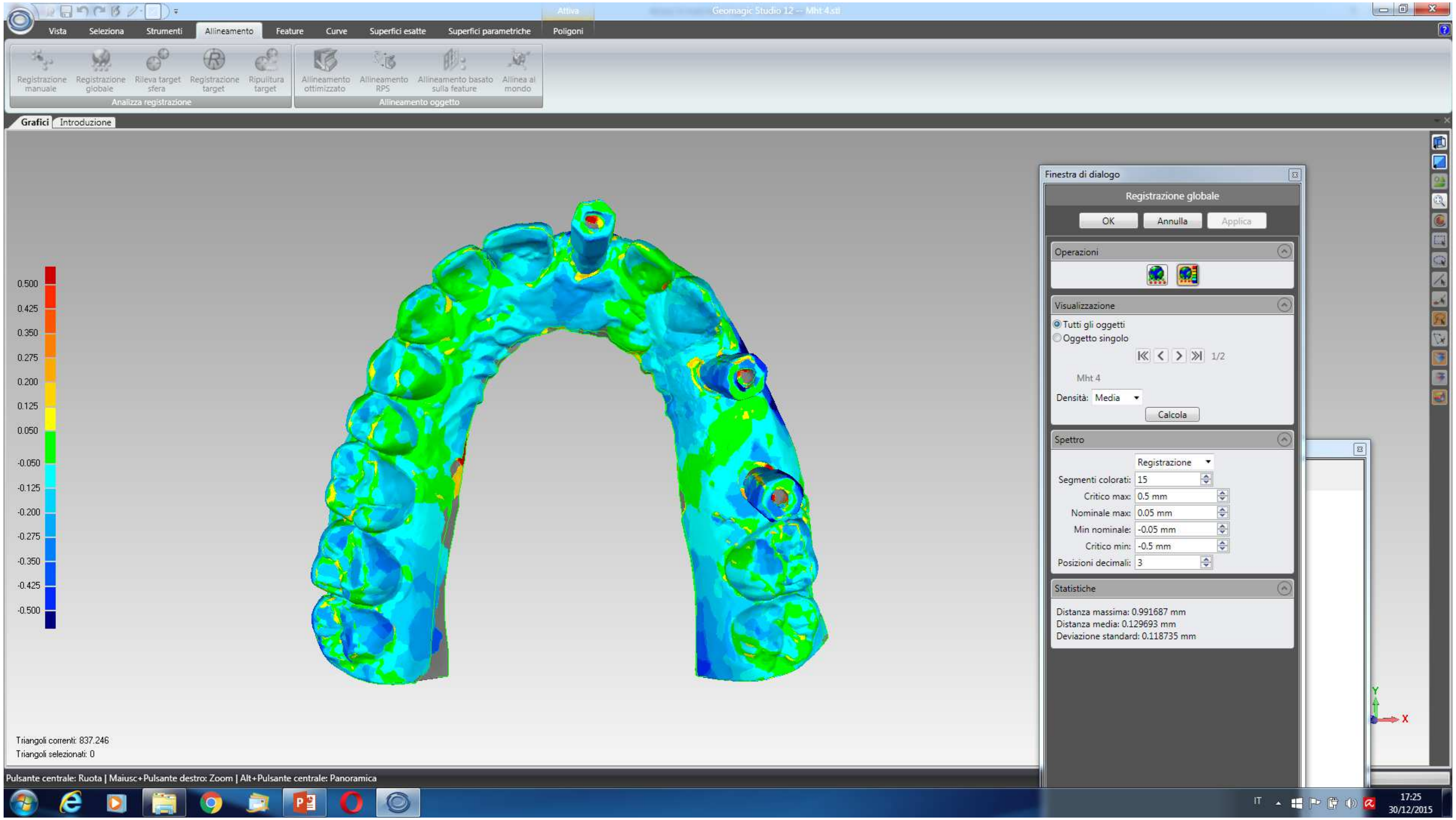

ZFX 4 VS REF (IMETRIC)

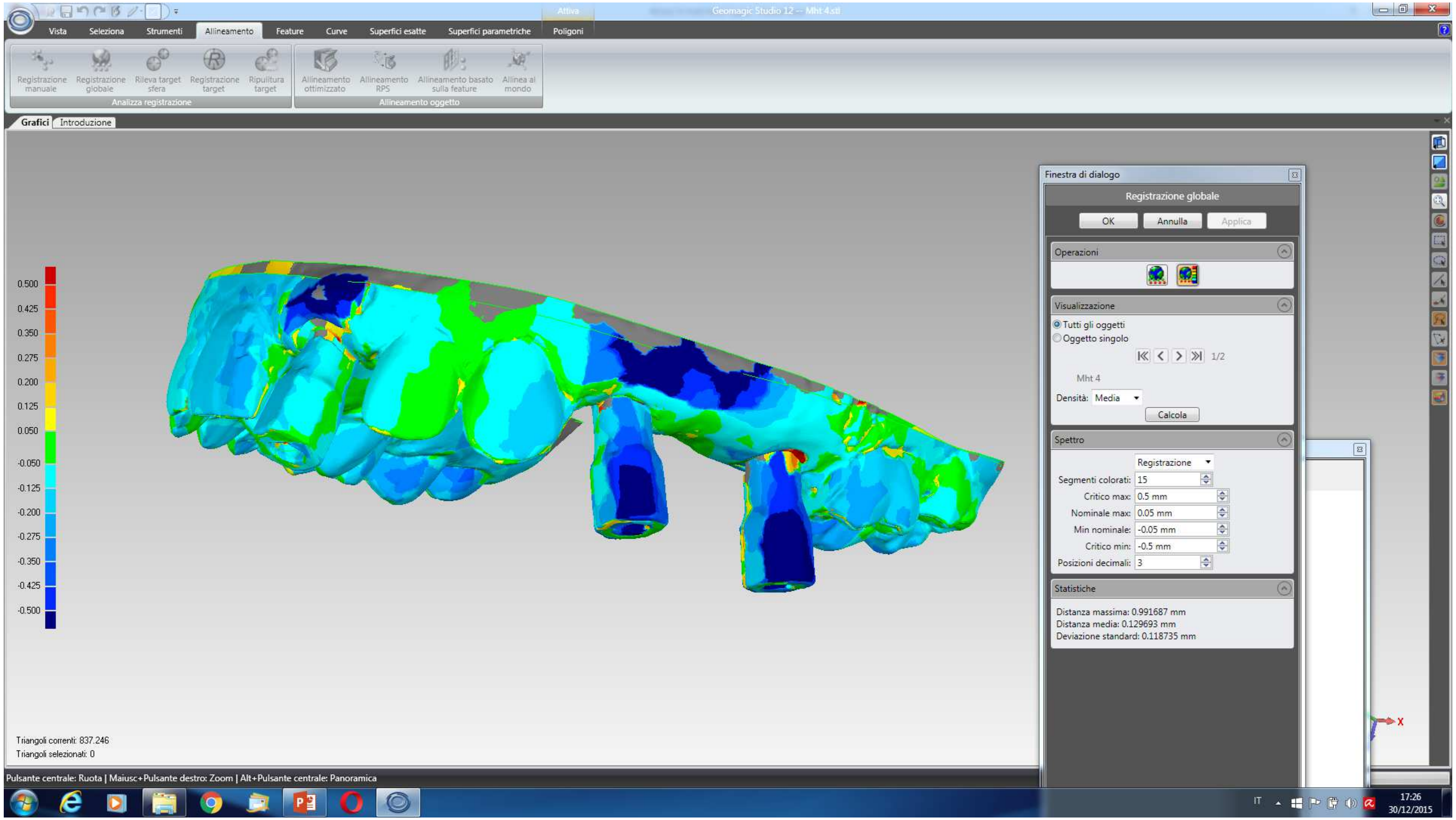

ZFX 5 VS REF (IMETRIC)

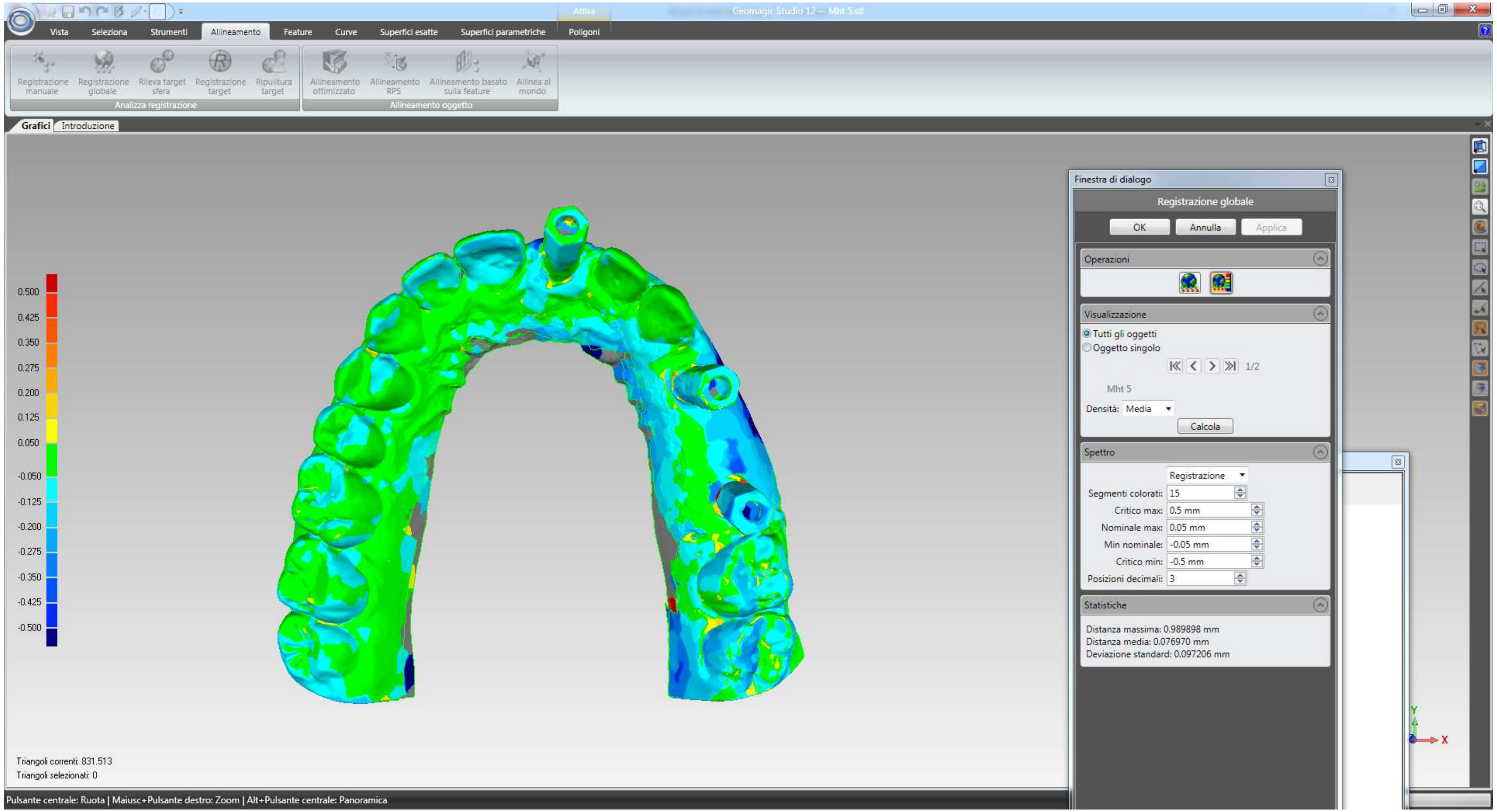

ZFX 5 VS REF (IMETRIC)

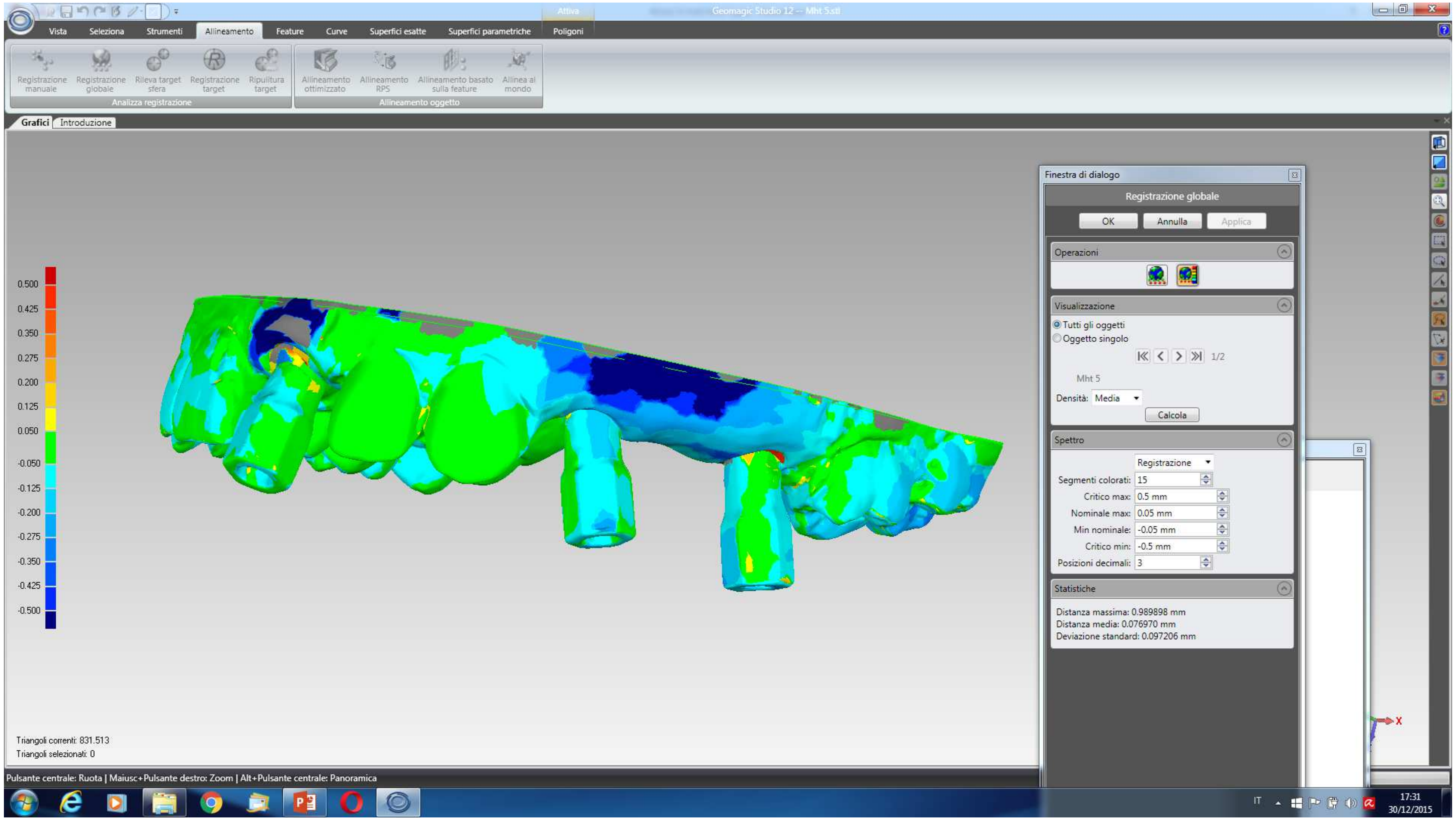

Overall general trueness with Zfx in the partially edentulous model

	Mean distance	SD	Maximum distance
Zfx 1	0.138	0.185	1.006
Zfx 2	0.099	0.115	0.987
Zfx 3	0.143	0.134	0.987
Zfx 4	0.129	0.118	0.991
Zfx 5	0.076	0.097	0.989

Overall Mht general trueness: 0.117 (0.028)

Care 1 vs REF (IMETRIC)

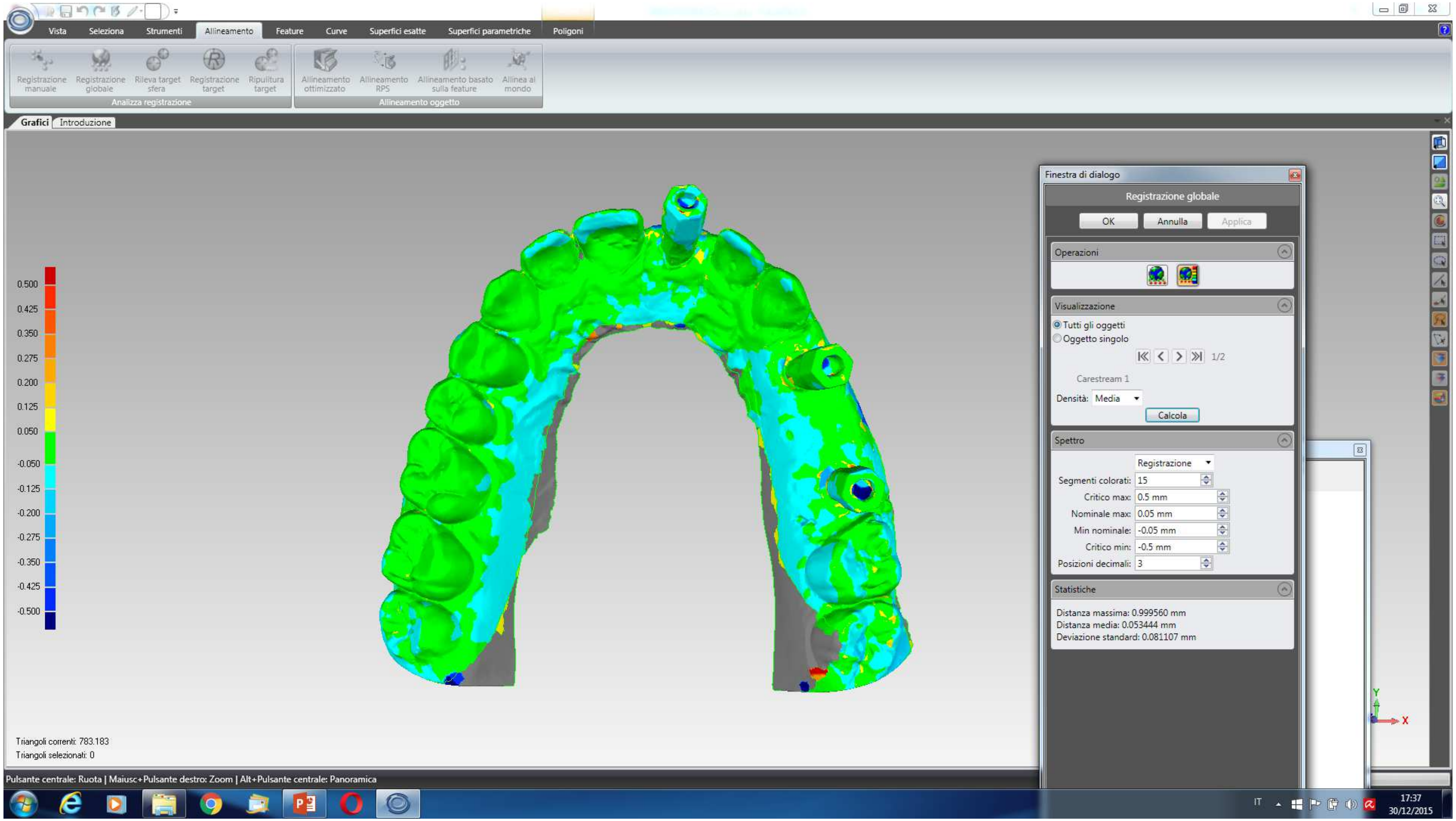

Care 1 vs REF (IMETRIC)

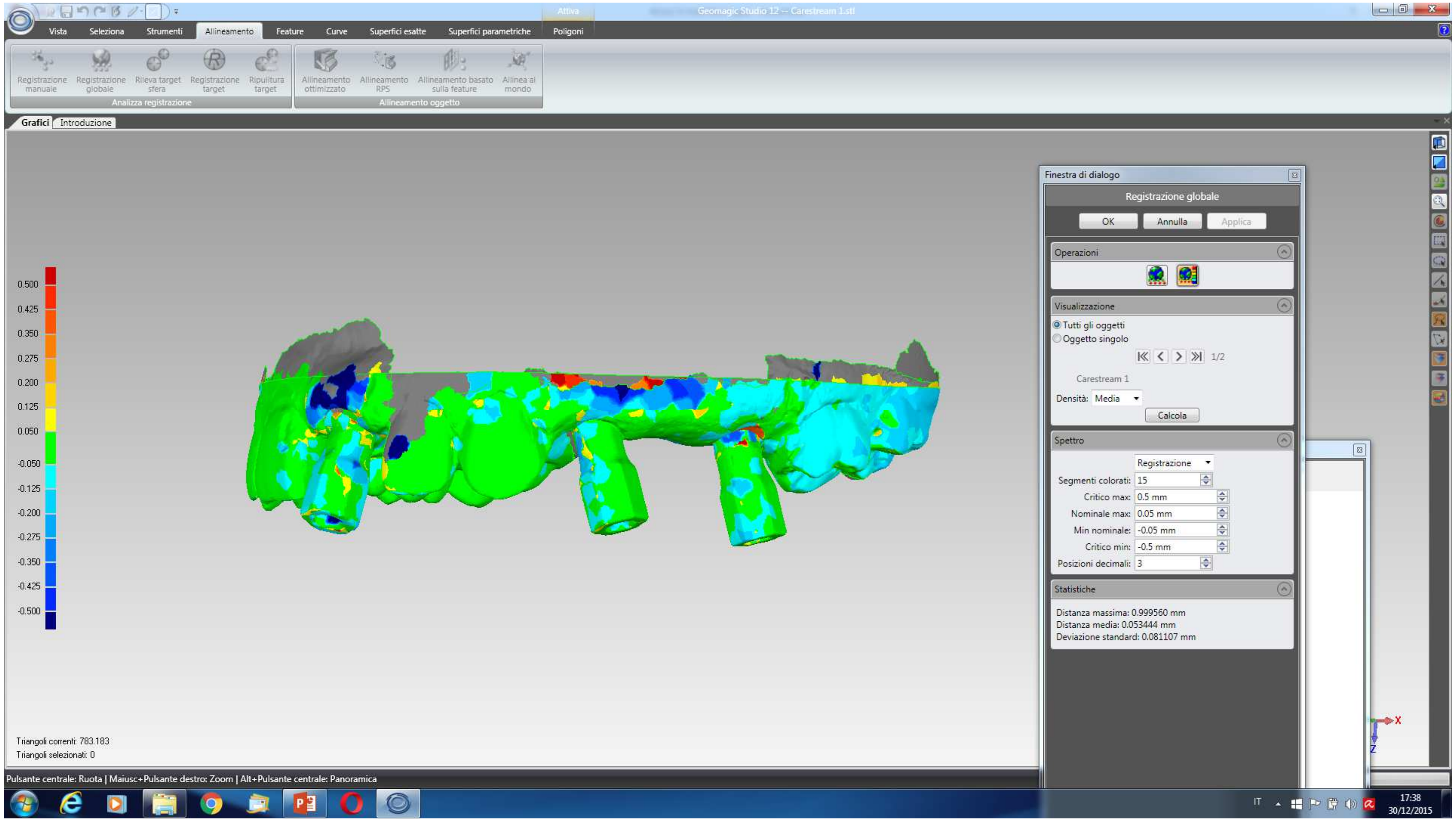

Care 2 vs REF (IMETRIC)

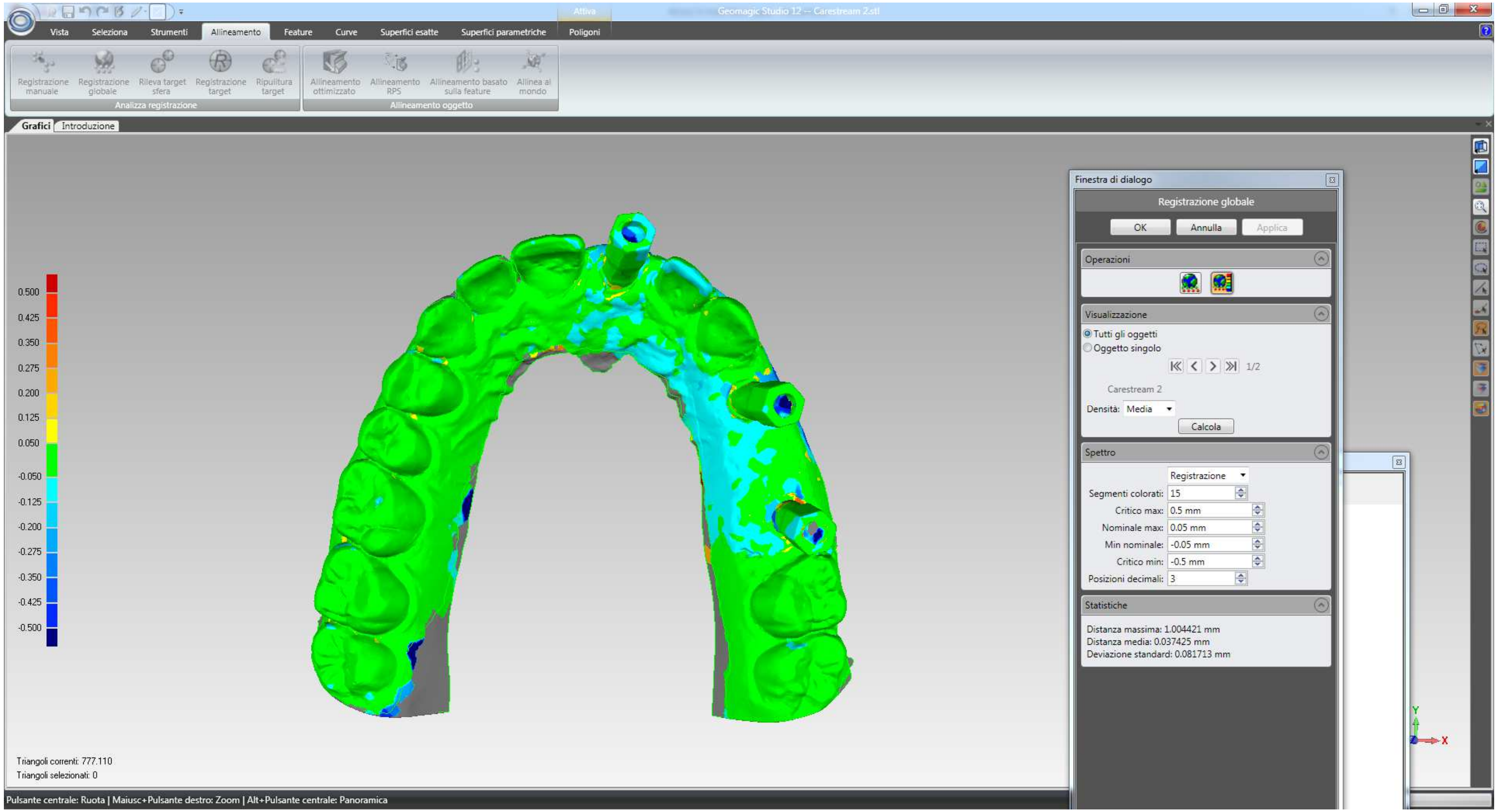

Care 2 vs REF (IMETRIC)

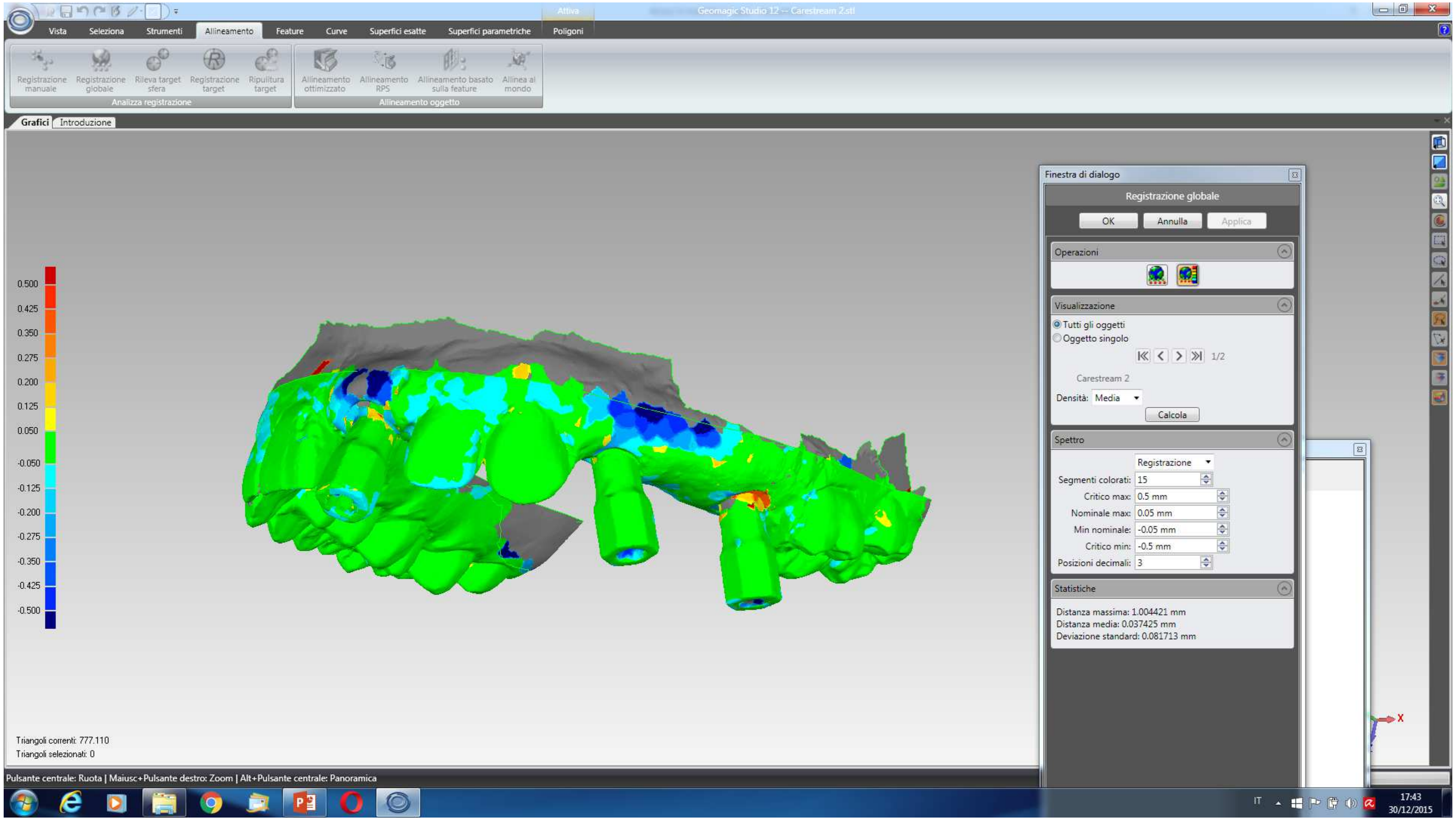

Care 3 vs REF (IMETRIC)

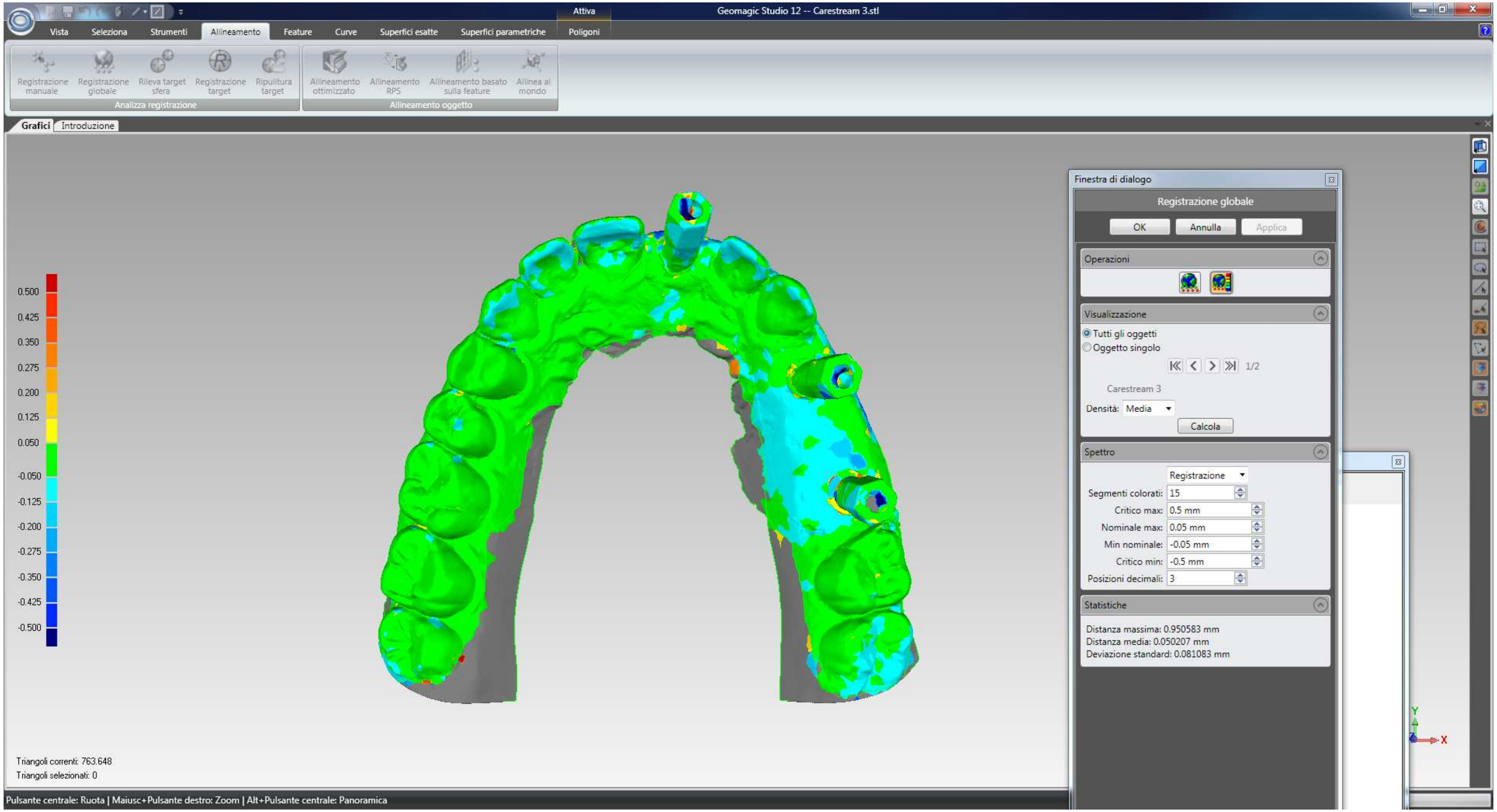

Care 3 vs REF (IMETRIC)

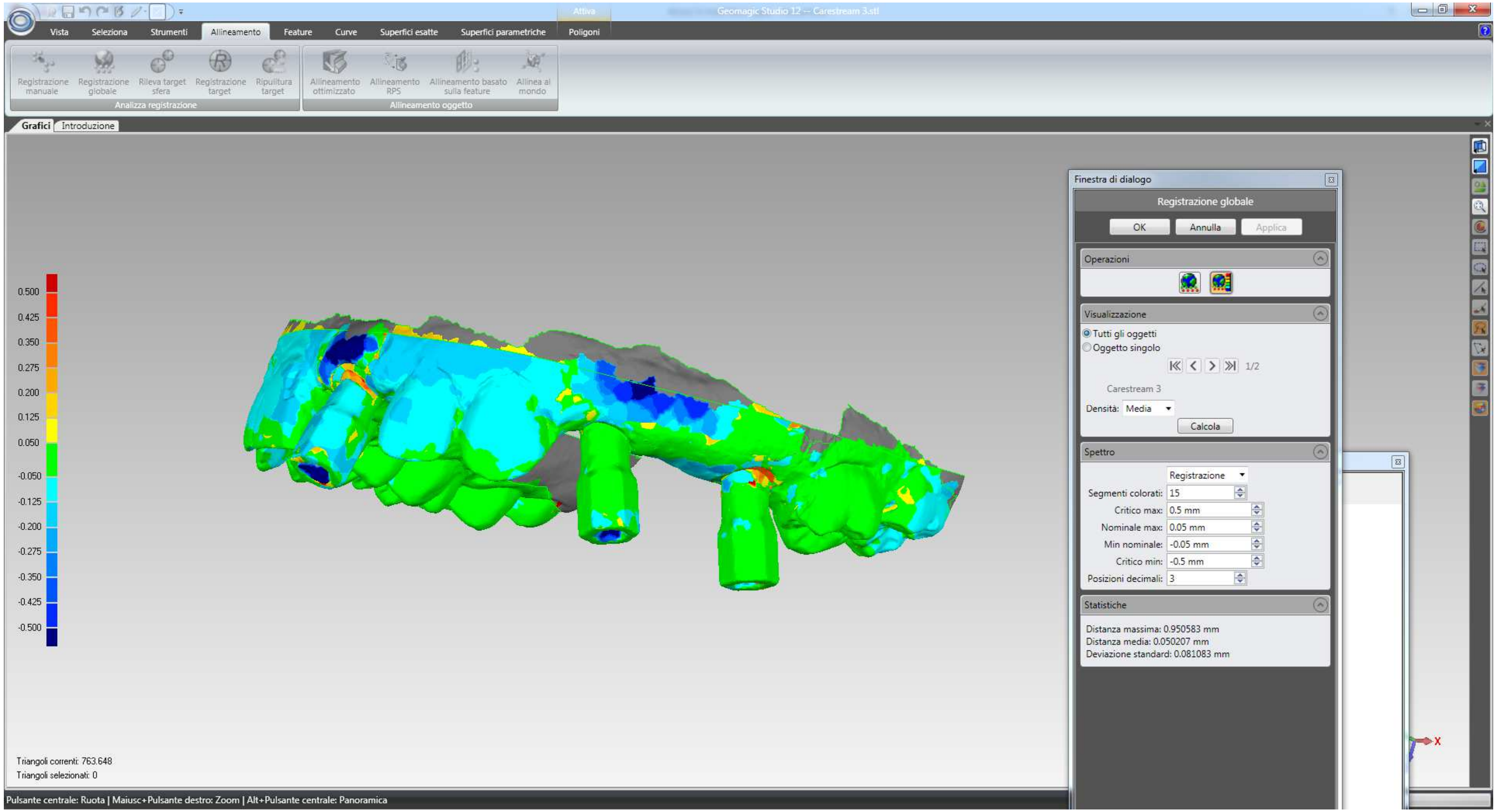

Care 4 vs REF (IMETRIC)

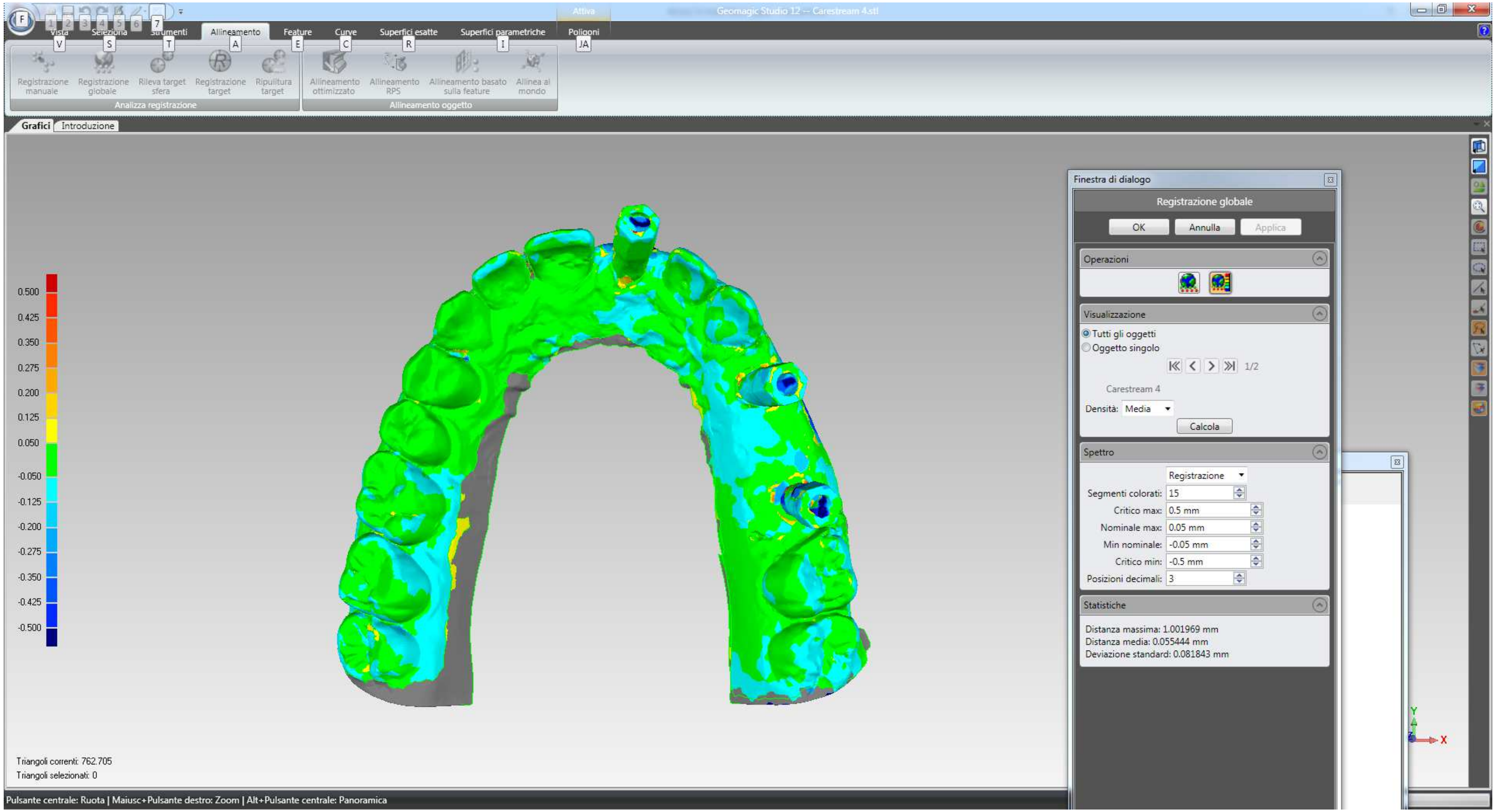

Care 4 vs REF (IMETRIC)

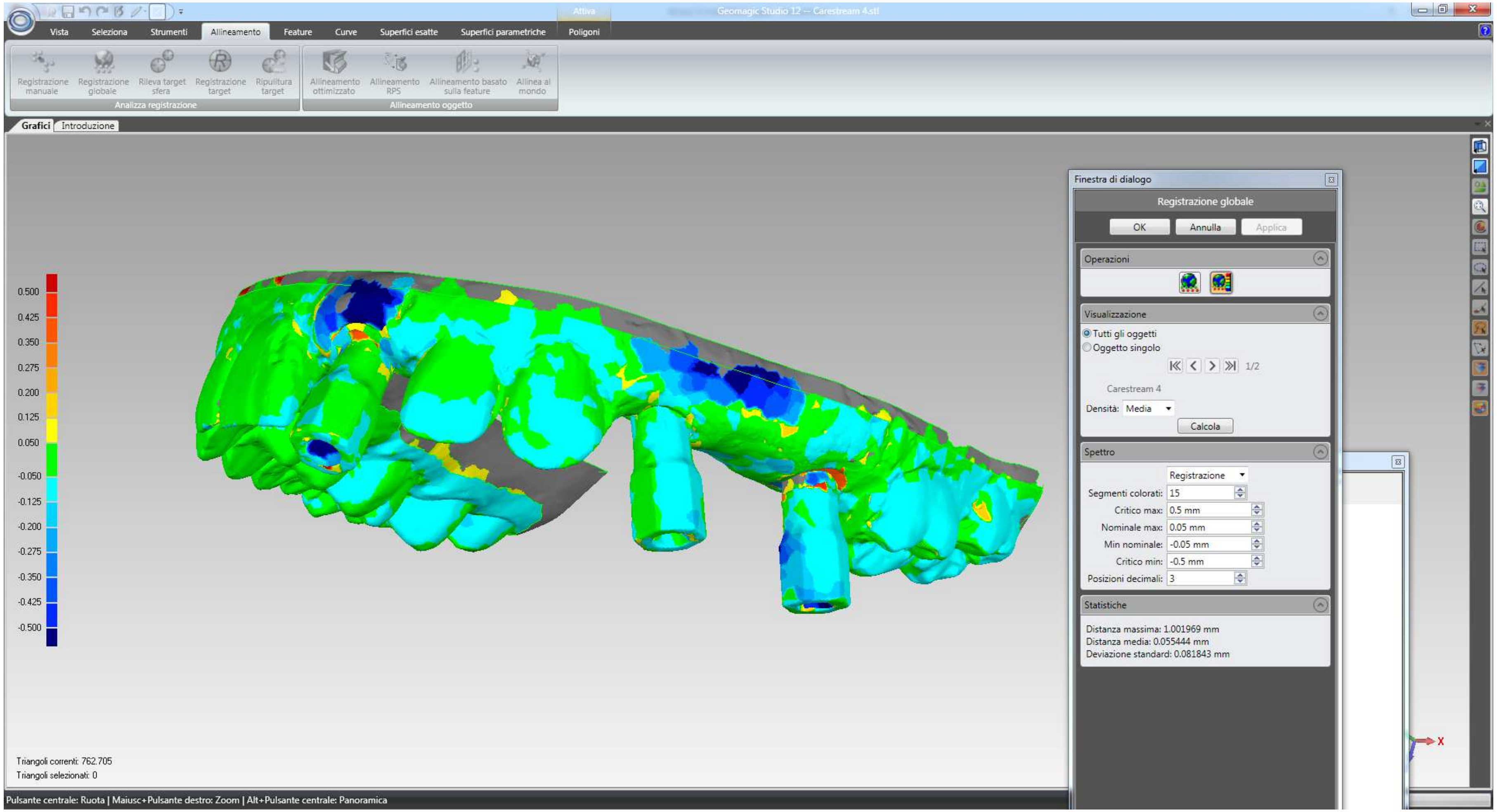

Care 5 vs REF (IMETRIC)

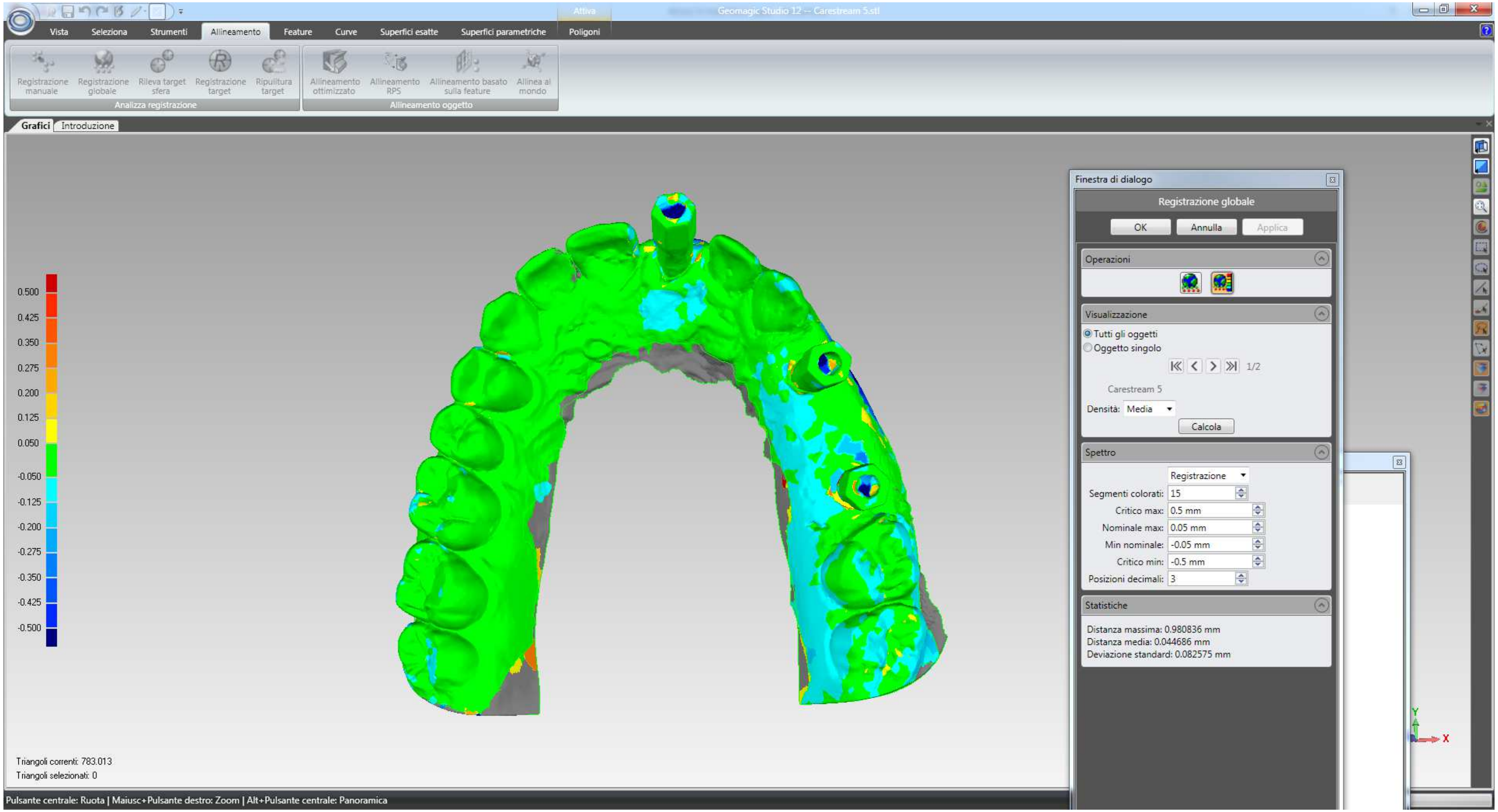

Care 5 vs REF (IMETRIC)

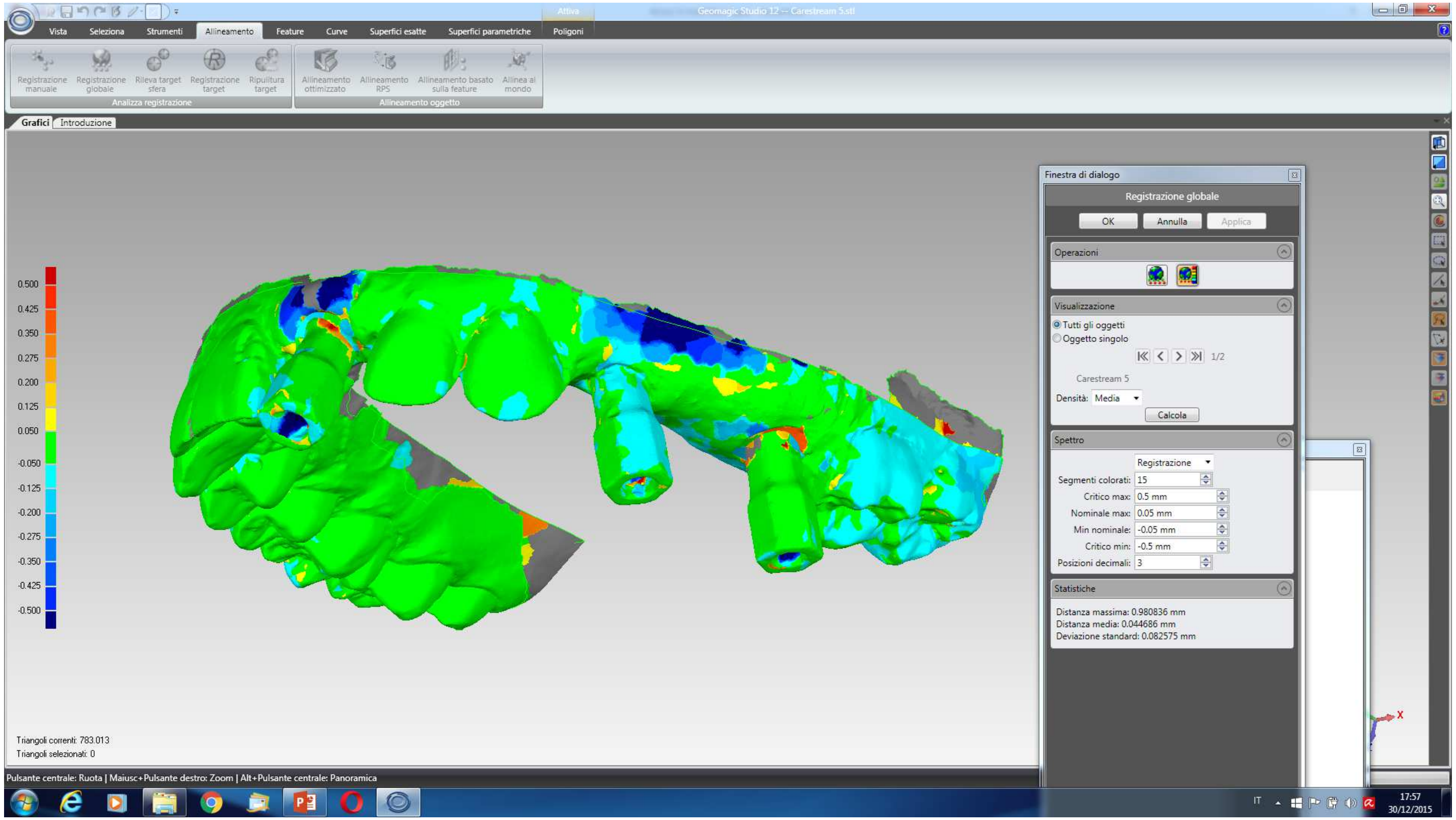

General trueness of Carestream in the partially edentulous model

	Mean distance	SD	Maximum distance
Carestream 1	0.053	0.081	0.999
Carestream 2	0.037	0.081	1.004
Carestream 3	0.050	0.081	0.950
Carestream 4	0.055	0.081	1.001
Carestream 5	0.044	0.082	0.980

Overall Care general trueness: 0.047 (0.007)

Plan 1 vs REF (IMETRIC)

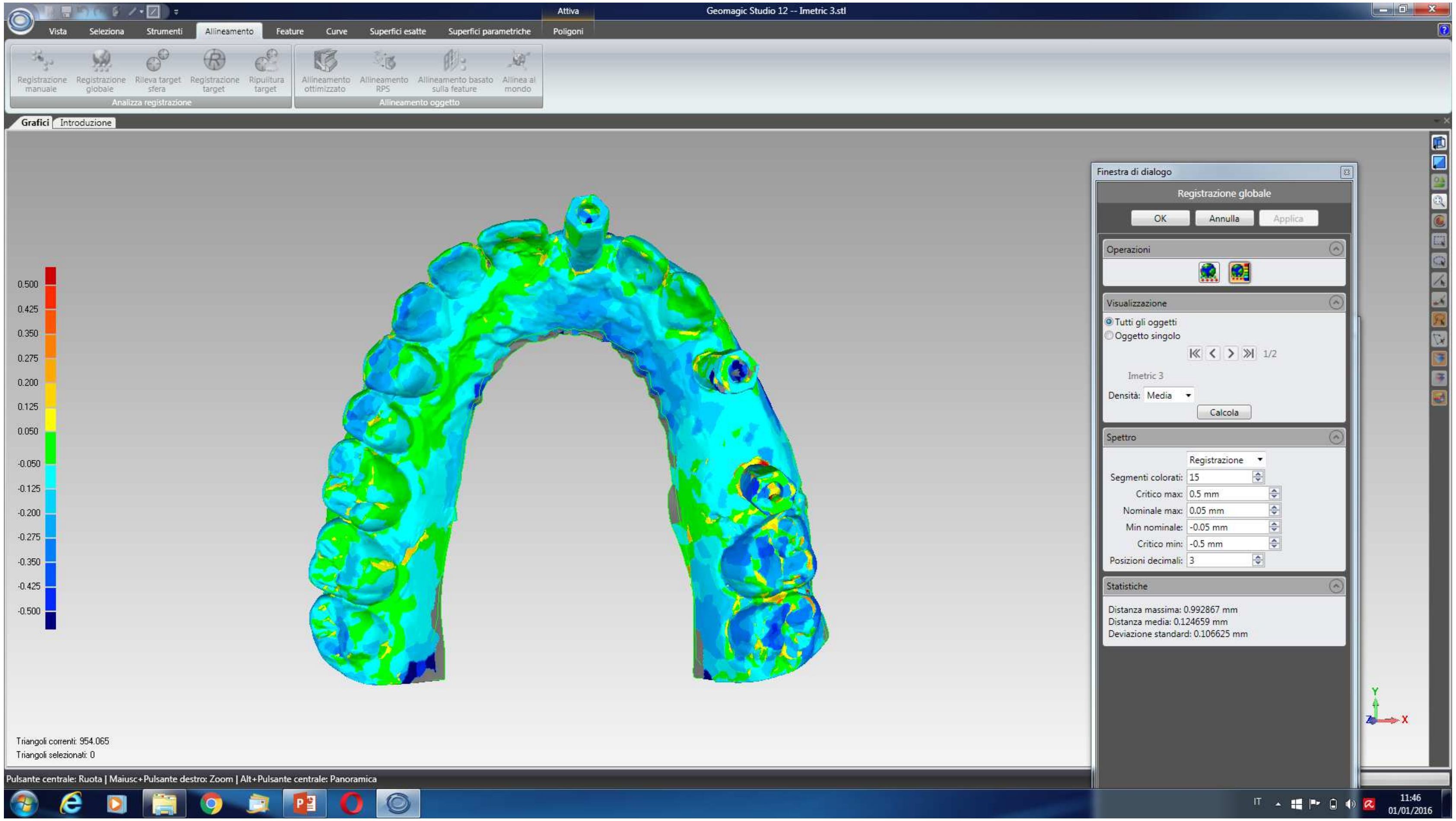

Plan 1 vs REF (IMETRIC)

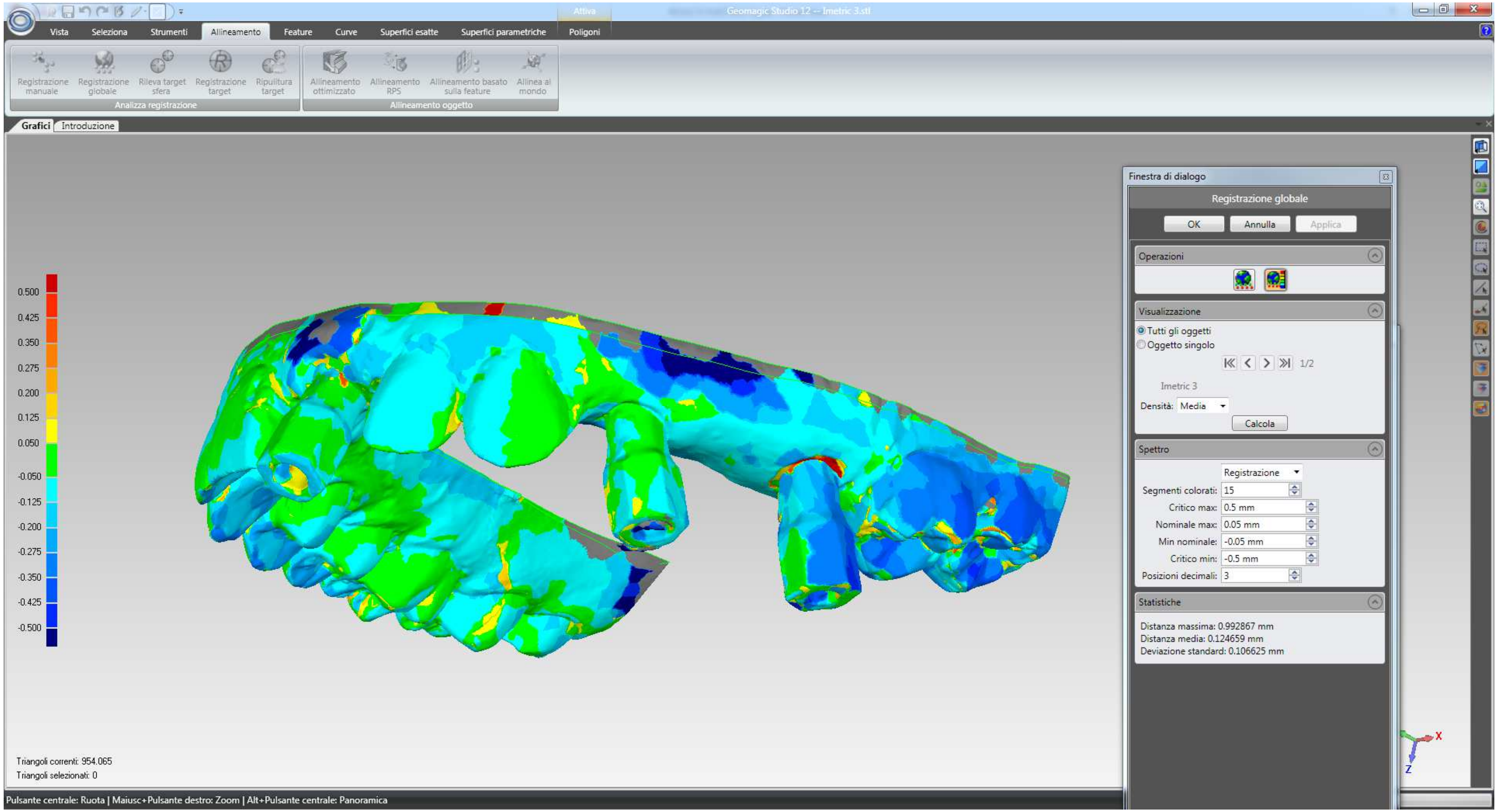

Plan 2 vs REF (IMETRIC)

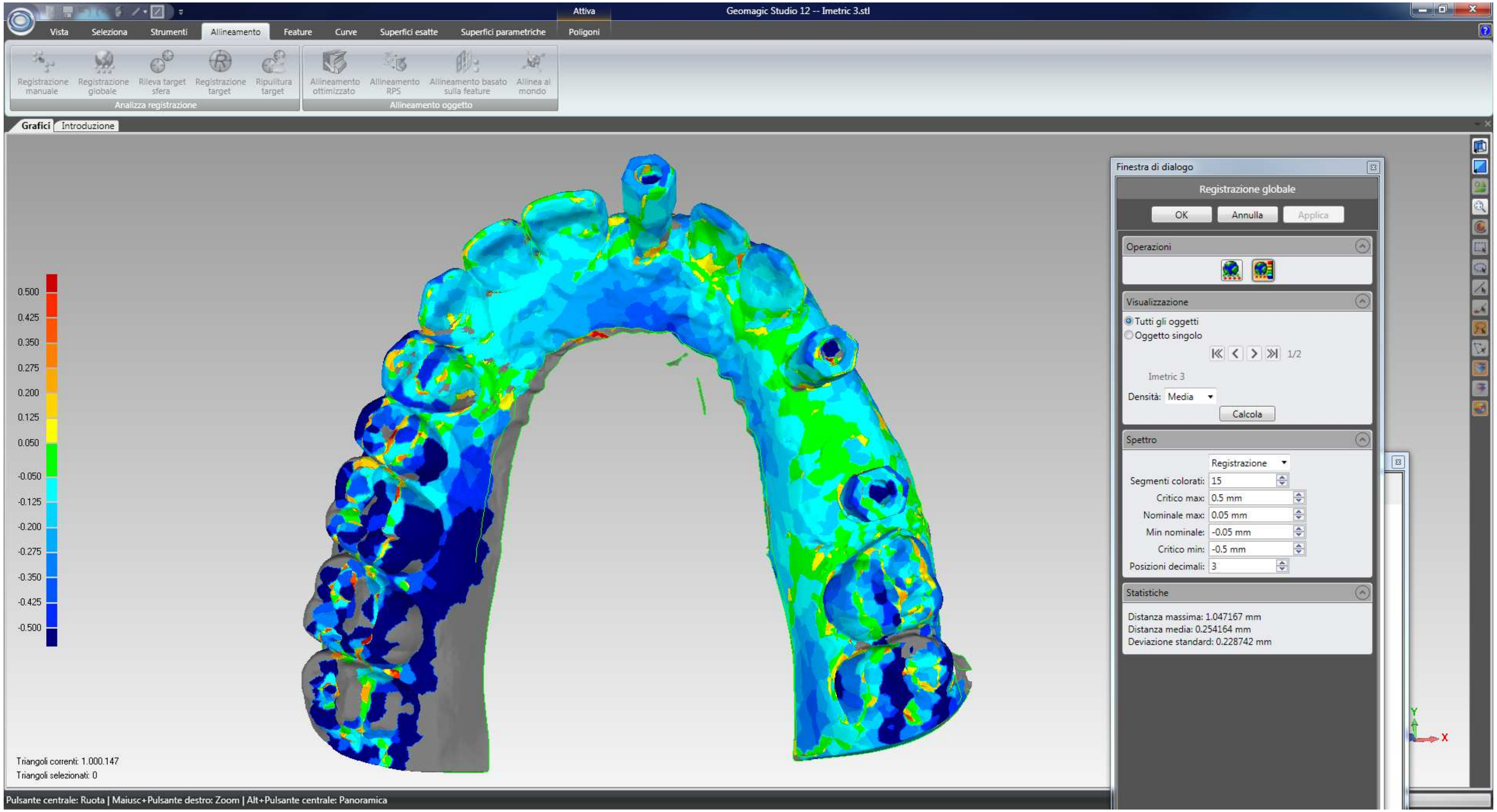

Plan 2 vs REF (IMETRIC)

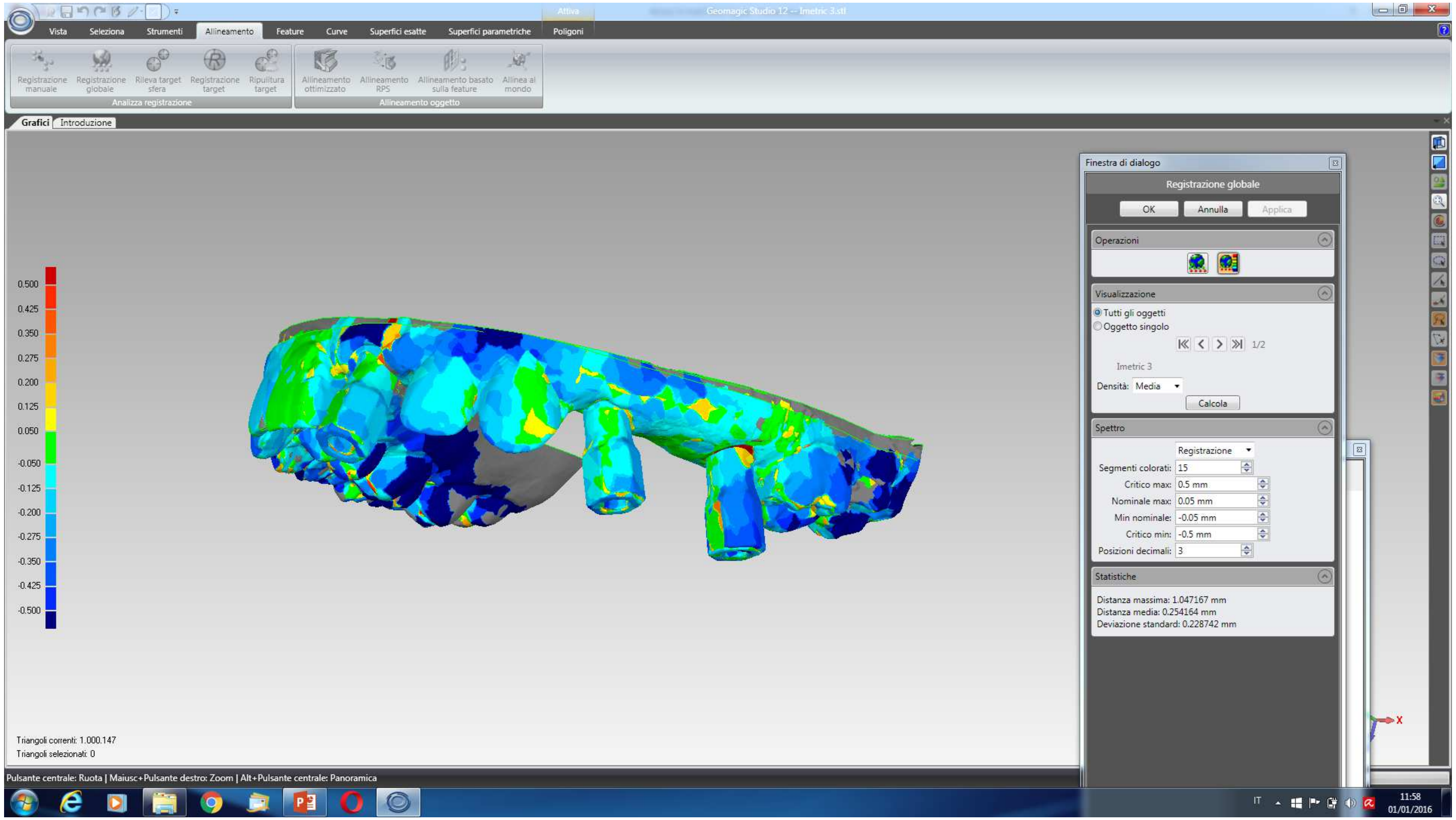

Plan 3 vs REF (IMETRIC)

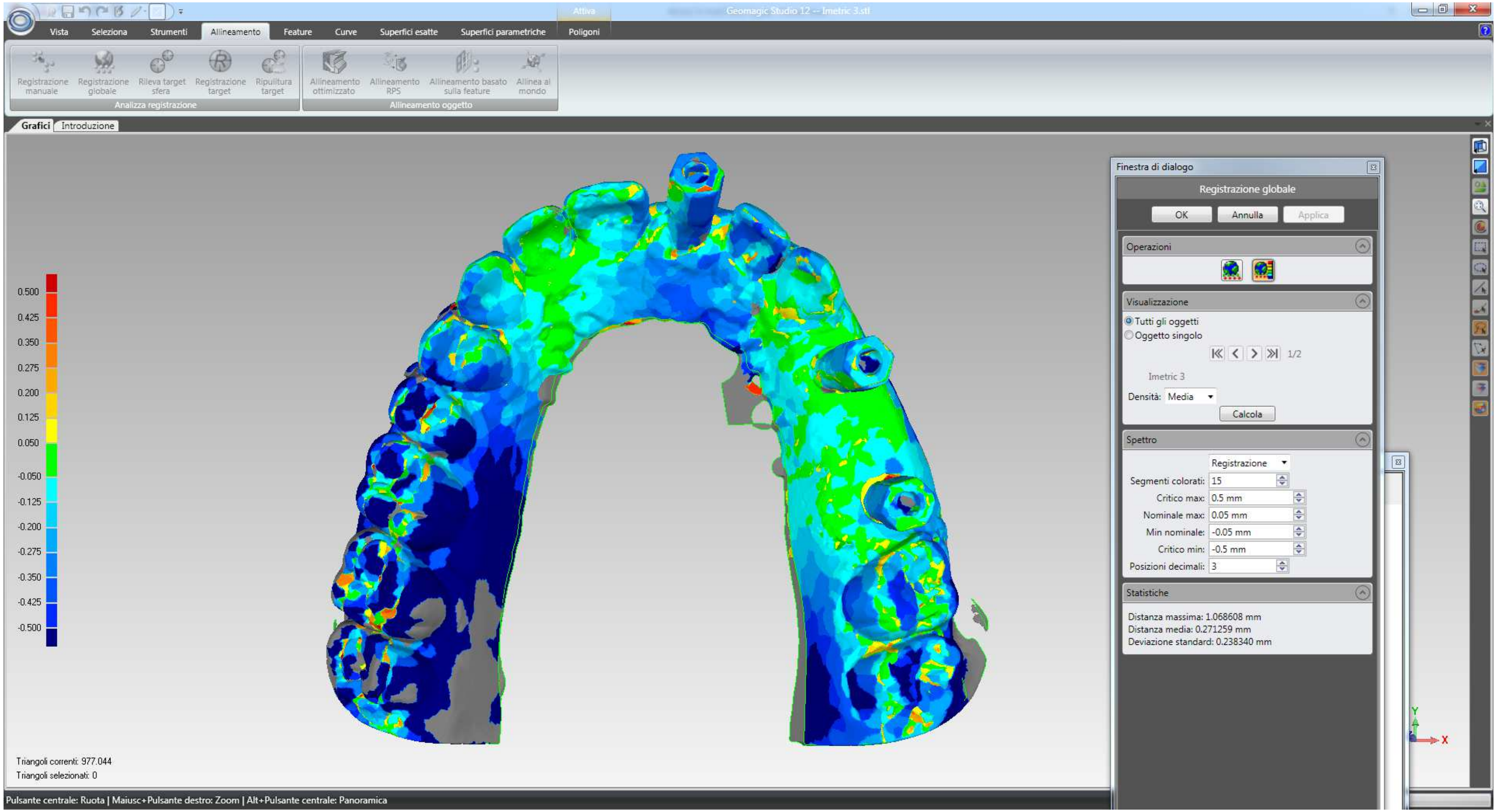

Plan 3 vs REF (IMETRIC)

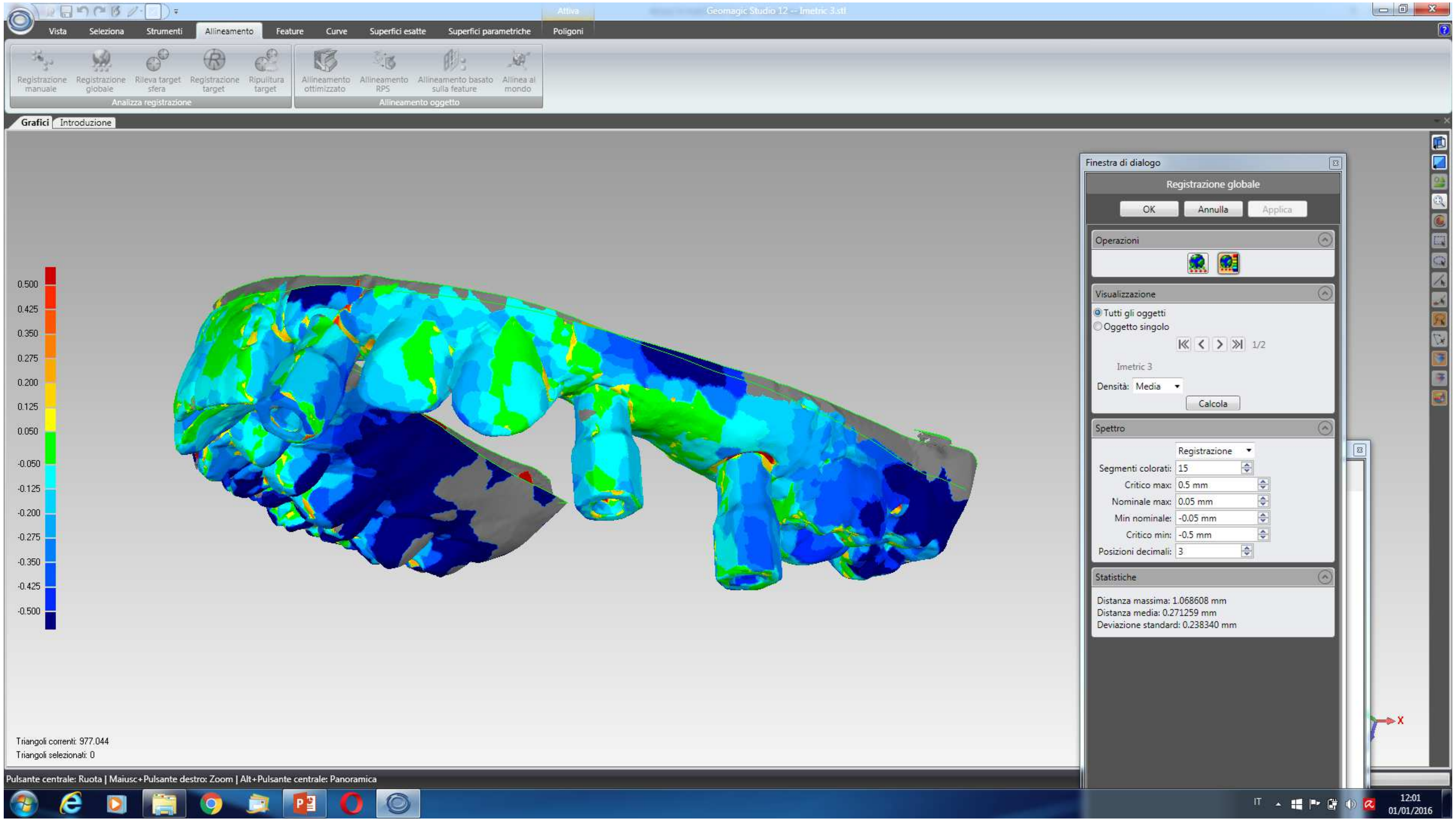

Plan 4 vs REF (IMETRIC)

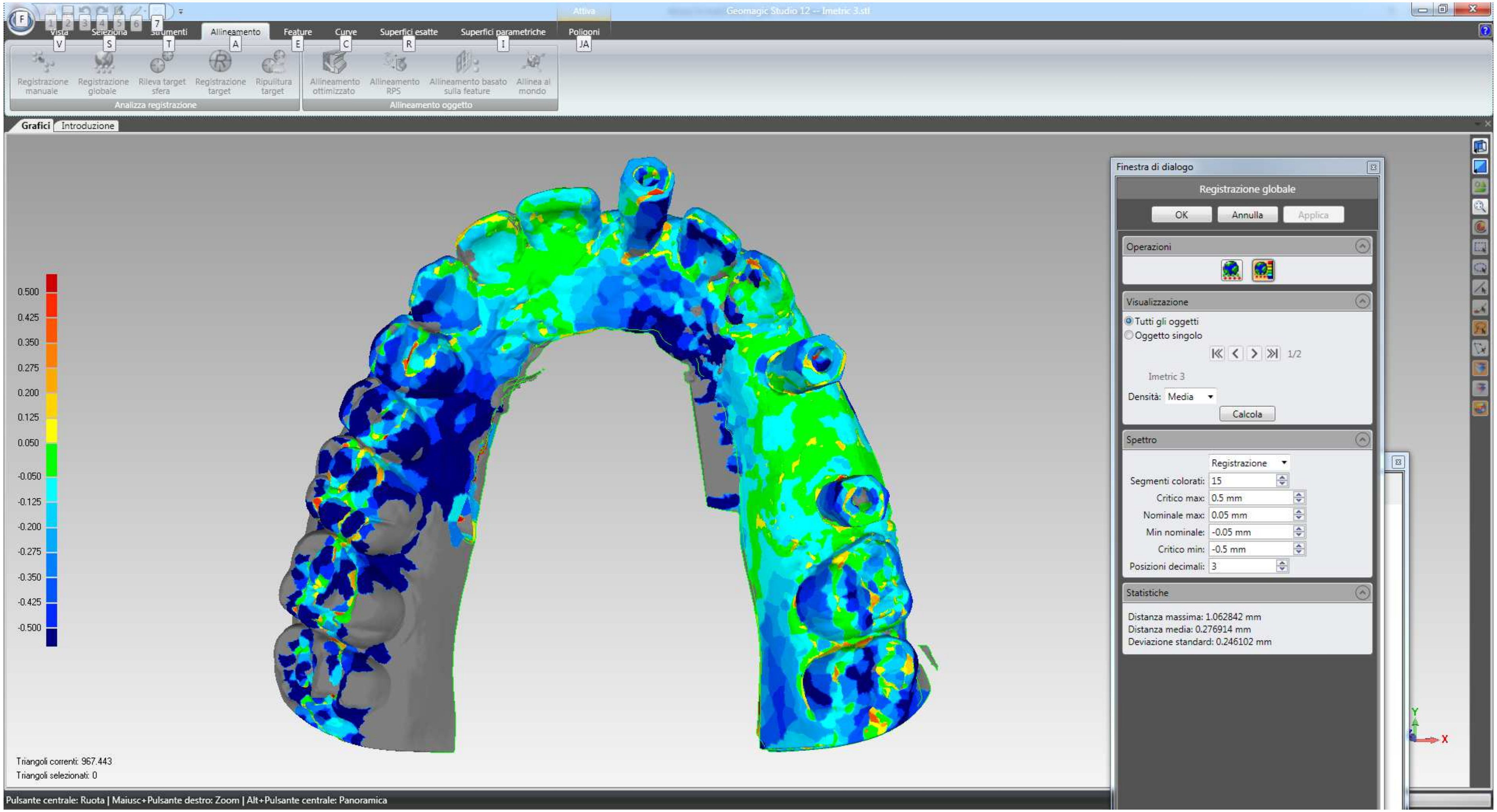

Plan 4 vs REF (IMETRIC)

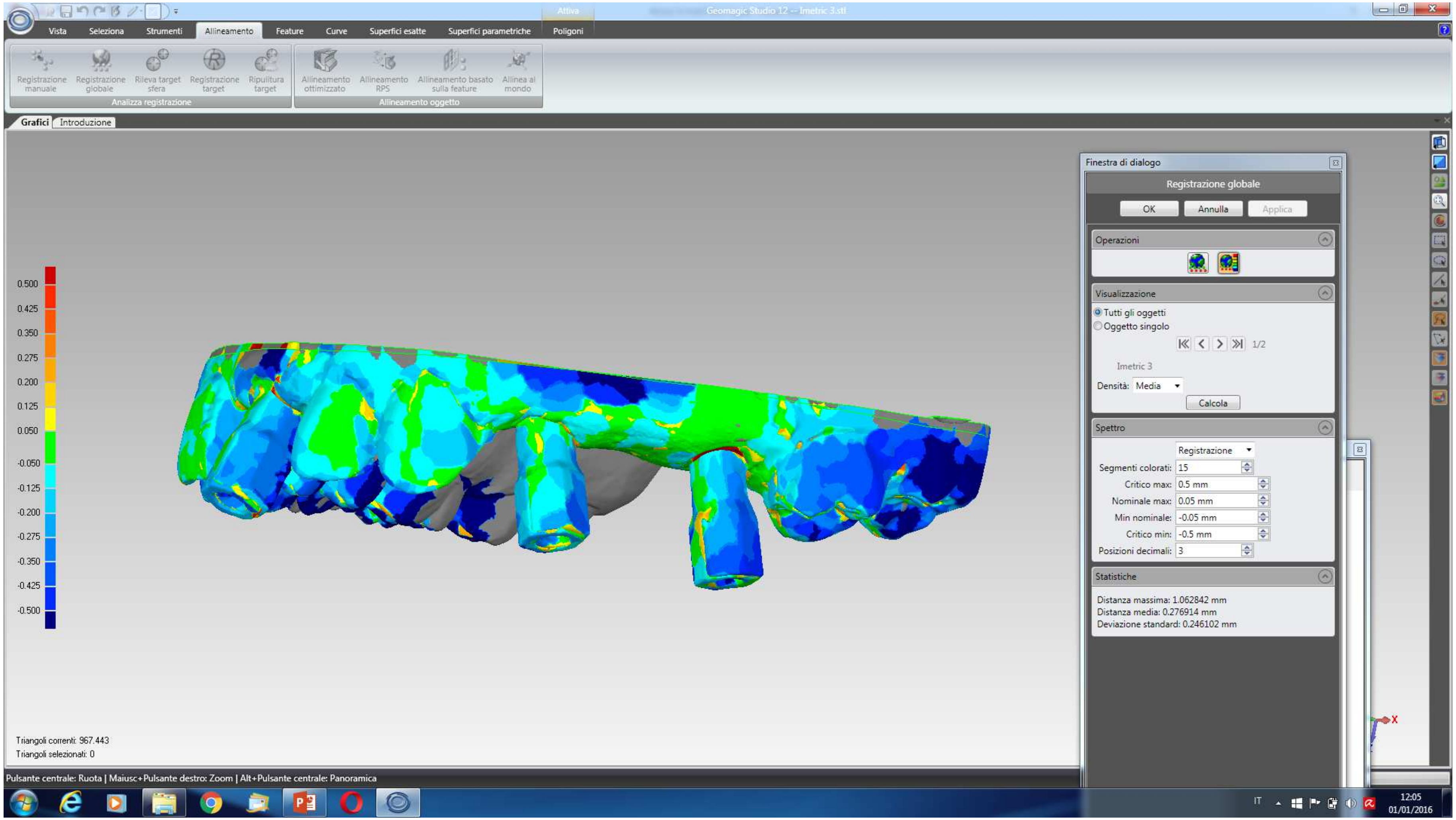

Plan 5 vs REF (IMETRIC)

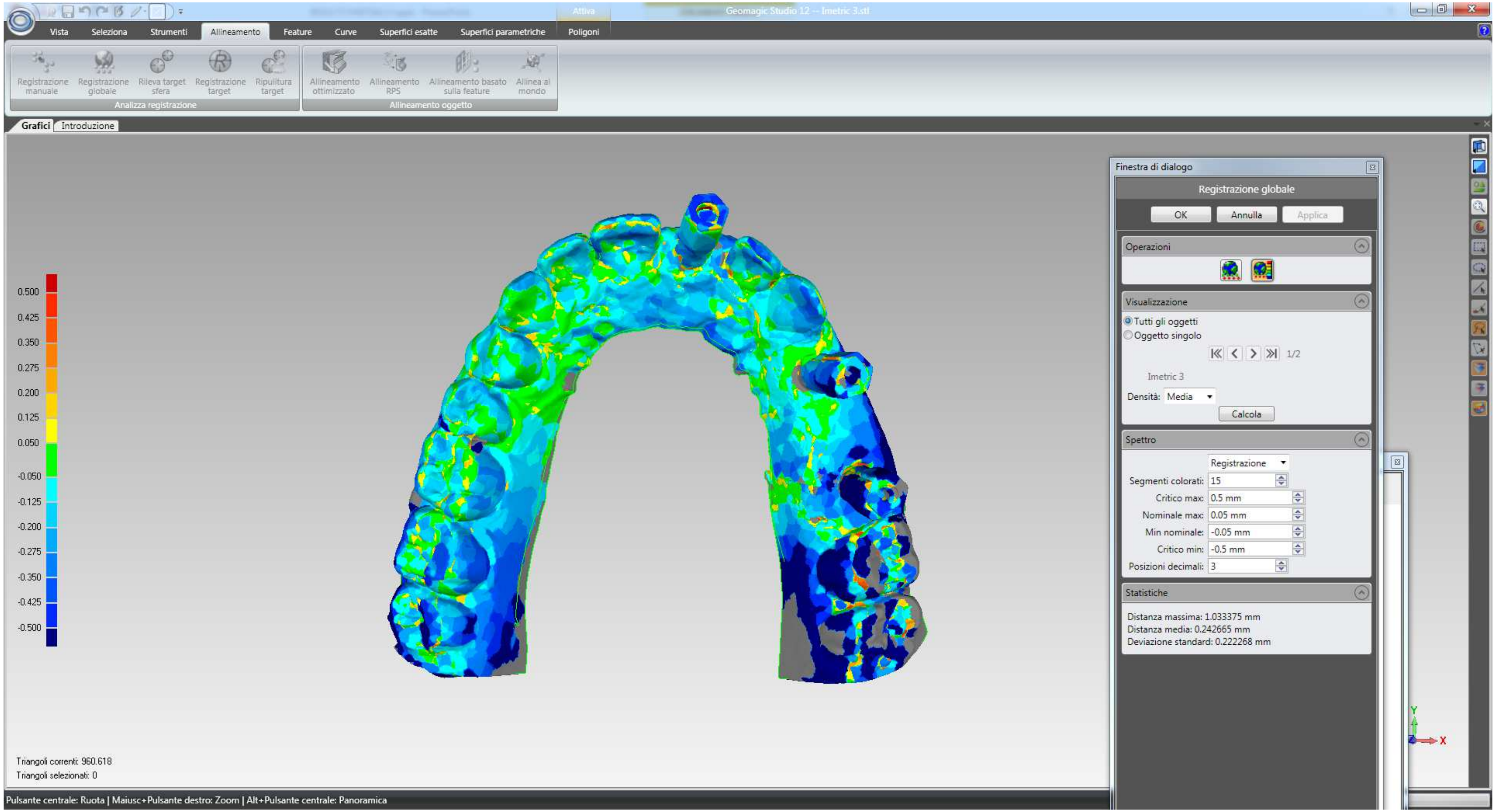

Plan 5 vs REF (IMETRIC)

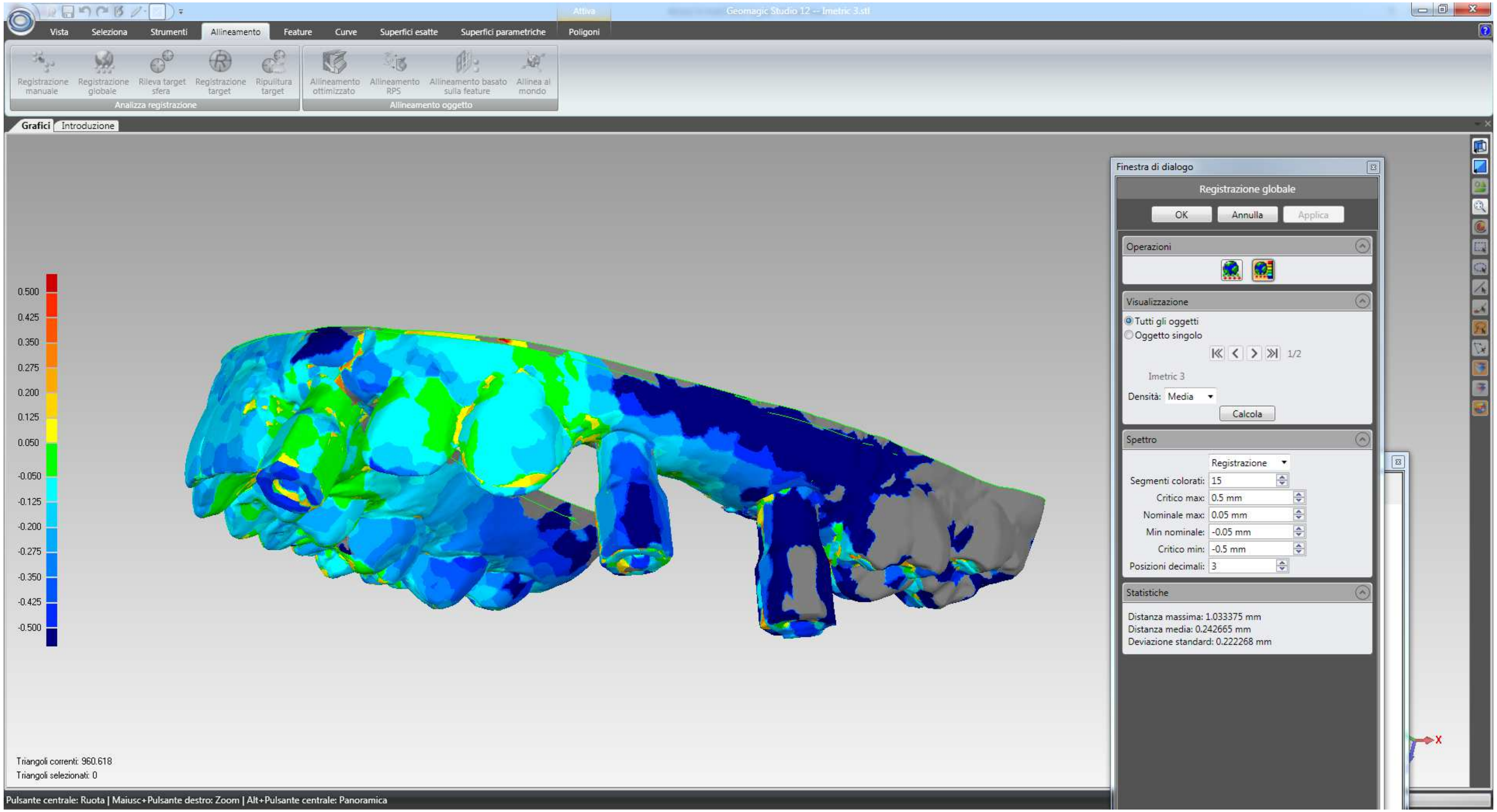

Planmeca general trueness in the partially edentulous model

	Mean distance	SD	Maximum distance
Plan 1	0.124	0.106	0.992
Plan 2	0.254	0.228	1.047
Plan 3	0.271	0.238	1.065
Plan 4	0.276	0.246	1.060
Plan 5	0.242	0.222	1.033

Overall Plane general trueness: 0.233 (0.062)

GENERAL PRECISION EVALUATIONS FOR TRIOS, ZFX INTRASCAN, CARESTREAM, PLANSCAN (SCANS WERE RANDOM BUT HERE WE ORDERED THEM) IN THE PARTIALLY EDENTULOUS MODEL

GENERAL SETTINGS FOR FINAL REGISTRATION (TRUENESS):

0.5 mm, 0.05 mm, -0.05 mm, -0.5 mm (see scale)

Trios 5 as reference (best accuracy) vs trios 1

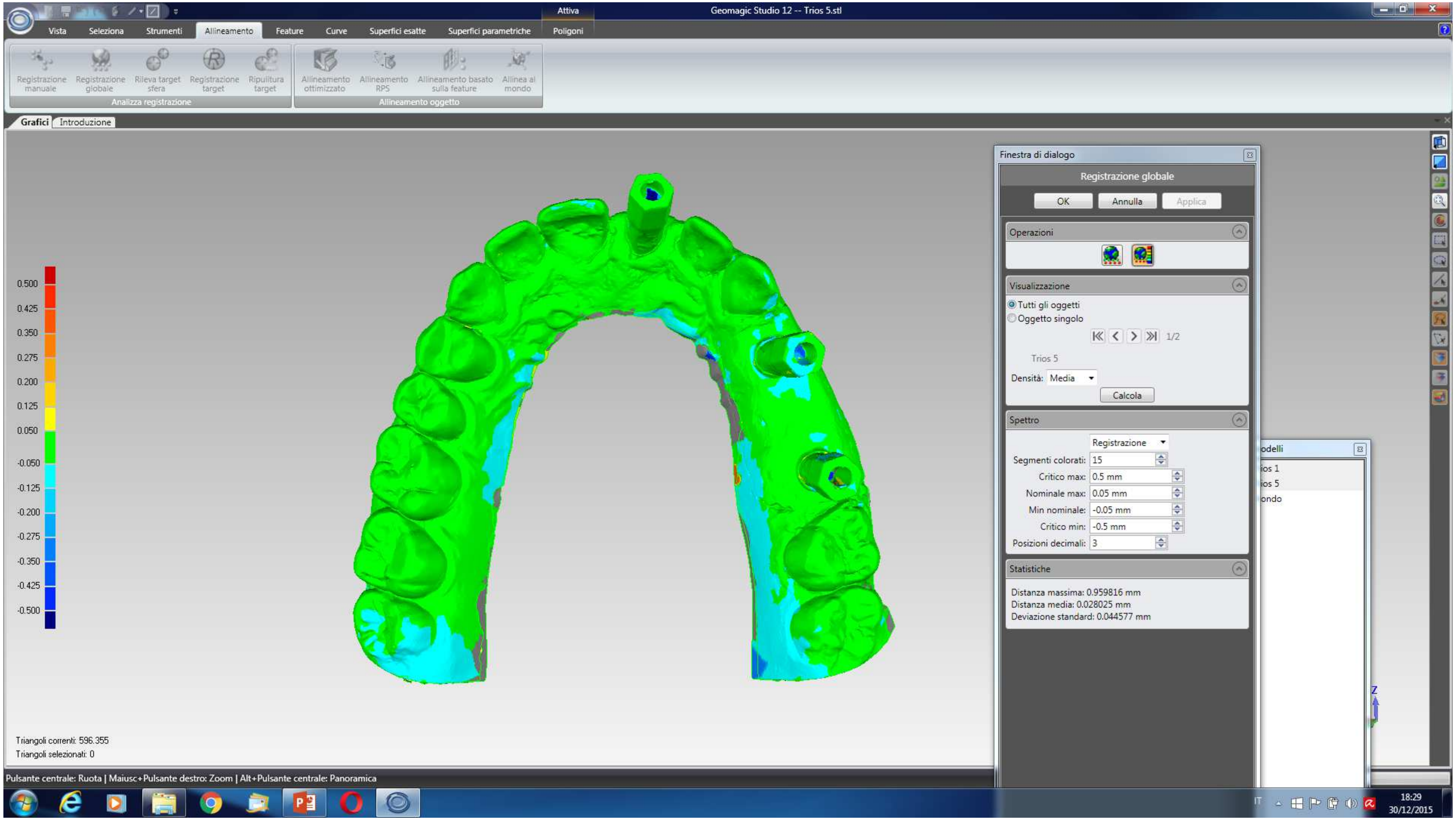

Trios 5 as reference (best accuracy) vs trios 1

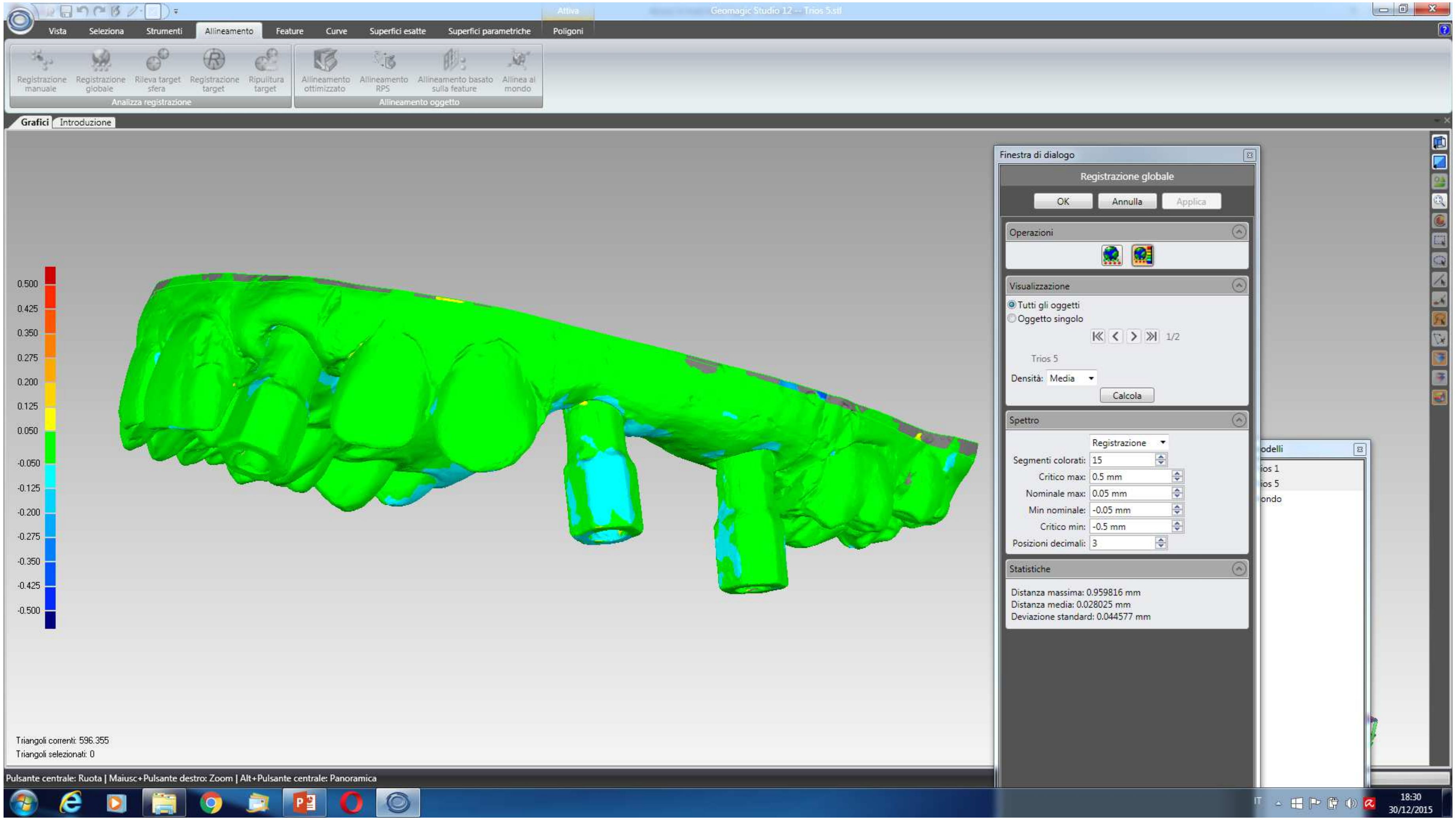

Trios 5 as reference (best accuracy) vs trios 2

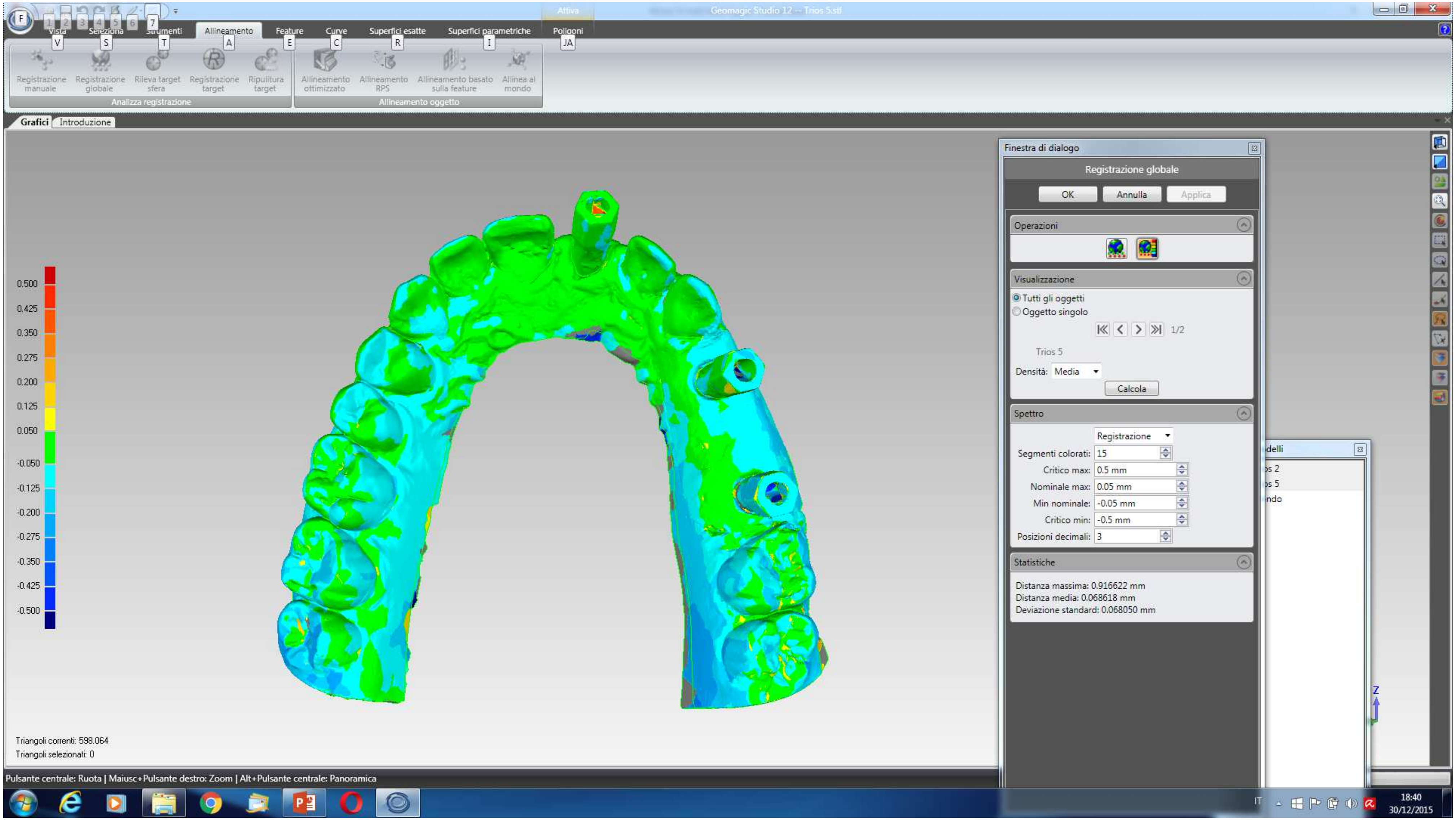

Trios 5 as reference (best accuracy) vs trios 2

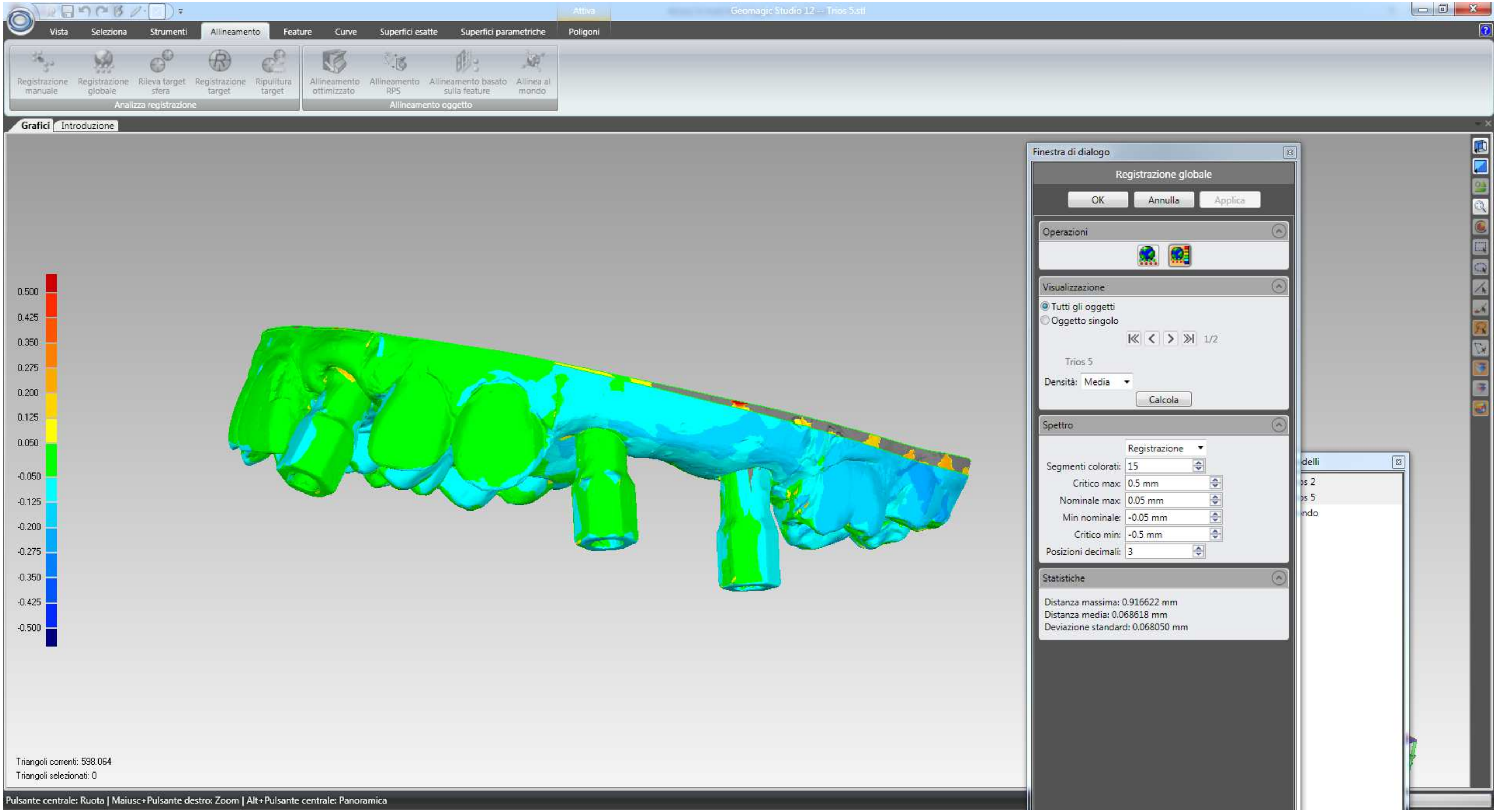

Trios 5 as reference (best accuracy) vs trios 3

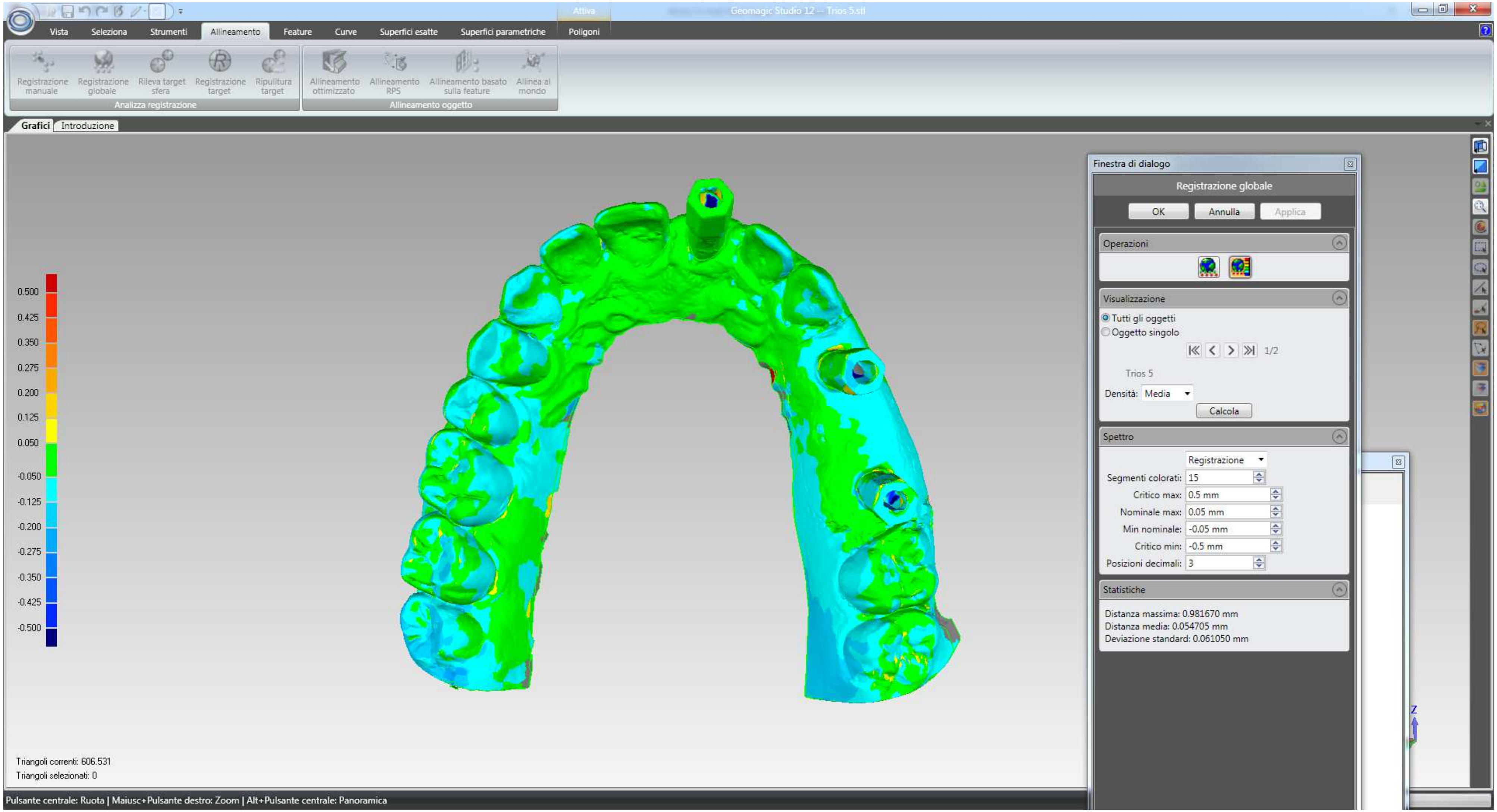

Trios 5 as reference (best accuracy) vs trios 3

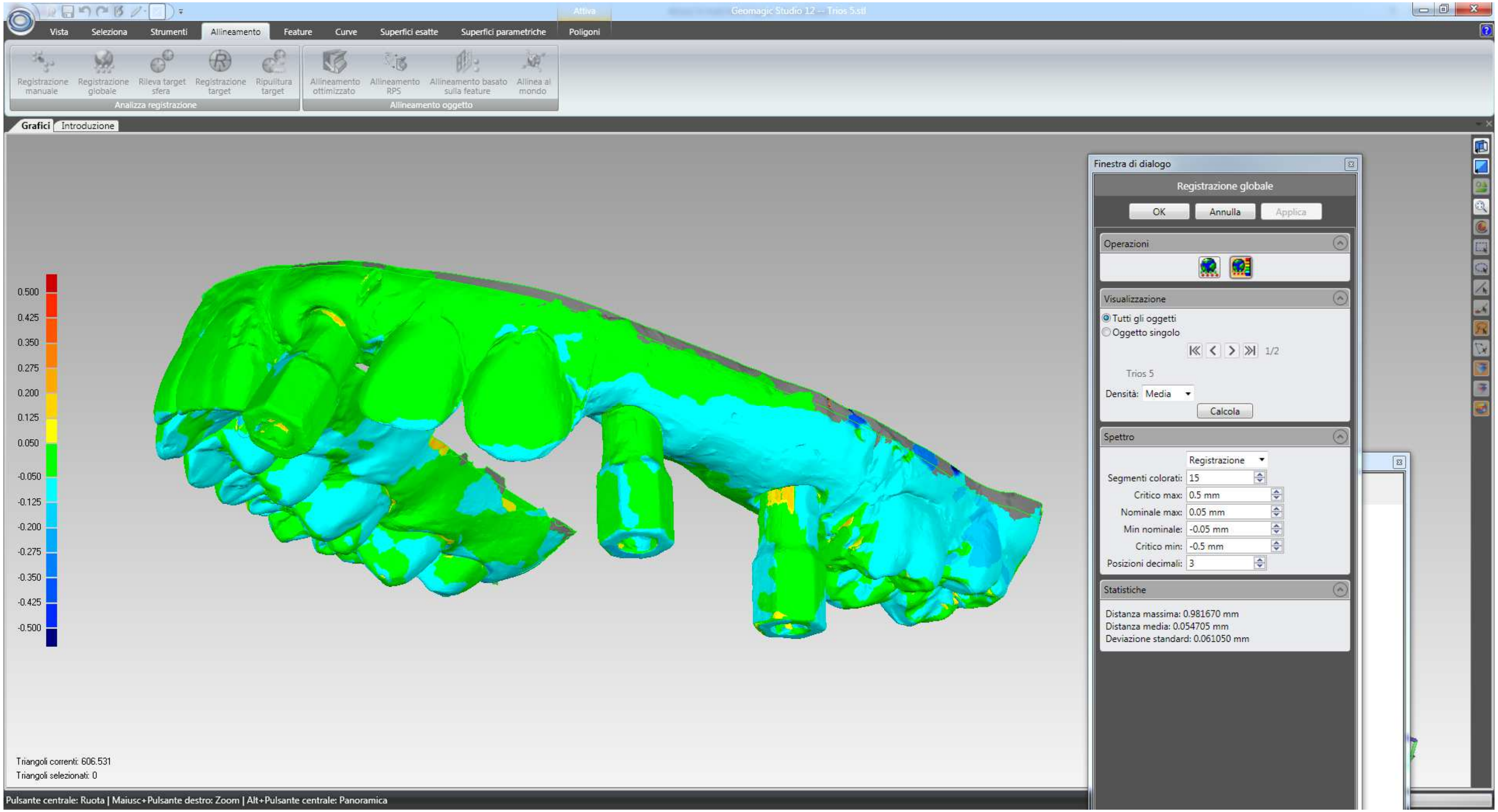

Trios 5 as reference (best accuracy) vs trios 4

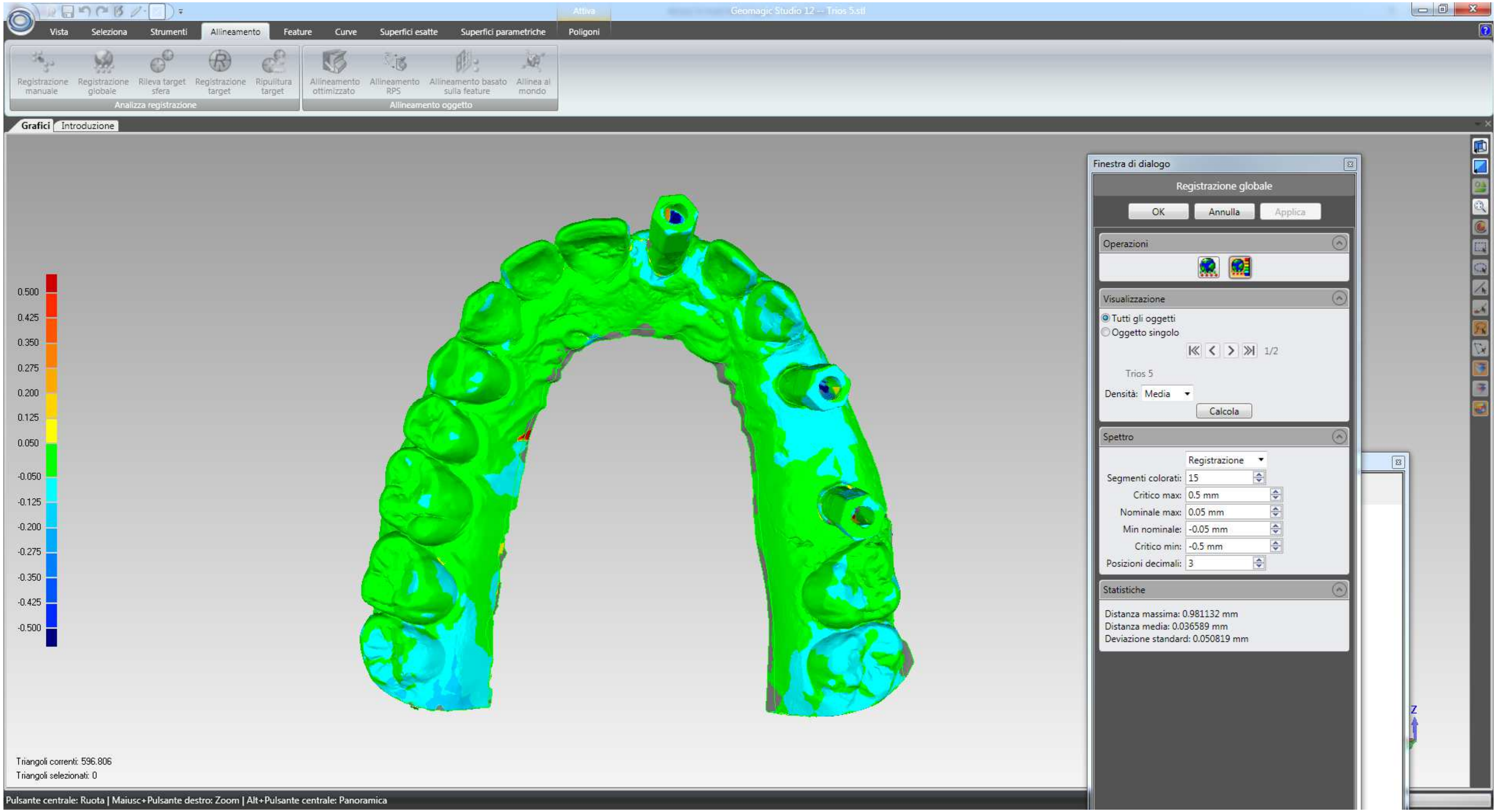

Trios 5 as reference (best accuracy) vs trios 4

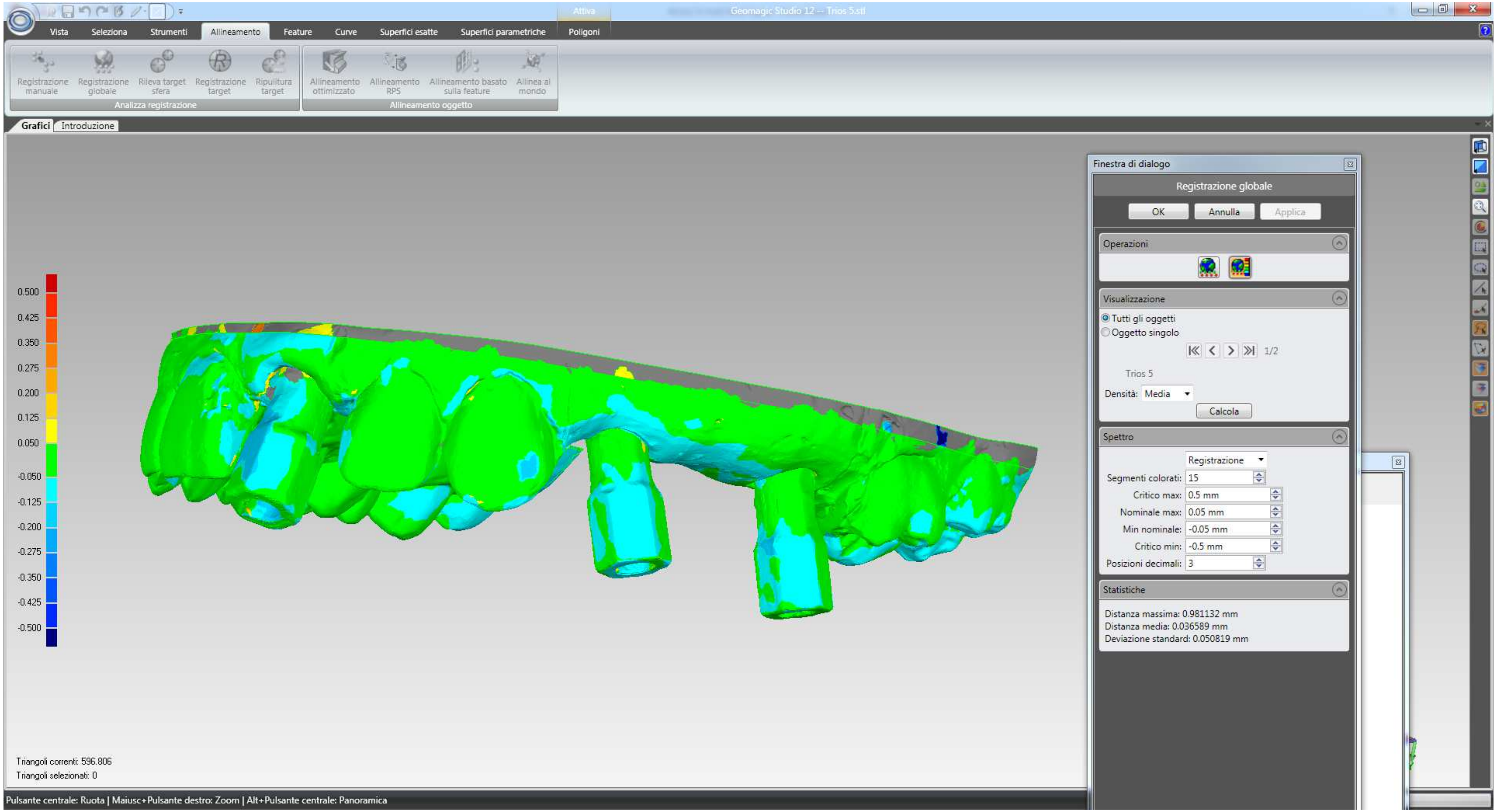

Trios 2 vs trios 4

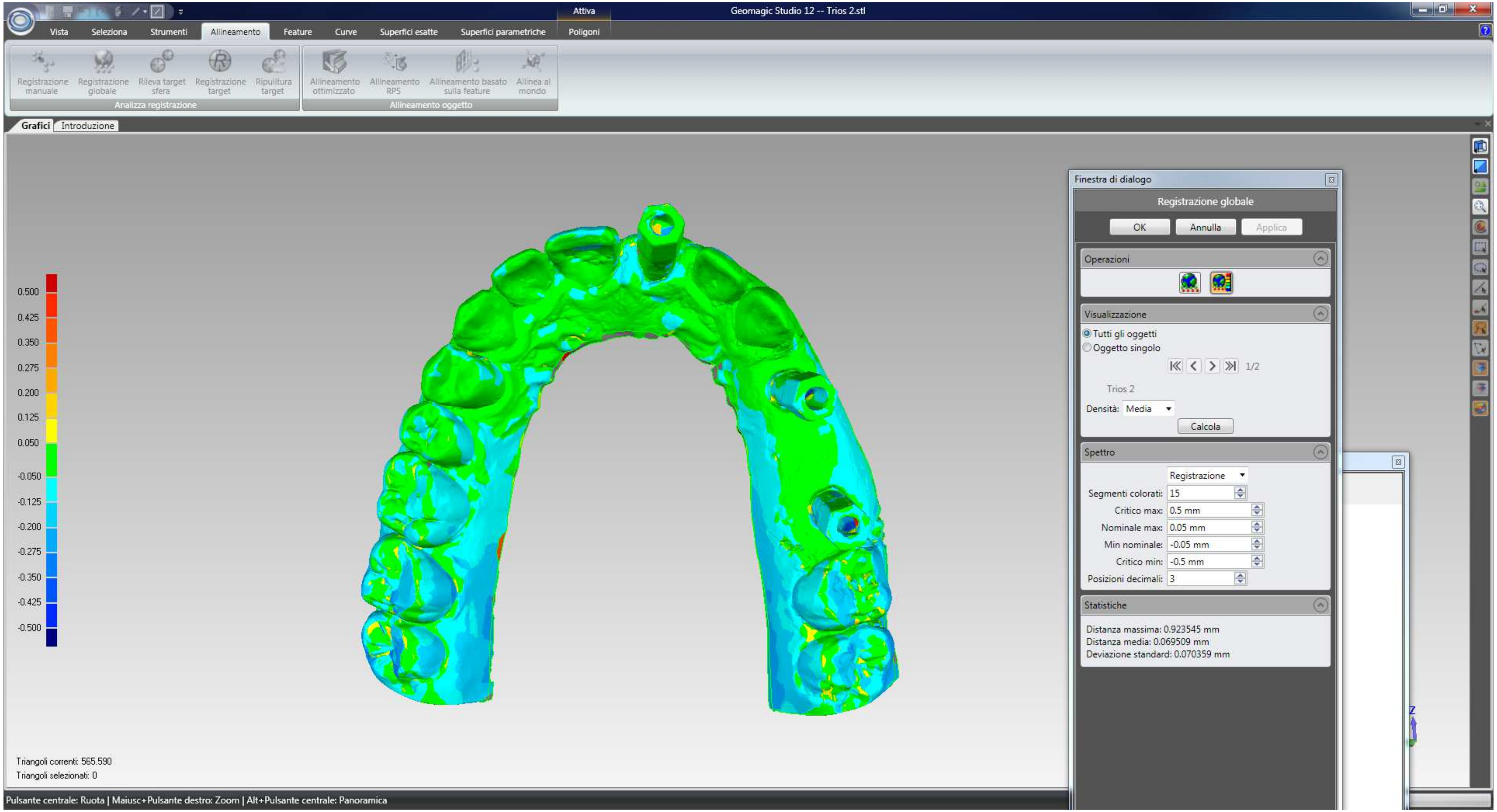

Trios 2 vs trios 4

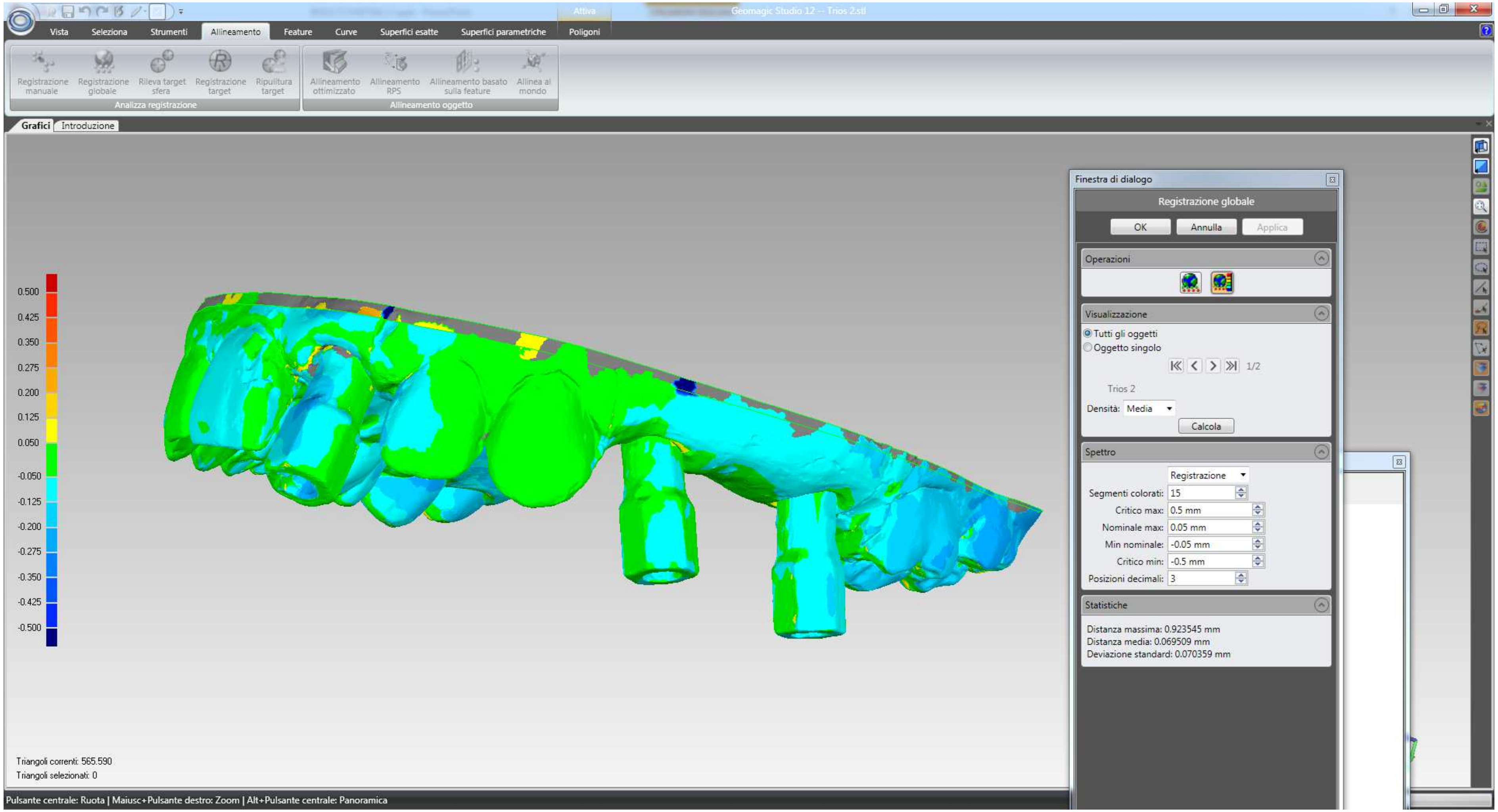

TRIOS overall general precision in the partially edentulous model

	Mean distance	SD	Maximum distance
Trios 5 vs 1	0.028	0.044	0.959
Trios 5 vs 2	0.068	0.068	0.916
Trios 5 vs 3	0.054	0.061	0.981
Trios 5 vs 4	0.036	0.050	0.981
Trios 2 vs 4	0.069	0.070	0.923

Overall Trios general precision: 0.051 (0.018)

Zfx 5 as reference (best accuracy) vs Zfx 1

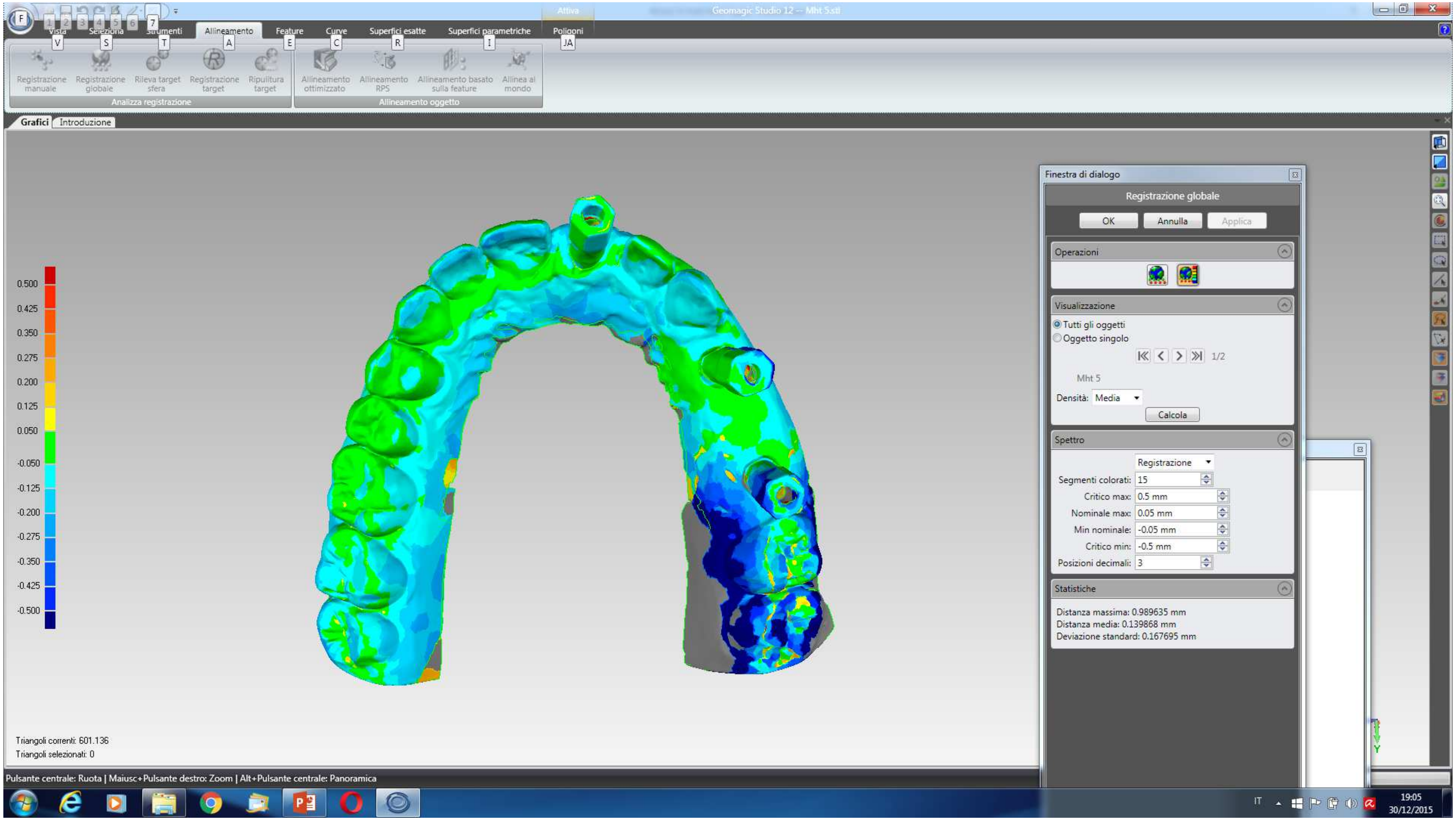

Zfx 5 as reference (best accuracy) vs Zfx 1

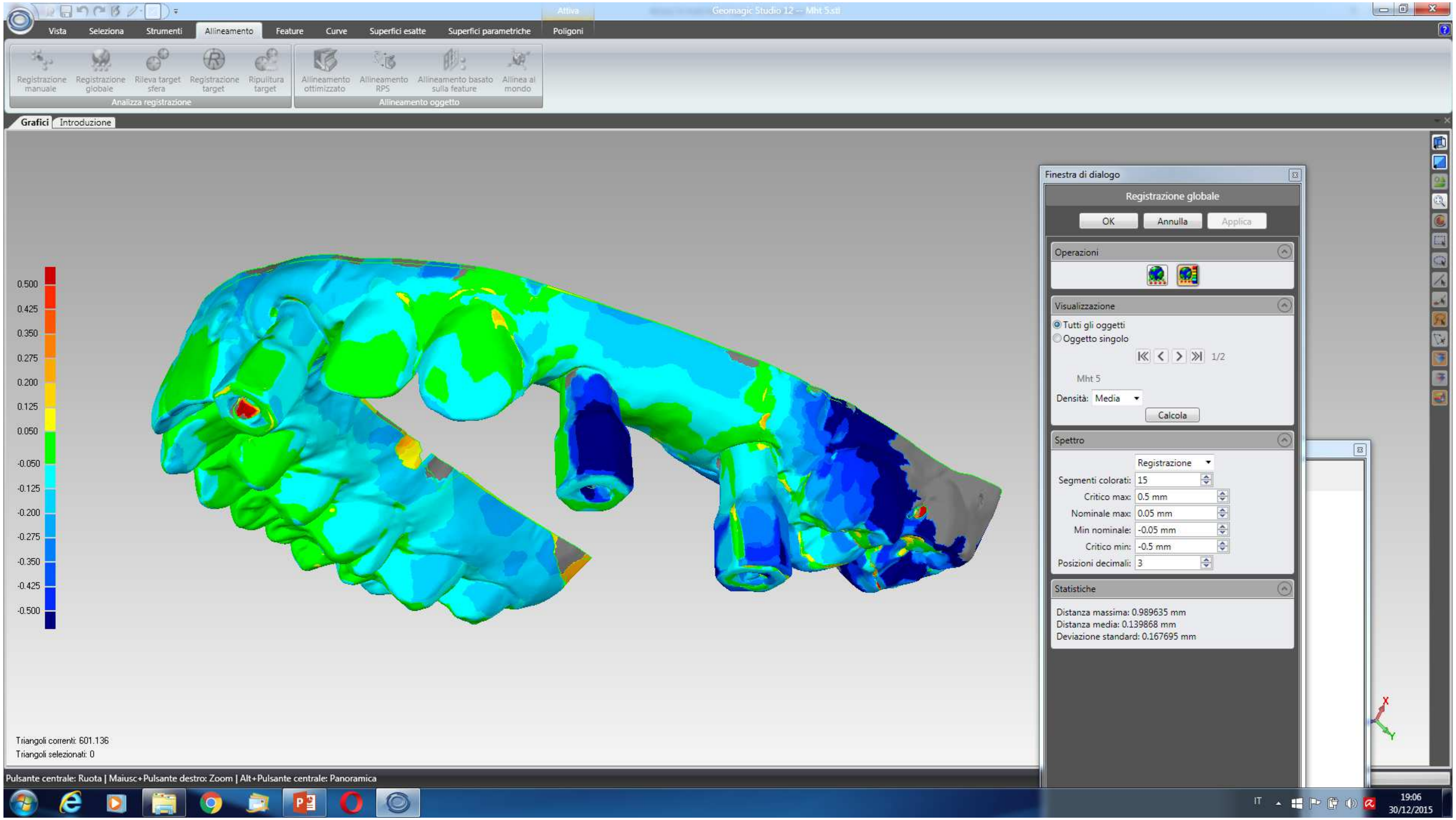

Zfx 5 as reference (best accuracy) vs Zfx 2

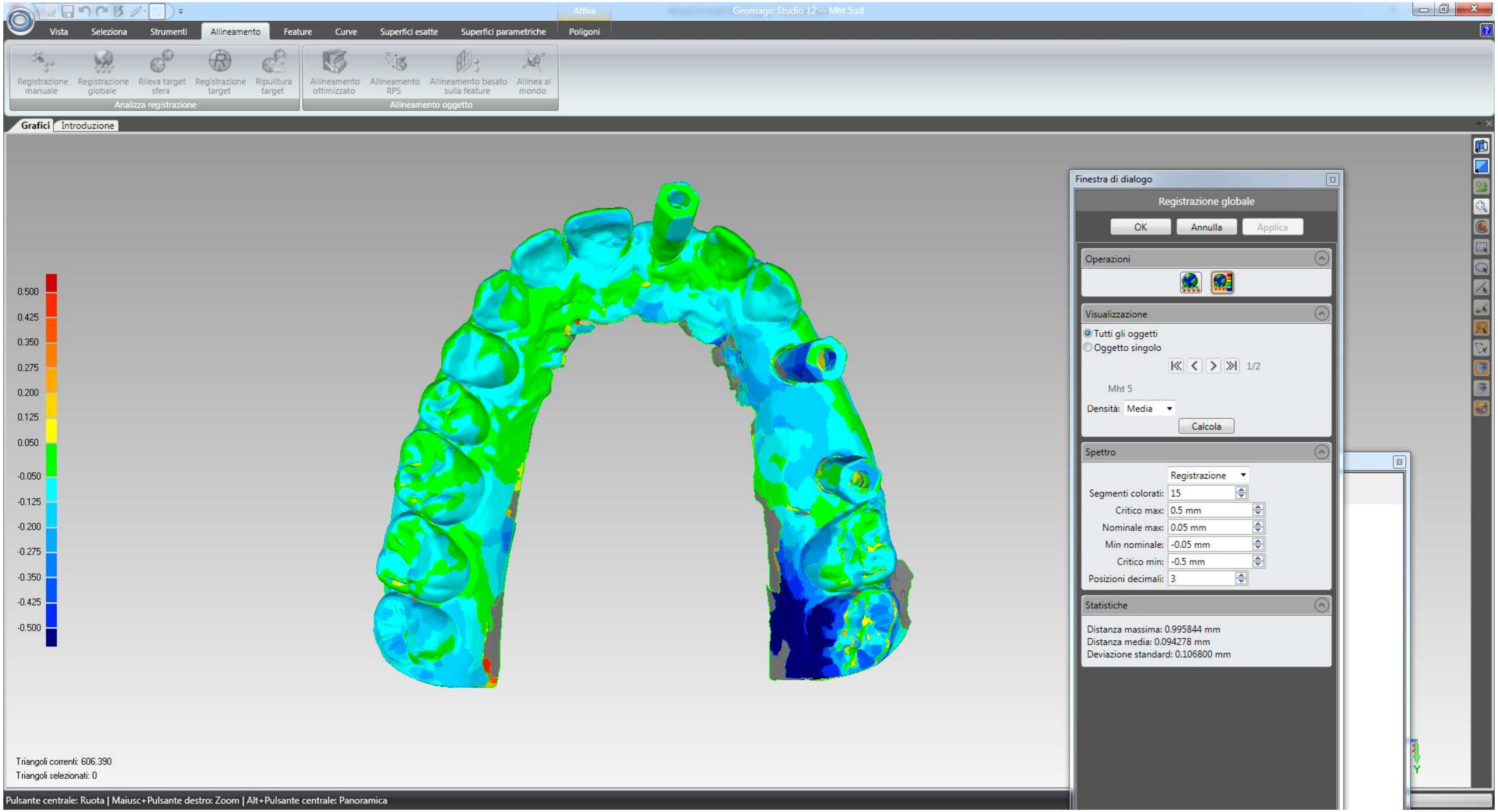

Zfx 5 as reference (best accuracy) vs Zfx 2

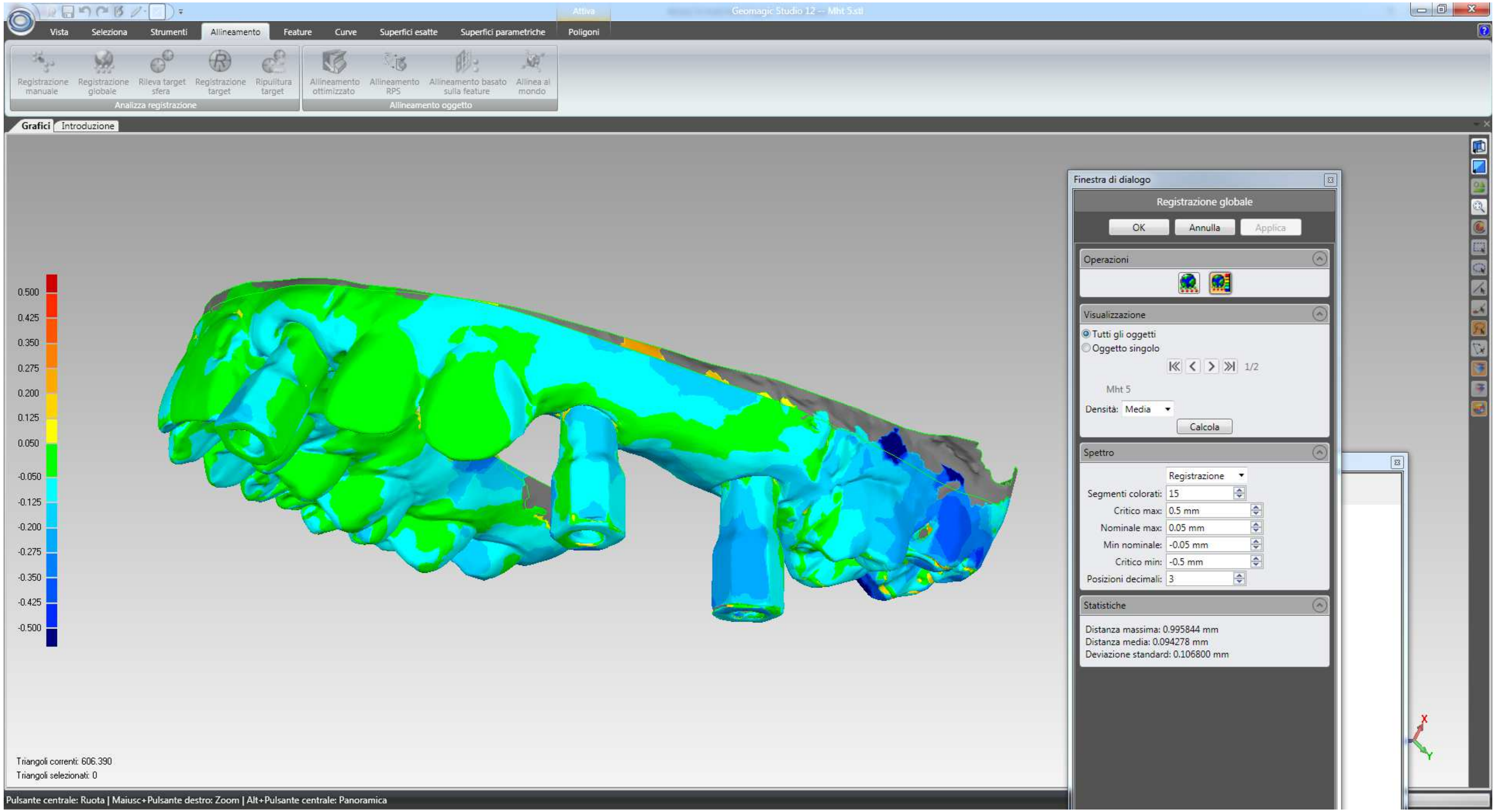

Zfx 5 as reference (best accuracy) vs Zfx 3

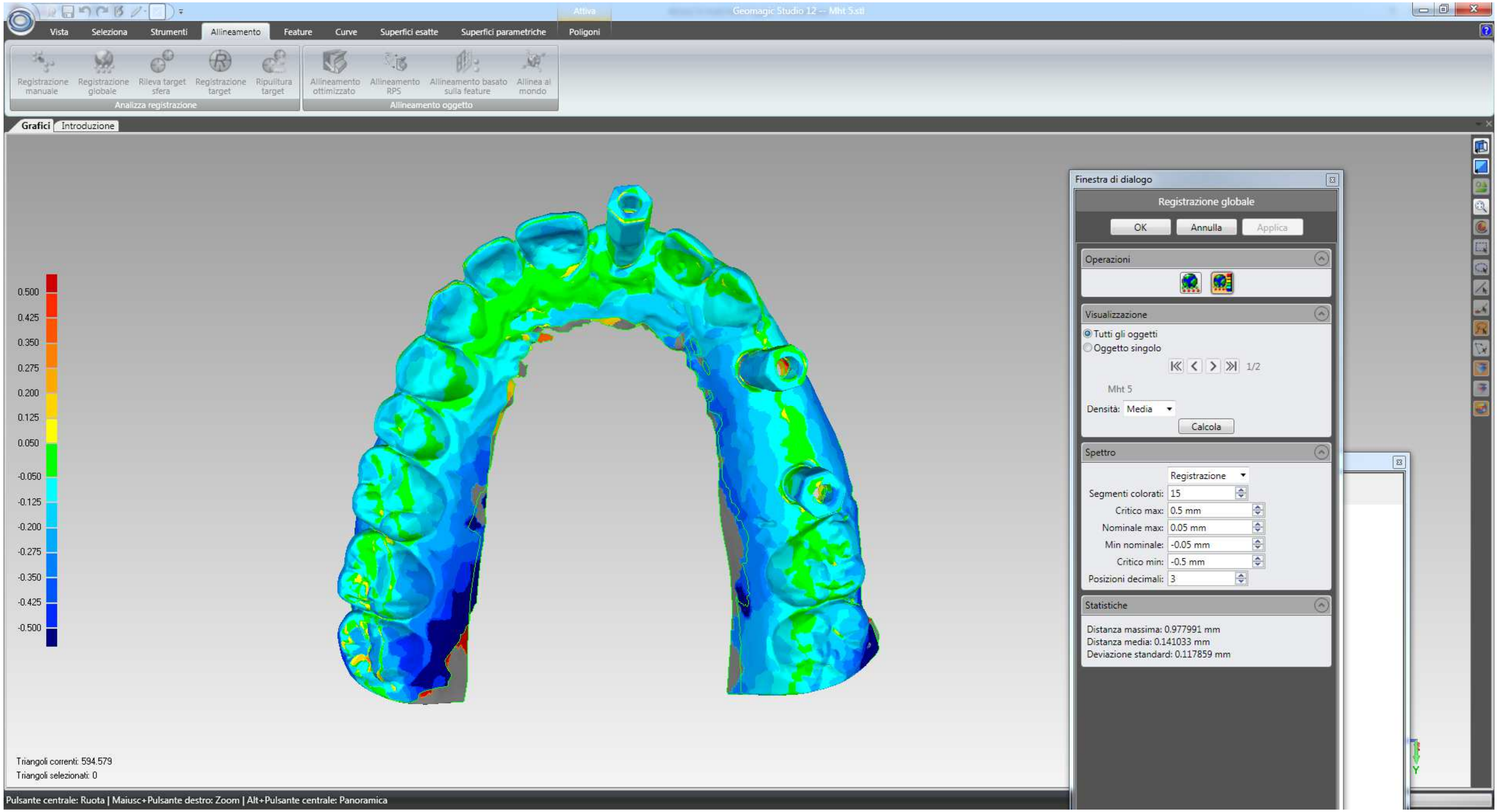

Zfx 5 as reference (best accuracy) vs Zfx 3

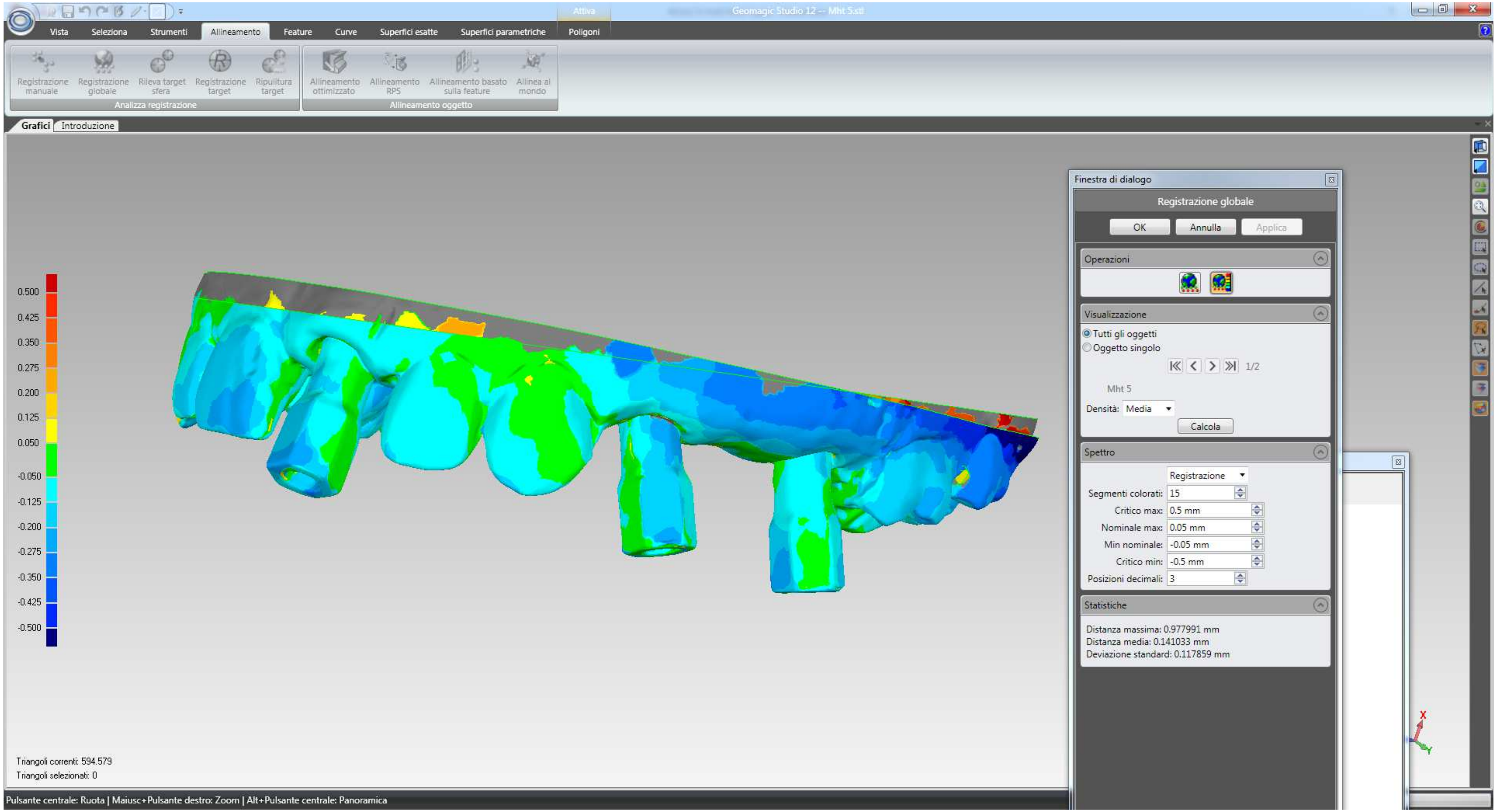

Zfx 5 as reference (best accuracy) vs Zfx 4

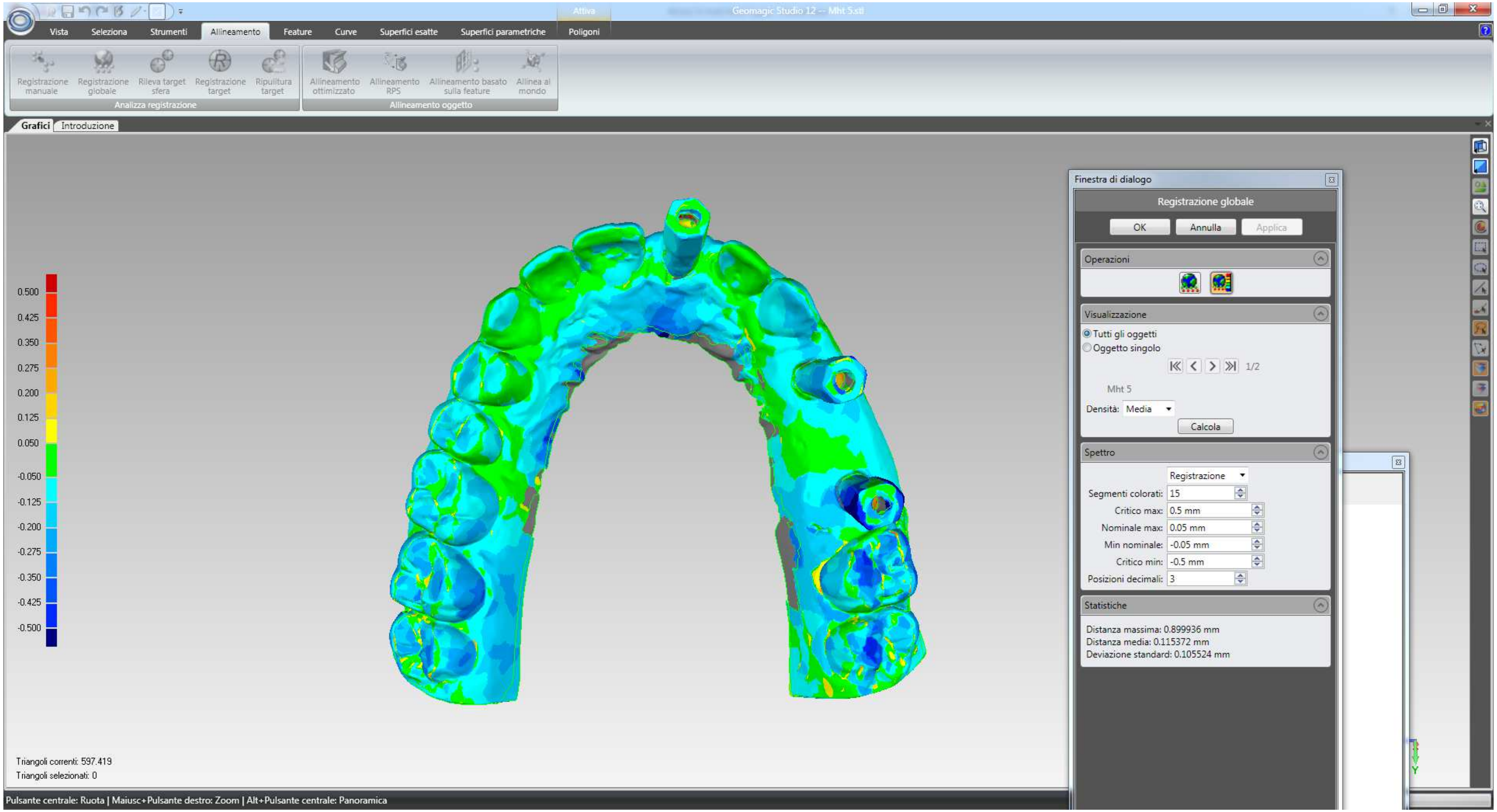

Zfx 5 as reference (best accuracy) vs Zfx 4

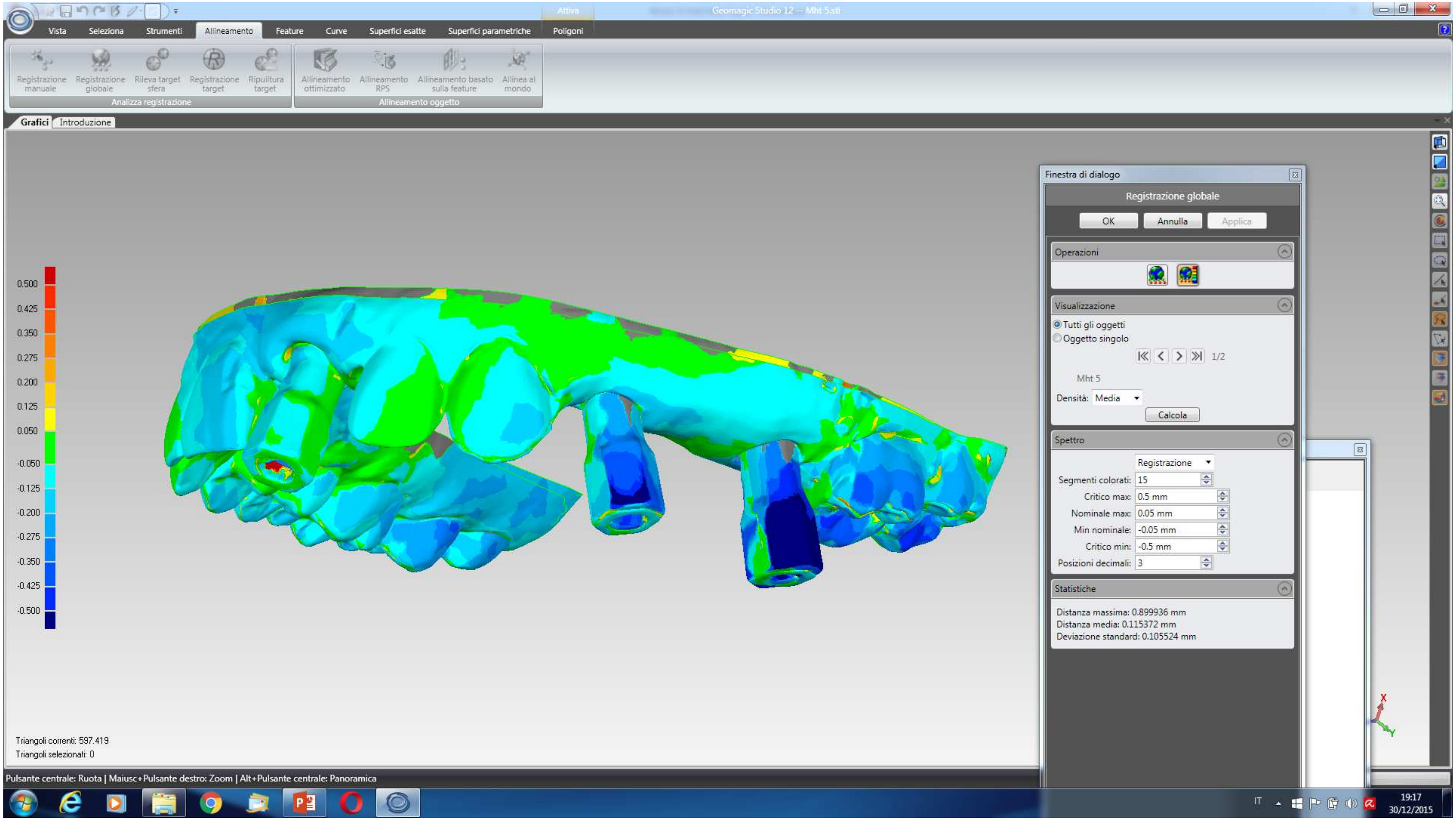

Zfx 2 vs Zfx 4

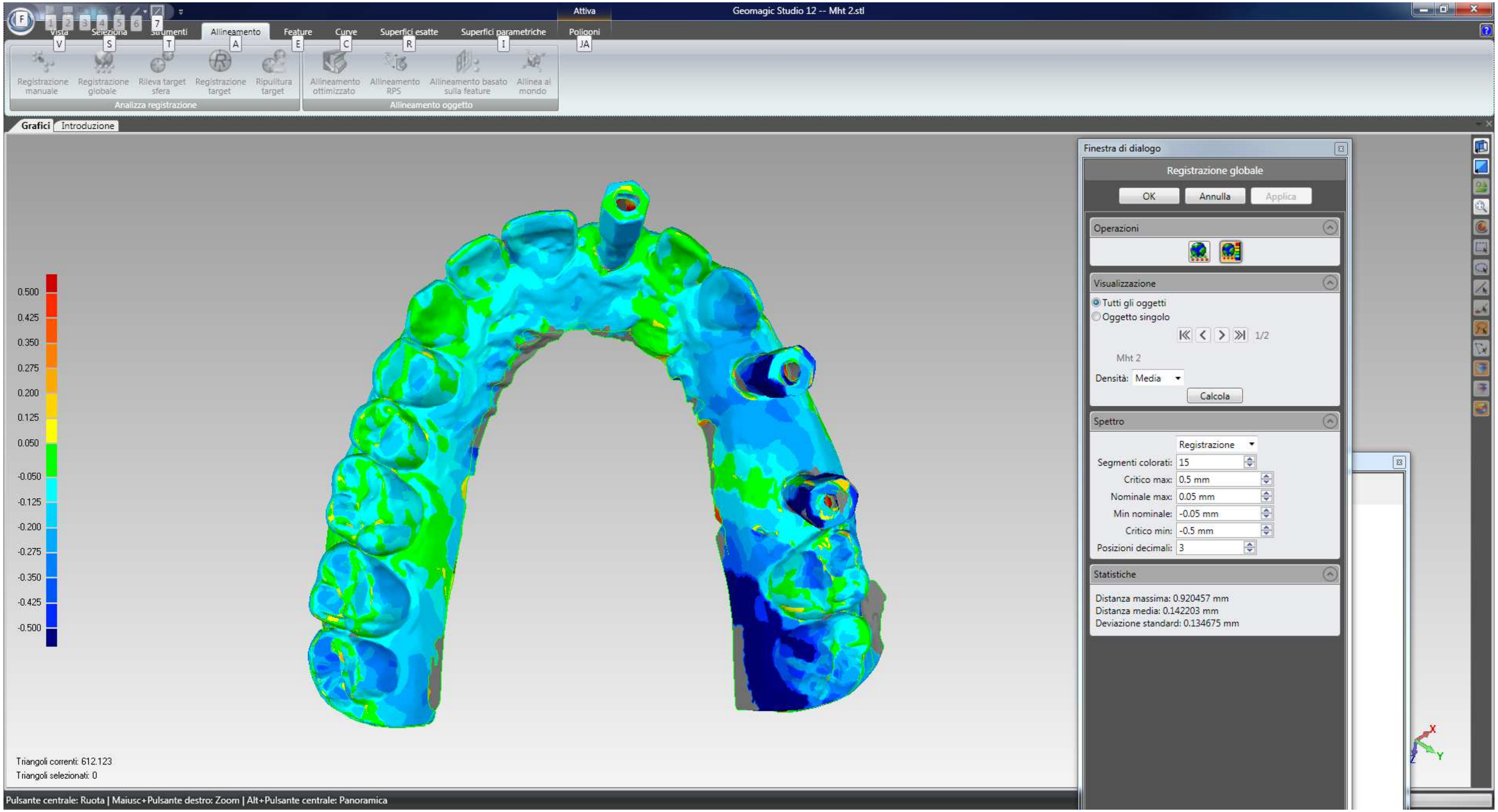
Geomagic Studio 12 -- Mht 2.stl
Registrazione globale
OK
Annulla
Applica
Operazioni
Visualizzazione
Tutti gli oggetti
Oggetto singolo
Mht 2
Densità: Media
Calcola
Spettro
Registrazione
Segmenti colorati: 15
Critico max: 0.5 mm
Nominale max: 0.05 mm
Min nominale: -0.05 mm
Critico min: -0.5 mm
Posizioni decimali: 3
Statistiche
Distanza massima: 0.920457 mm
Distanza media: 0.142203 mm
Deviazione standard: 0.134675 mm
0.500
0.425
0.350
0.275
0.200
0.125
0.050
-0.050
-0.125
-0.200
-0.275
-0.350
-0.425
-0.500
Triangoli correnti: 612.123
Triangoli selezionati: 0
Pulsante centrale: Ruota | Maiusc+Pulsante destro: Zoom | Alt+Pulsante centrale: Panoramica

Zfx 2 vs Zfx 4

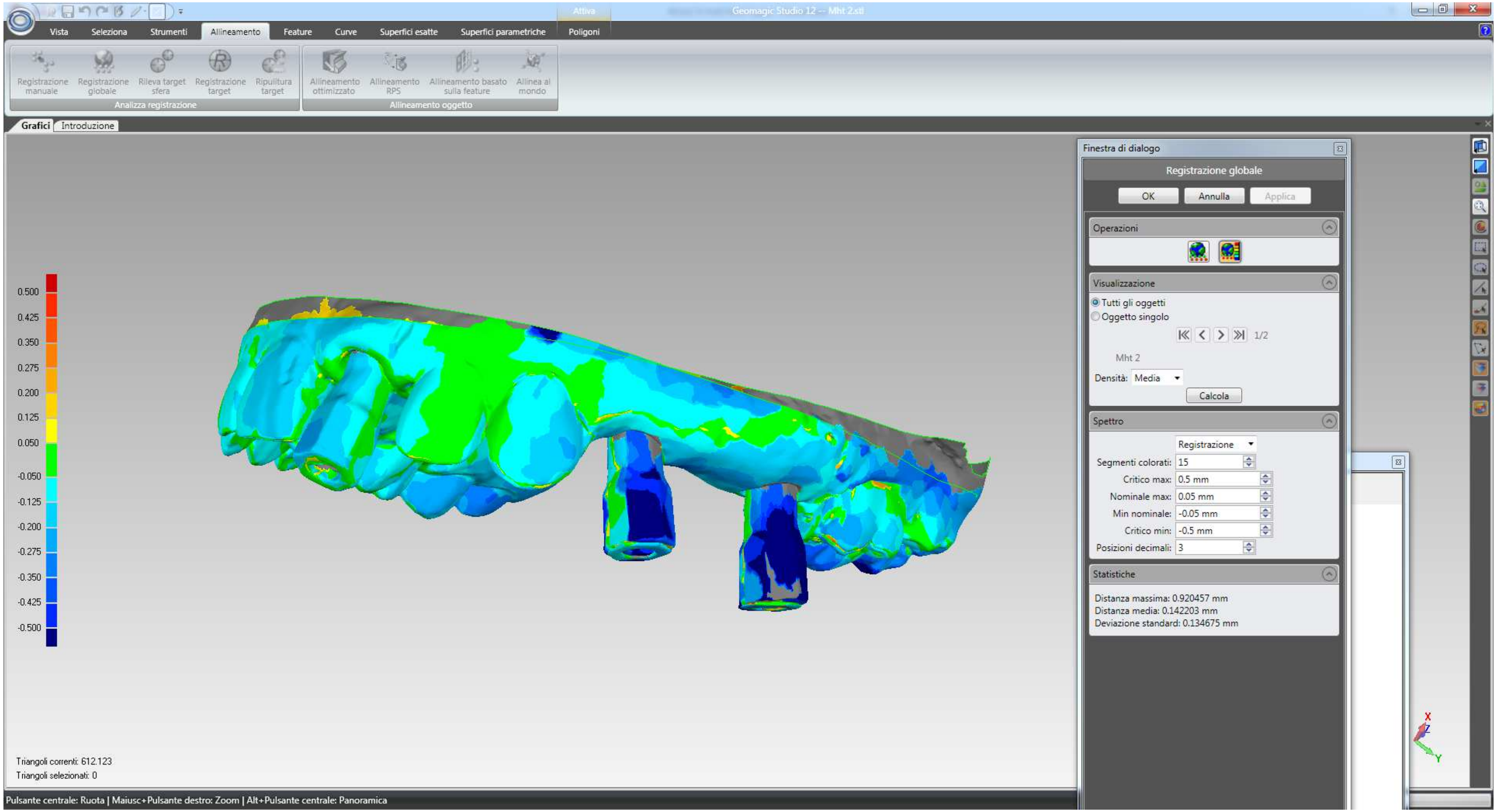
Geomagic Studio 12 -- Mht 2.stl
Vista
Seleziona
Strumenti
Allineamento
Feature
Curve
Superfici esatte
Superfici parametriche
Poligoni
Registrazione manuale
Registrazione globale
Rileva target sfera
Registrazione target
Ripulitura target
Analizza registrazione
Allineamento ottimizzato
Allineamento RPS
Allineamento basato sulla feature
Allinea al mondo
Allineamento oggetto
Grafici
Introduzione
0.500
0.425
0.350
0.275
0.200
0.125
0.050
-0.050
-0.125
-0.200
-0.275
-0.350
-0.425
-0.500
Triangoli correnti: 612.123
Triangoli selezionati: 0
Finestra di dialogo
Registrazione globale
OK
Annulla
Applica
Operazioni
Visualizzazione
Tutti gli oggetti
Oggetto singolo
1/2
Mht 2
Densità: Media
Calcola
Spettro
Registrazione
Segmenti colorati: 15
Critico max: 0.5 mm
Nominale max: 0.05 mm
Min nominale: -0.05 mm
Critico min: -0.5 mm
Posizioni decimali: 3
Statistiche
Distanza massima: 0.920457 mm
Distanza media: 0.142203 mm
Deviazione standard: 0.134675 mm
Pulsante centrale: Ruota | Maiusc+Pulsante destro: Zoom | Alt+Pulsante centrale: Panoramica

General precision of Zfx in the partially edentulous model

	Mean distance	SD	Maximum distance
Zfx 5 vs Zfx 1	0.139	0.167	0.989
Zfx 5 vs Zfx 2	0.094	0.106	0.995
Zfx 5 vs Zfx 3	0.141	0.117	0.977
Zfx 5 vs Zfx 4	0.115	0.105	0.899
Zfx 2 vs Zfx 4	0.142	0.134	0.920

Overall Zfx general precision: 0.126 (0.021)

Care 2 as reference (best accuracy) vs Care 1

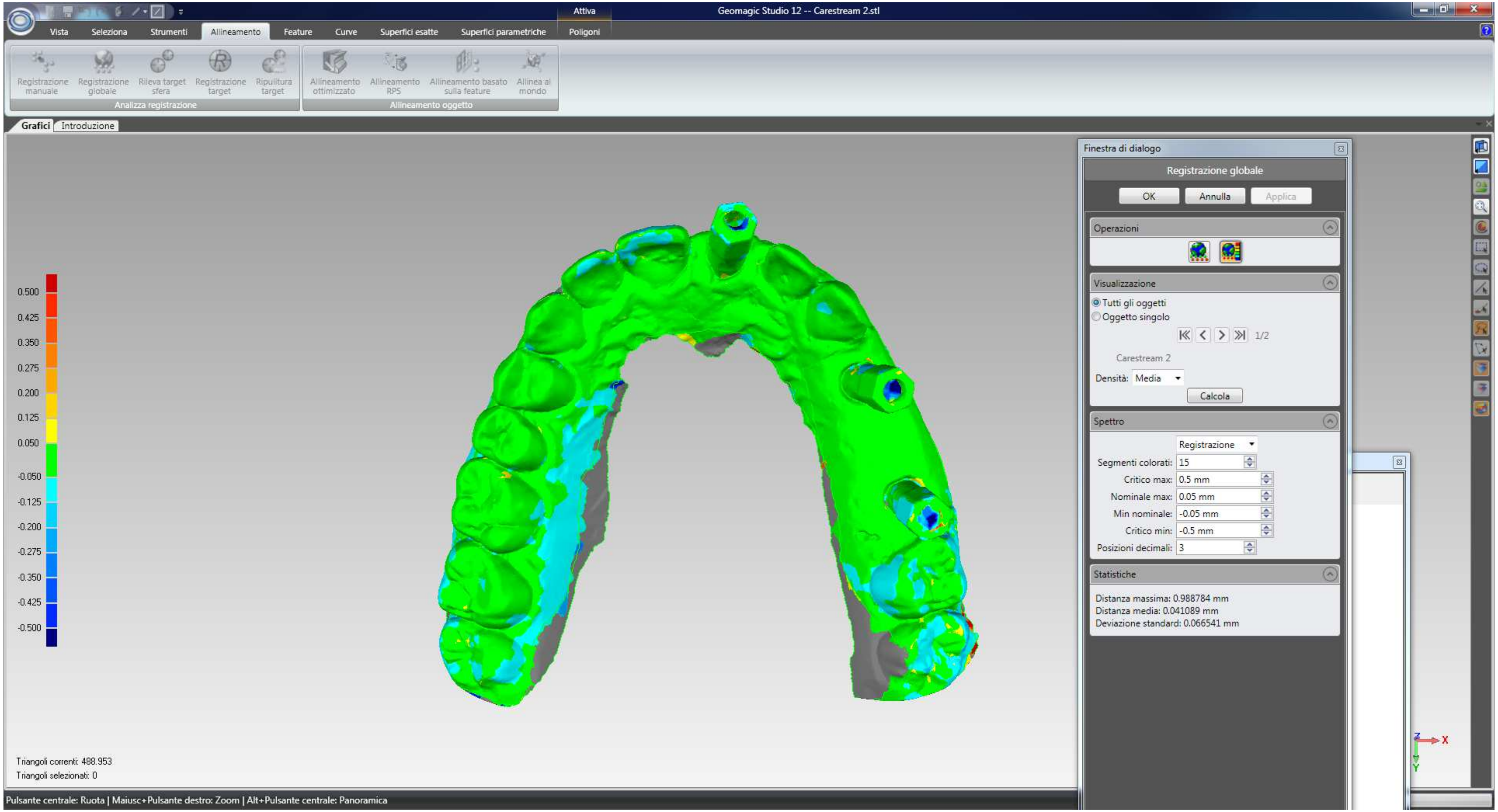

Care 2 as reference (best accuracy) vs Care 1

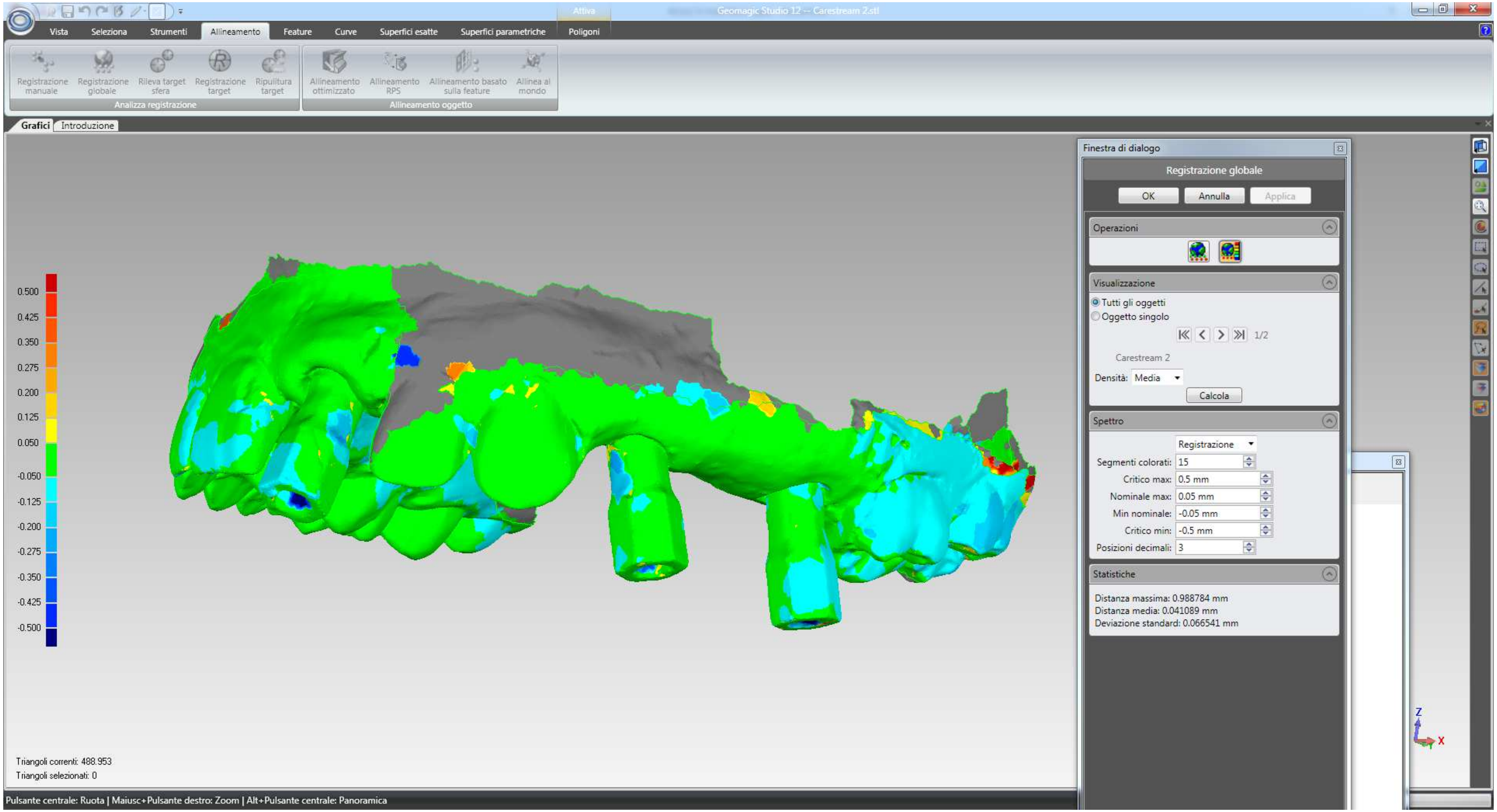

Care 2 as reference (best accuracy) vs Care 3

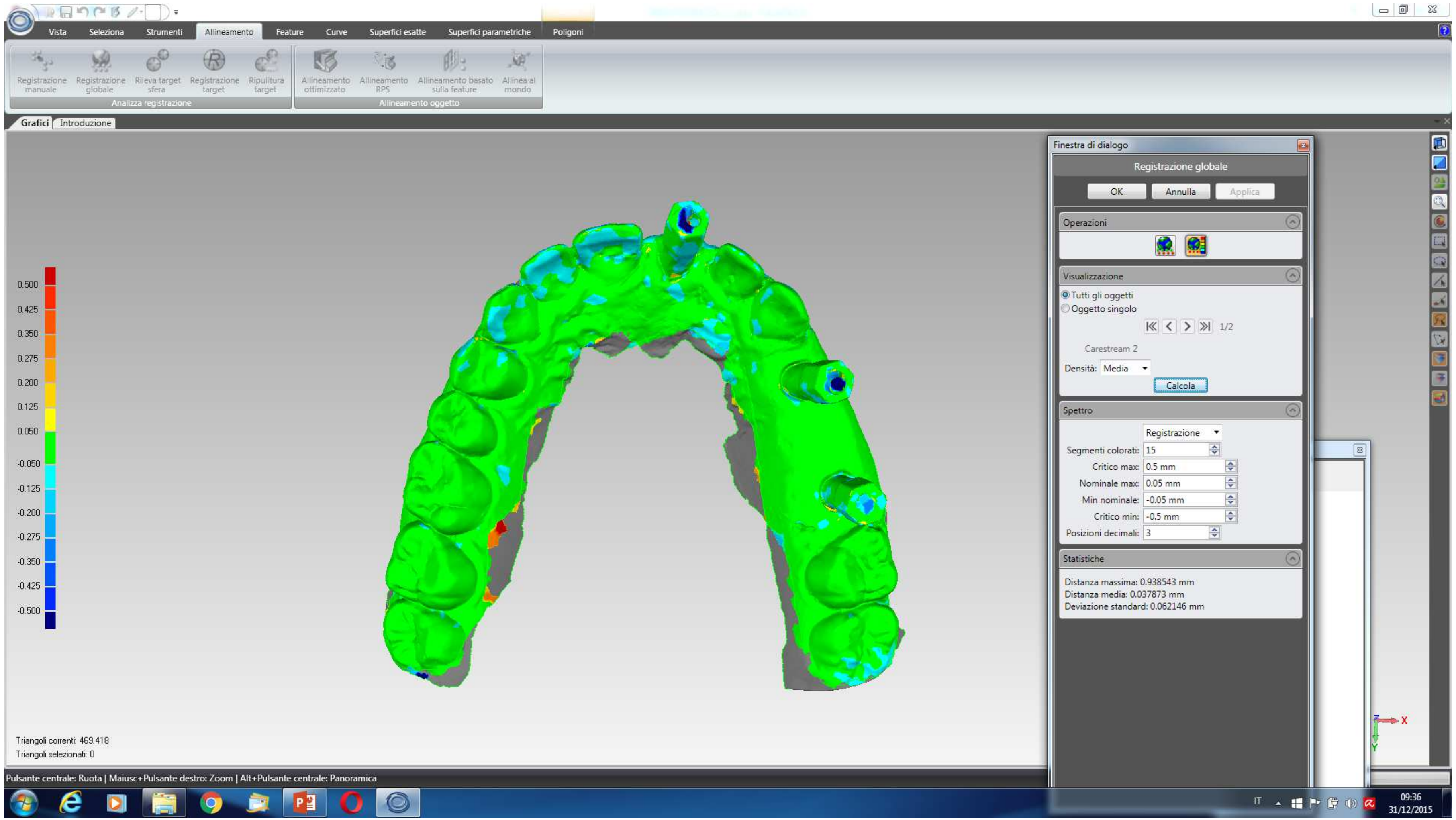

Care 2 as reference (best accuracy) vs Care 3

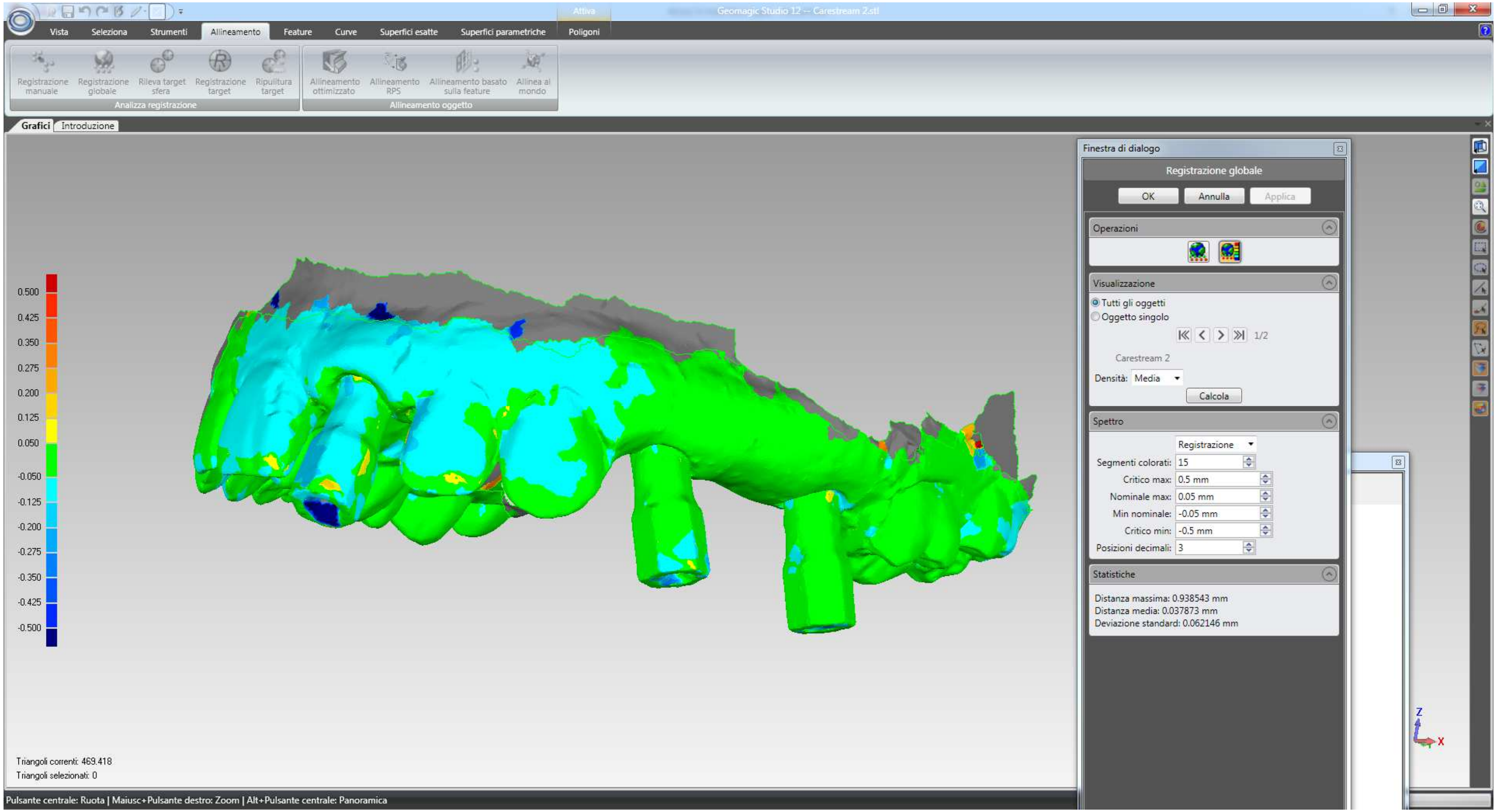

Care 2 as reference (best accuracy) vs Care 4

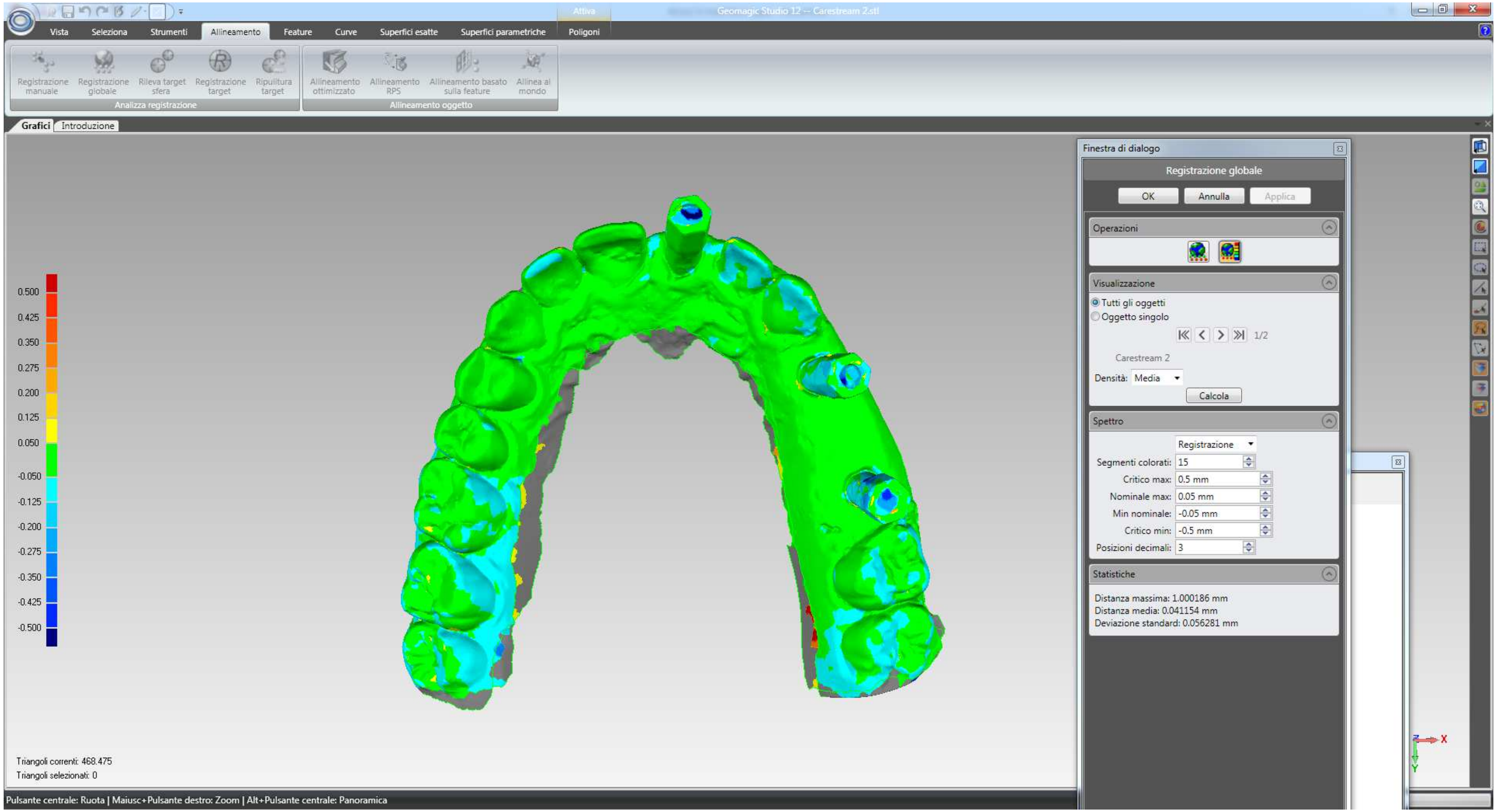

Care 2 as reference (best accuracy) vs Care 4

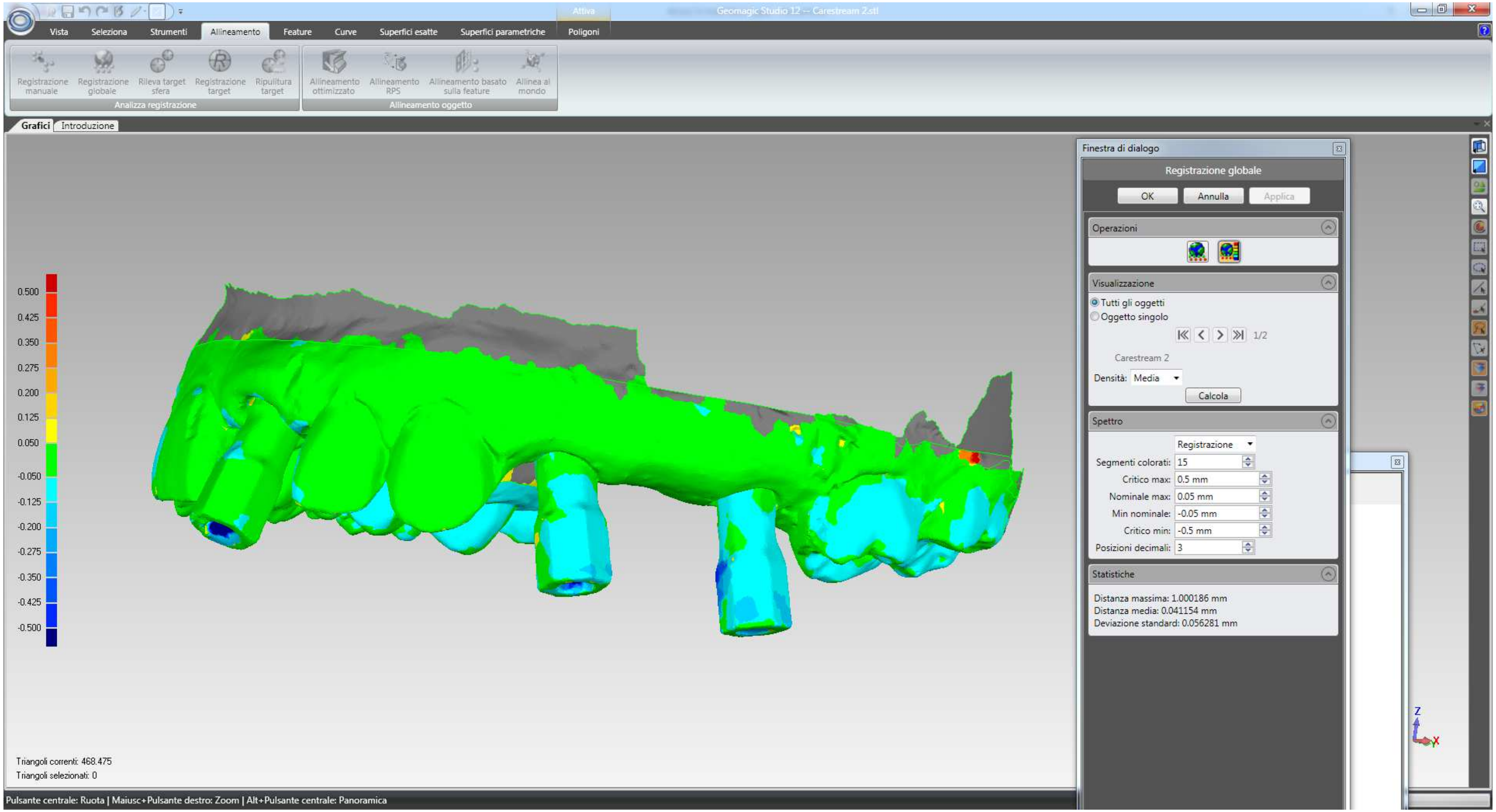

Care 2 as reference (best accuracy) vs Care 5

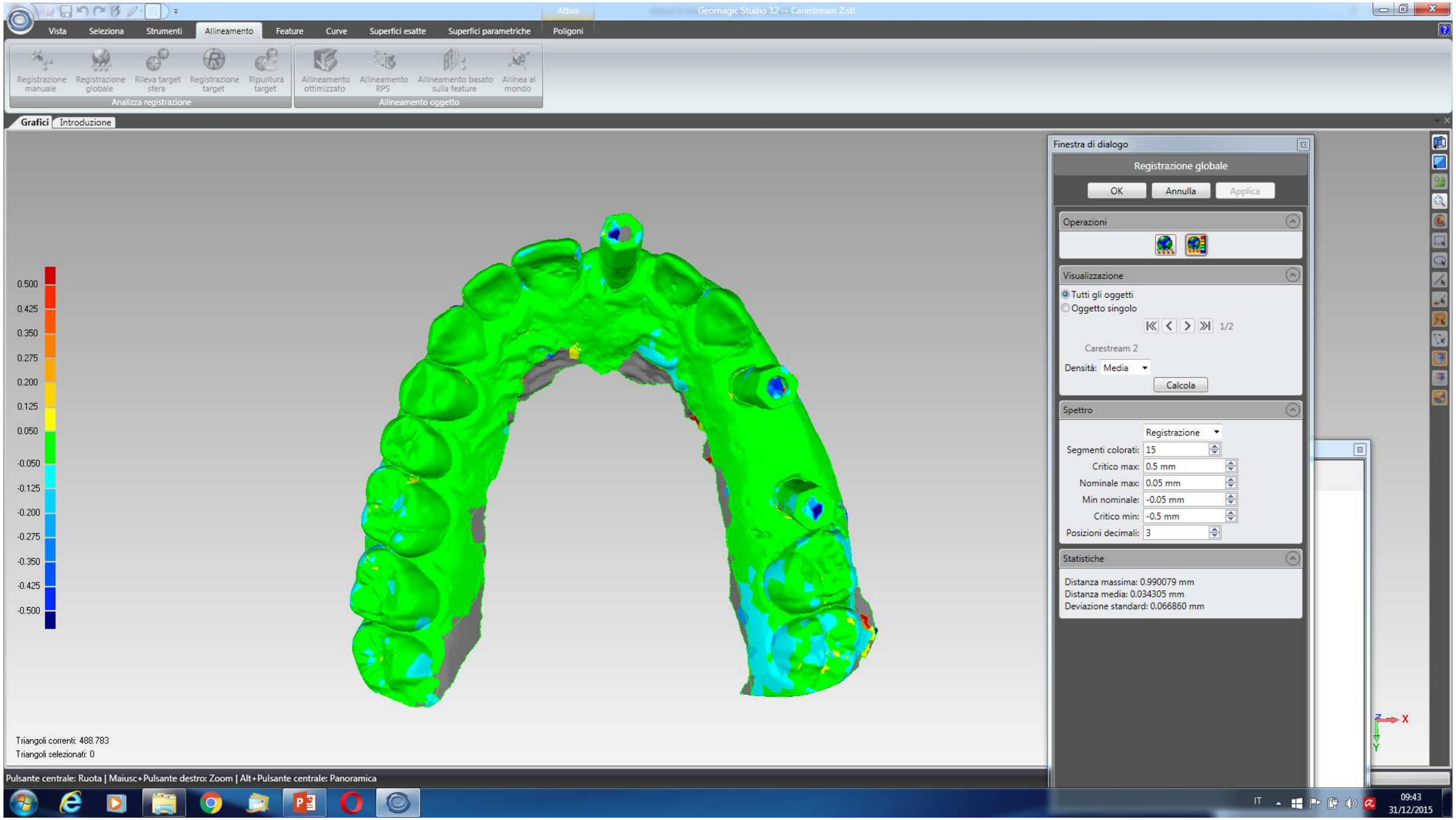

Care 2 as reference (best accuracy) vs Care 5

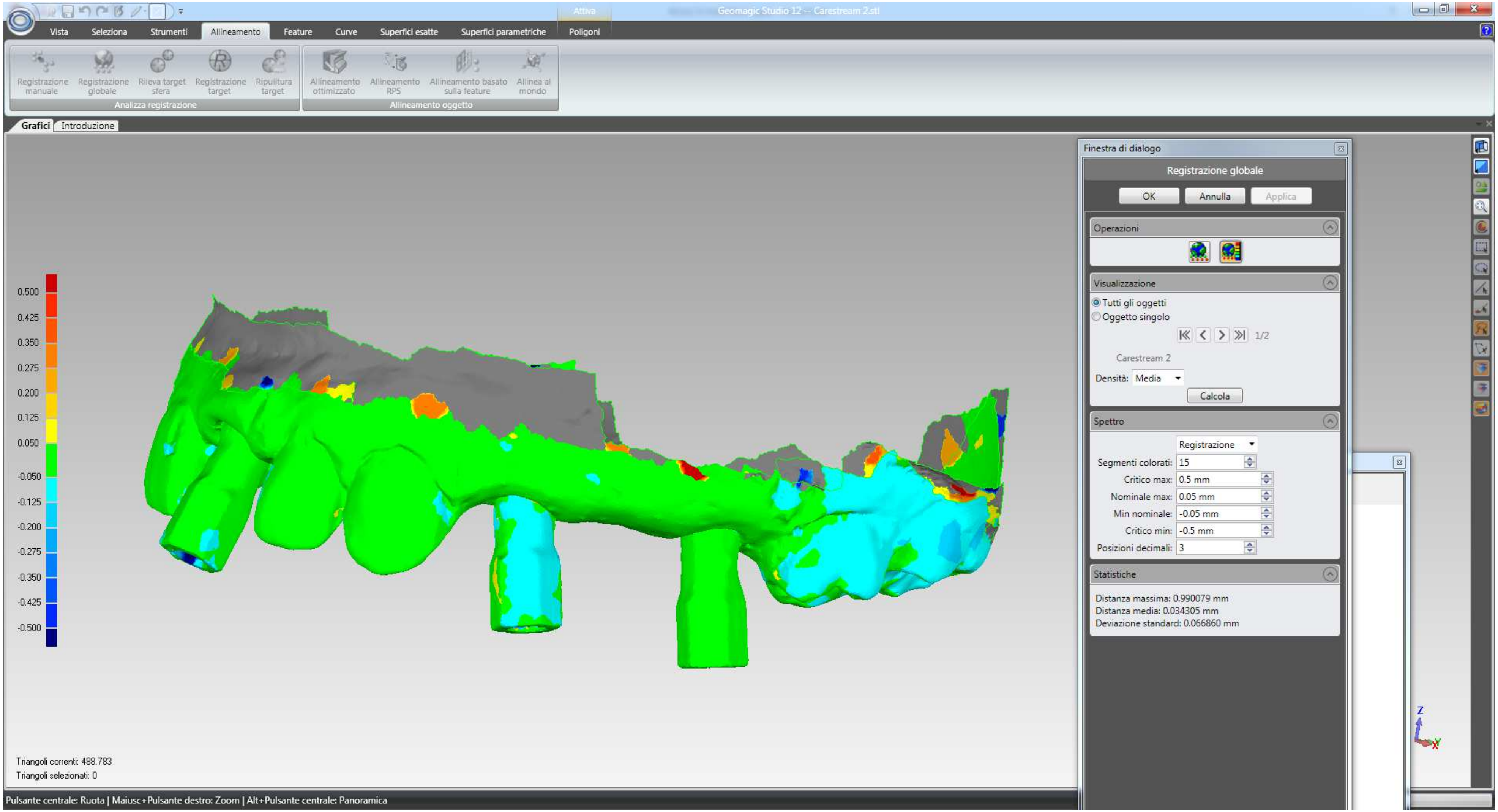

Care 1 vs Care 3

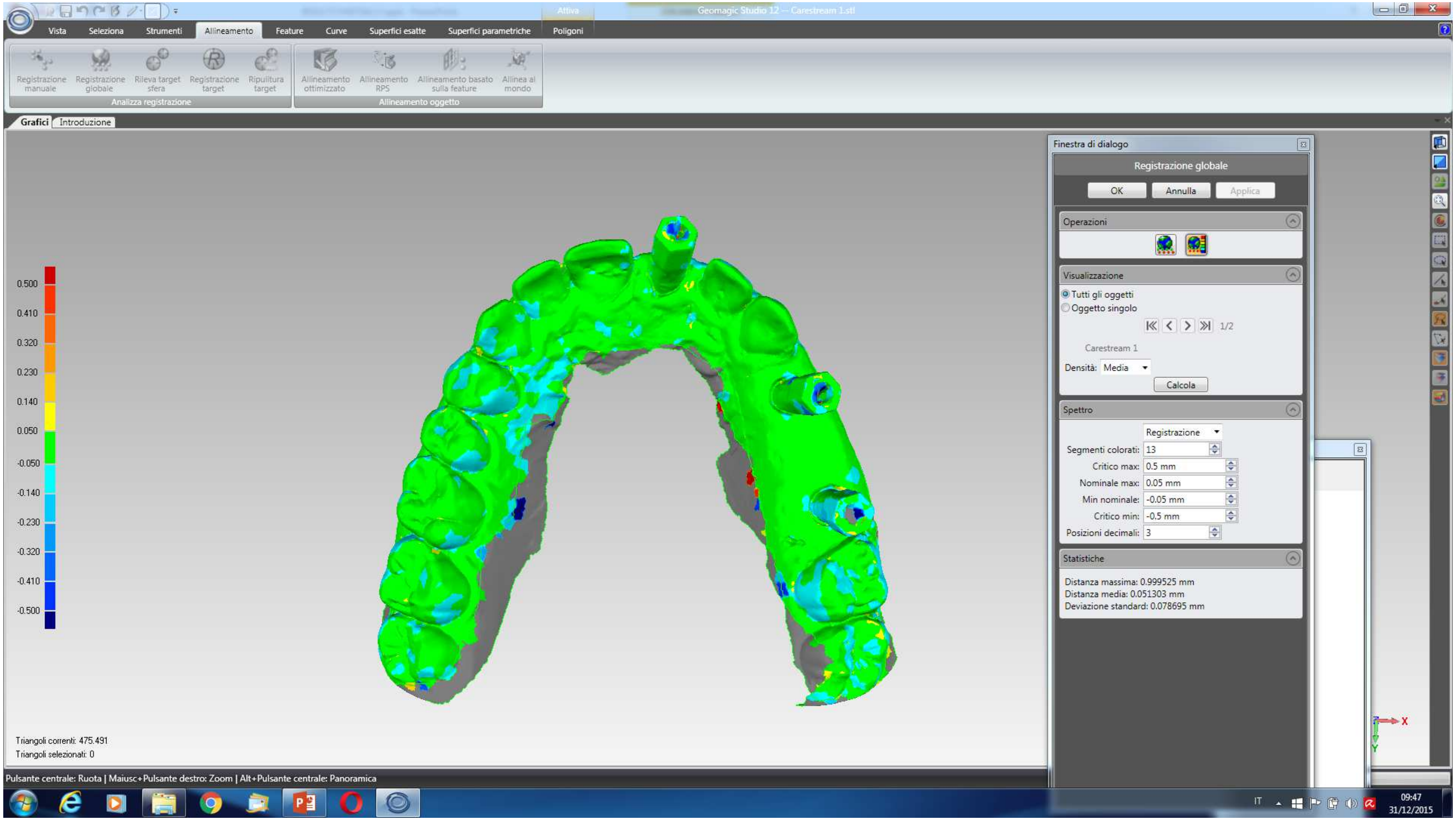

Care 1 vs Care 3

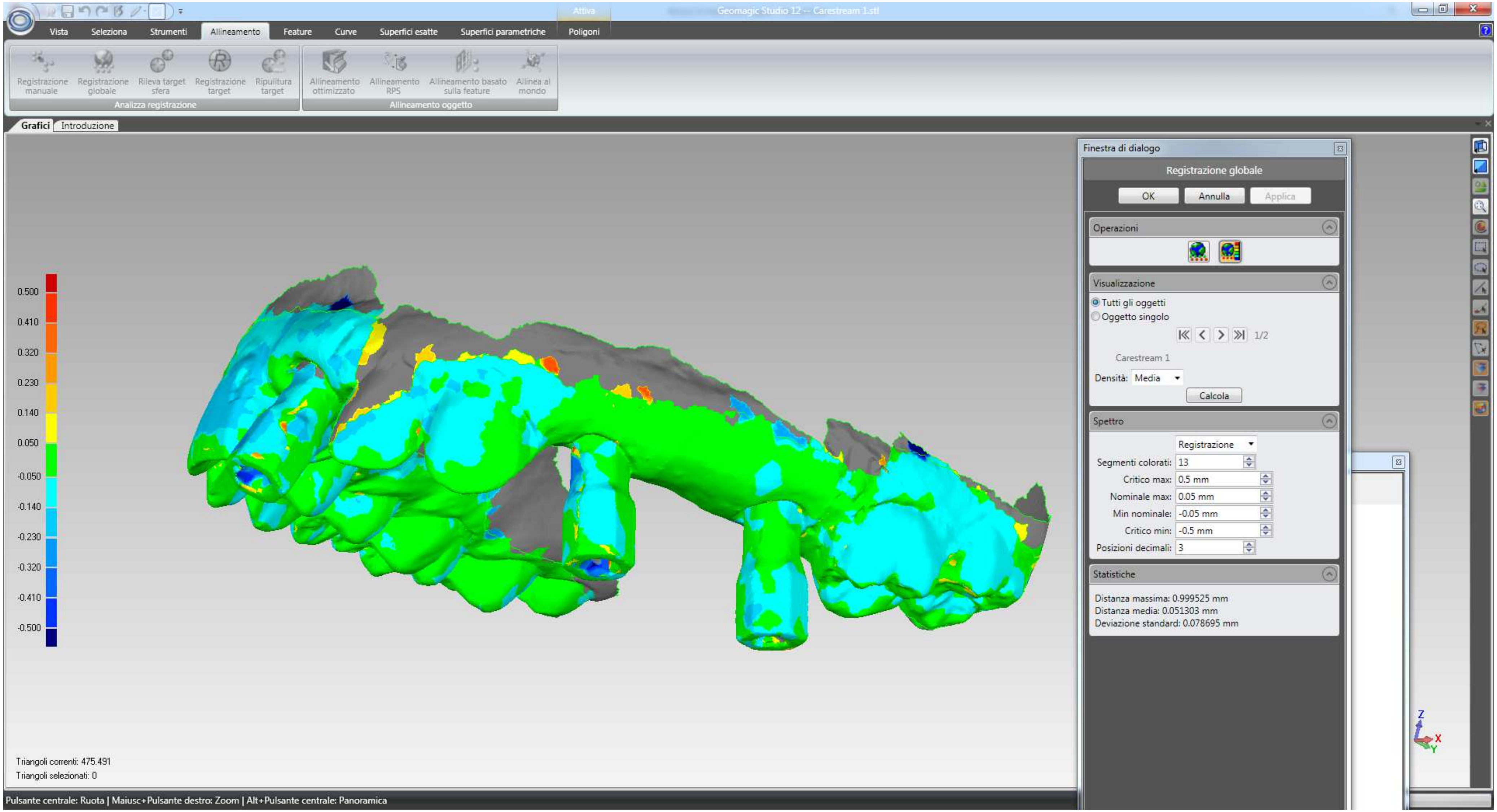

General precision Carestream in the partially edentulous model

	Mean distance	SD	Maximum distance
Care 2 vs Care 1	0.041	0.066	0.988
Care 2 vs Care 3	0.037	0.062	0.938
Care 2 vs Care 4	0.041	0.056	1.000
Care 2 vs Care 5	0.034	0.066	0.990
Care 1 vs Care 3	0.051	0.078	0.998

Overall Care general precision: 0.040 (0.006)

Plane 1 vs Plane 2

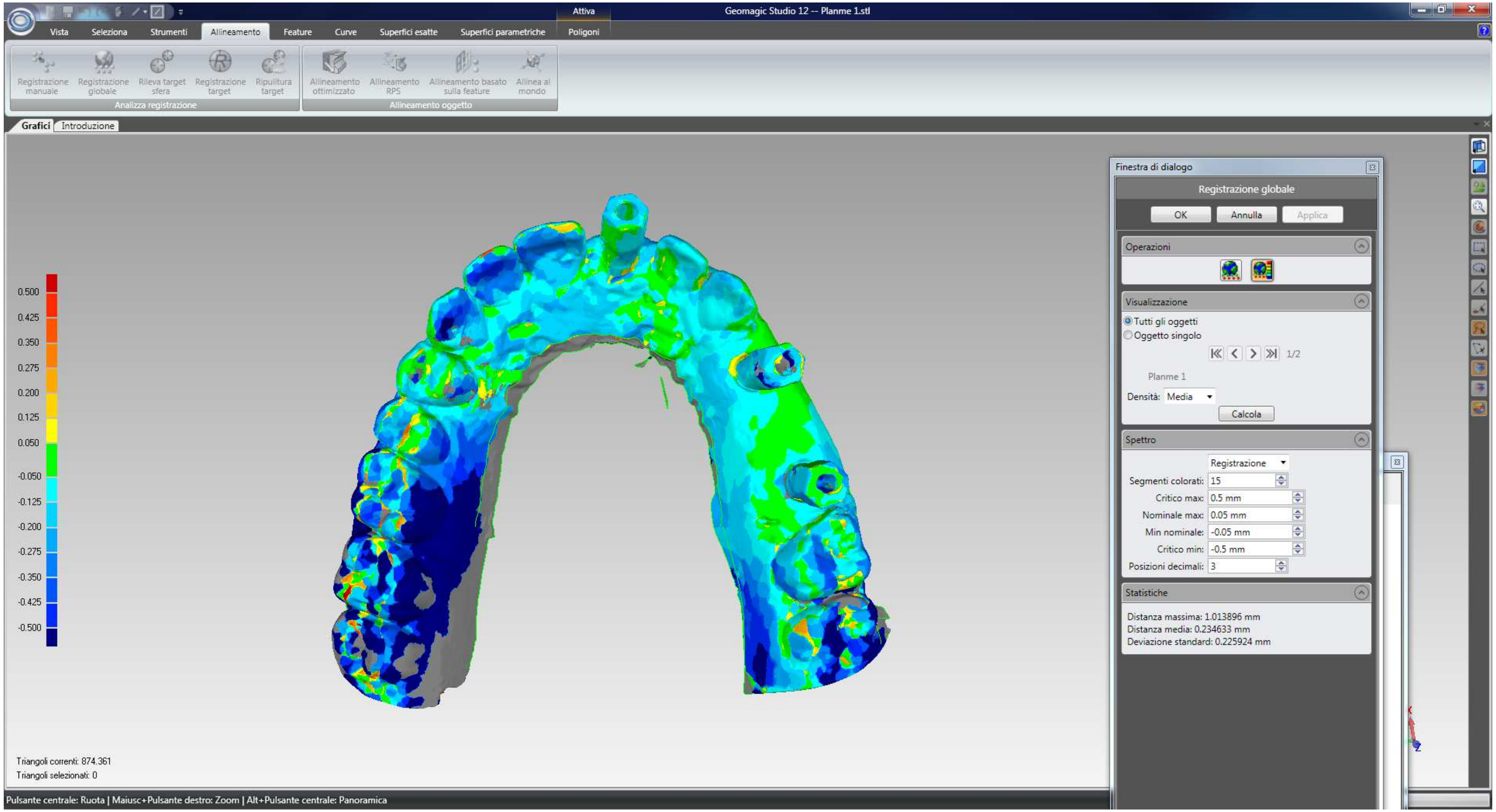

Plane 1 vs Plane 2

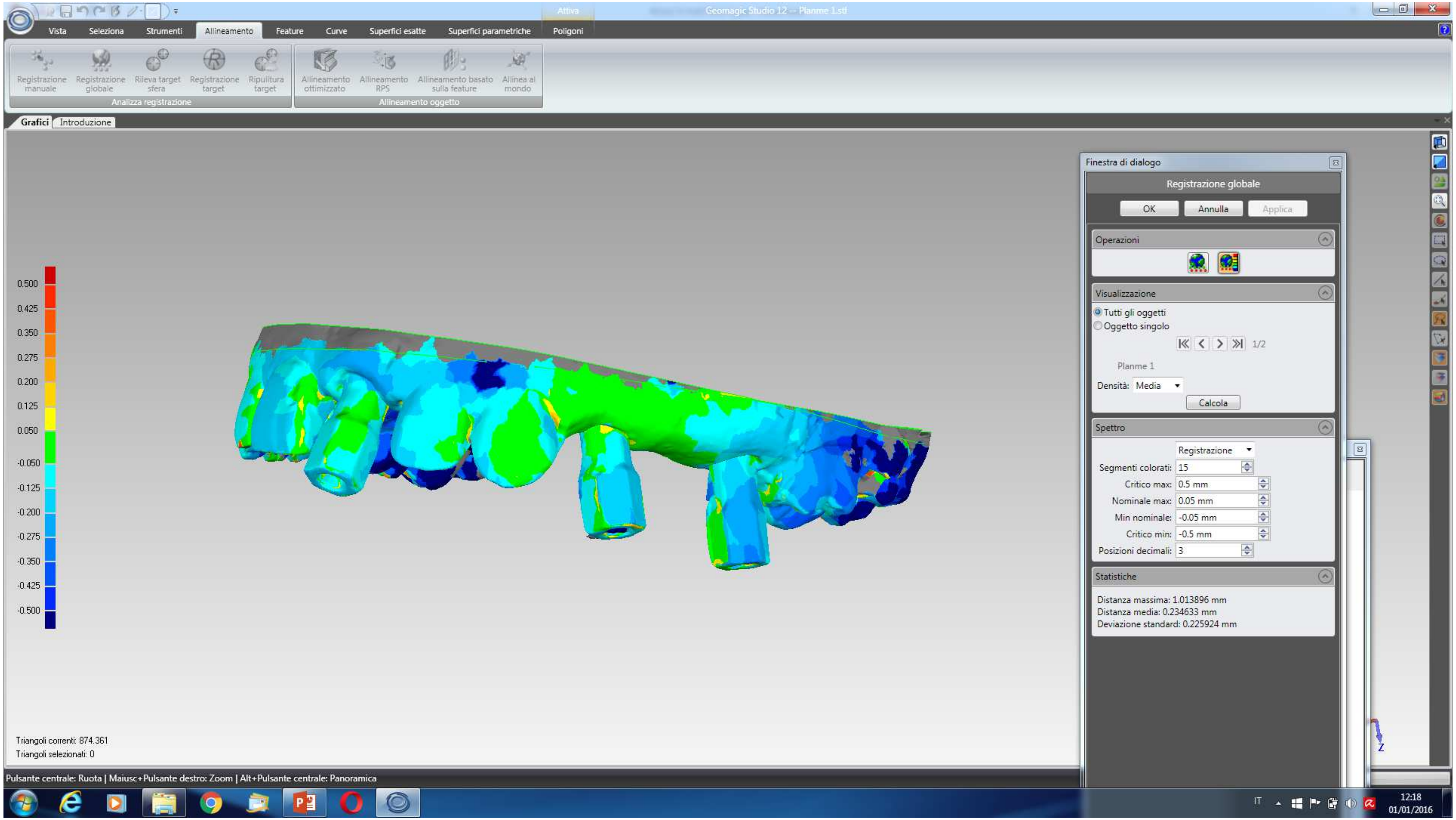

Plane 1 vs Plane 3

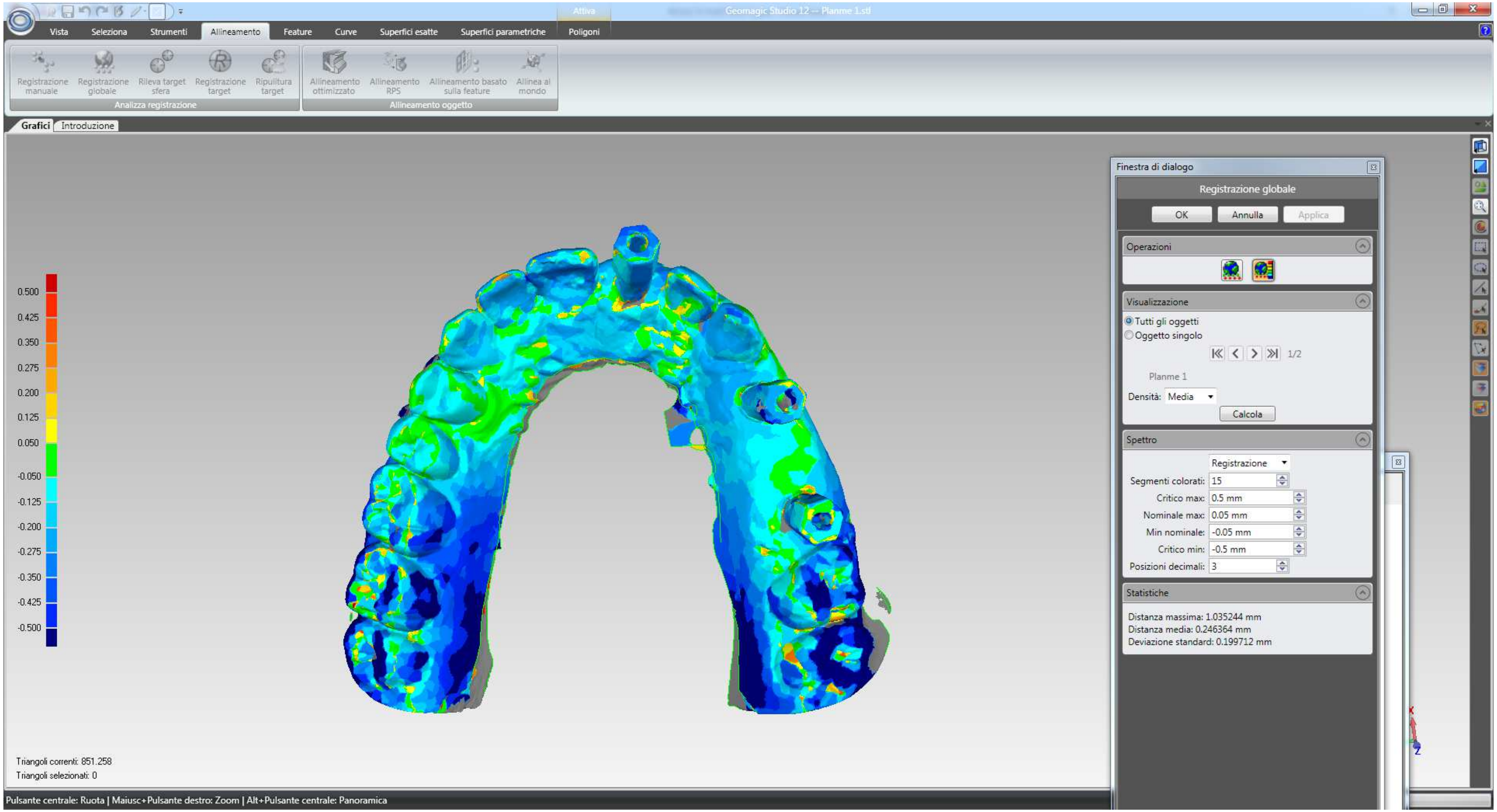

Plane 1 vs Plane 3

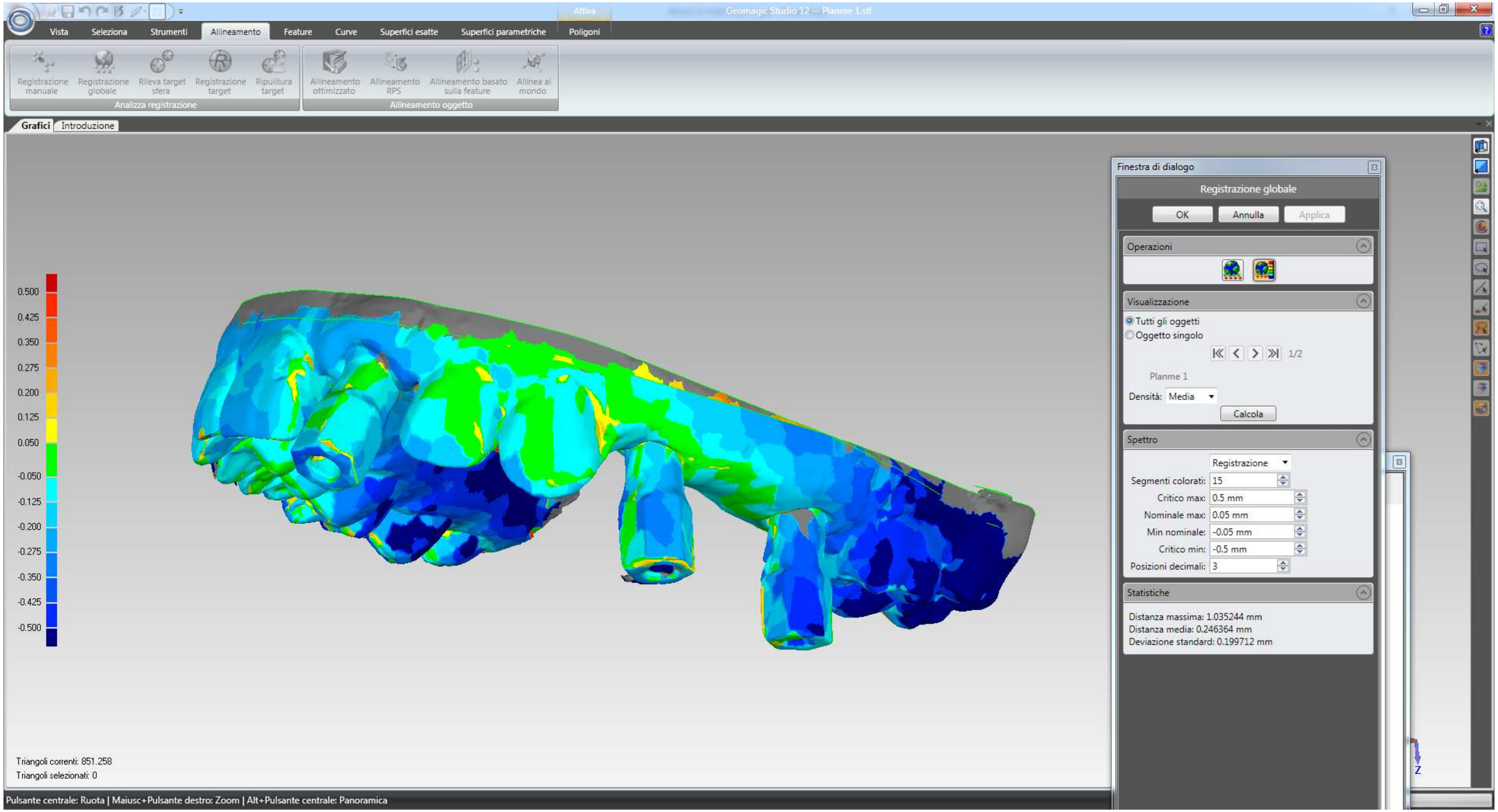

Plane 1 vs Plane 4

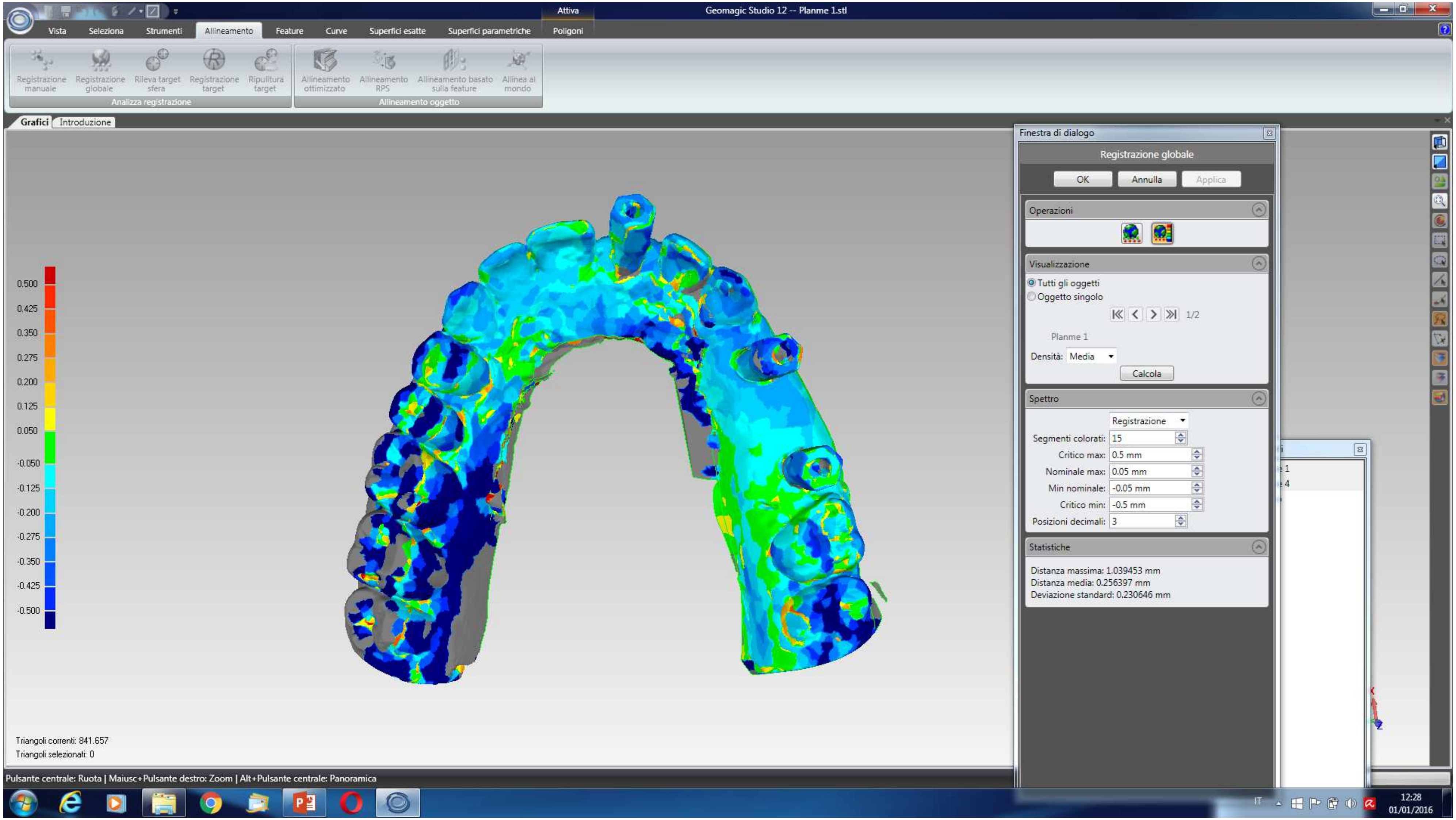

Plane 1 vs Plane 4

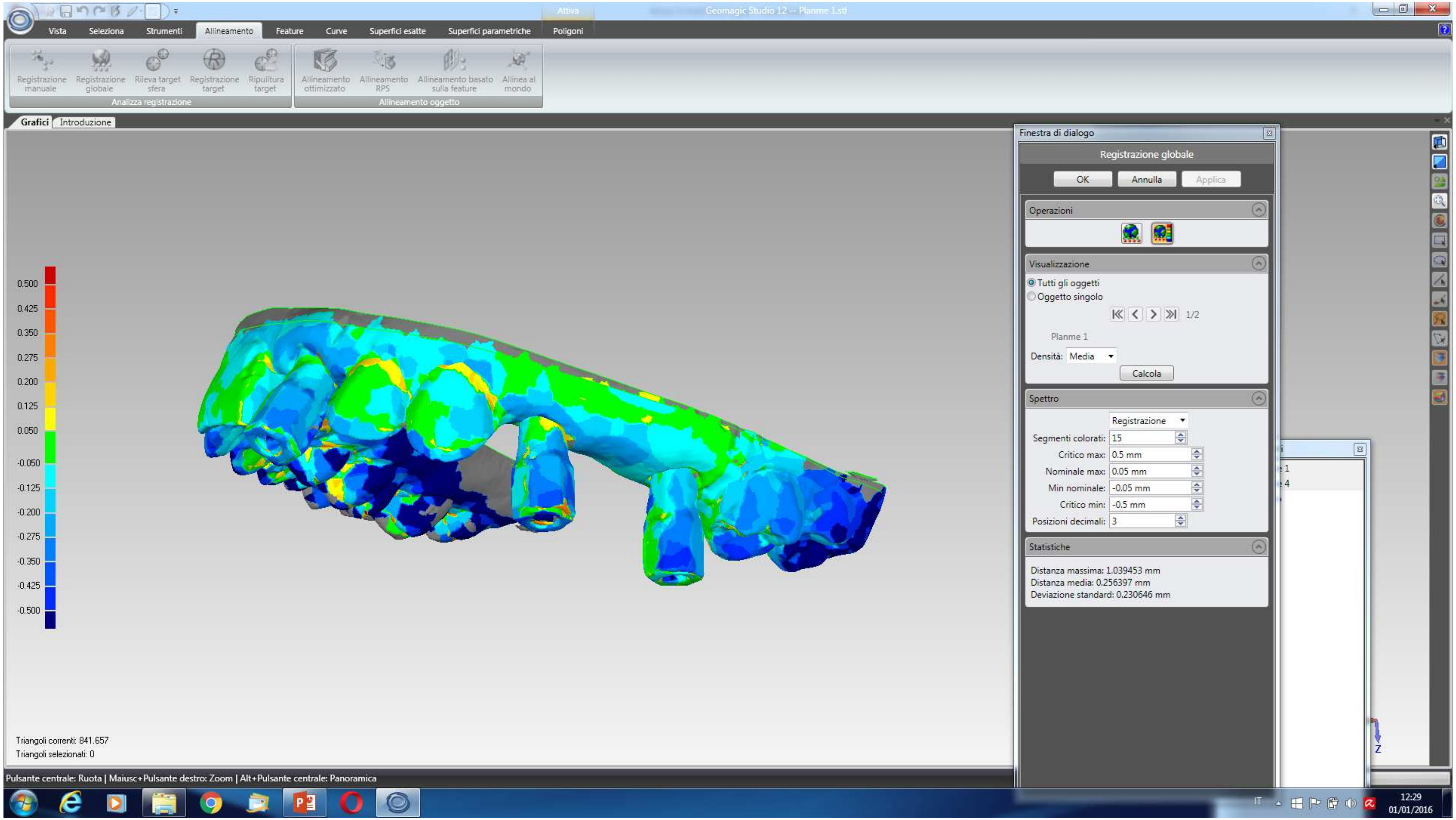

Plane 1 vs Plane 5

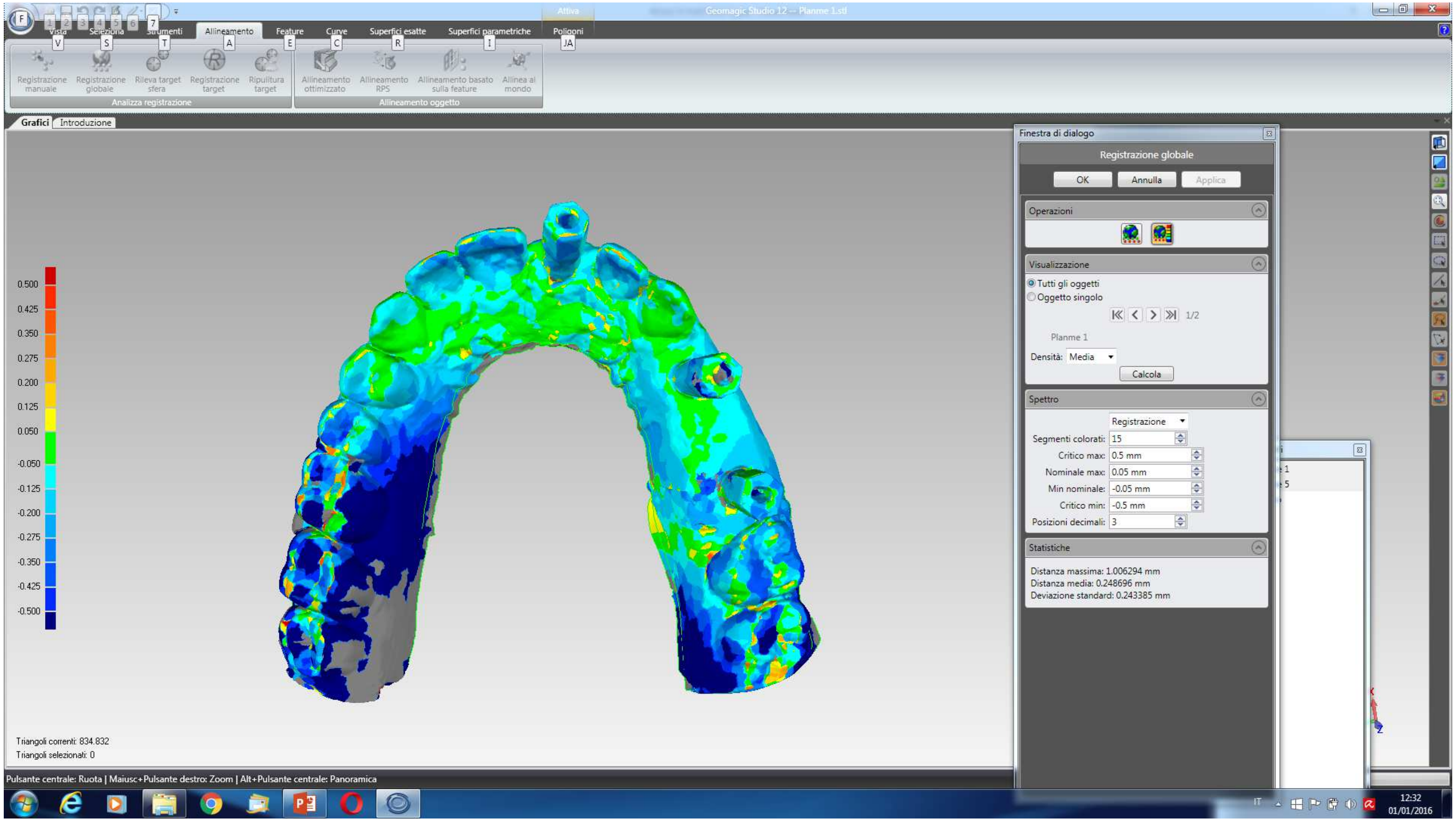

Plane 1 vs Plane 5

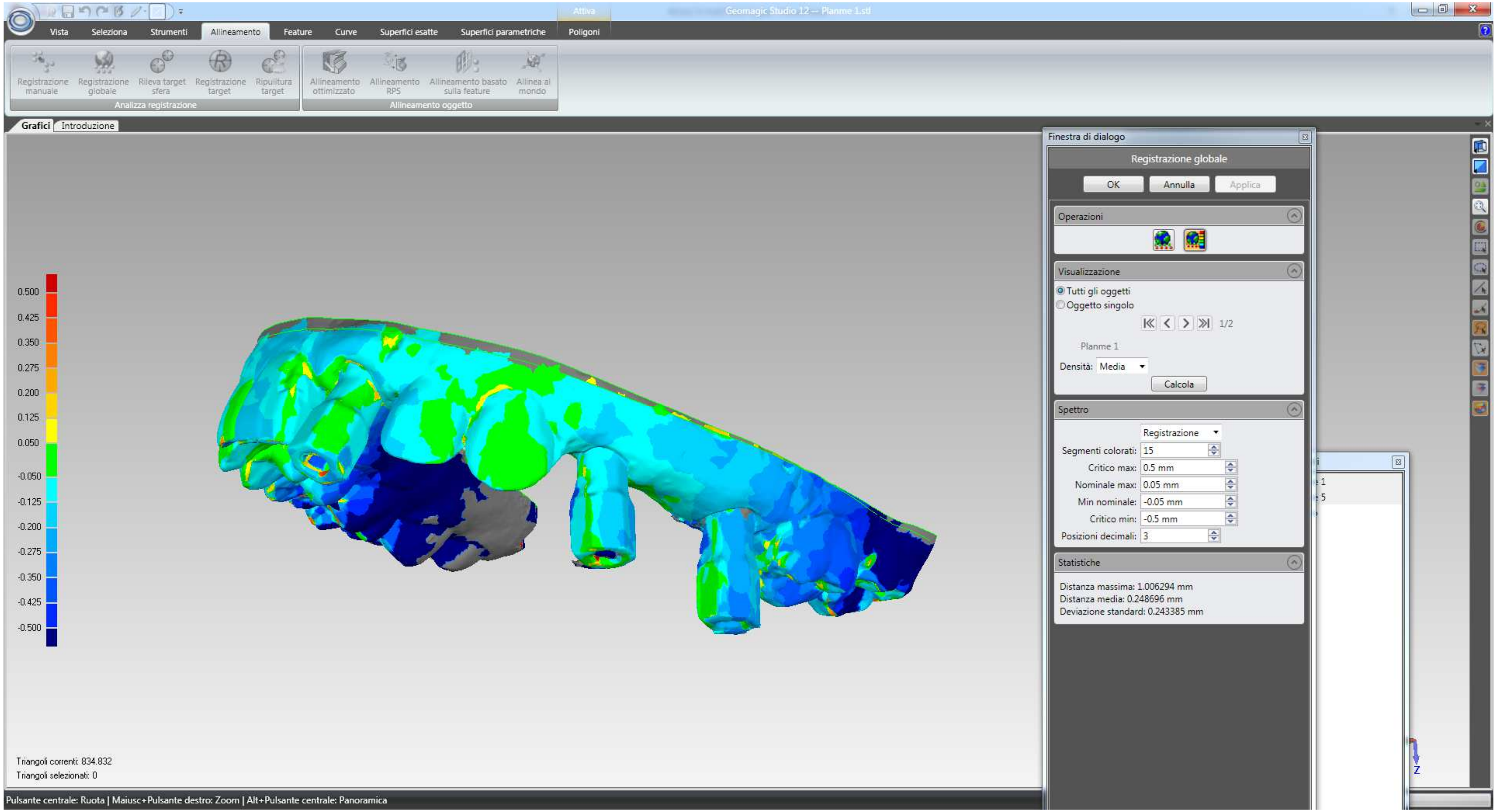

Plane 2 vs Plane 4

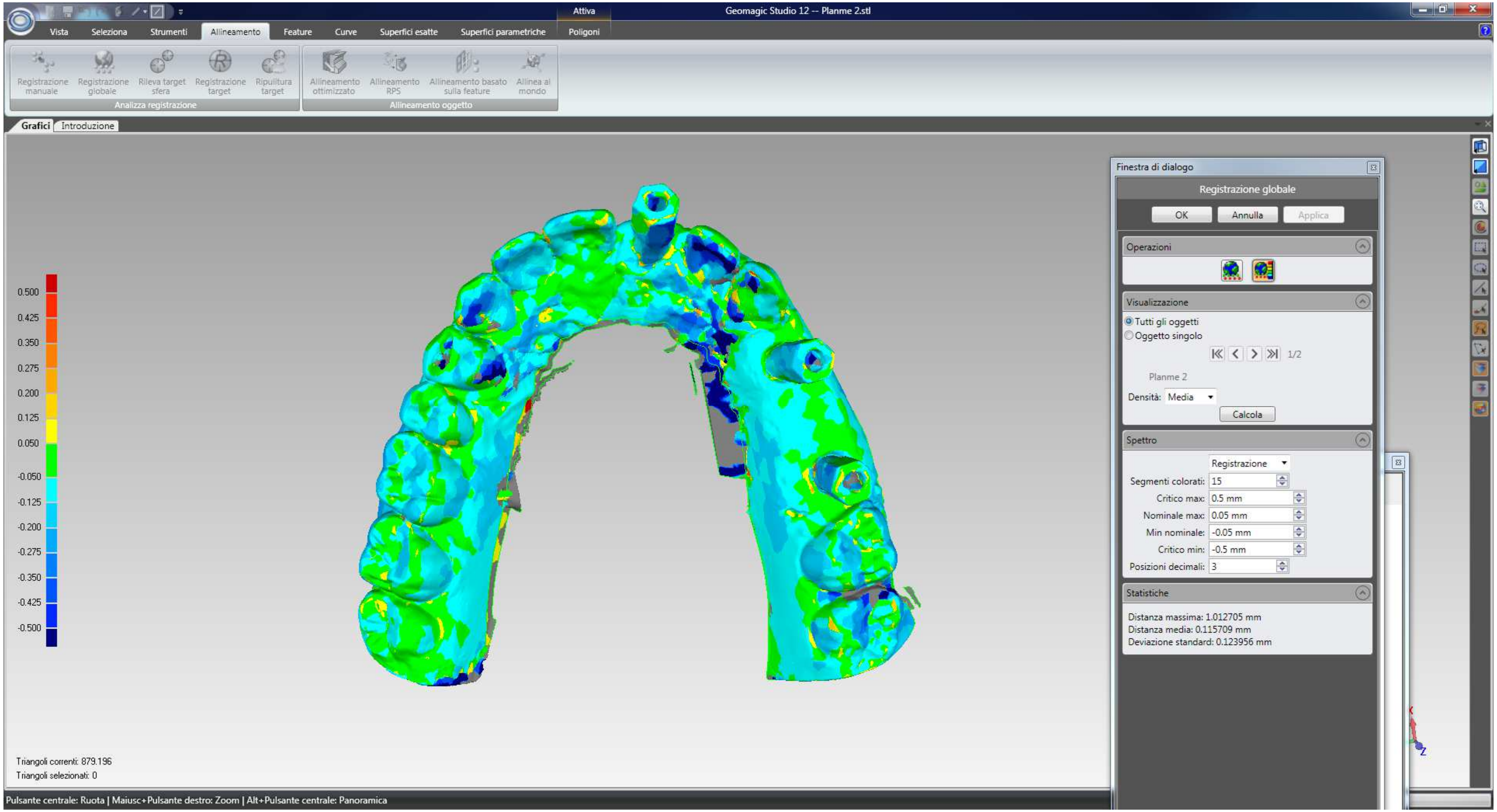

Plane 2 vs Plane 4

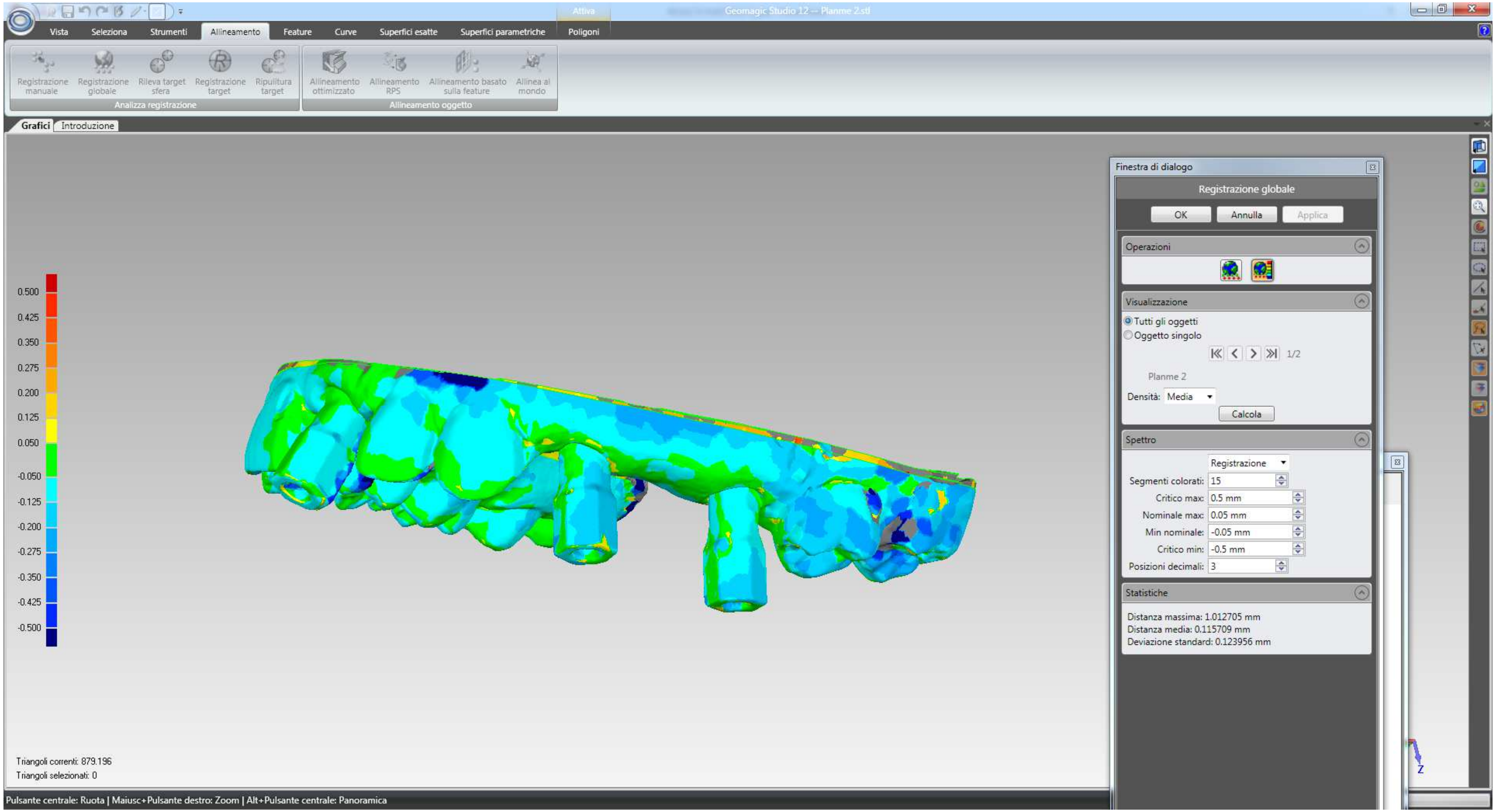

General precision Planscan scanner in the partially edentulous model

	Mean distance	SD	Maximum distance
Plan 1 vs Plan 2	0.234	0.225	1.013
Plan 1 vs Plan 3	0.246	0.199	1.035
Plan 1 vs Plan 4	0.256	0.230	1.039
Plan 1 vs Plan 5	0.248	0.243	1.006
Plan 2 vs Plan 4	0.115	0.123	1.014

Overall Plan general precision: 0.219 (0.059)

OVERALL RESULTS OF GENERAL TRUENESS AND GENERAL PRECISION FOR THE FOUR DIFFERENT SCANNERS IN THE PARTIALLY EDENTULOUS MODEL

Overall Carestream general accuracy: 0.047 (0.007)

Overall Trios general accuracy: 0.071 (0.019)

Overall Zfx general accuracy: 0.117 (0.028)

Overall Planscan general accuracy: 0.233 (0.062)

Overall Care general precision: 0.040 (0.006)

Overall Trios general precision: 0.051 (0.018)

Overall Zfx general precision: 0.126 (0.021)

Overall Planscan general precision: 0.219 (0.059)

OVERALL RESULTS OF LOCAL TRUENESS FOR THE FOUR DIFFERENT SCANNERS IN THE PARTIALLY EDENTULOUS MODEL

Mean error, local trueness, linear measurements

Misure lineari	Trios	Zfx	Care	Plan
S1-S2	- 0.004	- 0.111	0.034	0.112
S2-S3	0.061	- 0.051	0.051	0.207
S1-S3	- 0.033	- 0.155	- 0.060	0.122

Trios errore medio assoluto lineare: 0.032 (0.028)
Carestream errore medio assoluto lineare: 0.048 (0.013)
Zfx errore medio assoluto lineare: 0.105 (0.052)
Planmeca errore medio assoluto lineare: 0.147 (0.052)

Mean error, local trueness, angular measurements

	Trios	Zfx	Care	Plan
S1-S2-S3	- 0.33°	0.82°	0.09	-0.8°

Carestream errore medio assoluto angolare: 0.09°
Trios errore medio assoluto angolare: 0.33°
Planmeca errore medio assoluto angolare: 0.8°
Zfx errore medio assoluto angolare: 0.82°

GENERAL TRUENESS EVALUATIONS FOR TRIOS, ZFX INTRASCAN, CARESTREAM, PLANSCAN (SCANS WERE RANDOM BUT HERE WE ORDERED THEM) IN THE TOTALLY EDENTULOUS MODEL

GENERAL SETTINGS FOR FINAL REGISTRATION (TRUENESS):

0.5 mm, 0.05 mm, -0.05 mm, -0.5 mm (see scale)

TRIOS 1 vs REF (IMETRIC)

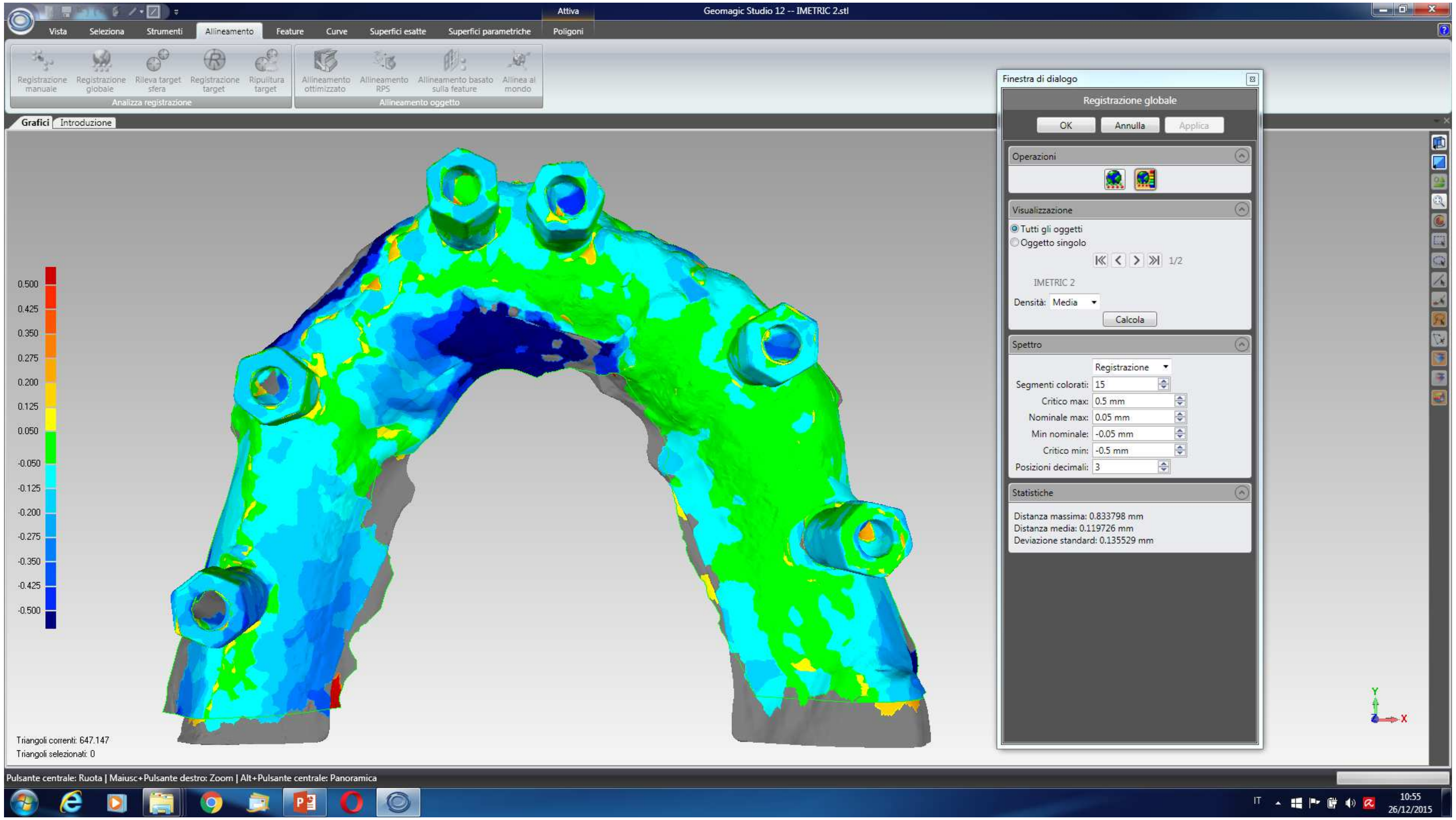

TRIOS 1 vs REF (IMETRIC)

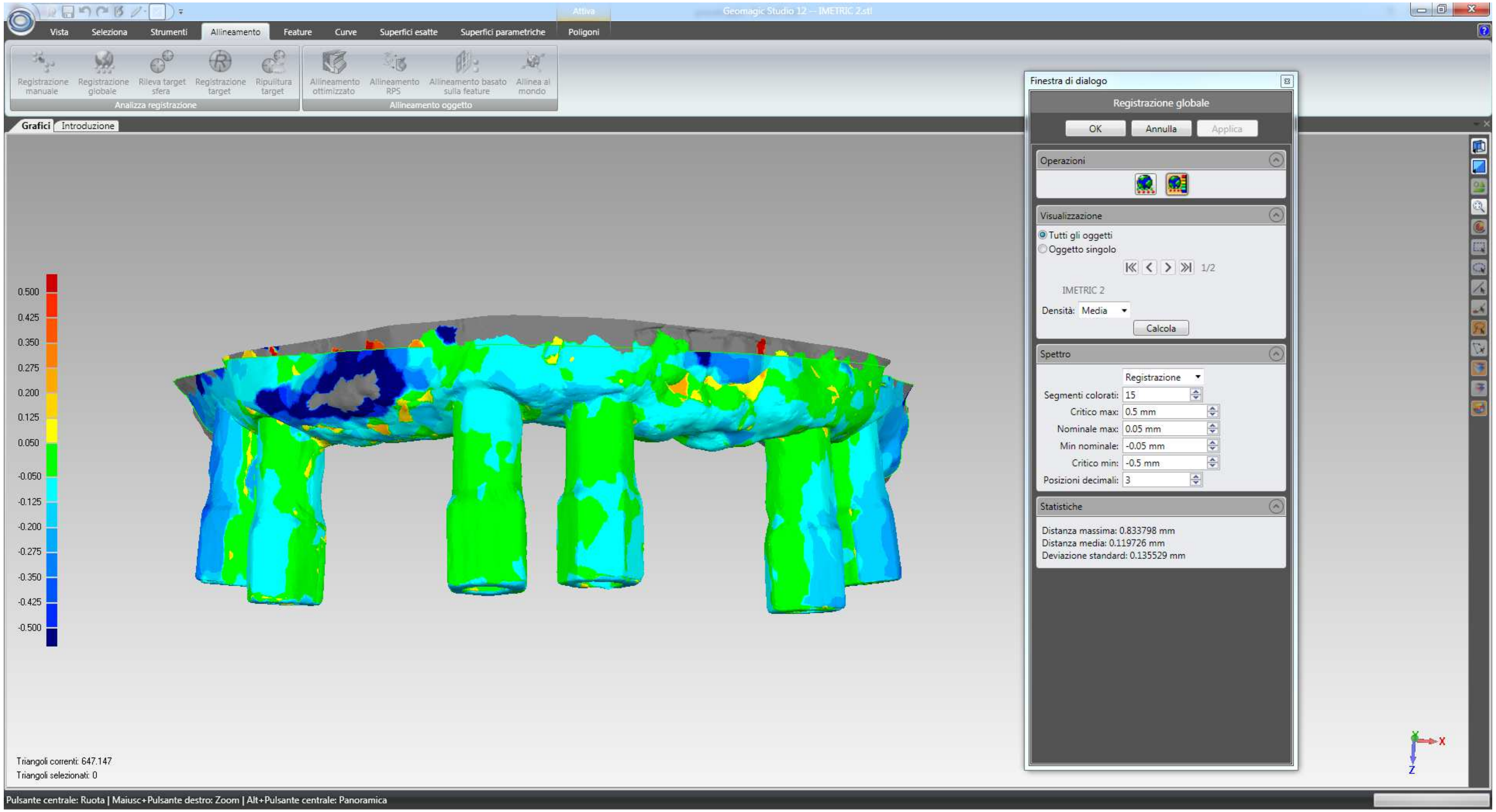

TRIOS 1 vs REF (IMETRIC)

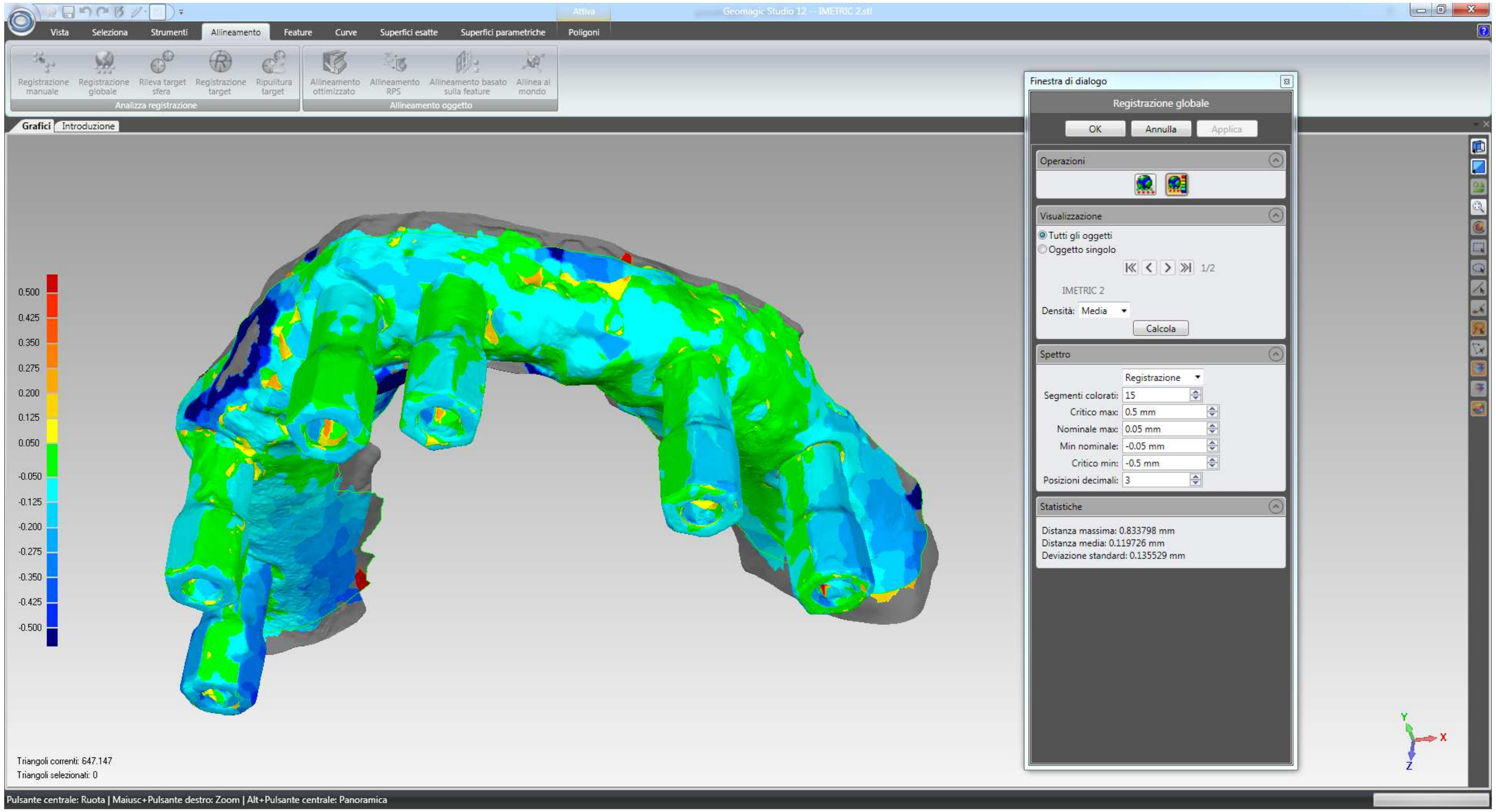

TRIOS 1 vs REF (IMETRIC)

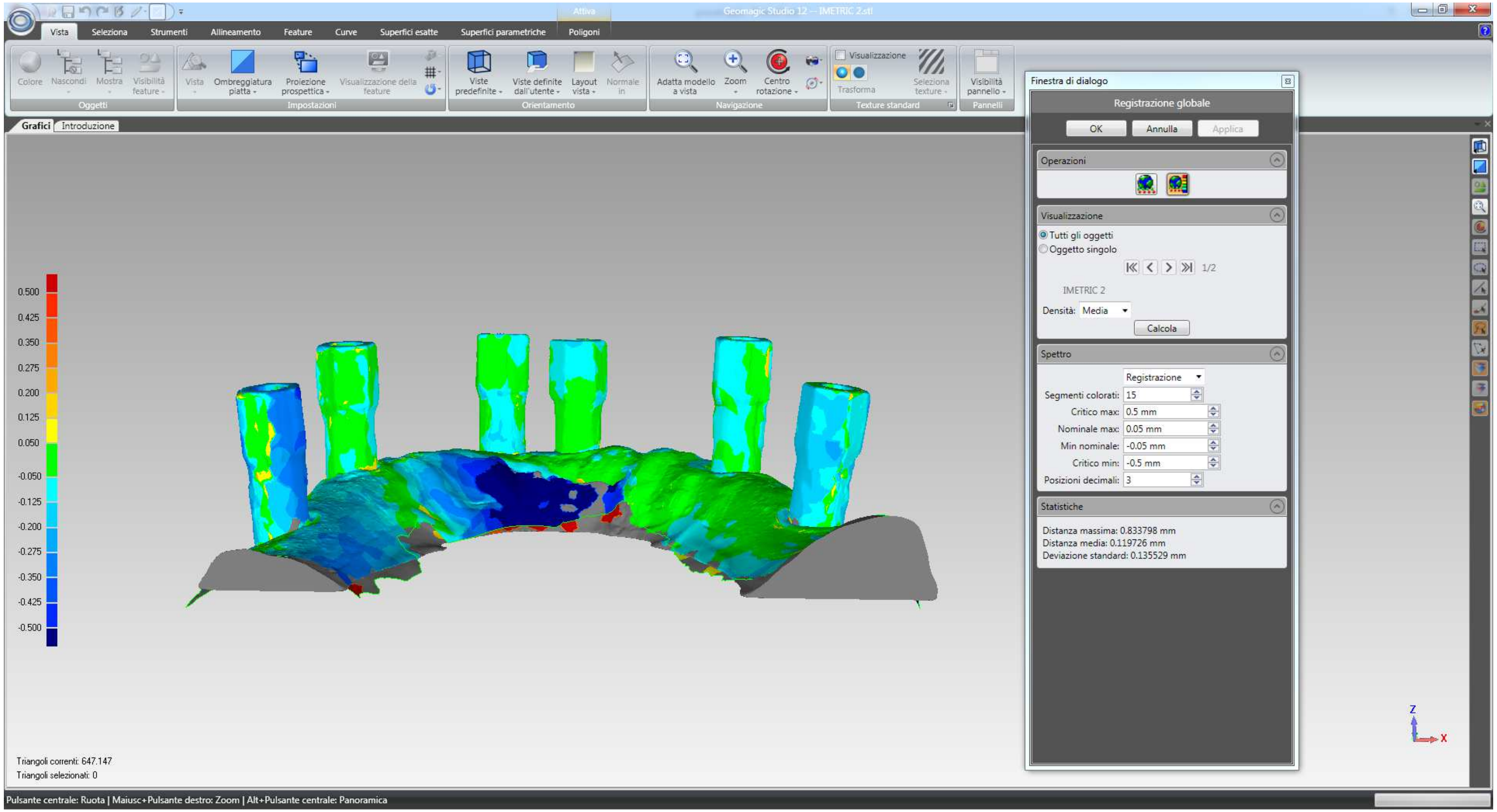

TRIOS 2 vs REF (IMETRIC)

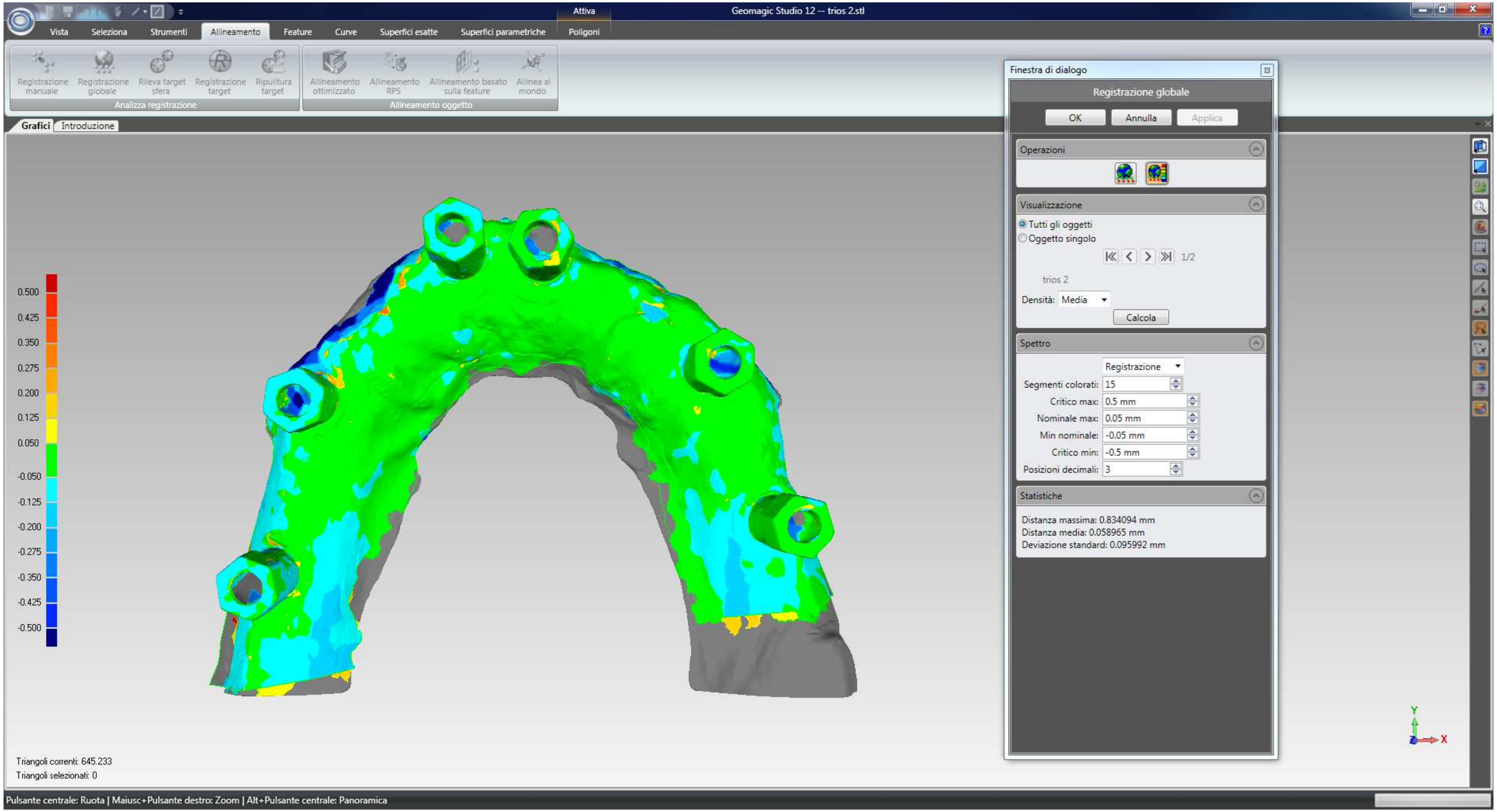

TRIOS 2 vs REF (IMETRIC)

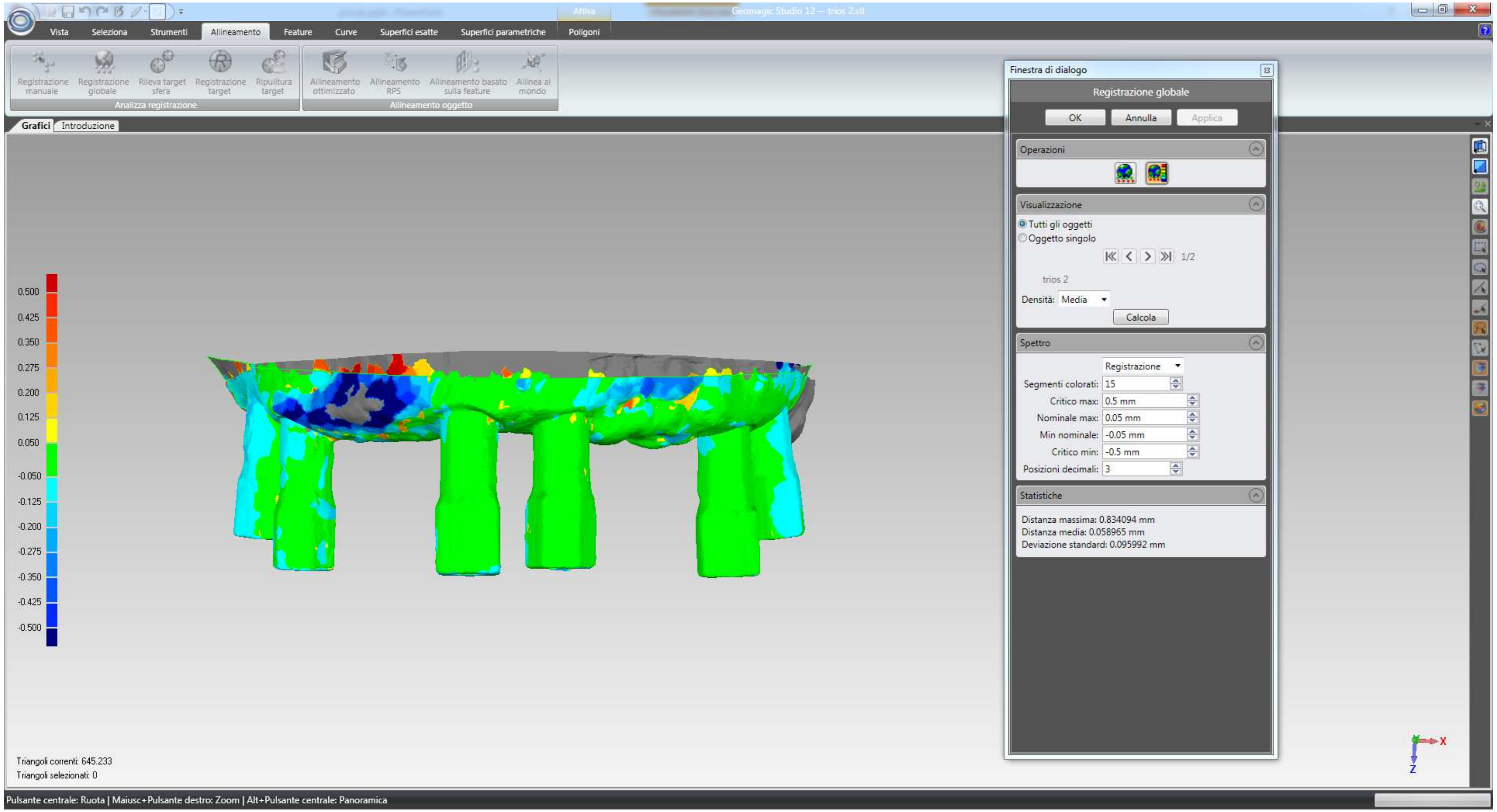

TRIOS 2 vs REF (IMETRIC)

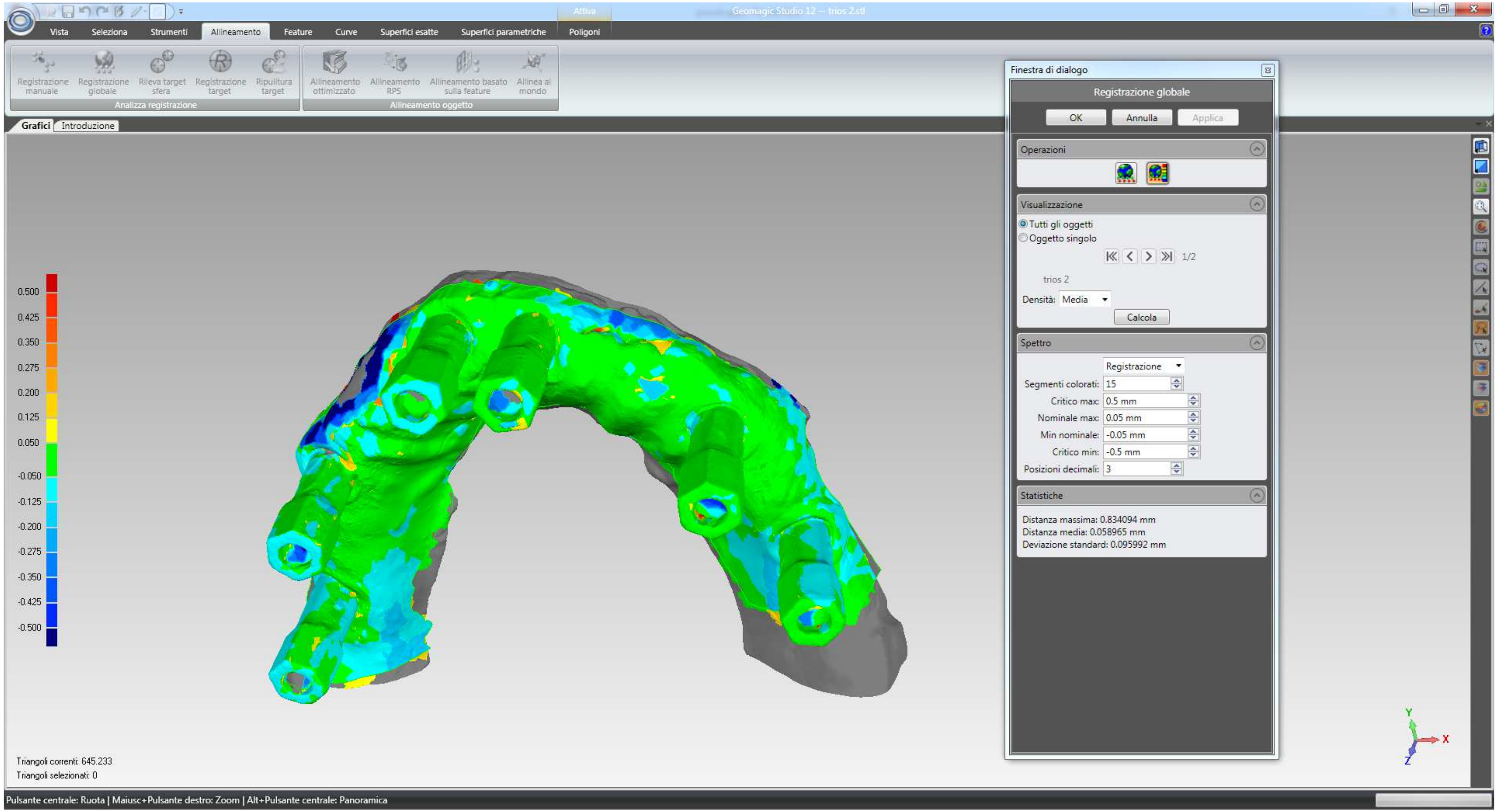

TRIOS 2 vs REF (IMETRIC)

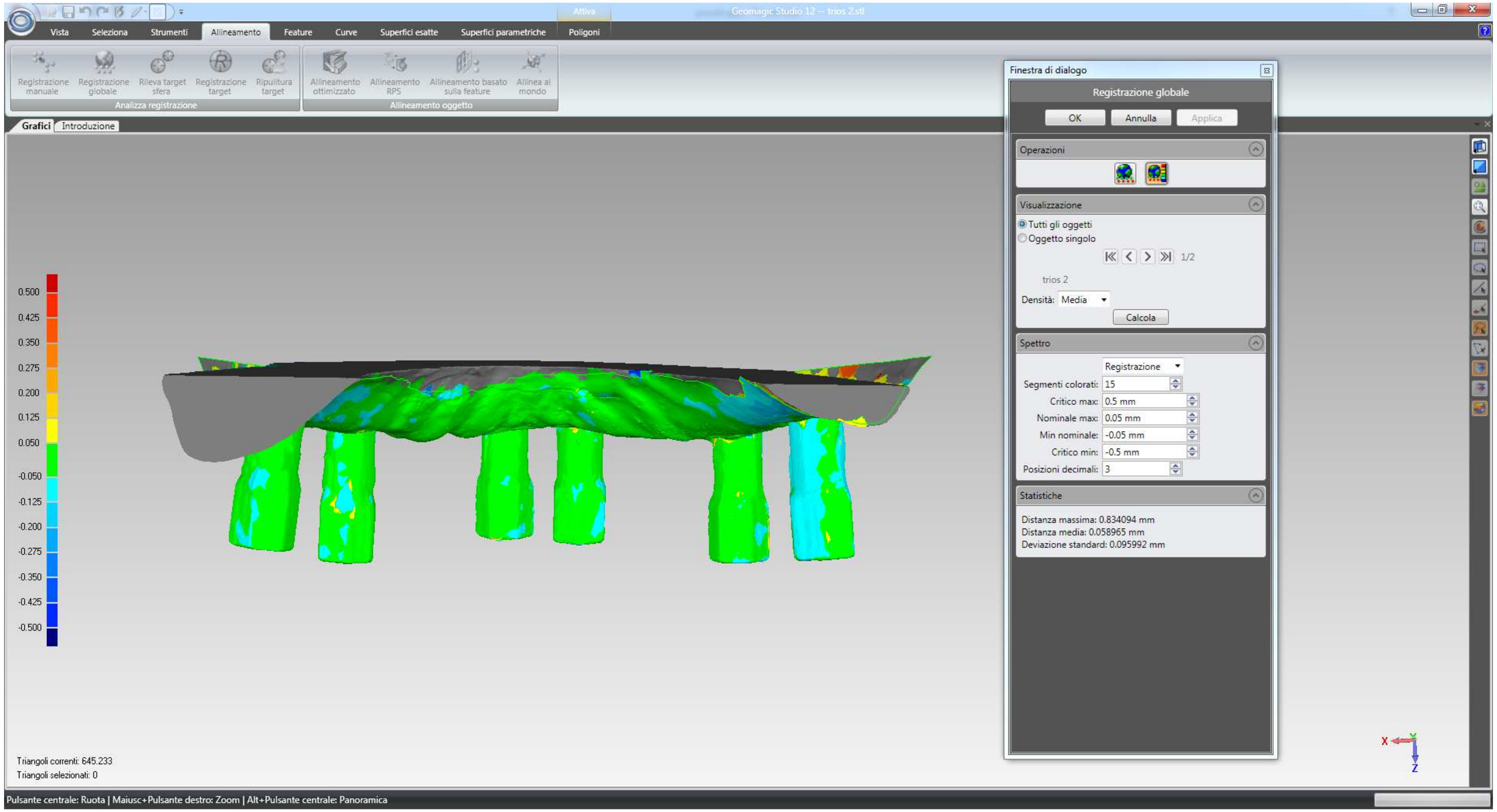

TRIOS 3 vs REF (IMETRIC)

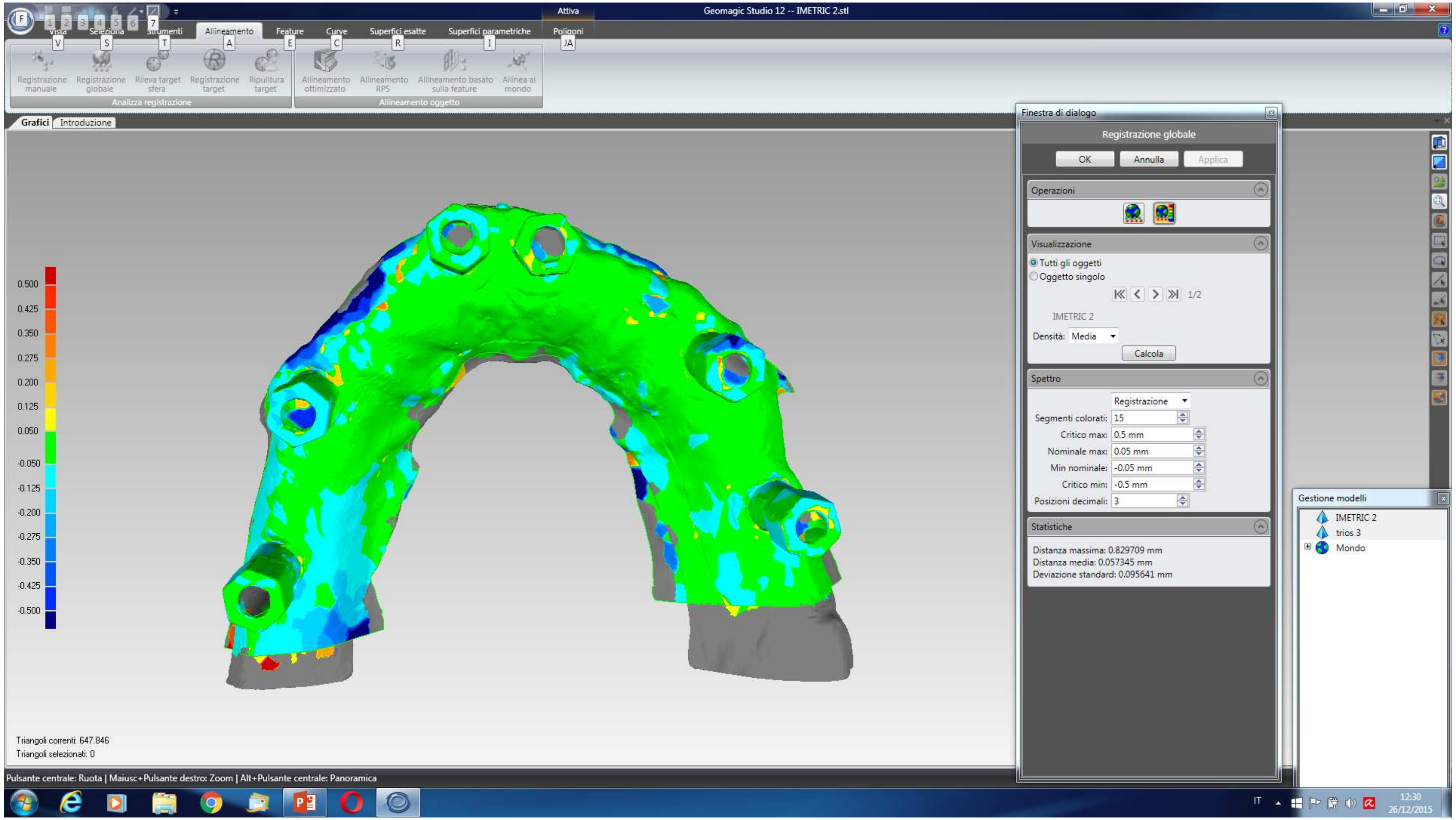

TRIOS 3 vs REF (IMETRIC)

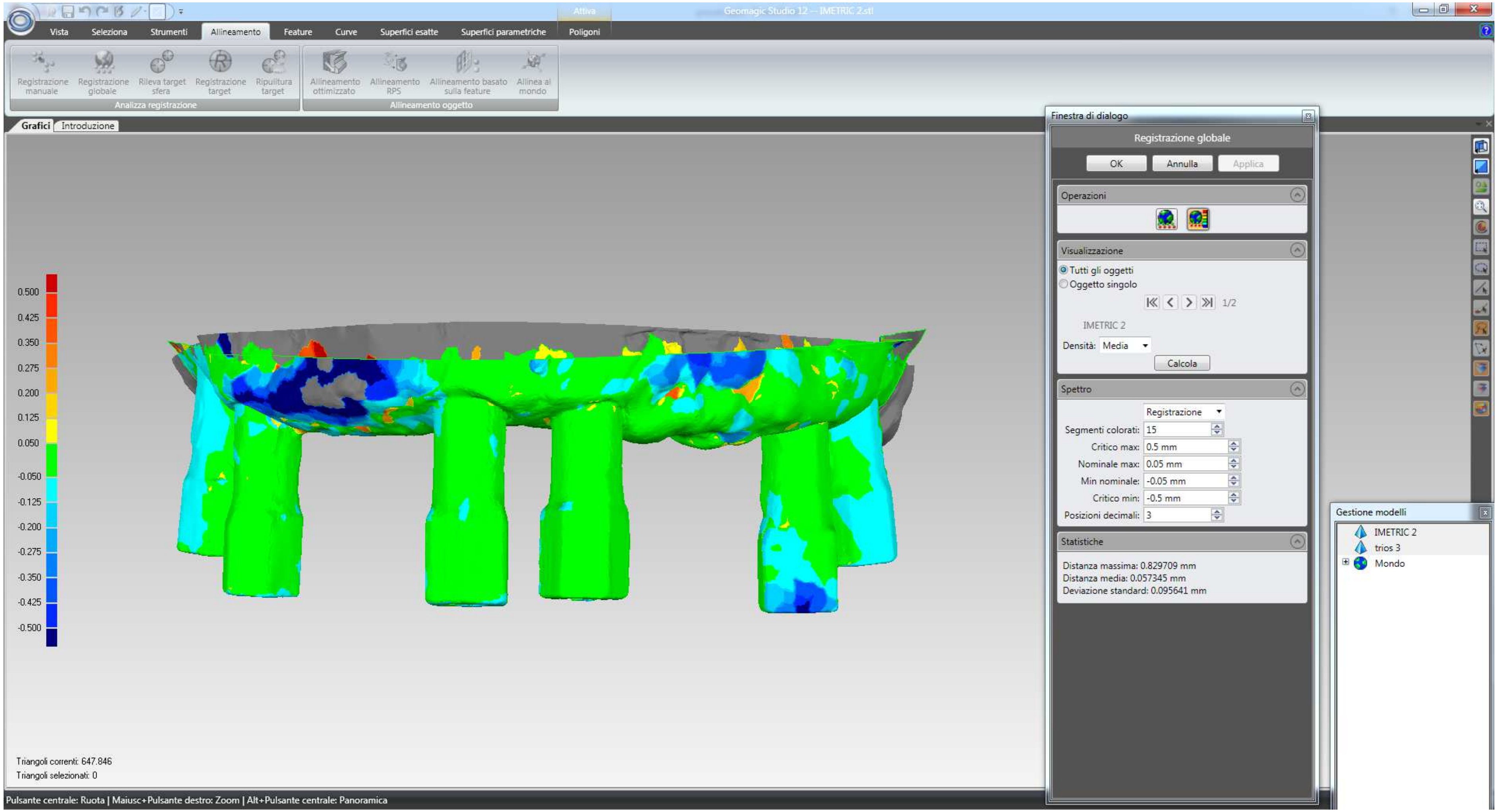

TRIOS 3 vs REF (IMETRIC)

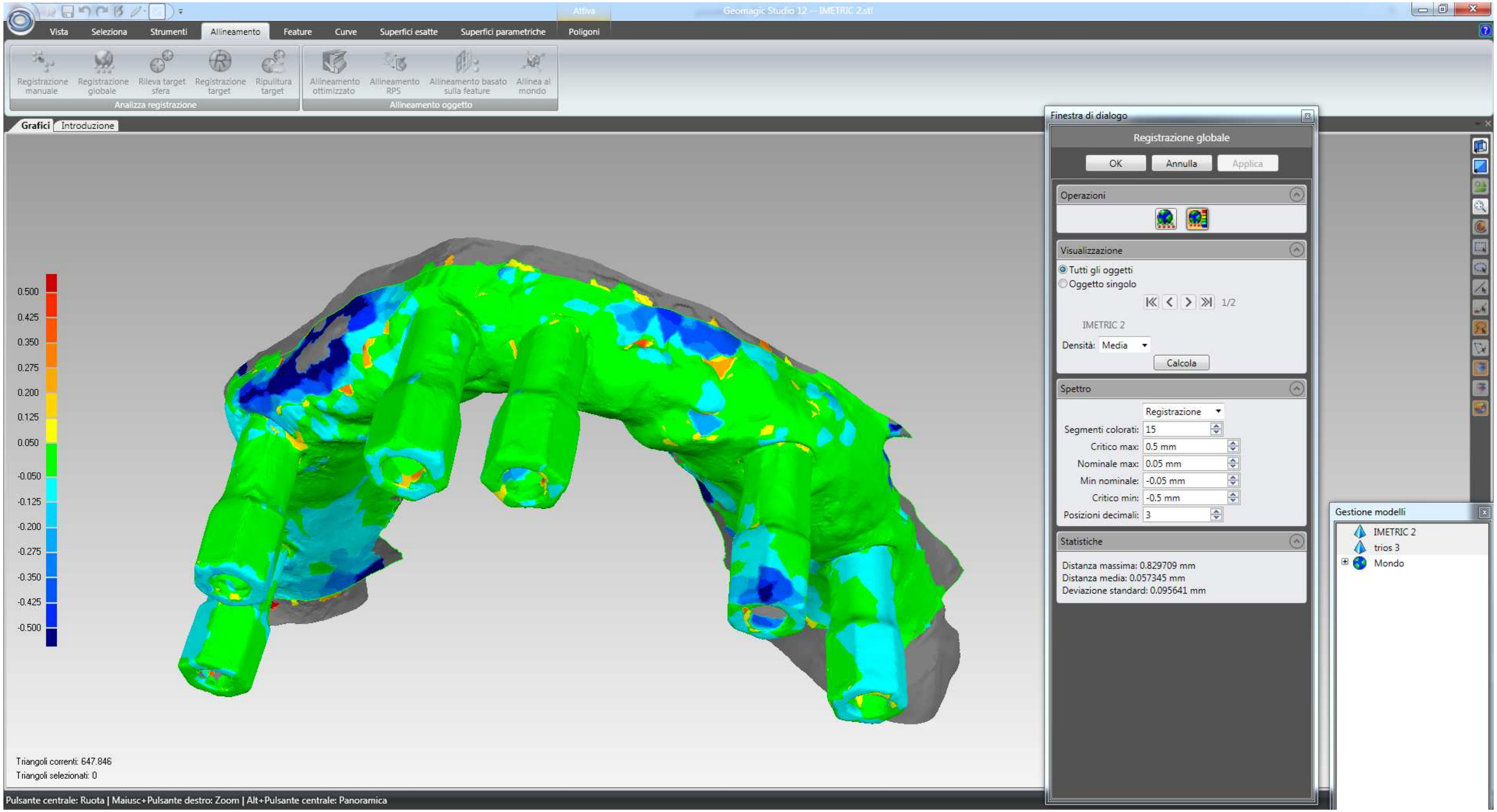

TRIOS 3 vs REF (IMETRIC)

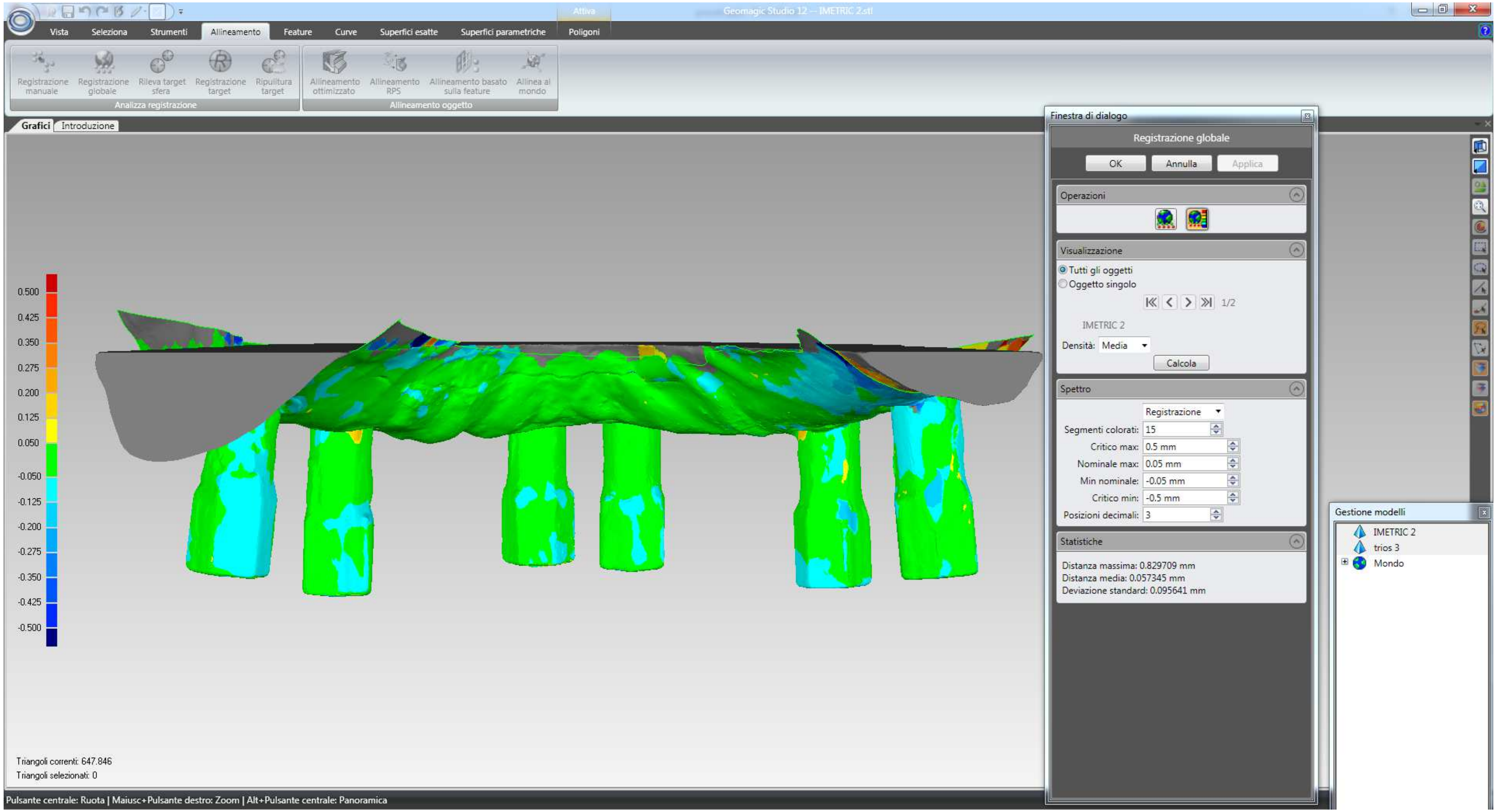

TRIOS 4 vs REF (IMETRIC)

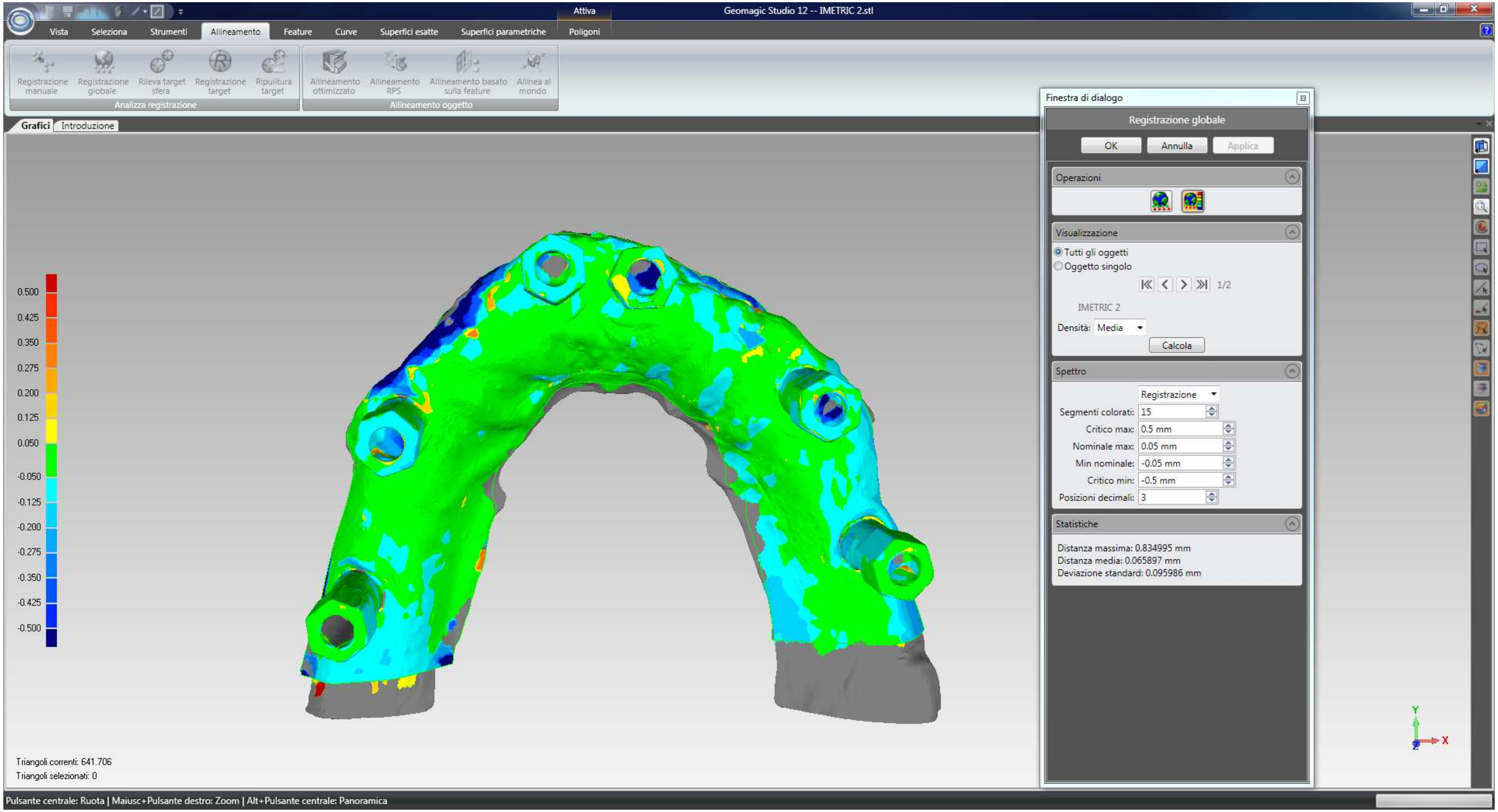

TRIOS 4 vs REF (IMETRIC)

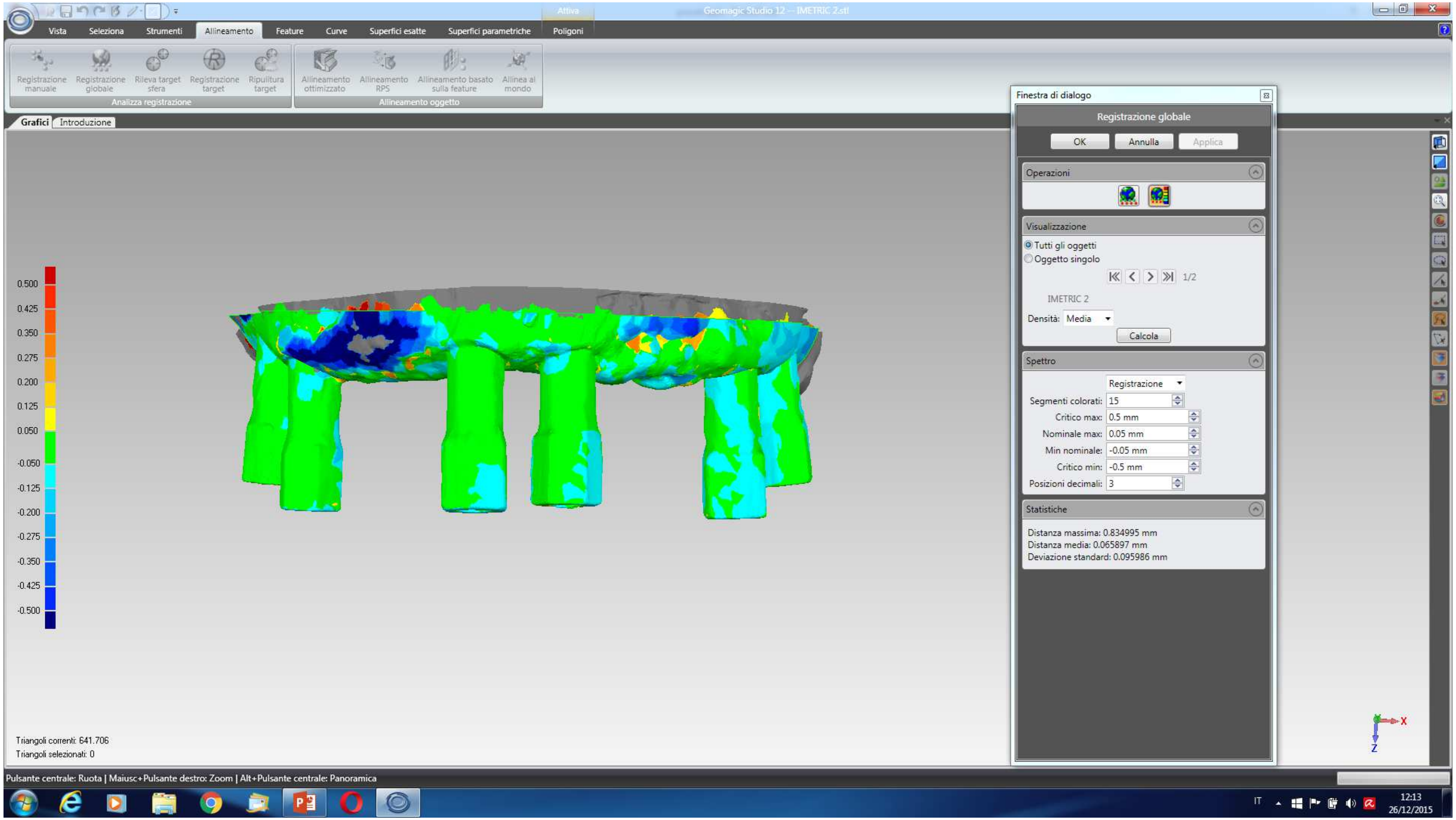

TRIOS 4 vs REF (IMETRIC)

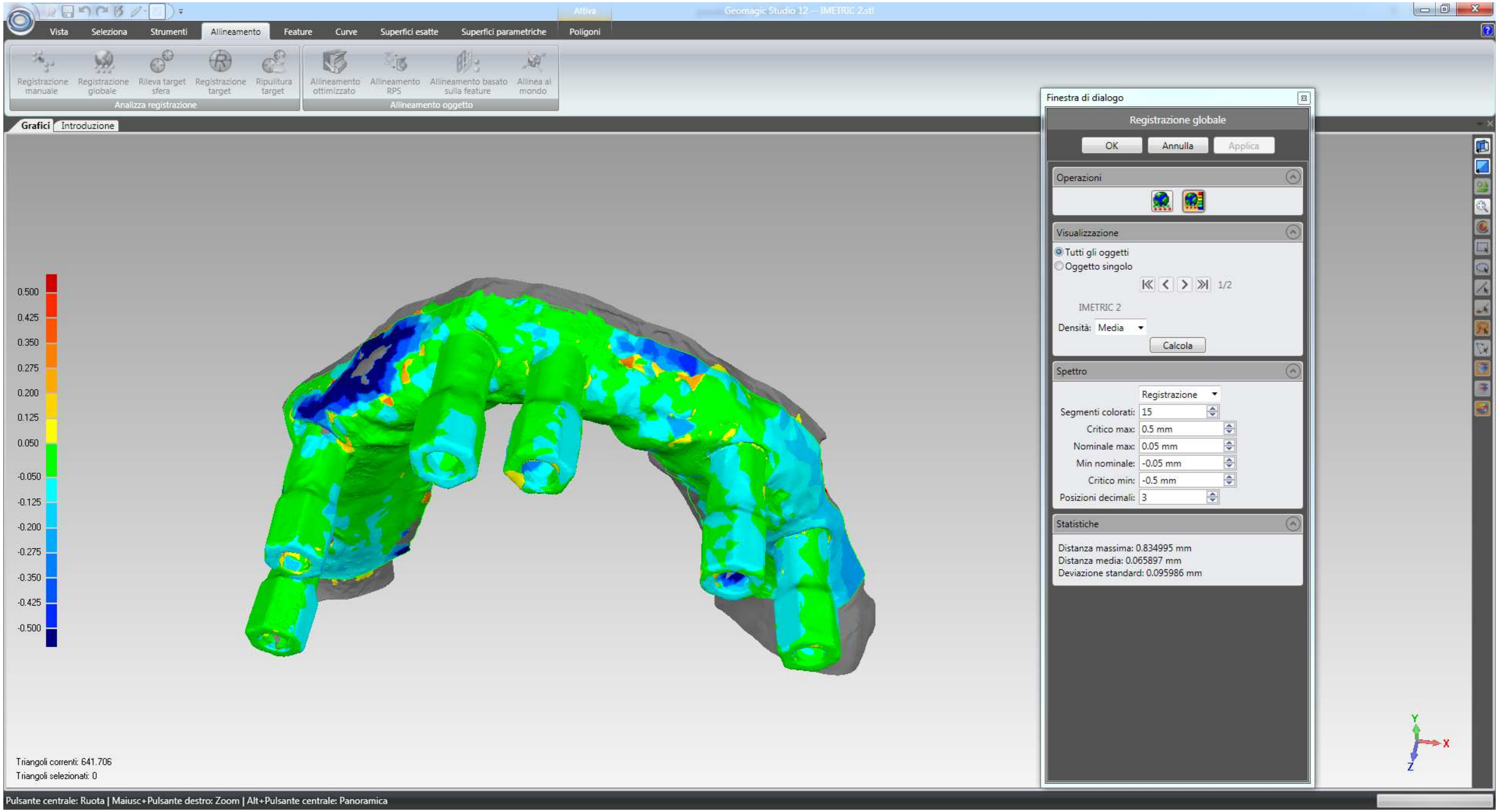

TRIOS 4 vs REF (IMETRIC)

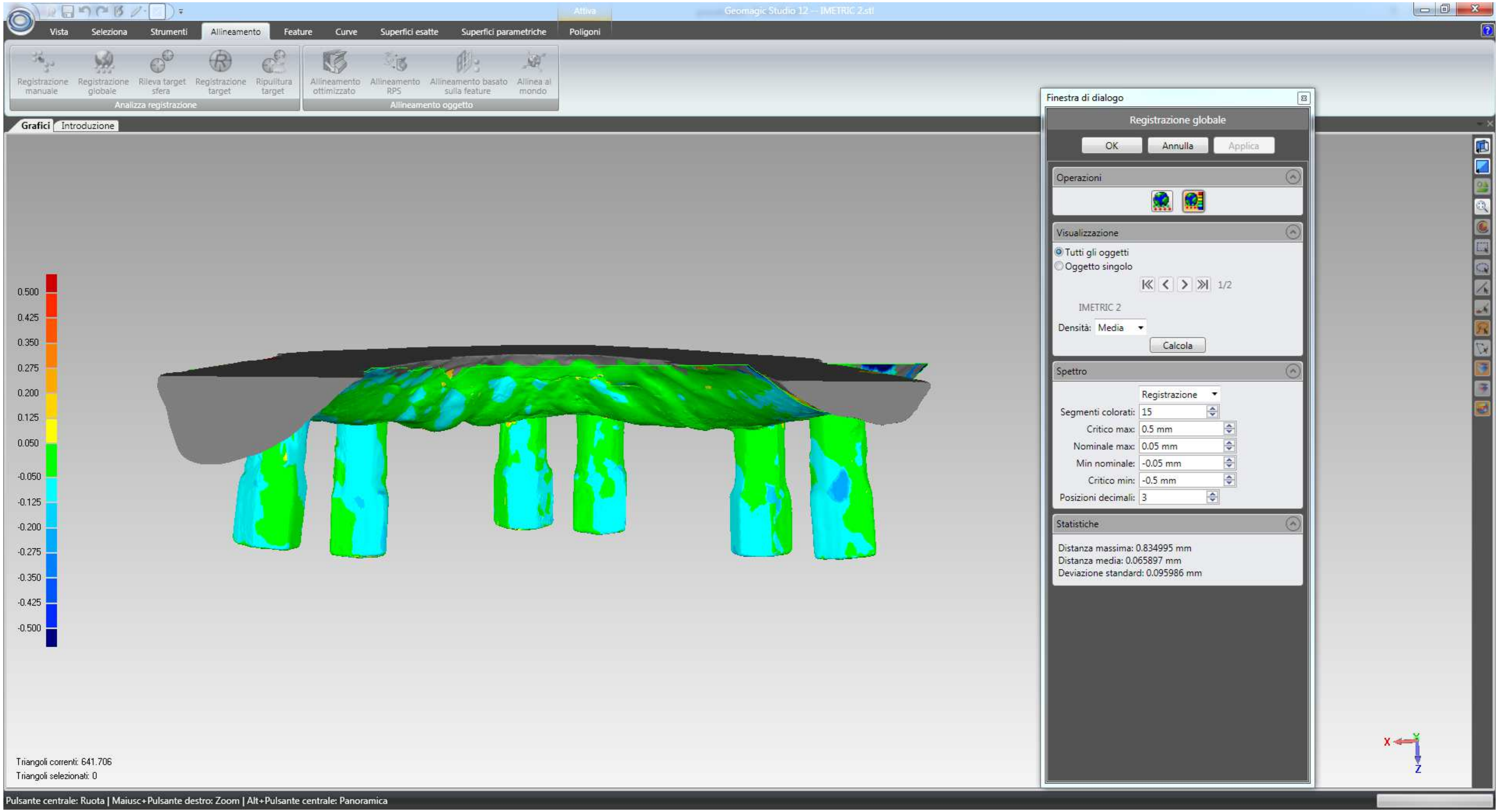

TRIOS 5 vs REF (IMETRIC)

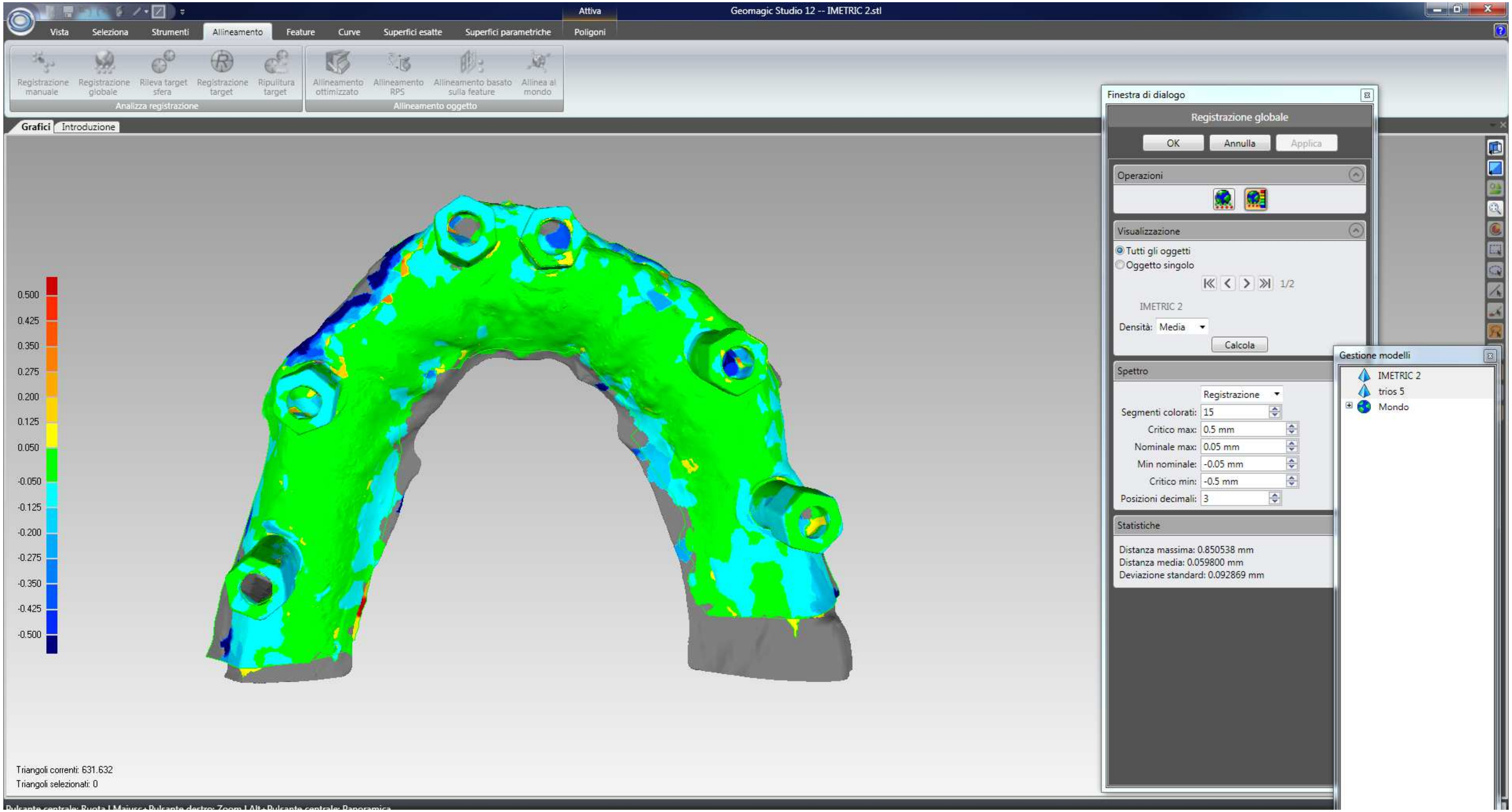

TRIOS 5 vs REF (IMETRIC)

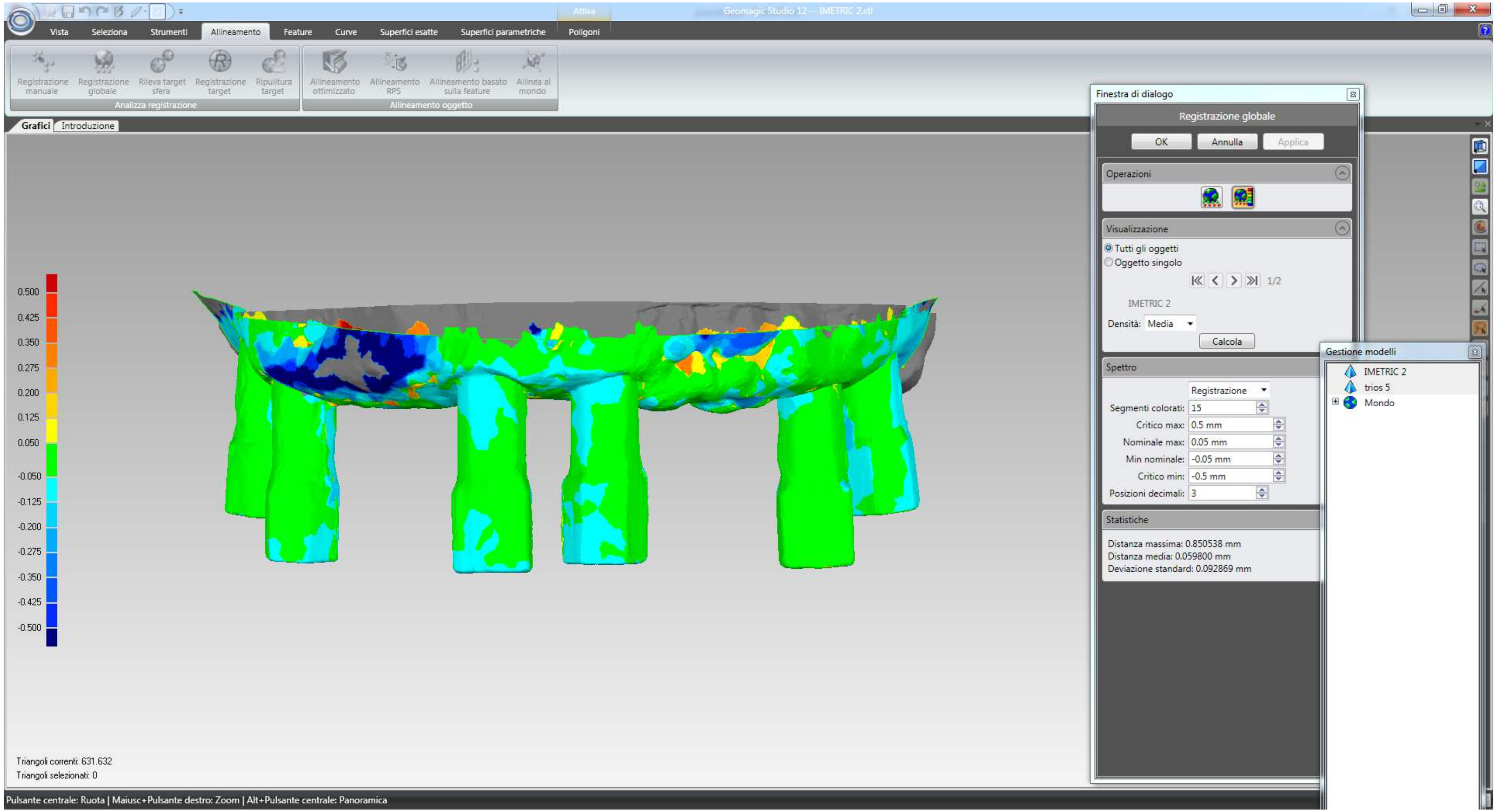

TRIOS 5 vs REF (IMETRIC)

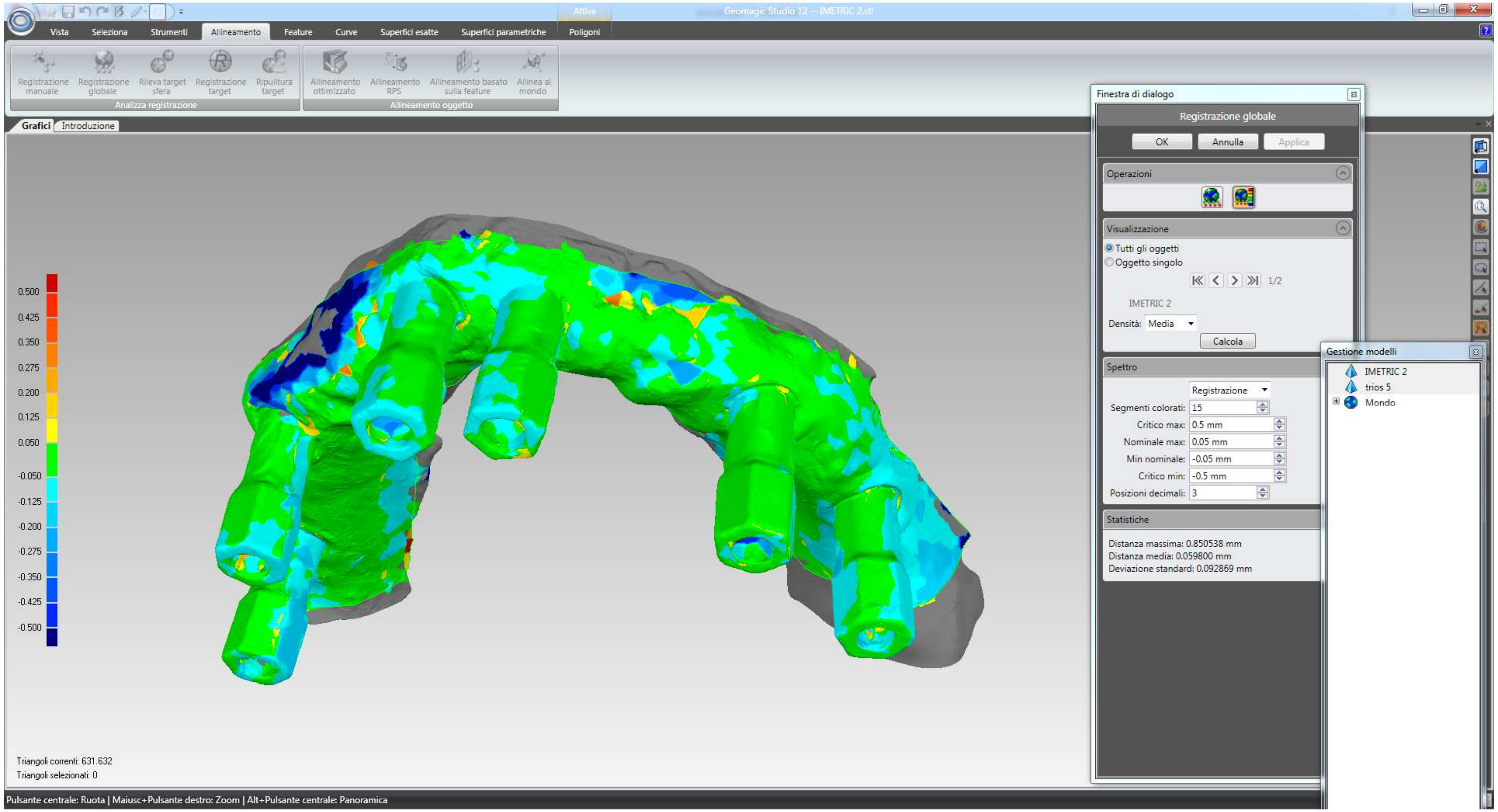

TRIOS 5 vs REF (IMETRIC)

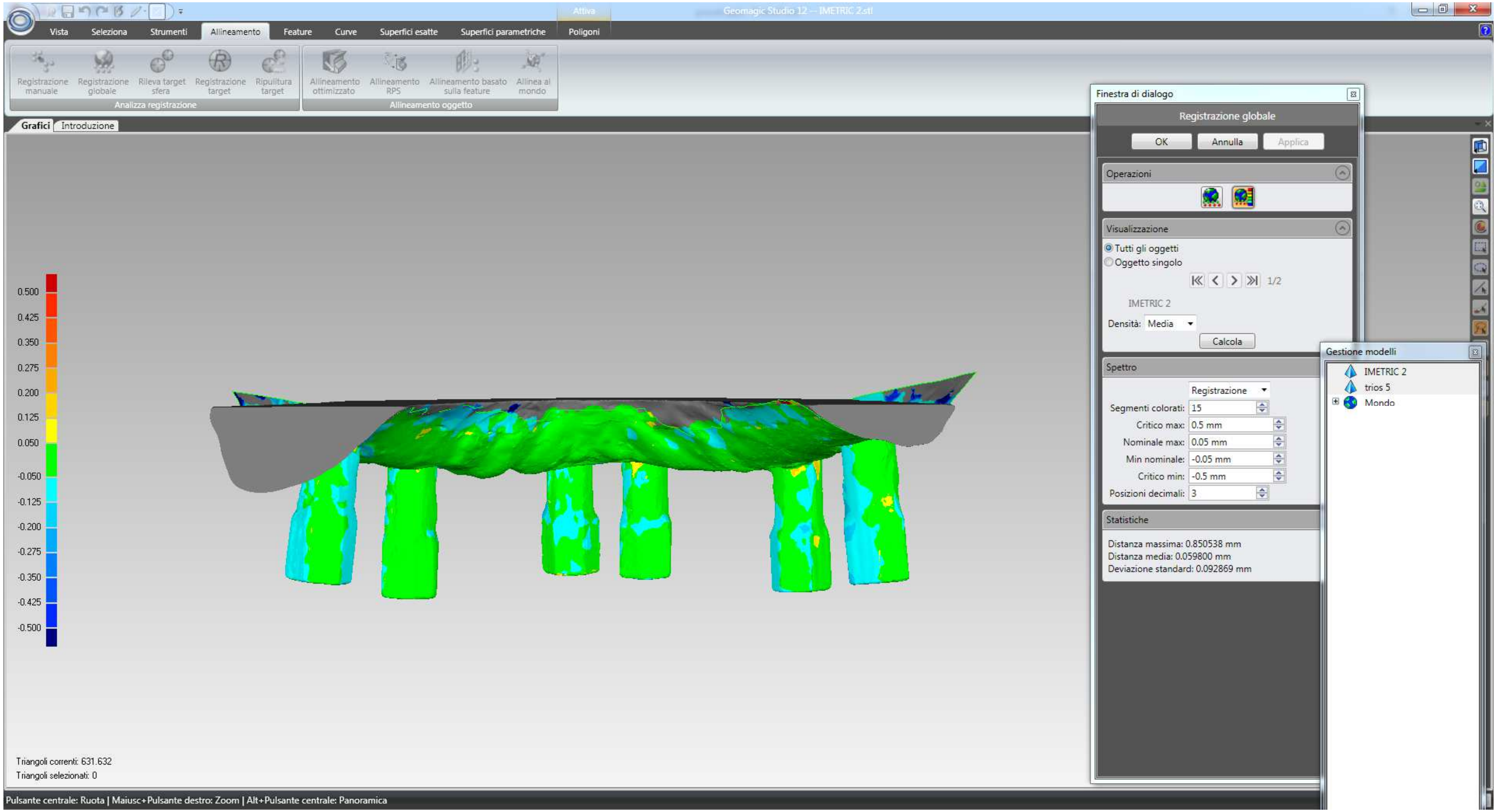

TRIOS general trueness in the totally edentulous model

	Mean distance	SD	Maximum distance
Trios 1	0.119	0.135	0.833
Trios 2	0.058	0.095	0.834
Trios 3	0.057	0.095	0.829
Trios 4	0.065	0.095	0.834
Trios 5	0.059	0.092	0.850

Overall Trios general accuracy: 0.071 (0.026)

ZFX 1 vs REF (IMETRIC)

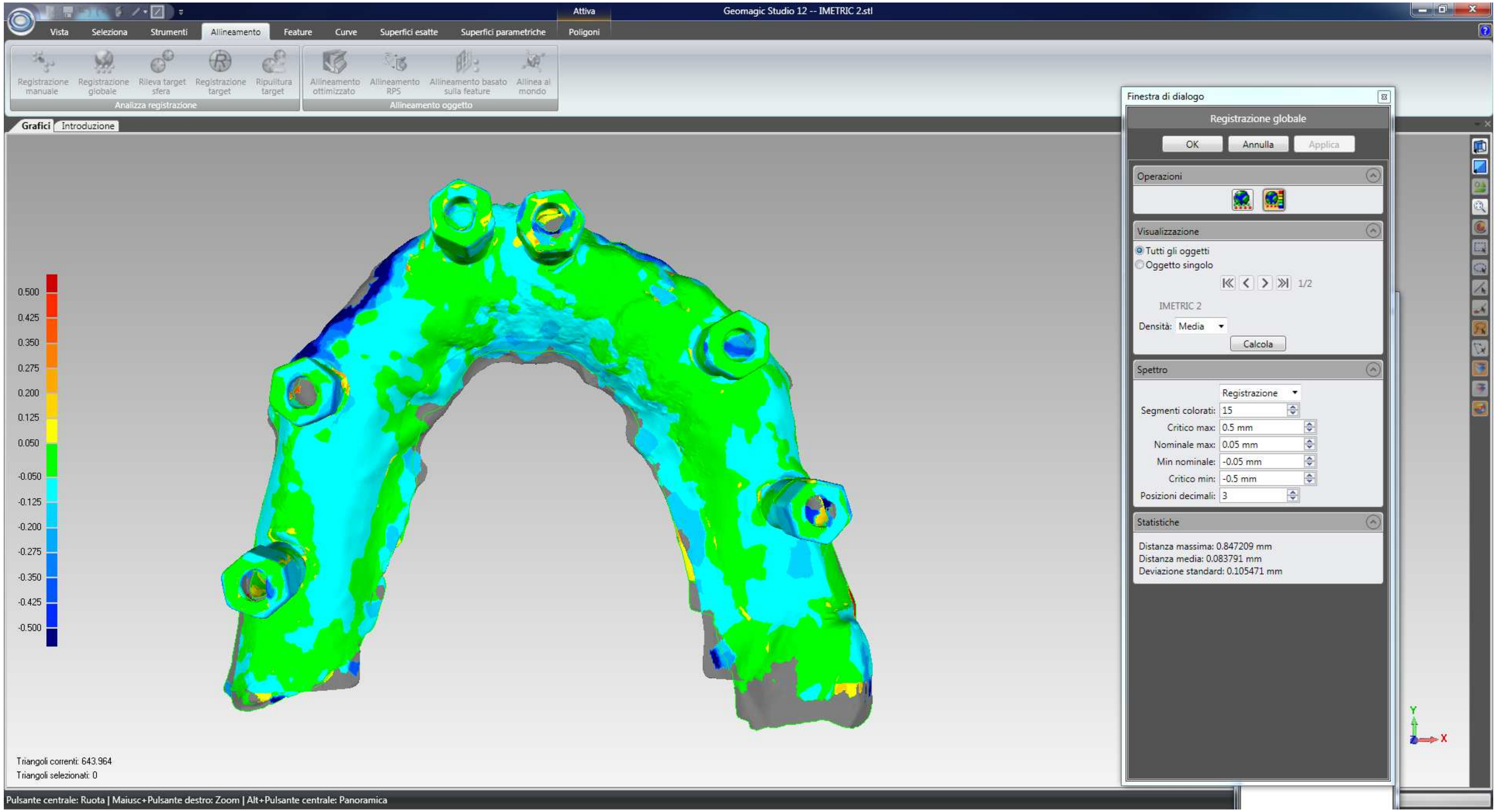

ZFX 1 vs REF (IMETRIC)

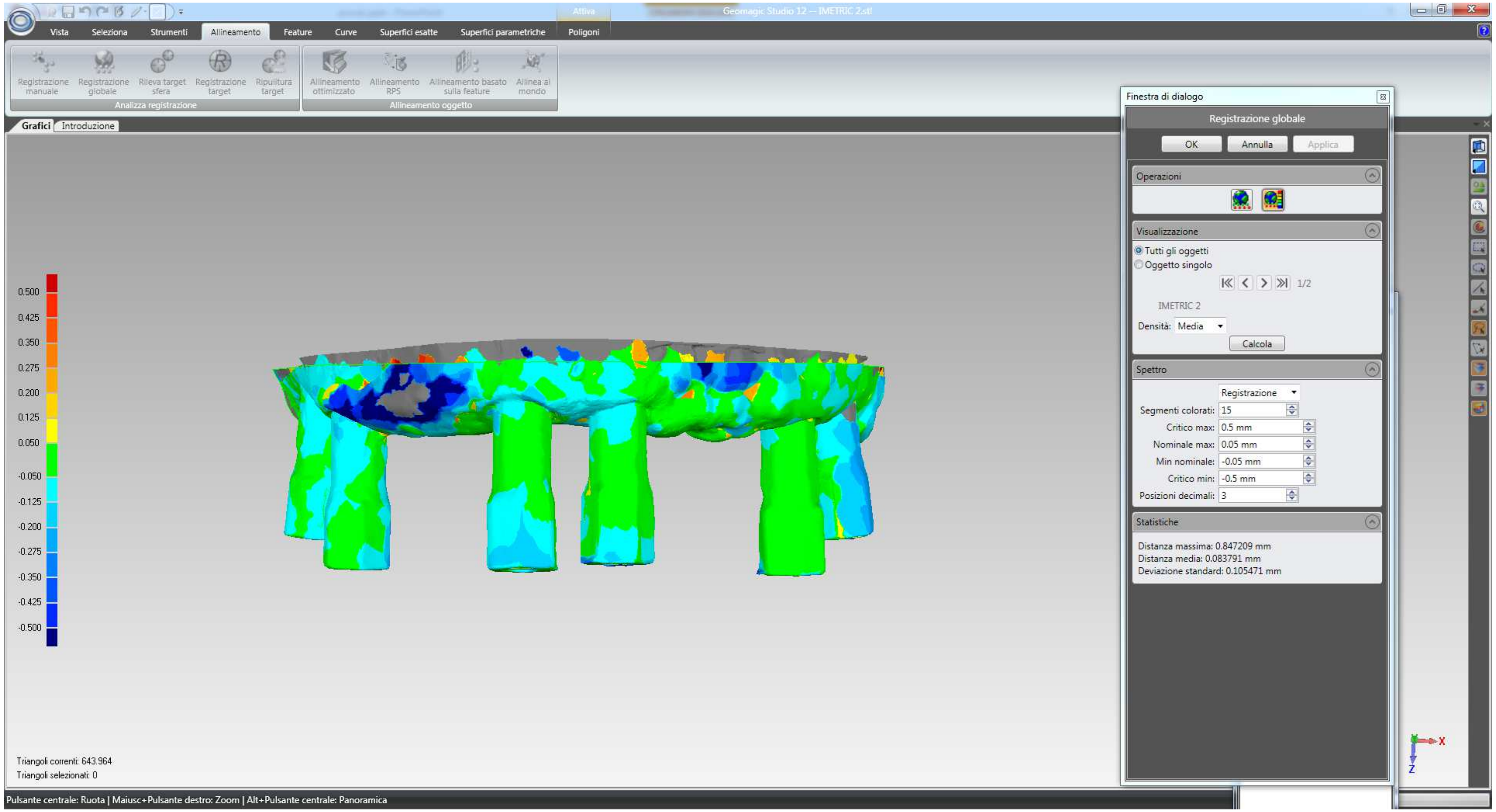

ZFX 1 vs REF (IMETRIC)

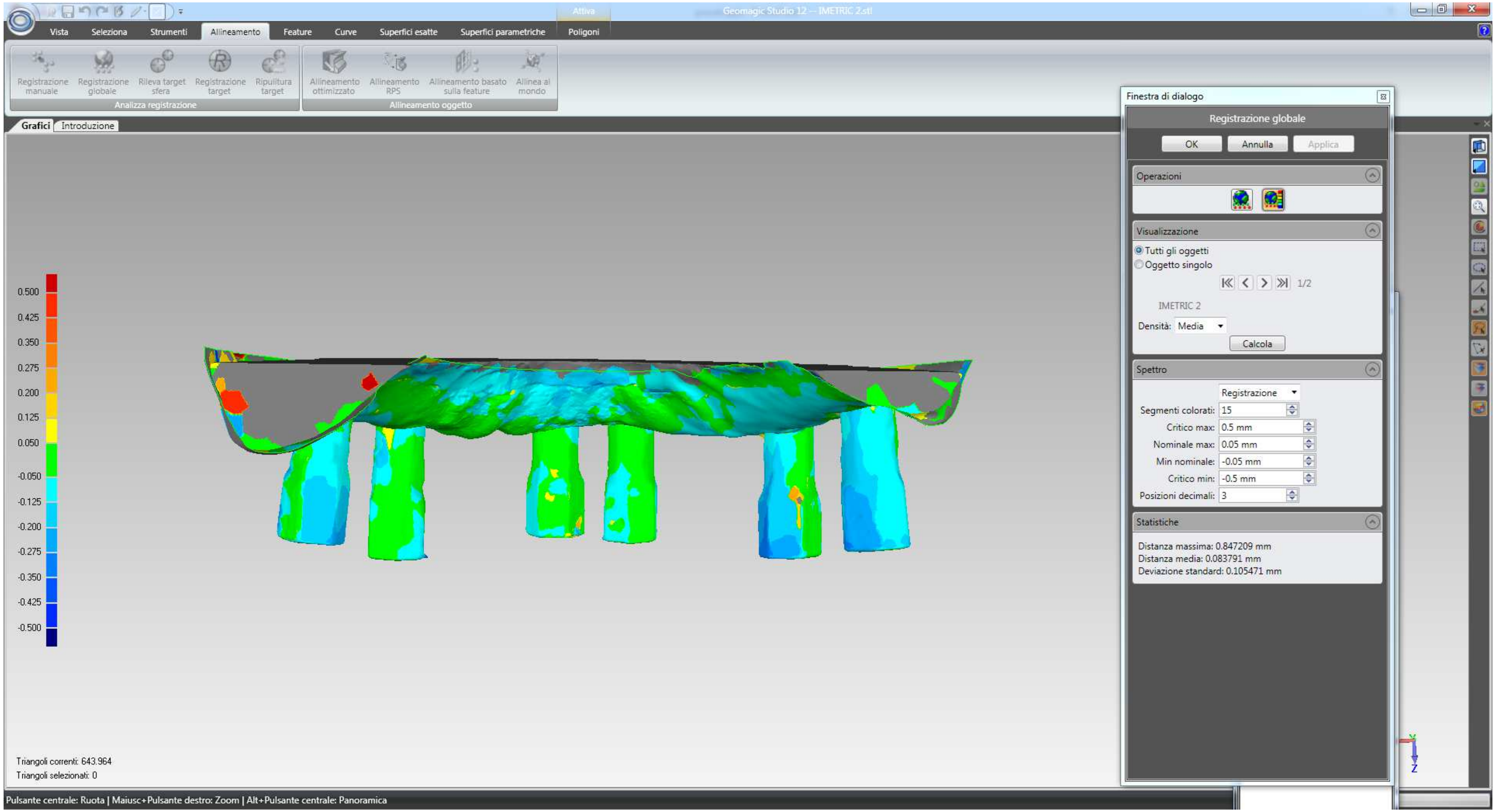

ZFX 1 vs REF (IMETRIC)

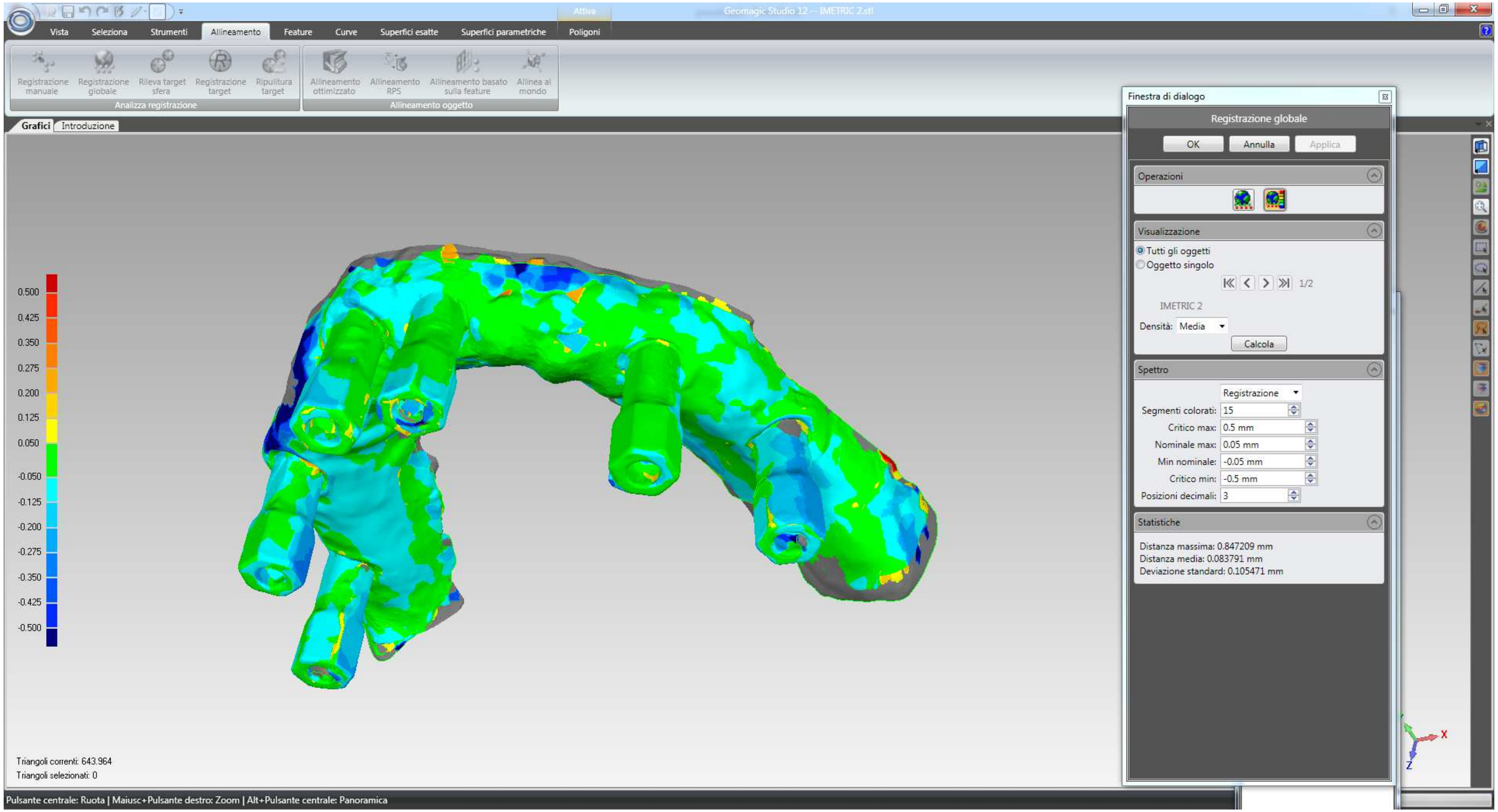

ZFX 2 vs REF (IMETRIC)

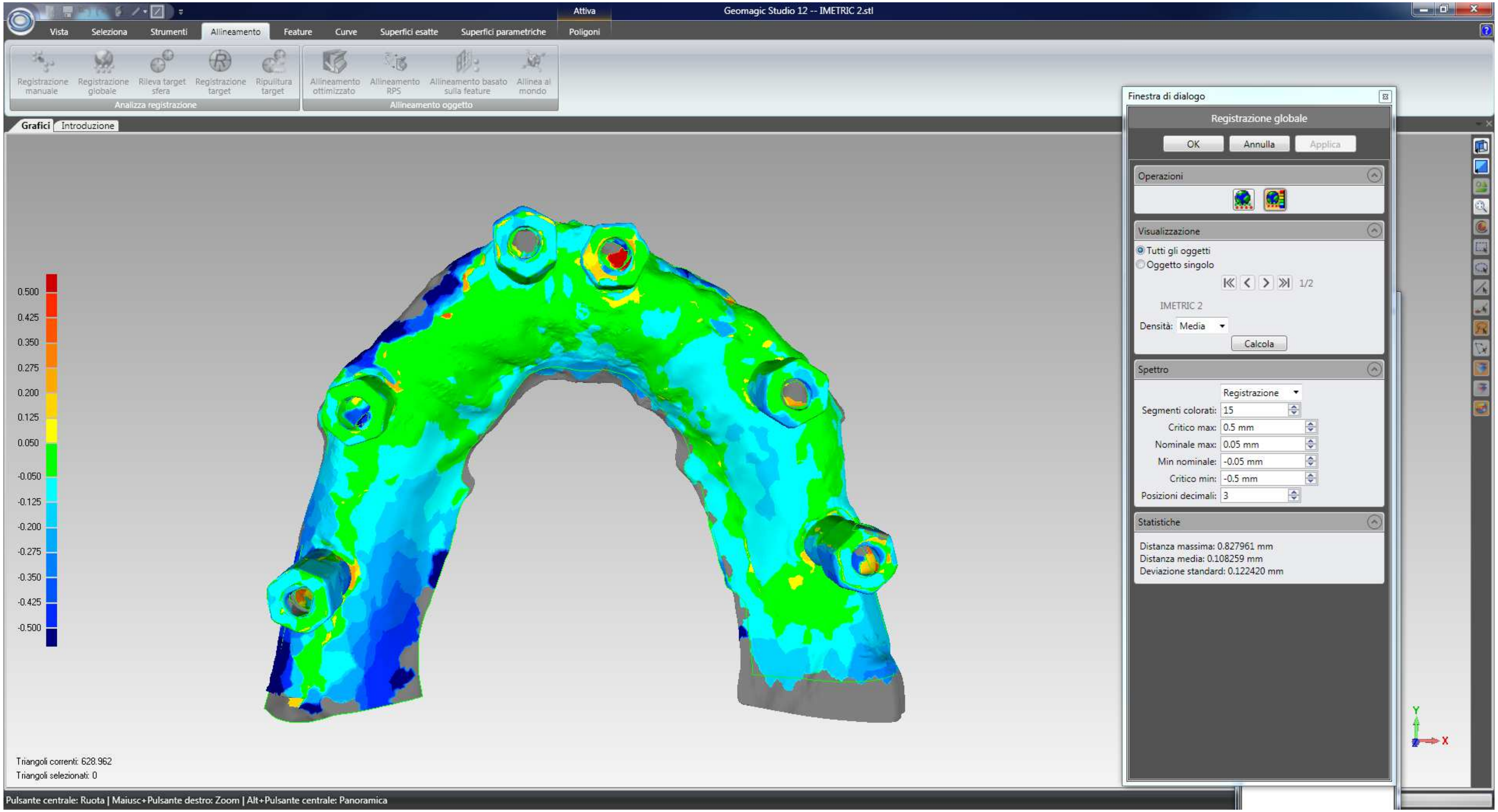

ZFX 2 vs REF (IMETRIC)

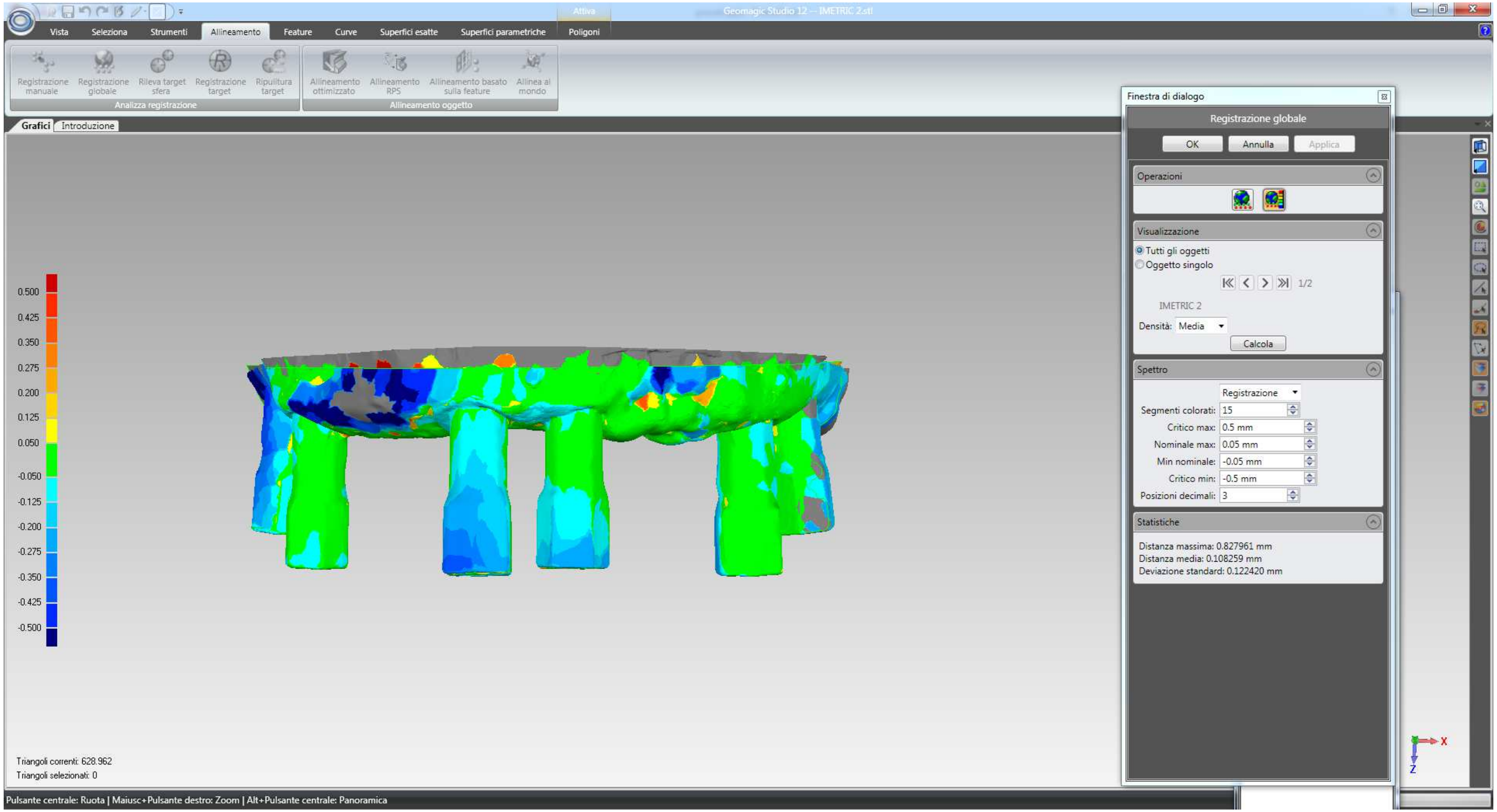

ZFX 2 vs REF (IMETRIC)

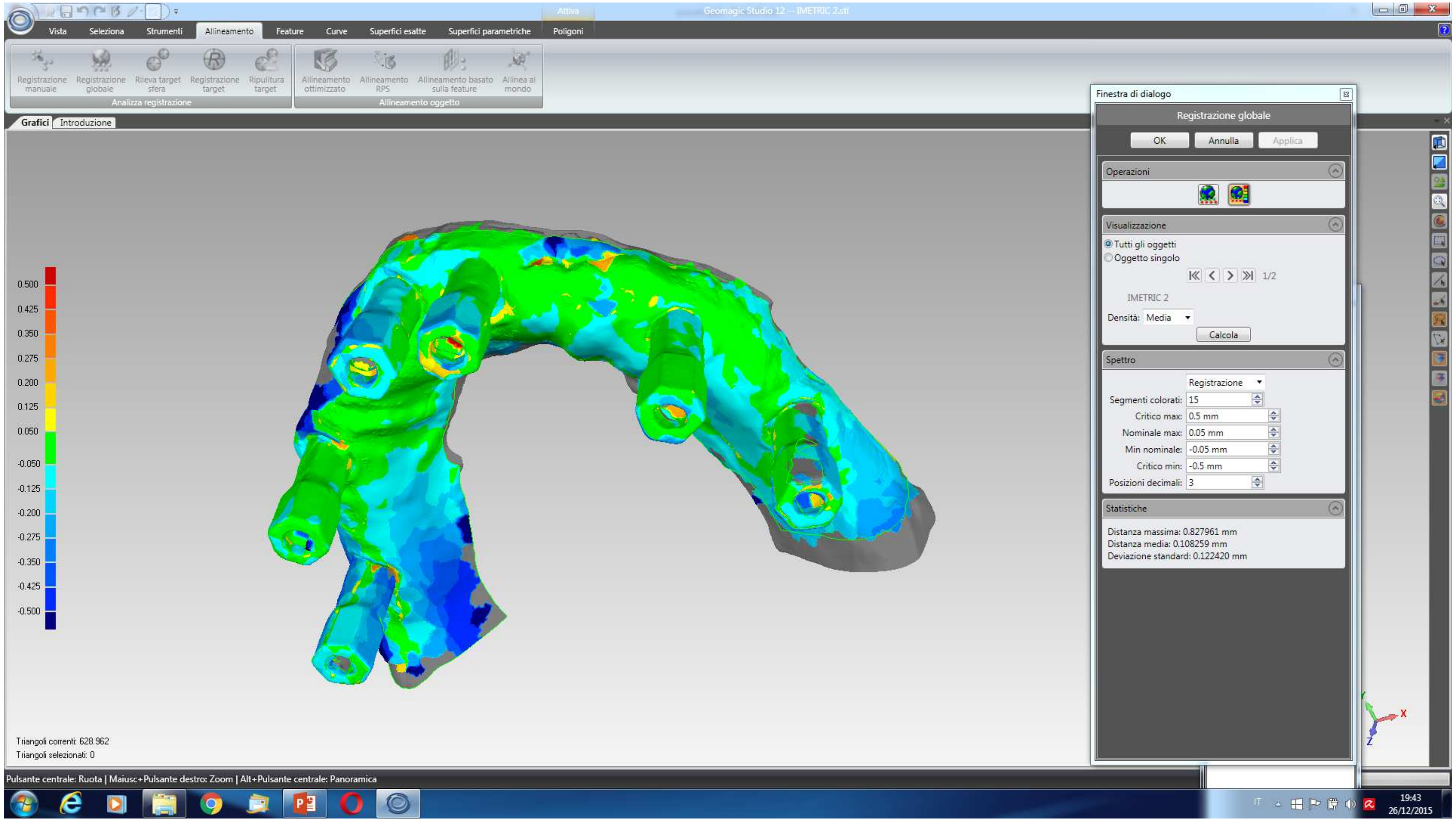

ZFX 2 vs REF (IMETRIC)

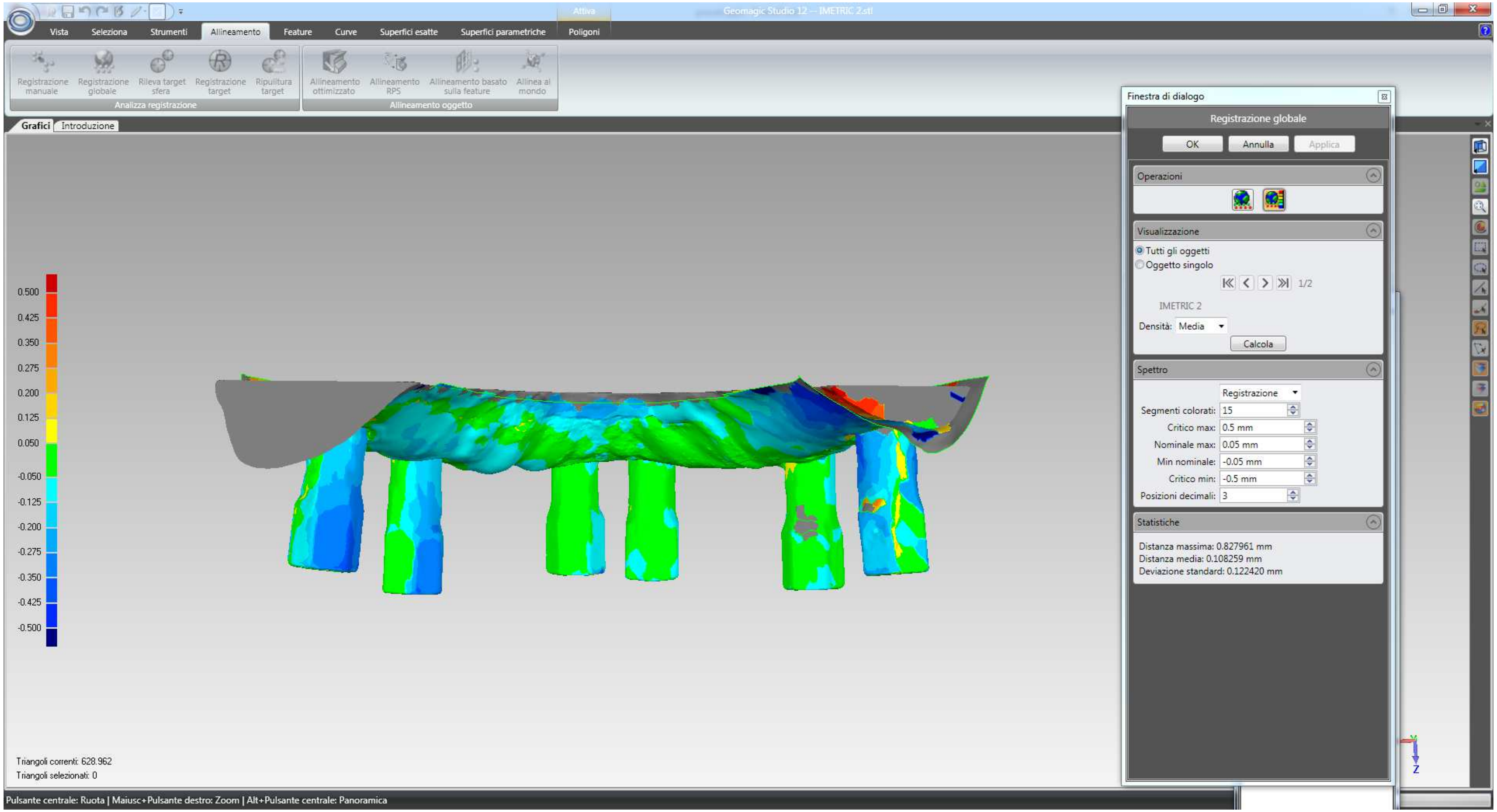

ZFX 3 vs REF (IMETRIC)

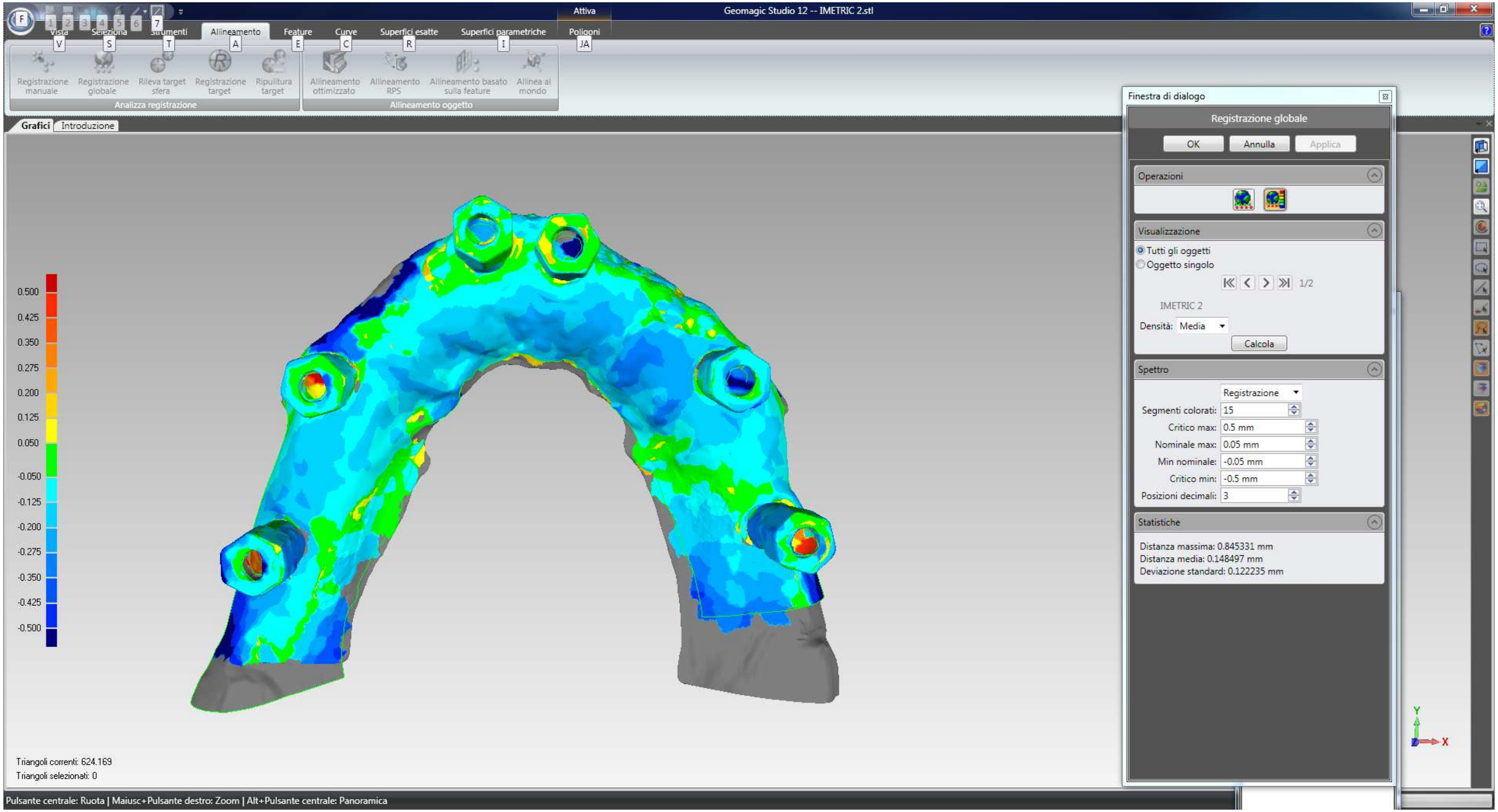

ZFX 3 vs REF (IMETRIC)

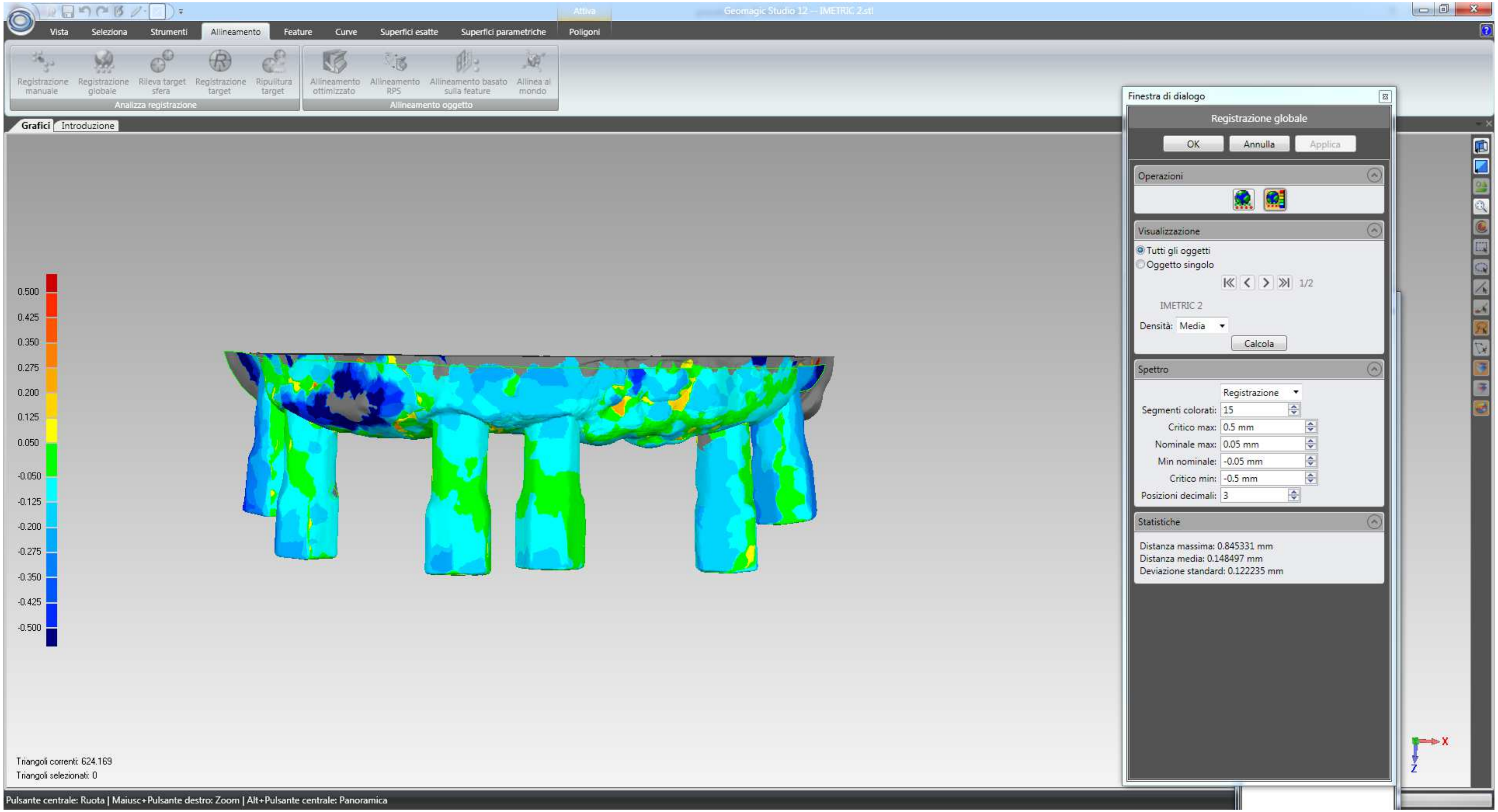

ZFX 3 vs REF (IMETRIC)

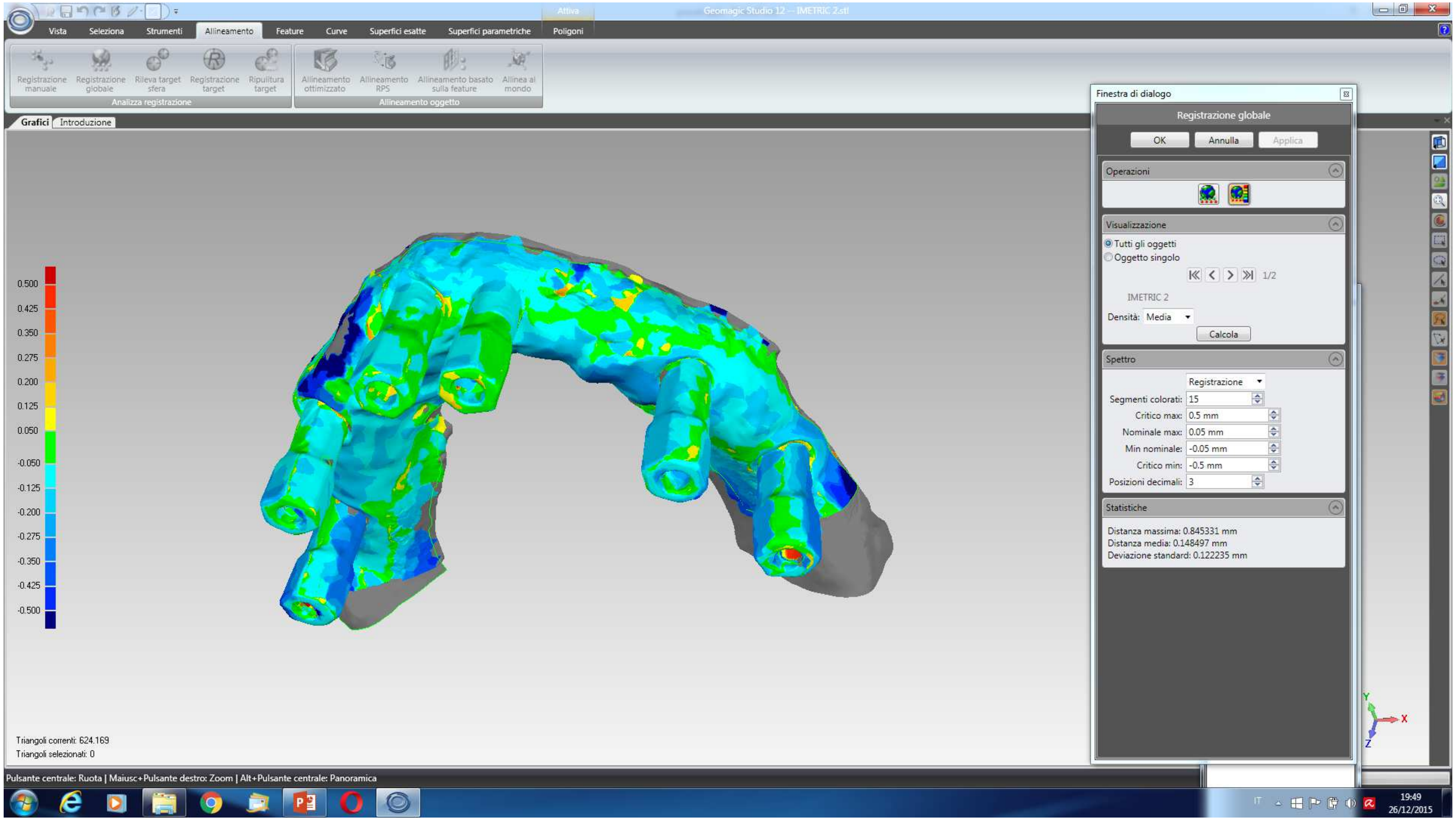

ZFX 3 vs REF (IMETRIC)

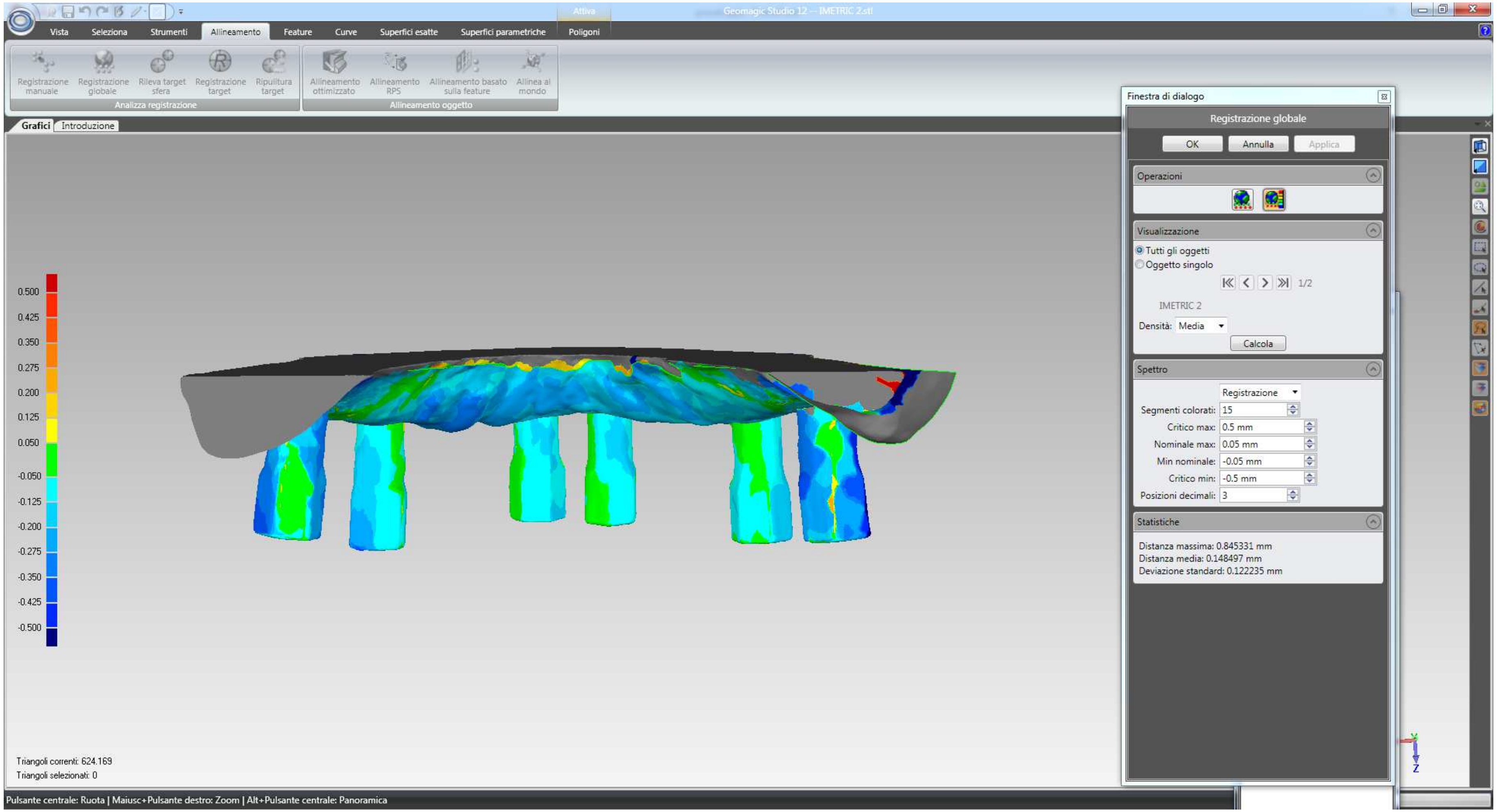

ZFX 4 vs REF (IMETRIC)

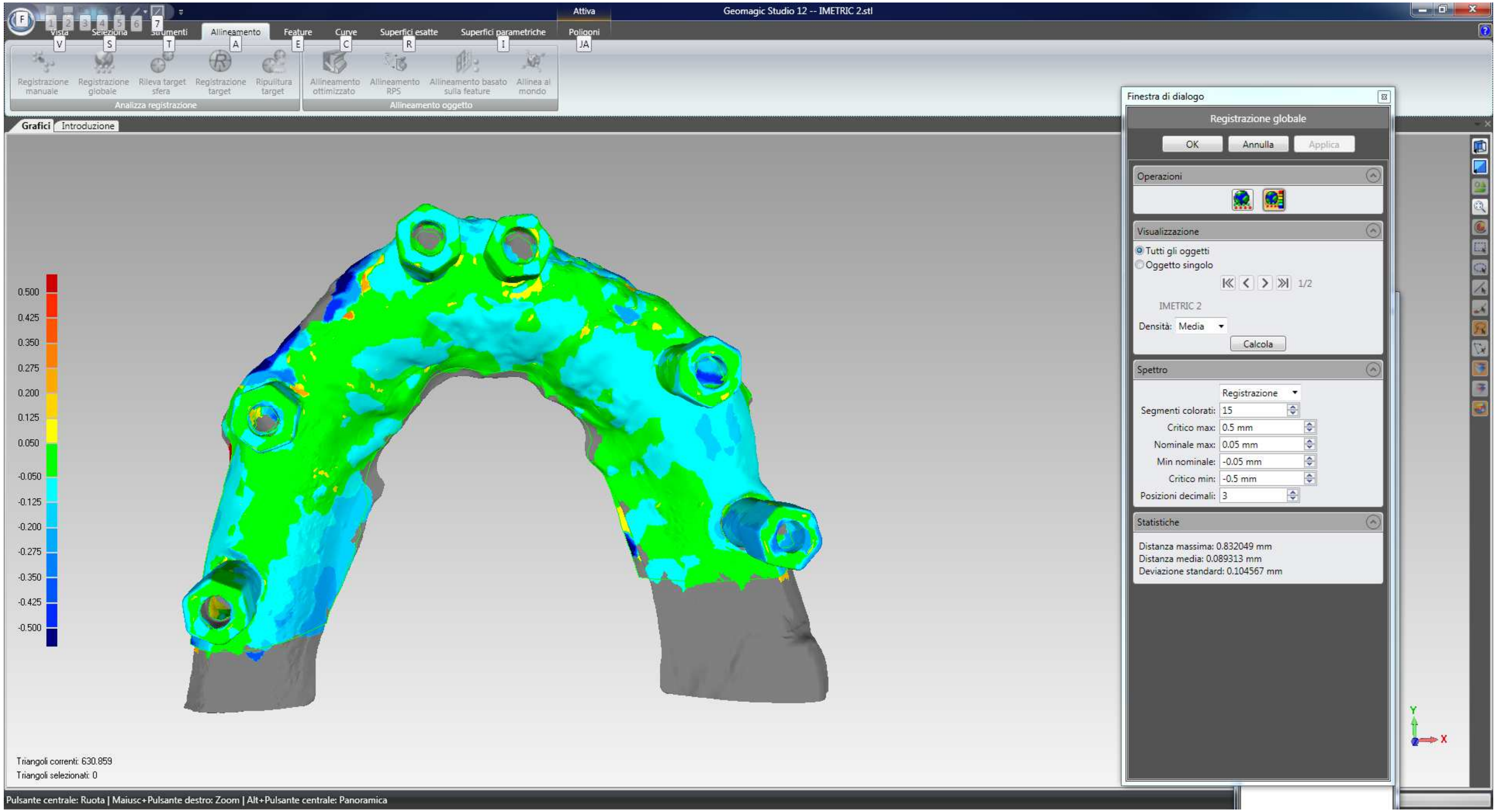

ZFX 4 vs REF (IMETRIC)

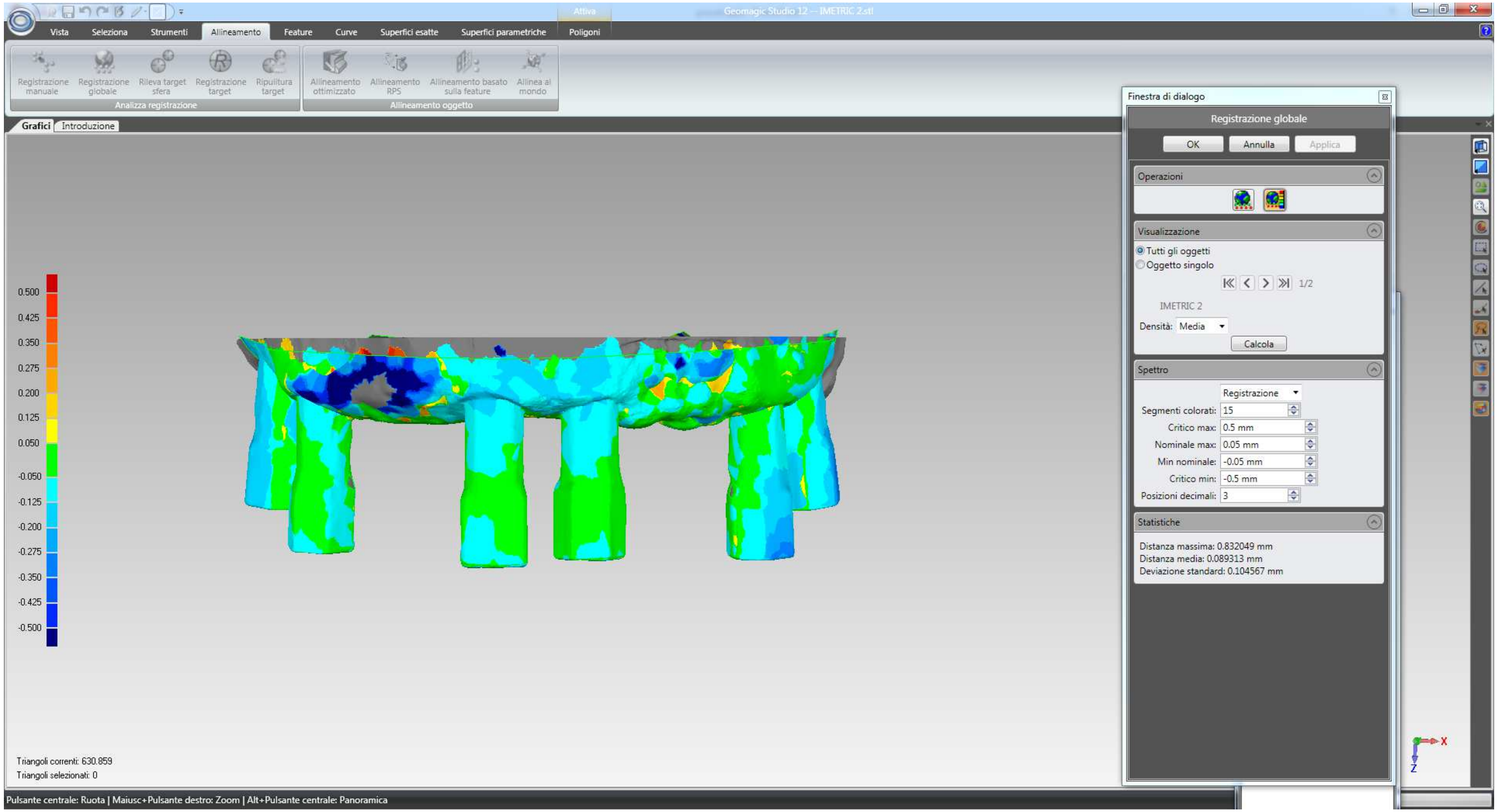

ZFX 4 vs REF (IMETRIC)

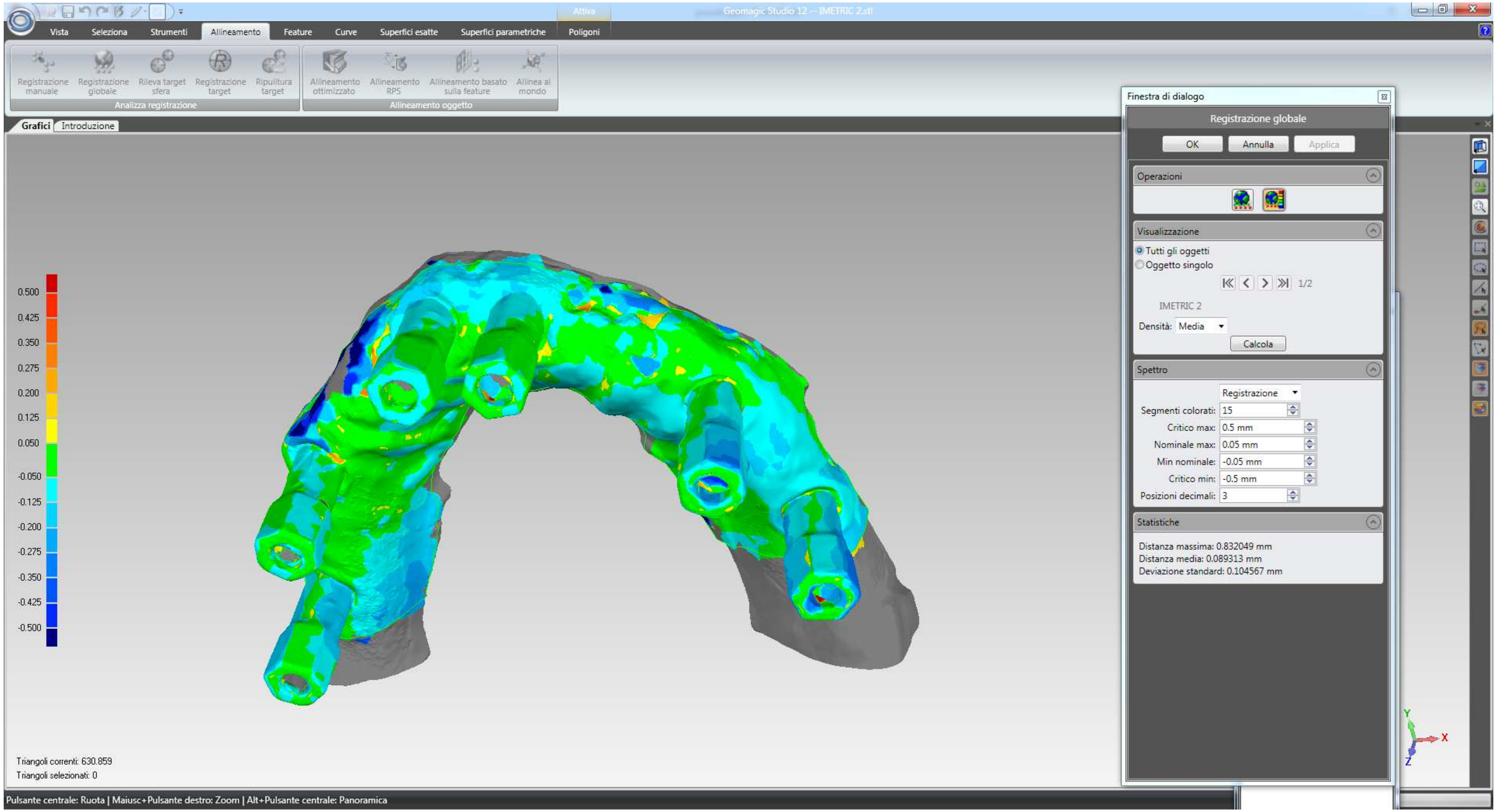

ZFX 4 vs REF (IMETRIC)

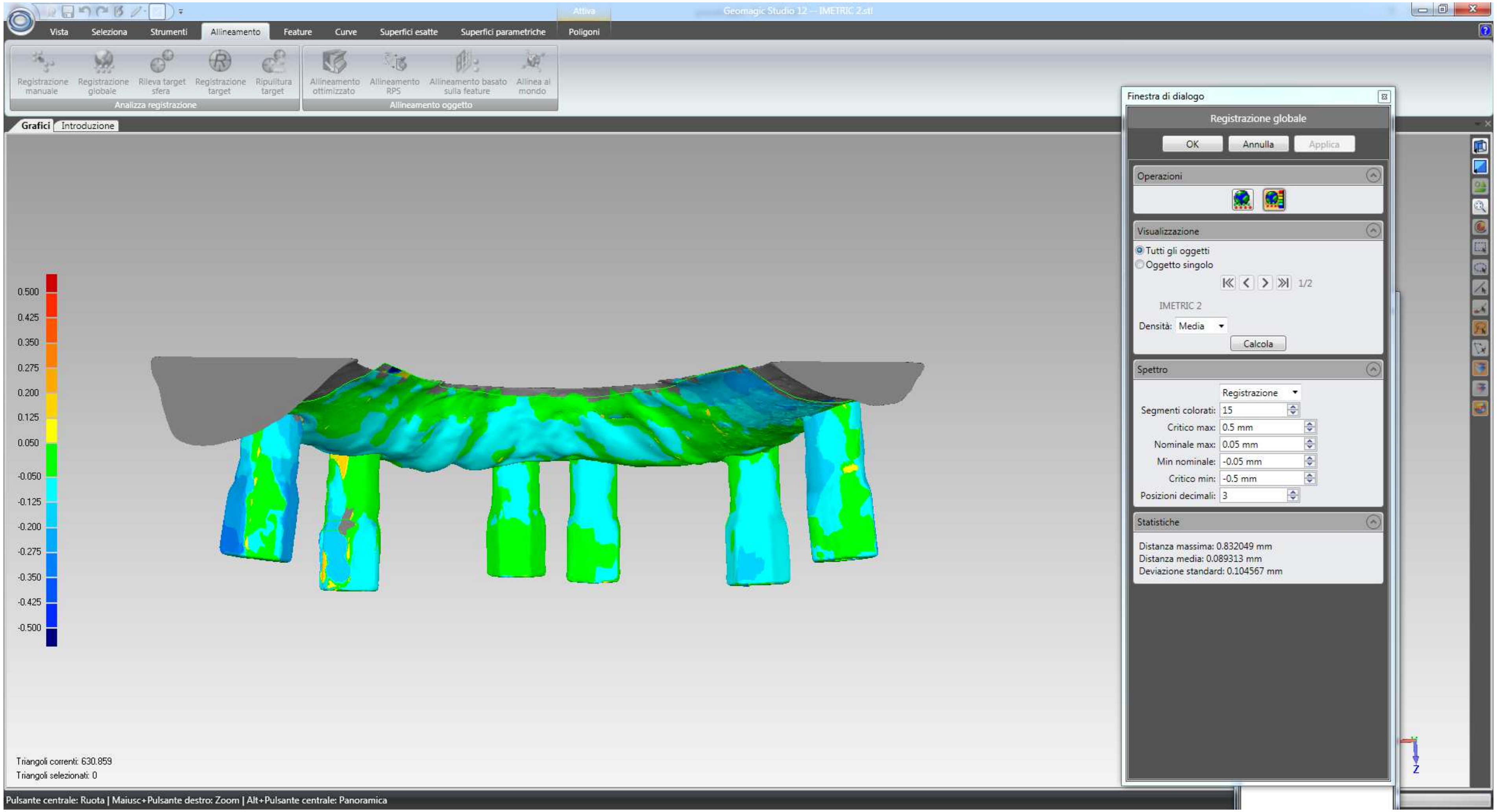

ZFX 5 vs REF (IMETRIC)

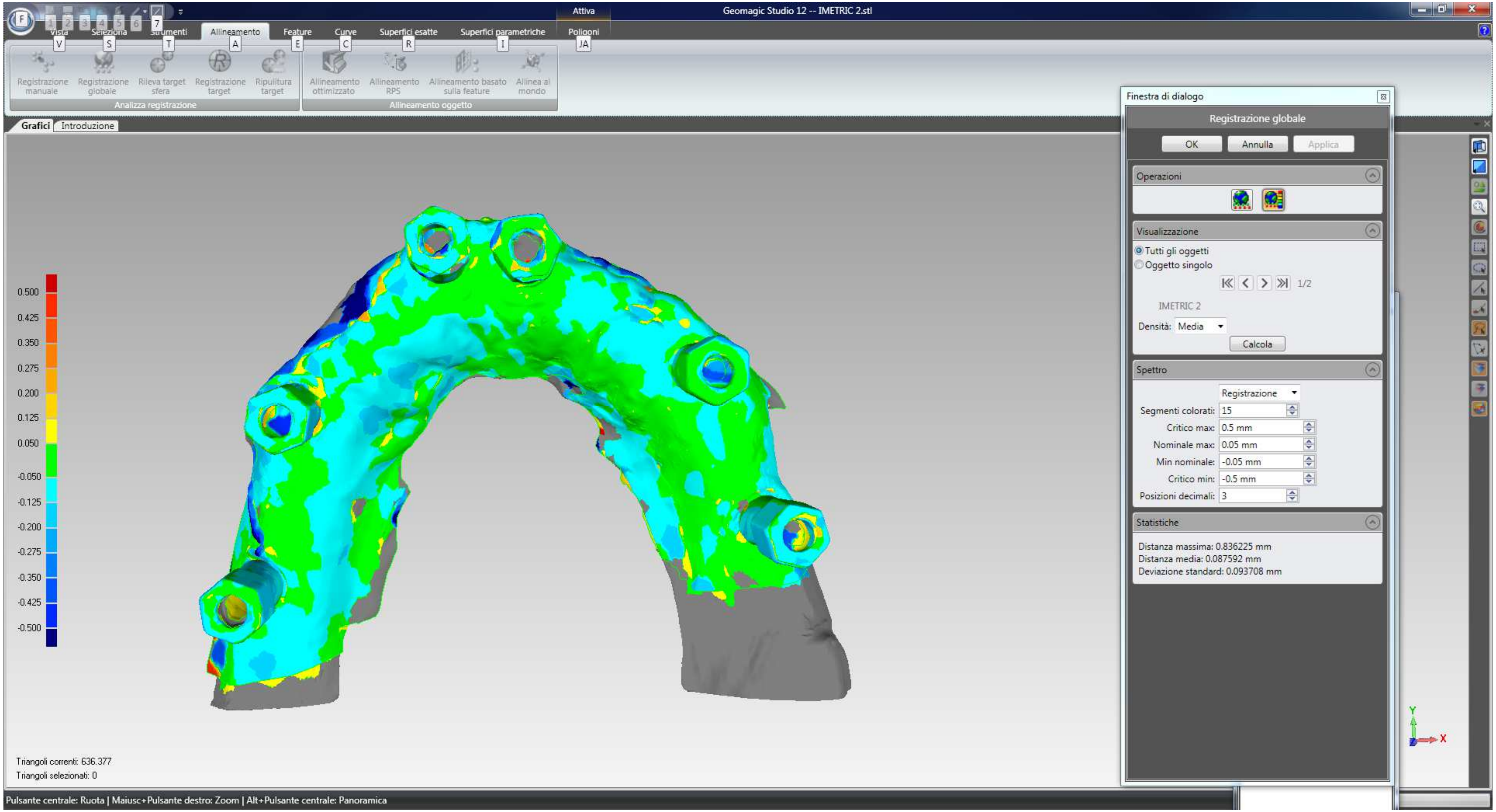

ZFX 5 vs REF (IMETRIC)

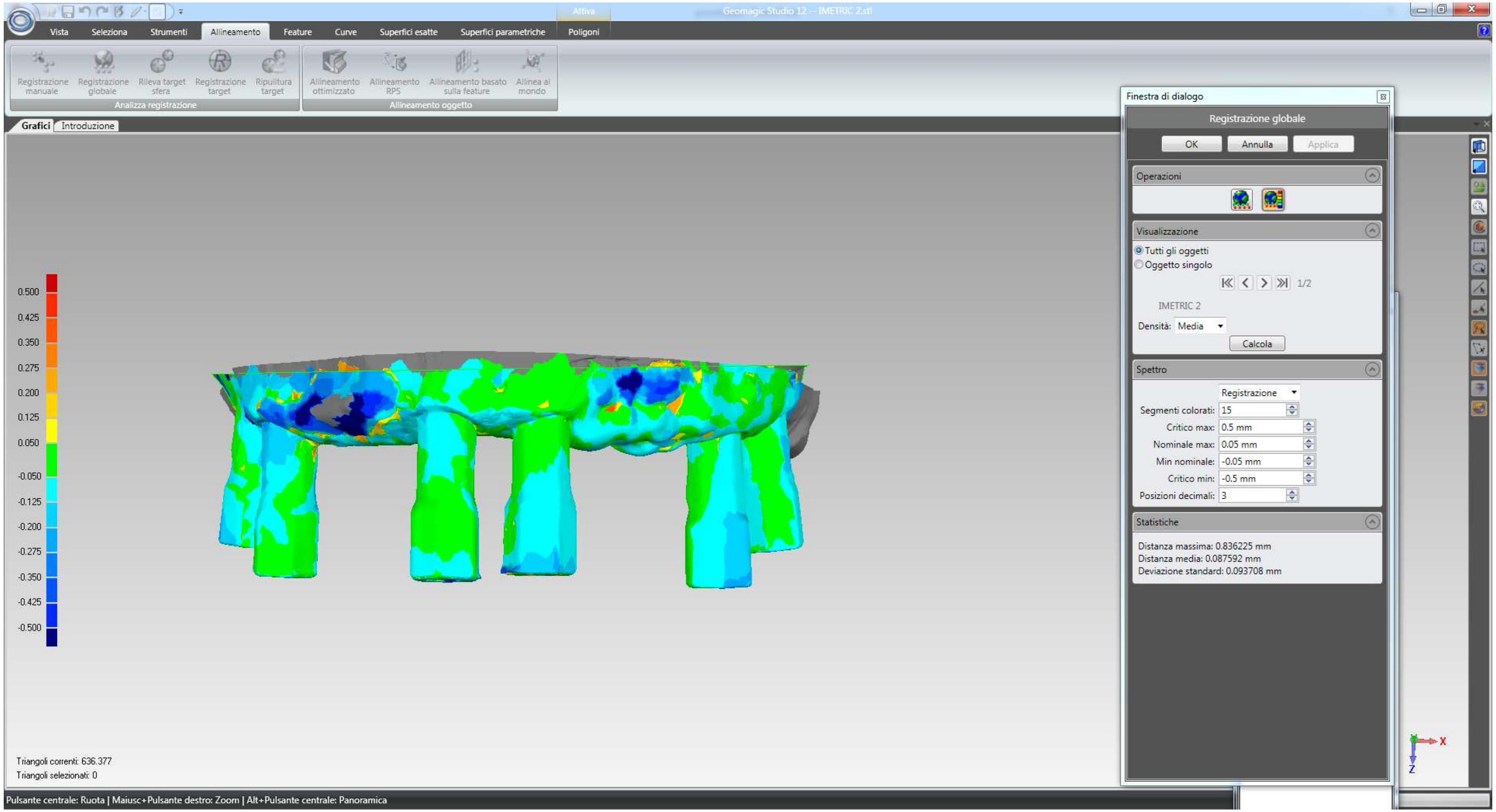

ZFX 5 vs REF (IMETRIC)

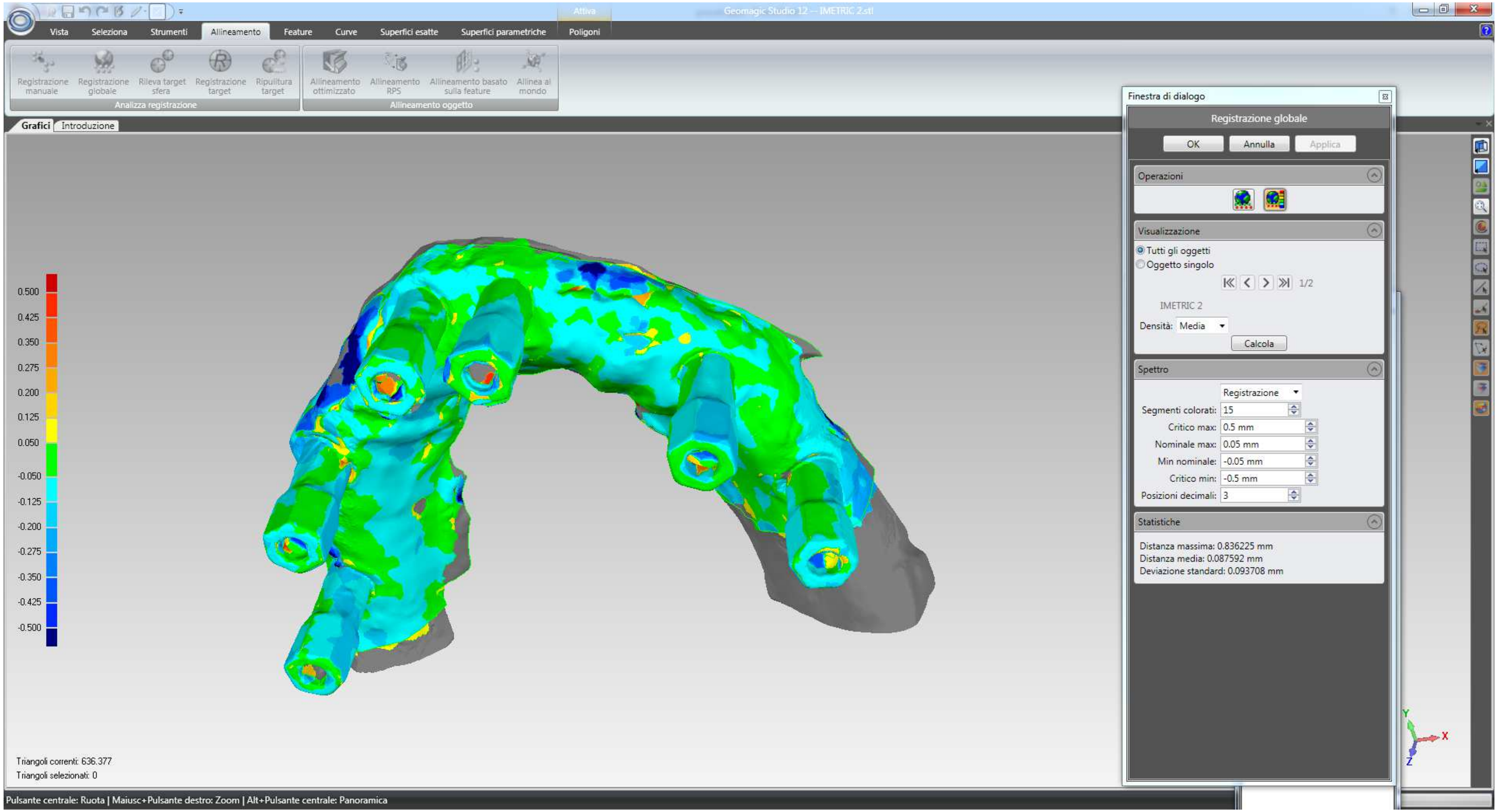

ZFX 5 vs REF (IMETRIC)

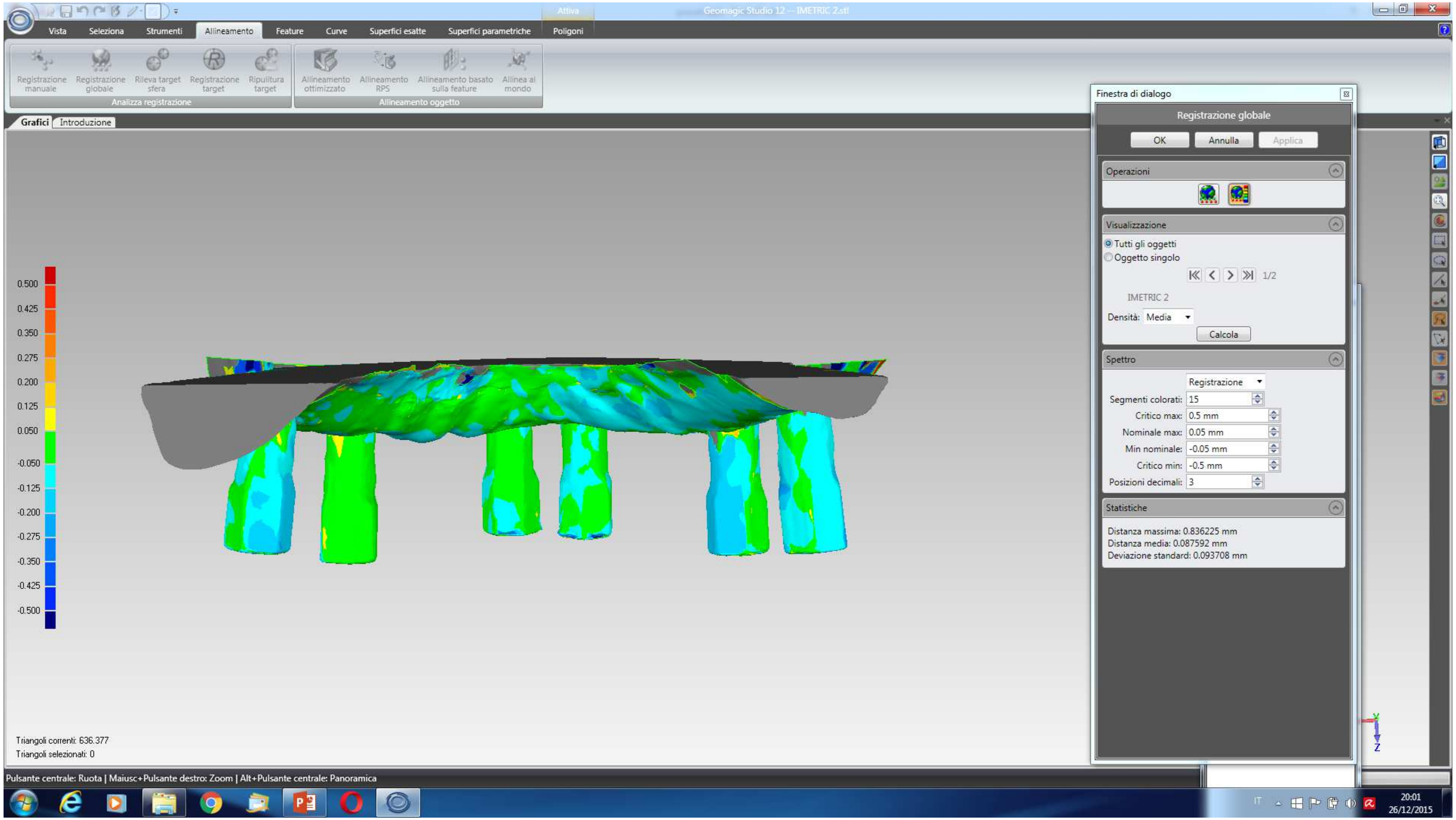

General trueness of Zfx Intrascan in the totally edentulous patient

	Mean distance	SD	Maximum distance
Zfx 1	0.083	0.105	0.847
Zfx 2	0.108	0.122	0.827
Zfx 3	0.148	0.122	0.845
Zfx 4	0.089	0.104	0.832
Zfx 5	0.087	0.093	0.836

Overall Zfx general accuracy: 0.103 (0.026)

Care 1 vs REF (IMETRIC)

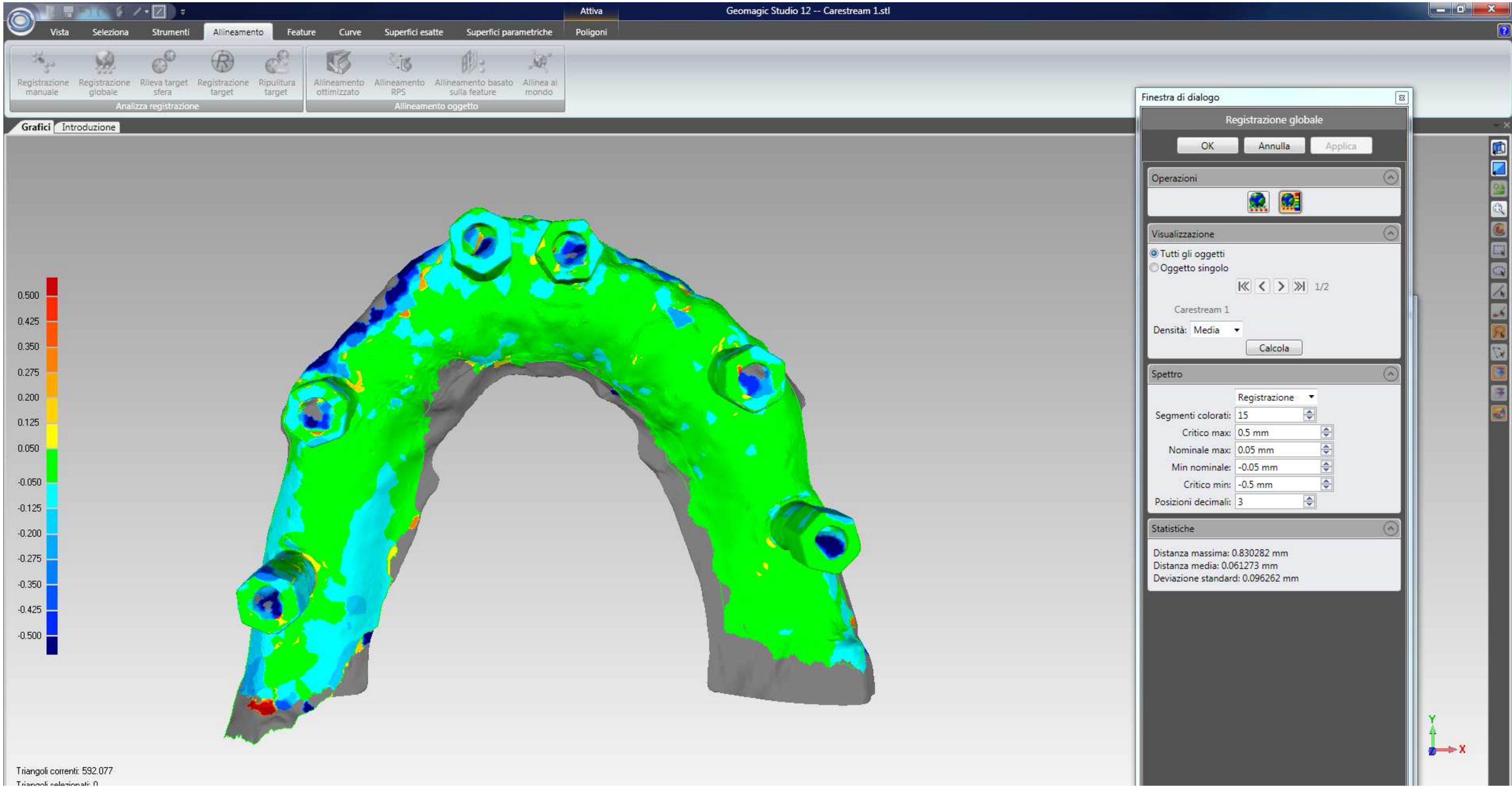

Care 1 vs REF (IMETRIC)

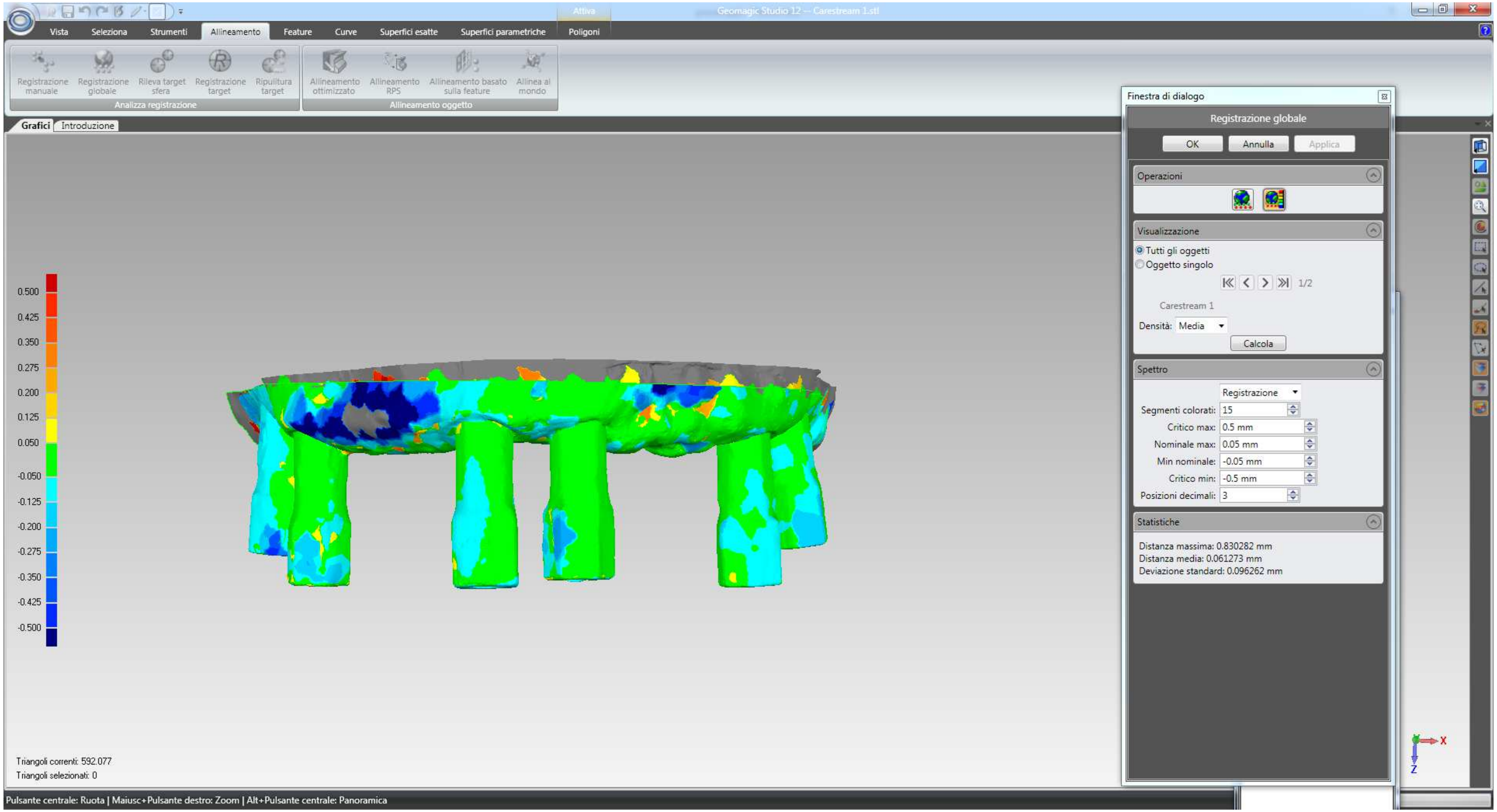

Care 1 vs REF (IMETRIC)

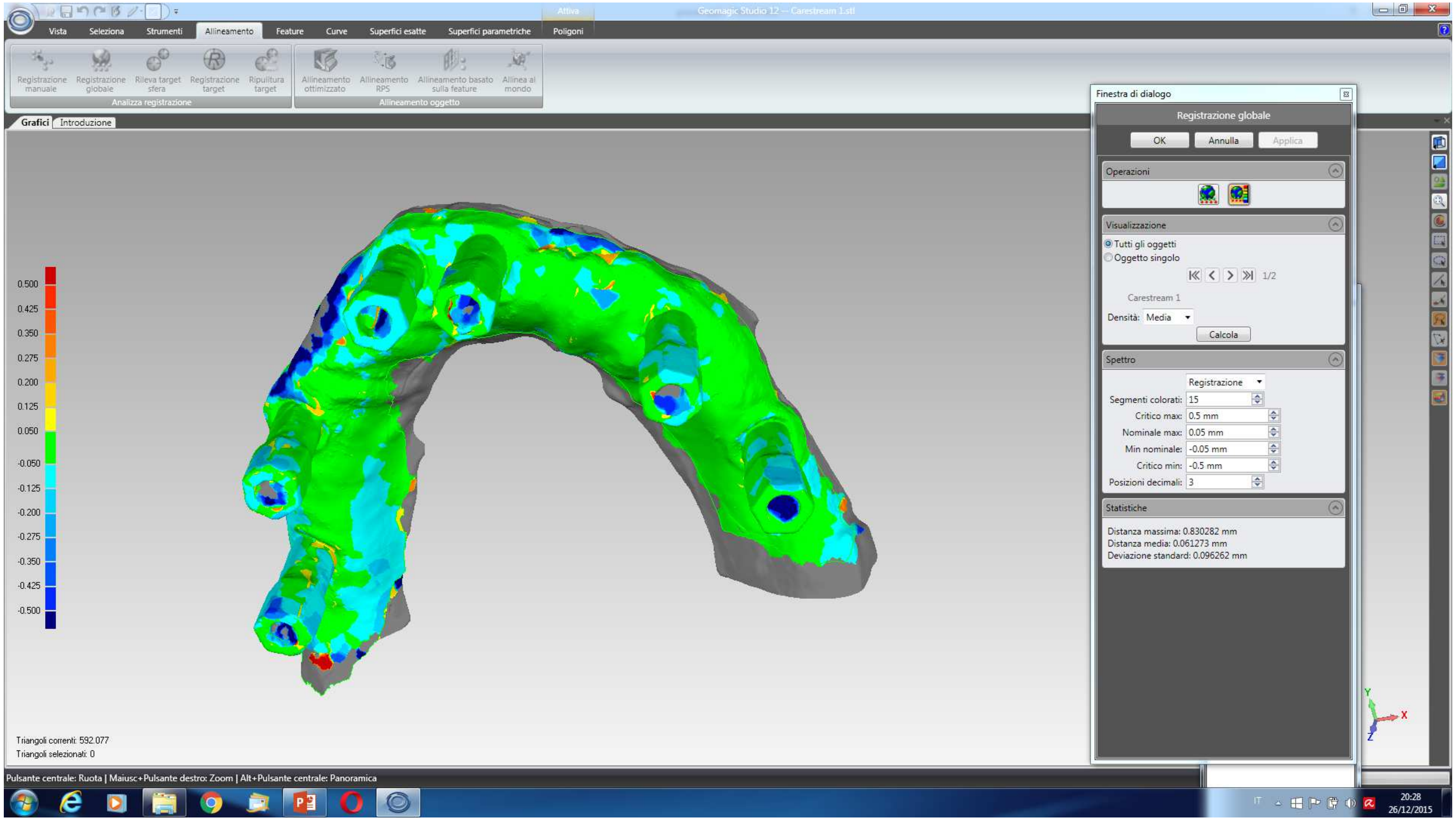

Care 1 vs REF (IMETRIC)

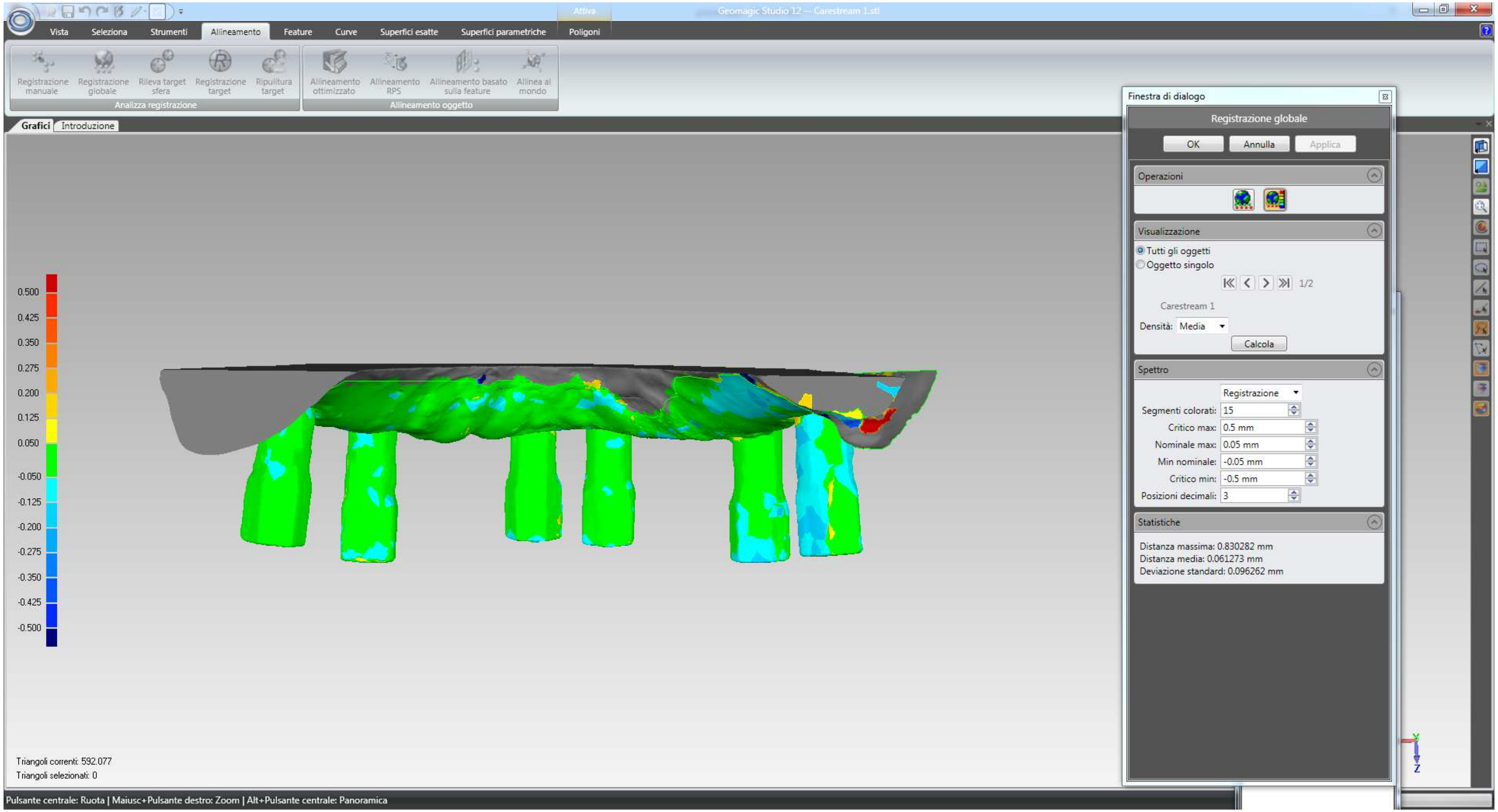

Care 2 vs REF (IMETRIC)

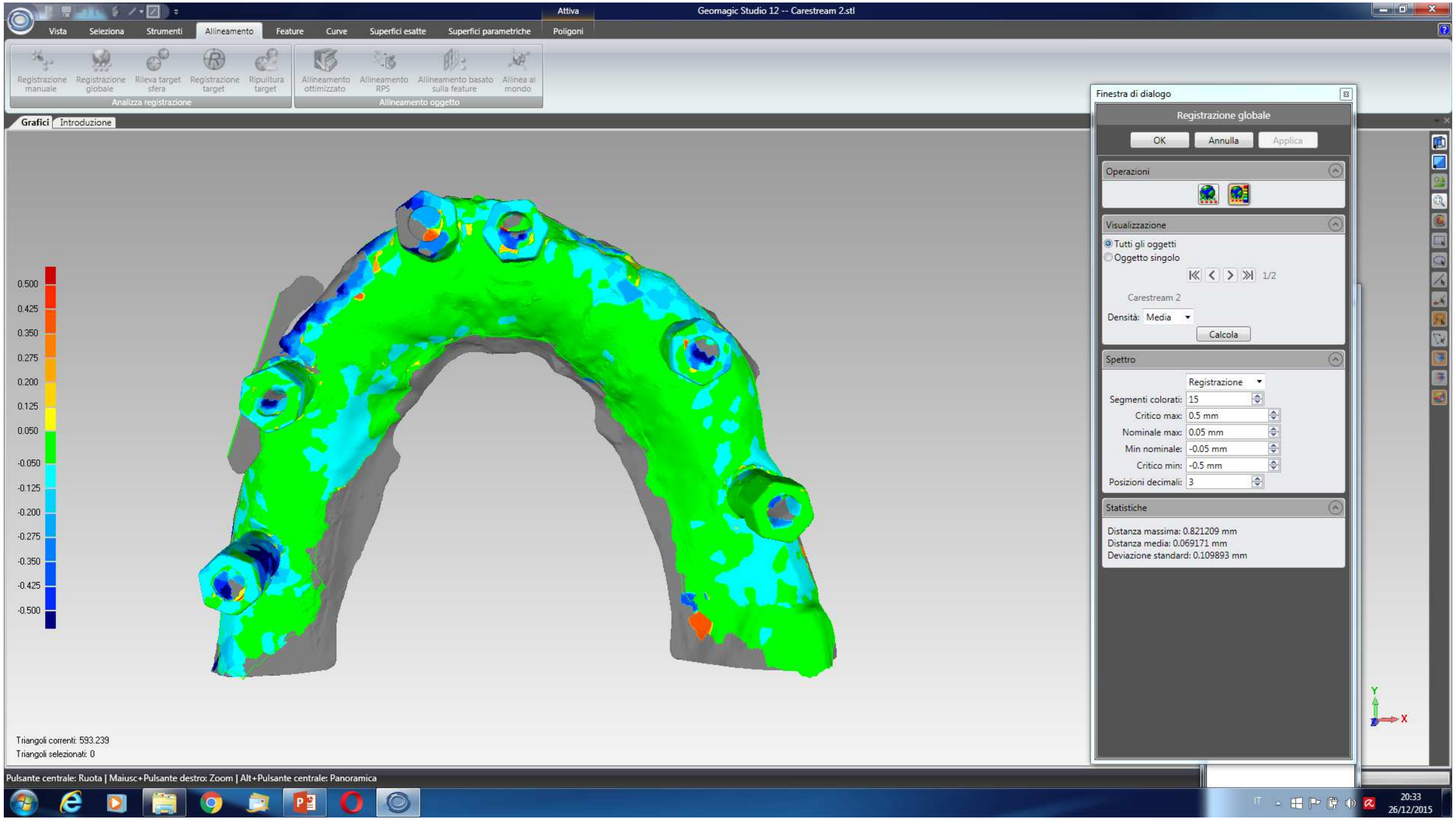

Care 2 vs REF (IMETRIC)

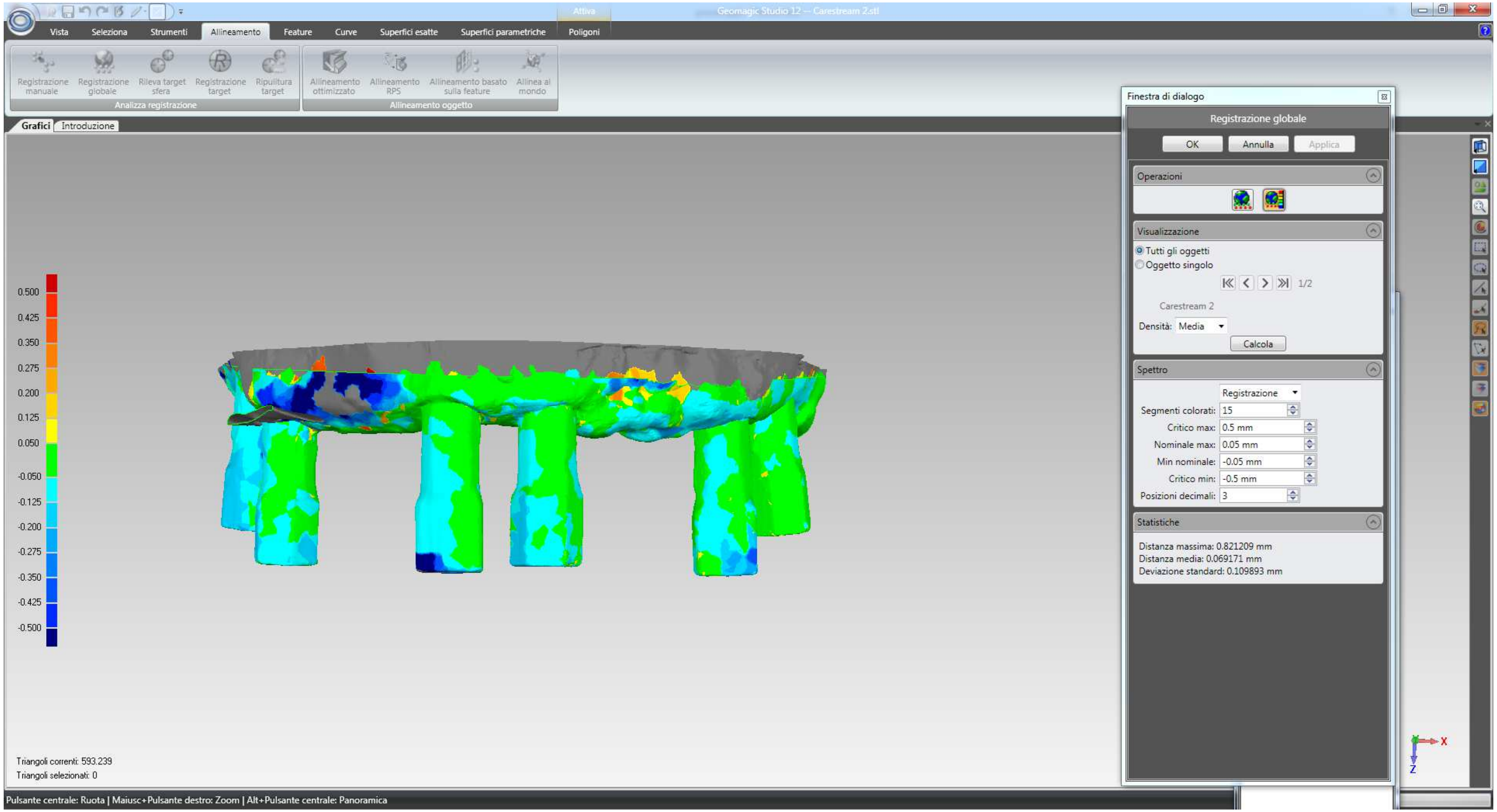

Care 2 vs REF (IMETRIC)

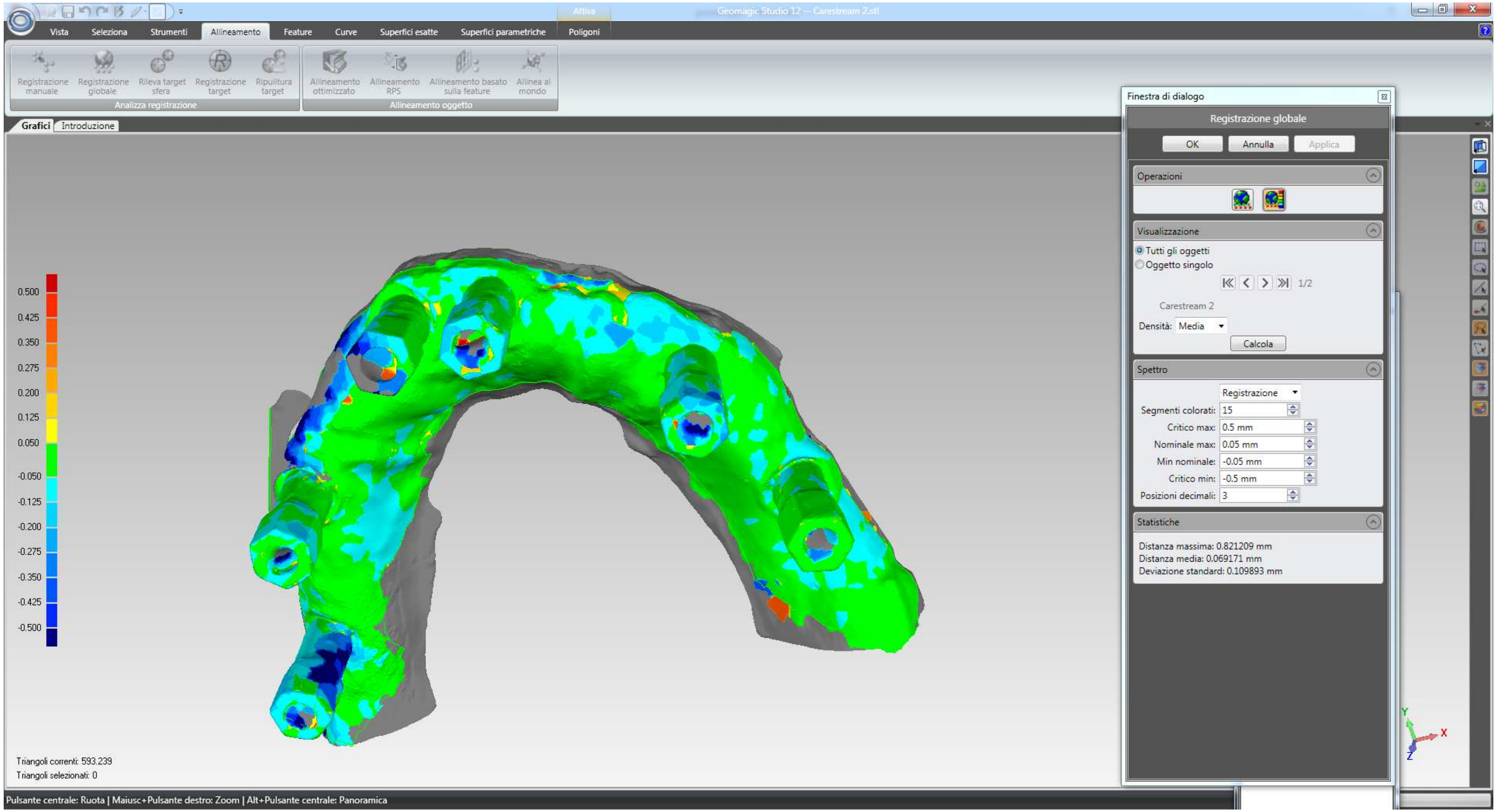

Care 2 vs REF (IMETRIC)

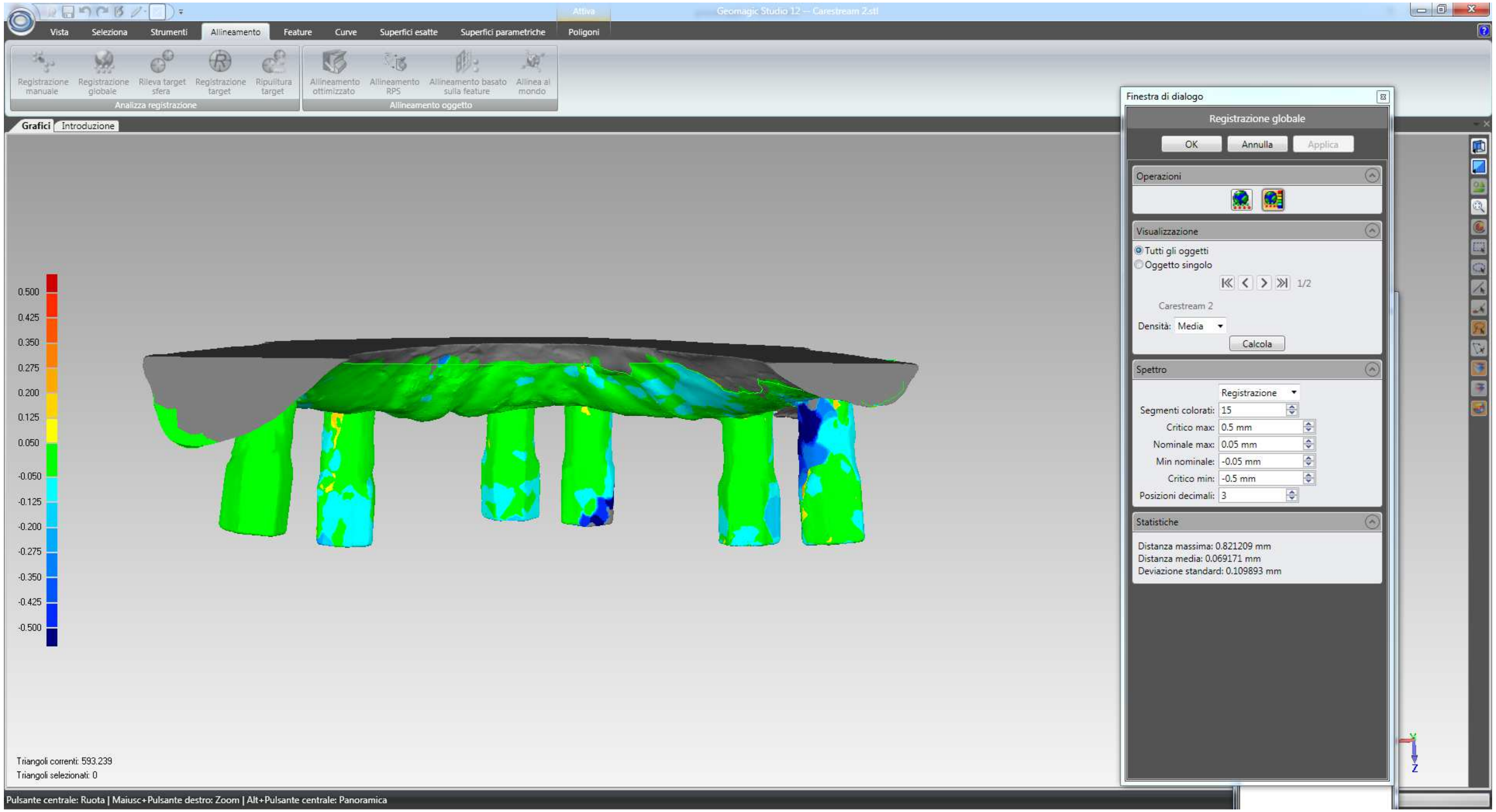

Care 3 vs REF (IMETRIC)

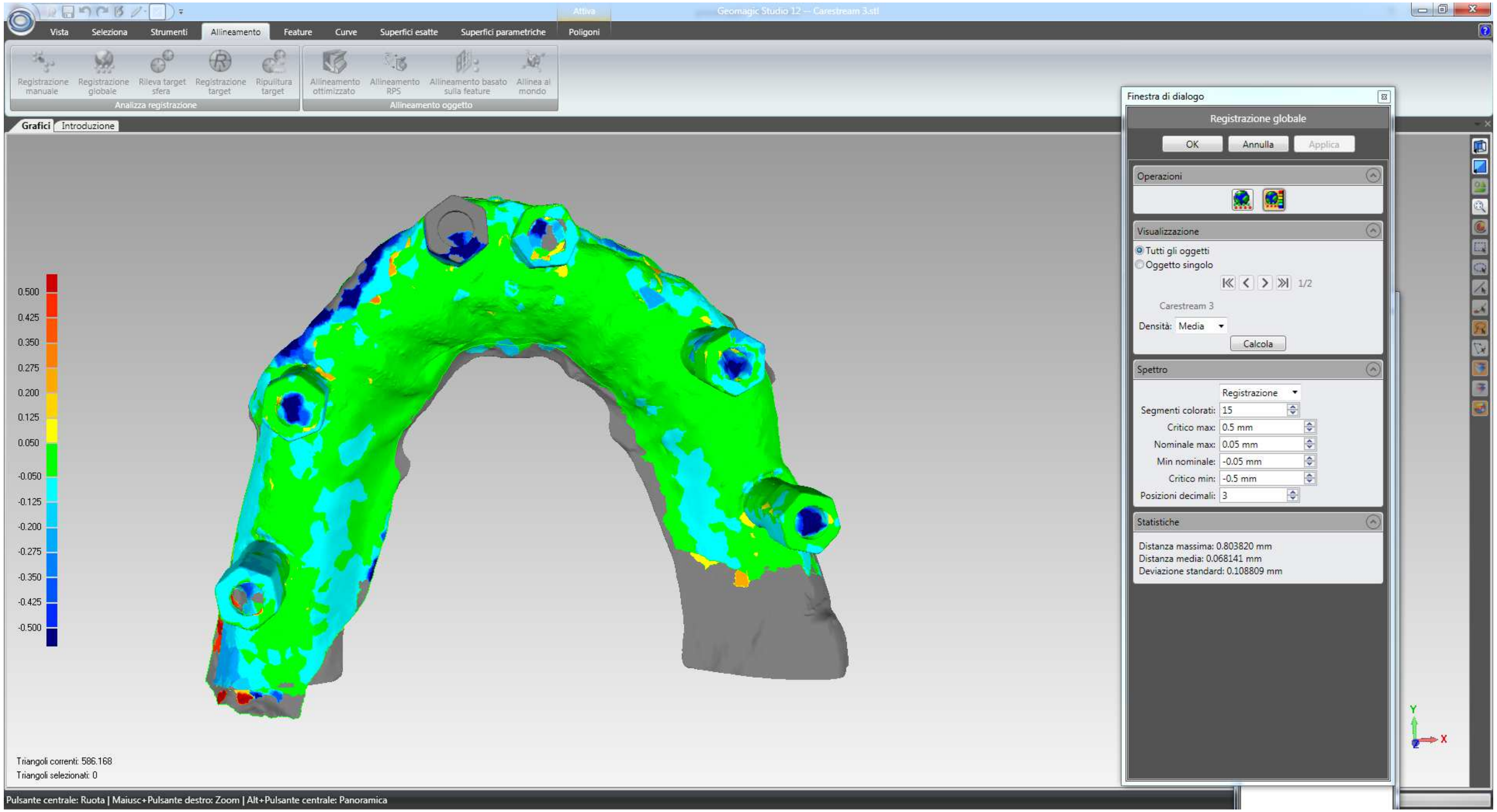

Care 3 vs REF (IMETRIC)

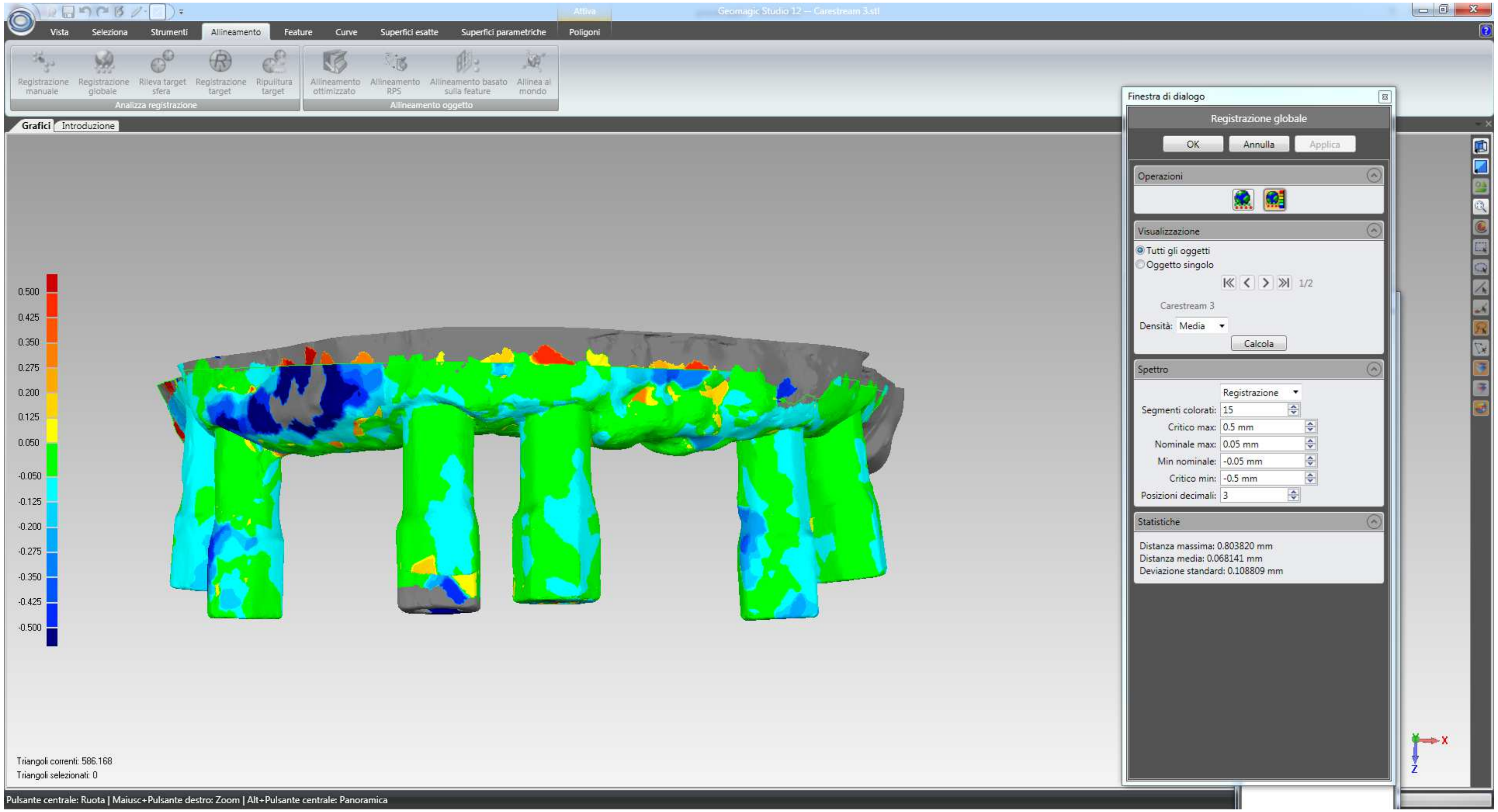

Care 3 vs REF (IMETRIC)

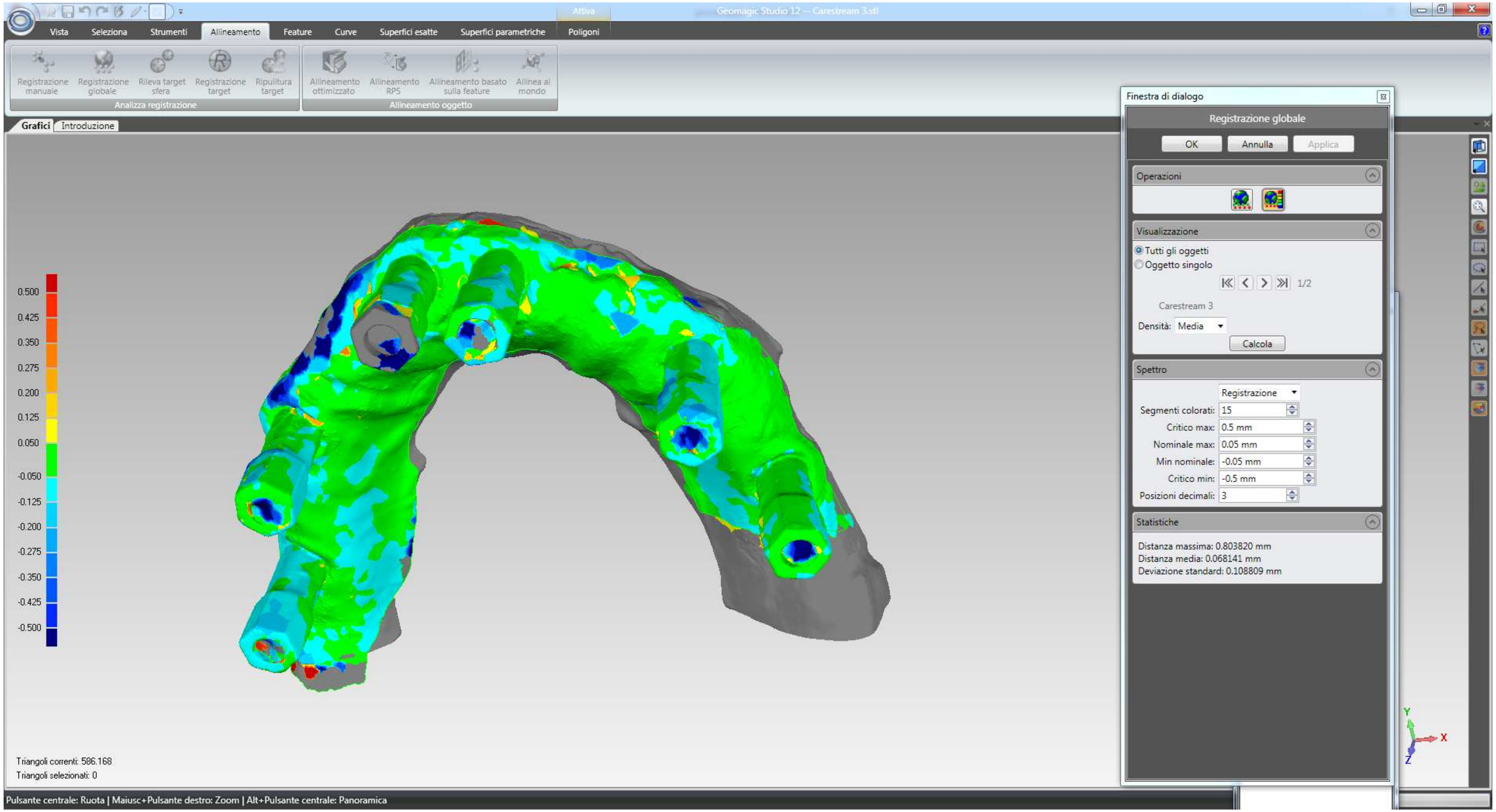

Care 3 vs REF (IMETRIC)

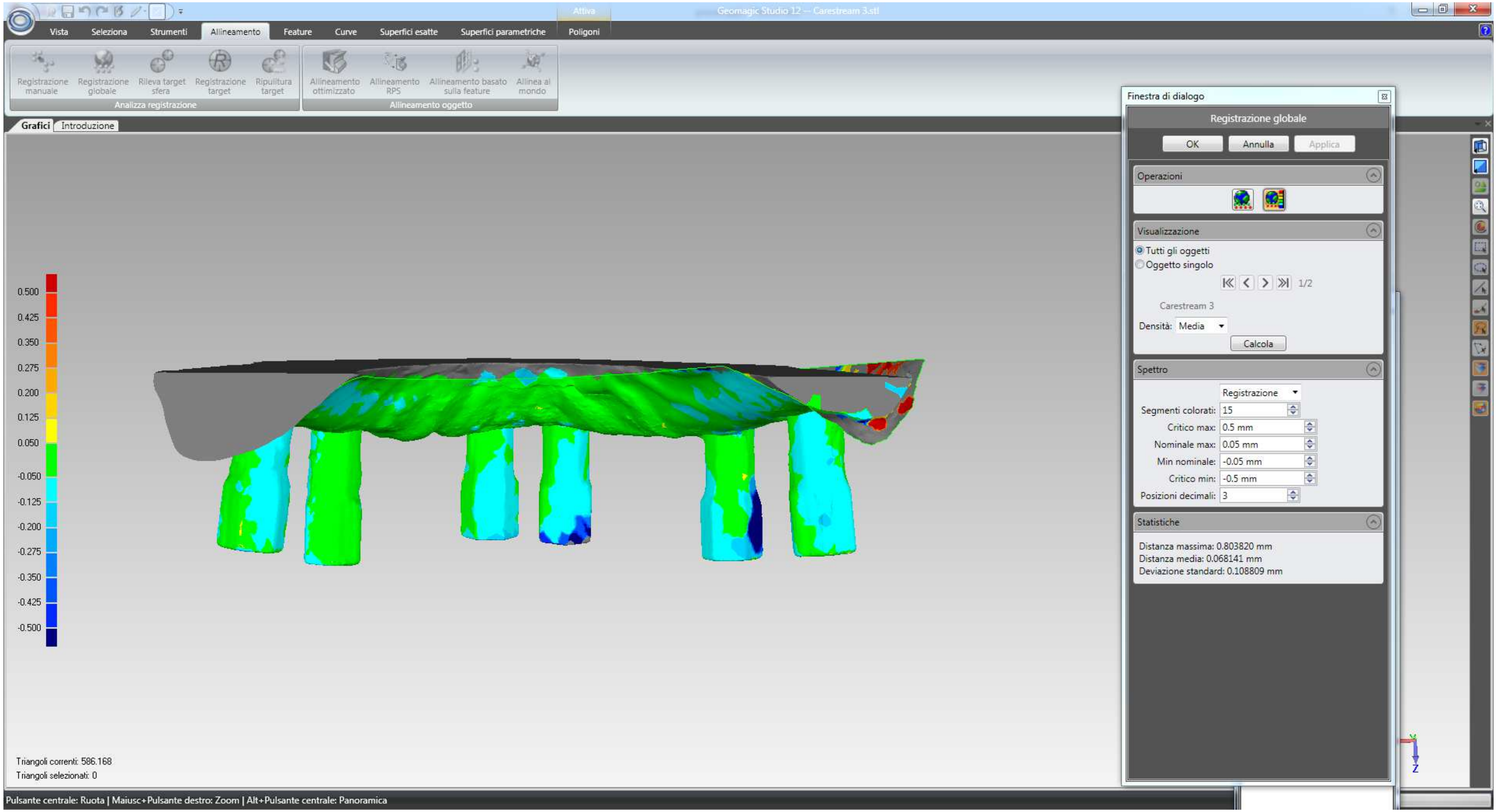

Care 4 vs REF (IMETRIC)

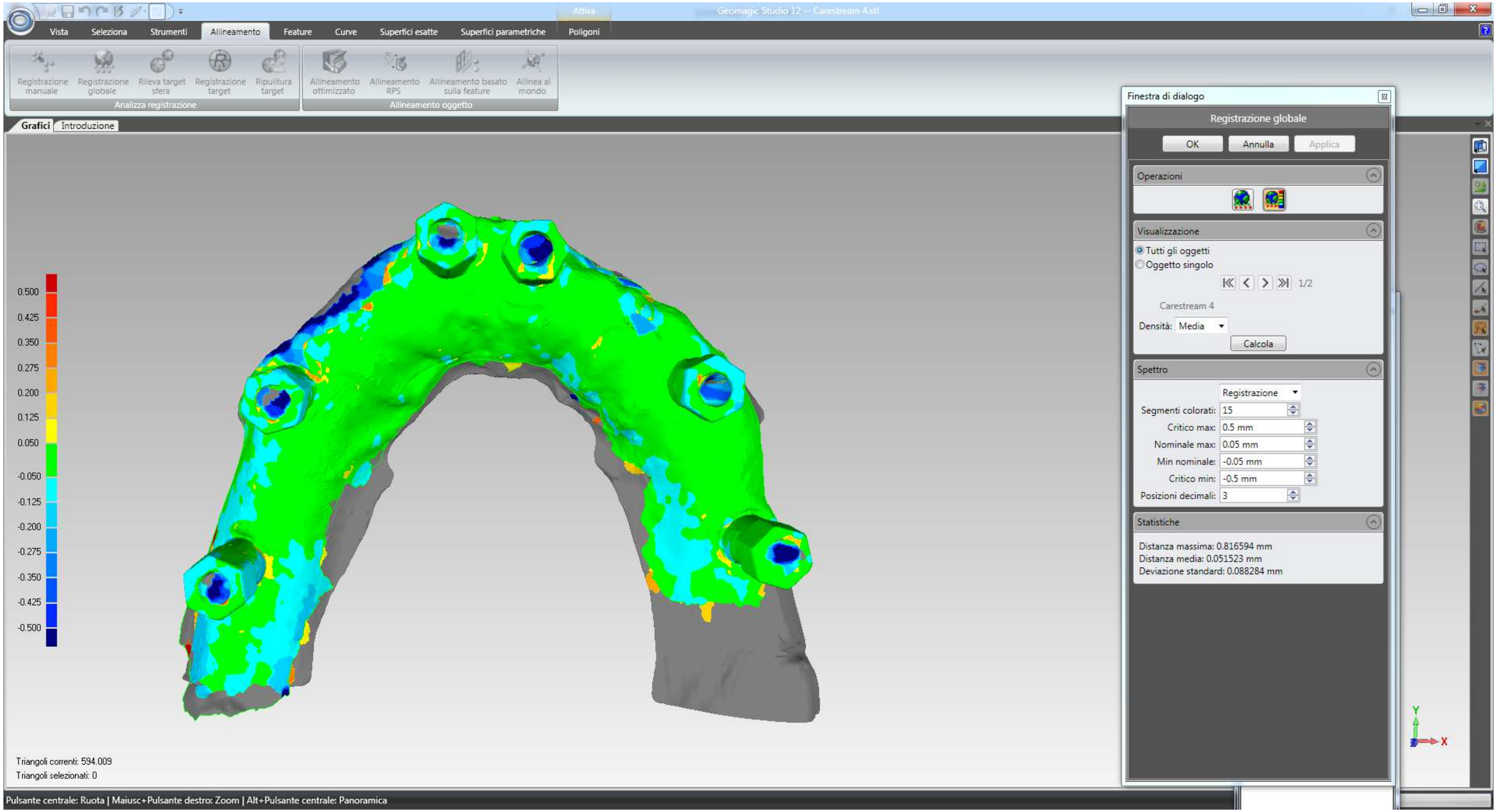

Care 4 vs REF (IMETRIC)

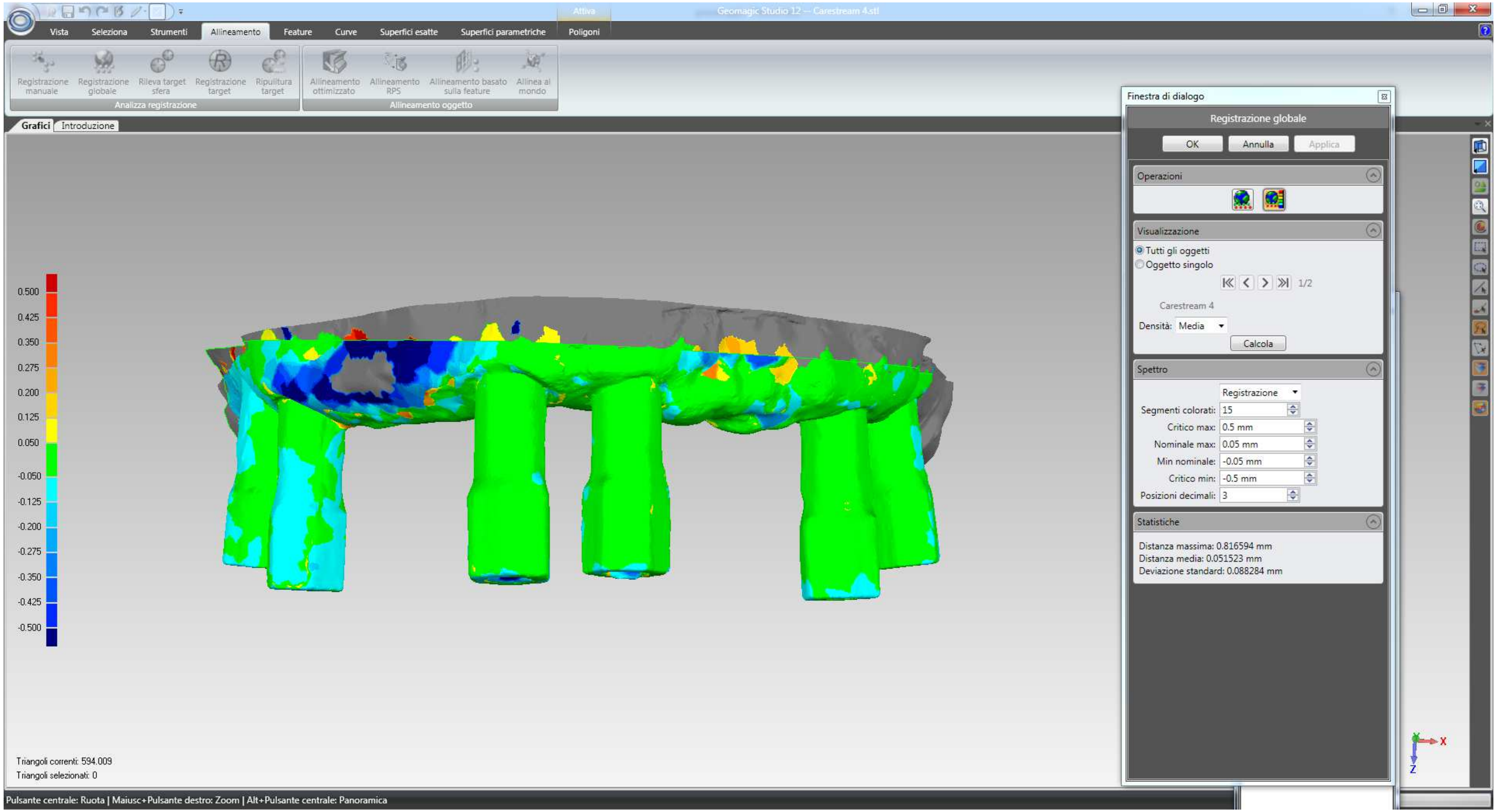

Care 4 vs REF (IMETRIC)

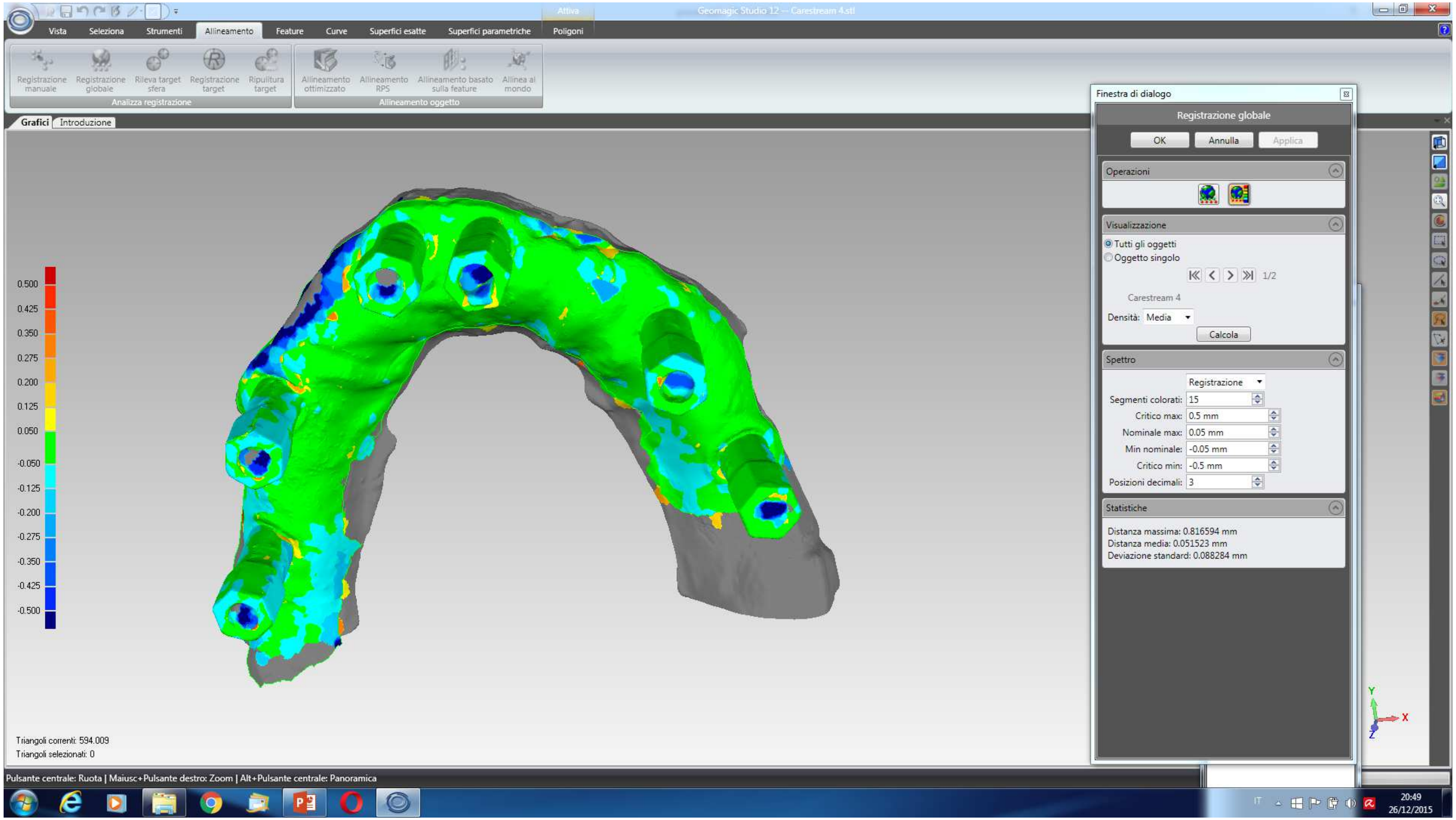

Care 4 vs REF (IMETRIC)

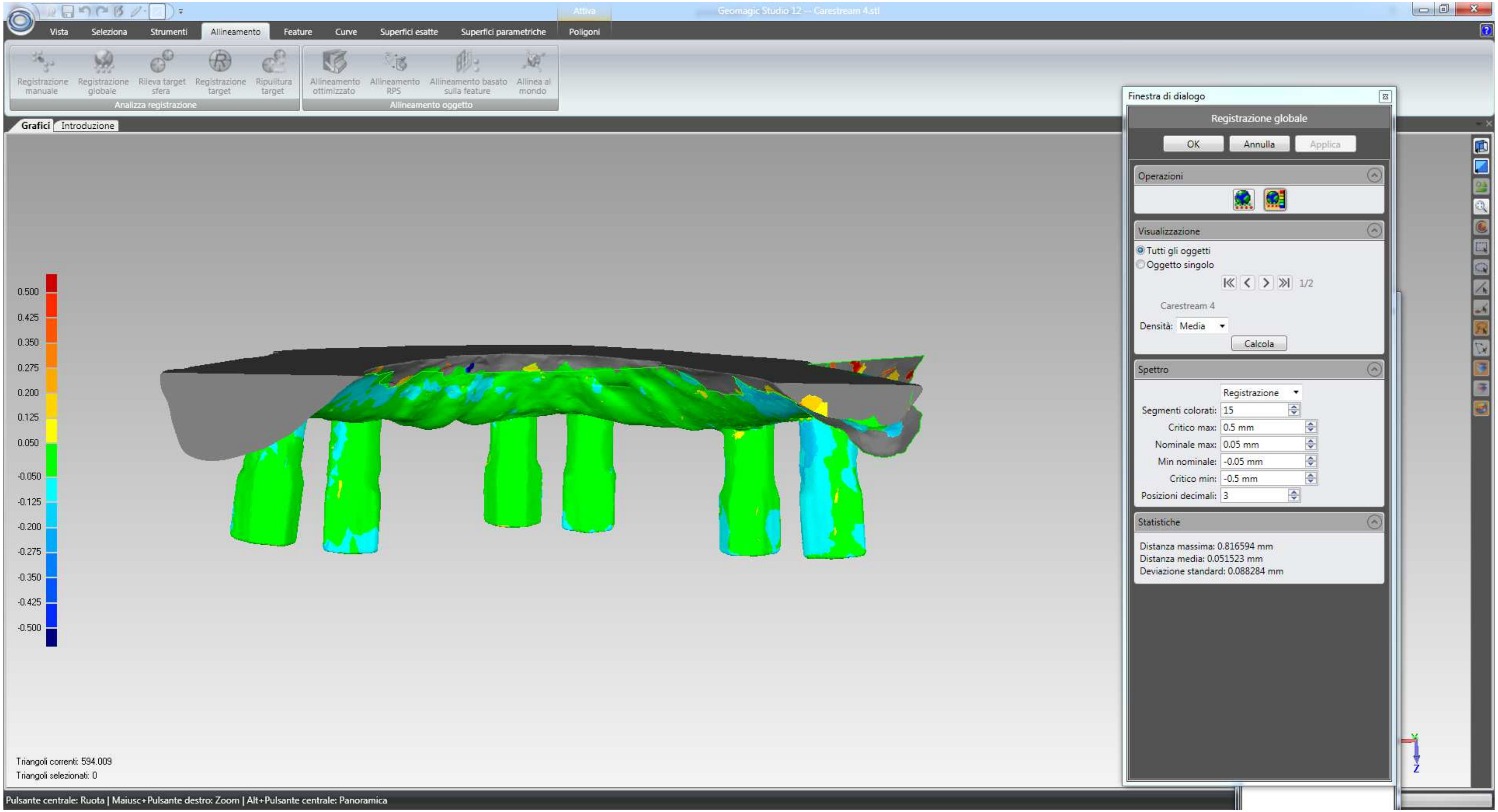

Care 5 vs REF (IMETRIC)

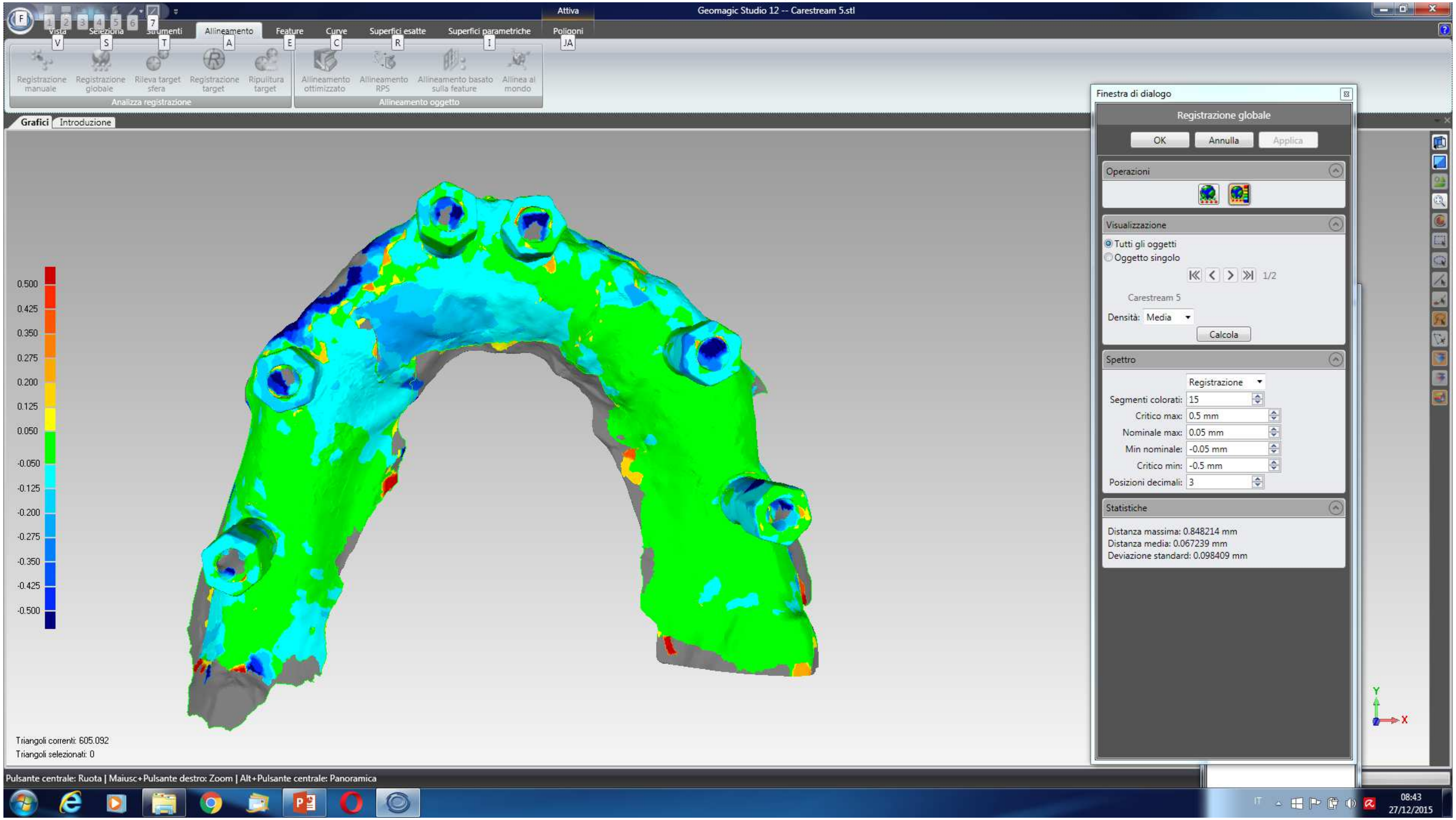

Care 5 vs REF (IMETRIC)

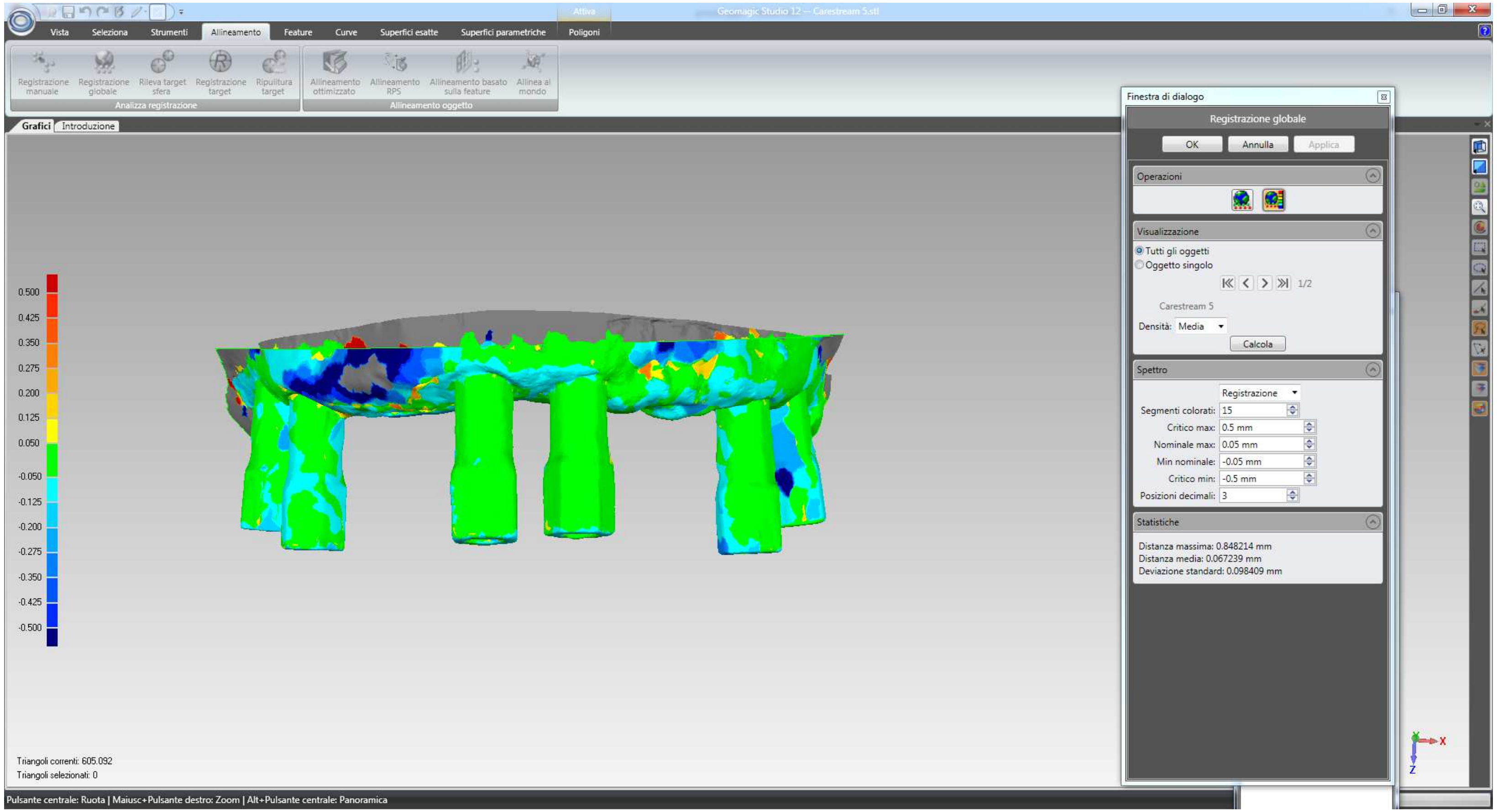

Care 5 vs REF (IMETRIC)

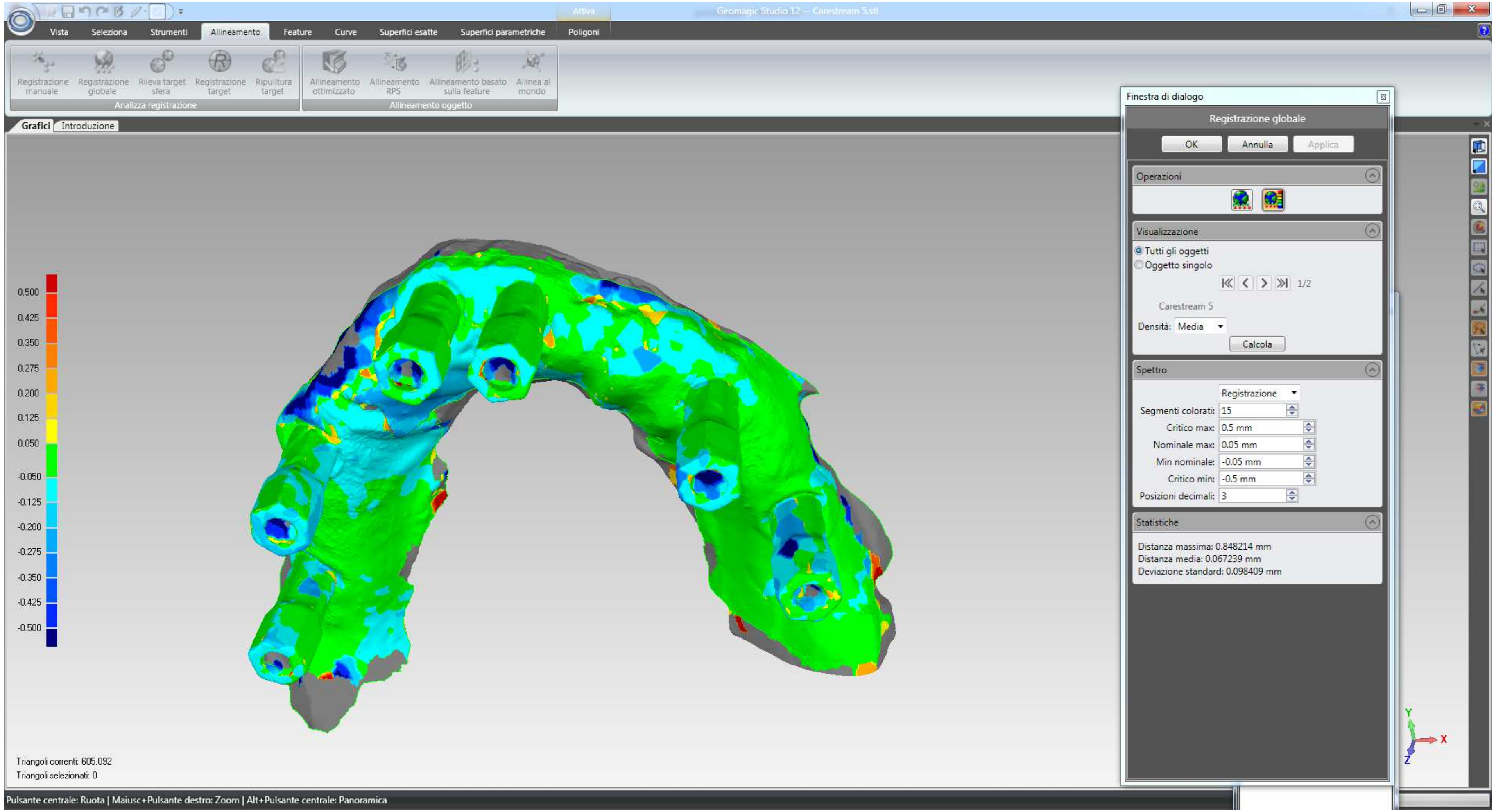

Care 5 vs REF (IMETRIC)

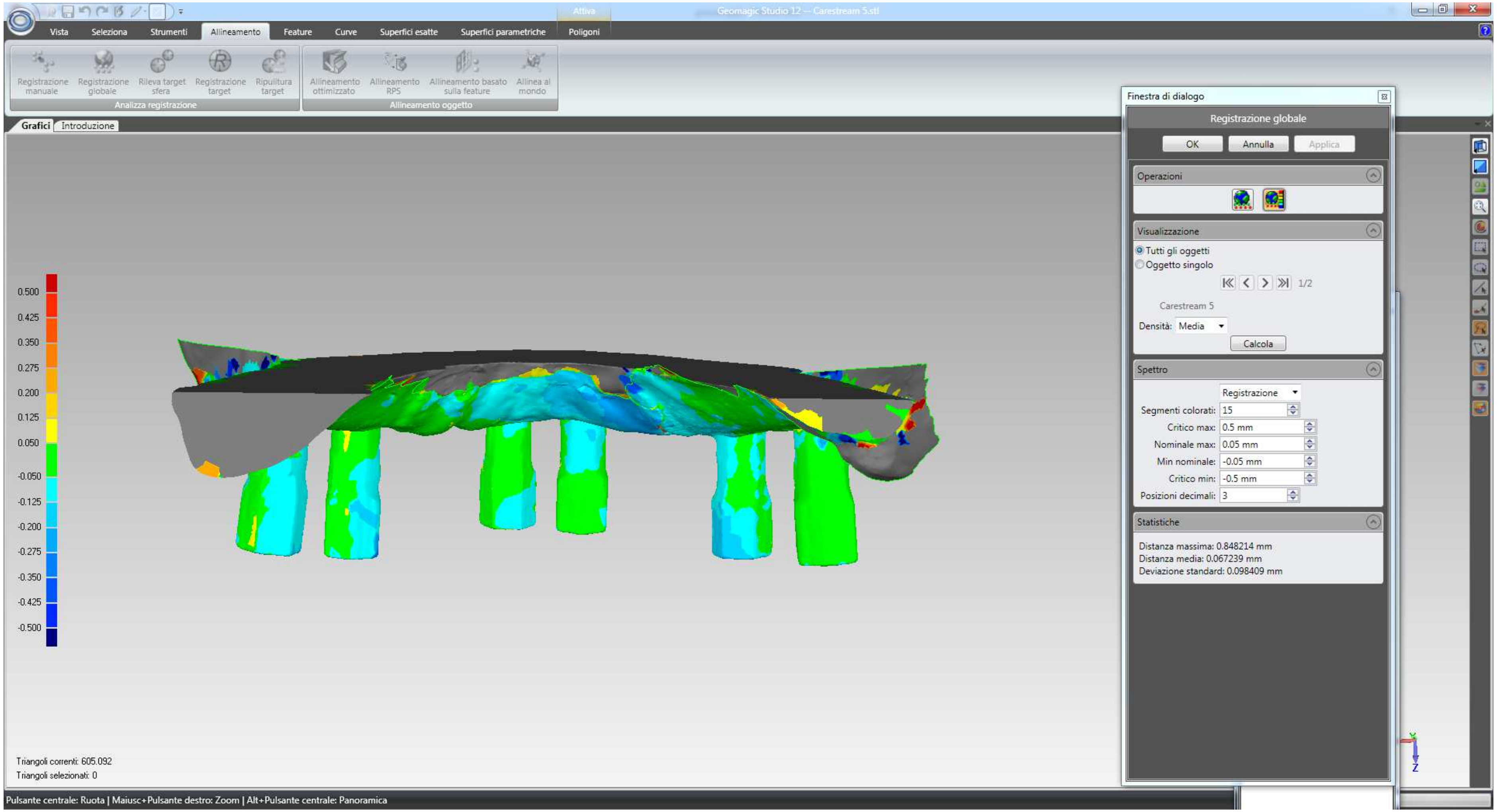

General trueness of the Carestream intraoral scanner in the totally edentulous model

	Mean distance	SD	Maximum distance
Carestream 1	0.061	0.096	0.830
Carestream 2	0.069	0.109	0.821
Carestream 3	0.068	0.108	0.803
Carestream 4	0.051	0.088	0.816
Carestream 5	0.067	0.098	0.848

Overall Care general accuracy: 0.063 (0.007)

Plan 1 vs REF (IMETRIC)

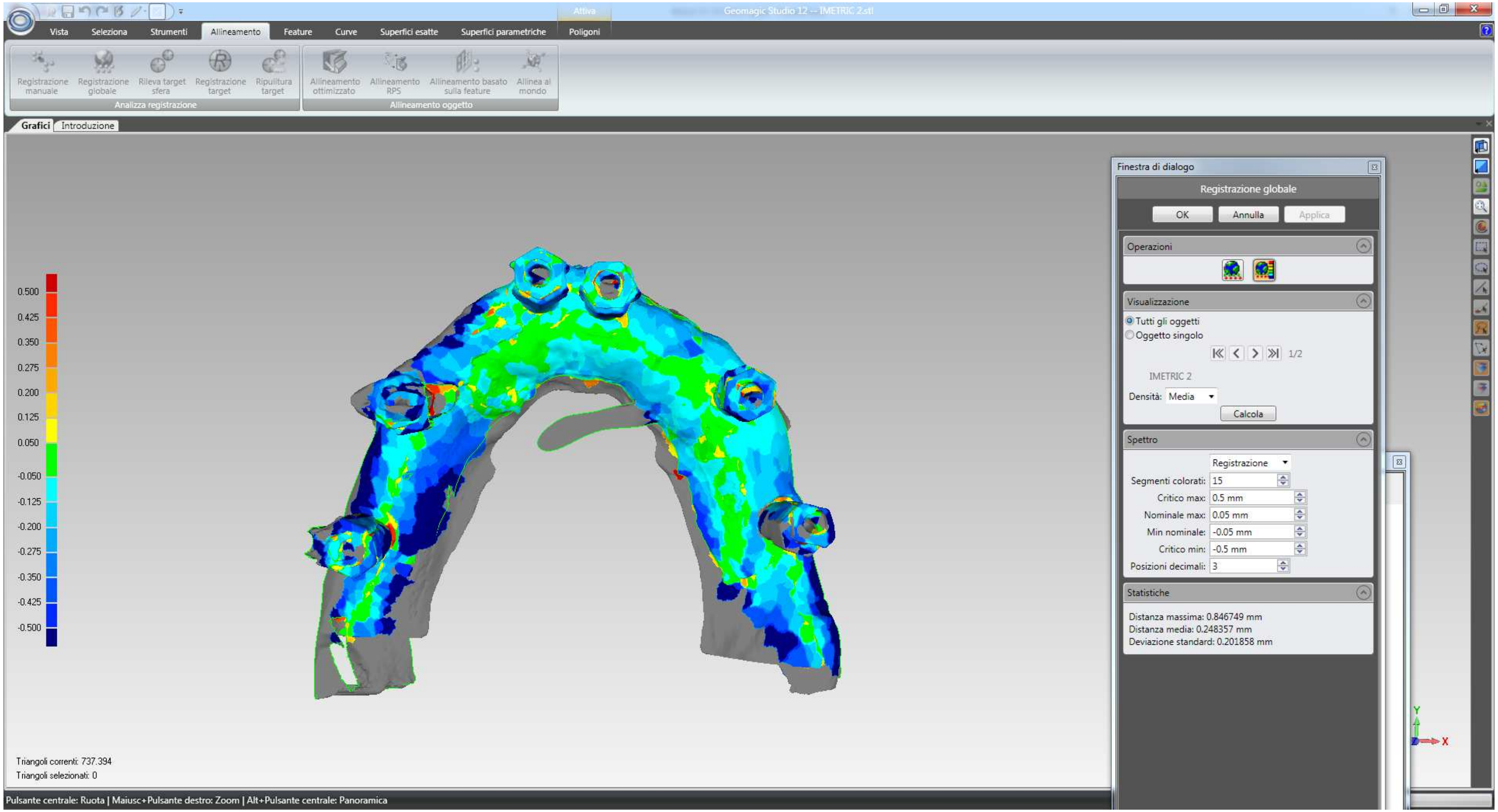

Plan 1 vs REF (IMETRIC)

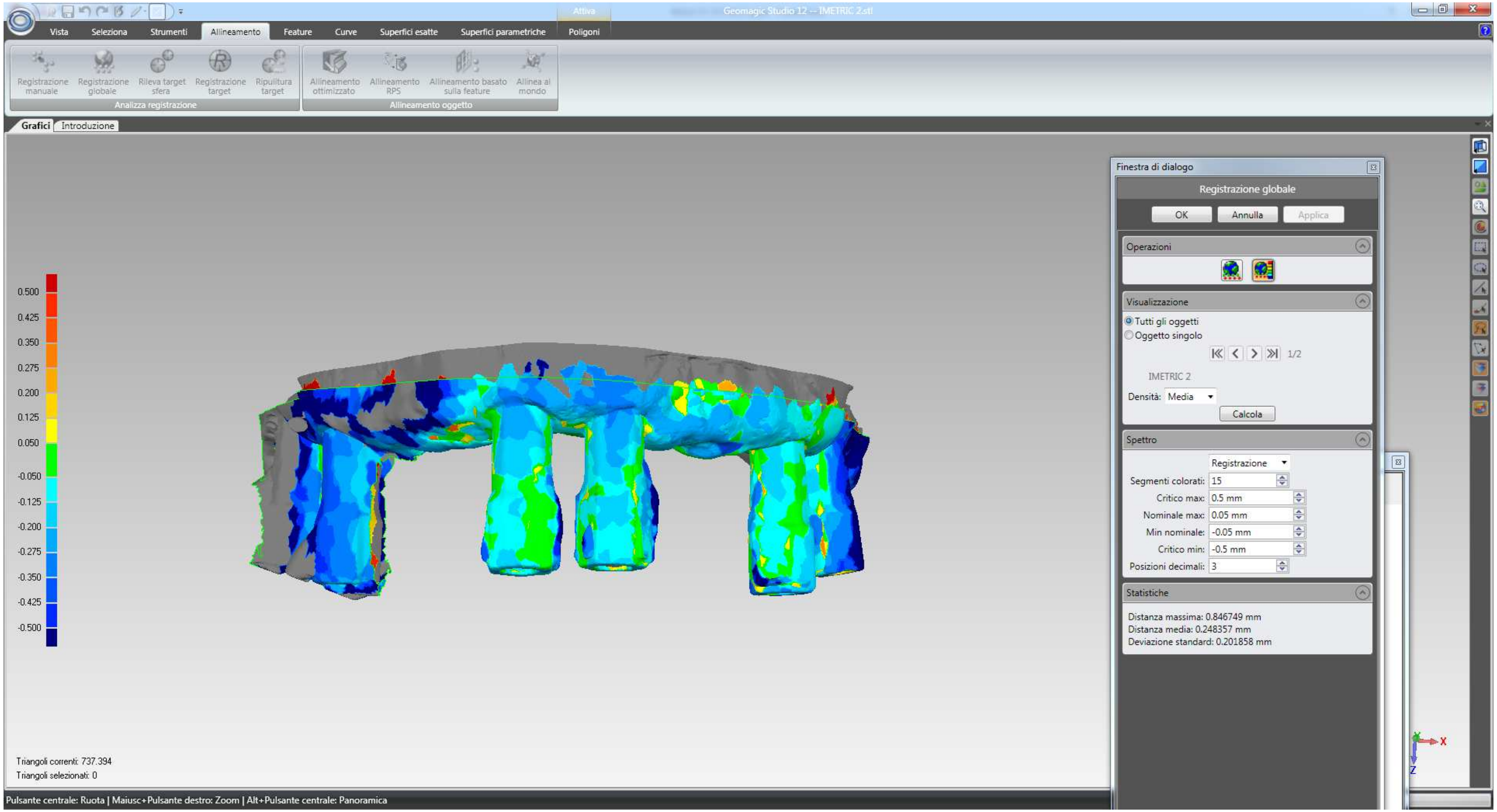

Plan 1 vs REF (IMETRIC)

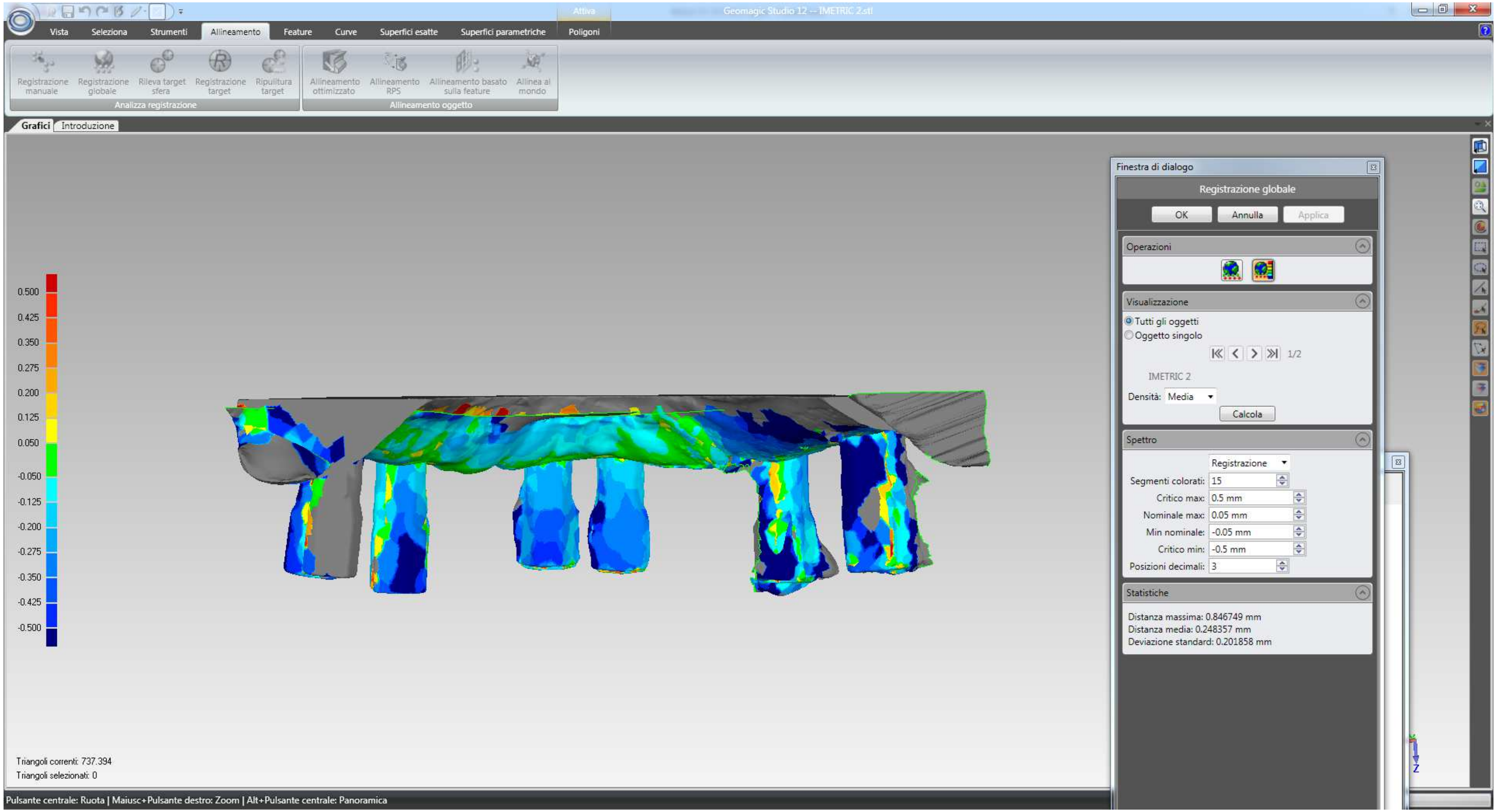

Plan 1 vs REF (IMETRIC)

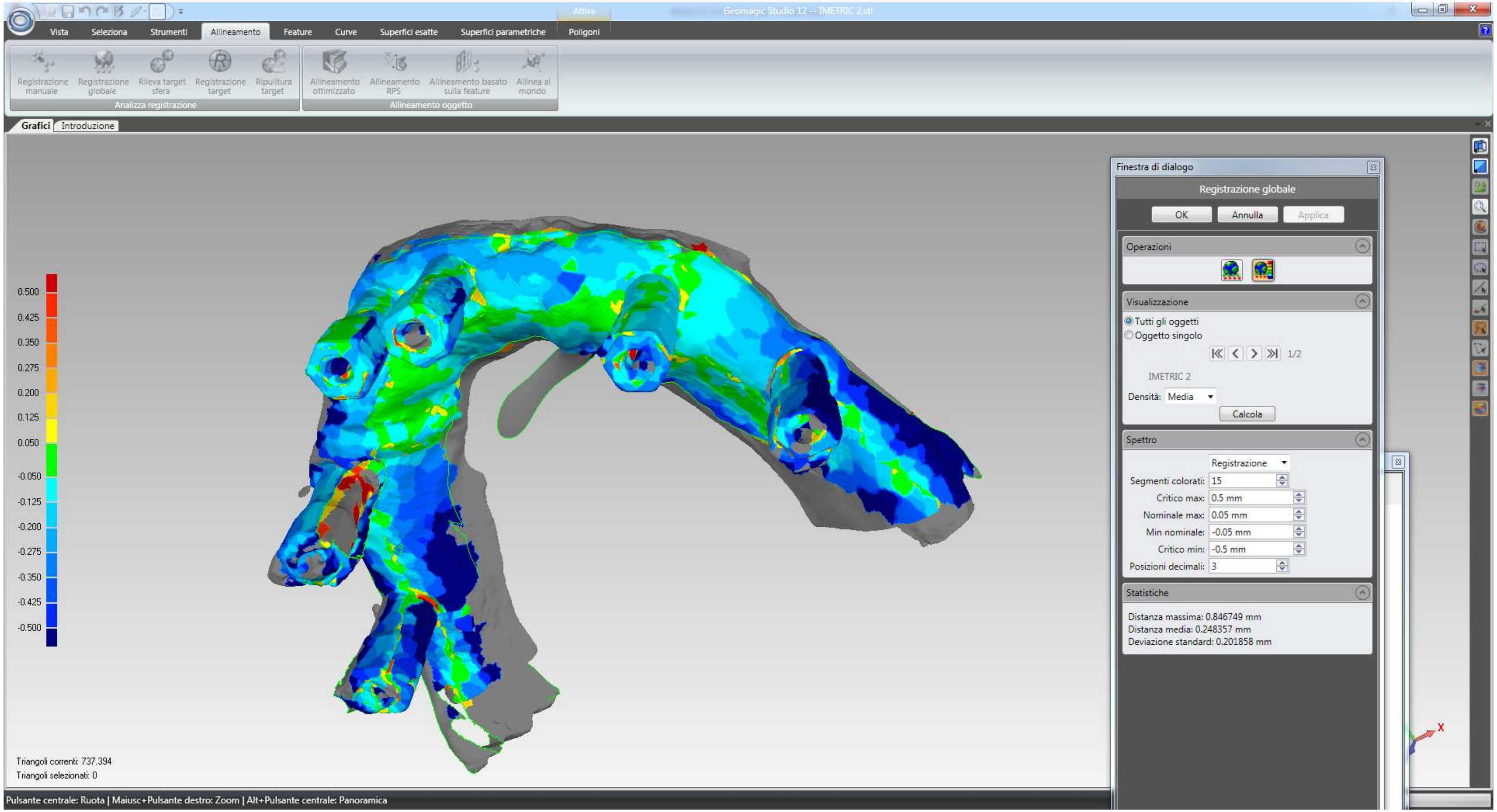

Plan 2 vs REF (IMETRIC)

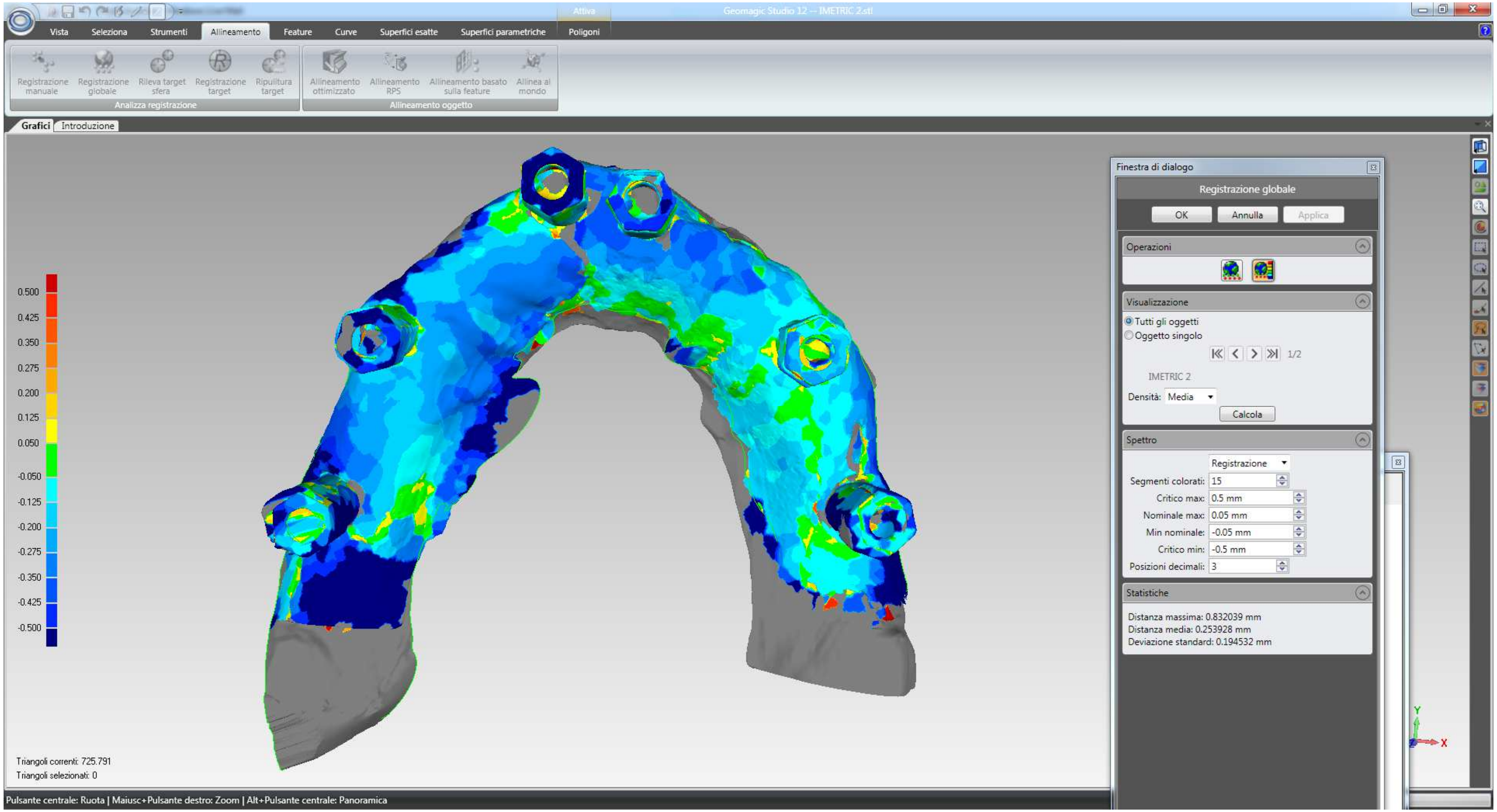

Plan 2 vs REF (IMETRIC)

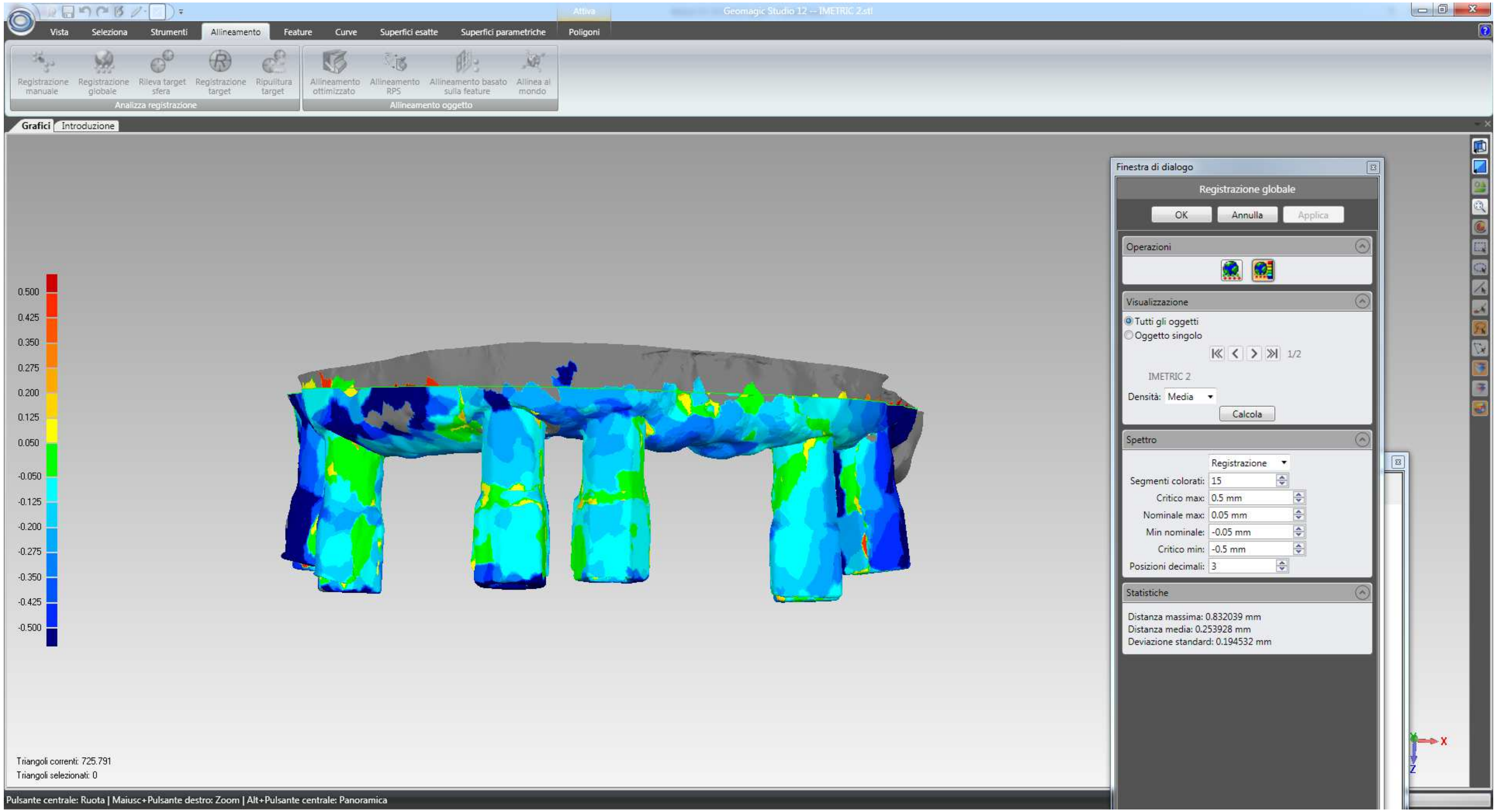

Plan 2 vs REF (IMETRIC)

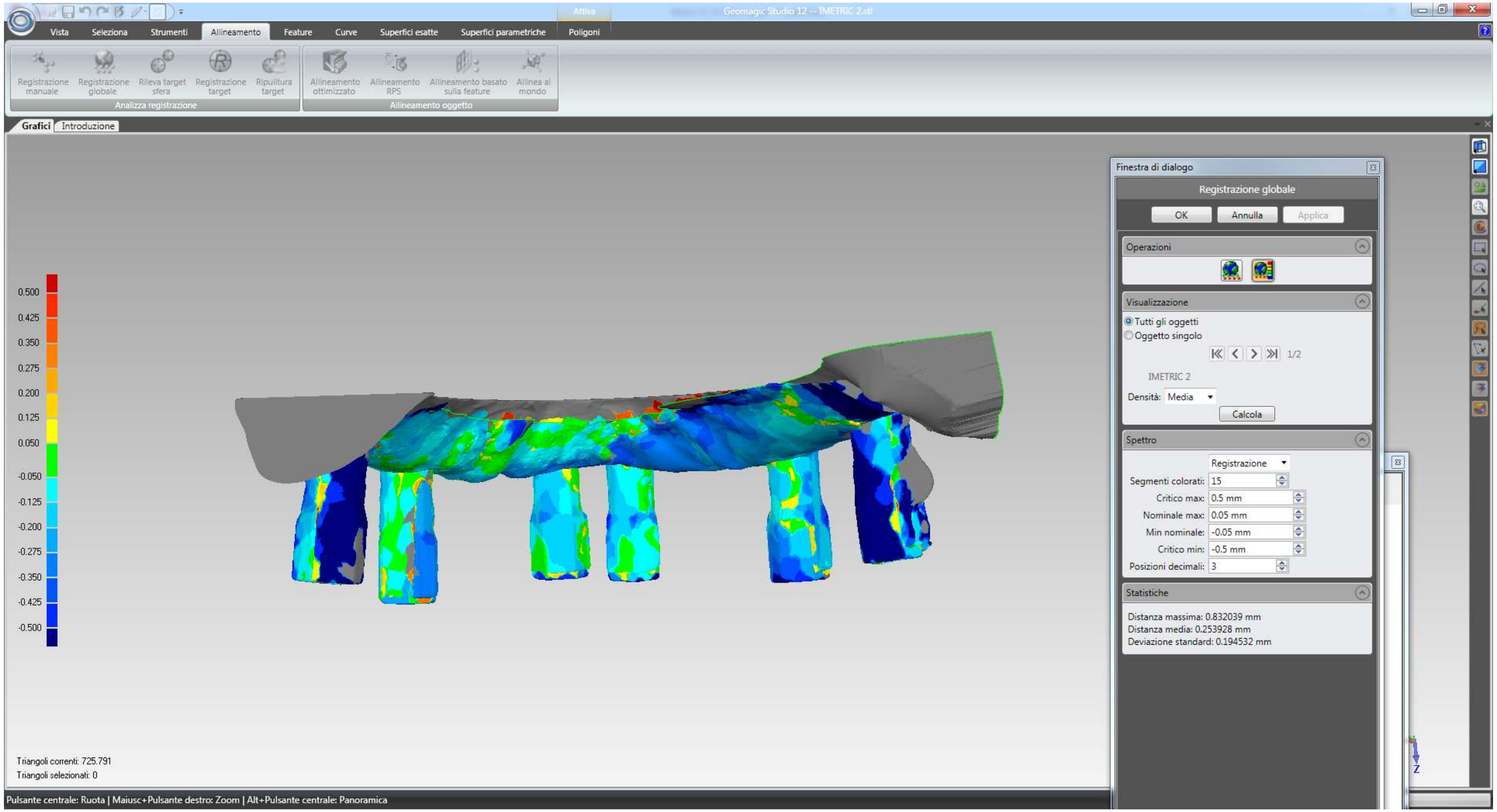

Plan 2 vs REF (IMETRIC)

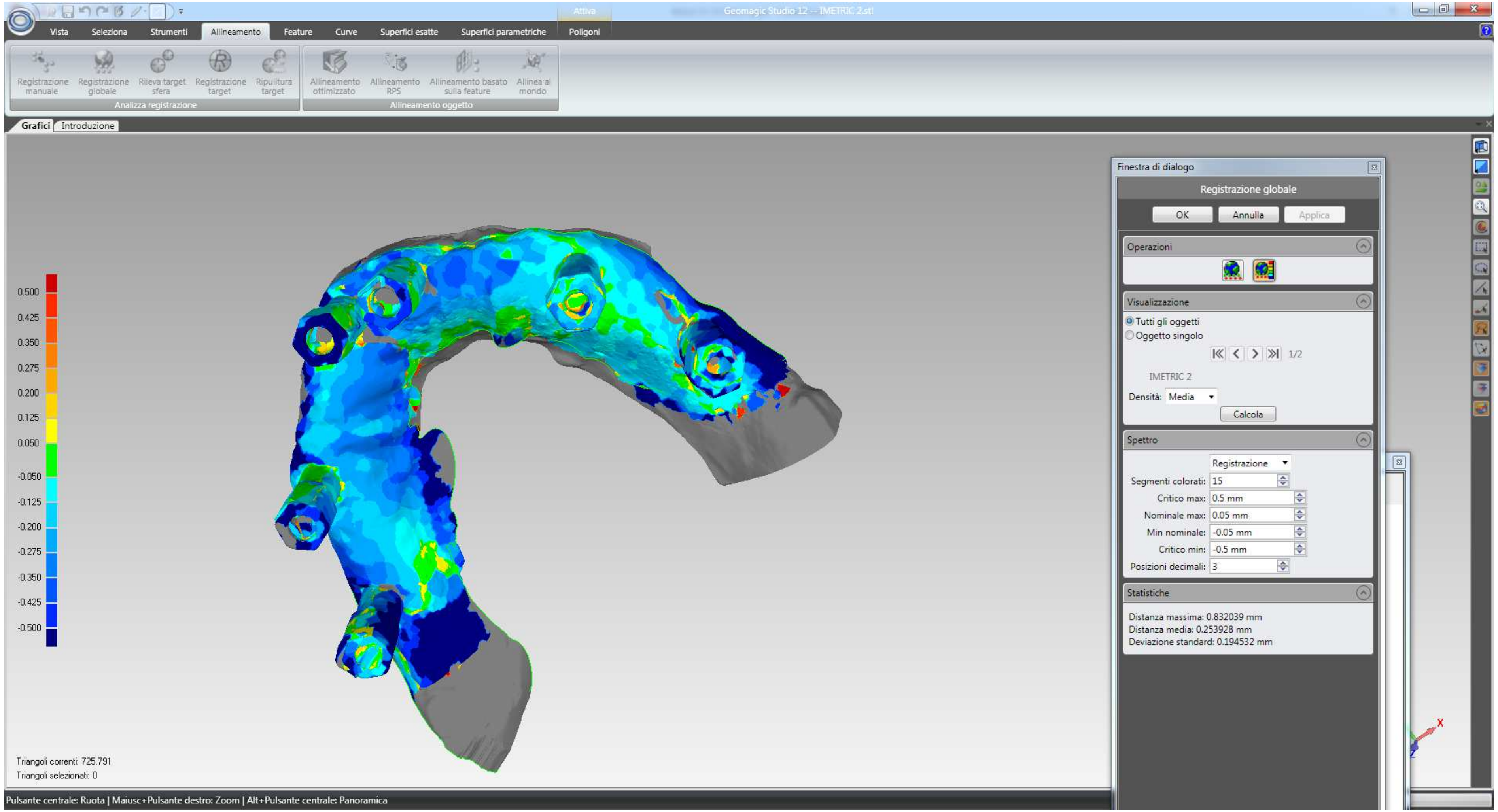

Plan 3 vs REF (IMETRIC)

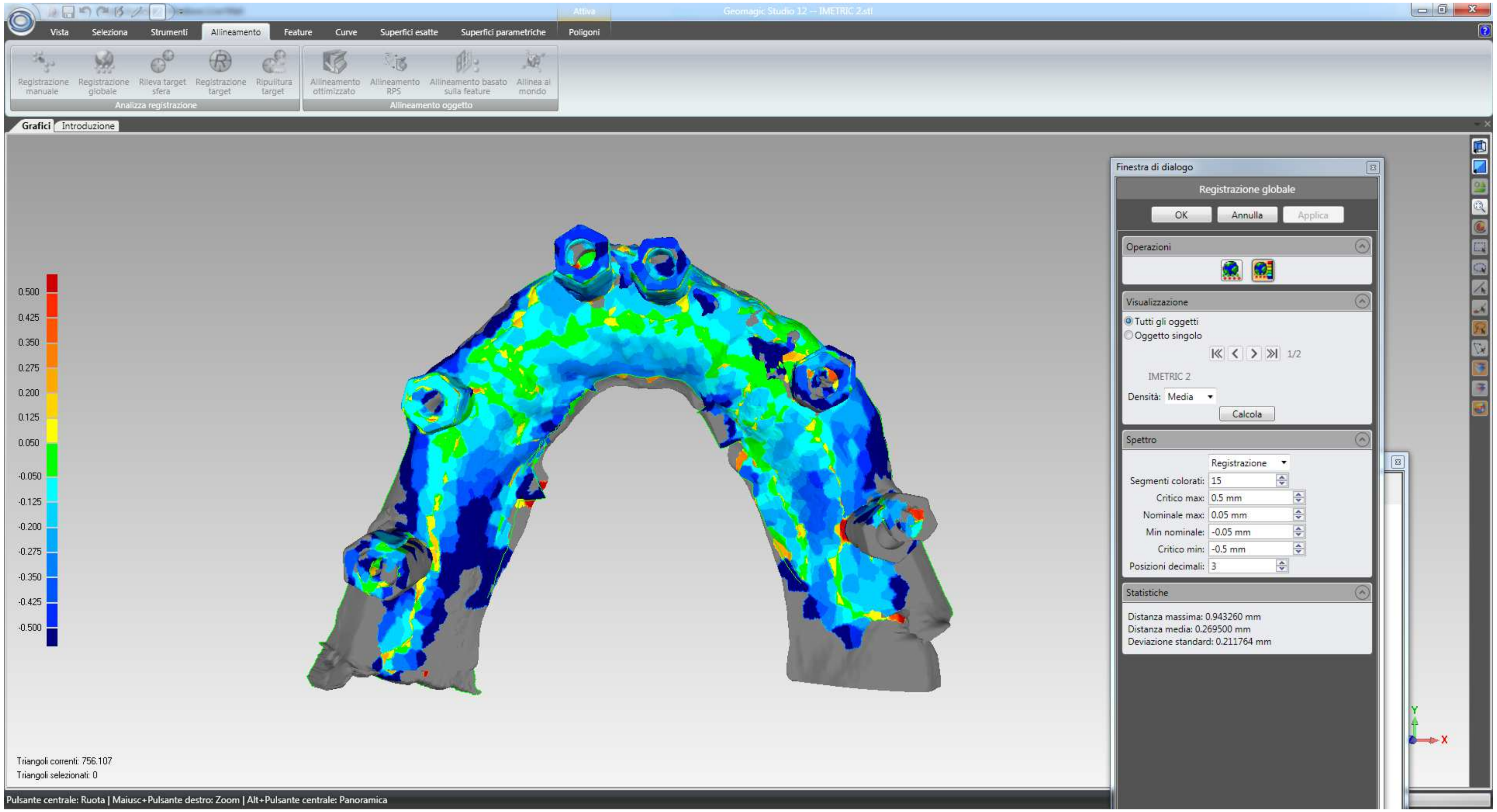

Plan 3 vs REF (IMETRIC)

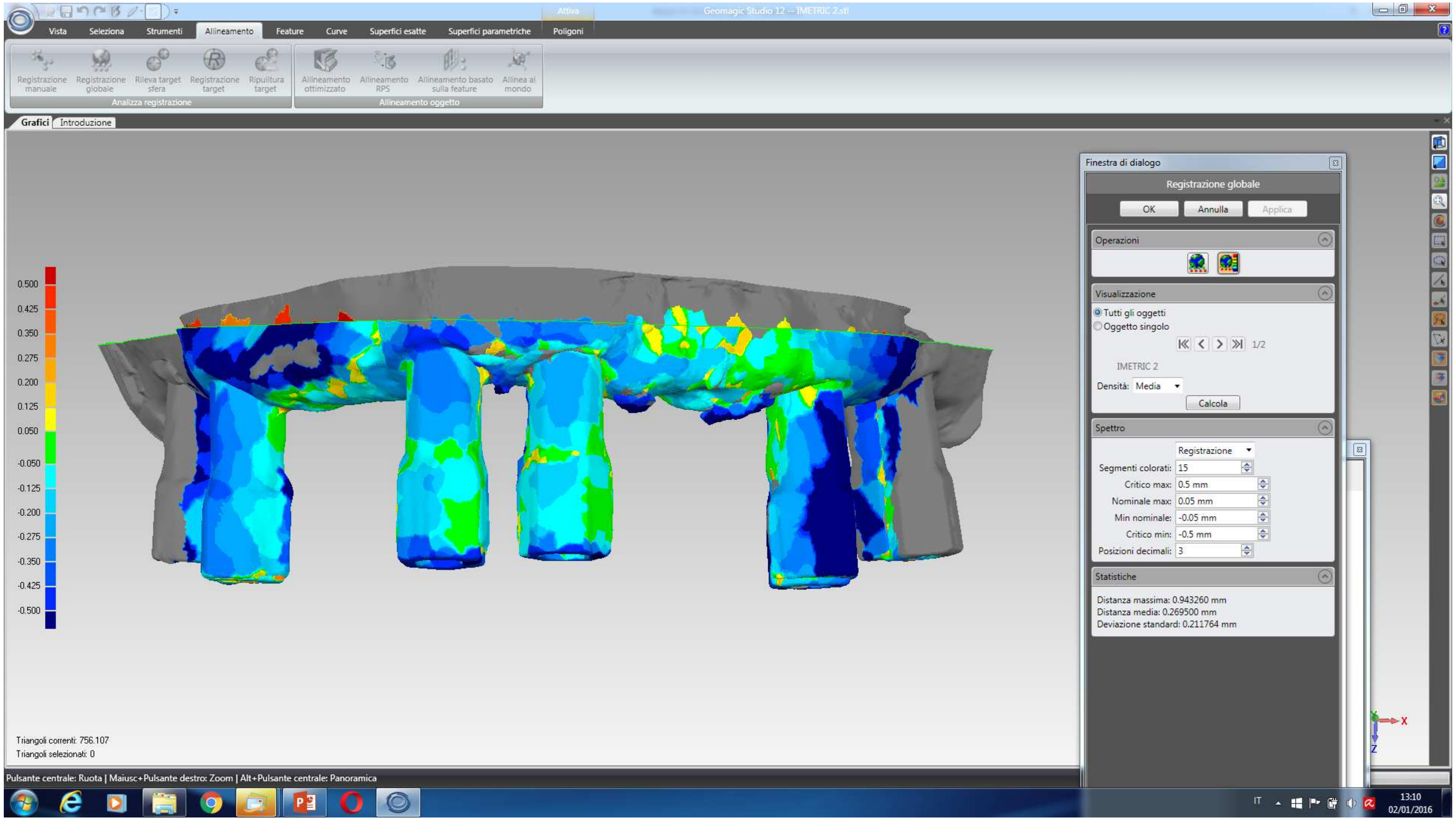

Plan 3 vs REF (IMETRIC)

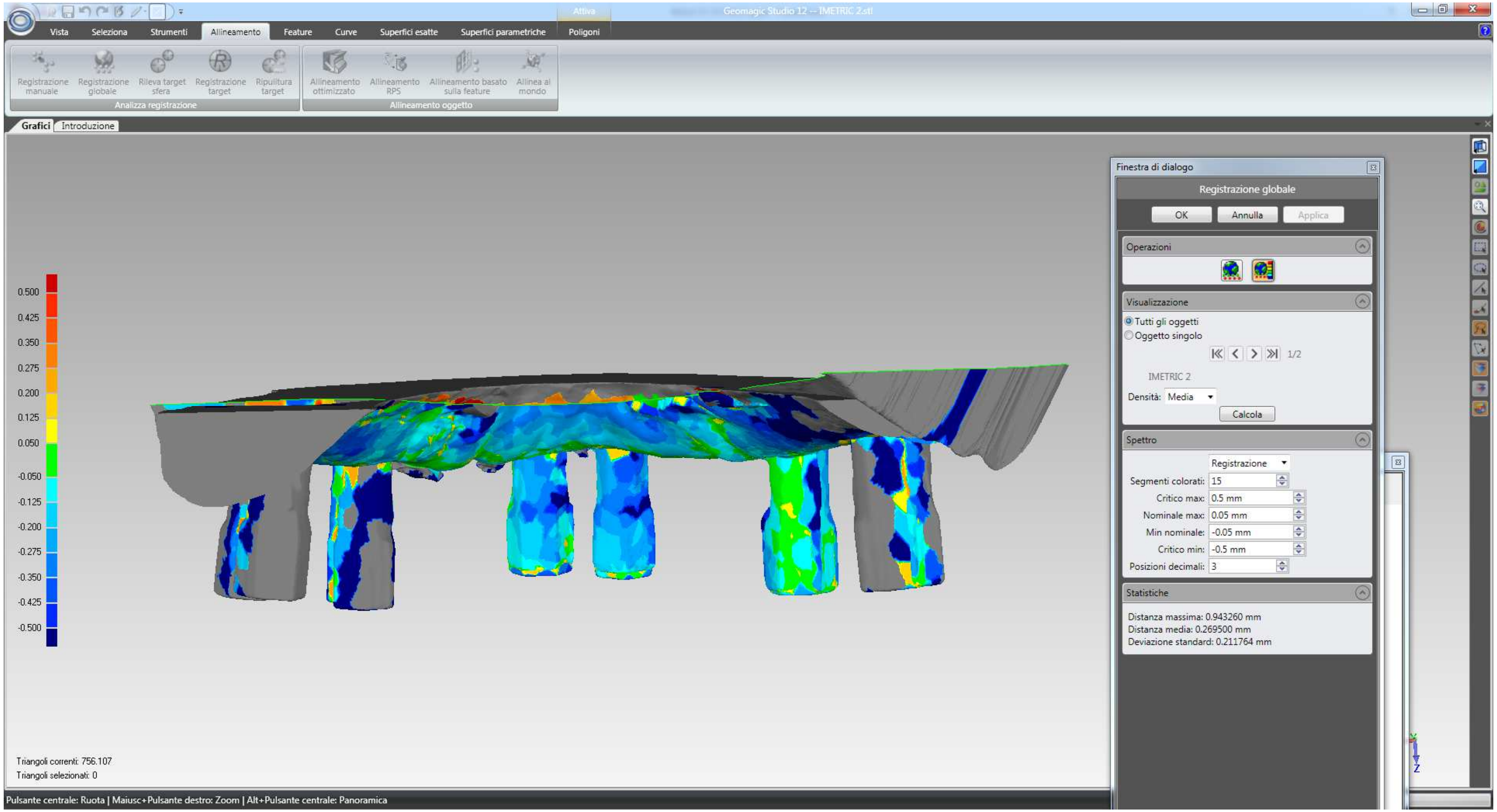

Plan 3 vs REF (IMETRIC)

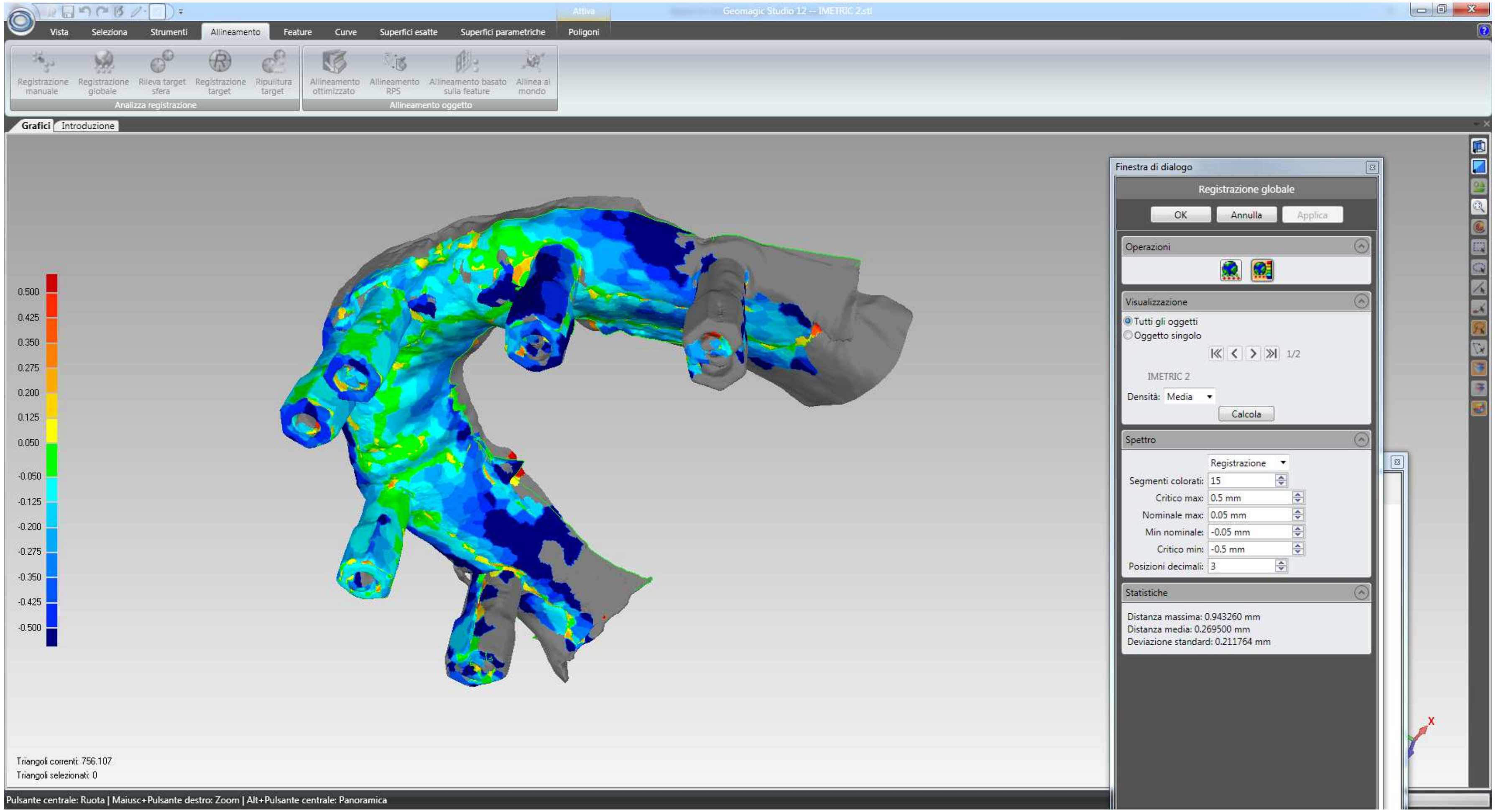

Plan 4 vs REF (IMETRIC)

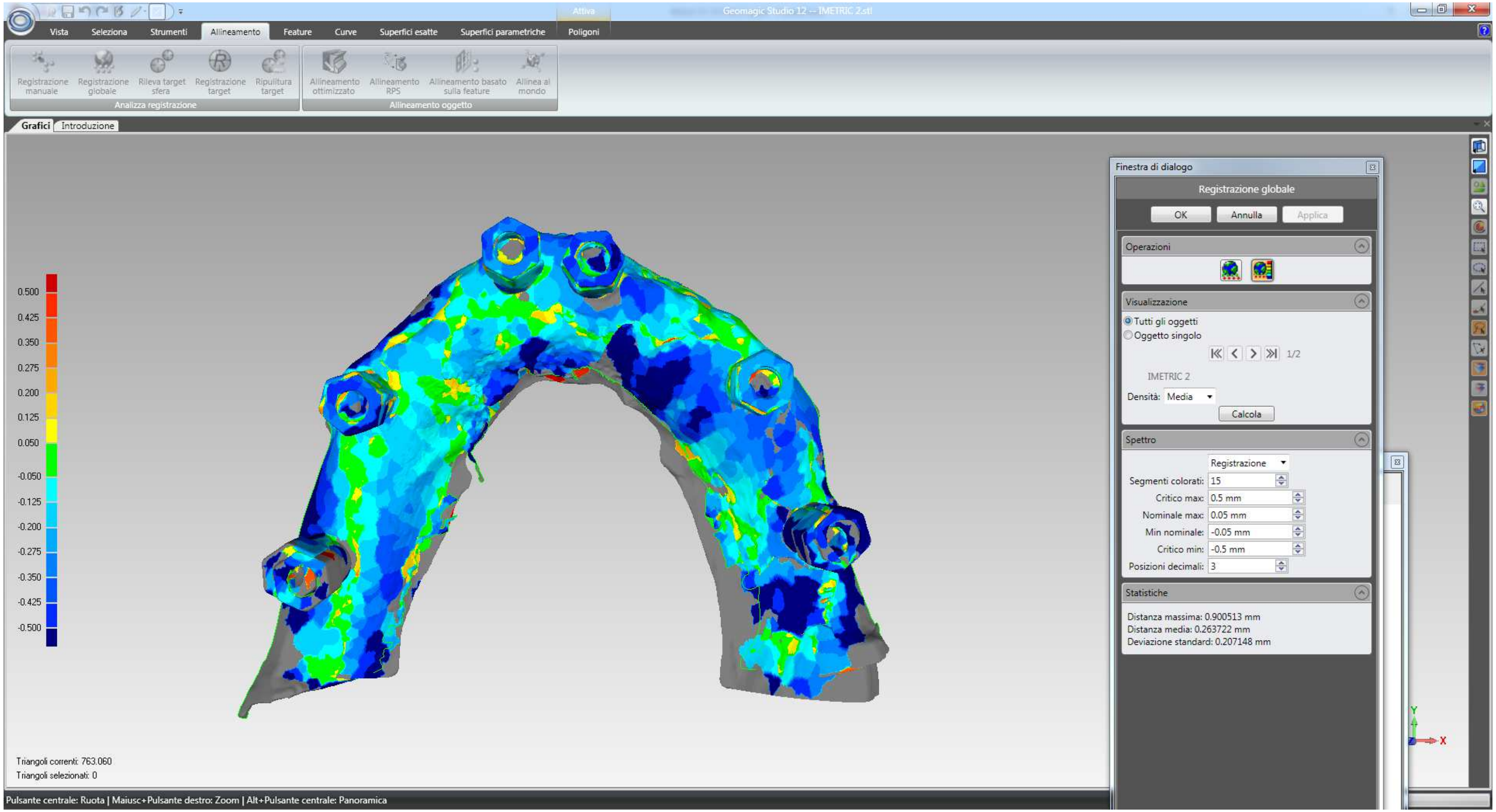

Plan 4 vs REF (IMETRIC)

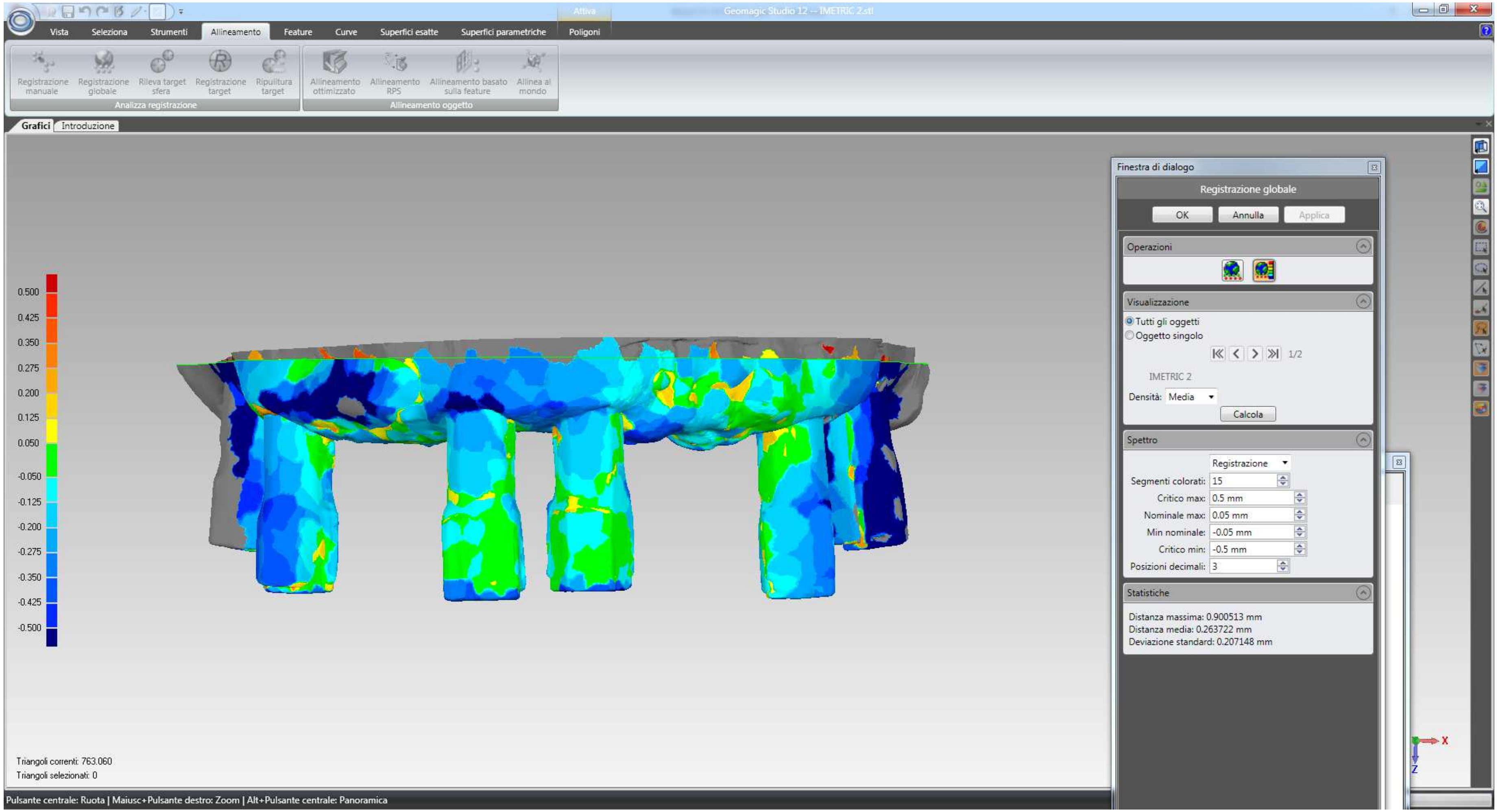

Plan 4 vs REF (IMETRIC)

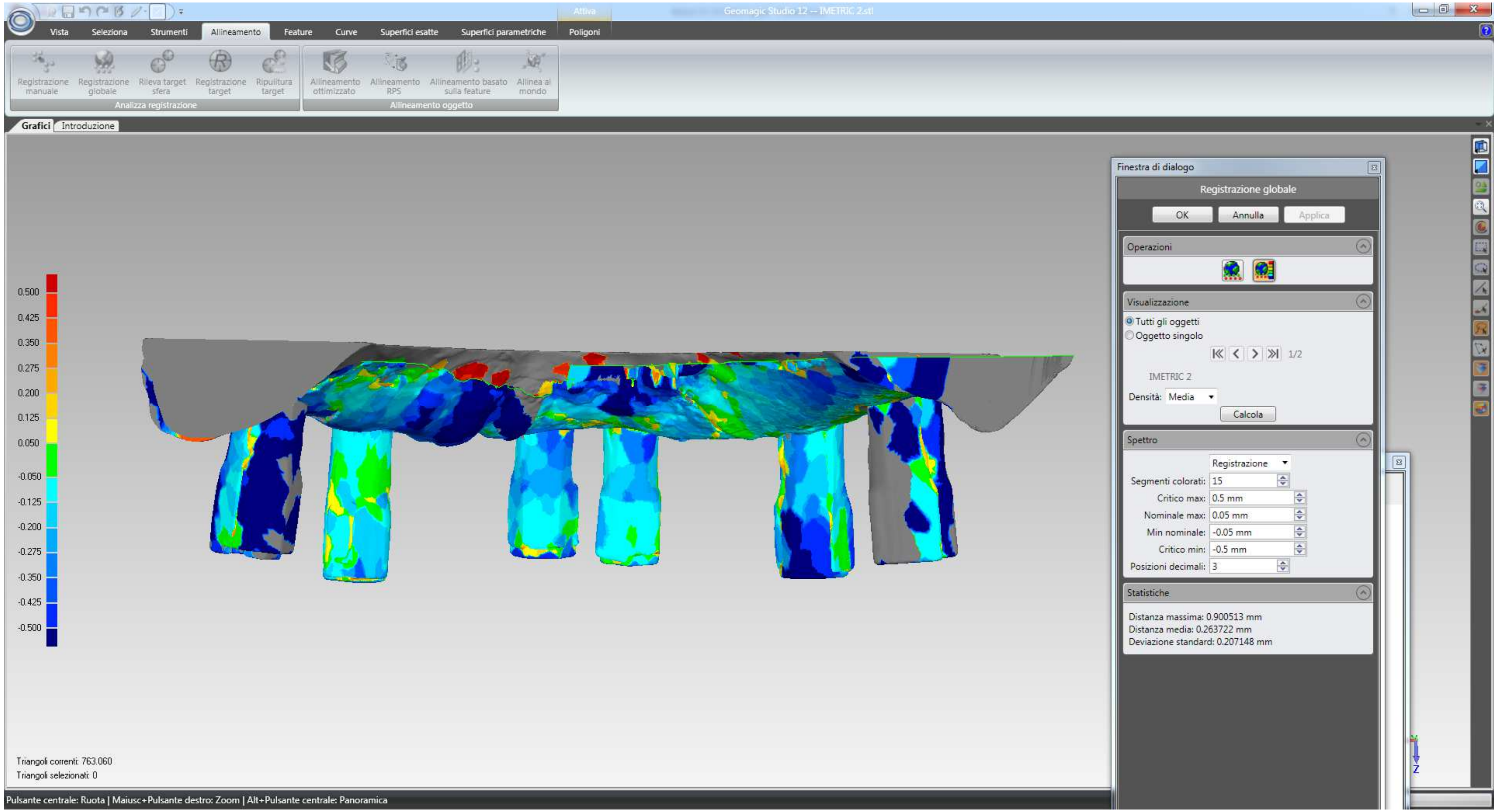

Plan 4 vs REF (IMETRIC)

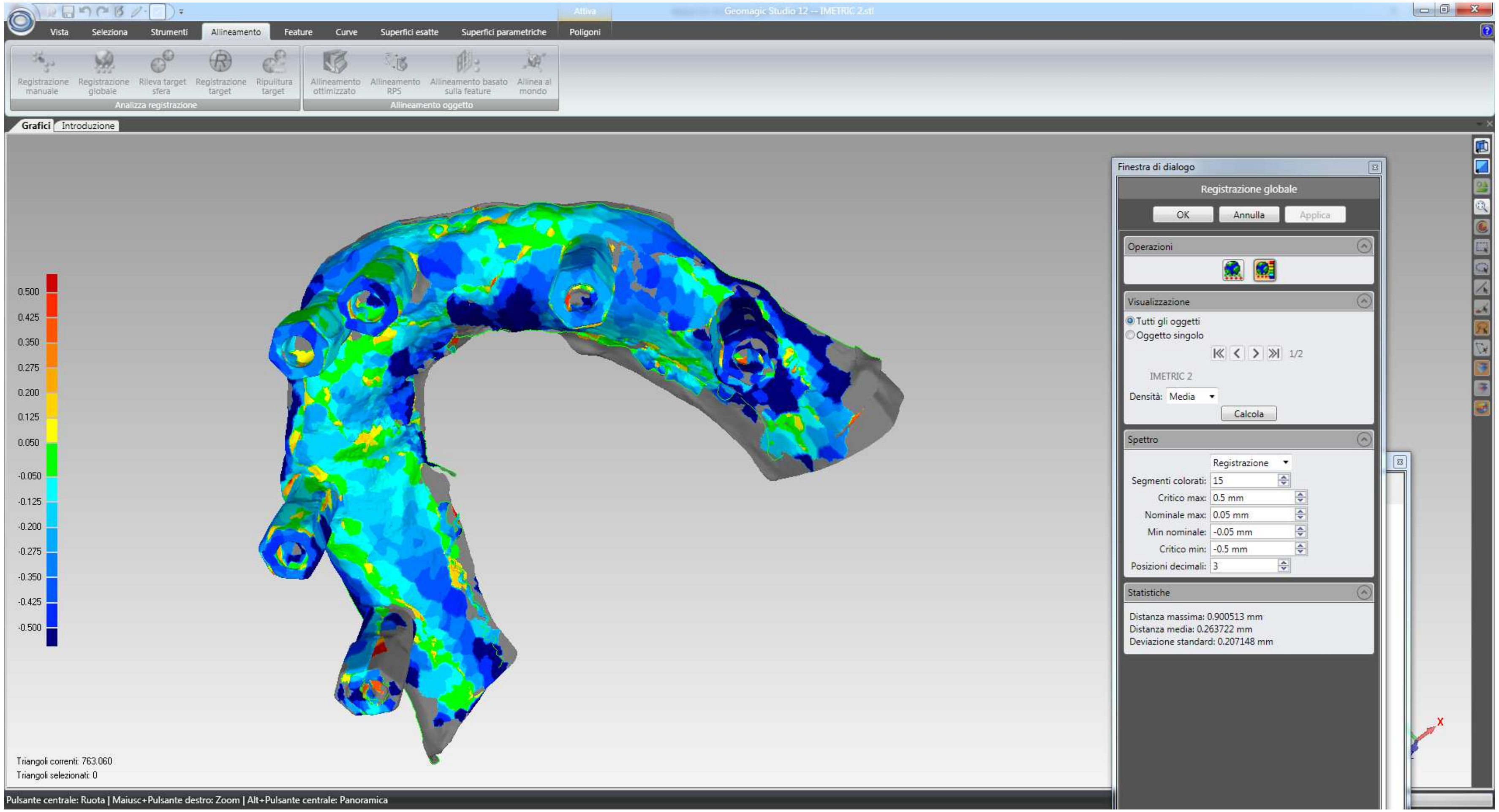

Plan 5 vs REF (IMETRIC)

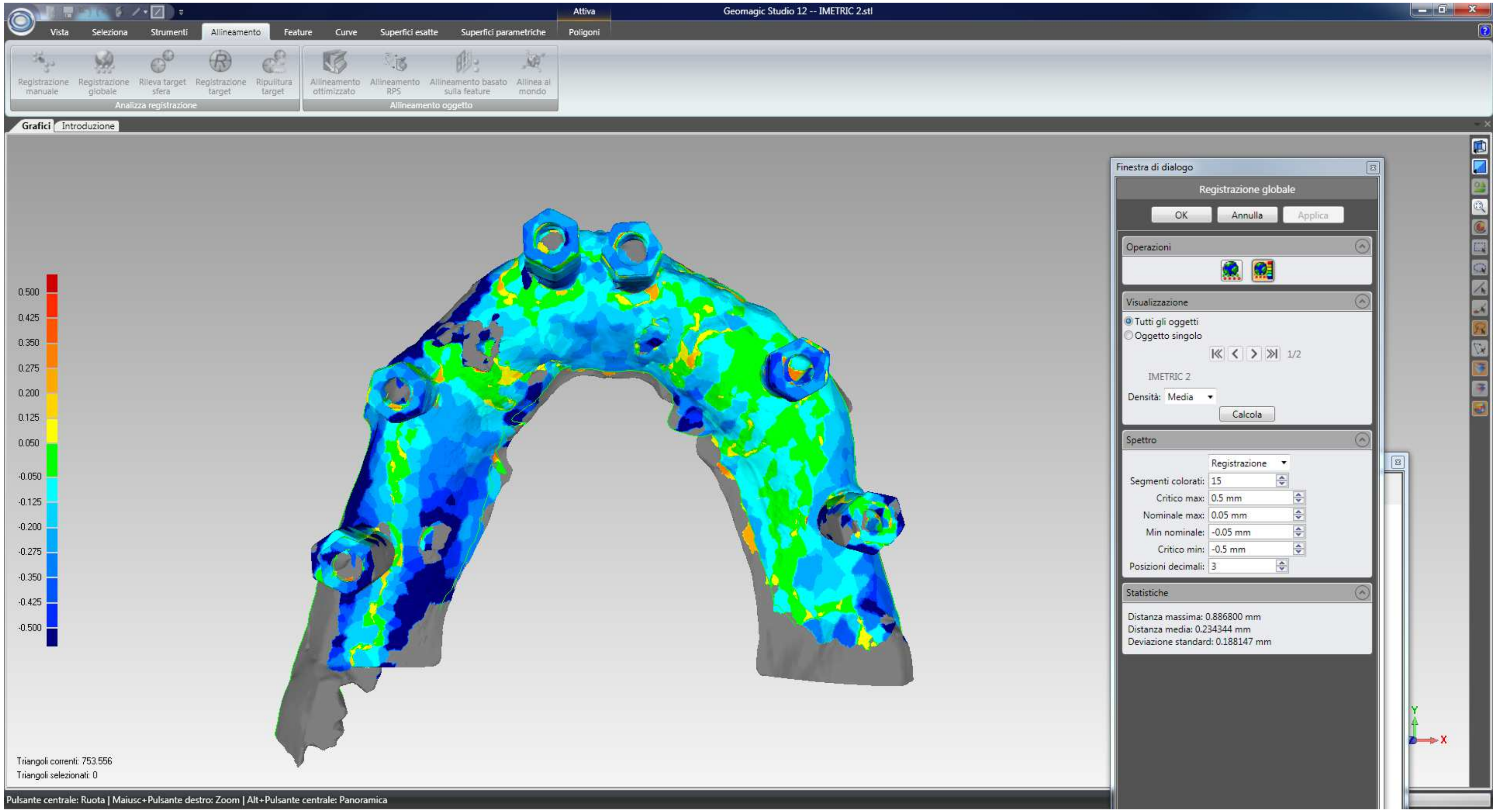

Plan 5 vs REF (IMETRIC)

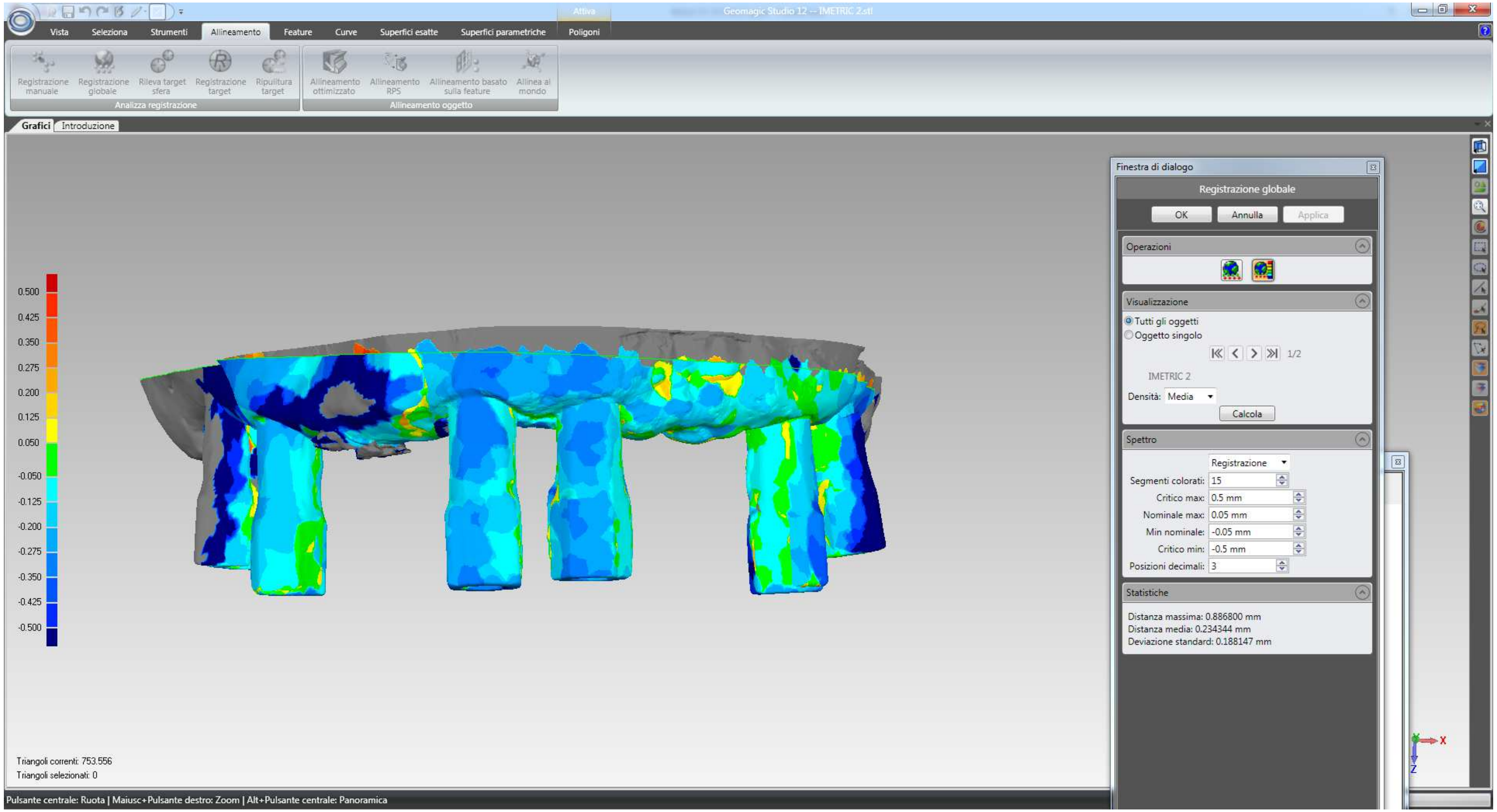

Plan 5 vs REF (IMETRIC)

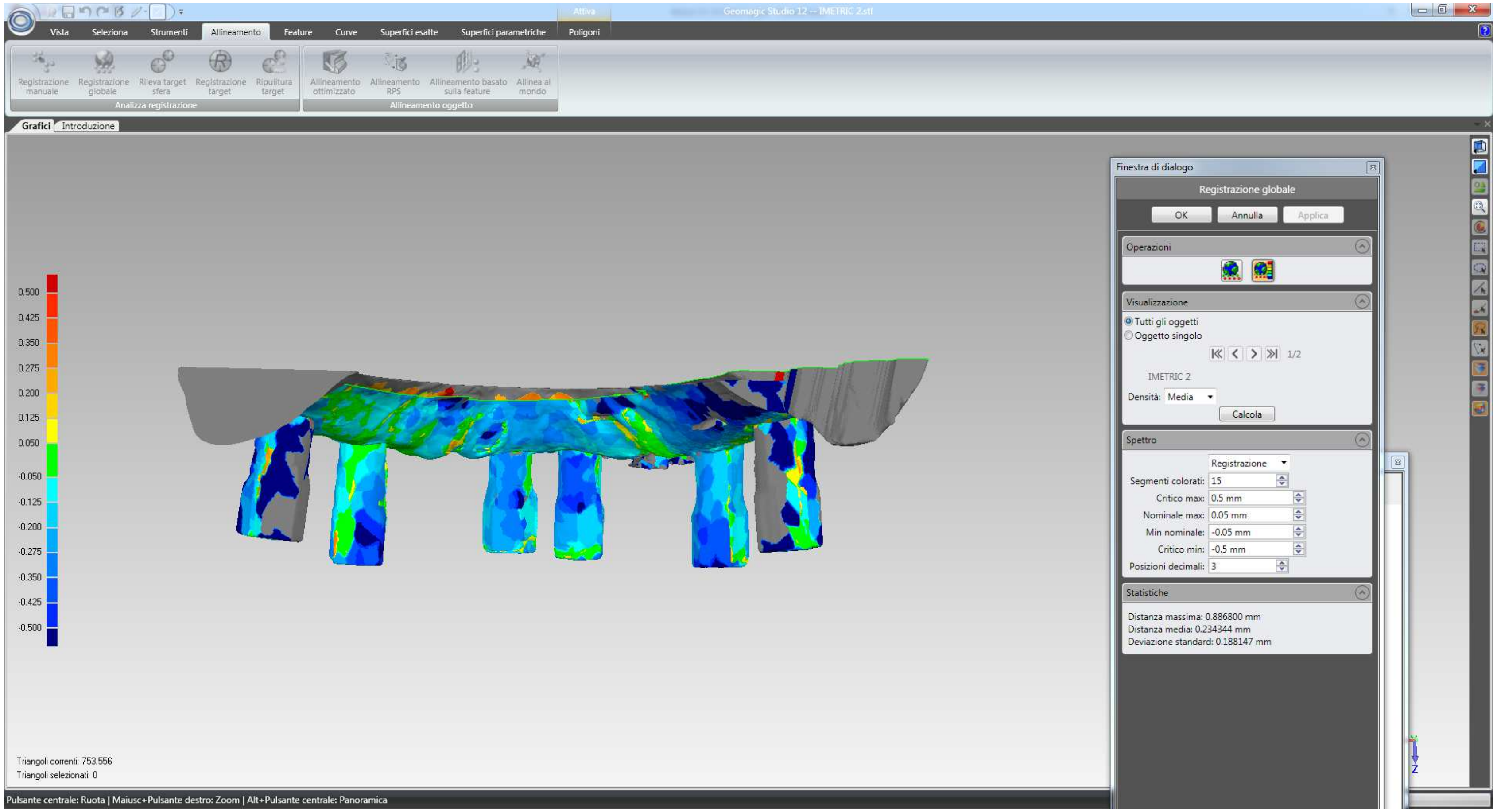

Plan 5 vs REF (IMETRIC)

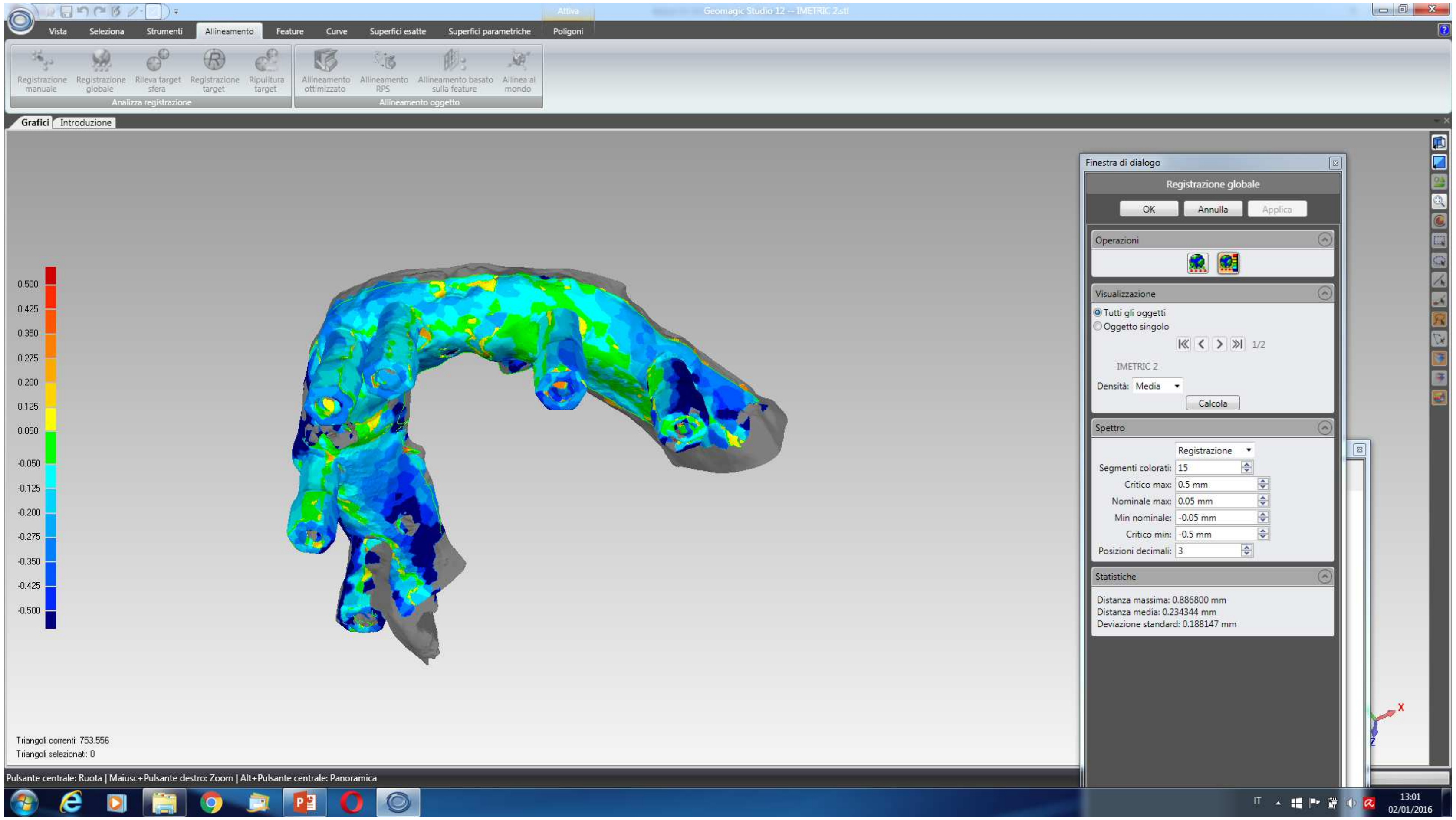

General trueness of the Planscan in the totally edentulous patient

	Mean distance	SD	Maximum distance
Plan 1	0.248	0.201	0.846
Plan 2	0.253	0.194	0.832
Plan 3	0.269	0.211	0.943
Plan 4	0.263	0.207	0.900
Plan 5	0.234	0.188	0.886

Overall Plane general accuracy: 0.253 (0.013)

GENERAL PRECISION EVALUATIONS FOR TRIOS, ZFX INTRASCAN, CARESTREAM, PLANSCAN (SCANS WERE RANDOM BUT HERE WE ORDERED THEM) IN THE TOTALLY EDENTULOUS MODEL

GENERAL SETTINGS FOR FINAL REGISTRATION (TRUENESS):

0.5 mm, 0.05 mm, -0.05 mm, -0.5 mm (see scale)

Trios 3 as reference (best accuracy) vs trios 1

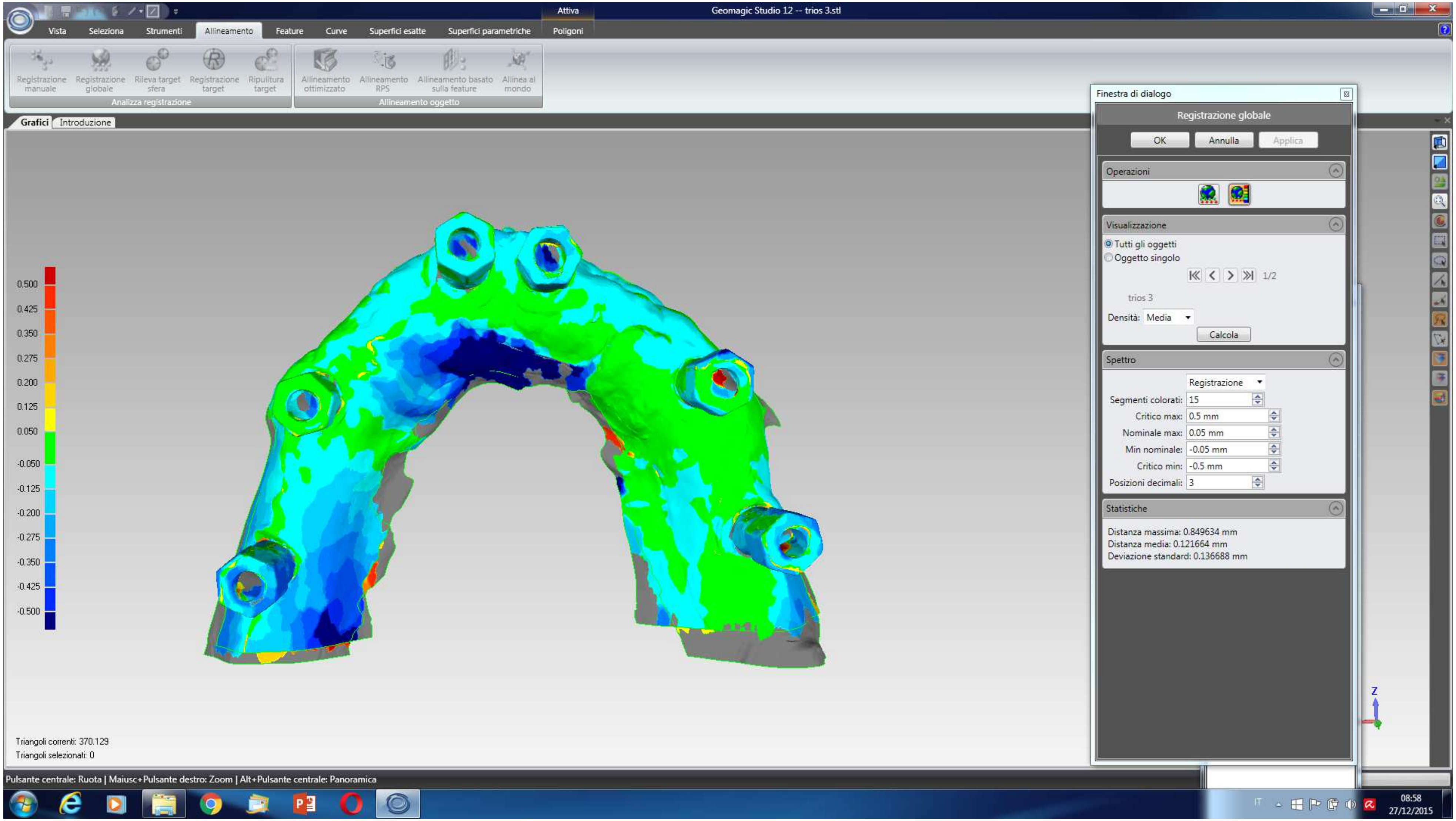

Trios 3 as reference (best accuracy) vs trios 1

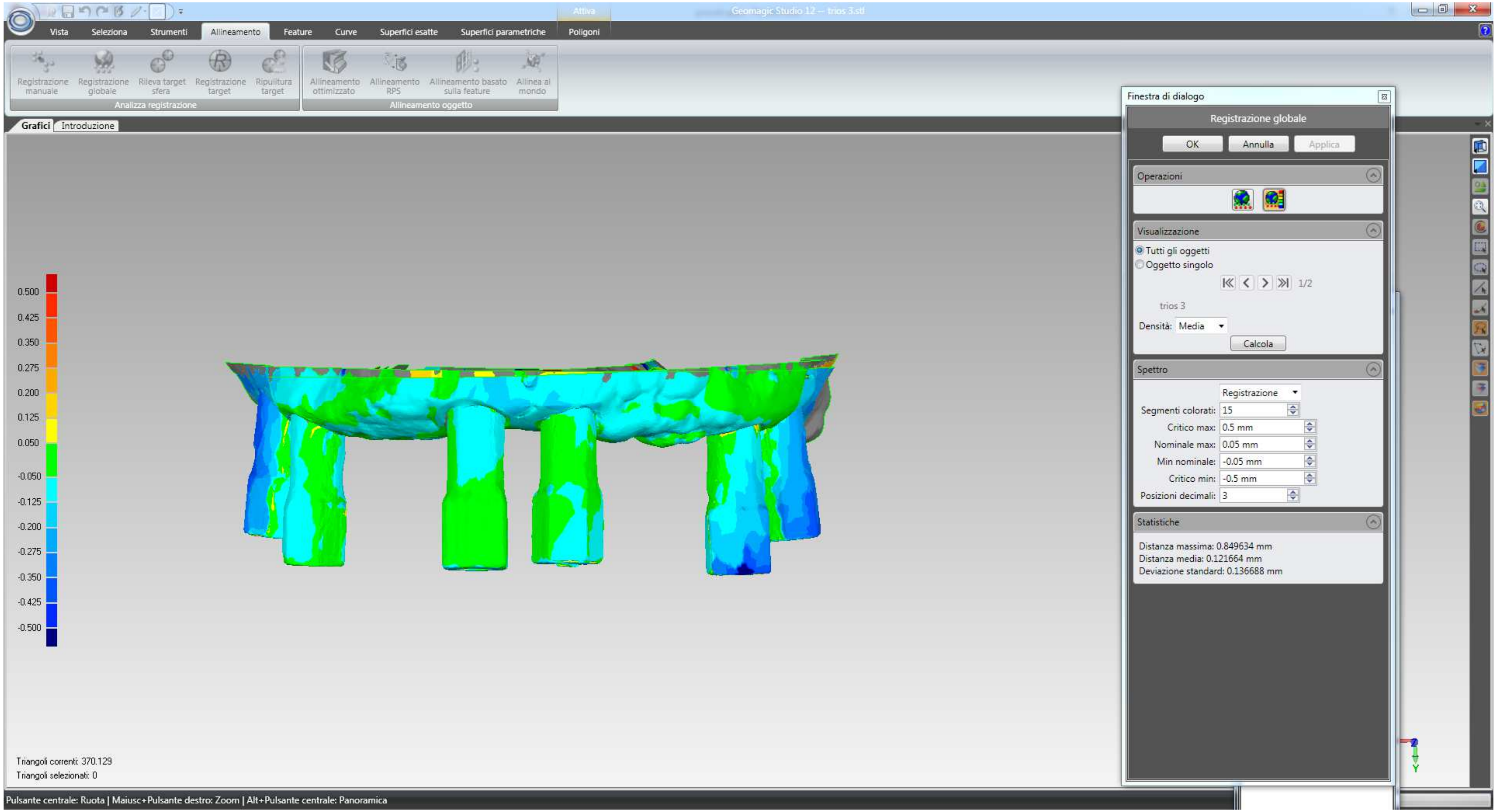

Trios 3 as reference (best accuracy) vs trios 1

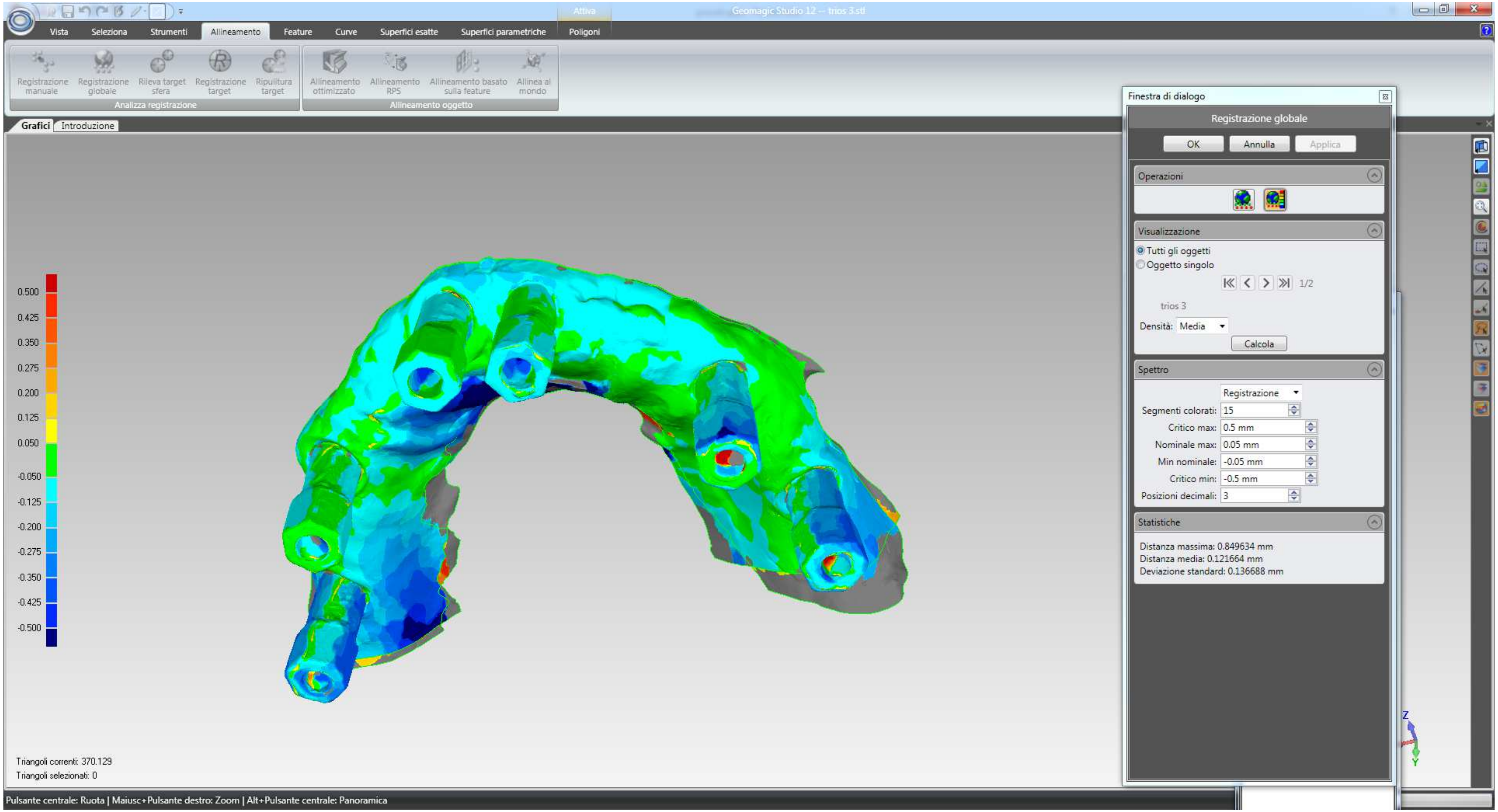

Trios 3 as reference (best accuracy) vs trios 1

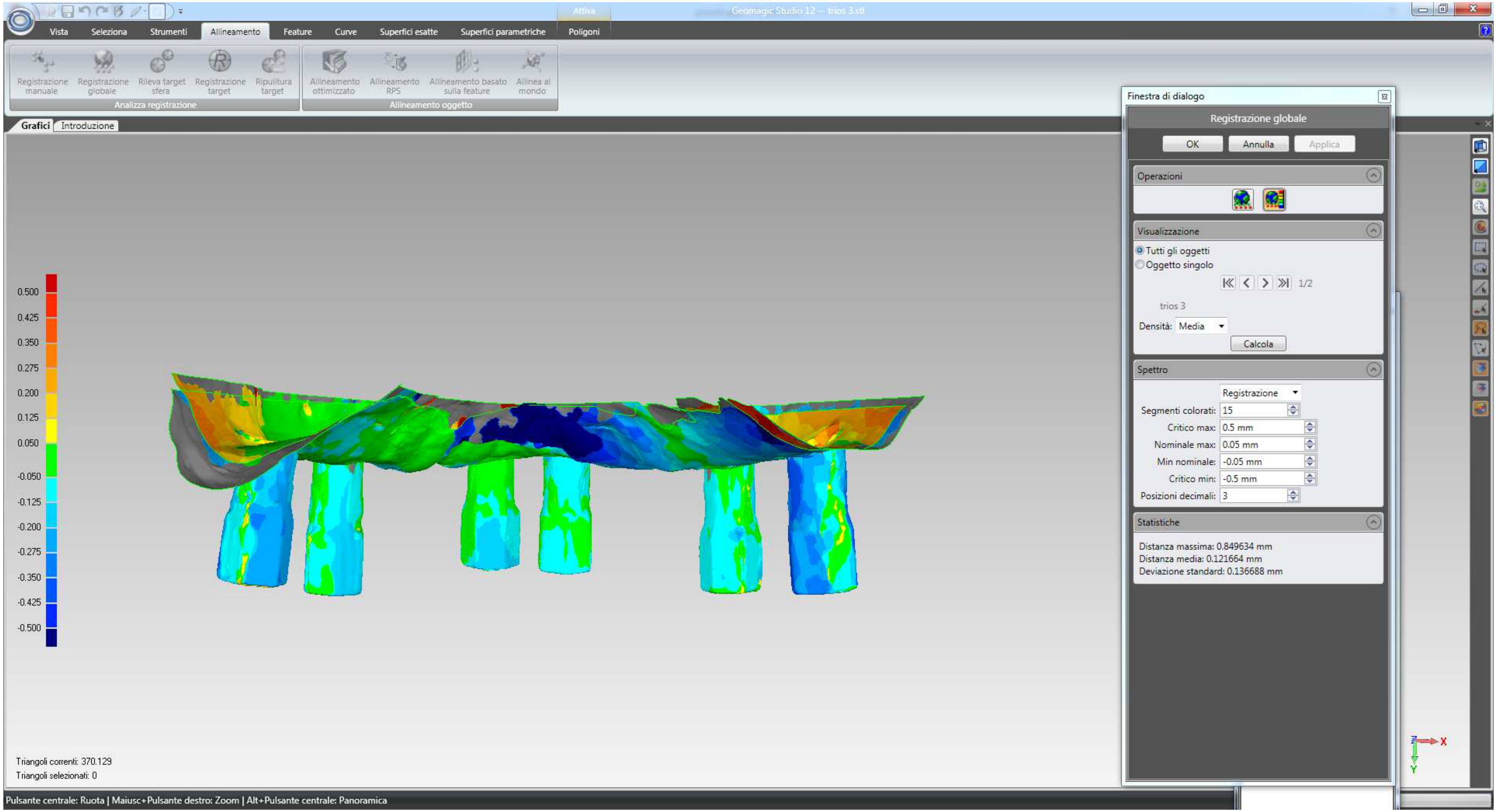

Trios 3 as reference (best accuracy) vs trios 2

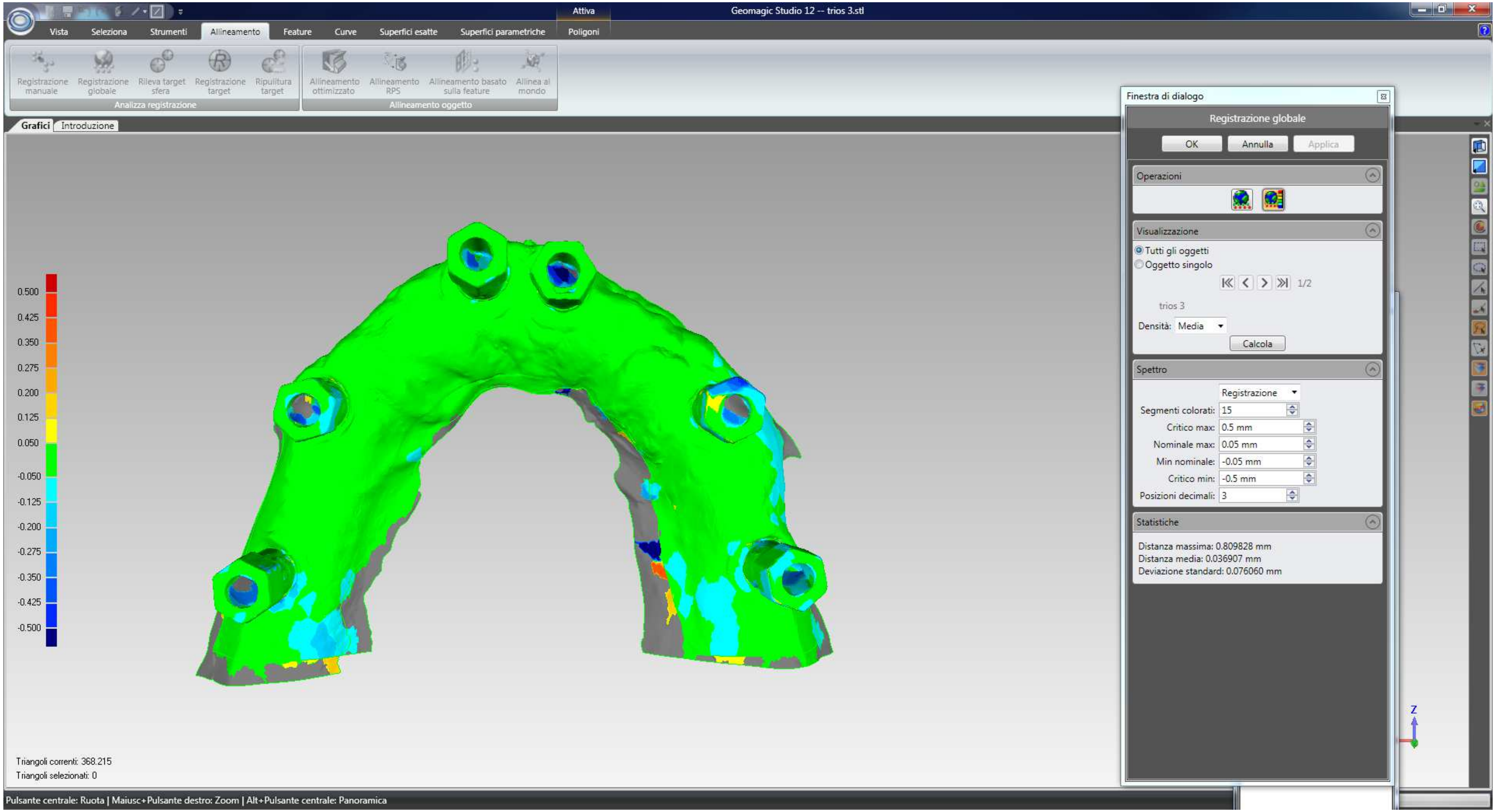

Trios 3 as reference (best accuracy) vs trios 2

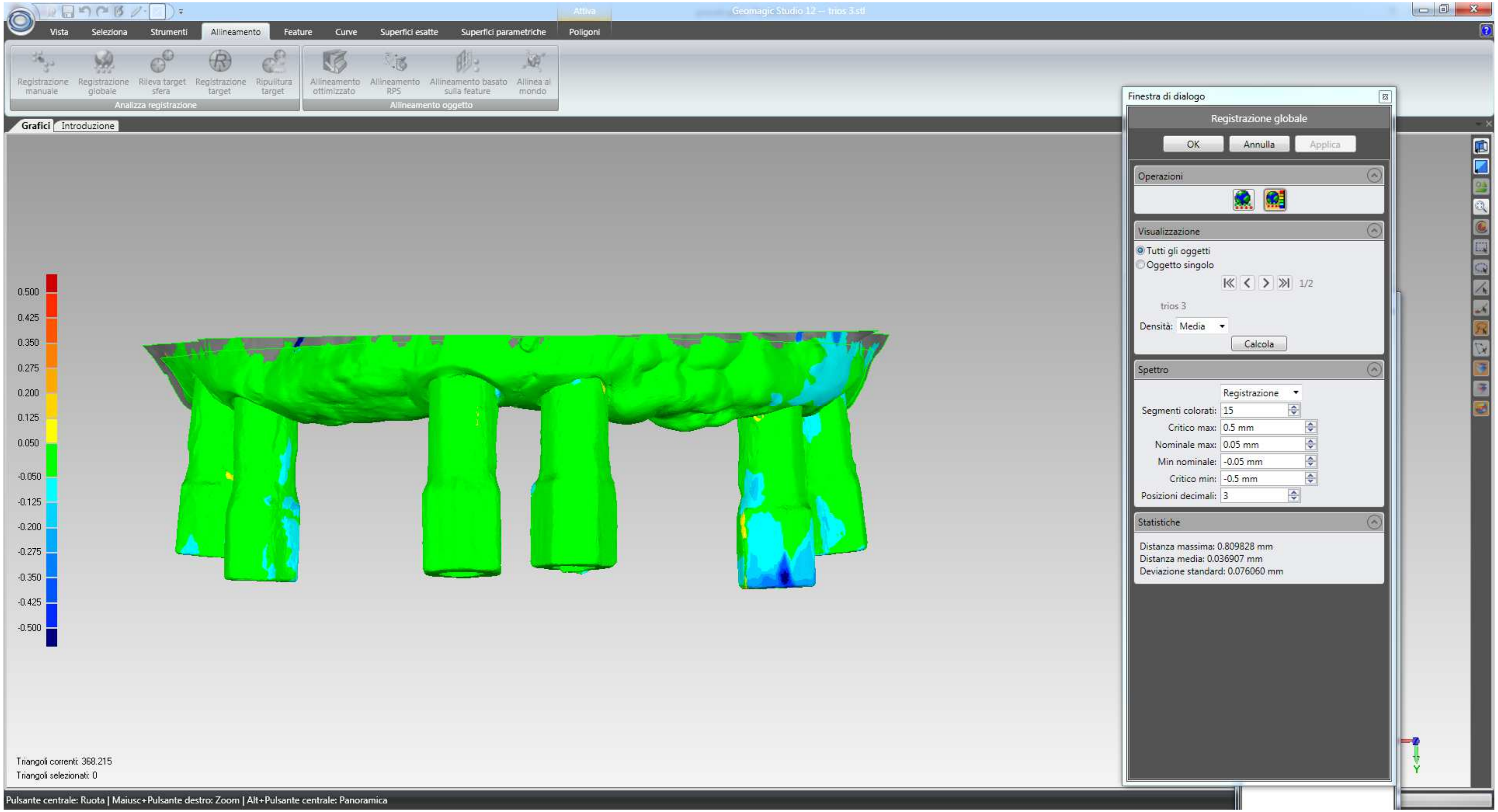

Trios 3 as reference (best accuracy) vs trios 2

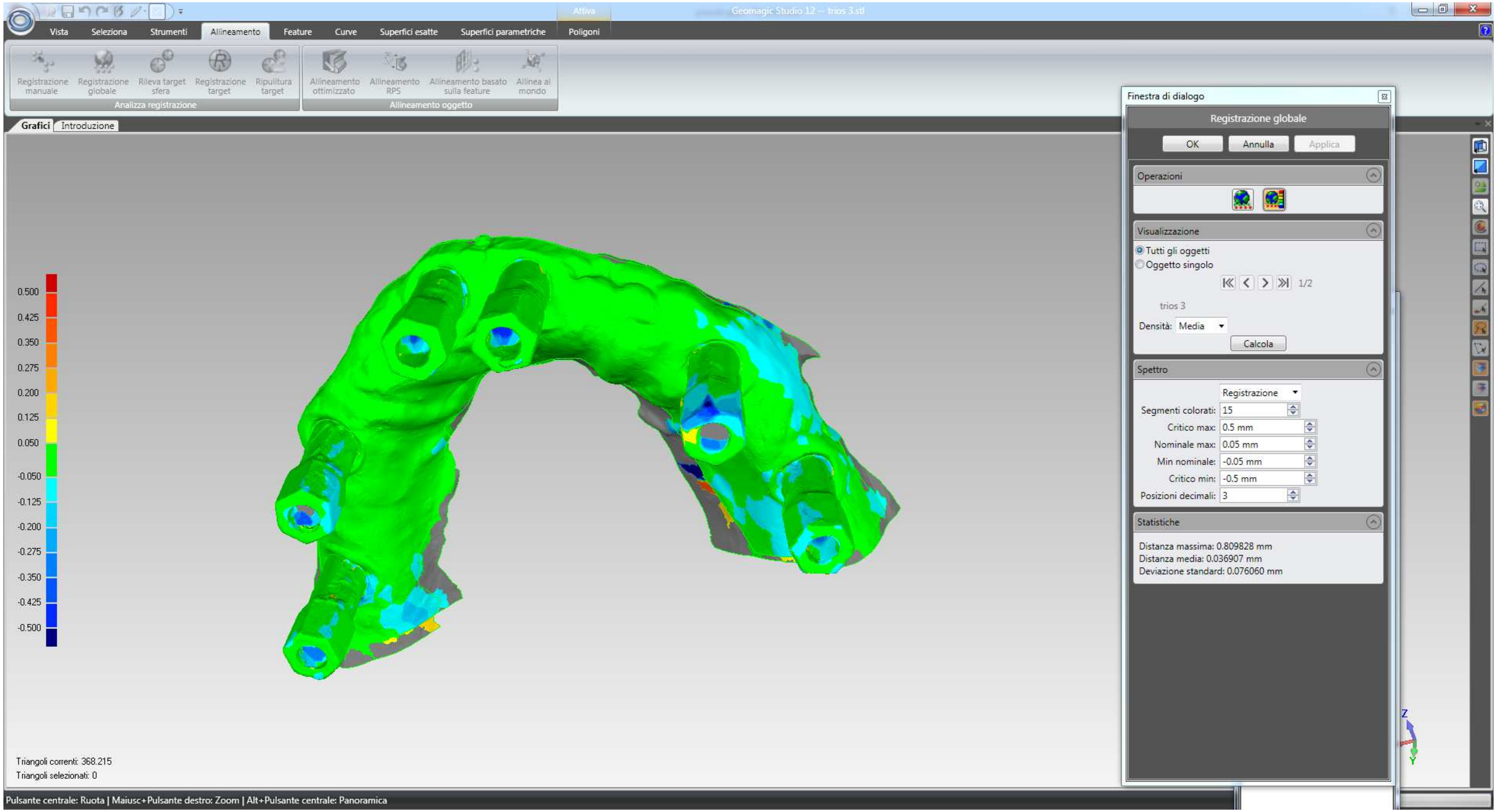

Trios 3 as reference (best accuracy) vs trios 2

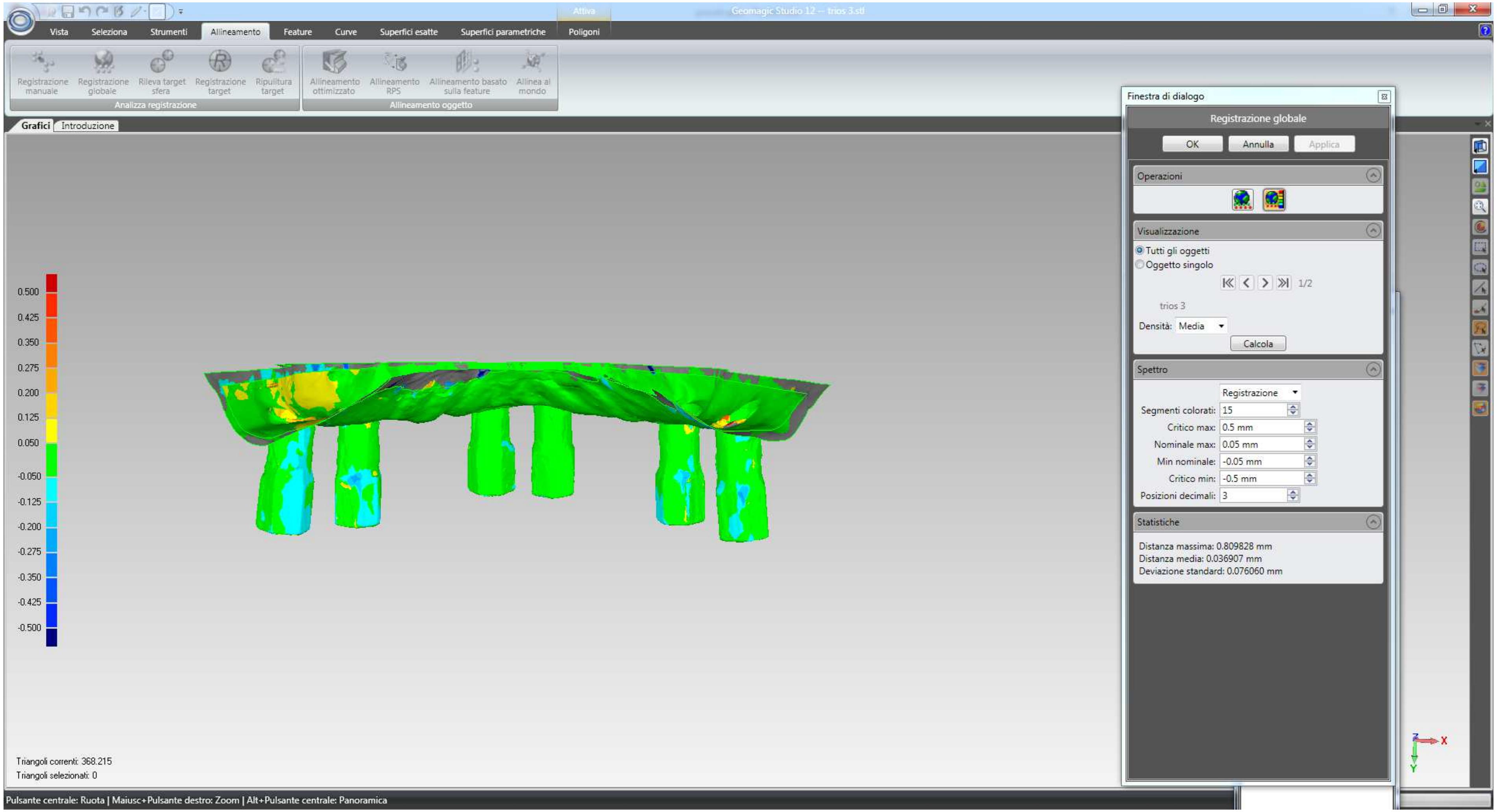

Trios 3 as reference (best accuracy) vs trios 4

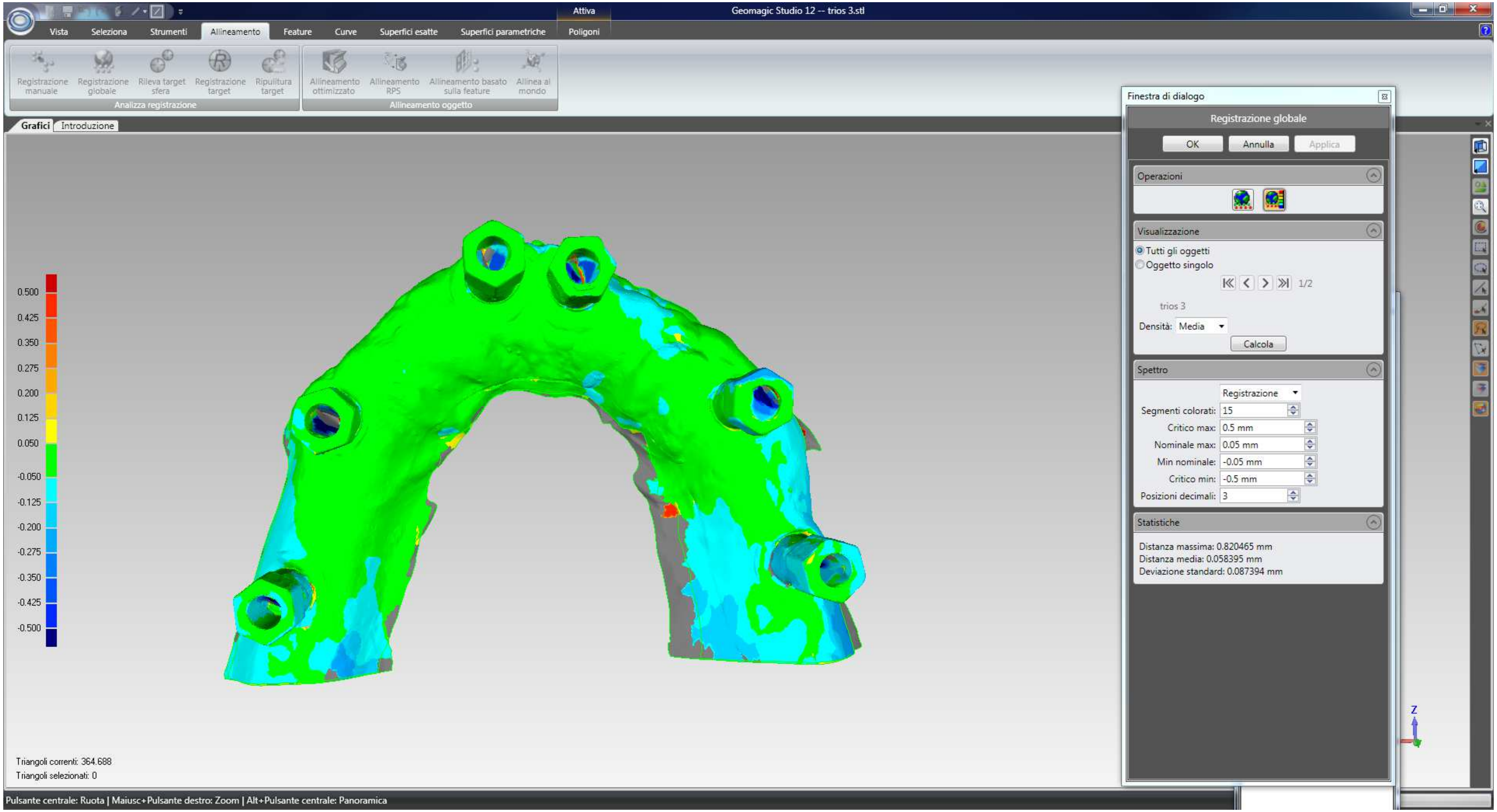

Trios 3 as reference (best accuracy) vs trios 4

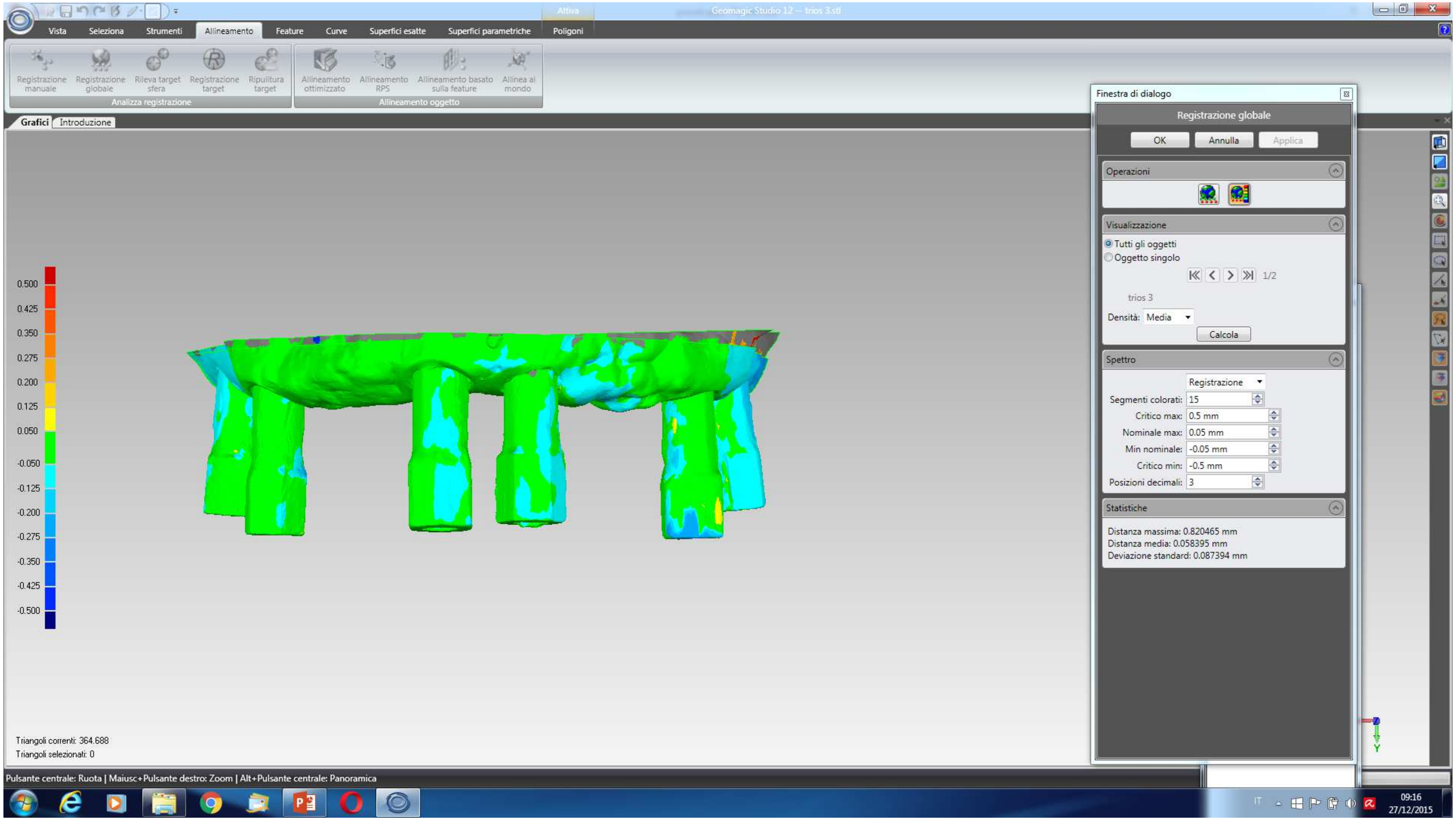

Trios 3 as reference (best accuracy) vs trios 4

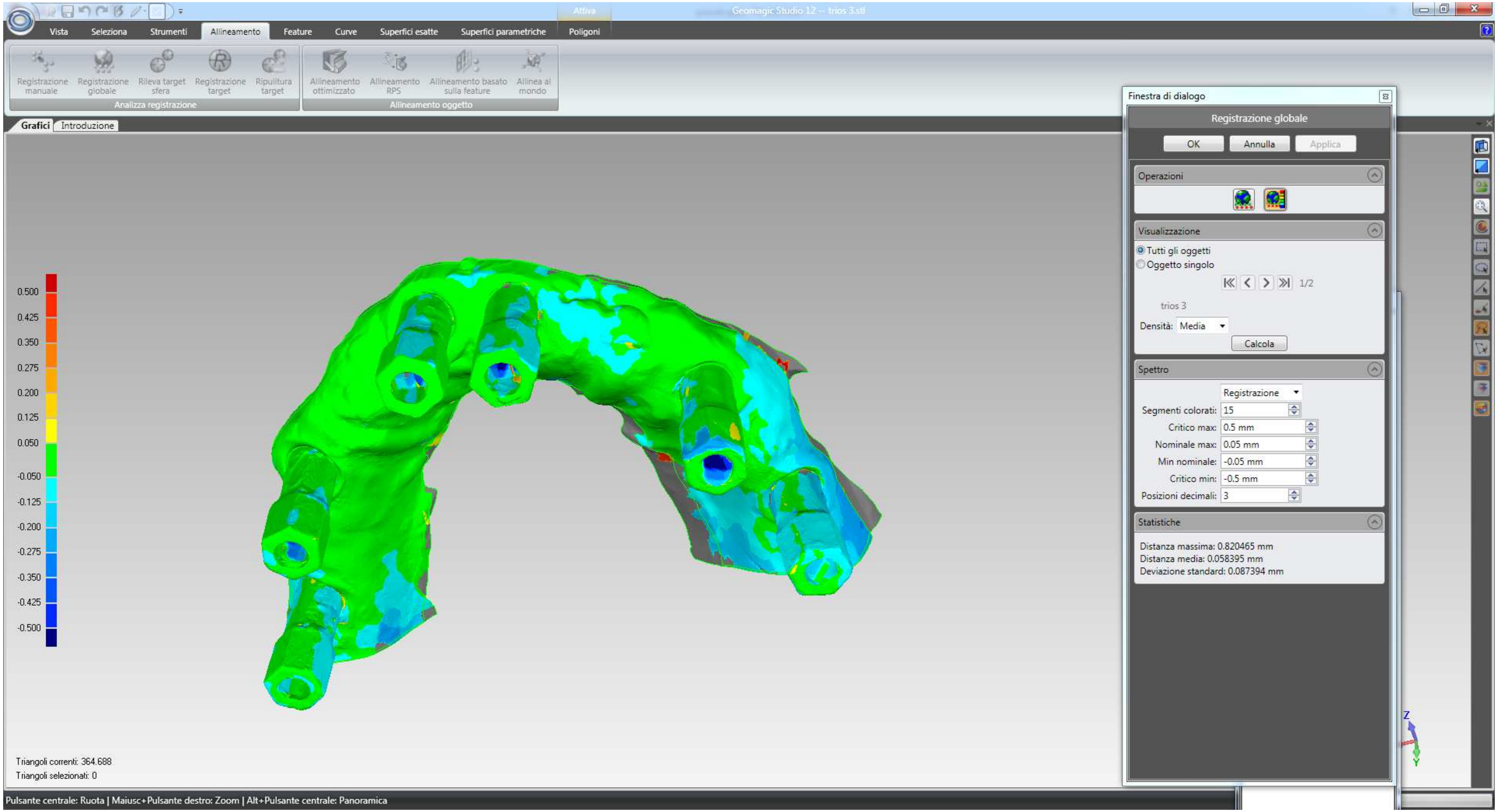

Trios 3 as reference (best accuracy) vs trios 4

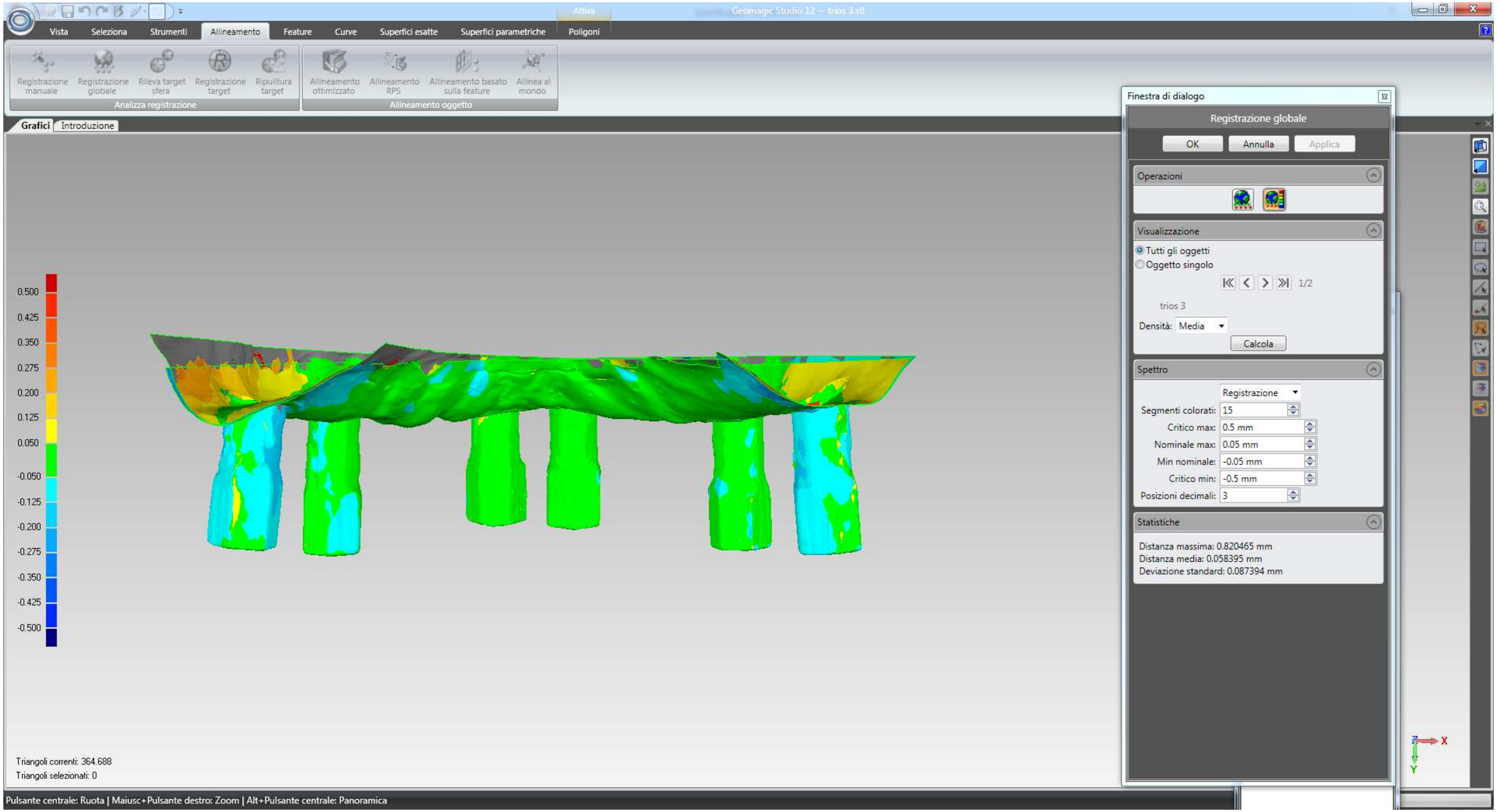

Trios 3 as reference (best accuracy) vs trios 5

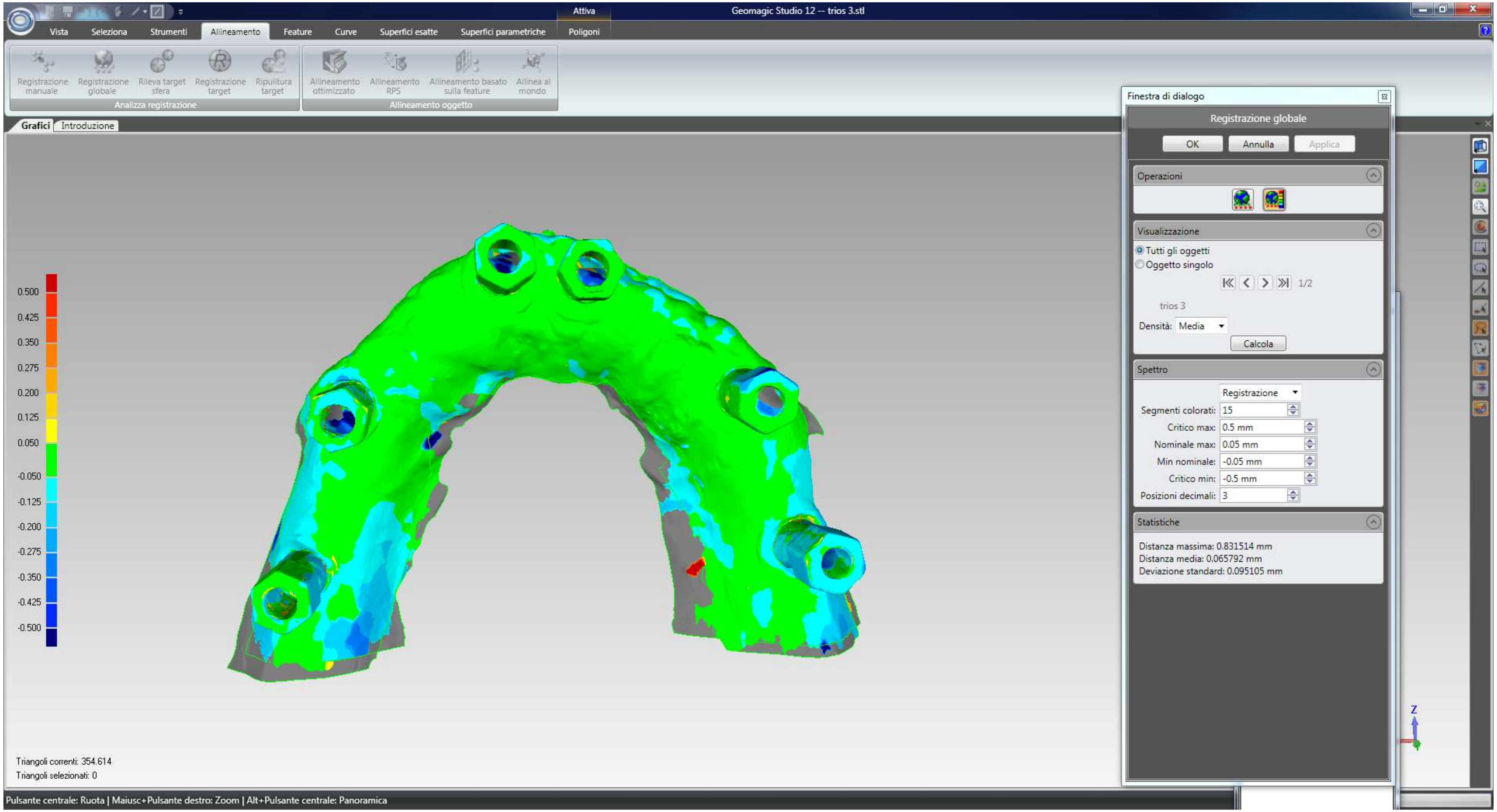

Trios 3 as reference (best accuracy) vs trios 5

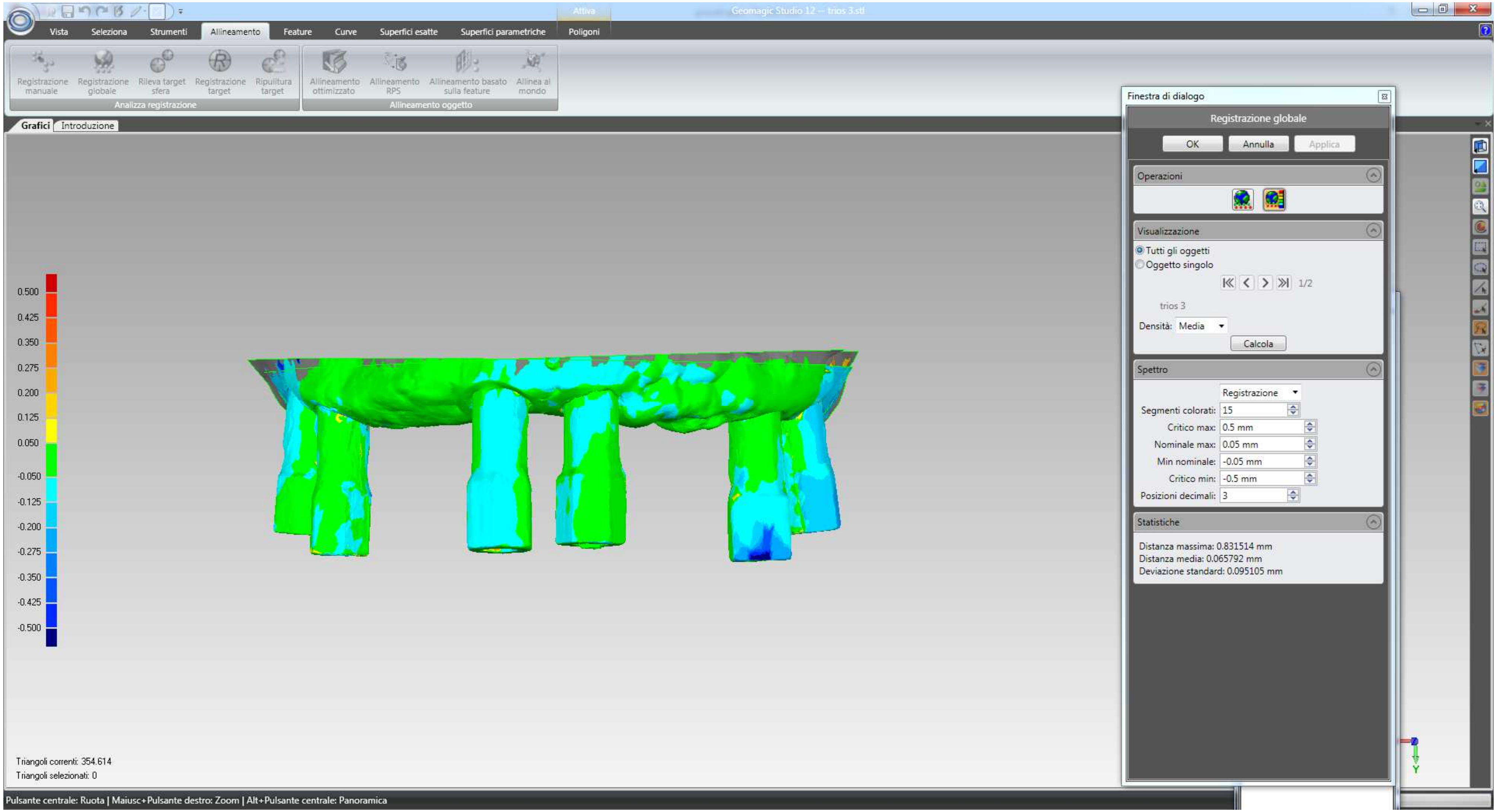

Trios 3 as reference (best accuracy) vs trios 5

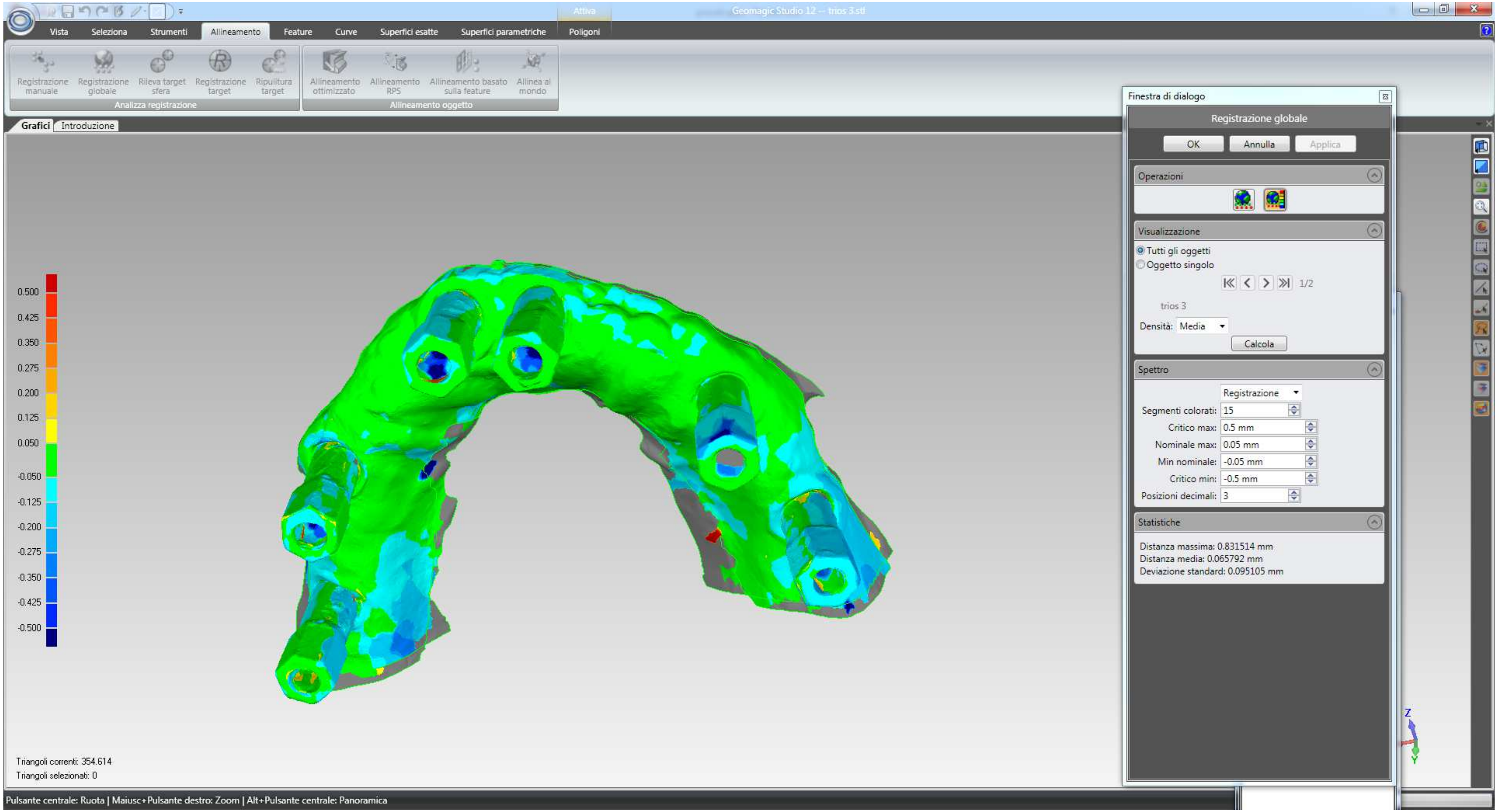

Trios 3 as reference (best accuracy) vs trios 5

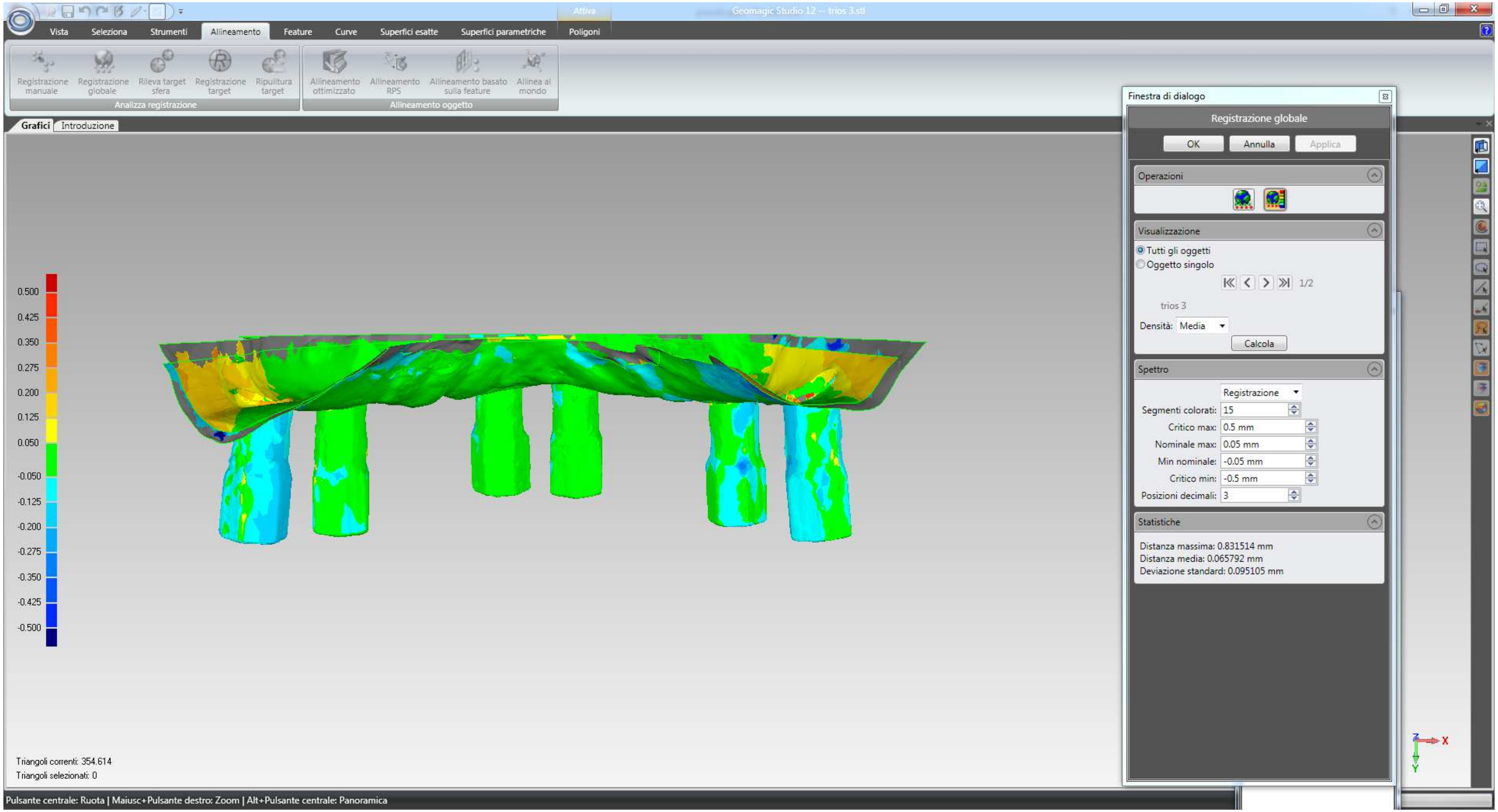

Trios 2 vs trios 4

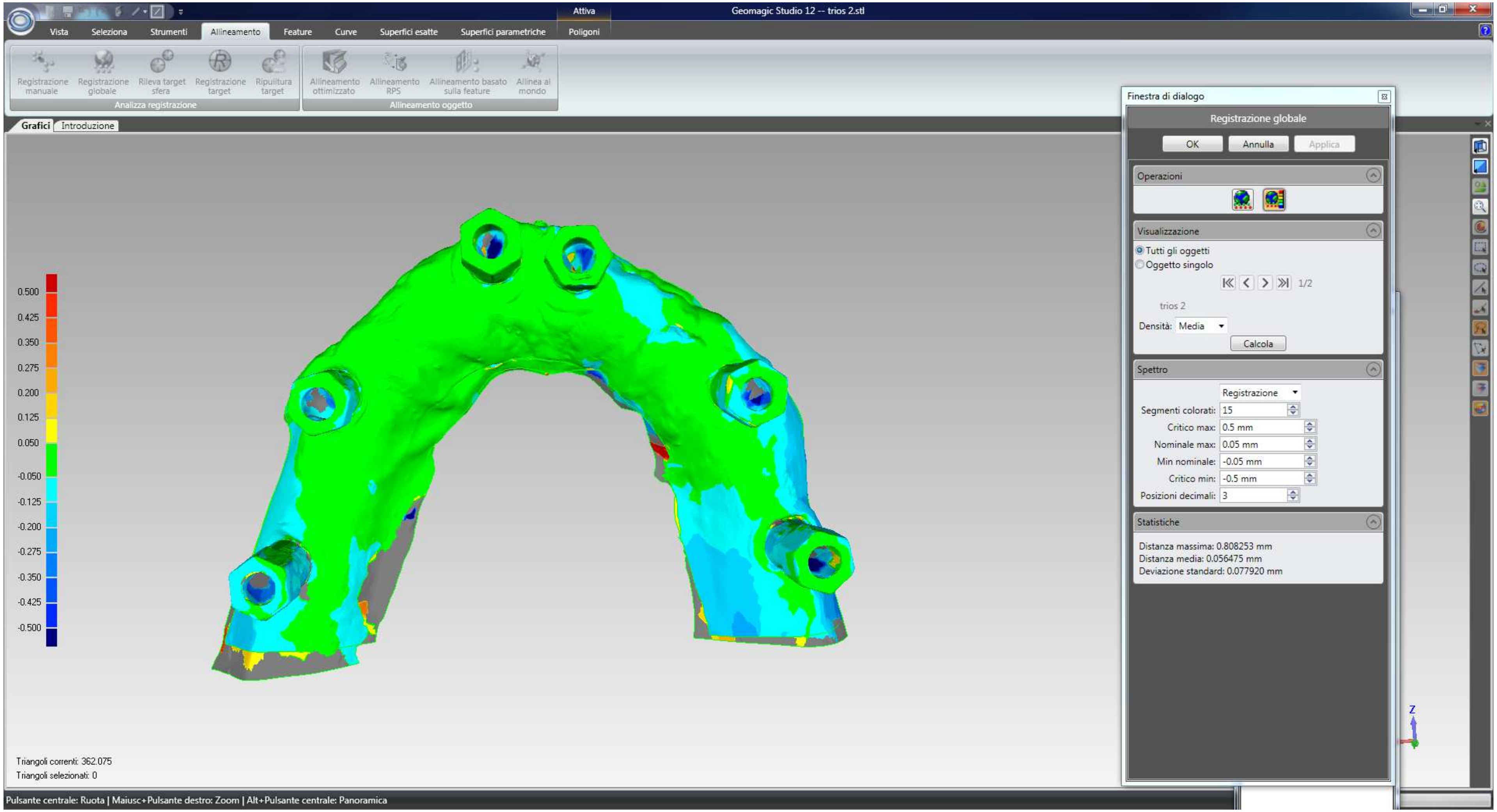

Trios 2 vs trios 4

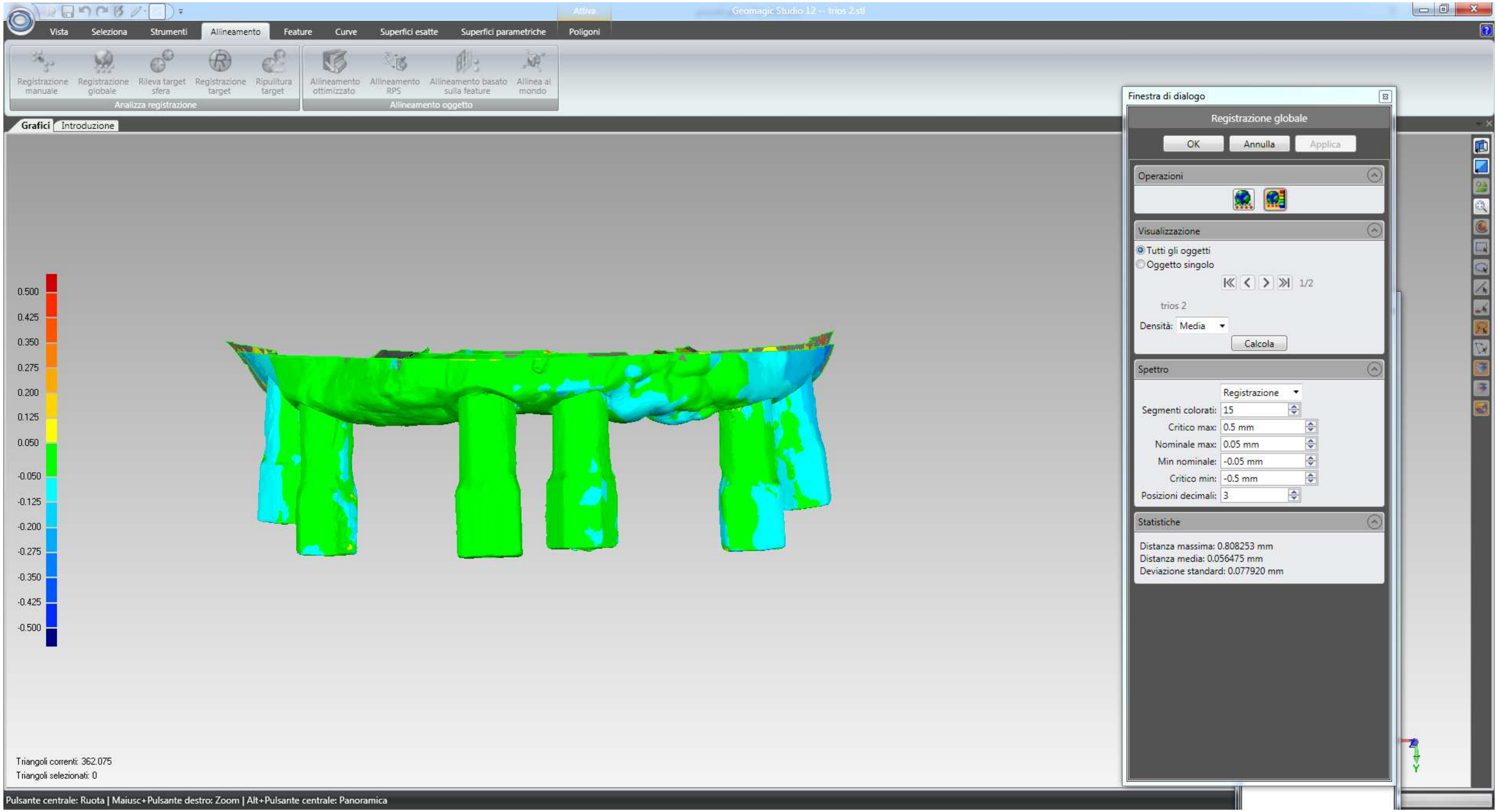

Trios 2 vs trios 4

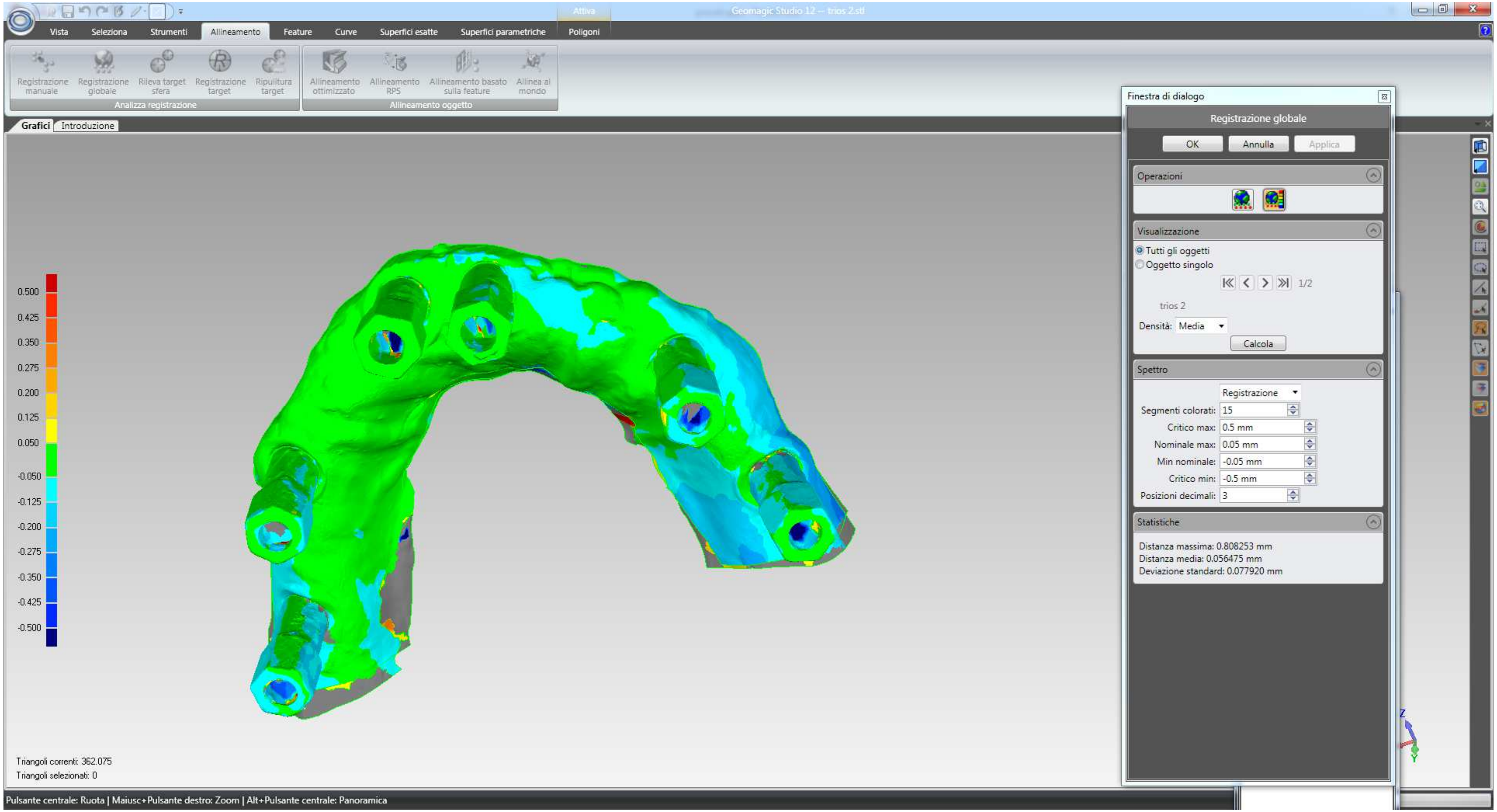

Trios 2 vs trios 4

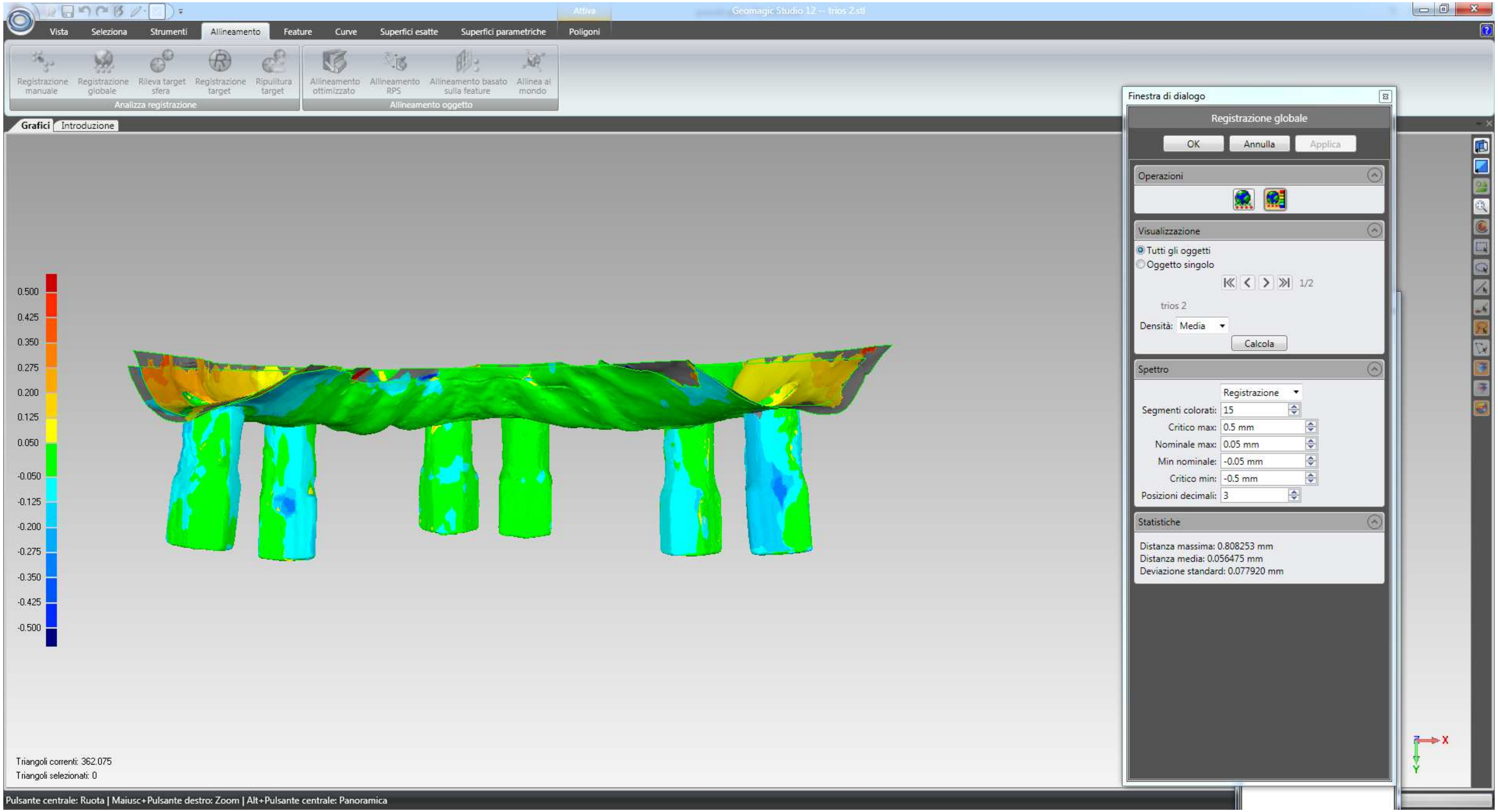

TRIOS overall general precision in the fully edentulous model

	Mean distance	SD	Maximum distance
Trios 3 vs 1	0.121	0.136	0.849
Trios 3 vs 2	0.035	0.076	0.809
Trios 3 vs 4	0.058	0.087	0.820
Trios 3 vs 5	0.065	0.095	0.831
Trios 2 vs 4	0.056	0.077	0.808

Overall Trios general precision: 0.067 (0.032)

Zfx 1 as reference (best accuracy) vs Zfx 2

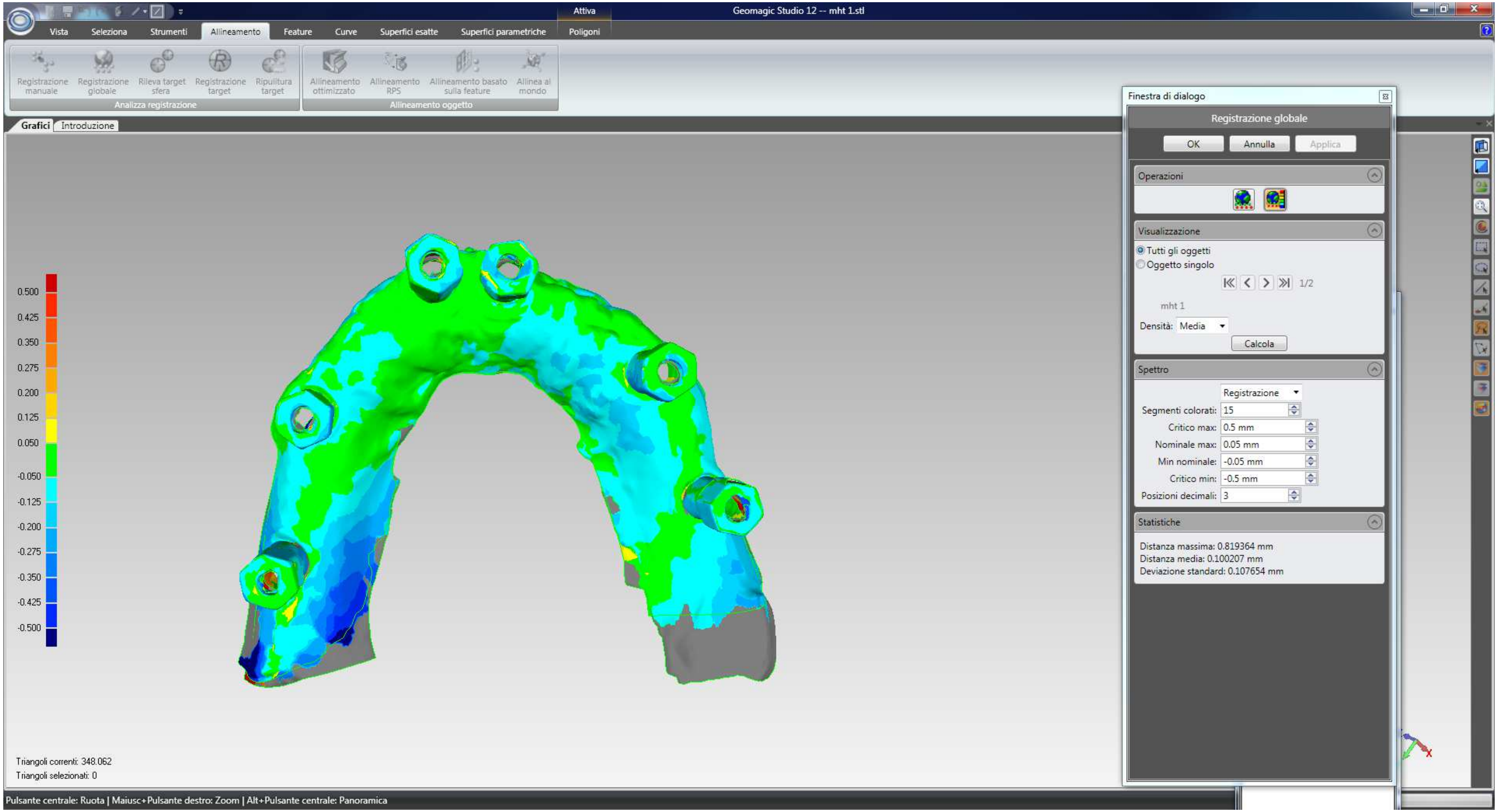

Zfx 1 as reference (best accuracy) vs Zfx 2

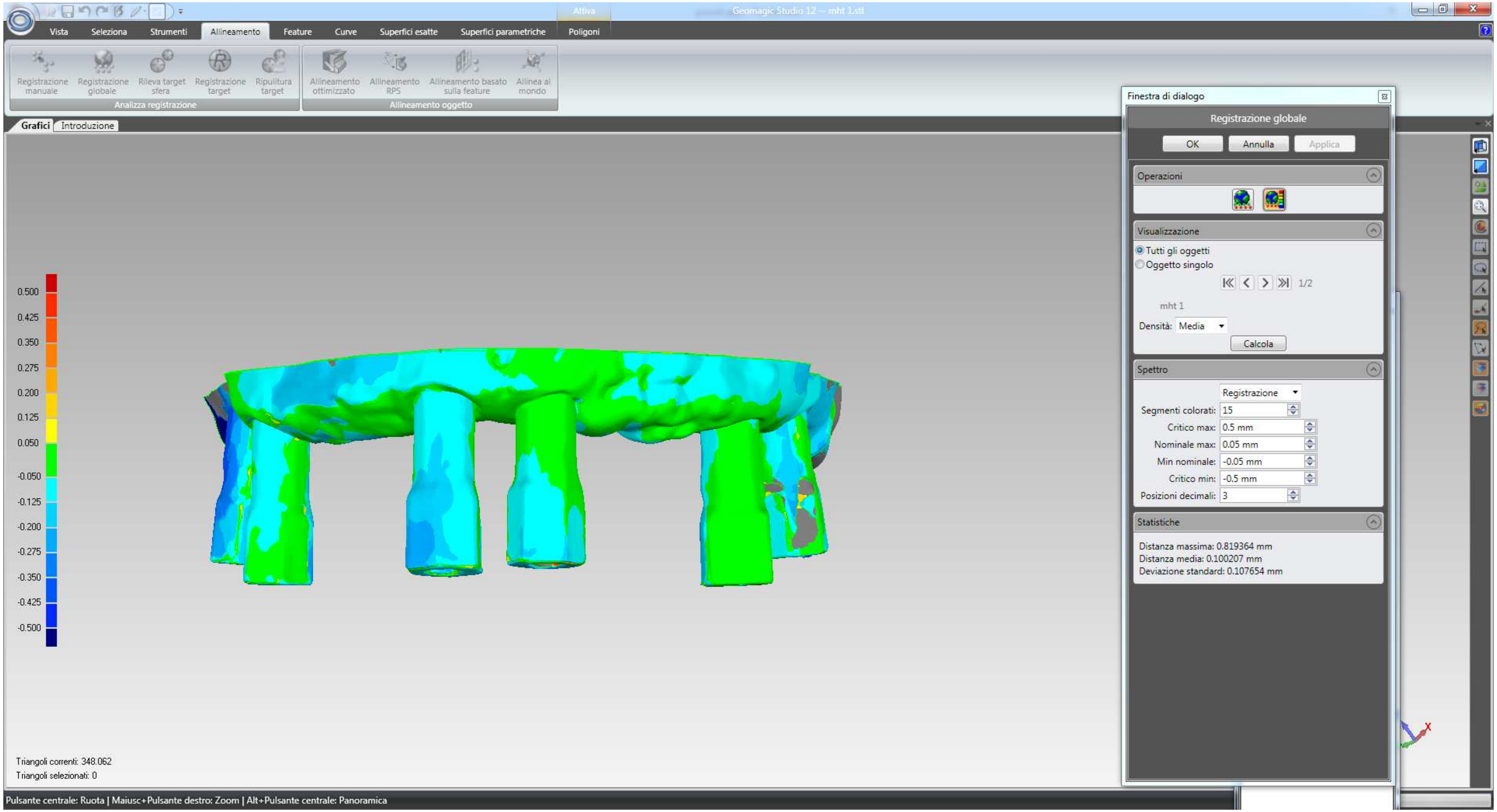

Zfx 1 as reference (best accuracy) vs Zfx 2

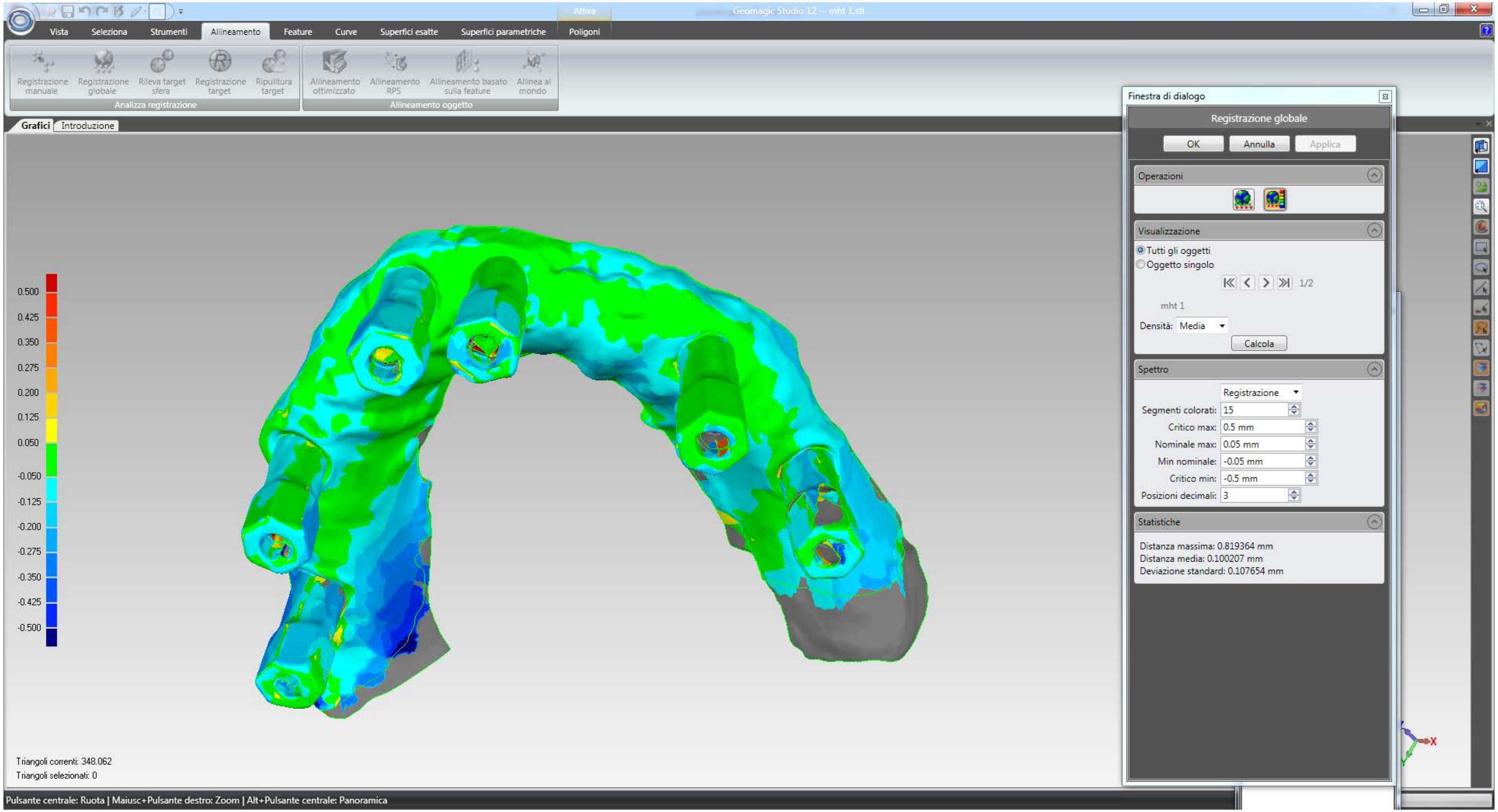

Zfx 1 as reference (best accuracy) vs Zfx 2

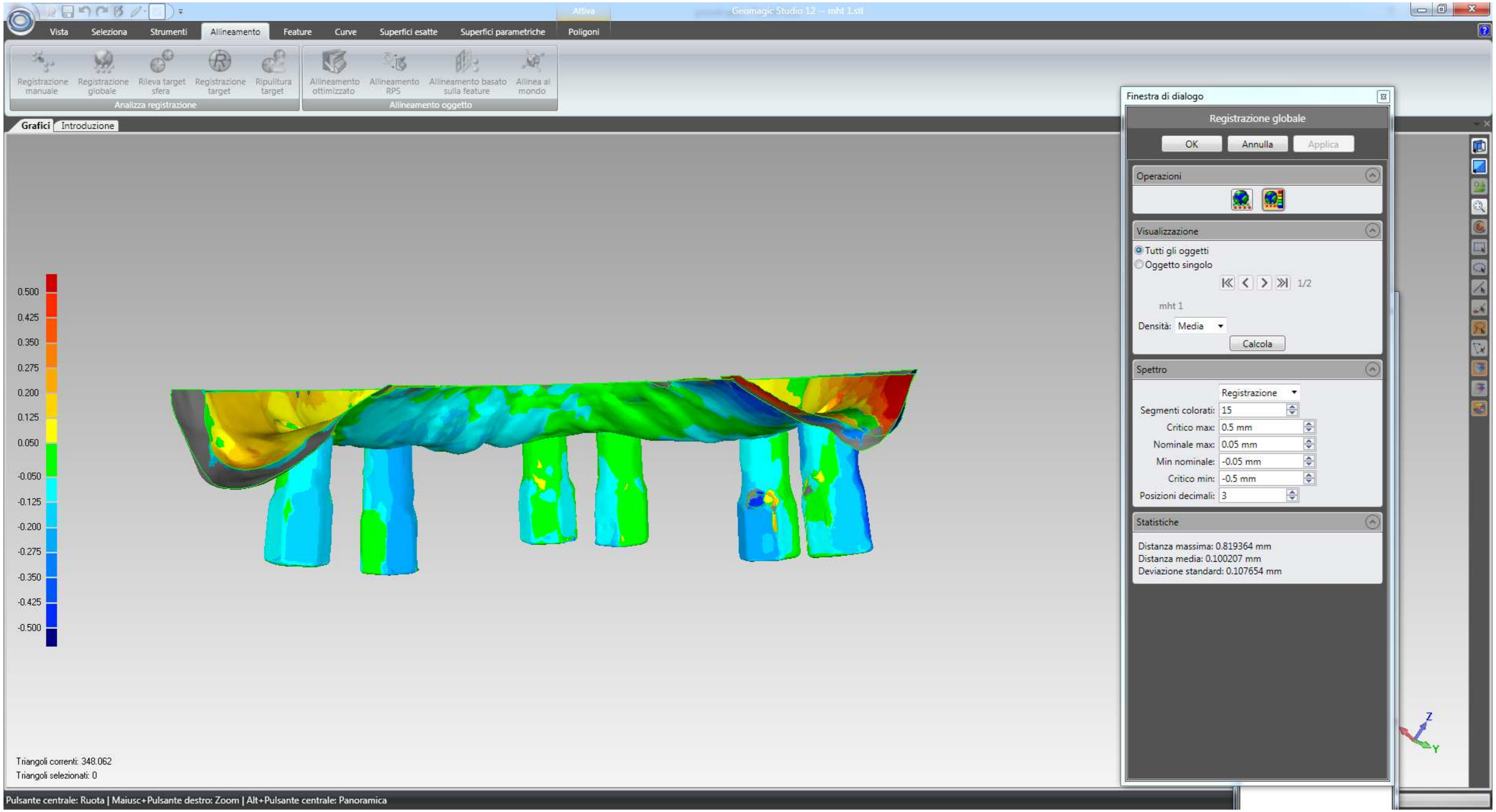

Zfx 1 as reference (best accuracy) vs Zfx 3

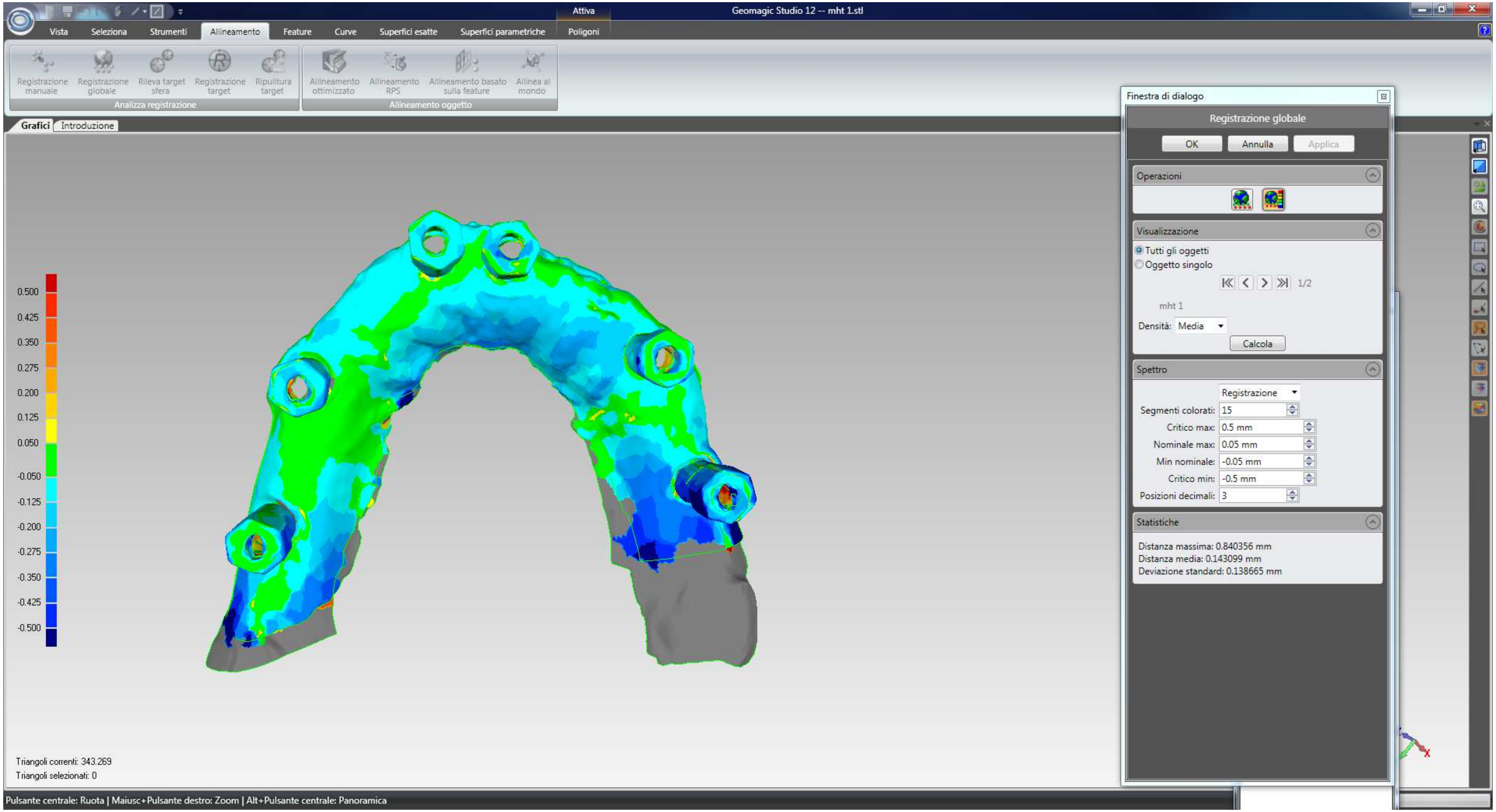

Zfx 1 as reference (best accuracy) vs Zfx 3

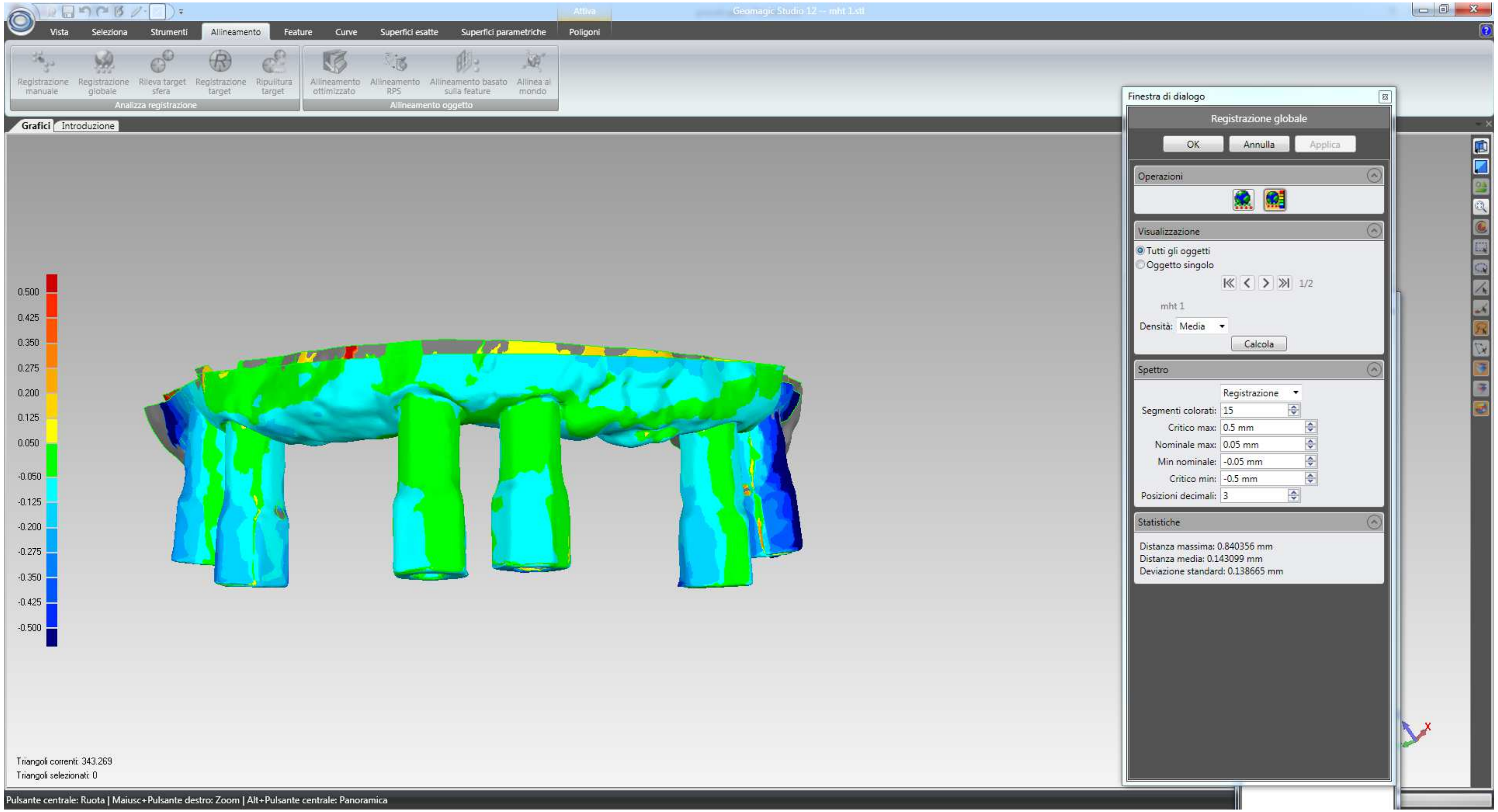

Zfx 1 as reference (best accuracy) vs Zfx 3

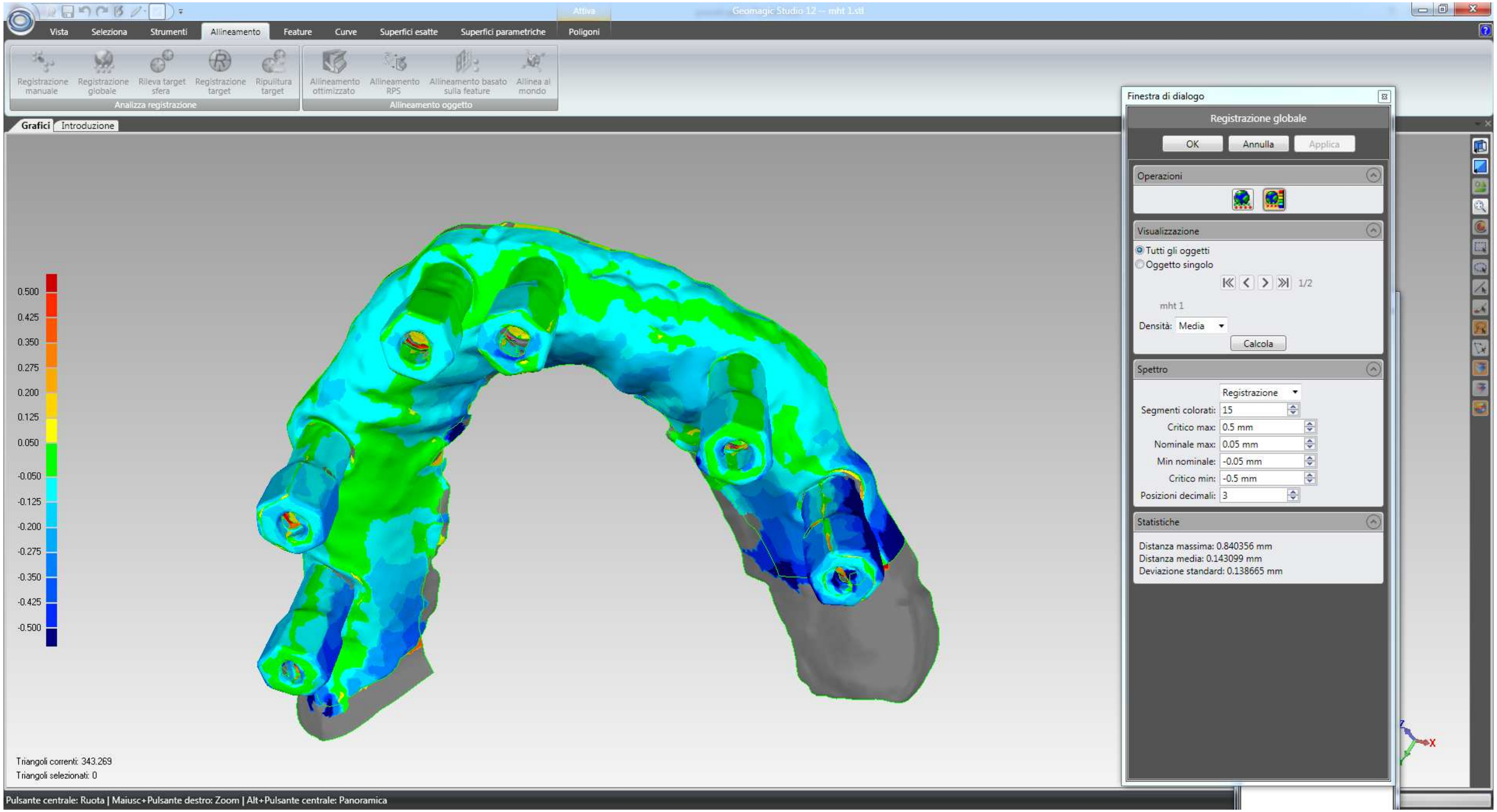

Zfx 1 as reference (best accuracy) vs Zfx 3

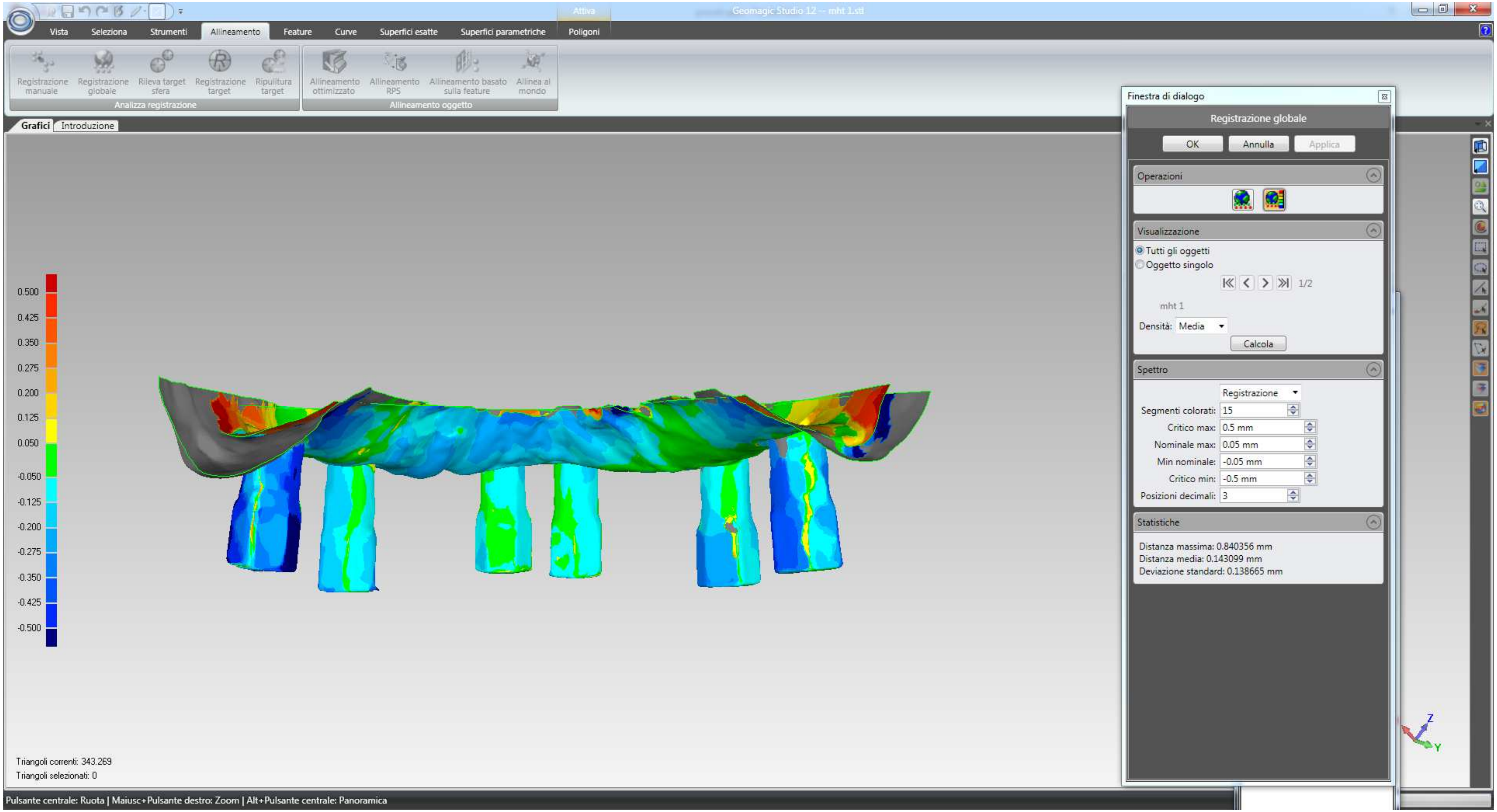

Zfx 1 as reference (best accuracy) vs Zfx 4

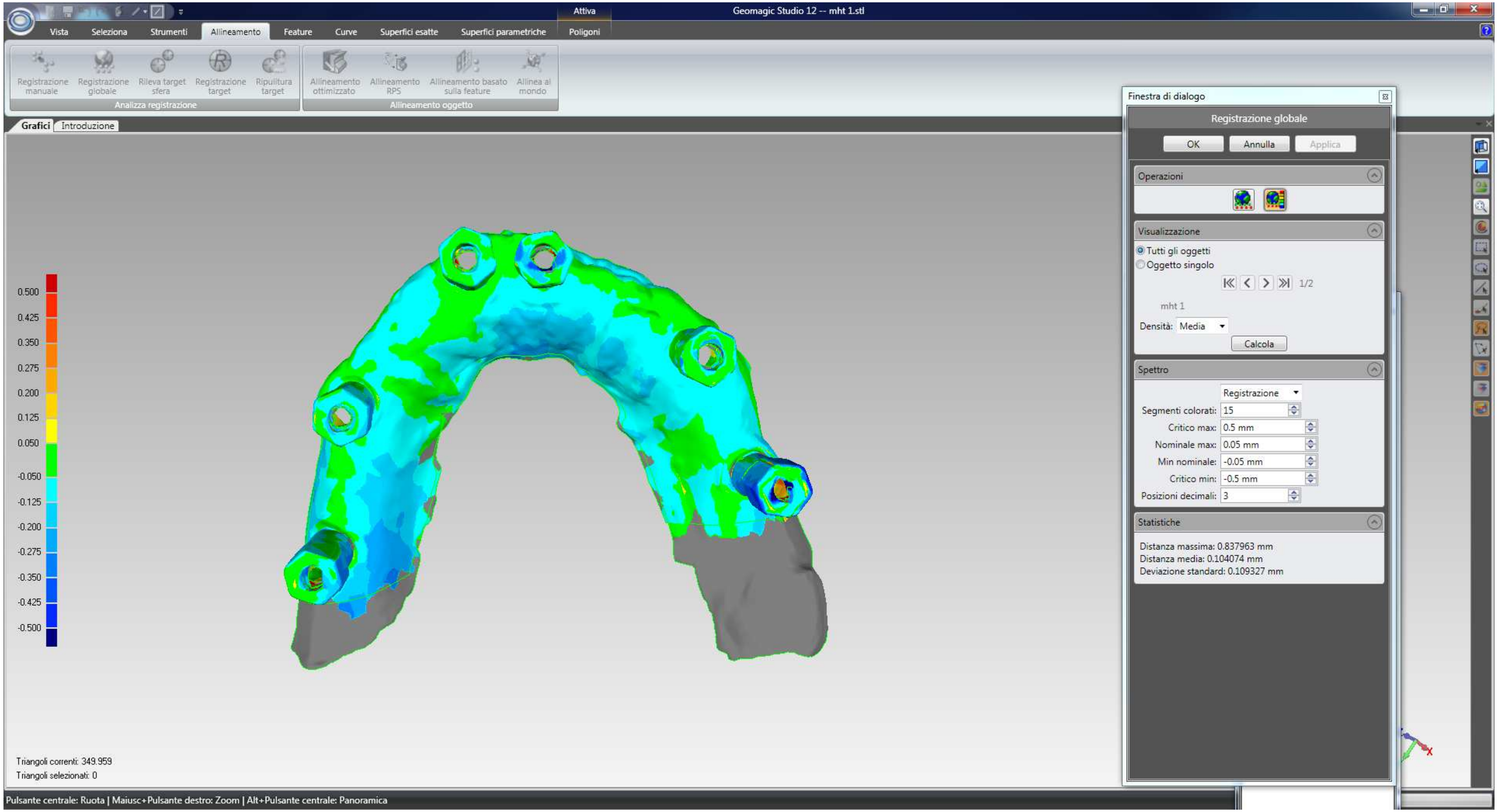

Zfx 1 as reference (best accuracy) vs Zfx 4

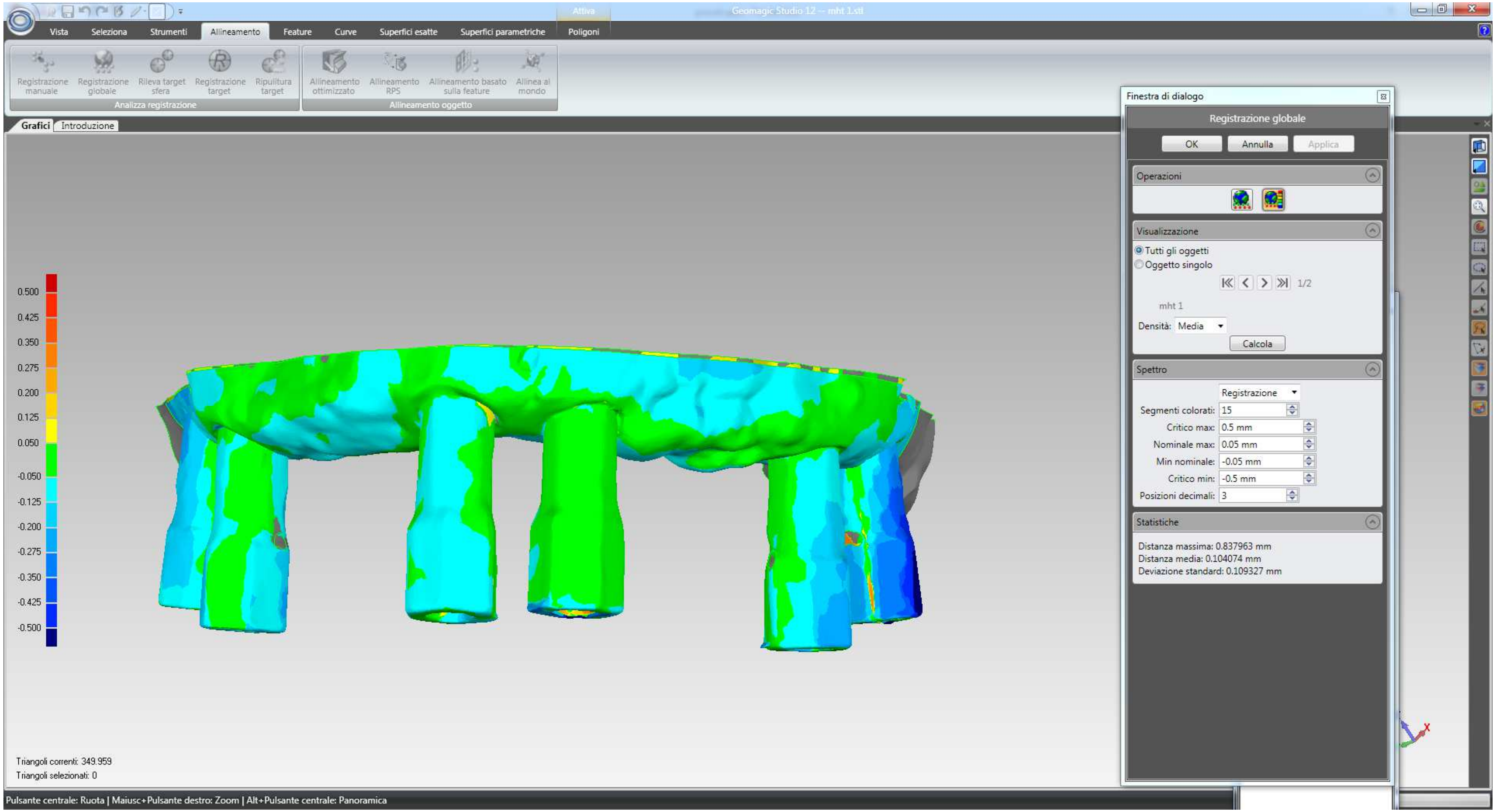

Zfx 1 as reference (best accuracy) vs Zfx 4

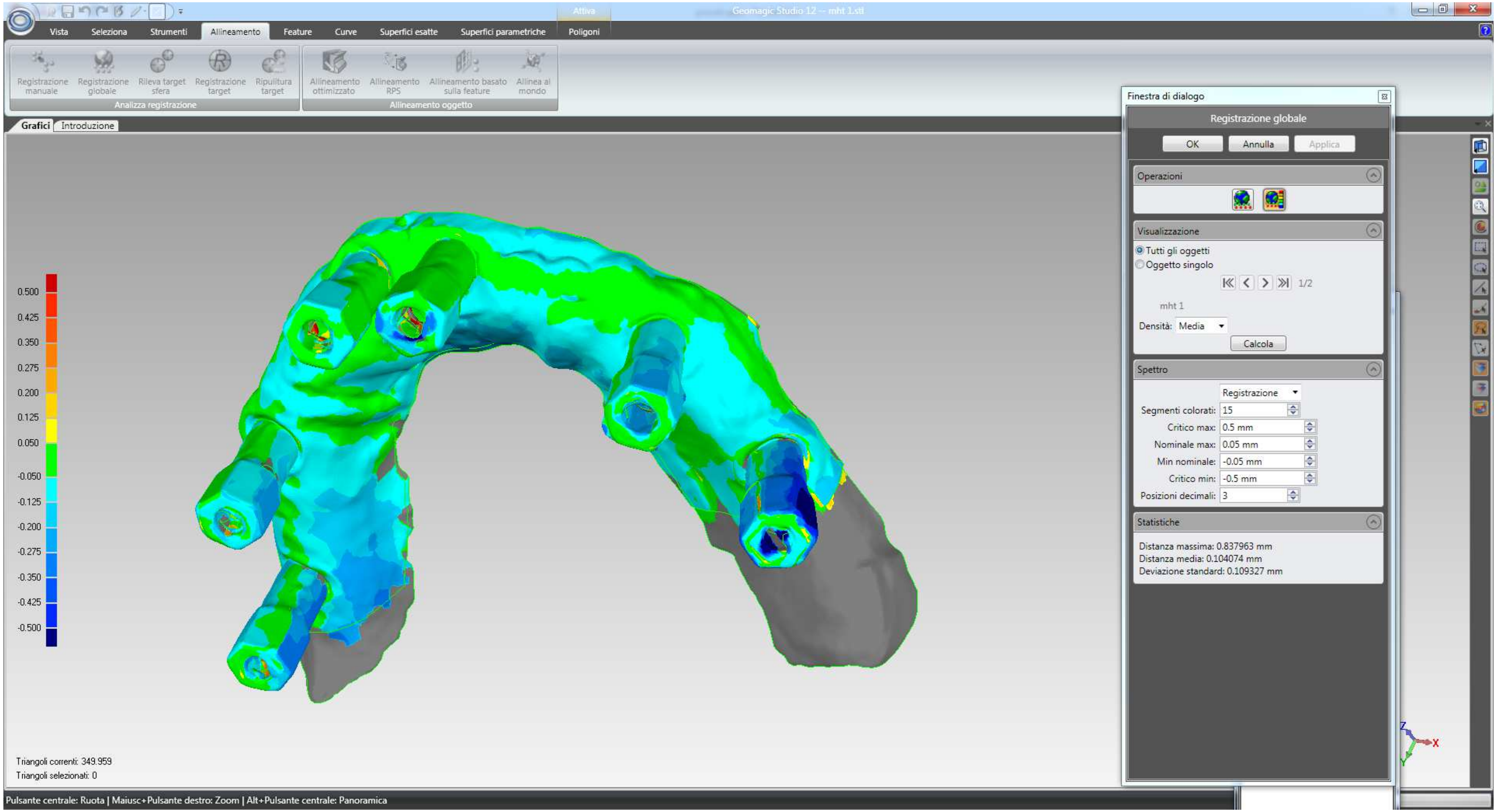

Zfx 1 as reference (best accuracy) vs Zfx 4

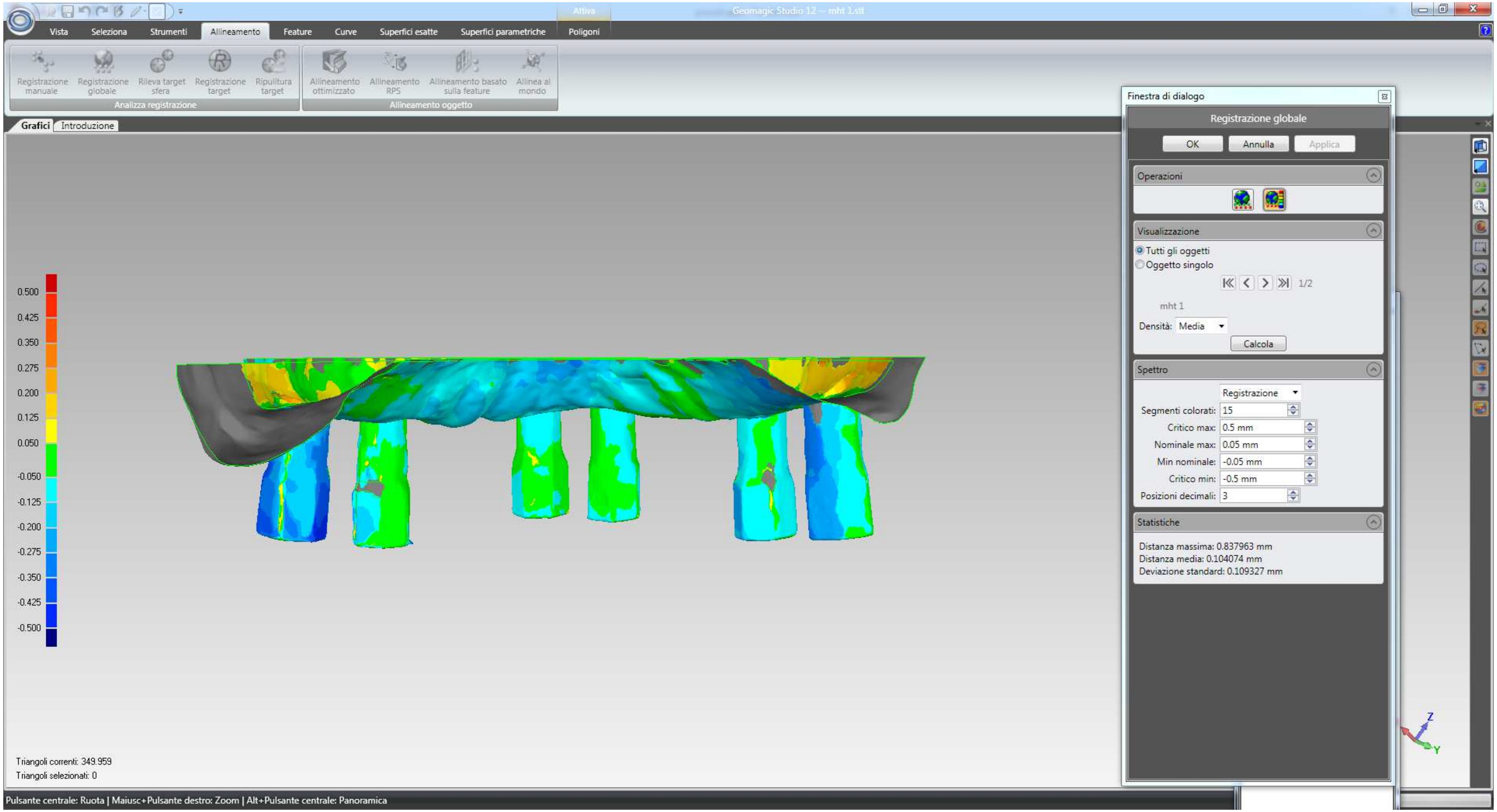

Zfx 1 as reference (best accuracy) vs Zfx 5

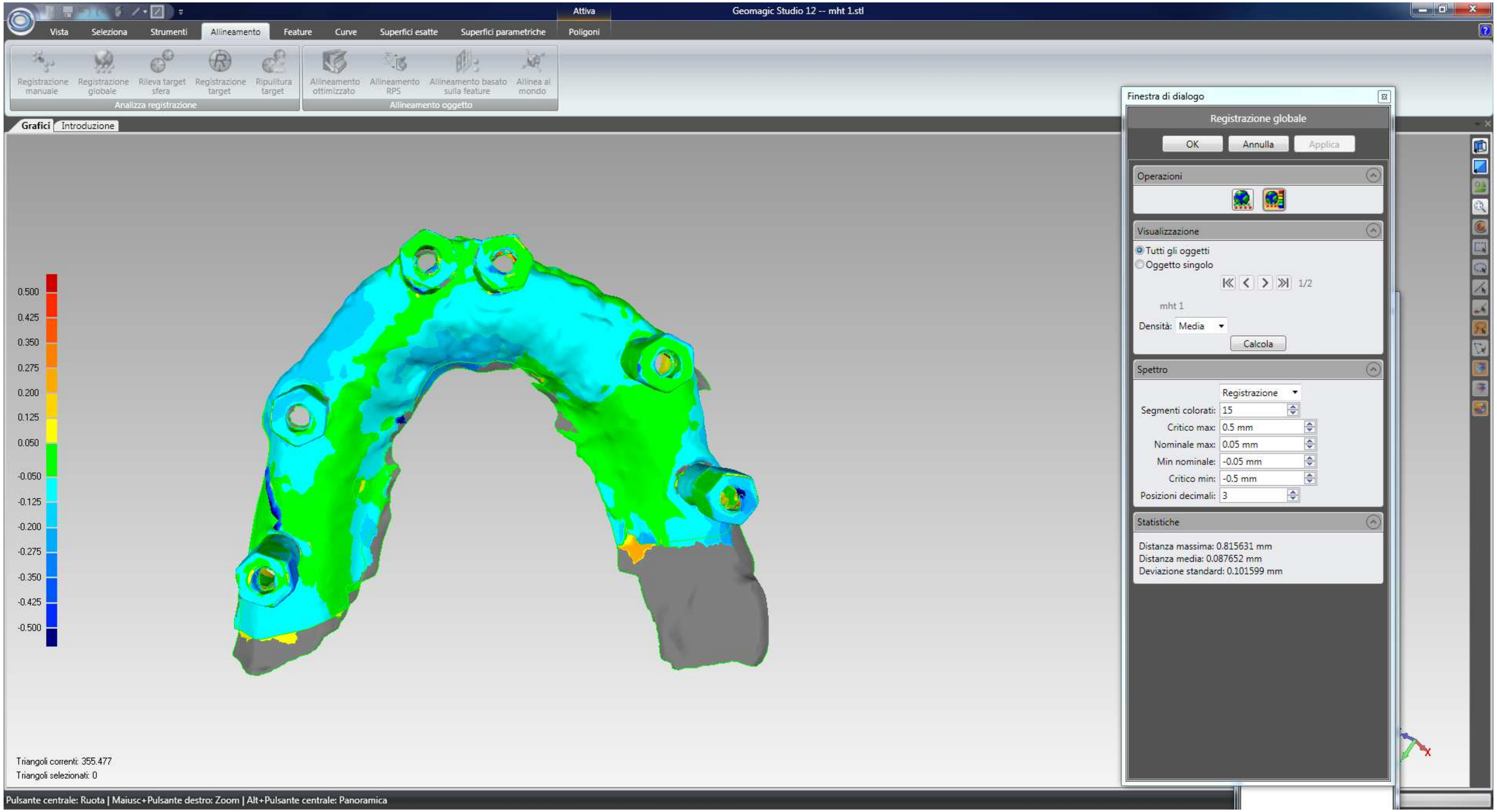

Zfx 1 as reference (best accuracy) vs Zfx 5

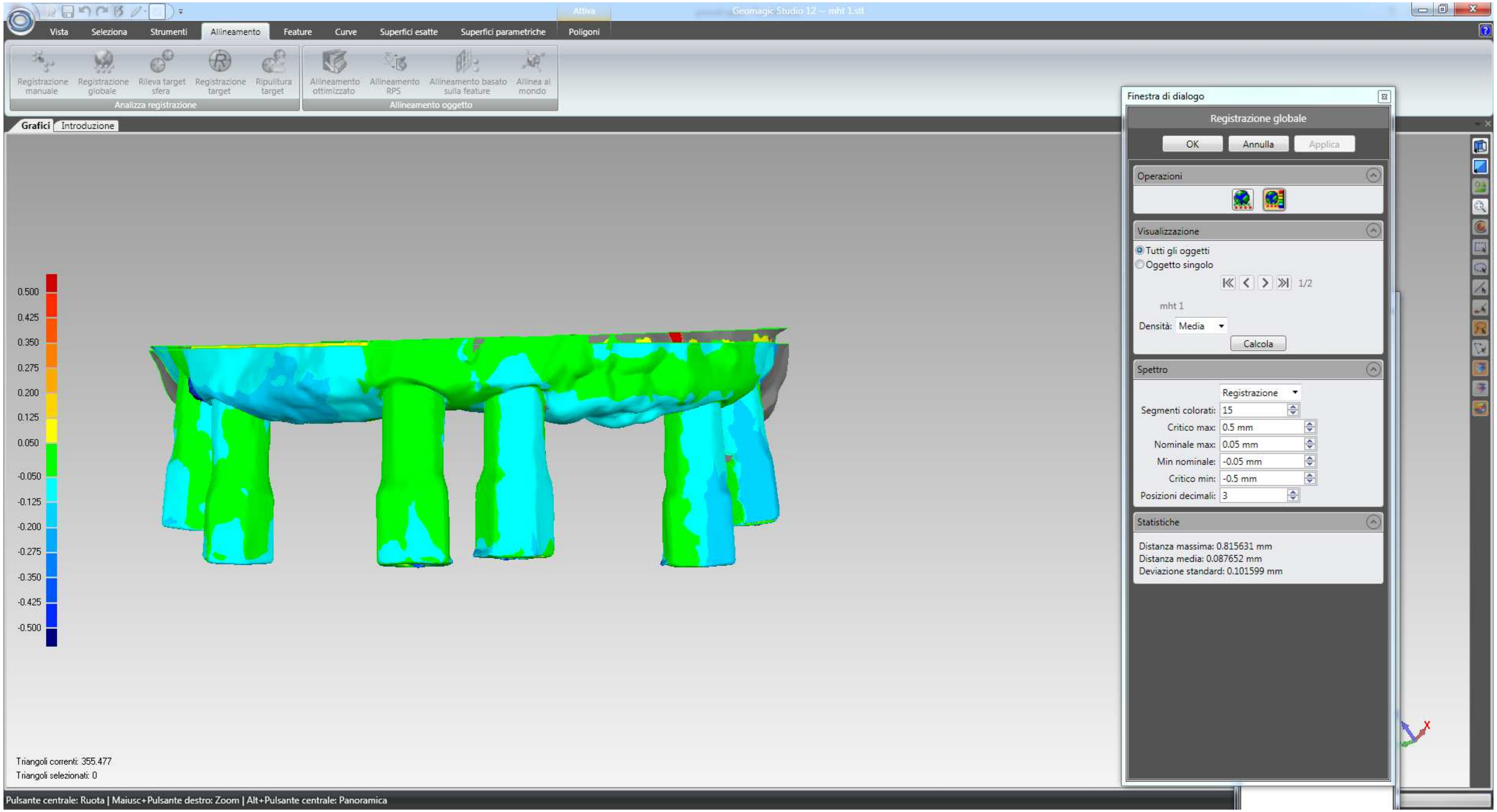

Zfx 1 as reference (best accuracy) vs Zfx 5

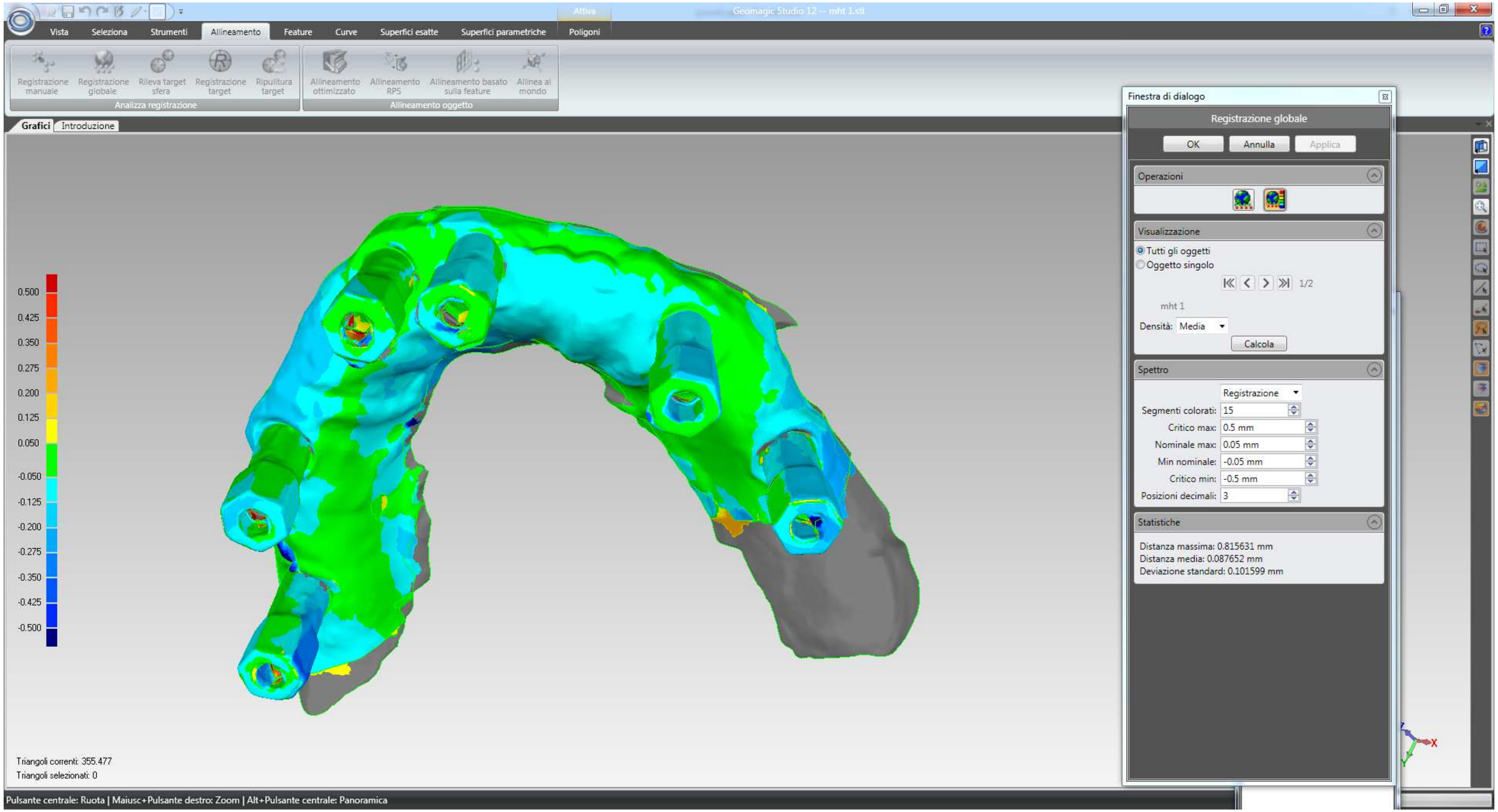

Zfx 1 as reference (best accuracy) vs Zfx 5

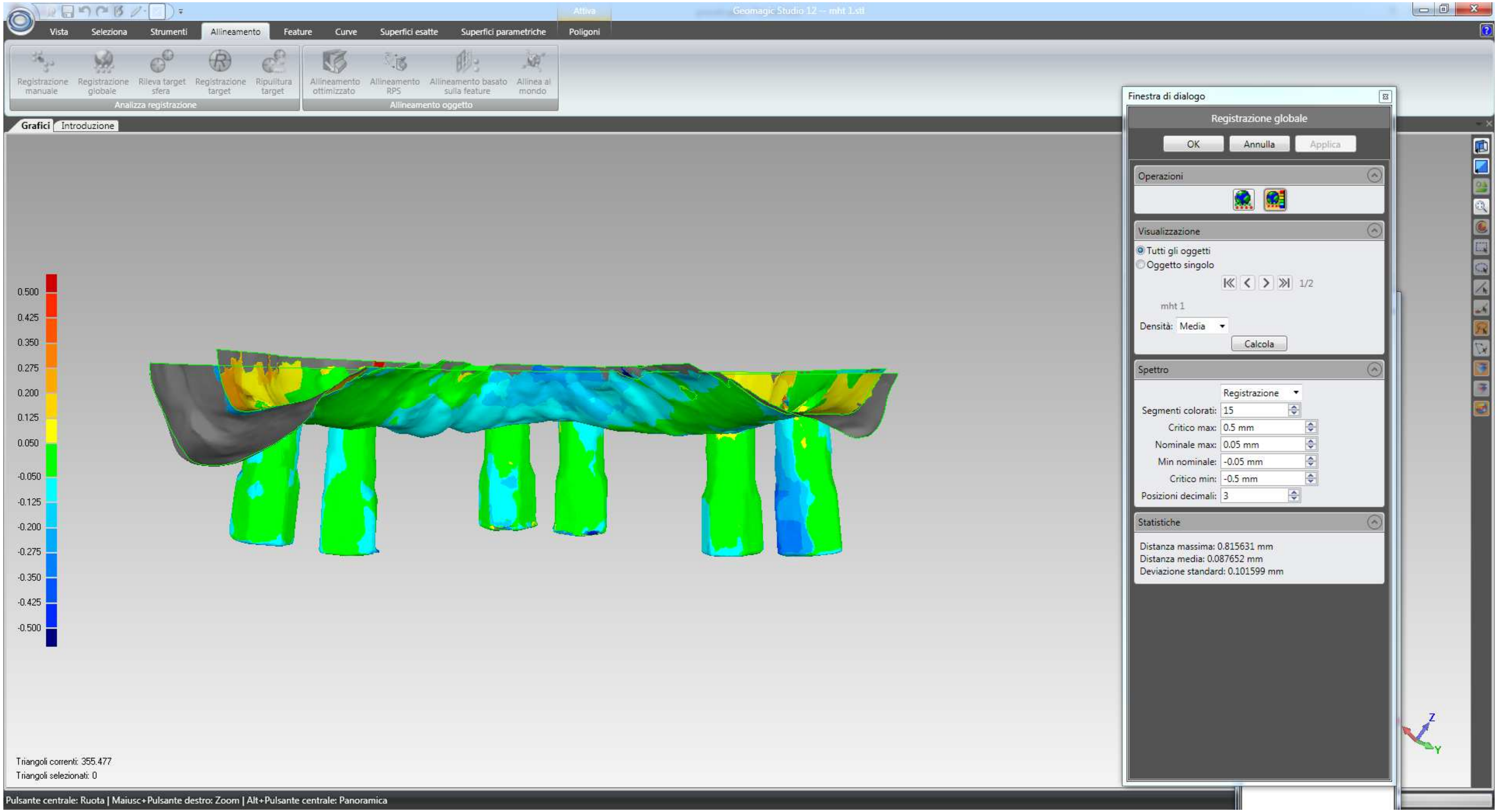

Zfx 2 vs Zfx 4

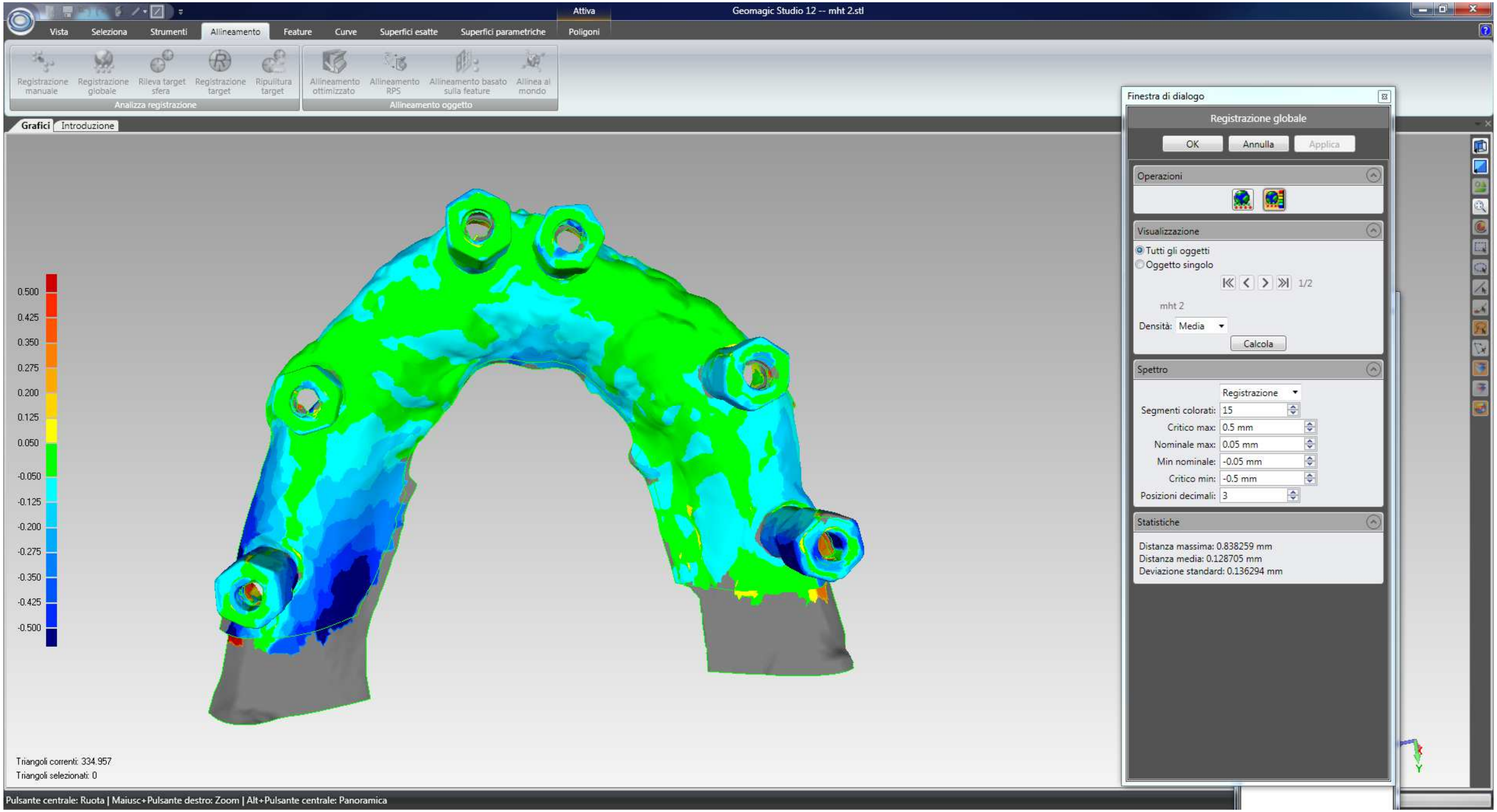

Attiva
Geomagic Studio 12 -- mht 2.stl
Vista
Seleziona
Strumenti
Allineamento
Feature
Curve
Superfici esatte
Superfici parametriche
Poligoni
Registrazione manuale
Registrazione globale
Rileva target sfera
Registrazione target
Ripulitura target
Analizza registrazione
Allineamento ottimizzato
Allineamento RPS
Allineamento basato sulla feature
Allinea al mondo
Allineamento oggetto
Grafici
Introduzione
0.500
0.425
0.350
0.275
0.200
0.125
0.050
-0.050
-0.125
-0.200
-0.275
-0.350
-0.425
-0.500
Triangoli correnti: 334.957
Triangoli selezionati: 0
Pulsante centrale: Ruota | Maiusc+Pulsante destro: Zoom | Alt+Pulsante centrale: Panoramica
Finestra di dialogo
Registrazione globale
OK
Annulla
Applica
Operazioni
Visualizzazione
Tutti gli oggetti
Oggetto singolo
1/2
mht 2
Densità: Media
Calcola
Spettro
Registrazione
Segmenti colorati: 15
Critico max: 0.5 mm
Nominale max: 0.05 mm
Min nominale: -0.05 mm
Critico min: -0.5 mm
Posizioni decimali: 3
Statistiche
Distanza massima: 0.838259 mm
Distanza media: 0.128705 mm
Deviazione standard: 0.136294 mm

Zfx 2 vs Zfx 4

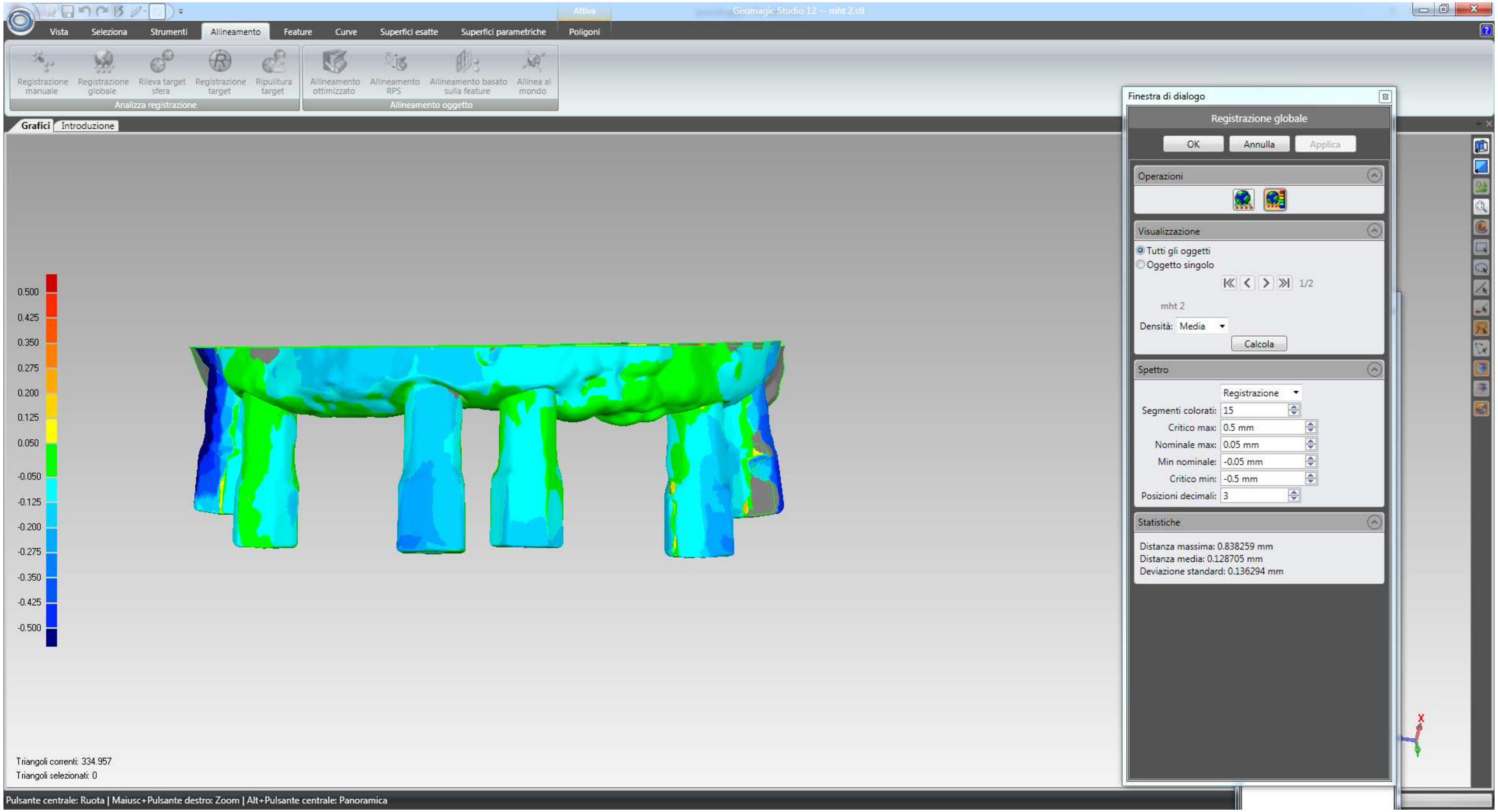
Geomagic Studio 12 -- mht 2.stl
Vista
Seleziona
Strumenti
Allineamento
Feature
Curve
Superfici esatte
Superfici parametriche
Poligoni
Registrazione manuale
Registrazione globale
Rileva target sfera
Registrazione target
Ripulitura target
Allineamento ottimizzato
Allineamento RPS
Allineamento basato sulla feature
Allinea al mondo
Analizza registrazione
Allineamento oggetto
Grafici
Introduzione
0.500
0.425
0.350
0.275
0.200
0.125
0.050
-0.050
-0.125
-0.200
-0.275
-0.350
-0.425
-0.500
Triangoli correnti: 334.957
Triangoli selezionati: 0
Pulsante centrale: Ruota | Maiusc+Pulsante destro: Zoom | Alt+Pulsante centrale: Panoramica
Finestra di dialogo
Registrazione globale
OK
Annulla
Applica
Operazioni
Visualizzazione
Tutti gli oggetti
Oggetto singolo
1/2
mht 2
Densità: Media
Calcola
Spettro
Registrazione
Segmenti colorati: 15
Critico max: 0.5 mm
Nominale max: 0.05 mm
Min nominale: -0.05 mm
Critico min: -0.5 mm
Posizioni decimali: 3
Statistiche
Distanza massima: 0.838259 mm
Distanza media: 0.128705 mm
Deviazione standard: 0.136294 mm

Zfx 2 vs Zfx 4

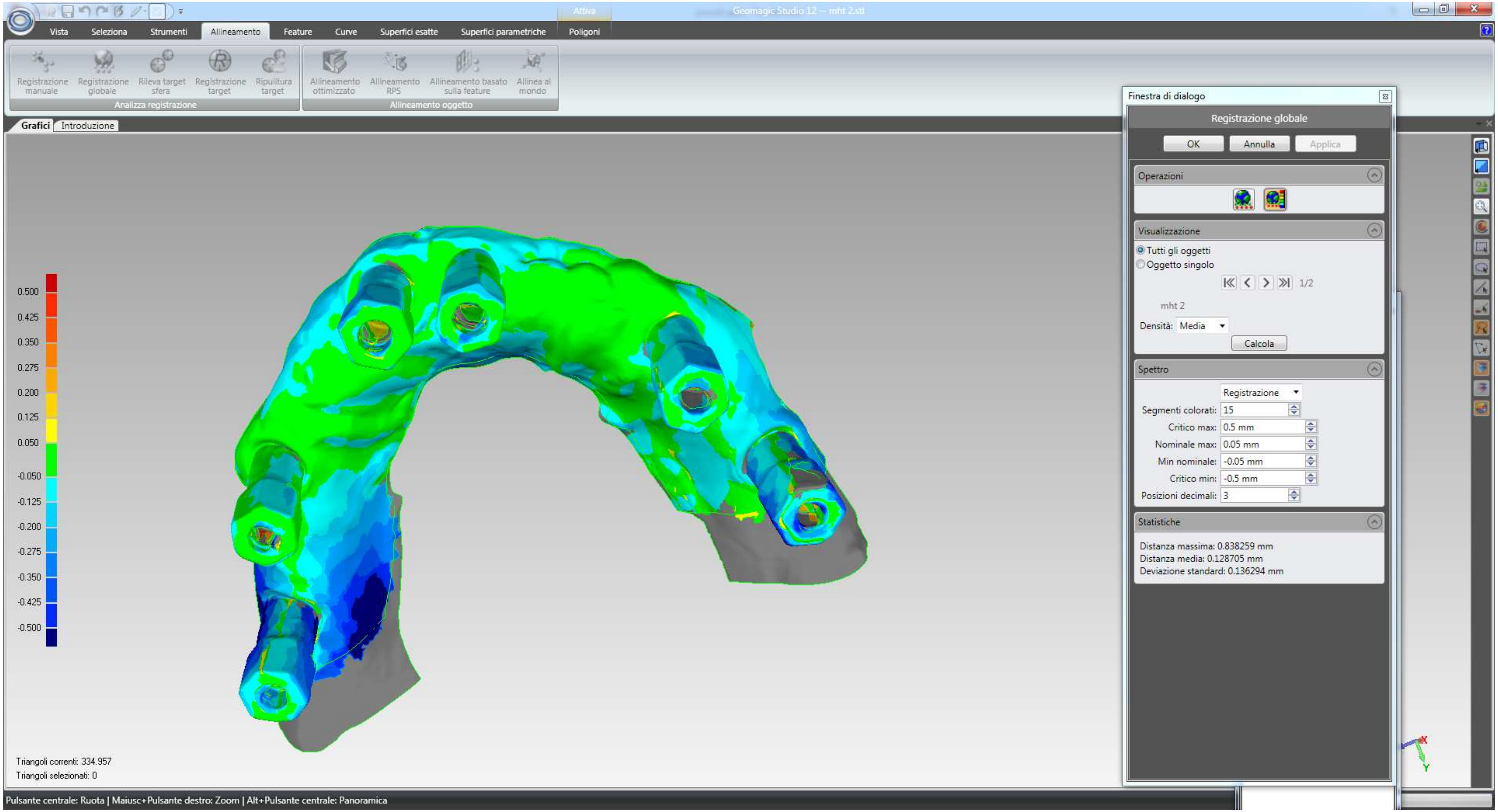
Geomagic Studio 12 -- mht 2.stl
Vista
Seleziona
Strumenti
Allineamento
Feature
Curve
Superfici esatte
Superfici parametriche
Poligoni
Registrazione manuale
Registrazione globale
Rileva target sfera
Registrazione target
Ripulitura target
Analizza registrazione
Allineamento ottimizzato
Allineamento RPS
Allineamento basato sulla feature
Allinea al mondo
Allineamento oggetto
Grafici
Introduzione
0.500
0.425
0.350
0.275
0.200
0.125
0.050
-0.050
-0.125
-0.200
-0.275
-0.350
-0.425
-0.500
Triangoli correnti: 334.957
Triangoli selezionati: 0
Finestra di dialogo
Registrazione globale
OK
Annulla
Applica
Operazioni
Visualizzazione
Tutti gli oggetti
Oggetto singolo
1/2
mht 2
Densità: Media
Calcola
Spettro
Registrazione
Segmenti colorati: 15
Critico max: 0.5 mm
Nominale max: 0.05 mm
Min nominale: -0.05 mm
Critico min: -0.5 mm
Posizioni decimali: 3
Statistiche
Distanza massima: 0.838259 mm
Distanza media: 0.128705 mm
Deviazione standard: 0.136294 mm
Pulsante centrale: Ruota | Maiusc+Pulsante destro: Zoom | Alt+Pulsante centrale: Panoramica

Zfx 2 vs Zfx 4

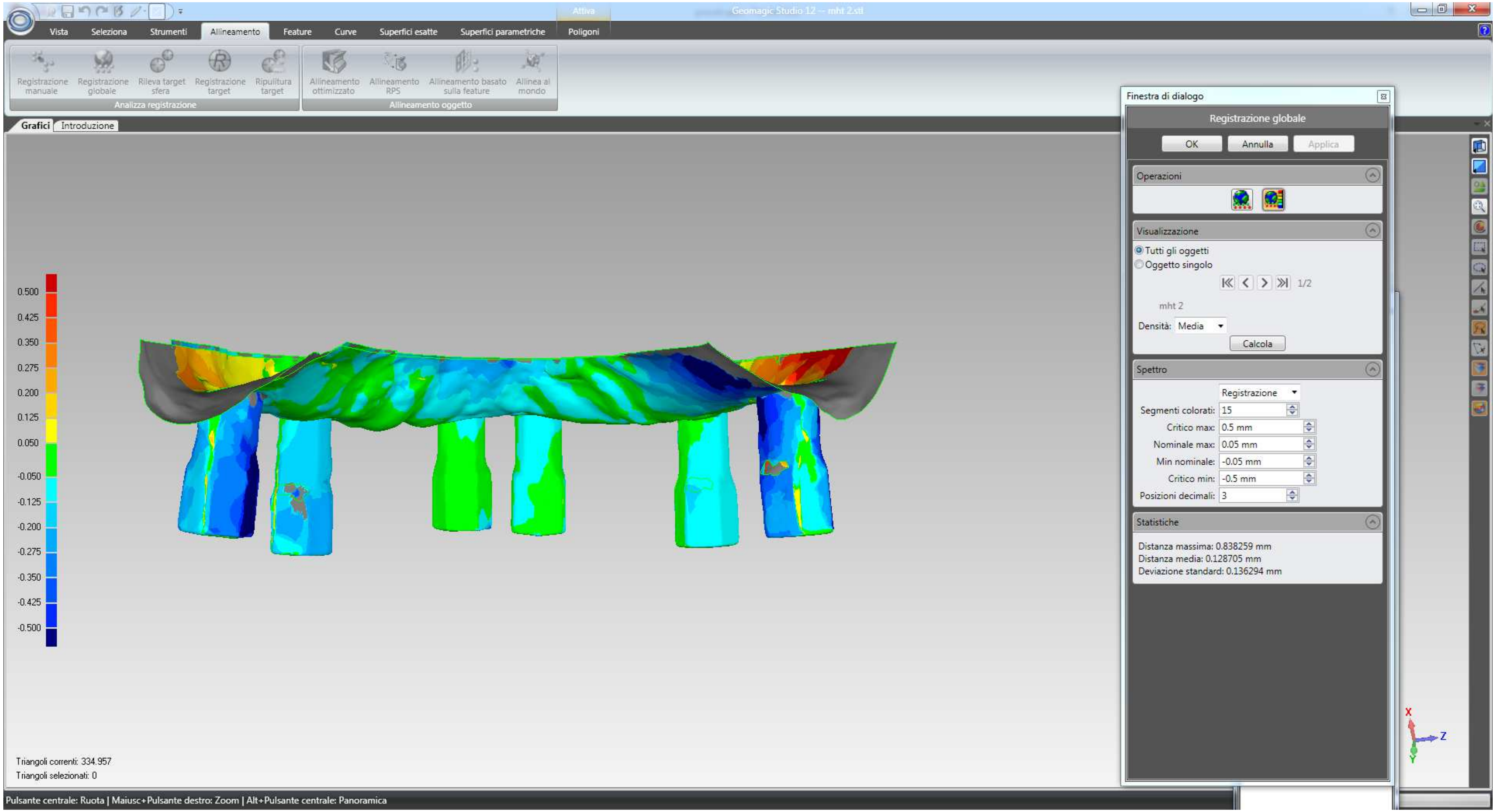
Geomagic Studio 12 -- mht 2.stl
Vista
Seleziona
Strumenti
Allineamento
Feature
Curve
Superfici esatte
Superfici parametriche
Poligoni
Registrazione manuale
Registrazione globale
Rileva target sfera
Registrazione target
Ripulitura target
Analizza registrazione
Allineamento ottimizzato
Allineamento RPS
Allineamento basato sulla feature
Allinea al mondo
Allineamento oggetto
Grafici
Introduzione
0.500
0.425
0.350
0.275
0.200
0.125
0.050
-0.050
-0.125
-0.200
-0.275
-0.350
-0.425
-0.500
Triangoli correnti: 334.957
Triangoli selezionati: 0
Finestra di dialogo
Registrazione globale
OK
Annulla
Applica
Operazioni
Visualizzazione
Tutti gli oggetti
Oggetto singolo
1/2
mht 2
Densità: Media
Calcola
Spettro
Registrazione
Segmenti colorati: 15
Critico max: 0.5 mm
Nominale max: 0.05 mm
Min nominale: -0.05 mm
Critico min: -0.5 mm
Posizioni decimali: 3
Statistiche
Distanza massima: 0.838259 mm
Distanza media: 0.128705 mm
Deviazione standard: 0.136294 mm
Pulsante centrale: Ruota | Maiusc+Pulsante destro: Zoom | Alt+Pulsante centrale: Panoramica

General precision Zfx in the totally edentulous patient

	Mean distance	SD	Maximum distance
Zfx 1 vs Zfx 2	0.100	0.107	0.819
Zfx 1 vs Zfx 3	0.143	0.138	0.840
Zfx 1 vs Zfx 4	0.104	0.109	0.837
Zfx 1 vs Zfx 5	0.087	0.101	0.815
Zfx 2 vs Zfx 4	0.128	0.136	0.838

Overall Mht general precision: 0.112 (0.022)

Care 4 as reference (best accuracy) vs Care 1

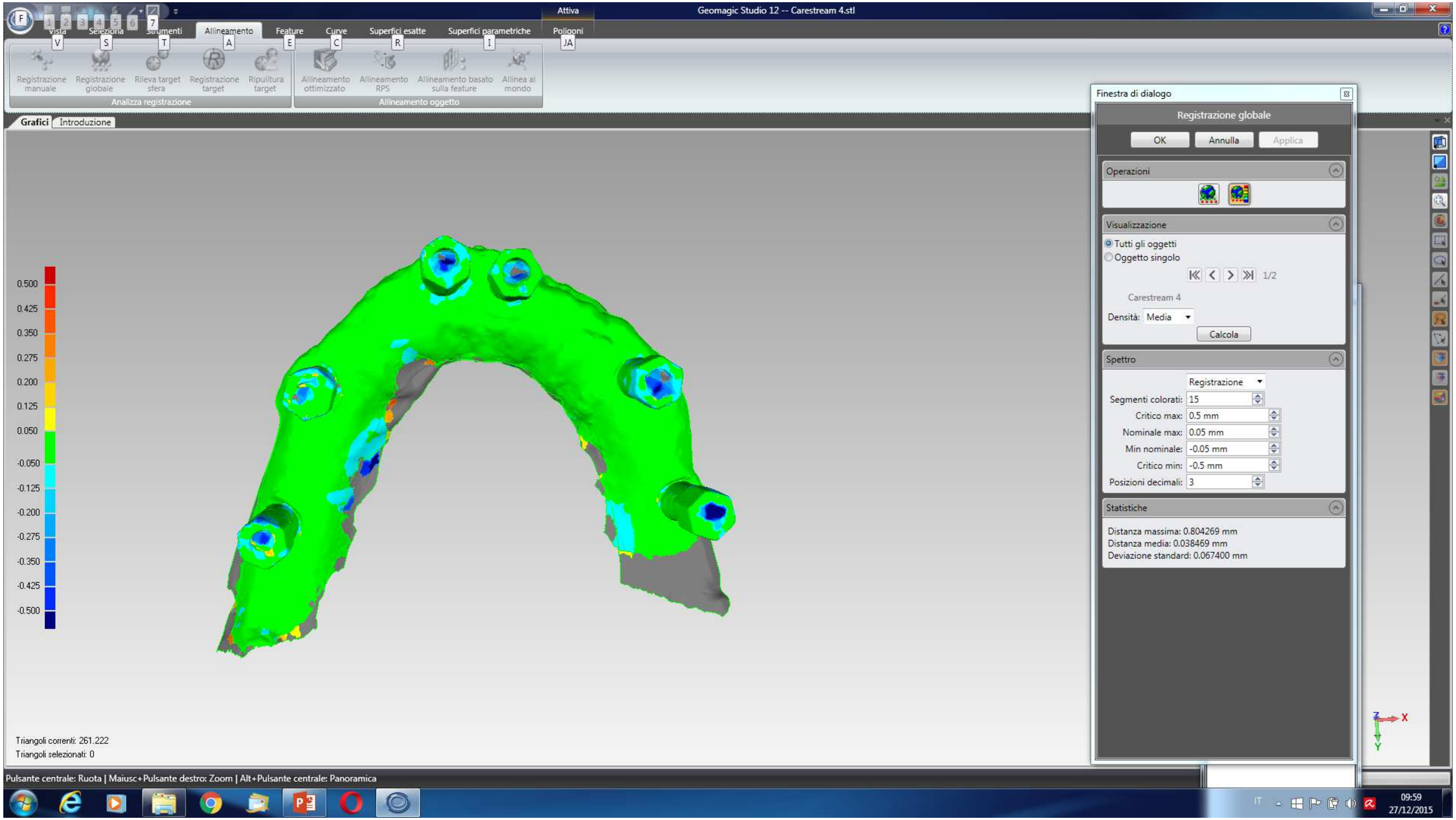

Care 4 as reference (best accuracy) vs Care 1

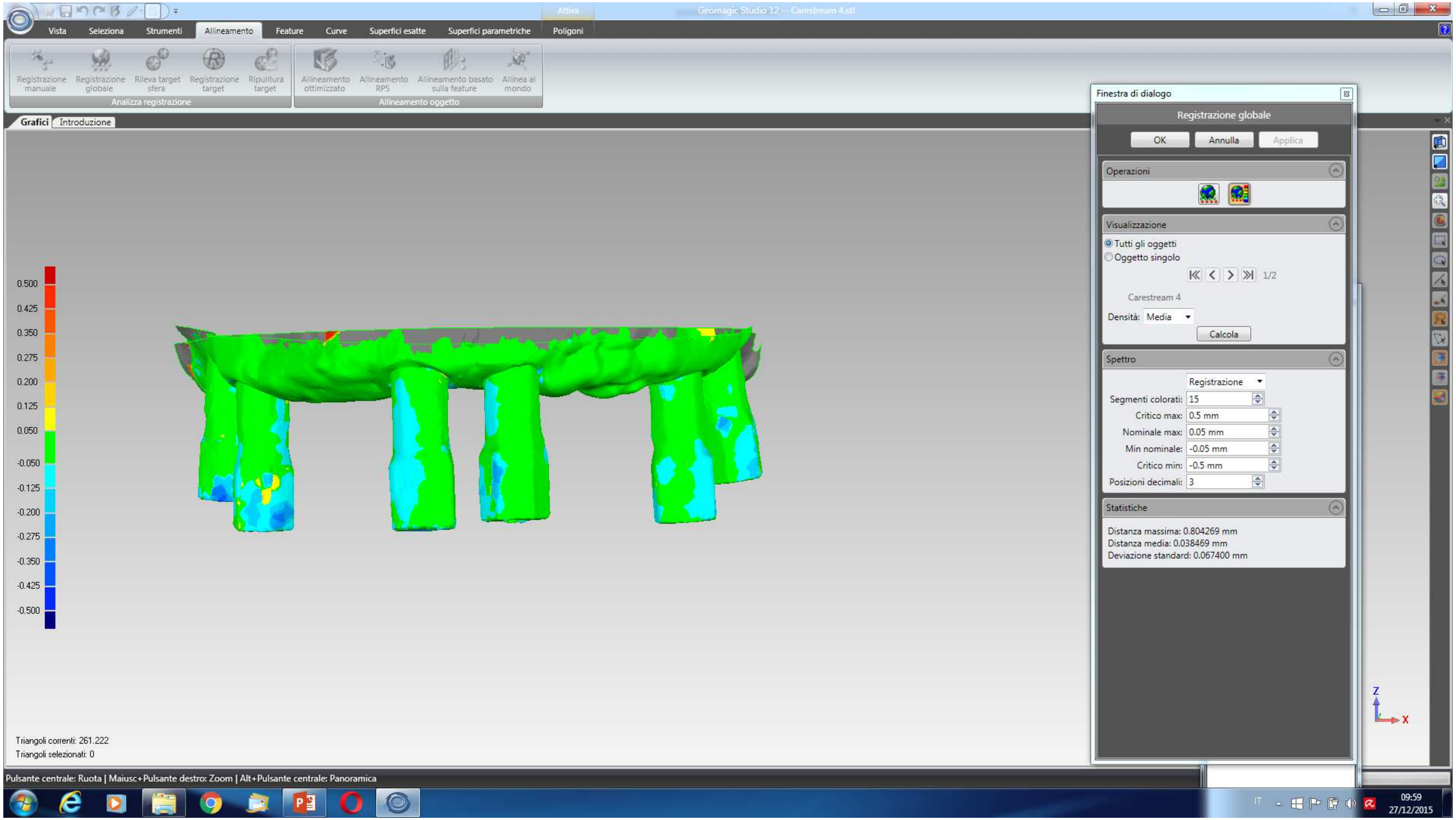

Care 4 as reference (best accuracy) vs Care 1

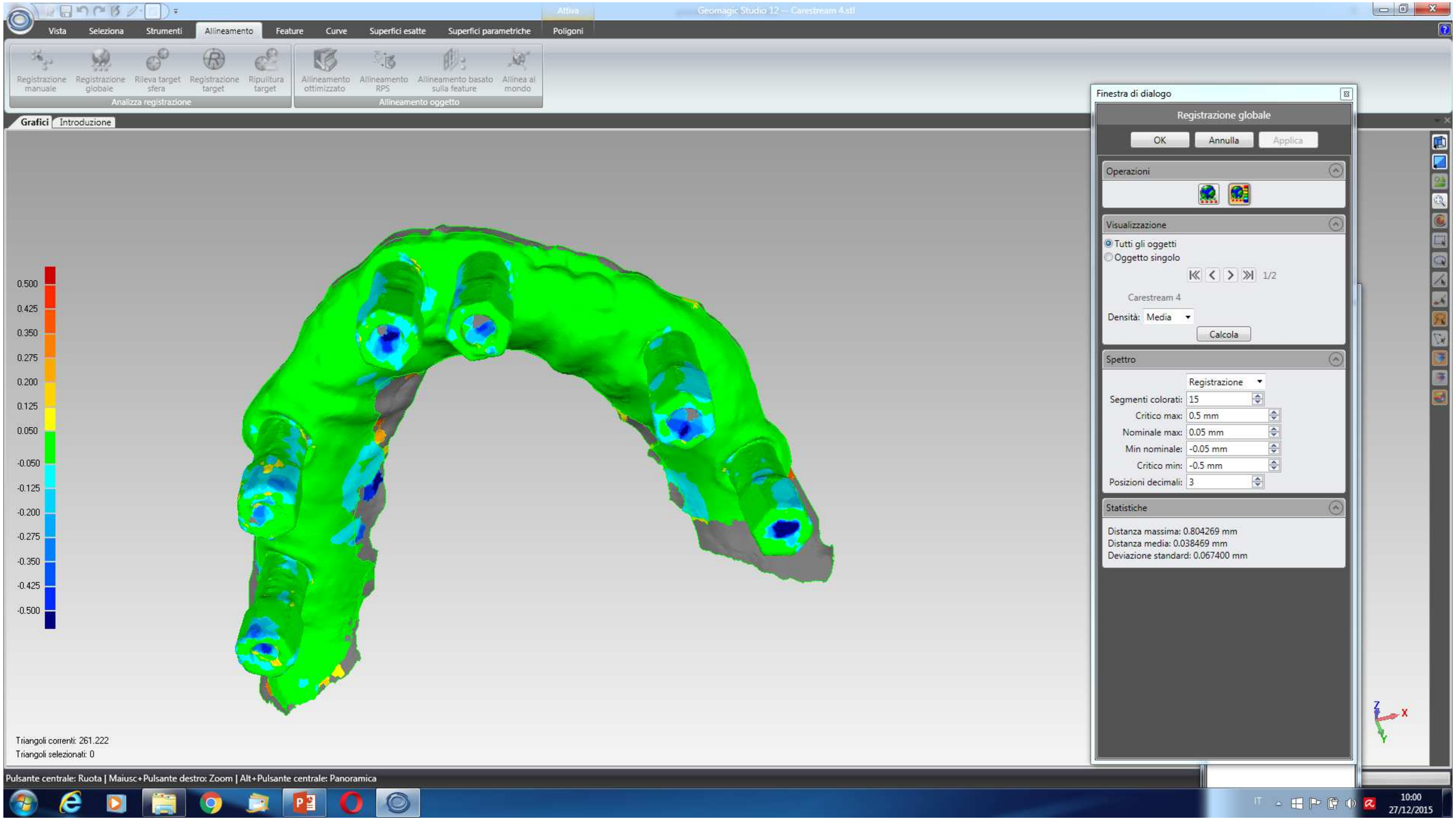

Care 4 as reference (best accuracy) vs Care 1

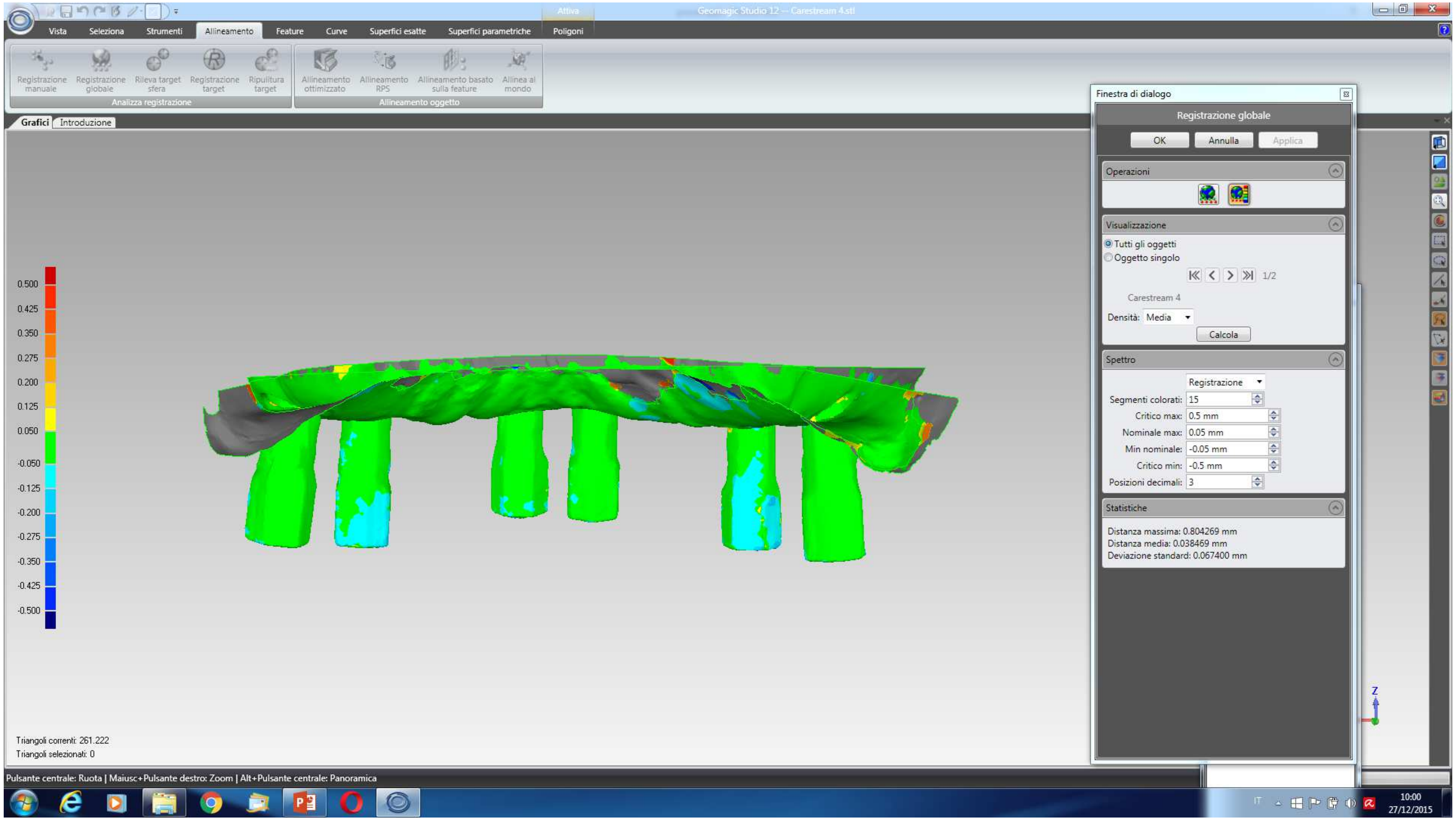

Care 4 as reference (best accuracy) vs Care 2

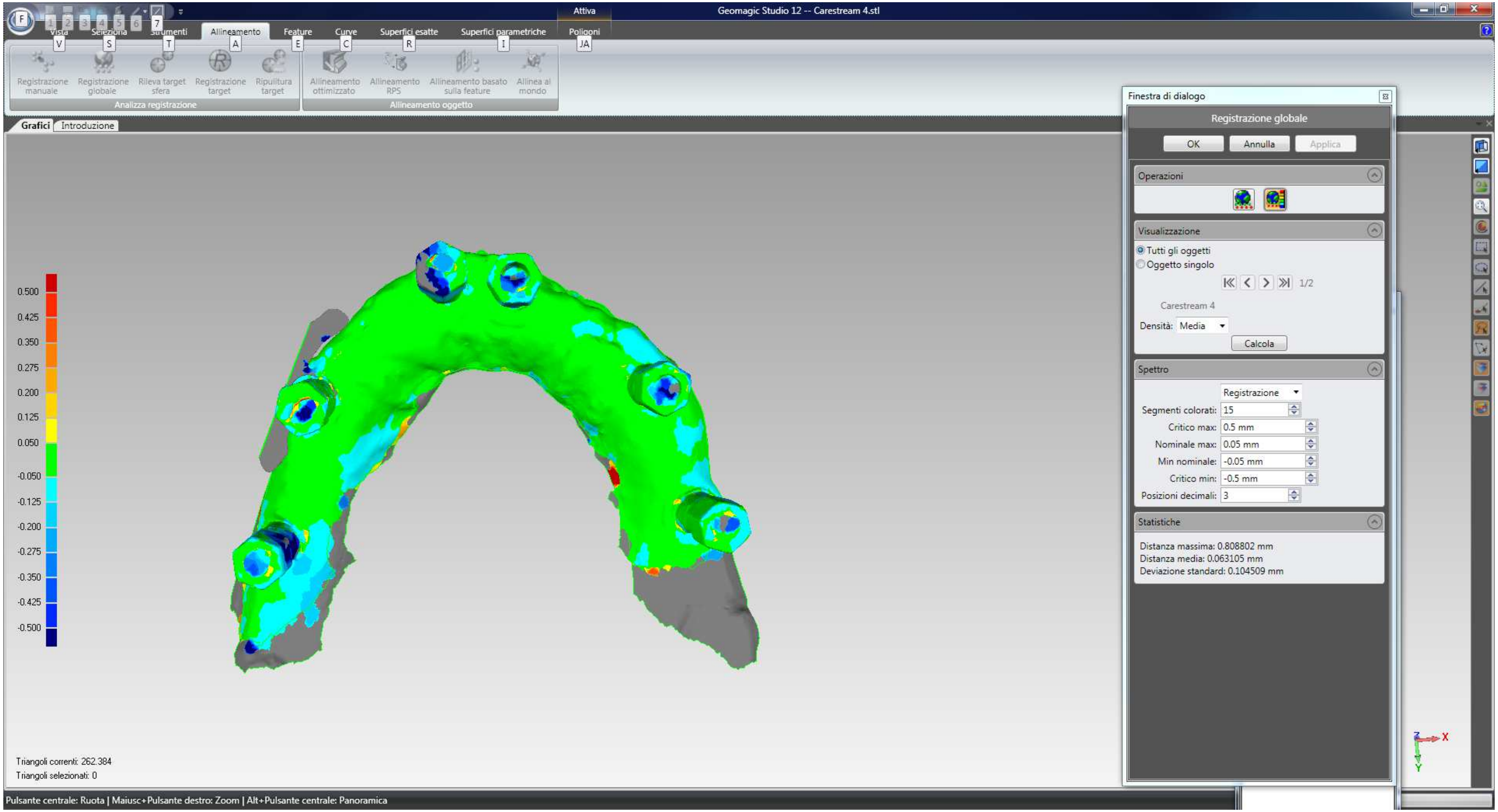

Care 4 as reference (best accuracy) vs Care 2

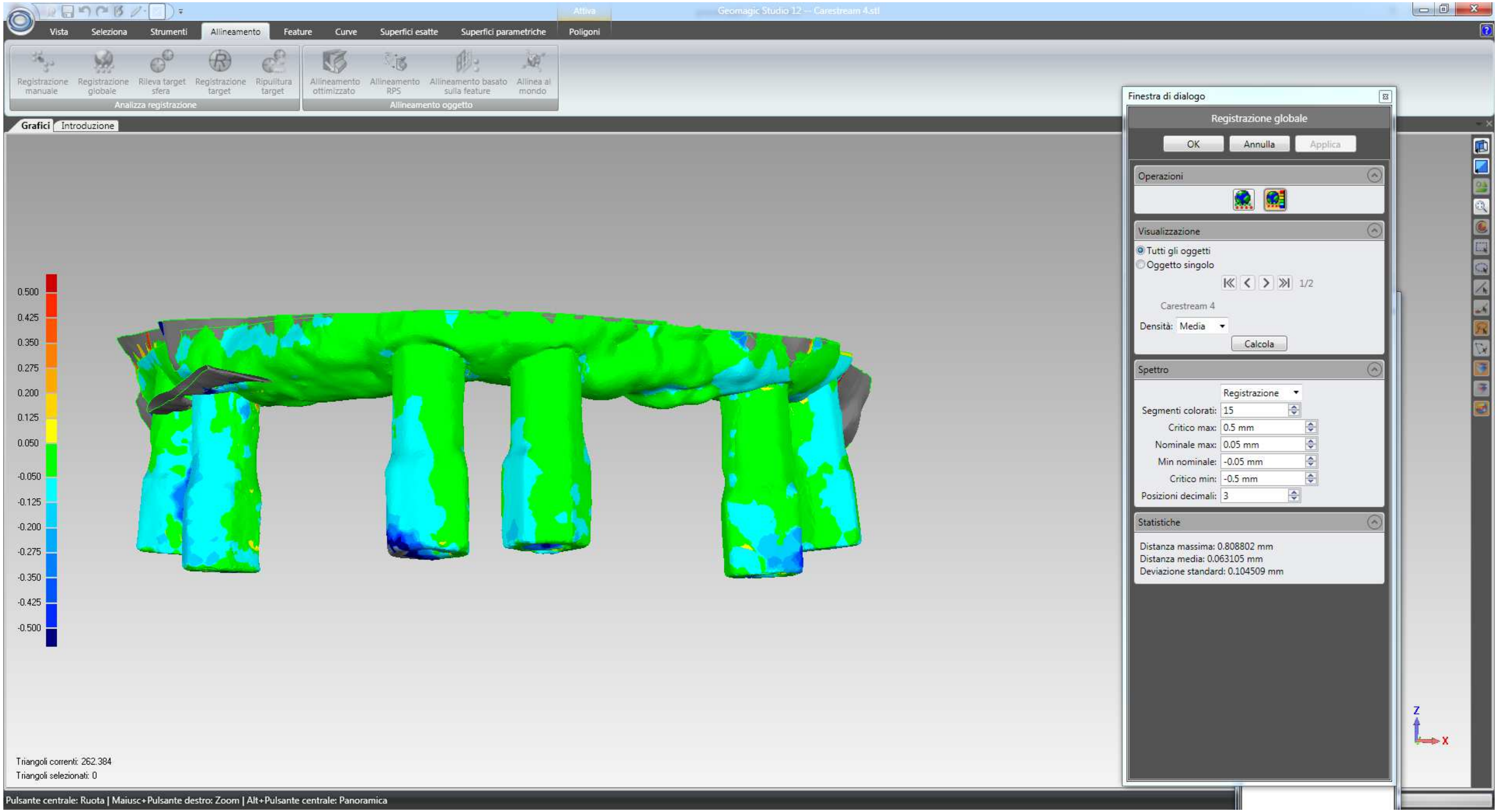

Care 4 as reference (best accuracy) vs Care 2

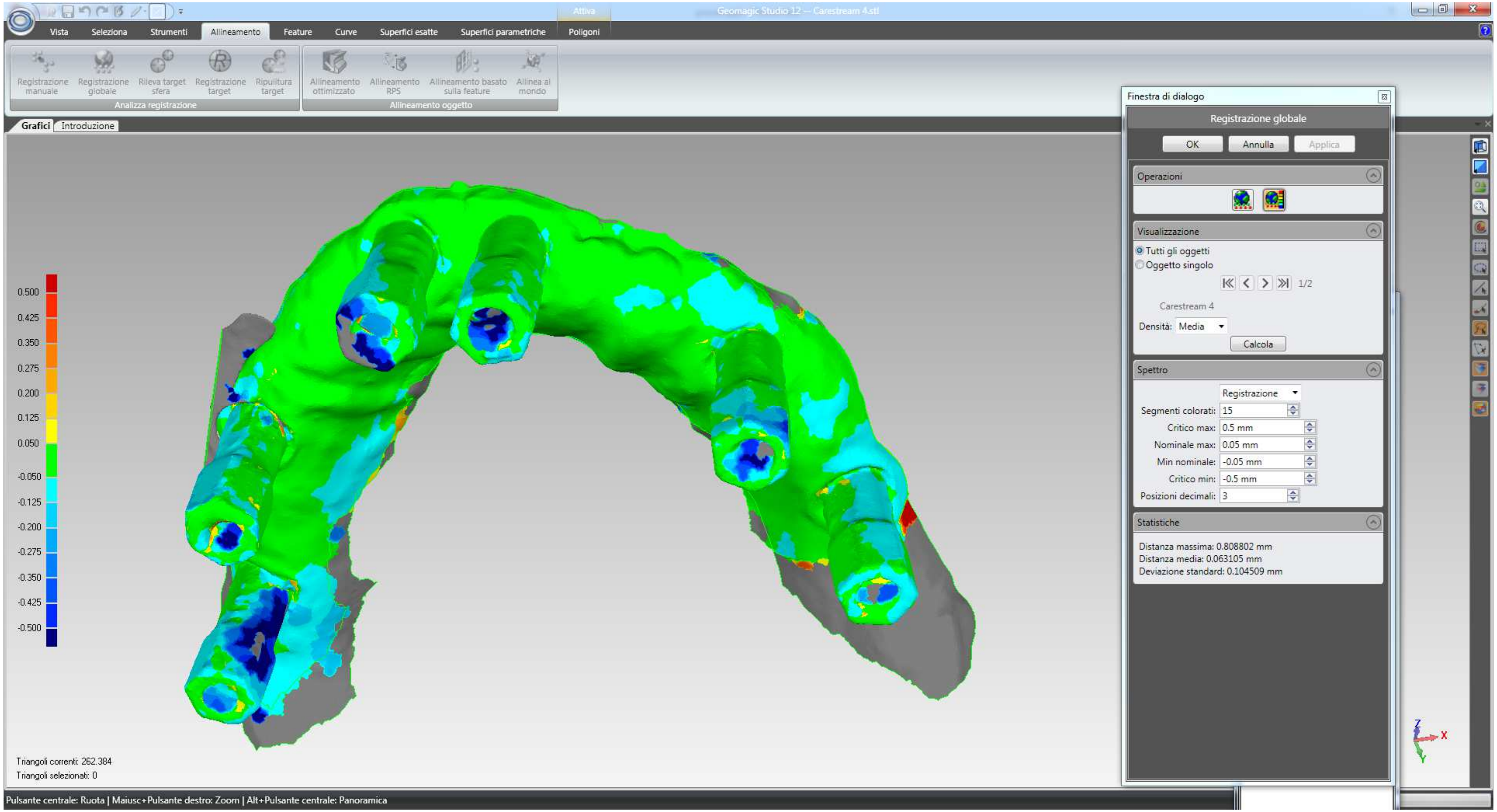

Care 4 as reference (best accuracy) vs Care 2

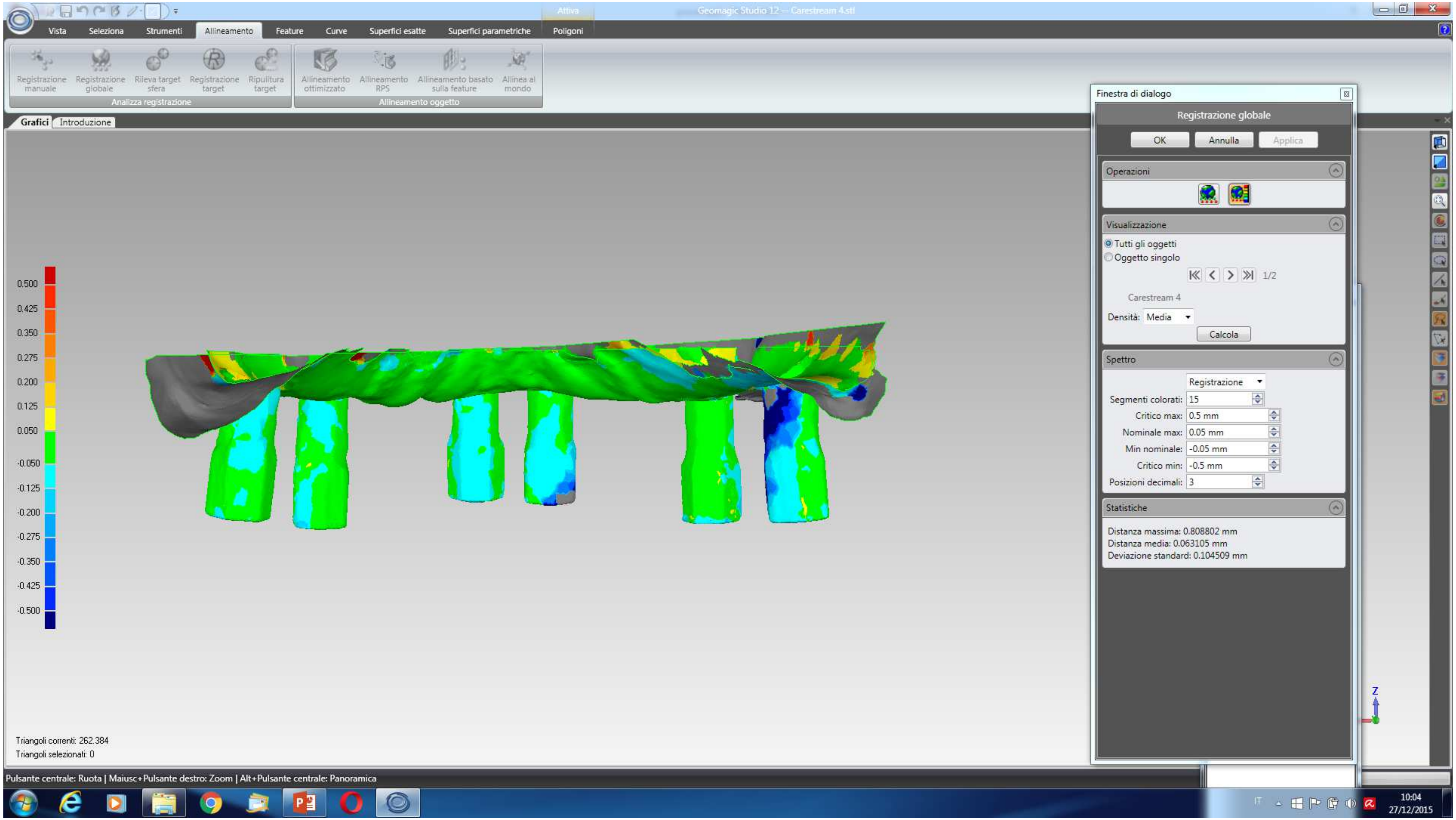

Care 4 as reference (best accuracy) vs Care 3

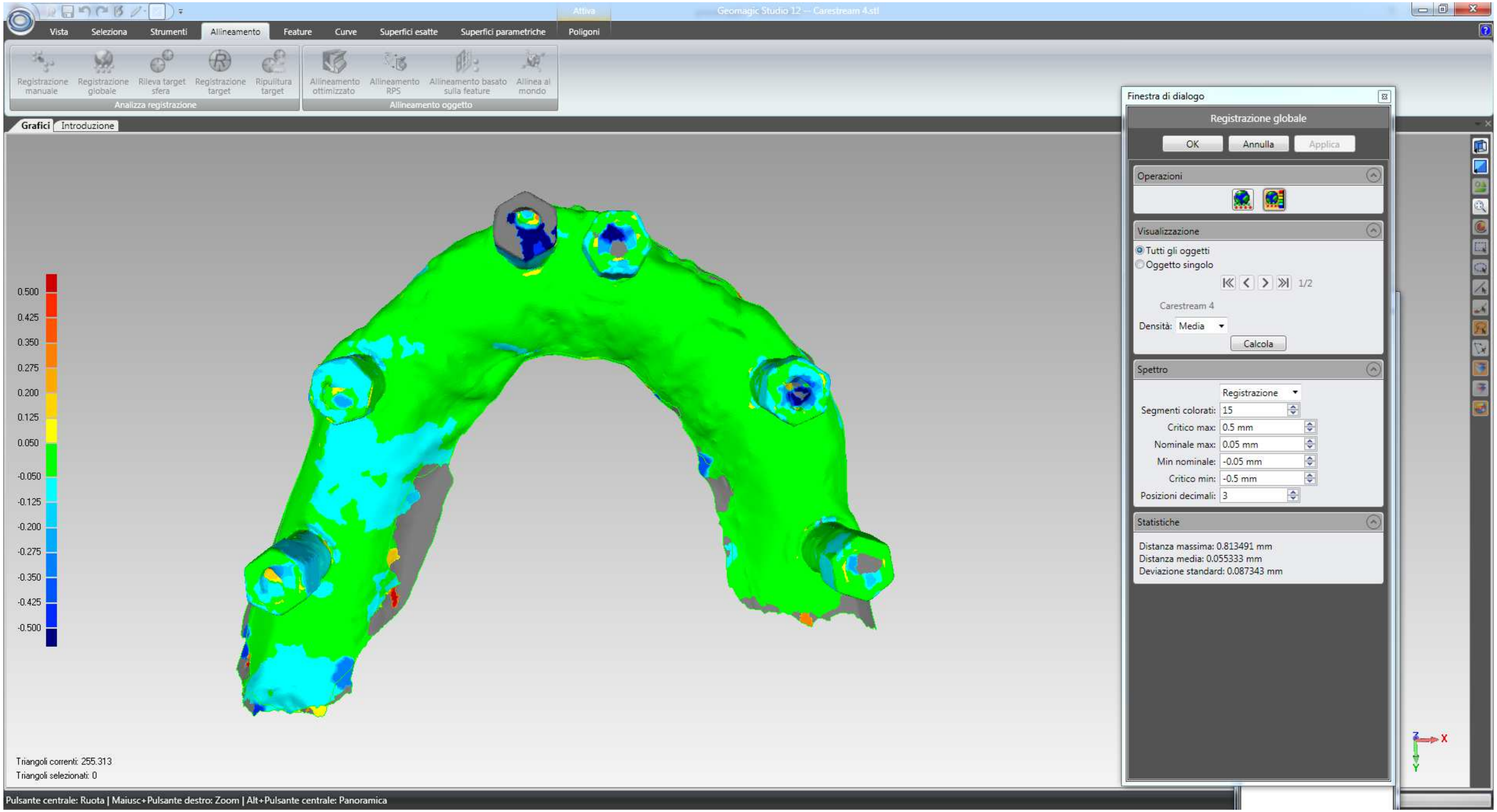

Care 4 as reference (best accuracy) vs Care 3

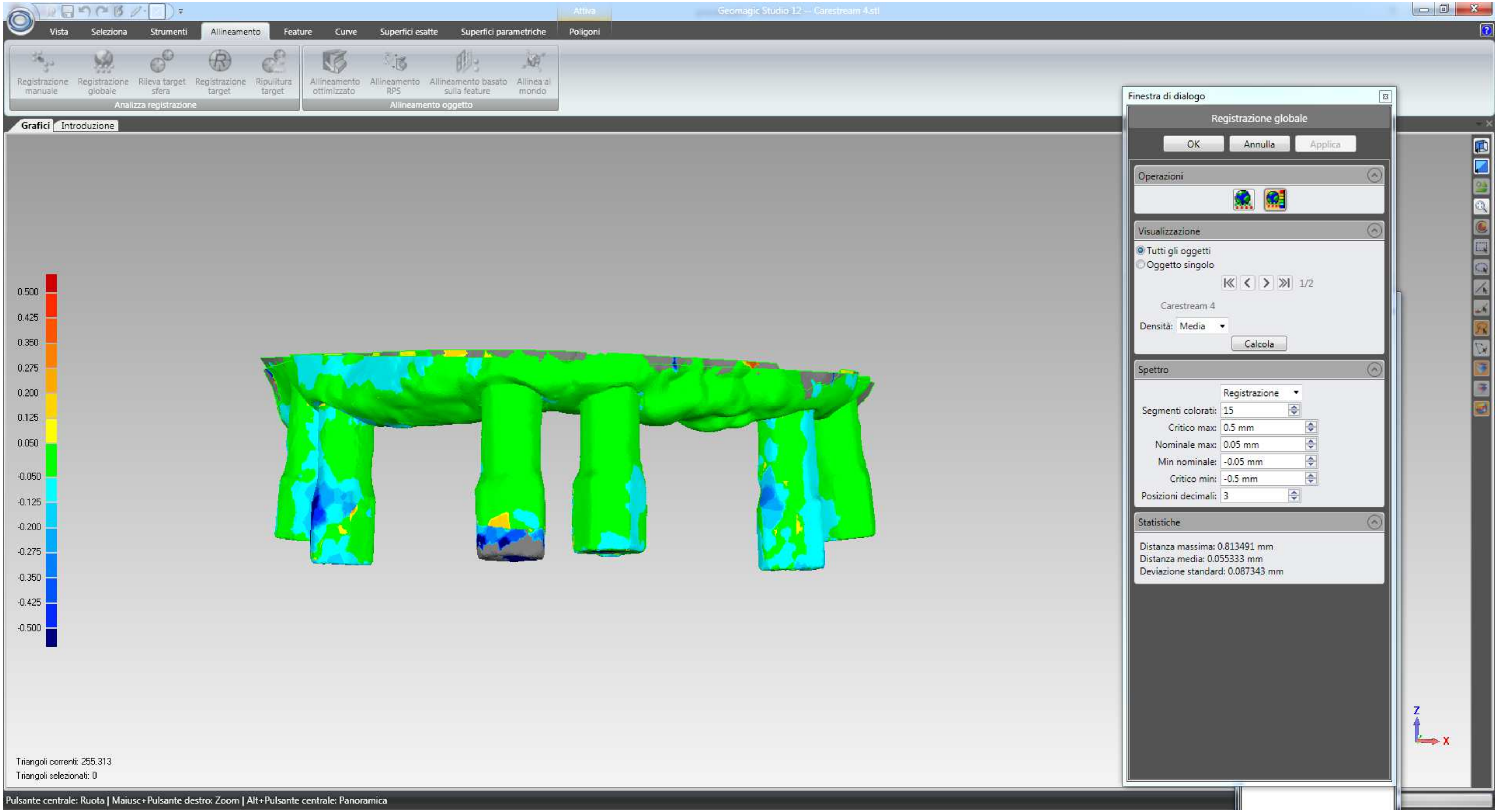

Care 4 as reference (best accuracy) vs Care 3

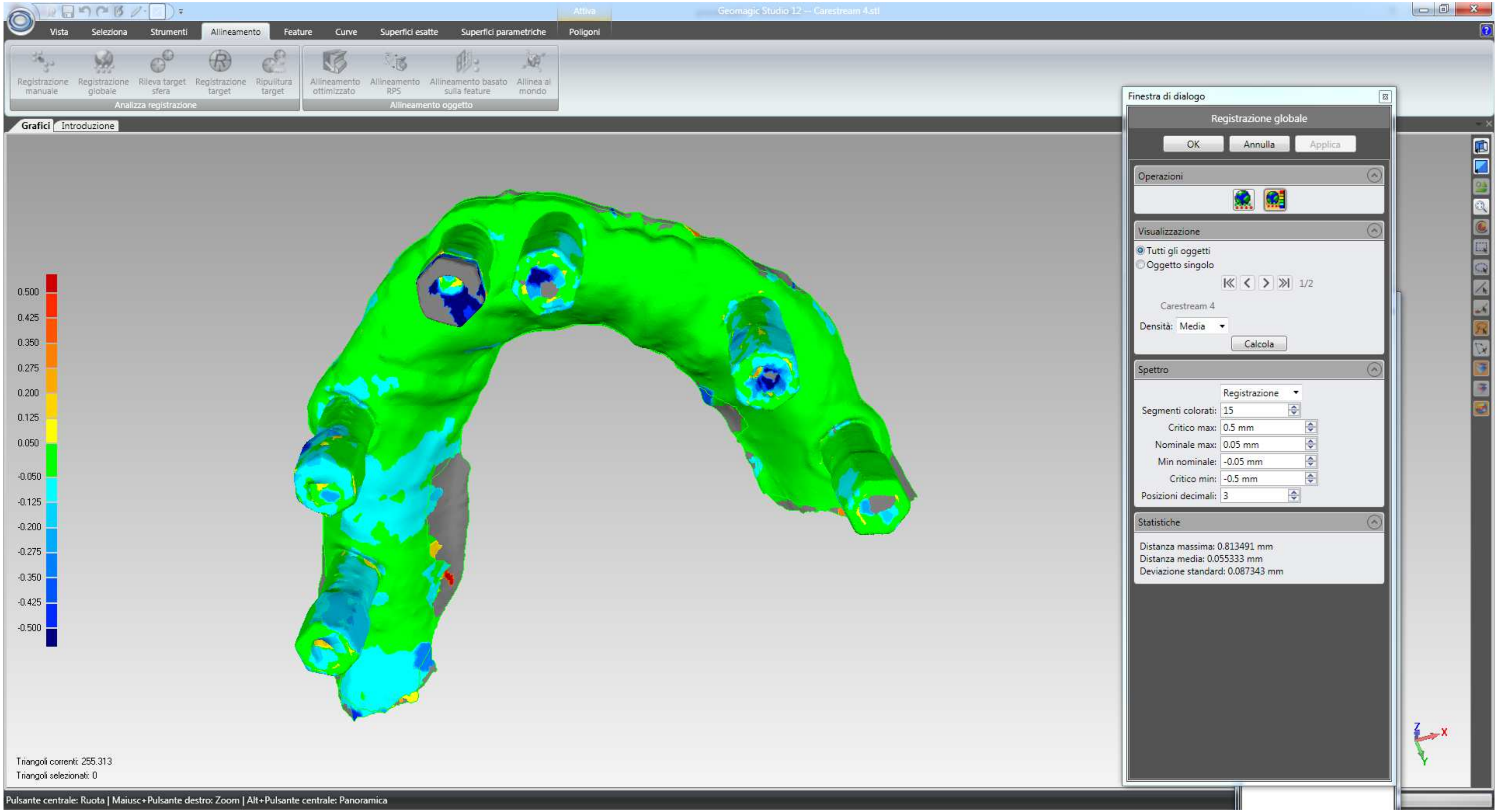

Care 4 as reference (best accuracy) vs Care 3

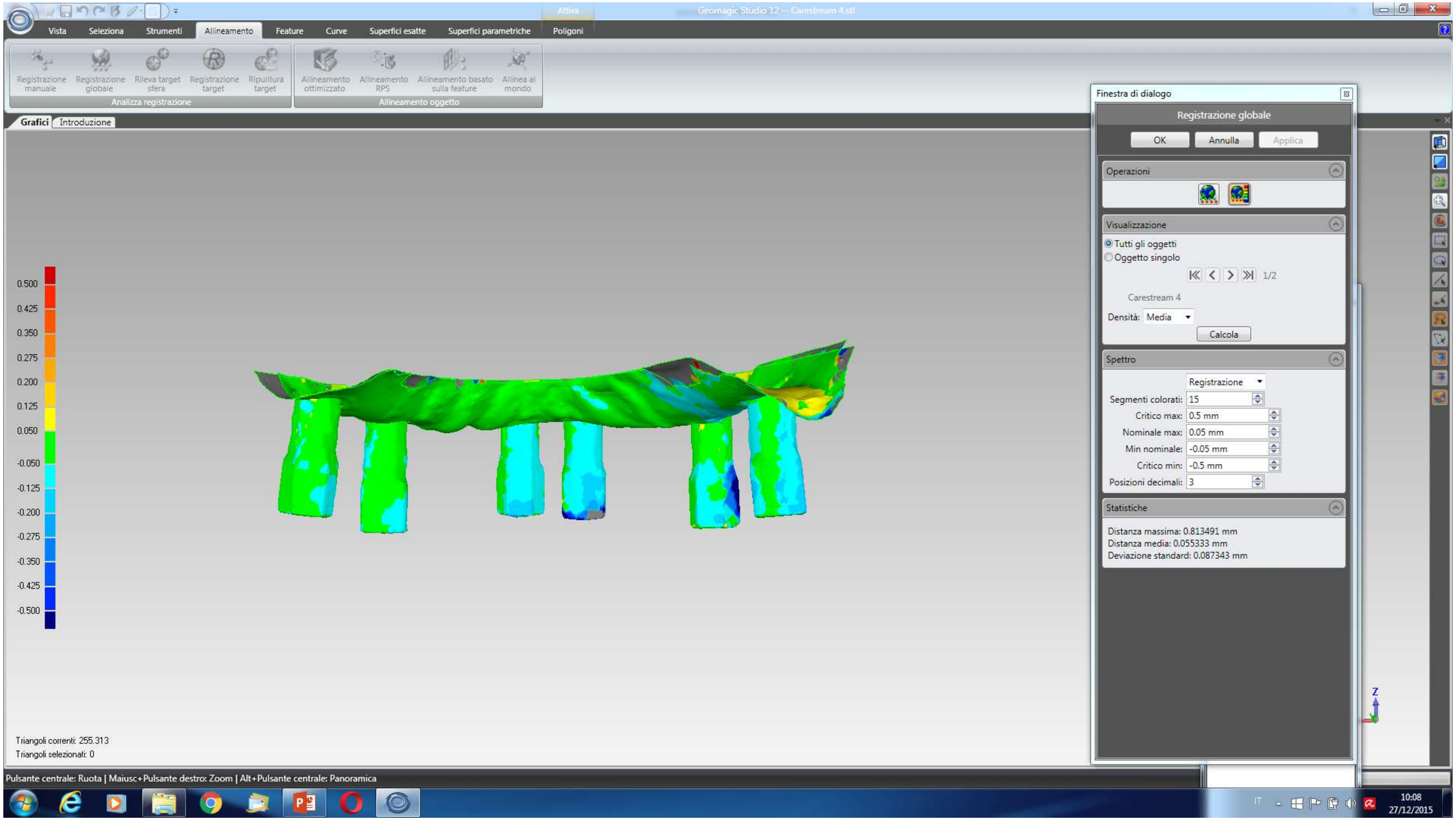

Care 4 as reference (best accuracy) vs Care 5

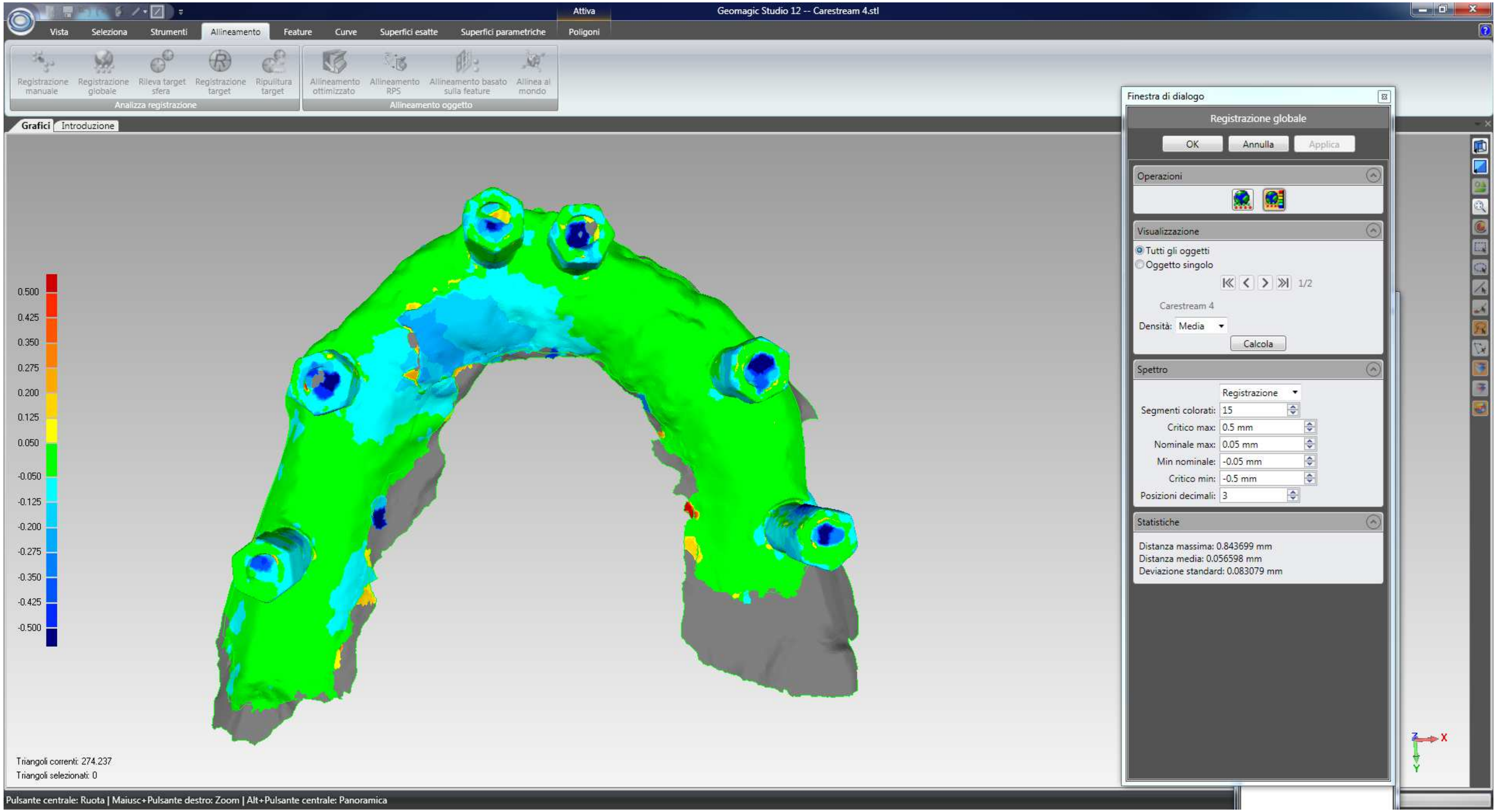

Care 4 as reference (best accuracy) vs Care 5

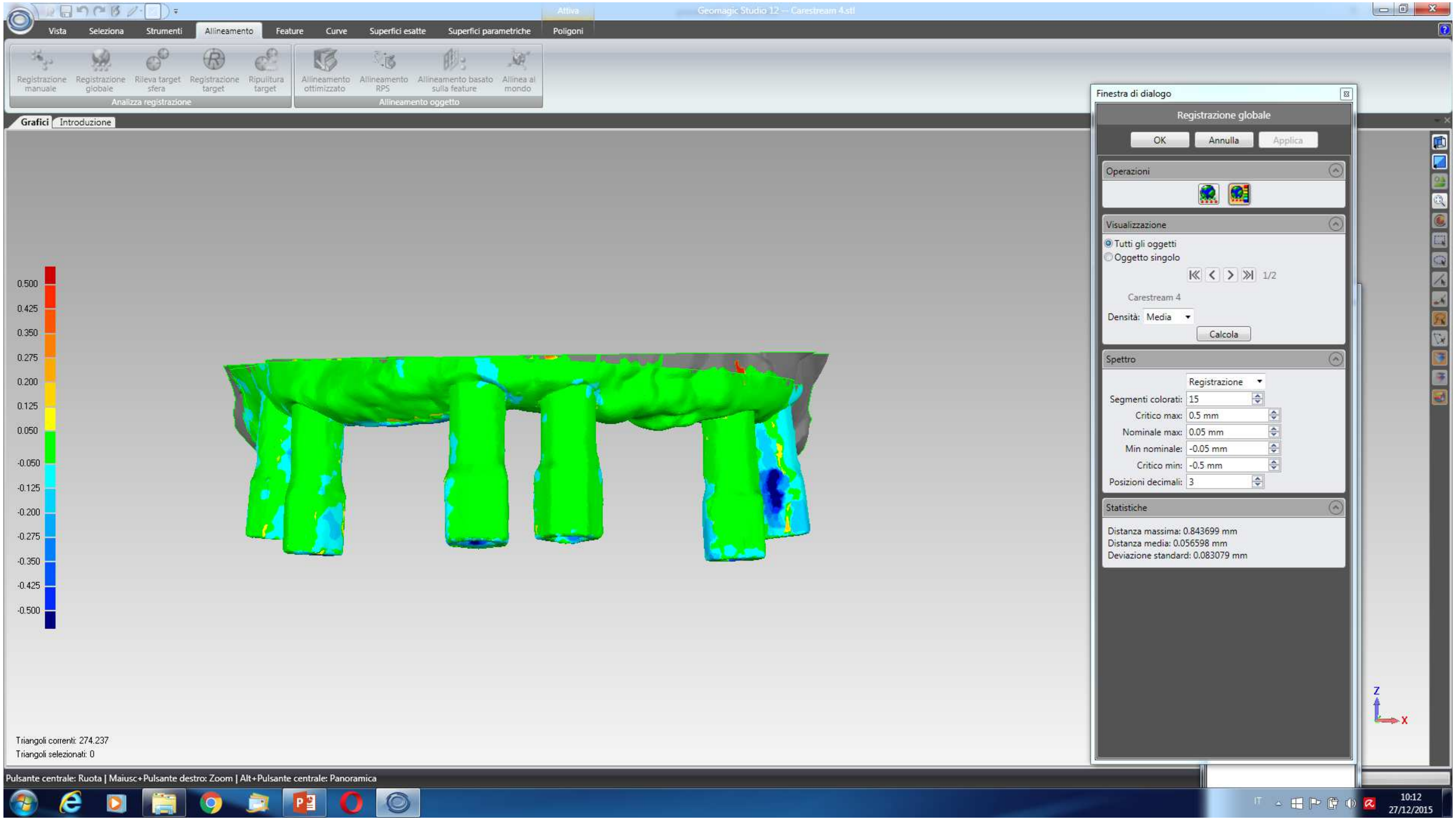

Care 4 as reference (best accuracy) vs Care 5

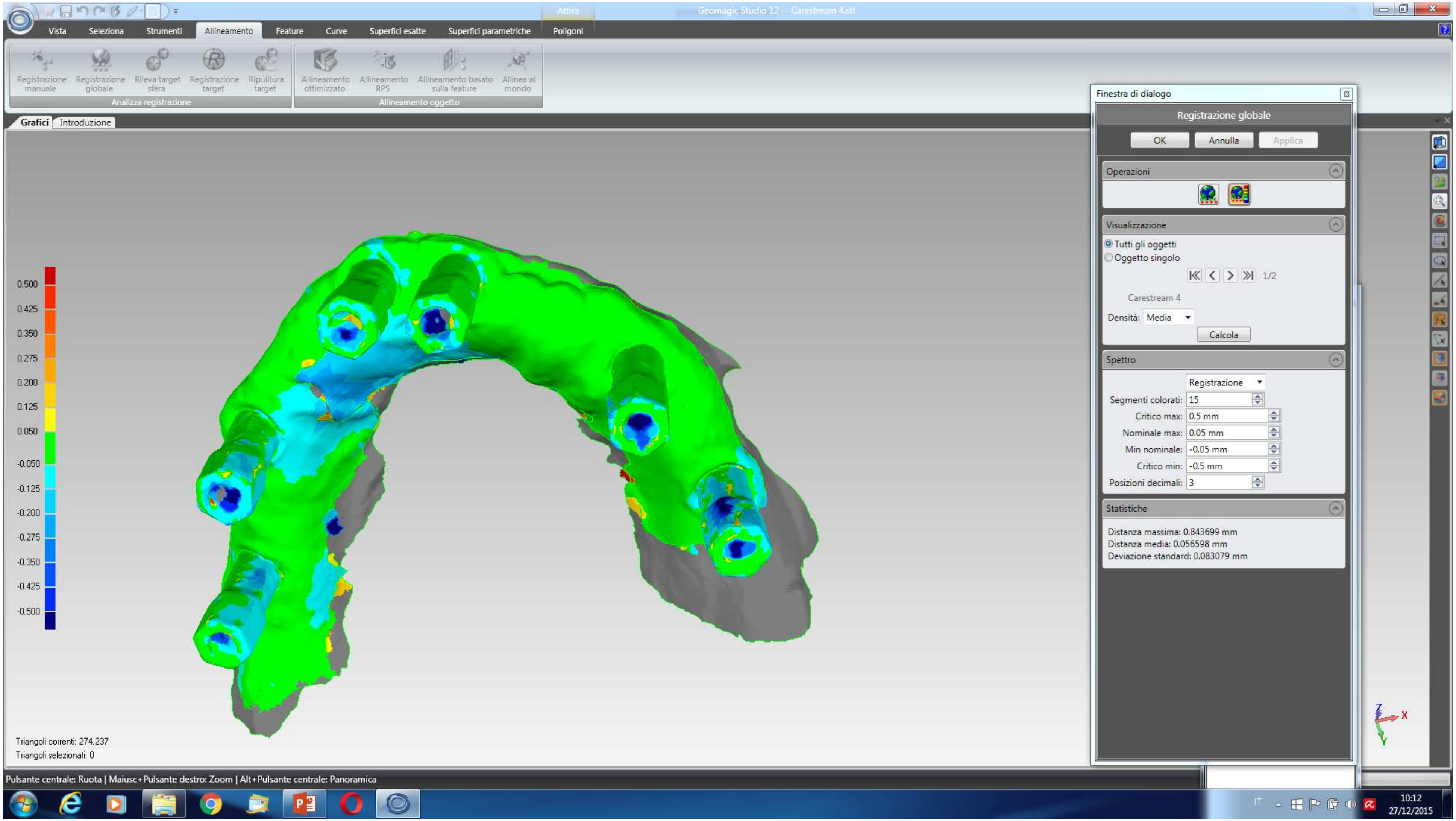

Care 4 as reference (best accuracy) vs Care 5

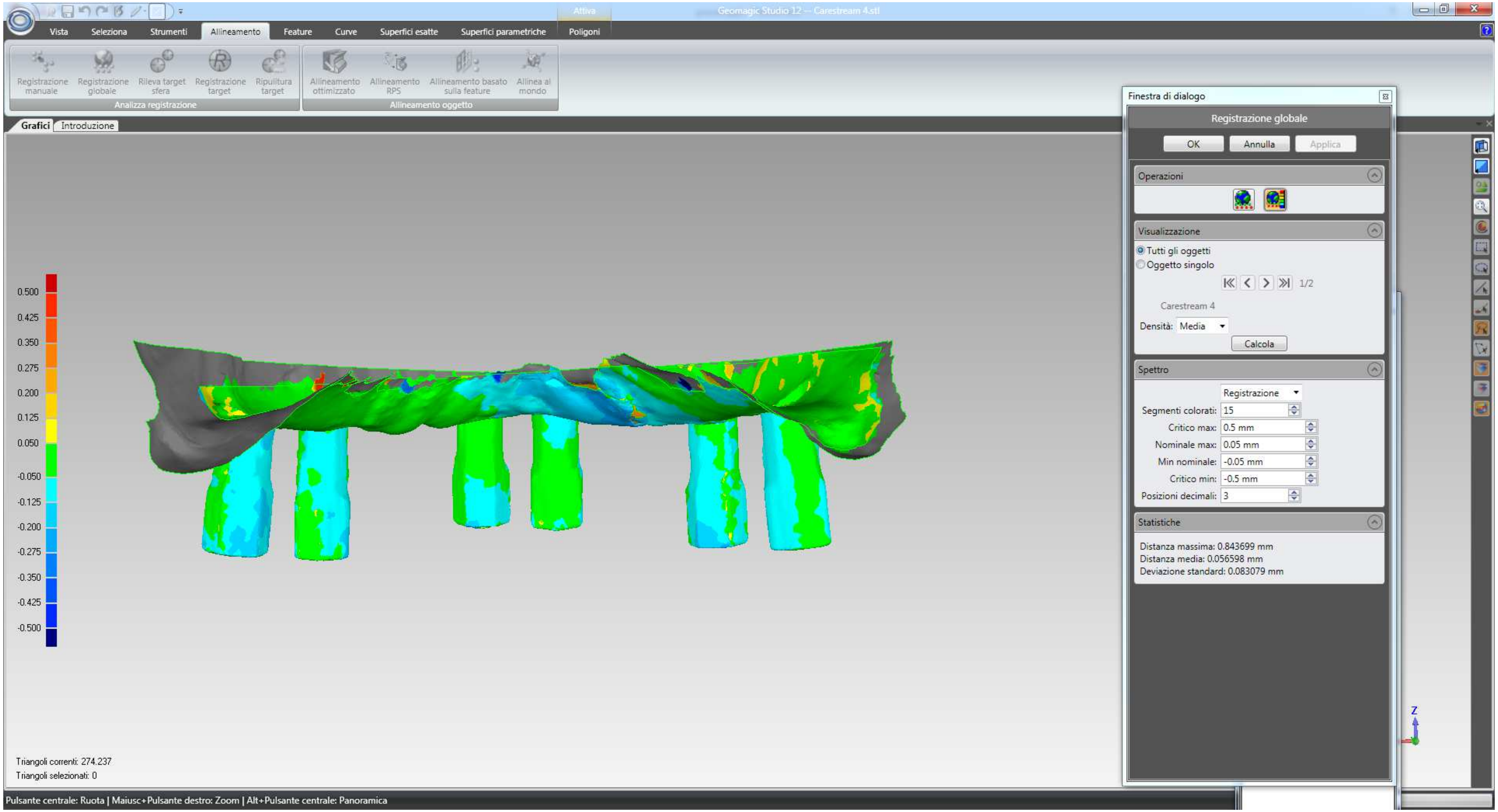

Care 2 vs Care 3

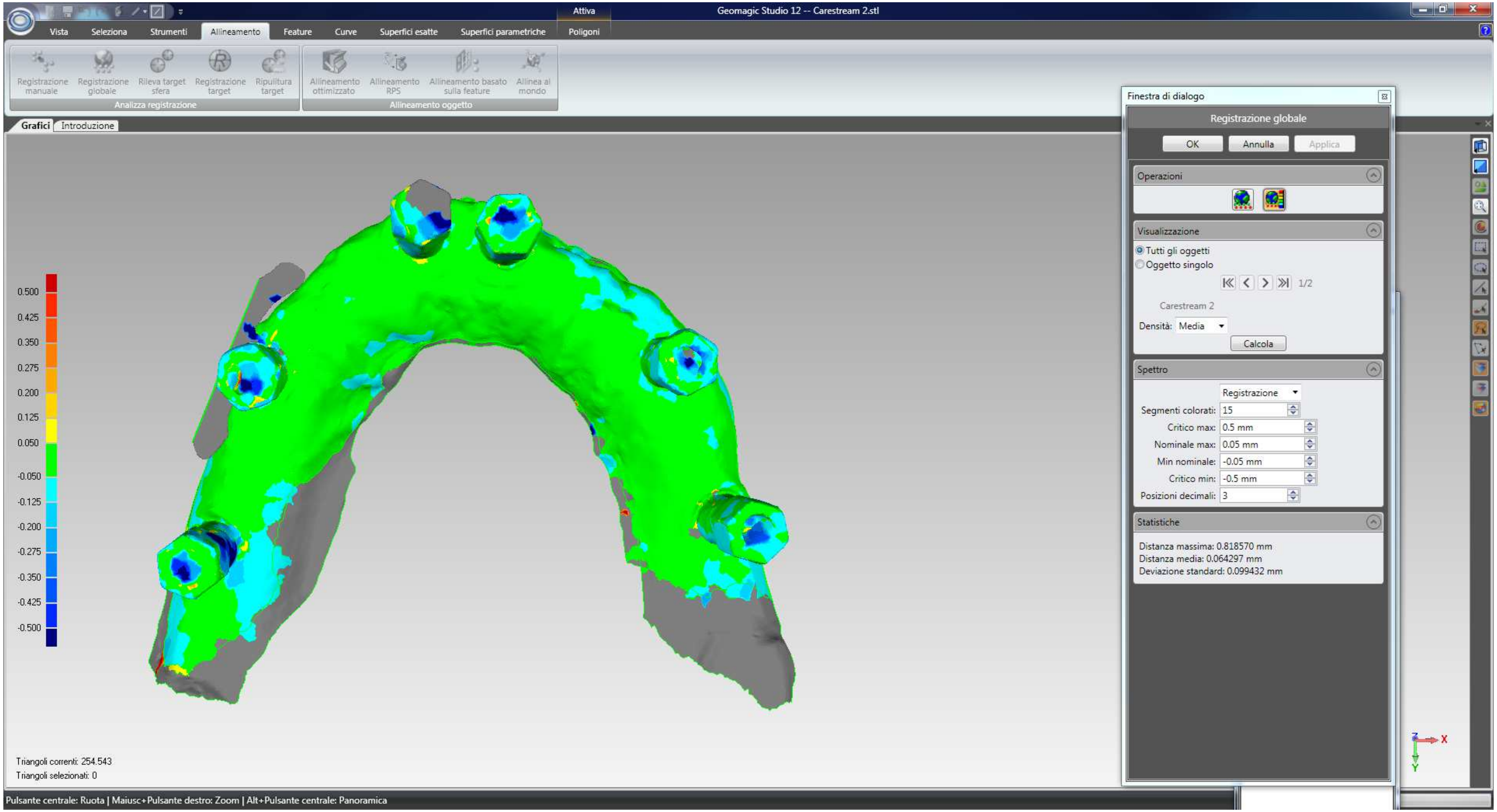

Care 2 vs Care 3

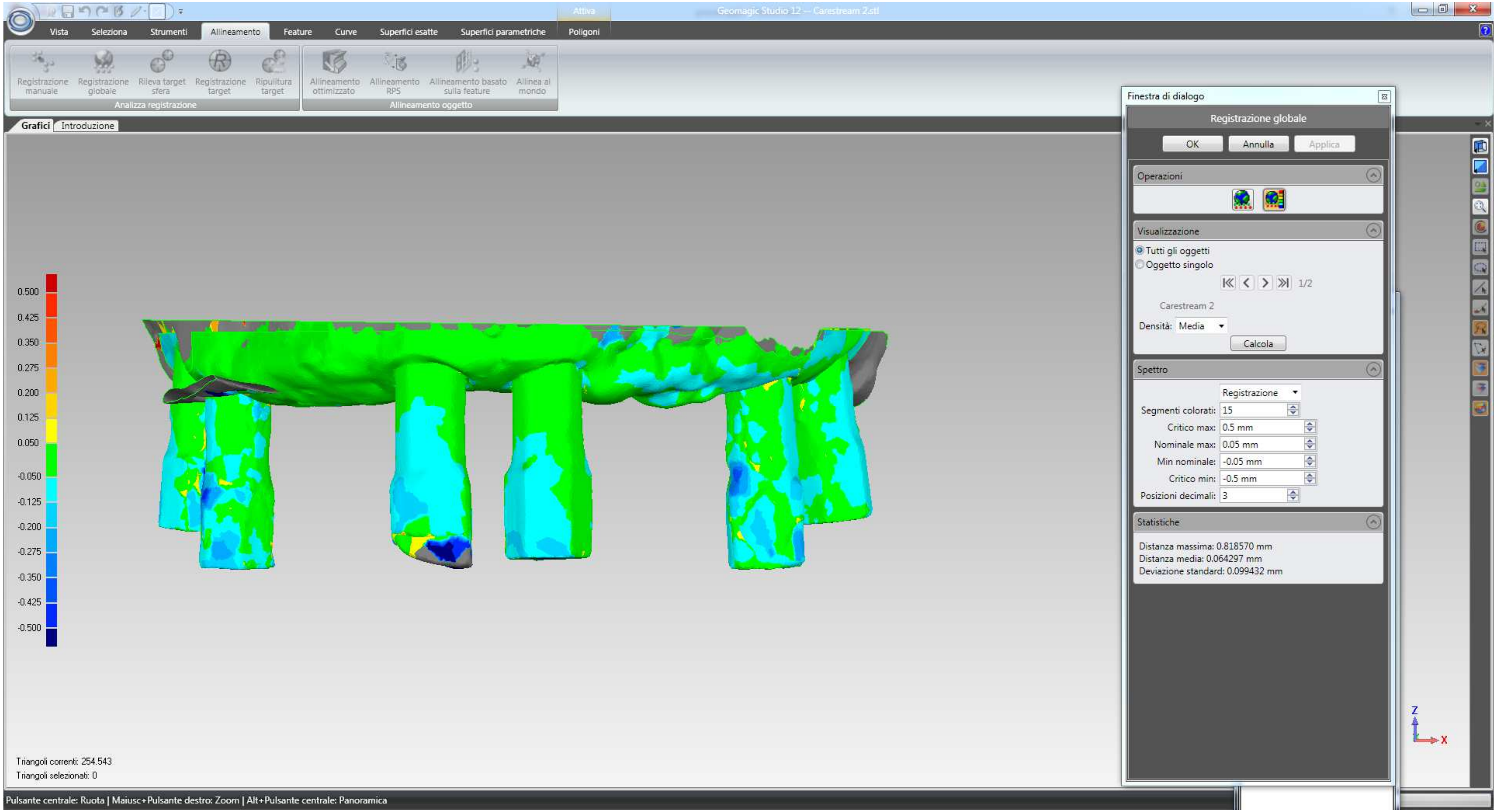

Care 2 vs Care 3

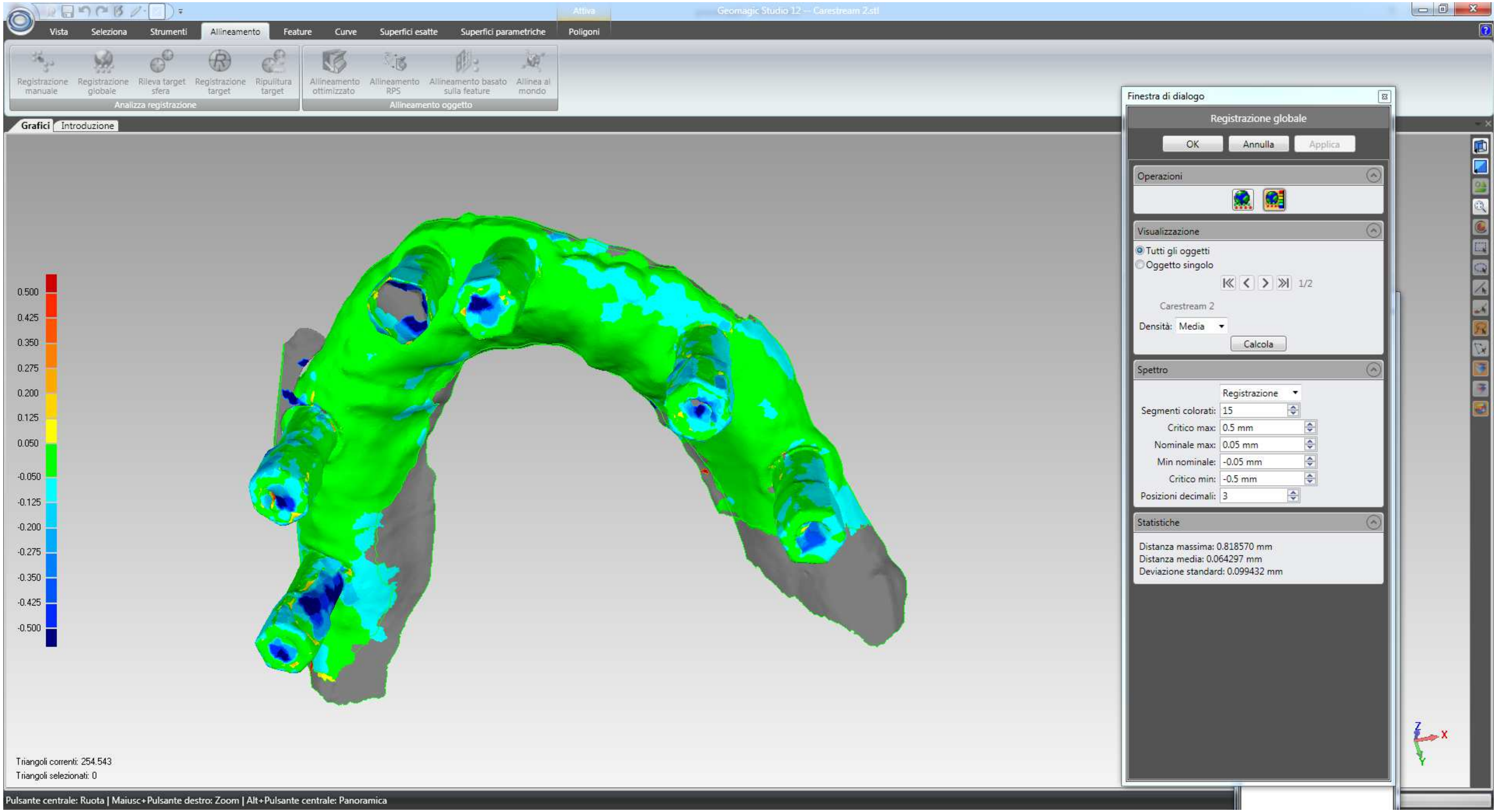

Care 2 vs Care 3

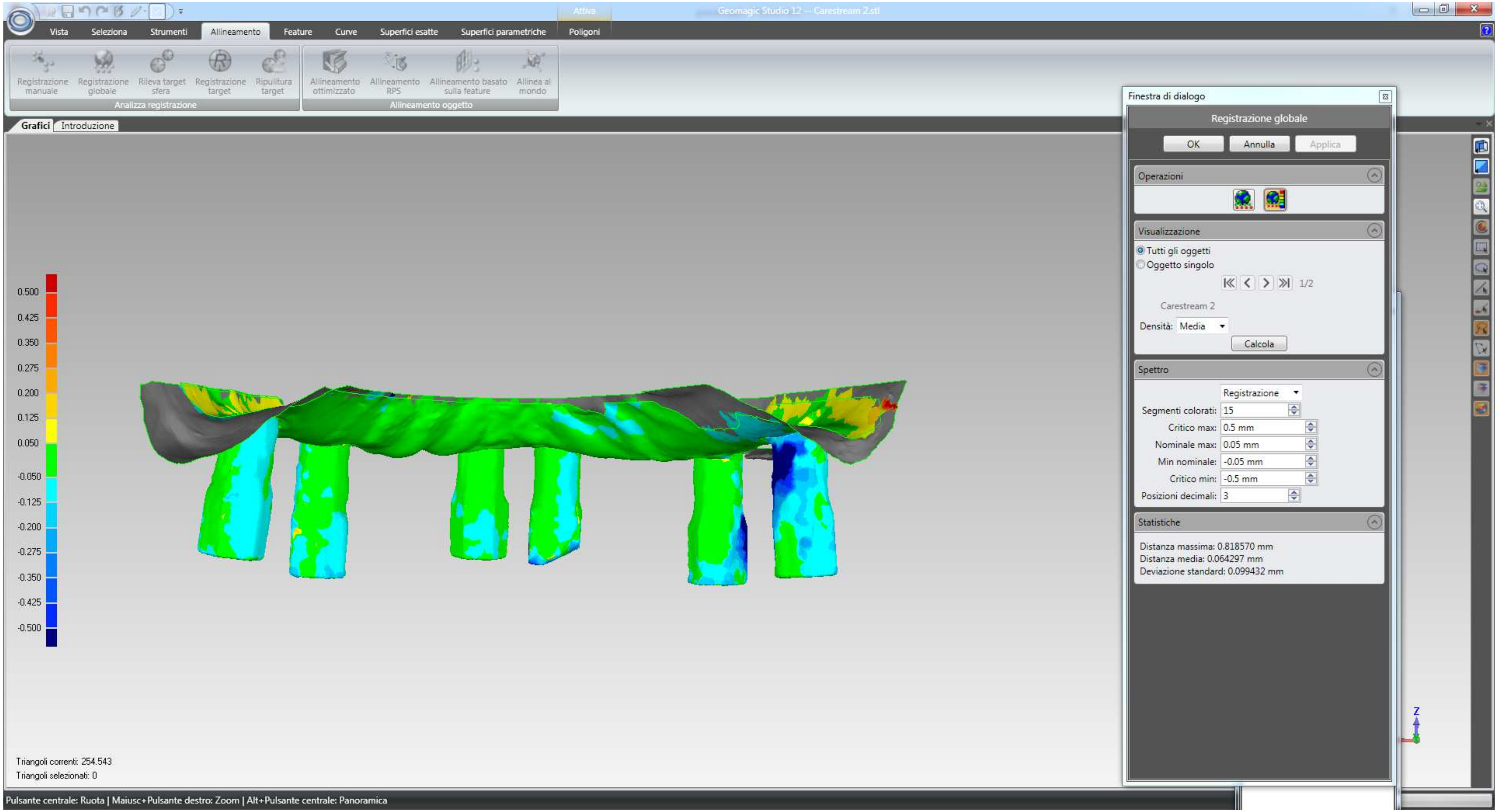

General precision of Carestream scanner in the totally edentulous

	Mean distance	SD	Maximum distance
Care 4 vs Care 1	0.038	0.067	0.804
Care 4 vs Care 2	0.063	0.104	0.808
Care 4 vs Care 3	0.055	0.087	0.813
Care 4 vs Care 5	0.056	0.083	0.843
Care 2 vs Care 3	0.064	0.099	0.818

Overall Care general precision: 0.055 (0.010)

Plane 5 vs Plane 1

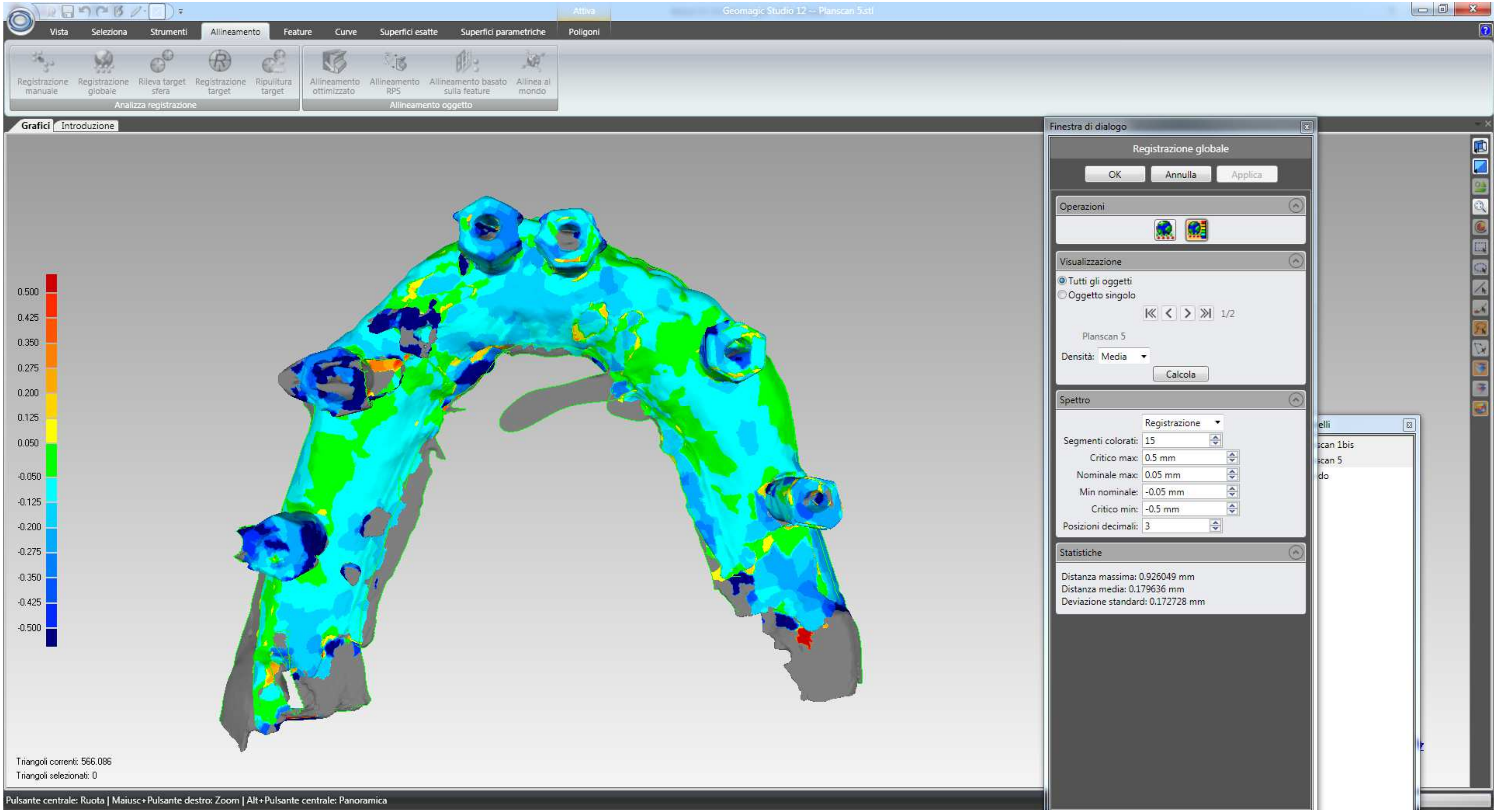

Plane 5 vs Plane 1

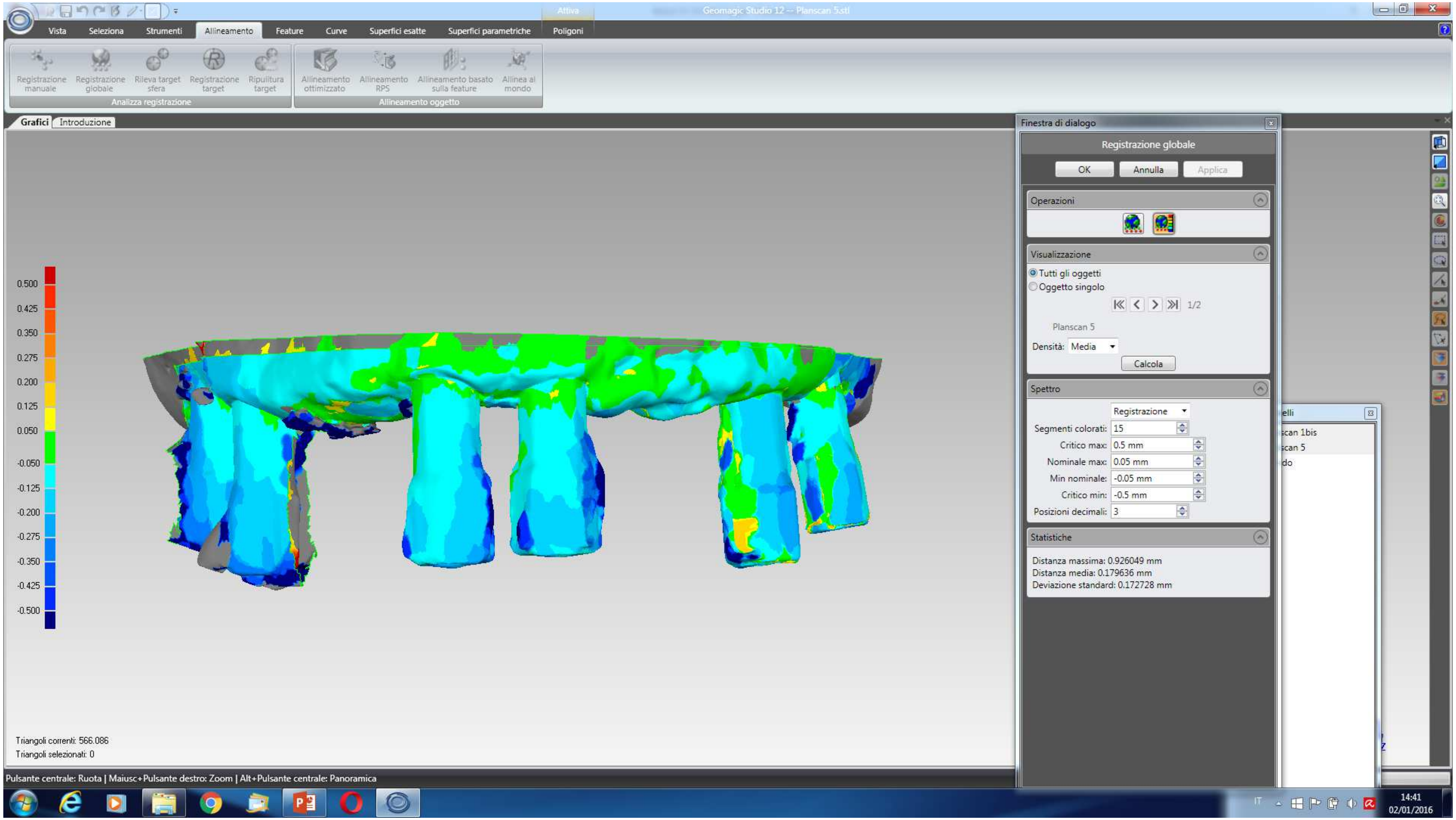

Plane 5 vs Plane 1

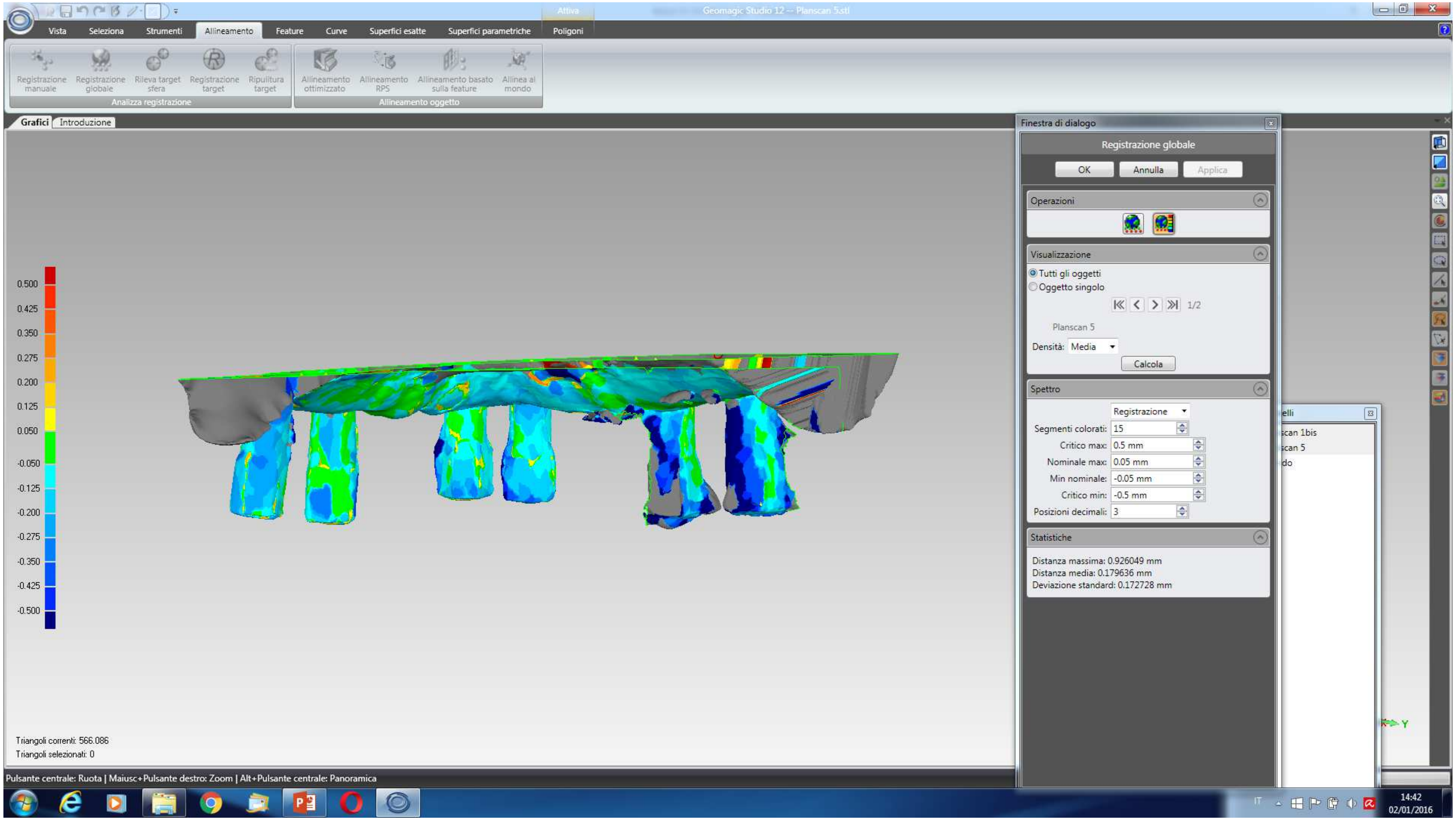

Plane 5 vs Plane 1

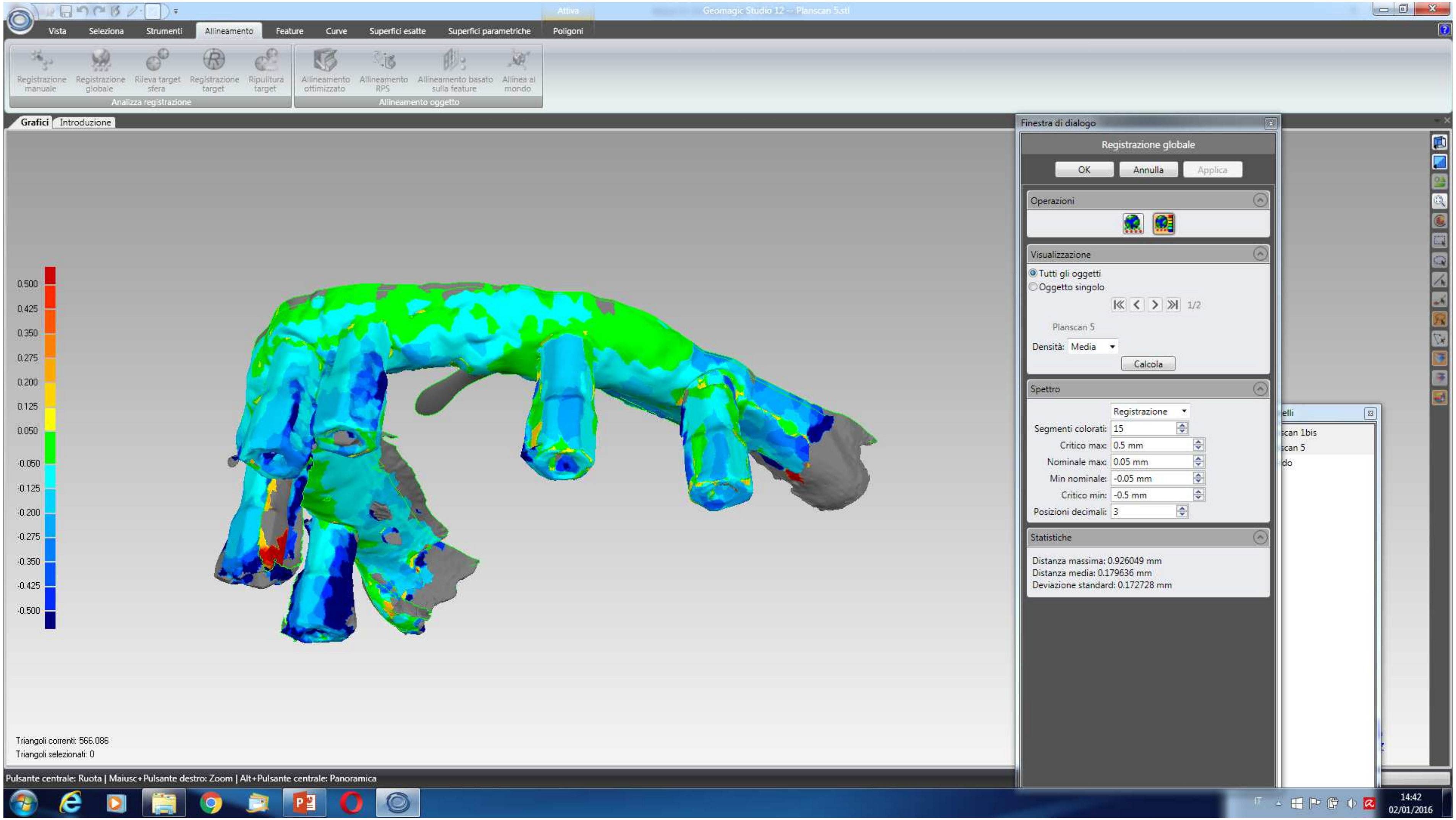

Plane 5 vs Plane 2

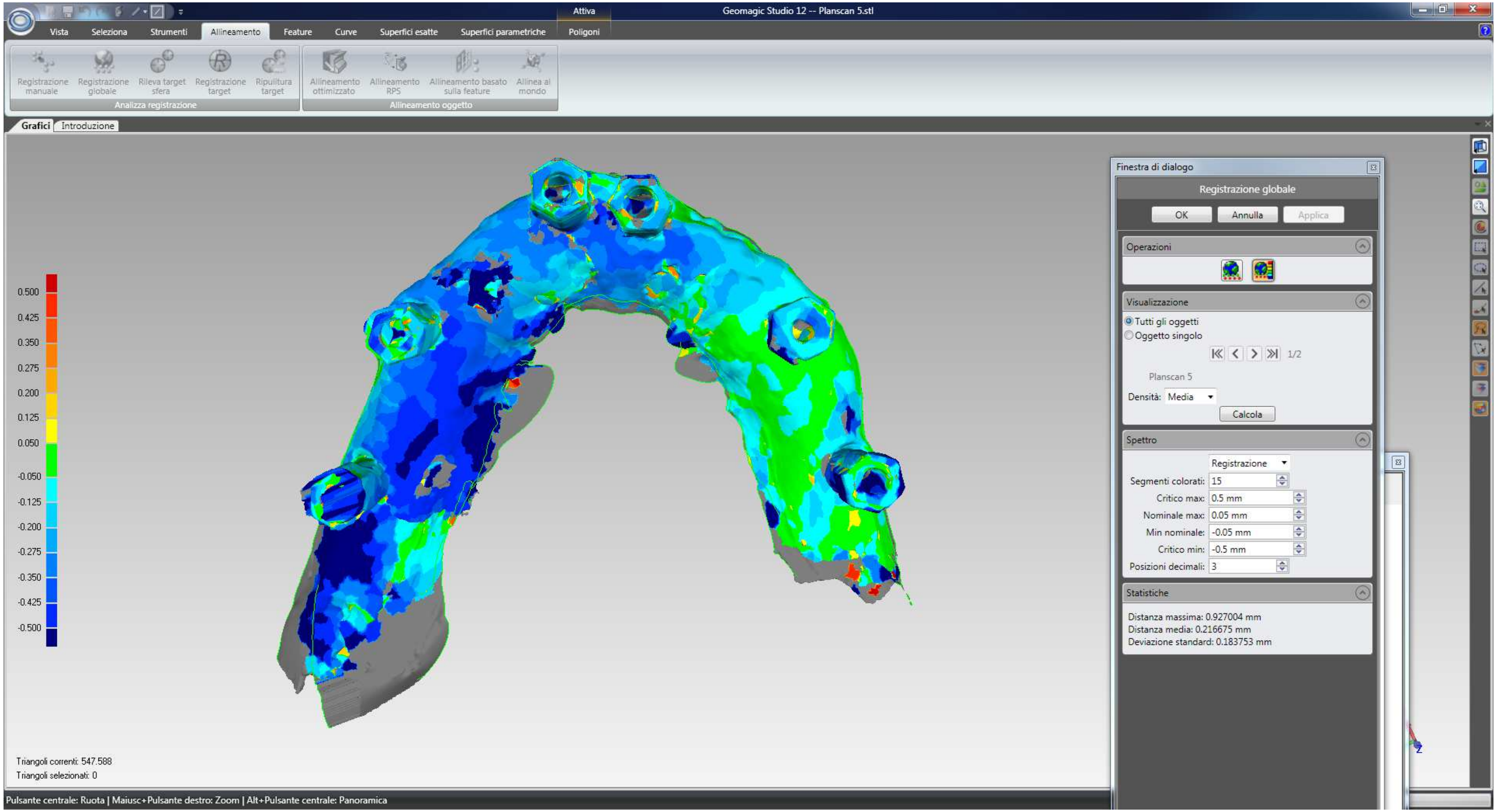

Plane 5 vs Plane 2

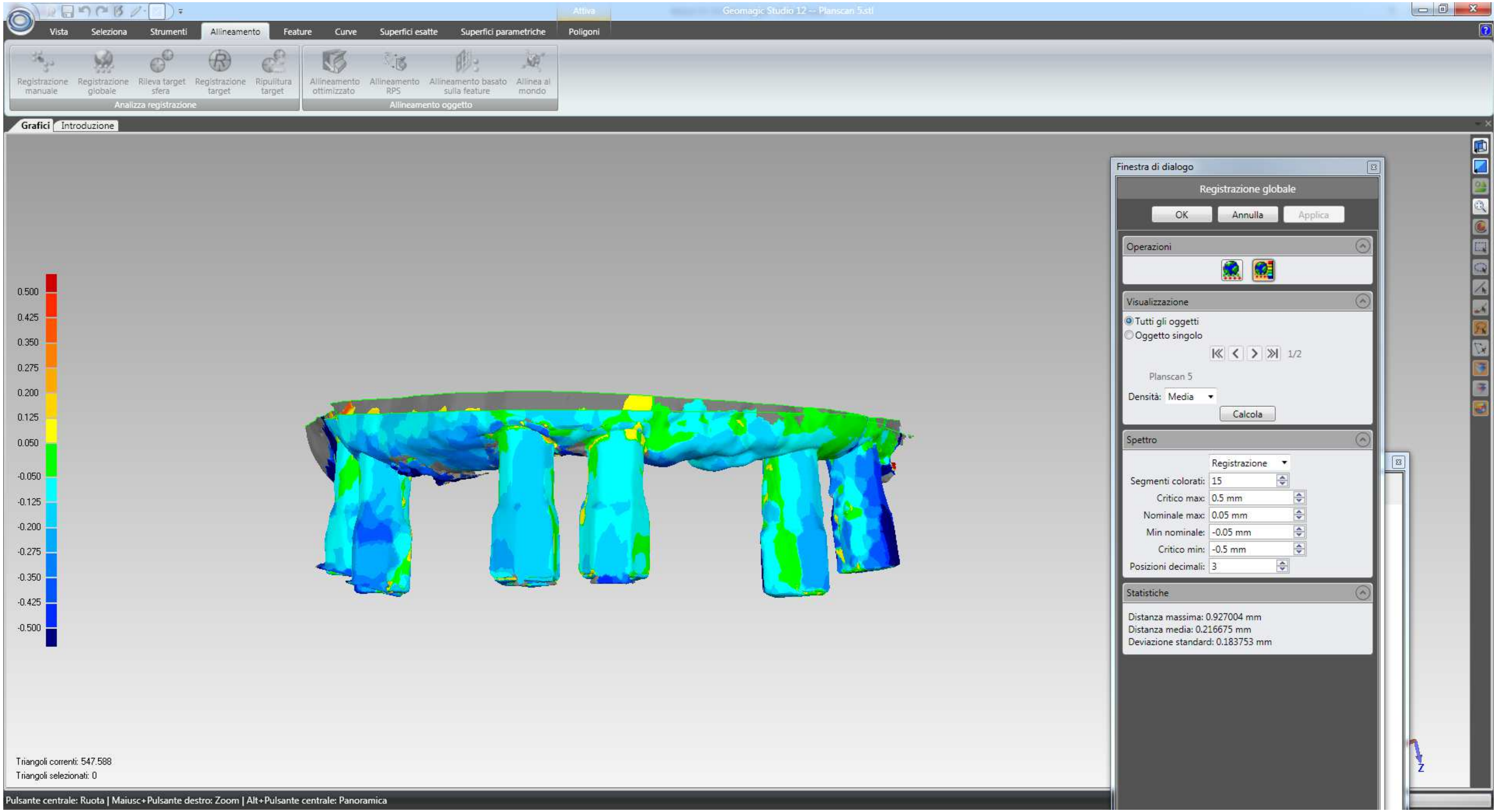

Plane 5 vs Plane 2

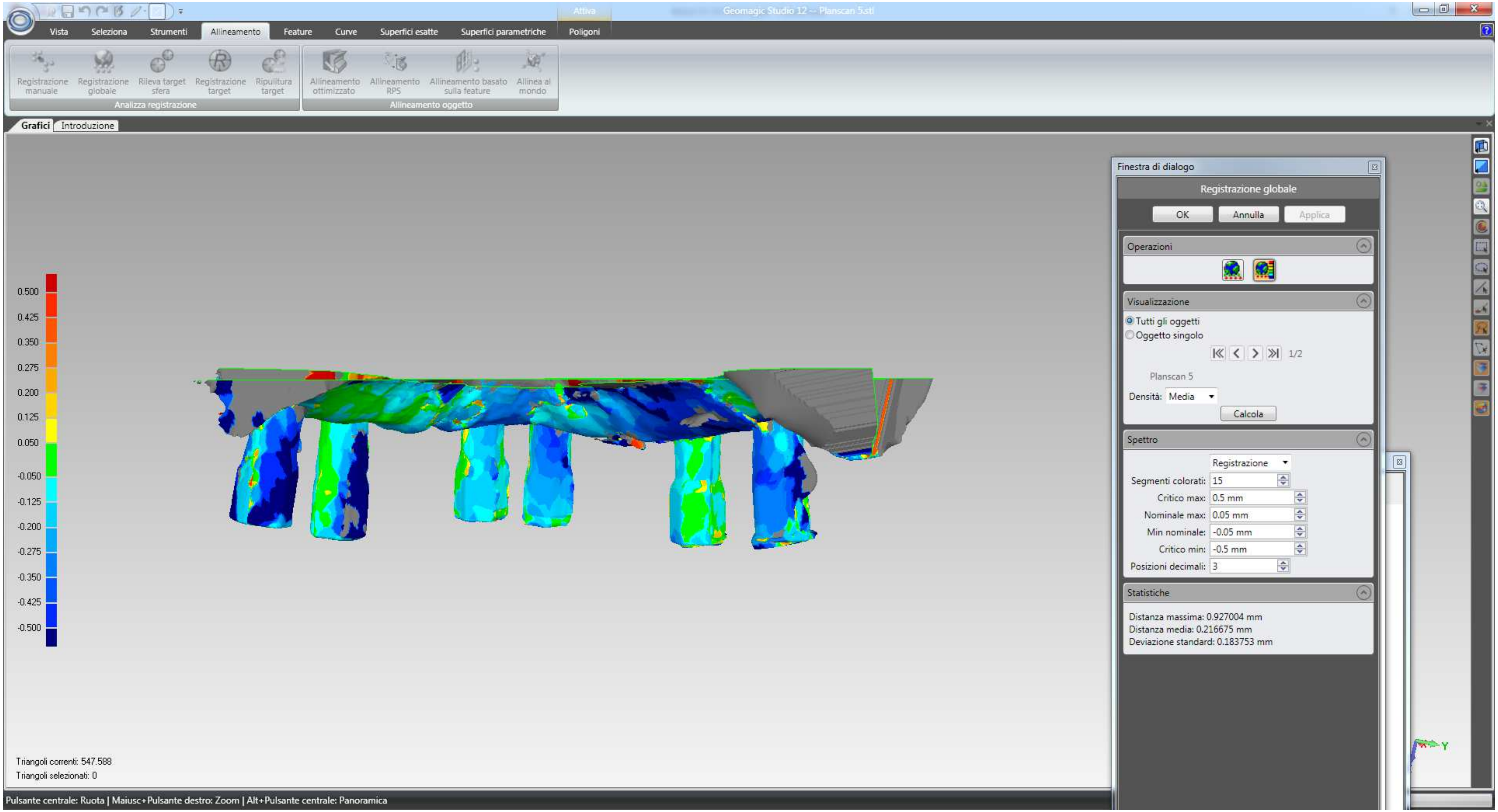

Plane 5 vs Plane 2

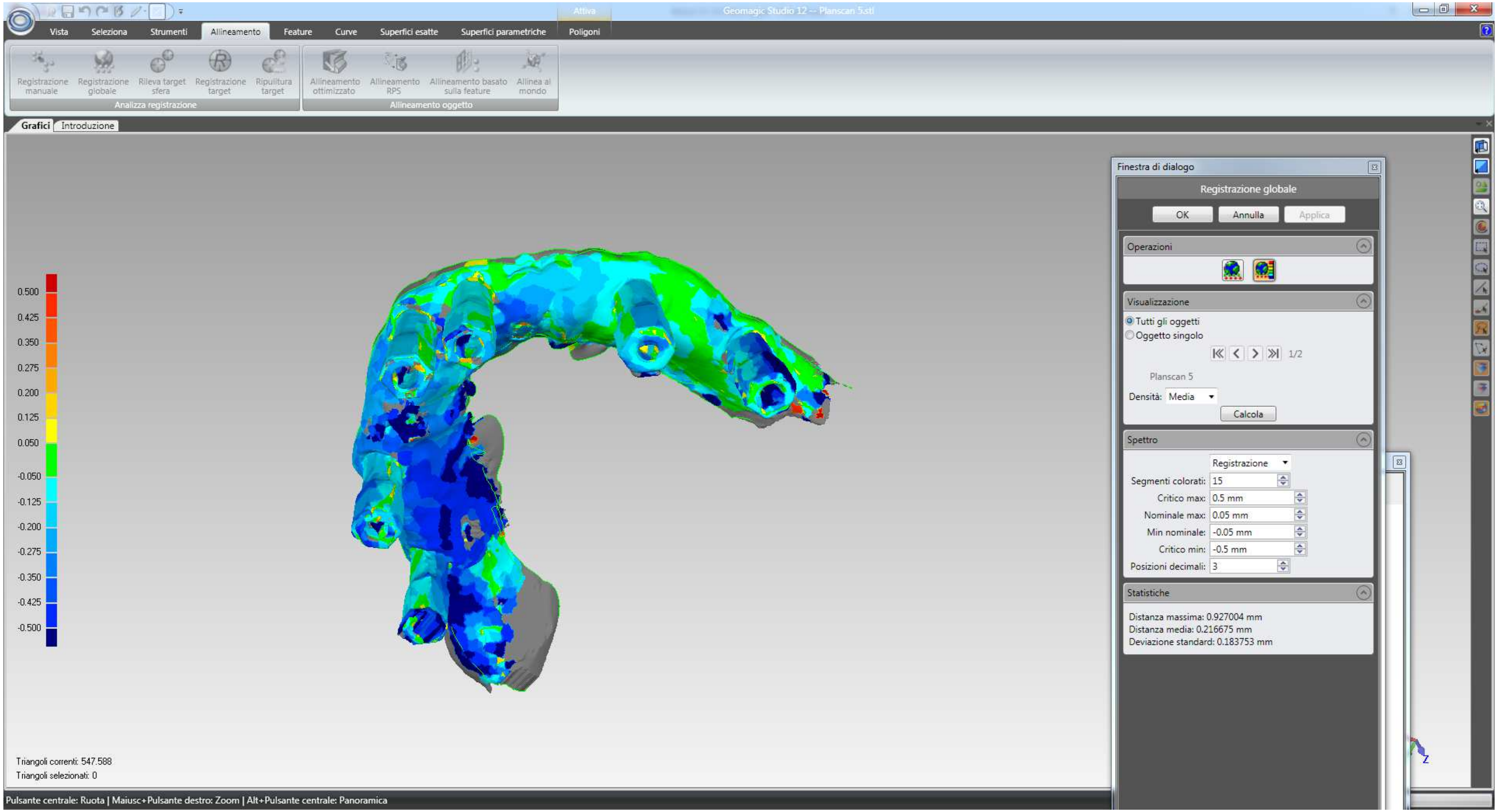

Plane 5 vs Plane 3

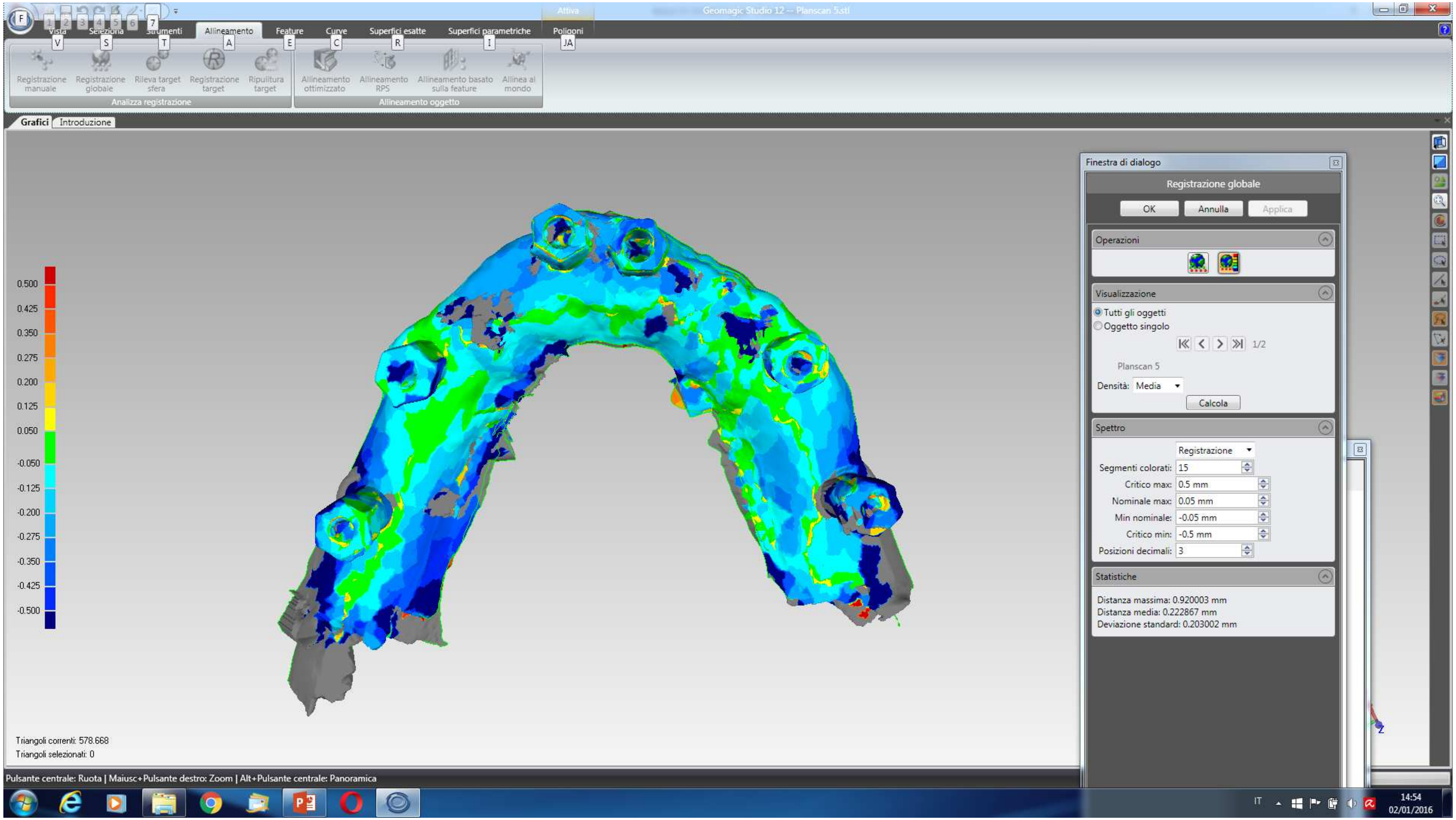

Plane 5 vs Plane 3

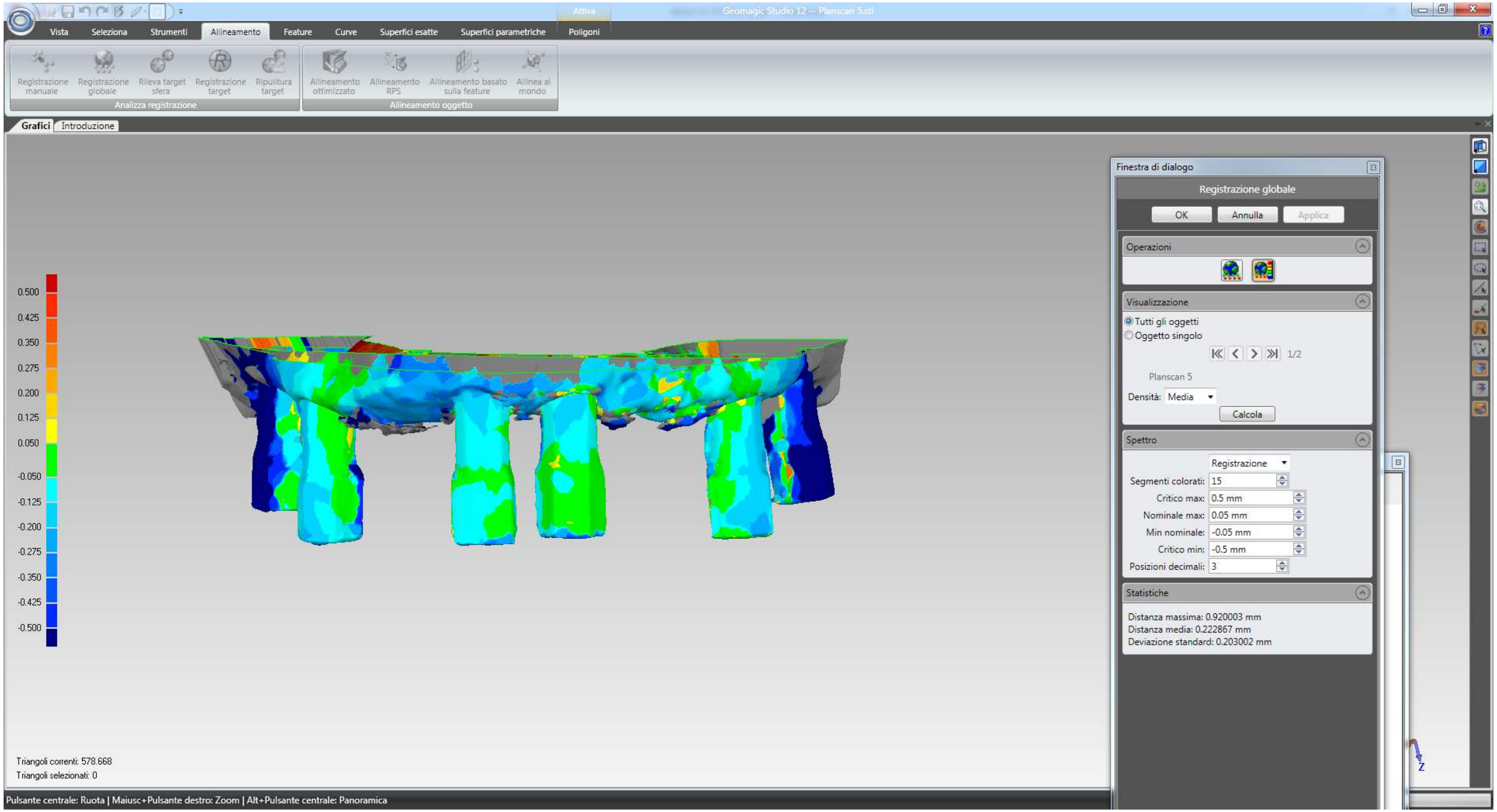

Plane 5 vs Plane 3

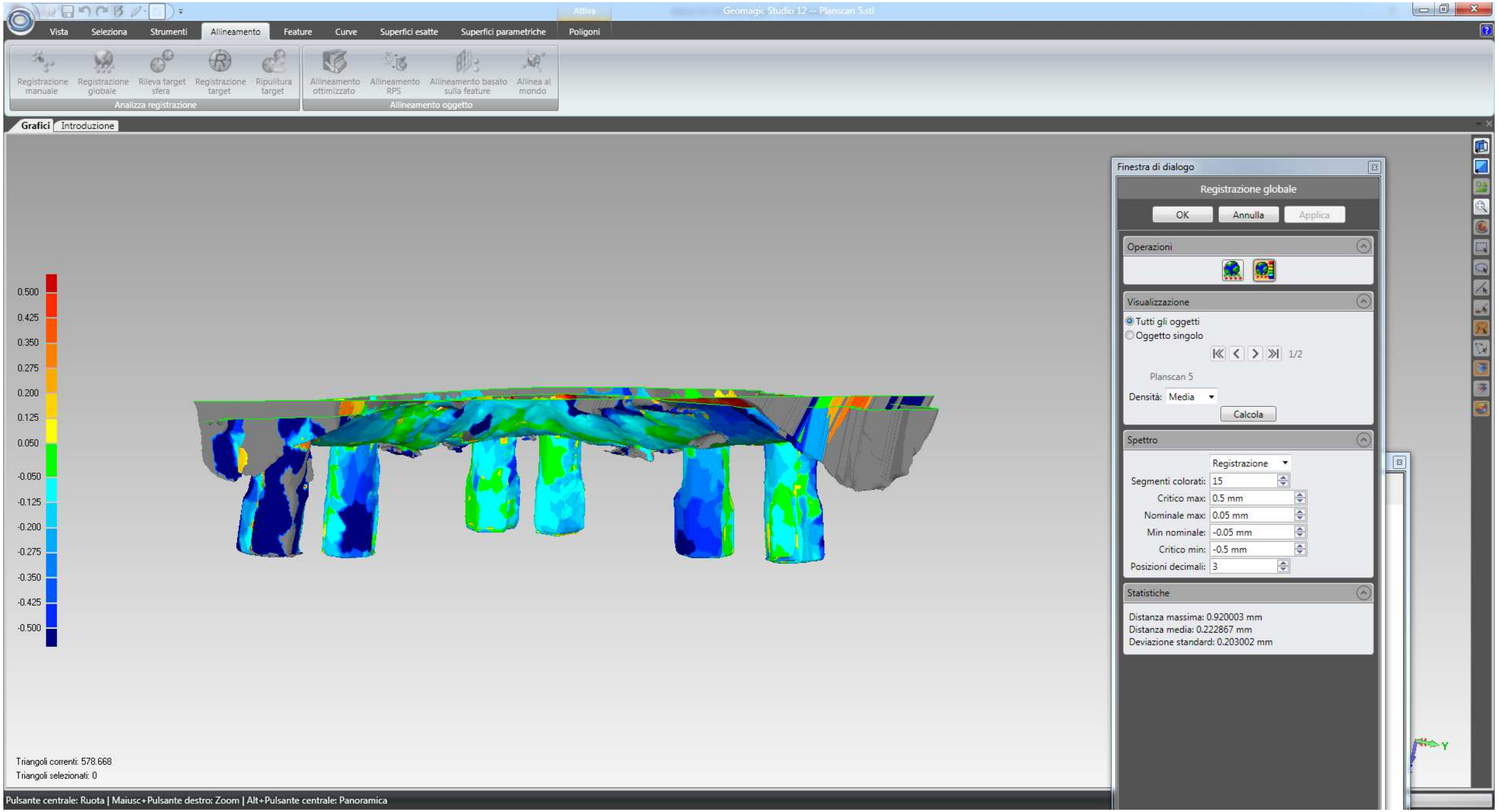

Plane 5 vs Plane 3

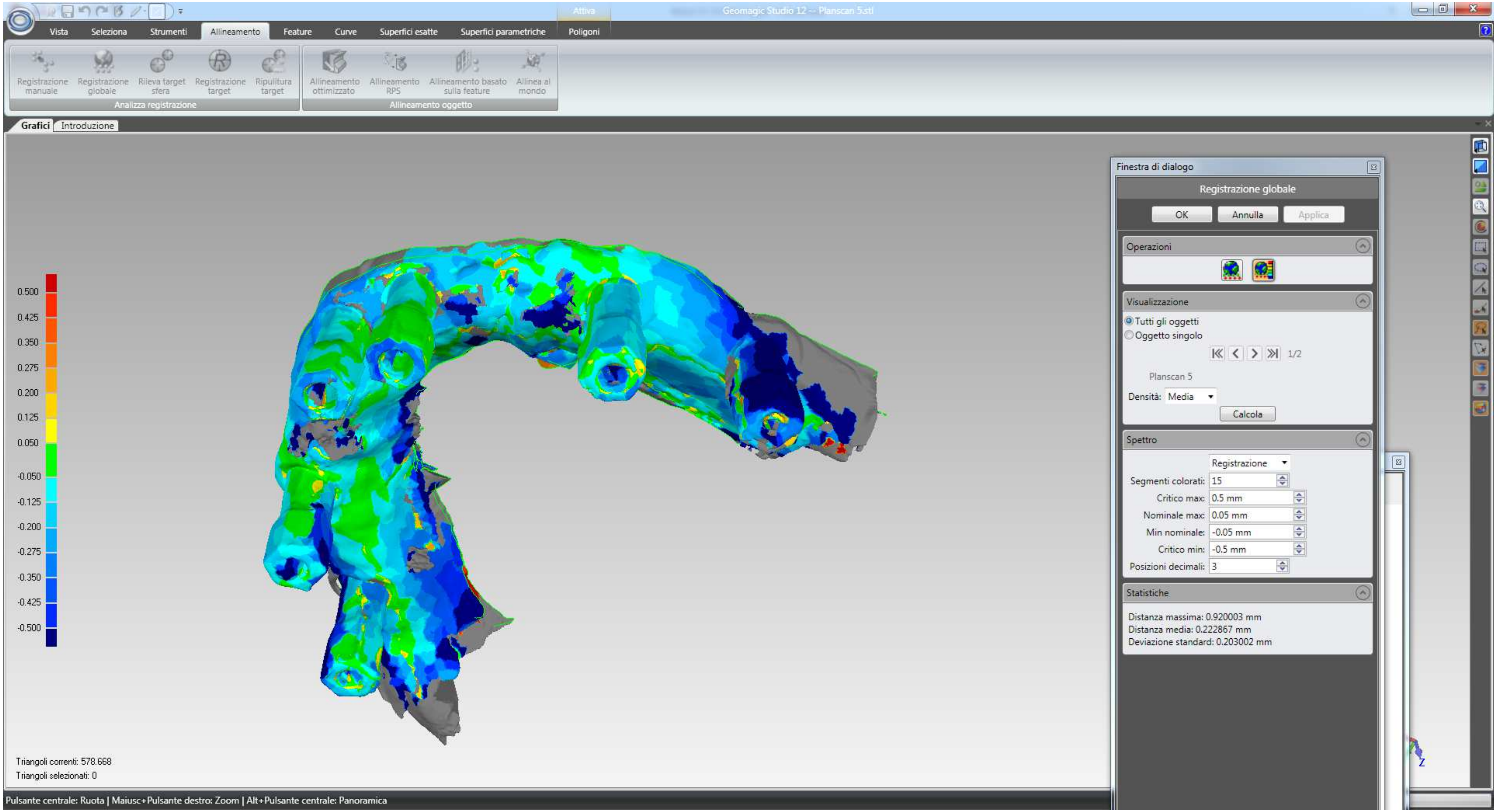

Plane 5 vs Plane 4

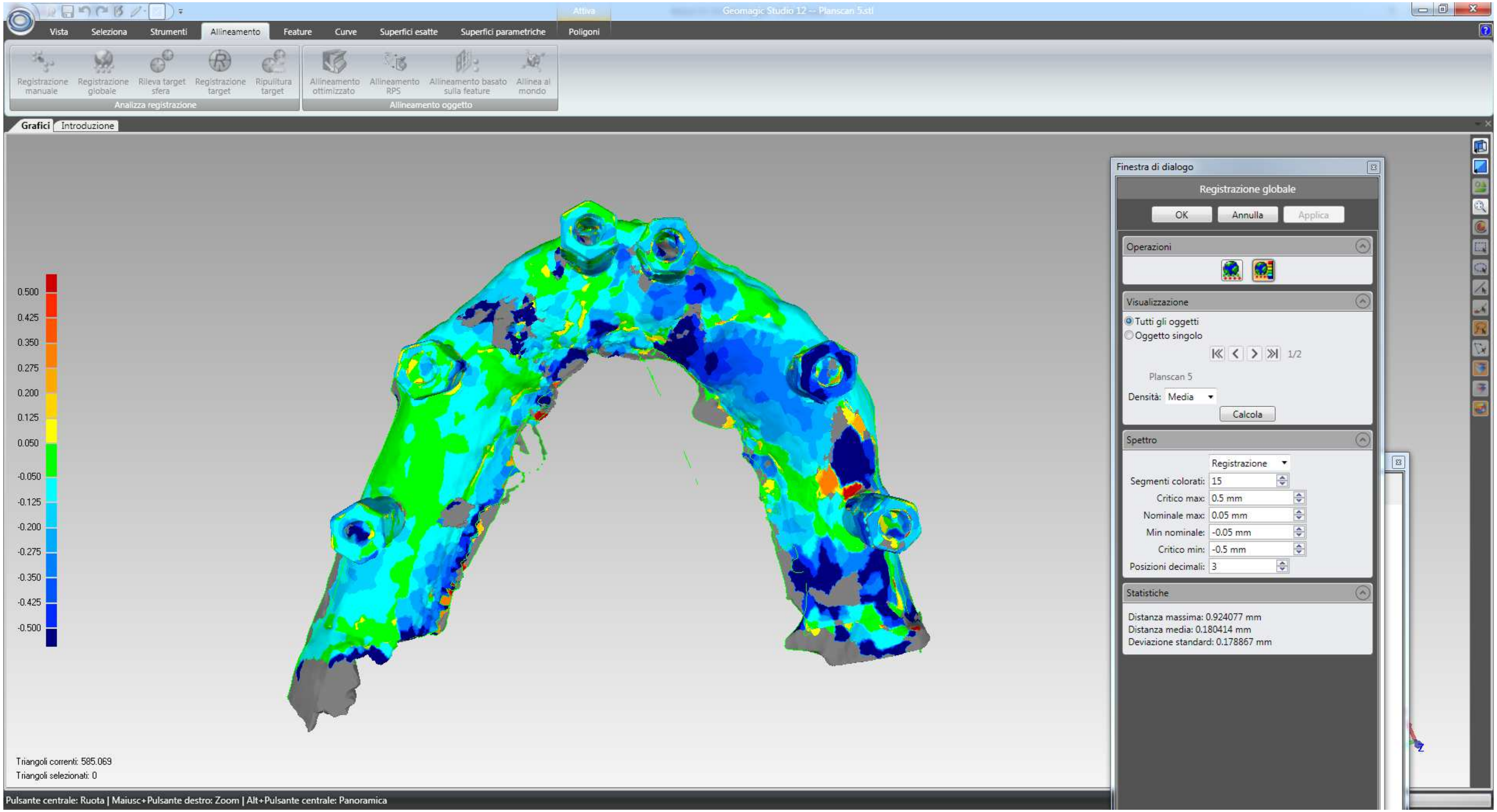

Plane 5 vs Plane 4

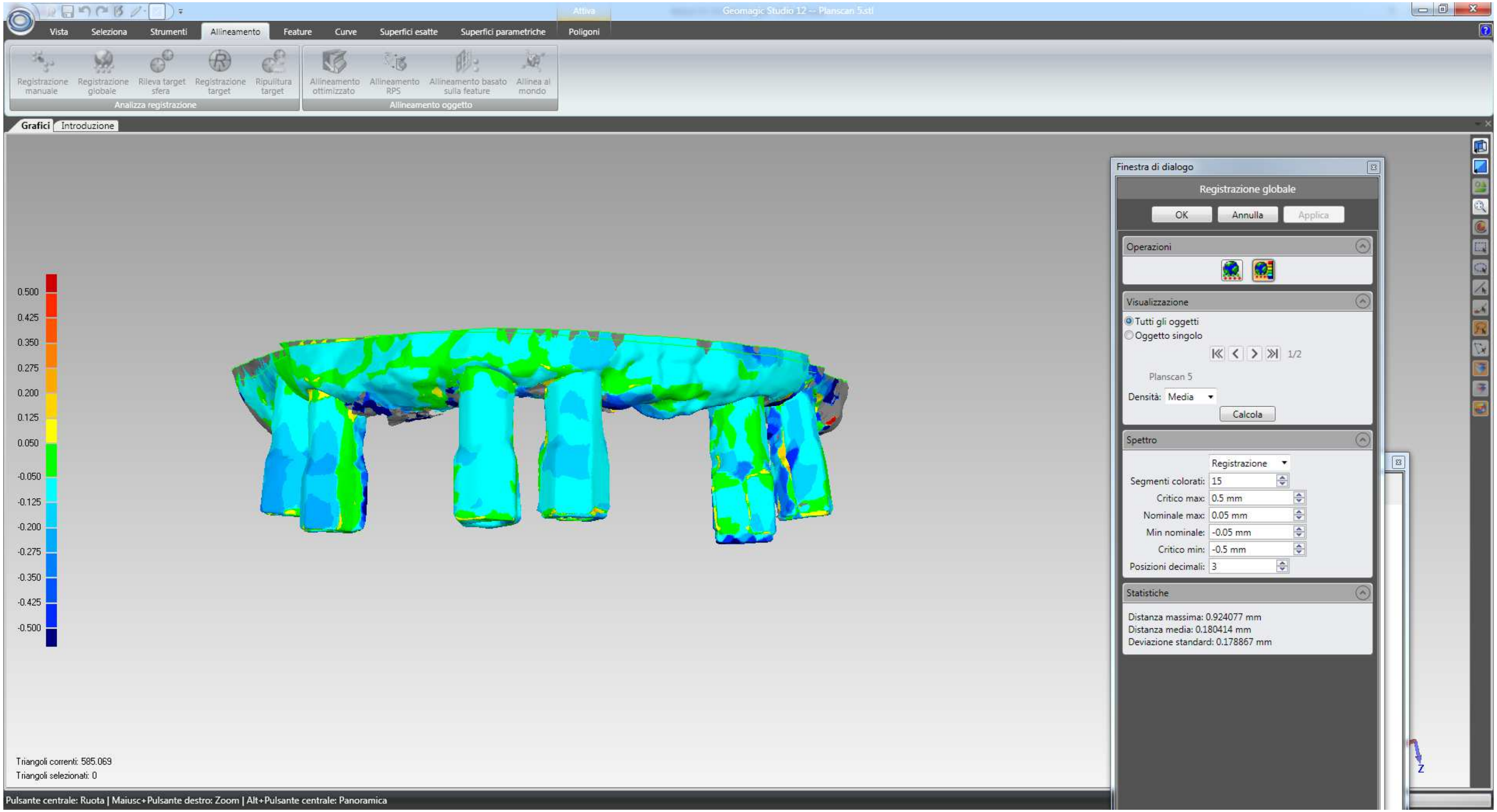

Plane 5 vs Plane 4

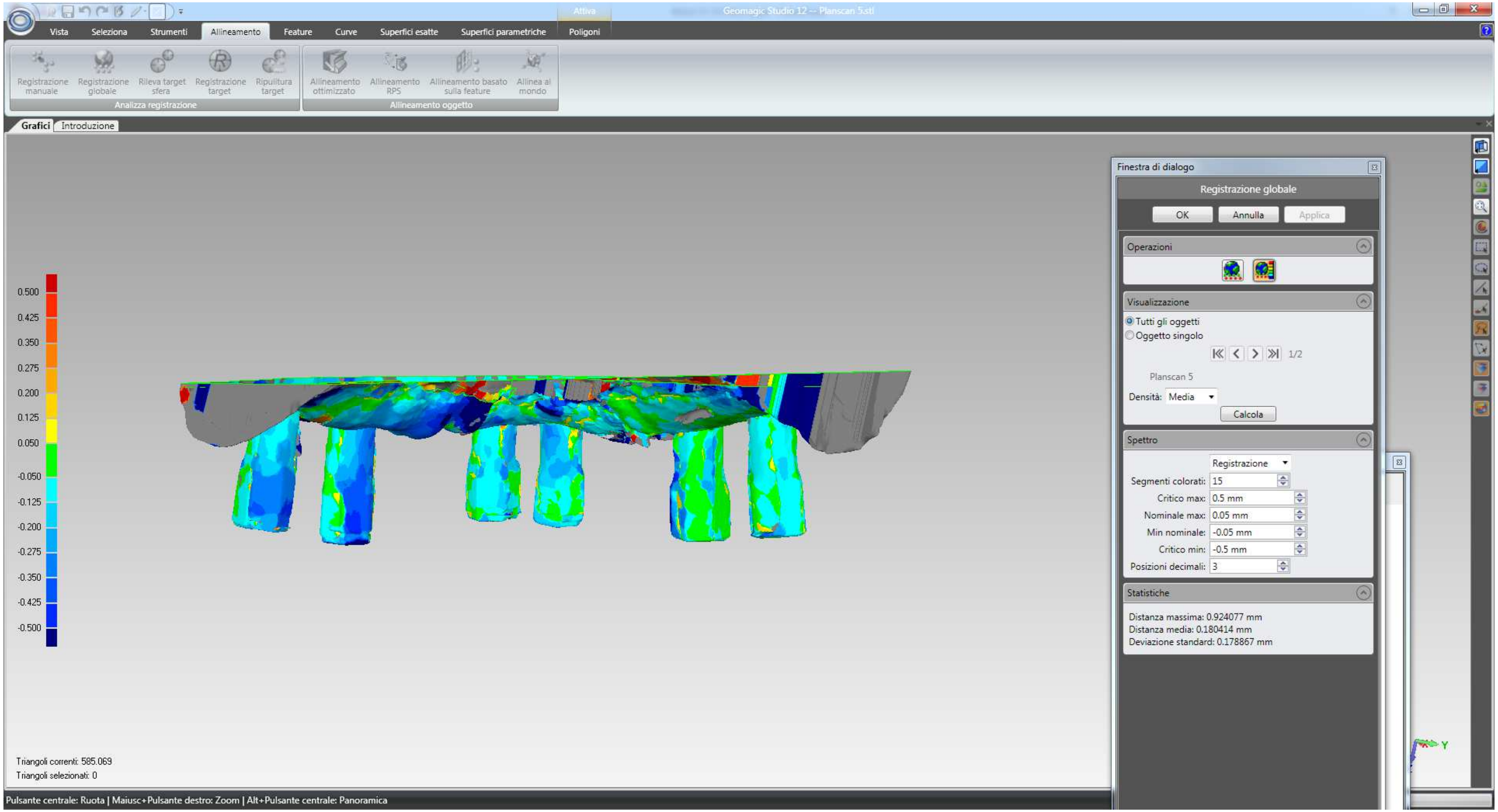

Plane 5 vs Plane 4

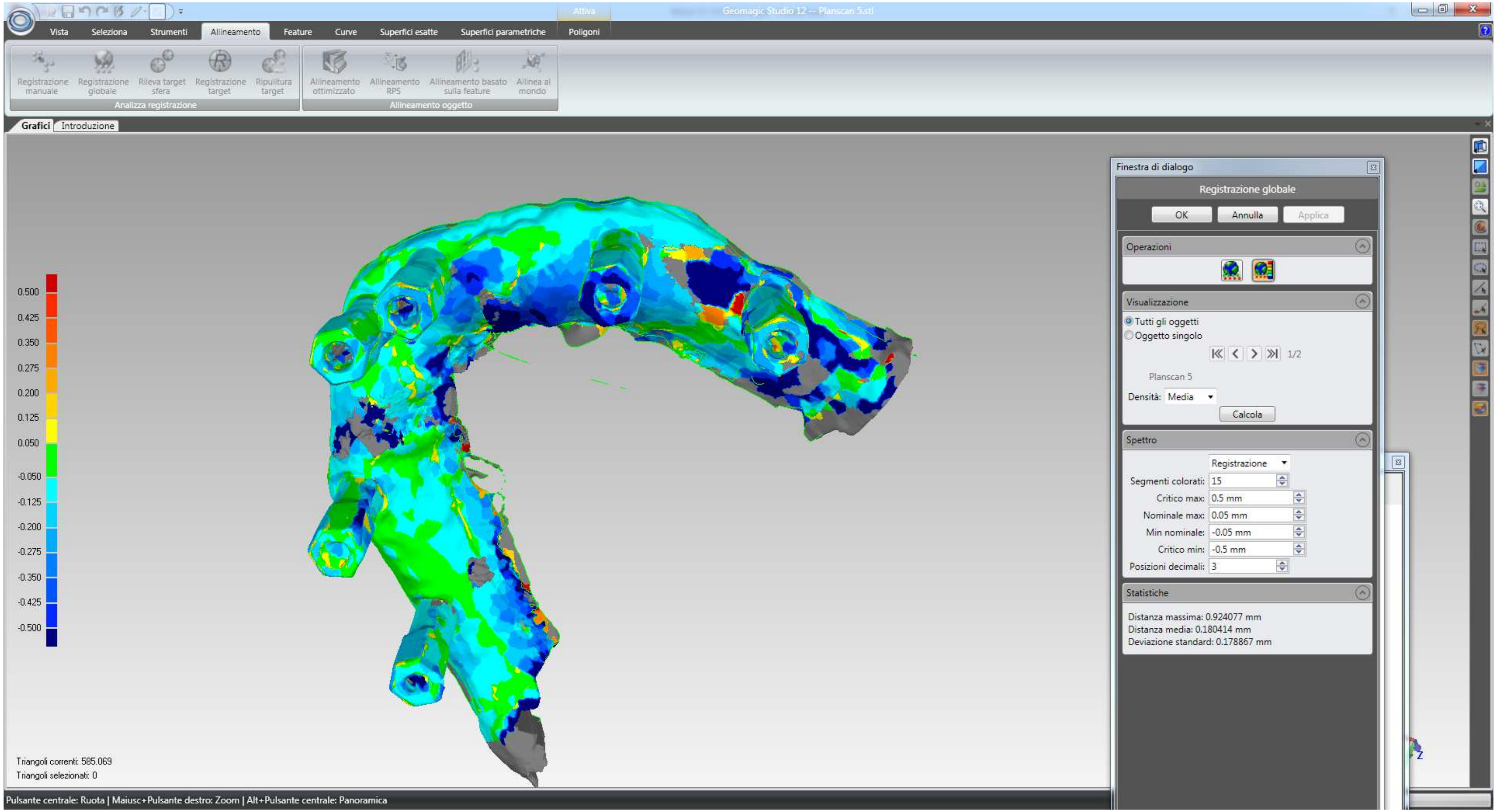

Plane 1 vs Plane 4

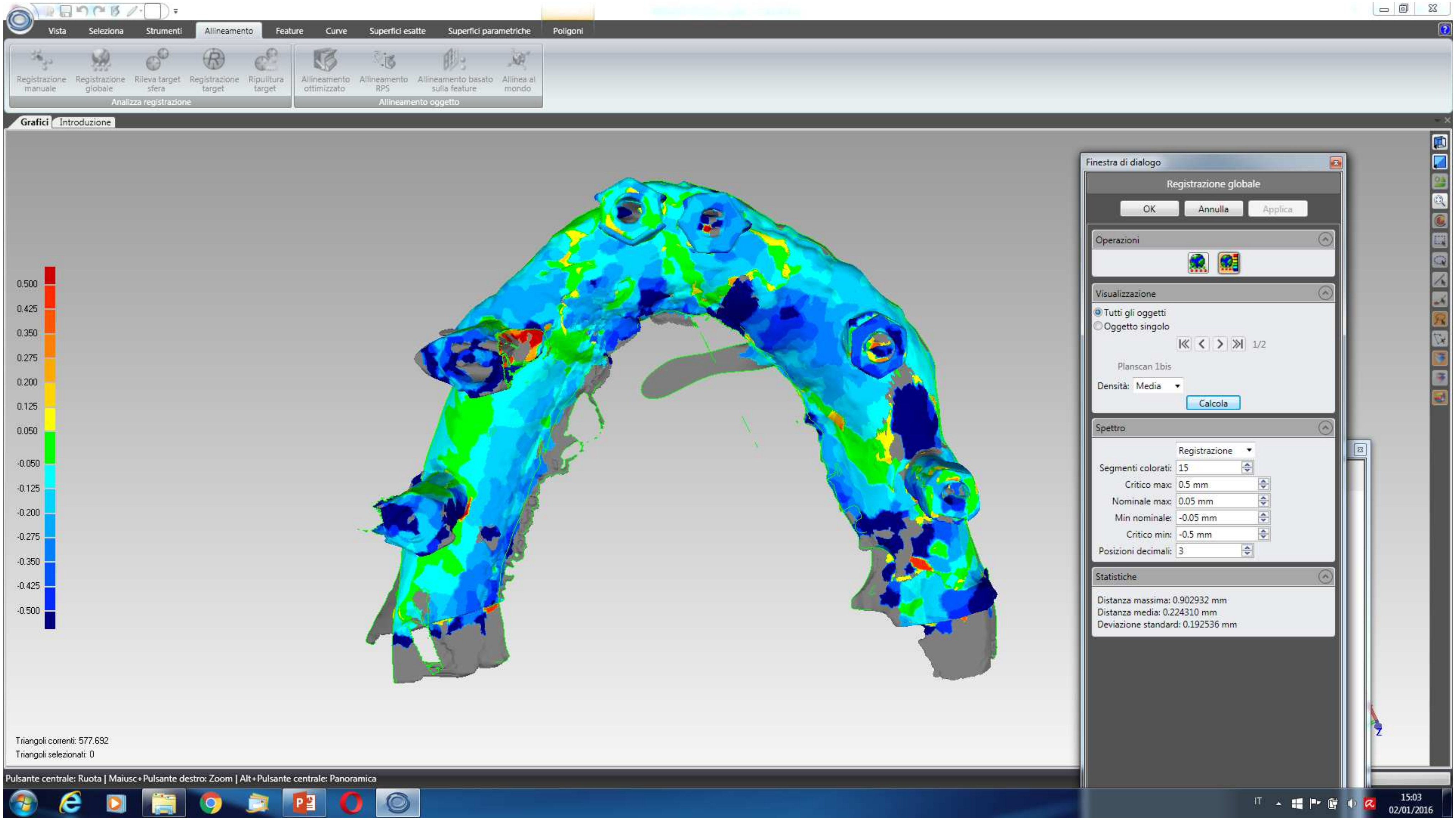

Plane 1 vs Plane 4

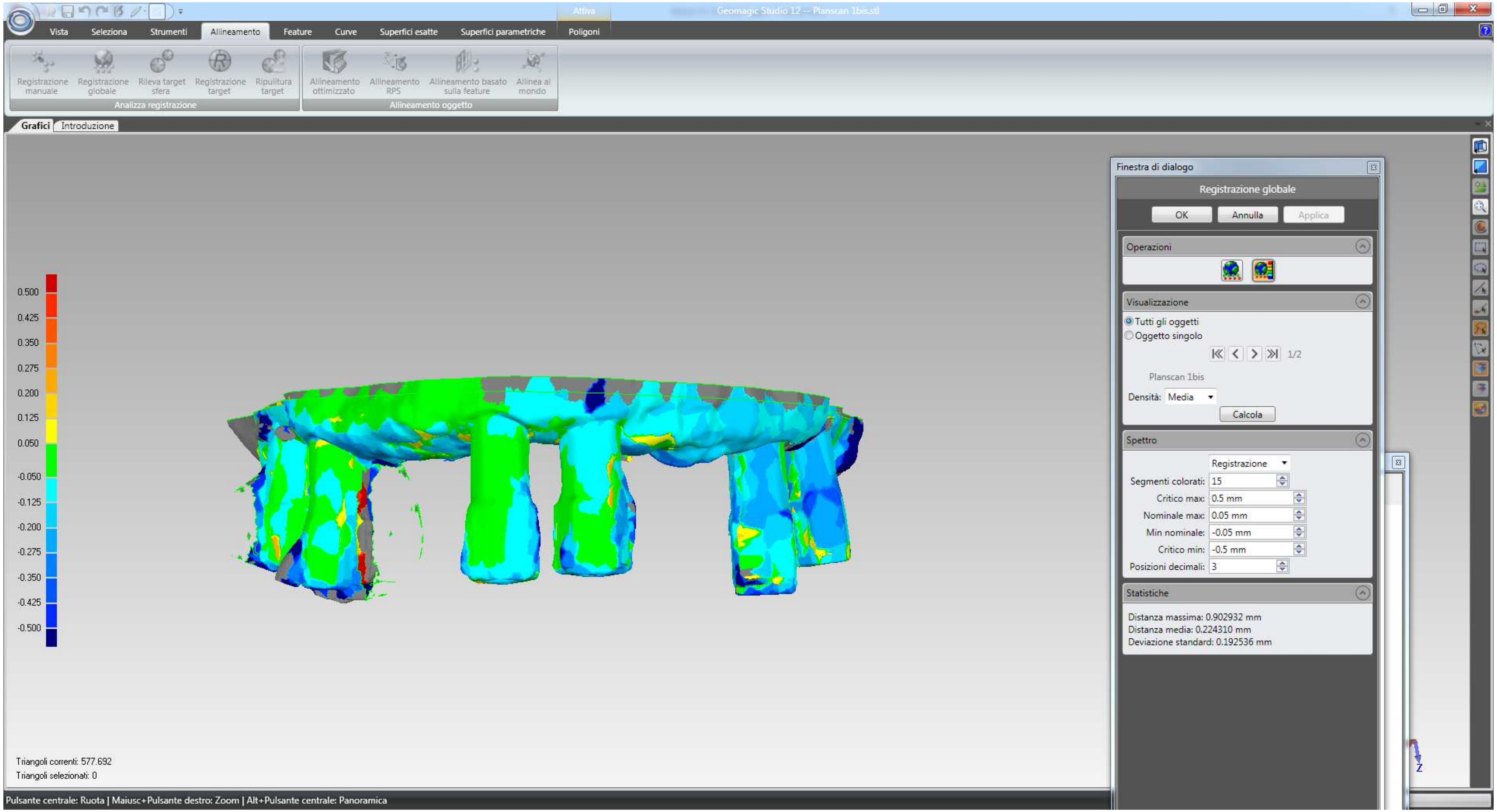

Plane 1 vs Plane 4

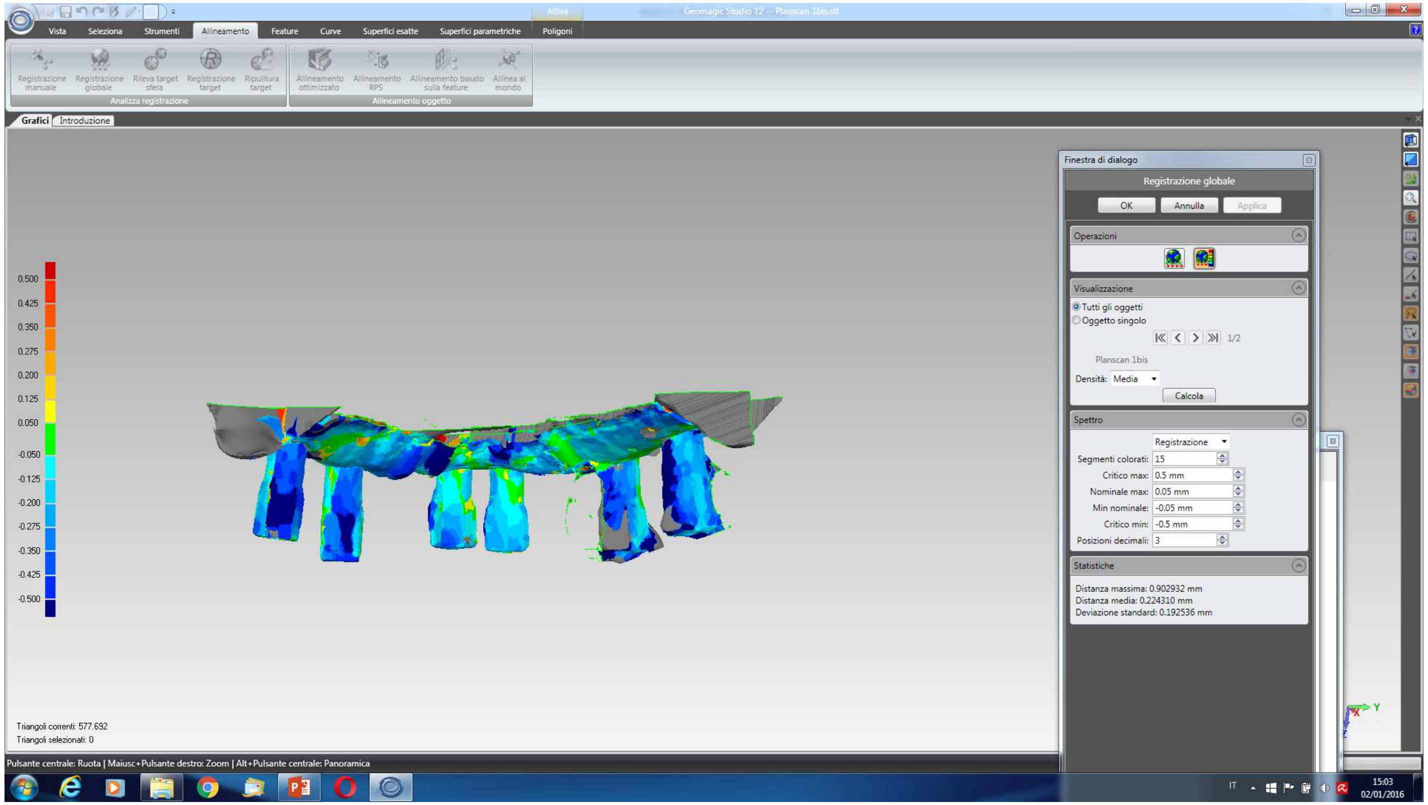

Plane 1 vs Plane 4

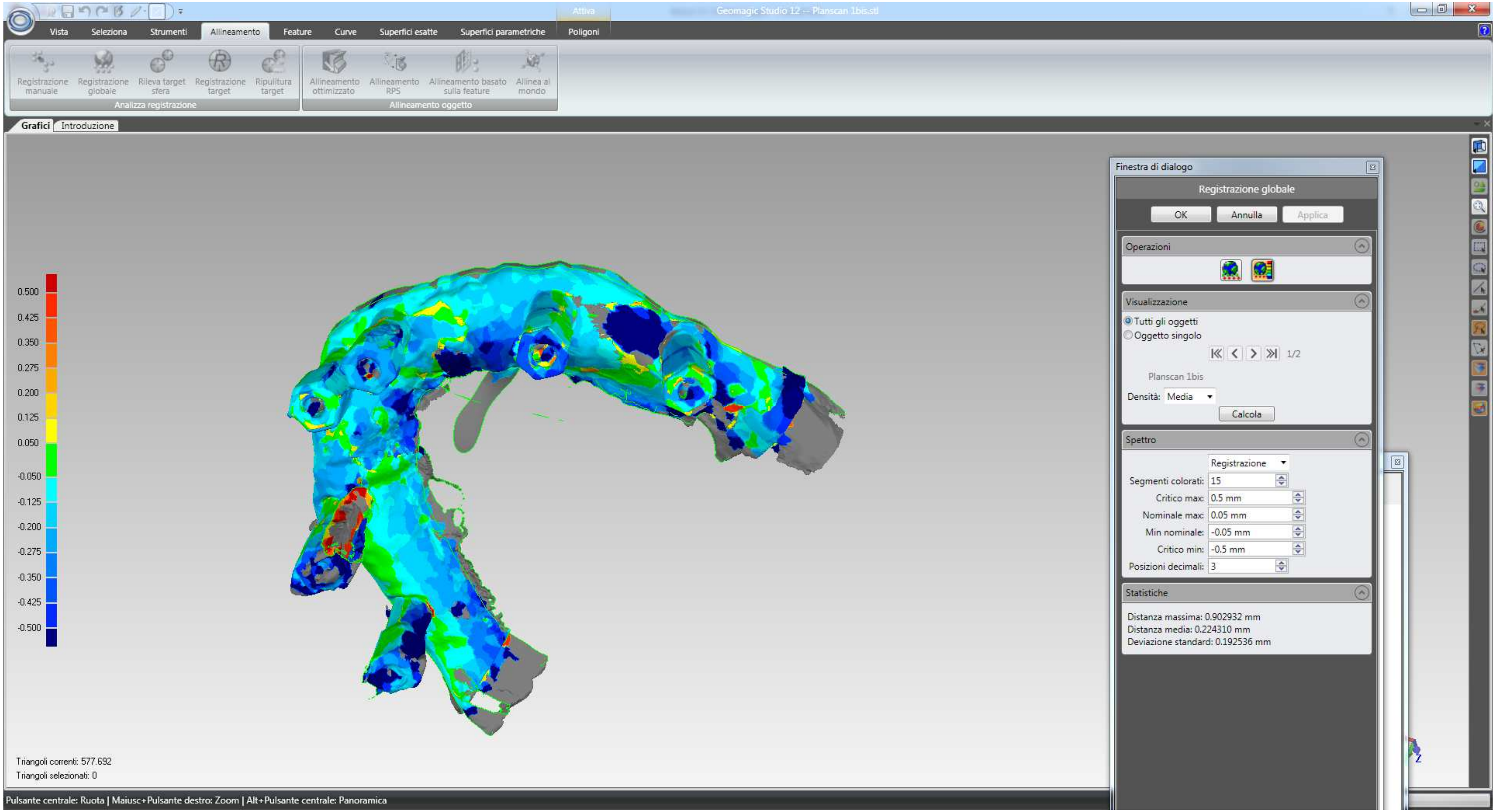

General precision of Planmeca scanner in the totally edentulous model

	Mean distance	SD	Maximum distance
Plane 5 vs Plane 1	0.179	0.172	0.926
Plane 5 vs Plane 2	0.216	0.183	0.927
Plane 5 vs Plane 3	0.222	0.203	0.920
Plane 5 vs Plane 4	0.180	0.178	0.924
Plane 1 vs Plane 4	0.224	0.192	0.902

Overall Plane general precision: 0.204 (0.022)

Overall Care general accuracy: 0.063 (0.007)
Overall Trios general accuracy: 0.071 (0.026)
Overall Mht general accuracy: 0.103 (0.026)
Overall Plane general accuracy: 0.253 (0.013)

Overall Care general precision: 0.055 (0.010)
Overall Trios general precision: 0.067 (0.032)
Overall Mht general precision: 0.112 (0.022)
Overall Plane general precision: 0.204 (0.022)

OVERALL RESULTS OF GENERAL TRUENESS AND GENERAL PRECISION
FOR THE FOUR DIFFERENT SCANNERS
IN THE TOTALLY EDENTULOUS MODEL

LOCAL TRIOS ACCURACY LINEAR MEASUREMENTS

Misure lineari su Trios	Imetric	Trios1	Trios2	Trios3	Trios 4	Trios 5	Media Trios	Errore medio lineare Trios
S1-S2	13.807	14.002	13.897	13.640	13.822	13.799	13.832 (0.133)	-0.025
S2-S3	18.737	18.753	18.691	18.776	18.664	18.753	18.727 (0.047)	0.01
S1-S3	31.159	31.276	31.293	31.133	31.220	31.163	31.217 (0.069)	-0.058
S3-S4	8.218	8.159	8.194	8.043	8.057	8.160	8.122 (0.067)	0.096
S4-S5	19.913	19.996	19.831	19.875	19.891	19.934	19.905 (0.062)	0.008
S5-S6	16.351	16.059	16.199	16.091	16.059	16.062	16.094 (0.060)	0.257
S4-S6	34.858	34.961	34.785	34.798	34.737	34.841	34.824 (0.084)	0.034

Misure angolari su Netfab	Imetric	Trios 1	Trios 2	Trios 3	Trios 4	Trios 5	Media Trios	Errore medio
S1-S2-S3	147.24°	147.50°	146.98°	148.52°	148.03°	147.55°	147.71° (0.58)	- 0.47°
S1-S3-S5	102.05°	102.41°	101.83°	101.73°	102.07°	101.93°	101.99° (0.26)	0.06°
S4-S5-S6	149.74°	150.37°	149.77°	149.36°	149.96°	149.70°	149.83° (0.37)	- 0.09°
S1-S3-S6	85.52°	85.99°	85.16°	85.20°	85.52°	85.77°	85.52° (0.35)	0.00°

LOCAL TRIOS ACCURACY ANGOLAR MEASUREMENTS ed errore medio angolare

LOCAL ZFX ACCURACY LINEAR MEASUREMENTS

Misure lineari su Zfx	Imetric	Zfx 1	Zfx 2	Zfx 3	Zfx 4	Zfx 5	Media Zfx	Errore medio lineare Zfx
S1-S2	13.807	14.023	14.004	13.967	14.316	14.068	14.075 (0.139)	-0.268
S2-S3	18.737	18.477	18.731	18.898	18.554	18.684	18.668 (0.163)	0,069
S1-S3	31.159	31.087	31.406	31.433	31.500	31.250	31.335 (0.166)	-0.176
S3-S4	8.218	8.199	8.398	8.292	8.288	8.351	8.305 (0.074)	-0.087
S4-S5	19.913	20.178	20.116	20.196	19.966	20.022	20.095 (0.099)	-0.182
S5-S6	16.351	16.320	16.123	16.385	16.276	16.057	16.232 (0.137)	0.119
S4-S6	34.858	35.312	35.142	35.498	35.155	35.124	35.246 (0.159)	-0.388

Misure angolari su Netfab	Imetric	Zfx 1	Zfx 2	Zfx 3	Zfx 4	Zfx 5	Media Zfx	Errore medio angolare
S1-S2-S3	147.24°	146.77°	147.38°	147.58°	146.76°	146.32°	146.96° (0.51)	0.28°
S1-S3-S5	102.05°	101.07°	100.78°	102.35°	102.43°	101.02°	101.53° (0.79)	0.52°
S4-S5-S6	149.74°	150.45°	150.46°	150.04°	149.59°	150.73°	150.25° (0.44)	-0.51°
S1-S3-S6	85.52°	84.84°	84.45°	86.14°	85.90°	85.20°	85.30° (0.70)	0.22°

LOCAL ZFX ACCURACY ANGOLAR MEASUREMENTS ed errore medio angolare

LOCAL CARE ACCURACY LINEAR MEASUREMENTS

Misure lineari su Care	Imetric	Care 1	Care 2	Care 3	Care 4	Care 5	Media Care	Errore medio lineare Care
S1-S2	13.807	13.732	13.884	13.638	13.893	14.077	13.844 (0.168)	-0.037
S2-S3	18.737	18.638	18.173	18.351	18.485	18.398	18.409 (0.171)	0.328
S1-S3	31.159	31.095	30.845	30.804	31.049	31.122	30.983 (0.147)	0.176
S3-S4	8.218	8.131	8.315	8.469	8.196	8.322	8.286 (0.130)	-0.068
S4-S5	19.913	20.148	19.658	19.363	19.879	19.961	19.801 (0.301)	0.112
S5-S6	16.351	16.154	16.194	16.137	16.246	16.047	16.155 (0.073)	0.196
S4-S6	34.858	34.996	34.745	34.173	34.900	34.848	34.732 (0.325)	0.126

LOCAL CARE ACCURACY ANGOLAR MEASUREMENTS
ed errore medio angolare

Misure angolari su Netfab	Imetric	Care 1	Care 2	Care 3	Care 4	Care 5	Media Care	Errore medio angolare
S1-S2-S3	147.24°	147.68°	149.92°	148.82°	147.60°	147.69°	148.34° (1.01)	-1.1°
S1-S3-S5	102.05°	101.78°	101.49°	101.59°	101.82°	102.34°	101.80° (0.32)	0.25°
S4-S5-S6	149.74°	149.17°	149.86°	147.19°	149.14°	149.37°	148.94° (1.02)	0.8°
S1-S3-S6	85.52°	84.96°	85.23°	85.22°	85.44°	85.54°	85.27° (0.22)	0.25°

LOCAL PLANE ACCURACY LINEAR MEASUREMENTS

Misure lineari su Geomagic	Imetric	Plan 1	Plan 2	Plan 3	Plan 4	Plan 5	Media Plan	Errore medio lineare Plan
S1-S2	13.807	13.849	13.933	13.611	13.746	14.008	13.829 (0.156)	- 0.022
S2-S3	18.737	18.514	18.304	18.214	18.245	18.195	18.294 (0.129)	0.443
S1-S3	31.159	31.146	30.969	30.703	30.890	30.937	30.929 (0.159)	0.23
S3-S4	8.218	8.016	8.205	8.003	8.244	8.164	8.126 (0.110)	0.092
S4-S5	19.913	20.123	19.927	19.796	19.784	19.716	19.869 (0.161)	0.044
S5-S6	16.351	15.760	15.920	15.977	16.060	15.927	15.928 (0.109)	0.423
S4-S6	34.858	34.927	34.870	34.785	34.835	34.732	34.829 (0.075)	0.029

LOCAL PLANE ACCURACY ANGOLAR MEASUREMENTS ed errore medio angolare

Misure angolari su Netfab	Imetric	Plan 1	Plan 2	Plan 3	Plan 4	Plan 5	Media Plan	Errore medio angolare
S1-S2-S3	147.24°	149.49°	147.52°	149.20°	148.87°	148.15°	148.64° (0.80)	- 1.4°
S1-S3-S5	102.05°	104.78°	103.94°	106.46°	104.82°	104.92°	104.98° (0.91)	- 2.93°
S4-S5-S6	149.74°	149.53°	150.74°	151.88°	151.09°	151.04°	150.85° (0.85)	- 1.11°
S1-S3-S6	85.52°	87.54°	88.93°	91.19°	89.08°	89.07°	89.16° (1.30)	- 3.64°

OVERALL RESULTS OF LOCAL TRUENESS FOR THE FOUR DIFFERENT SCANNERS IN THE TOTALLY EDENTULOUS MODEL

Accuratezza locale lineare errore medio

Misure lineari	Trios	Zfx	Care	Plan
S1-S2	-0.025	-0.268	-0.037	- 0.022
S2-S3	0.01	0.069	0.328	0.443
S1-S3	-0.058	-0.176	0.176	0.23
S3-S4	0.096	-0.087	-0.068	0.092
S4-S5	0.008	-0.182	0.112	0.044
S5-S6	0.257	0.119	0.196	0.423
S4-S6	0.034	-0.388	0.126	0.029

Trios errore medio assoluto lineare 0.069 (0.088)
Care errore medio assoluto lineare 0.149 (0.096)
Zfx errore medio assoluto lineare 0.184 (0.112)
Plane errore medio assoluto lineare 0.183 (0.184)

Accuratezza locale angolare errore medio

	Trios	Zfx	Care	Plane
S1-S2-S3	- 0.47°	0.28°	-1.1°	- 1.4°
S1-S3-S5	0.06°	0.52°	0.25°	- 2.93°
S4-S5-S6	- 0.09°	-0.51°	0.8°	- 1.11°
S1-S3-S6	0.00°	0.22°	0.25°	- 3.64°

Trios errore medio assoluto angolare 0.15° (0.21°)
Zfx errore medio assoluto angolare 0.38° (0.15°)
Care errore medio assoluto 0.6° (0.42°)
Plane errore medio angolare 2.27° (1.21°)
